# Dual-Specificity Anti-sigma Factor Reinforces Control of Cell-Type Specific Gene Expression in *Bacillus subtilis*

**DOI:** 10.1371/journal.pgen.1005104

**Published:** 2015-04-02

**Authors:** Mónica Serrano, JinXin Gao, João Bota, Ashley R. Bate, Jeffrey Meisner, Patrick Eichenberger, Charles P. Moran, Adriano O. Henriques

**Affiliations:** 1 Instituto de Tecnologia Química e Biológica António Xavier, Universidade Nova de Lisboa, Estação Agronómica Nacional, Oeiras, Portugal; 2 Emory University School of Medicine, Atlanta, Georgia, United States of America; 3 New York University, Department of Biology, New York, New York, United States of America; Indiana University, UNITED STATES

## Abstract

Gene expression during spore development in *Bacillus subtilis* is controlled by cell type-specific RNA polymerase sigma factors. σ^F^and σ^E^ control early stages of development in the forespore and the mother cell, respectively. When, at an intermediate stage in development, the mother cell engulfs the forespore, σ^F^ is replaced by σ^G^ and σ^E^ is replaced by σ^K^. The anti-sigma factor *CsfB* is produced under the control of σ^F^ and binds to and inhibits the auto-regulatory σ^G^, but not σ^F^. A position in region 2.1, occupied by an asparagine in σ^G^ and by a glutamate in ο^F^, is sufficient for CsfB discrimination of the two sigmas, and allows it to delay the early to late switch in forespore gene expression. We now show that following engulfment completion, *csfB* is switched on in the mother cell under the control of σ^K^ and that CsfB binds to and inhibits σ^E^ but not σ^K^, possibly to facilitate the switch from early to late gene expression. We show that a position in region 2.3 occupied by a conserved asparagine in σ^E^ and by a conserved glutamate in σ^K^ suffices for discrimination by CsfB. We also show that CsfB prevents activation of σ^G^ in the mother cell and the premature σ^G^-dependent activation of σ^K^. Thus, CsfB establishes negative feedback loops that curtail the activity of σ^E^ and prevent the ectopic activation of σ^G^ in the mother cell. The capacity of CsfB to directly block σ^E^ activity may also explain how CsfB plays a role as one of the several mechanisms that prevent σ^E^ activation in the forespore. Thus the capacity of CsfB to differentiate between the highly similar σ^F^/σ^G^ and σ^E^/σ^K^ pairs allows it to rinforce the cell-type specificity of these sigma factors and the transition from early to late development in B. subtilis, and possibly in all sporeformers that encode a CsfB orthologue.

## Author Summary

Precise temporal and cell-type specific regulation of gene expression is required for development of differentiated cells even in simple organisms. Endospore development by the bacterium *Bacillus subtilis* involves only two types of differentiated cells, a forespore that develops into the endospore, and a mother cell that nurtures the developing endospore. During development temporal and cell-type specific regulation of gene expression is controlled by transcription factors called sigma factors (σ). An anti-sigma factor known as CsfB binds to σ^G^ to prevent its premature activity in the forespore. We found that CsfB is also expressed in the mother cell where it blocks ectopic activity of σ^G^, and blocks the activity σ^E^ to allow σ^K^ to take over control of gene expression during the final stages of development. Our finding that CsfB directly blocks σ^E^ activity also explains how CsfB plays a role in preventing ectopic activity of σ^E^ in the forespore. Remarkably, each of the major roles of CsfB, (*i*.*e*., control of ectopic σ^G^ and σ^E^ activities, and the temporal limitation of σ^E^ activity) is also accomplished by redundant regulatory processes. This redundancy reinforces control of key regulatory steps to insure reliability and stability of the developmental process.

## Introduction

Developmental transcription networks underlie all cellular differentiation processes. These networks usually integrate a variety of environmental and cellular inputs to activate regulators such as transcription factors that control the expression of cell type-specific genes [1,2]. Superimposed onto these transcription networks, are protein-protein interaction, signal transduction and metabolic networks [3]. Overall, the combination of these different networks ensures the timely production of proteins and other components essential for the morphogenesis of newborn cells. Ultimately, a complete understanding of cellular differentiation requires detailed knowledge of how the circuitry of transcriptional regulators influences global gene expression in space and time to drive cell morphogenesis at sequential stages of development.

**Fig 1:**
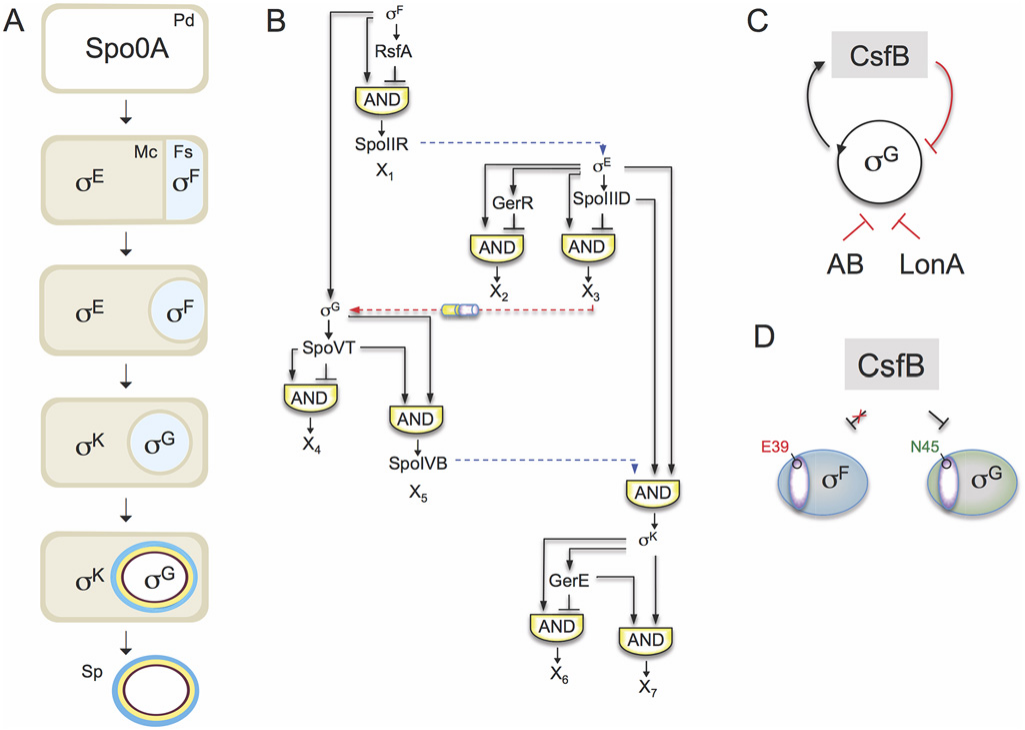
The sporulation network and the action of CsfB on σ^G^. **A**: the main morphological stages of sporulation are represented, with the main regulatory proteins active in the indicated cells. Pd, pre-divisional cell; Mc, mother cell; Fs, forespore; Sp, mature spore. **B**: organization of the transcriptional network of sporulation. The broken blue lines represent cell-cell signalling pathways. First, σ^F^ drives production of a protein, SpoIIR, secreted to the intermembrane space. SpoIIR then activates a membrane-embedded protease that triggers the proteolytic activation of pro-σ^E^ [18]. σ^E^ and σ^F^ activity are required for the assembly of a cell-cell secretion system (the SpoIIQ-SpoIIIAH channel in the figure) that, following engulfment completion, allows the mother cell to nourish the isolated forespore, thus enabling continued macromoelcualr synthesis and activation of the σ^G^ auto-regulatory loop [67]. Lastly, σ^G^ controls production of a signaling protein, SpoIVB, secreted into the intermembrane space that activates the machinery responsible for the proteolytical activation of σ^K^ [18]. The negative feedback loop through which σ^K^ limits production of σ^E^ is omitted for simplicity. **C**: the panel represents the composite negative feedback loop that operates in pre-divisional cells, and possibly also in the forespore prior to engulfment completion, to prevent activation of the σ^G^ positive auto-regulatory loop. Transcriptional and protein-protein interactions are shown in black and red, respectively. **D**: a single amino acid residue in region 2.1 (purple sector) allows CsfB to discriminate between the highly similar forespore sigma factors σ^F^ and σ^G^: N45 of *B*. *subtilis* σ^G^ allows binding by CsfB, whereas a glutamate at the same position precludes binding. Conversely, a glutamate at the homologous position of σ^F^ (E39) impedes binding by CsfB whereas an asparagine at the same position is sufficient for binding. N45 and E39 are conserved among *Bacillus* orthologues of σ^G^ and σ^F^.

Spore formation in the bacterium *Bacillus subtilis* is an example of a prokaryotic cell differentiation process. At the onset of sporulation, triggered by severe nutrient scarcity, the rod-shaped cell divides close to one of its poles producing a small forespore, the future spore, and a larger mother cell (Fig. 1A). The mother cell nurtures development of the forespore, but undergoes autolysis to release the mature spore at the end of the process. Soon after asymmetric division, the mother cell engulfs the forespore, which becomes isolated from the external medium and separated from the mother cell cytoplasm by a double membrane and an intermembrane space. Following engulfment completion, gene expression in the mother cell drives the last stages of spore maturation by promoting the assembly of concentric protective structures. In parallel, gene expression in the forespore prepares the future spore for dormancy.

The sporulation regulatory network includes four RNA polymerase sigma subunits that are activated in a cell type-specific manner and define a regulatory cascade that constitutes the core of the transcription network. σ^F^ and σ^E^ control early stages in development in the forespore and in the mother cell, respectively. At late stages of development, *i*.*e*., post-engulfment, σ^F^ is replaced by σ^G^, and σ^E^ is replaced by σ^K^ (Fig. 1A). The genes for the cell type-specific sigma factors are part of a genomic signature for sporulation [4,5,6,7]. A second level of regulation results from the action of additional transcription factors, three in the mother cell (SpoIIID, GerR, and GerE), and two in the forespore (RsfA and SpoVT), which are less conserved among spore-formers. The combination of the primary regulators (*i*.*e*., the sporulation sigma factors) and the secondary regulators (ancillary transcription factors) organizes the two cell-specific lines of gene expression into a series of interlocked type-1 coherent and incoherent feed-forward loops (FFLs) [8,9](Fig. 1B). Coherent type-1 FFLs are used as persistence detectors whereas incoherent type-1 FFL generate pulses of gene expression (reviewed by [3]).

The first mother cell-specific sigma factor, σ^E^, has a central role in controlling engulfment. Together with the ancillary factor SpoIIID, σ^E^ also turns on the genes required for the synthesis of pro-σ^K^ and the machinery that triggers proteolytic activation of σ^K^. In parallel, SpoIIID and GerR, both acting as repressors, switch off two classes of genes initially activated by σ^E^. Thus, the early transcription network in the mother cell includes a type-1 coherent FFL (with SpoIIID as an activator), leading to the production of σ^K^, and two type-1 incoherent FFLs (with SpoIIID and GerR as negative regulators). Both SpoIIID and GerR are therefore critical to the early to late developmental transition characterized by the activation of σ^K^ and the inhibition of a large fraction of the σ^E^ regulon in the mother cell [9]. Once active, σ^K^ triggers a negative feedback loop that lowers production of σ^E^ and decreases the levels of SpoIIID [10,11,12].

Superimposed onto the transcriptional network are several cell-cell signaling pathways that operate at critical stages of morphogenesis, across the forespore membranes, to allow activation of the next sigma factor in the cascade, in the adjacent cell (Fig. 1B). The requirement for σ^G^ for σ^K^ activity is an example of such a pathway. σ^F^ drives the initial transcription of the gene for σ^G^ in the forespore [13], but the main period of σ^G^ activity, dependent on activation of an auto-regulatory loop, only begins after engulfment completion [14,15,16,17]. σ^G^ then controls production of a signaling protein, SpoIVB, which is secreted into the intermembrane space and activates the machinery responsible for the proteolytical activation of σ^K^ [18].

Another level of control of sigma factor activity is through the inhibitory action of anti-sigma factors. CsfB (also called Gin, for inhibitor of sigma G) is a Zn^2+^-containing anti-sigma factor that inhibits σ^G^ [19,20,21,22]. CsfB is part of a composite negative feedback loop that together with another anti-sigma factor, SpoIIAB, and the LonA protease, prevents activation of the auto-regulatory σ^G^ in pre-divisional cells (Fig. 1C) [20]. After the onset of sporulation CsfB is also produced in the forespore under σ^F^ control [23], and has a role in delaying the onset of σ^G^ activity until engulfment completion [14,24]. Importantly, while σ^F^ and σ^G^ are highly similar proteins, σ^F^ itself is refractory to the action of CsfB. The basis for this selectivity can be traced to a single asparagine residue in a conserved β´-interacting surface of σ^G^ [20]. Likewise, a conserved glutamate residue of σ^F^ is important for resistance (Fig. 1D). The anti-σ^G^ activity of CsfB, and the delayed transcription of the gene encoding σ^G^ relative to other σ^F^-dependent genes are redundant mechanisms ensuring that the onset of σ^G^ activity begins at the appropriate time, possibly to coincide with engulfment completion. Accurate temporal control of σ^G^ activity is important, because it leads to the activation of σ^K^ [18] and premature activity of σ^K^ interferes with spore morphogenesis [25]. Timely activation of σ^K^ is important, as it ensures that the final stages in the assembly of the spore surface structures only initiate after the forespore is engulfed [18]. The σ^G^-dependent production of SpoIVB, together with delayed transcription of genes required for pro-σ^K^ synthesis and processing, which require both σ^E^ and SpoIIID (Fig. 1B; above), effectively couples pro-σ^K^activation to engulfment completion. In total, timely activation of the cell type-specific sigma factors enforces the directionality and fidelity of the morphogenetic process [7,18].

This works focuses on an additional role of the anti-sigma factor CsfB in controlling cell type-specific gene expression during spore development. We show that in addition to its previously characterized functions in the forespore and pre-divisional cells, the anti-sigma factor CsfB also plays a role in the mother cell where its synthesis is activated under the control of σ^K^. We show that CsfB binds to and inhibits σ^E^ *in vitro* and *in vivo*, while σ^K^ is resistant to CsfB. A single residue in conserved region 2.3 of the sigma subunit is sufficient for the discrimination by CsfB and swapping this specificity interferes with the temporal progression of the mother cell line of gene expression. Thus, CsfB is part of a negative feedback loop that acts to limit the activity of σ^E^ following engulfment completion. Furthermore, we show that CsfB is also involved in preventing ectopic activity of σ^G^ in the mother cell. Therefore, CsfB is intricately connected to both cell-specific lines of gene expression to enforce the cell-type specific action of σ^G^ and the early to late developmental transition in the two cell types undergoing sporulation.

## Results

### Following engulfment completion, expression of *csfB* is turned on in the mother cell from a σ^K^-dependent promoter

**Fig 2:**
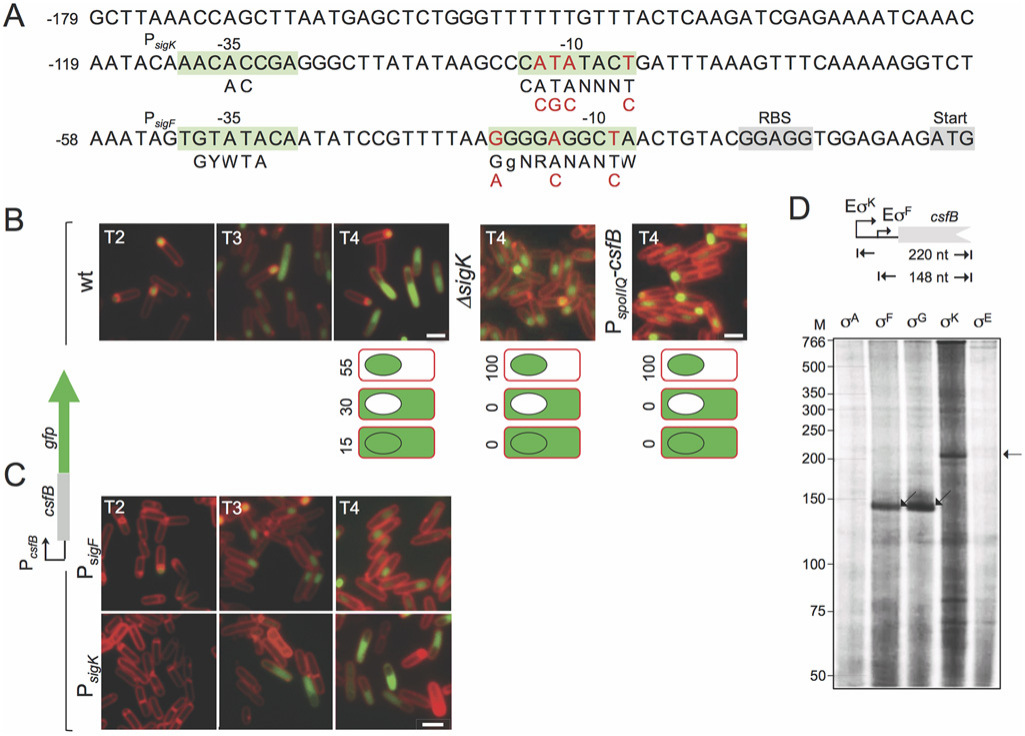
Expression of *csfB* in sporulating cells. **A**: the panel represents the regulatory region of the *csfB* gene, with the -35 and -10 elements of putative σ^F^- or σ^K^-dependent promoters. The consensus sequences for promoter recognition by σ^F^or σ^K^are represented below the promoter sequence (R is A or G; W is A or T; Y is C or T; N, is any base) [8,9]. The point mutations introduced in the putative σ^F^- or σ^K^-dependent promoters are indicated in red. The ribosome-binding site (RBS) and start codons are also represented, for reference. **B** and **C**: the localization of a CsfB-GFP fusion protein, expressed from the *amyE* locus in a *csfB* mutant background was monitored by fluorescence microscopy at the indicated times (in hours) after the onset of sporulation in re-suspension medium, in cultures of the following strains: an otherwise wild type, a Δ*sigK* mutant, a strain expressing CsfB-GFP from the forespore-specific P_*spoIIQ*_ promoter (in B); strains bearing the point mutations indicated in A, in the σ^F^ (P_*sigK*_) or σ^K^ promoters (P_*sigF*_) (in C). Cells were stained with the membrane dye FM4–64 prior to fluorescence microscopy. Scale bars, 1 μm. The cartoons in B represent the percentage of cells with GFP fluorescence in the coloured compartment at hour 4 of sporulation in re-suspension medium. **D**: *in vitro* transcription reactions using RNA polymerase containing the indicated *B*. *subtilis* sigma factors, and a PCR template containing the *csfB* promoter. Products of about 148 or 220 nucleotides were expected for transcripts initiating from the putative σ^F^- and σ^K^-dependent promoters, as represented.

During sporulation, expression of *csfB* in the forespore is controlled by σ^F^ and occurs prior to engulfment completion [20,23]. Accordingly, sequences centered at about 26 bp (GTATA) and 48 bp (GGGGAGGCTA) upstream of the *csfB* start codon match the consensus for σ^F^-controlled promoters [8] (Fig. 2A). Presumably, the same σ^F^-type promoter can also be recognized by σ^G^ in pre-divisional cells [8,20].

In addition, sequences matching the -10 (CATATACT) and -35 (AACACCGA) elements of the σ^K^ consensus binding sequence are present in the *csfB* regulatory region, upstream of the putative σ^F^ promoter [26] (Fig. 2A). This suggested to us that expression of *csfB* could also take place in the mother cell, at a later stage in development, after σ^K^ is activated [25]. To test this possibility, we first examined expression of a functional *csfB-gfp* fusion inserted at the non-essential *amyE* locus of a strain deleted for the native *csfB* gene [20]. Cells were induced to sporulate by resuspension in a nutrient poor medium and examined by phase contrast and fluorescence microscopy at different times after resuspension (which marks the beginning of sporulation under these culturing conditions). In a wild type background, expression of *csfB-gfp* was first detected in the forespore of sporulating cells (sporangia), two hours after the onset of sporulation, consistent with the time of activation of σ^F^ (Fig. 2B) [20]. However, by hour 3 of sporulation CsfB-GFP fluorescence was also observed in the mother cell of sporangia that had completed the process of forespore engulfment, as indicated by the loss of FM4–64 staining at the forespore membranes (Fig. 2B). At hour 4 of sporulation, 55% of sporangia exhibited a fluorescence signal restricted to the forespore, 30% fluoresced only in the mother cell, and 15% in both cell types (Fig. 2B). Moreover, the appearance of a CsfB-GFP signal in the mother cell seemed to occur concomitantly with a decrease in the forespore-specific fluorescence signal (Fig. 2B). By contrast, no CsfB-GFP fluorescence was seen in the mother cell of a *sigK* deletion mutant, even though these sporangia remained capable of completing engulfment (Fig. 2B). These results suggest that σ^K^ is responsible for the mother cell-specific accumulation of CsfB-GFP.

An alternative explanation, however, is that CsfB-GFP may be exported from the forespore to the mother cell upon engulfment completion, in an unindentified process that would require σ^K^ activity. To test this idea we replaced the native promoter sequences (P_*csfB*_) driving expression of the *csfB-gfp* fusion by P_*spoIIQ*_, a well-characterized forespore-specific, σ^F^-dependent promoter [22,27,28]. In this strain, accumulation of CsfB-GFP was detected in the forespore from hour 2 of sporulation onwards as expected, but was never observed in the mother cell even after σ^K^ had been activated (Fig. 2B). Thus, mother cell-specific transcription, and not transport from the forespore, is responsible for CsfB-GFP accumulation in the mother cell at late stages of development.

To more precisely delineate the respective contributions of the *csfB* promoters, we introduced point mutations in each of the σ^F^- and σ^K^-type putative -10 elements (highlighted in red in Fig. 2A) and examined production of CsfB-GFP during sporulation. In a strain bearing mutations in the putative σ^K^-10 element, in which *csfB-gfp* expression is presumably only driven by the σ^F^-dependent promoter (P_*sigF*_ in Fig. 2C), CsfB-GFP is restricted to the forespore. Conversely, when point mutations were introduced in the -10 region of the putative σ^F^-dependent promoter (P_*sigK*_ in Fig. 2C), CsfB-GFP only accumulates in the mother cell.

Immunoblot analysis of whole cell extracts of sporulating cells producing CsfB-GFP under the control of the wild type promoters revealed a steady increase in the accumulation of the fusion protein, between hour 2 and 5 of sporulation (S1A Fig). By contrast, when expression of the fusion was solely dependent on P_*sigF*_, the intensity of the CsfB-GFP band strongly decreased after hour 3, whereas when expression was driven exclusively from P_*sigK*_, CsfB-GFP accumulated only from hour 3 onwards (S1A Fig). These observations are consistent with the presumed windows of activity of each of the *csfB* promoters (Fig. 2A), as well as the fluorescence microscopy experiments described above (Fig. 2B and C). We also carried out experiments with a *lacZ* transcriptional fusion and similarly detected two periods of *csfB* expression, the first beginning around hour 2 of sporulation and the second around hour 3–4 (S1B Fig). The first period of β-galactosidase activity was not observed when expression of the fusion relied only on P_*sigK*_, whereas the second period was absent when expression of *csfB* was controlled by P_*sigF*_ (S1B Fig). These results are in line with the view that expression of *csfB* takes place at two developmental stages during sporulation, first in the forespore, under the control of σ^F^, and later in the mother cell, under the control of σ^K^.

Finally, we conducted *in vitro* transcription reactions in which core RNA polymerase, purified from *B*. *subtilis*, was reconstituted either with each of the four cell type-specific sporulation sigma factors or with the main sigma subunit, σ^A^. All σ factors were overproduced and purified from *E*. *coli* cells (S2A Fig; see also S1 Text). While the σ^A^ or σ^E^ forms of RNA polymerase did not initiate transcription from the *csfB* promoter region, a run-off product of 148 nucleotides was obtained with the σ^F^- and σ^G^-containing holoenzymes (Fig. 2D). The size of this transcript is consistent with the location of P_*sigF*_ and in line with the idea that σ^F^and σ^G^ utilize the same *csfB* promoter (Fig. 2A). In addition, a product of 220 nucleotides was obtained with the σ^K^-reconstituted holoenzyme (Fig. 2D), in agreement with the location of the predicted P_*sigK*_ (Fig. 2A). In all, our findings suggest that, following engulfment completion, expression of *csfB* is switched off in the forespore by an unknown mechanism and activated in the mother cell, under the direct control of σ^K^.

### CsfB modulates expression of the σ^E^ and σ^K^ regulons

In pre-divisional sporangia, σ^G^ activity is inhibited by a composite negative-feedback loop involving CsfB [20]. We have argued that the σ^F^-promoter of *csfB* is recognized and used by σ^G^ to achieve this mechanism of negative auto-regulation (Fig. 1C). Thus, inactivation of P_*sigF*_ should mimic the previously described activity of σ^G^ in pre-divisional cells of a *csfB* deletion mutant [20]. In support of this hypothesis, we found that P_*sigK*_ cells (carrying mutations in the σ^F^promoter) show activity of σ^G^ in pre-divisional cells, as measured by the expression of transcriptional fusions of *lacZ* and *cfp* to the σ^G^-dependent *sspE* promoter (S3 Fig; see also S1 Text). These results confirm that the σ^F^-type promoter of *csfB* is the one used by σ^G^ in pre-divisional cells.

**Fig 3:**
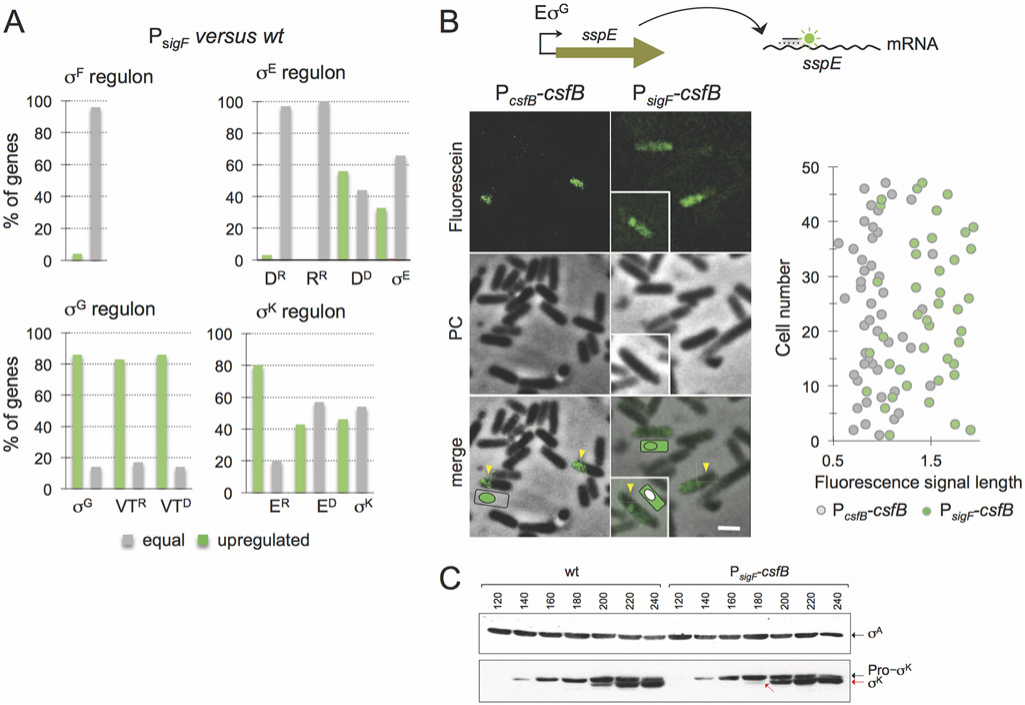
Role of CsfB in the mother cell. **A**: microarrays were used to assess the impact of mutational inactivation of the σ^G^-dependent promoter for the *csfB* gene (P_*sigF*_ strain) on sporulation-specific gene expression. The panel represents the percentage of genes in each of the sporulation-specific sigma regulons, whose expression was increased (green bars) or unaffected (grey bars) in the P_*sigF*_ mutant relative to the wild type. Genes, repressed (“R”) by or dependent (“D”) on the following ancillary transcription factors, are shown: D, SpoIIID; R, GerR; VT, SpoVT; E, GerE. **B**: the panel illustrates the results of a fluorescence *in situ* hybridization (FISH) experiment to localize the *sspE* mRNA, produced under σ^G^ control, in a wild type strain and in the P_*sigF*_ mutant. The mRNA was localized using a fluorescein-labelled anti-sense oligonucleotide and fluorescence microscopy. PC, phase contrast. The graph on the right shows the length (in μm) distribution of the fluorescence signal measured along the longitudinal axis of the cells. **C**: immunoblot analysis of pro-σ^K^ and σ^K^. Samples from cultures of the wild type and P_*sigF*_-*csfB* strain were collected at the indicated times (in minutes) after the induction of sporulation by re-suspension. Whole cell extracts were prepared, proteins (15 μg) electrophoretically resolved and immunobloted with anti-σ^K^ and anti-σ^A^ (as a loading control) antibodies. Arrows indicate the position of pro-σ^K^ (in black) and σ^K^ (red).

Next, we used DNA microarrays to examine sporulation-specific gene expression in the P_*sigF*_ strain, which does not express CsfB in the mother cell, in comparison to the wild type. The total RNA used for these studies was extracted and purified from cultures in resuspension medium at hour 3 of sporulation, *i*.*e*., shortly after *csfB* is turned on in the mother cell (above). We observed important changes in expression of several of the sporulation sigma factor regulons. Nevertheless, the expression of nearly all of the genes in the σ^F^regulon remained unchanged in the P_*sigF*_ strain versus the wild type (Fig. 3A; S3 Table); however, this does not include the group of forespore-expressed genes that are under the dual control of σ^F^and σ^G^ (see below, σ^G^ regulon). By contrast, a large fraction of the σ^E^-dependent genes was upregulated in the P_*sigF*_ strain relative to the wild type (Fig. 3A; S3 Table). Importantly, the σ^E^ regulon can be sub-divided into 4 groups: i) genes that rely on σ^E^ alone for expression, ii) those that require SpoIIID as a positive factor, iii) those that are repressed by SpoIIID, and iv), those that are repressed by GerR [9]. A closer inspection of the transcriptional profiling data revealed that the σ^E-^dependent genes that are upregulated in the P_*sigF*_ strain are the first and second groups, *i*.*e*., those that rely exclusively on σ^E^ for expression and those that are activated by SpoIIID (Fig. 3A; S3 Table). Conversely, essentially no upregulated genes are seen among the σ^E^-dependent genes that are repressed by GerR or SpoIIID (Fig. 3A; S3 Table). These genes correspond to pulses X_2_ and X_3_ in Fig. 1B [9]. It is likely that transcription of these genes has already been turned off by the time CsfB starts to accumulate in the mother cell, explaining the lack of effect of the mutation. In contrast, the increased expression of the σ^E-^ and σ^E^/SpoIIID-dependent genes suggests that in the absence of CsfB, the activity of σ^E^ in the mother cell is increased or prolonged.

Several of the σ^E^/SpoIIID-dependent genes are involved in production and activation of σ^K^[9,29]. The σ^K^ regulon itself can be sub-divided into genes whose expression is activated by σ^K^ alone, those that require GerE for full expression (pulse X_7_ in Fig. 1B) and those repressed by GerE (pulse X_6_) [9]. Transcriptional profiling of the P_*sigF*_ strain shows that a significant fraction of the σ^K^-dependent genes is upregulated, regardless of GerE dependency (Fig. 3A; S3 Table). We presume that the increased expression of the σ^K^ regulon may result from augmented activity of σ^K^ (but see also below, section on the ectopic activation of the σ^G^ regulon).

In all, the genome-wide transcriptional profiling data highlight the importance of *csfB* for modulating the levels of σ^E^- and σ^K^-dependent gene expression in the mother cell, and are consistent with the interpretation that perturbations affecting the mother cell line of gene expression in a *csfB* mutant are mainly caused by an increased and protracted activity of σ^E^.

### CsfB contributes to the inhibition of σ^G^ and the timely activation of σ^K^ in the mother cell

A somewhat unexpected result of the global transcriptional profiling analysis was that the mutation in the σ^K^-dependent promoter of *csfB* also caused a generalized increase in the expression of the σ^G^ regulon (Fig. 3A; S3 Table), including genes that are repressed (pulse X_4_ in Fig. 1B) or activated by SpoVT (pulse X_5_). We considered two possibilities. First that the increase in σ^E^ activity in the P_*sigF*_ strain somehow led to an increase in the activity of σ^G^ in the forespore. Second, that eliminating expression of *csfB* in the mother cell alleviated some of the restrictions imposed on σ^G^ to become active in this cell type. To test these hypotheses, we first examined the localization of the fluorescence signal from a P_*sspE*_-CFP fusion in cells of the P_*sigF*_-*csfB* strain, in comparison with the wild type, at hour 3 of sporulation in resuspension medium. In the wild type strain, and as expected for a σ^G^-controlled gene, the fluorescence signal was confined to engulfed forespores (S4A Fig, yellow arrows). Fluorescence from P_*sspE*_-*cfp* was also detected in engulfed forespores for the P_*sigF*_ strain (S4A Fig, yellow arrows). However, for the P_*sigF*_ mutant, a week fluorescence signal was also detected in the mother cell prior to engulfment completion (S4A Fig, white arrows). The quantitative analysis of the signal shows that the mother cell-associated fluorescence in P_*sigF*_ sporangia that have not completed the engulfment sequence is consistently higher than for the wild type (S4B Fig; see also S1 Text).

In an attempt to verify this observation, we turned to fluorescence *in situ* hybridization (FISH) using a specific antisense DNA probe labelled with Cy3, in an attempt to localize the *sspE* mRNA [30]. Because at this time in sporulation the forespore is not yet recognizable by phase contrast microscopy, we relied mainly on the size of the fluorescence signal to distinguish mother cell or sporangia (larger) from forespore (smaller) localization. The FISH images suggest that the signal is confined to the forespore in the wild type strain, whereas the signal is mostly associated with whole cells (sporangia) in the P_*sigF*_ strain (Fig. 3B). In cells of the mutant strain, the size of the fluorescence signal is 1.5 to 2 times longer than that observed for the wild type strain (Fig. 3B). Moreover, the size of the fluorescence signal for the P_*sigF*_ strain coincides with the size of the sporangia as judged from the phase contrast images (Fig. 3B). This suggests whole cell production of the *sspE* mRNA. Some cells of the P_*sigF*_ mutant do not show a forespore-associated signal (Fig. 3B, insert). This suggests that expression of *sspE* may initiate earlier in the mother cell than in the forespore, in line with the detection of P_*sspE*_-CFP in the mother cell, for the P_*sigF*_ strain, prior to engulfment completion (S4 Fig; see also above). In any event, the results are consistent with the hypothesis that the *sspE* mRNA is also produced in the mother cell. Therefore, these data suggest that eliminating transcription of *csfB* from the P_*sigK*_ promoter increases transcription of σ^G^-dependent genes in the mother cell and thus that CsfB contributes to the negative regulation of σ^G^ in this cell. Presumably, CsfB is part of a composite negative feedback loop limiting σ^G^ activity in the mother cell similar to the mechanism that prevents activation of σ^G^ in pre-divisional cells. In these cells, CsfB plays a key role in inhibiting σ^G^ activity, along with the SpoIIAB anti-sigma factor and the LonA protease [20,31,32]. Previous work has shown that when the *sigG* gene is placed under the control of a σ^E^-controlled promoter, SpoIIAB and LonA are also important for the inhibition of σ^G^ activity in the mother cell [15,20,32]. In an extension of this work, we now show that SpoIIAB plays the leading role in σ^G^ inhibition in the mother cell, while LonA and CsfB appear to have largely redundant supportive contributions (S5A Fig). Nevertheless, these results demonstrate the ability of CsfB to act as an inhibitor of σ^G^ activity in the mother cell, in line with the observed σ^G^ activity in the P_*sigF*_ strain (Fig. 3 and S4 Fig).

Importantly, during the course of these experiments aiming at directly testing for a role of CsfB in the inhibition of σ^G^ in the mother cell (above), we noticed that the forced production of σ^G^ in the mother cell resulted in premature activity of σ^K^ (S5B Fig). Activation of σ^K^ requires pro-σ^K^ processing, which in turn depends on the activity of σ^G^ in the forespore, following engulfment completion [25,33]. Since σ^G^ shows some activity prior to engulfment completion in the P_*sigF*_ strain (above), we therefore considered the possibility that the augmented transcription of the σ^K^-controlled genes, as detected in our microarray analysis (above), could result in part from premature processing of pro-σ^K^ independently of the σ^G^-dependent pathway that operates following engulfment completion (S5C Fig). To test this idea, we surveyed the accumulation of pro-σ^K^ and σ^K^ by immunoblotting, throughout sporulation. Pro-σ^K^ was first detected in both the wild type strain and in the P_*sigF*_ strain 140 min after resuspension in sporulation medium (Fig. 3C). However, mature σ^K^ is first detected 180 min after resuspension for the P_*sigF*_ strain, and only at minute 200 for the wild type (Fig. 3C). Moreover, even at minute 200, the fraction of mature σ^K^ is higher for the P_*sigF*_ strain (Fig. 3C). Therefore, the lack of *csfB* expression in the mother cell, can lead to premature activation of pro-σ^K^, again underscoring the need for strict inhibition of σ^G^ activity in the mother cell.

### CsfB forms a complex with σ^E^ but not with σ^K^ in sporulating cells

**Fig 4:**
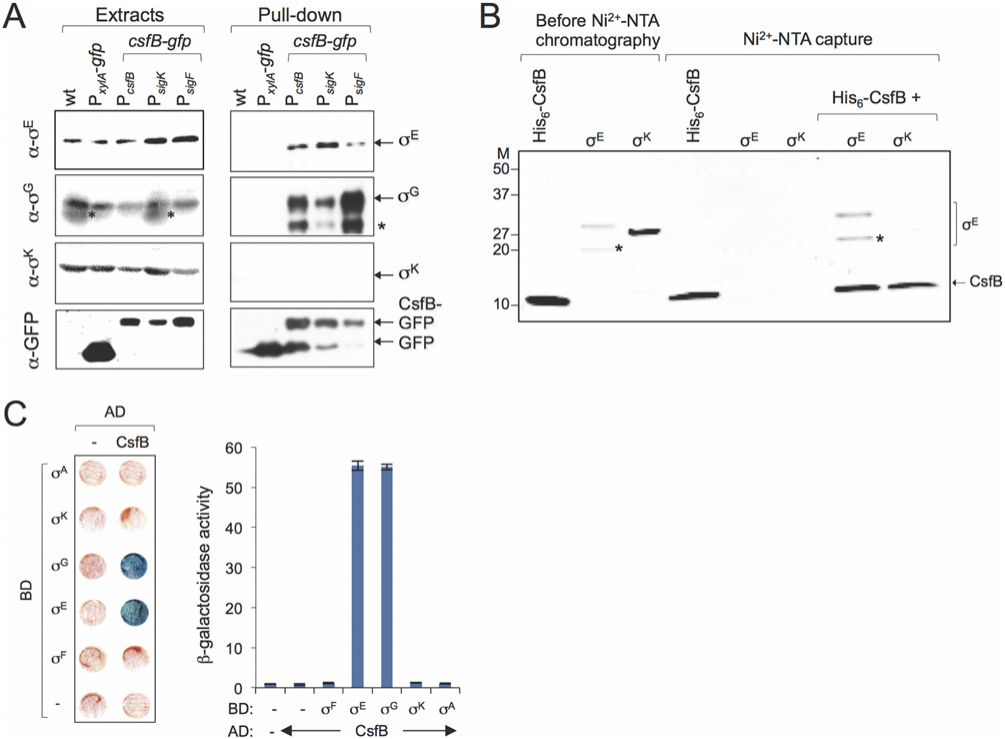
Sigma-CsfB interactions. **A**: pull-down assays using GBP. Whole cell extracts were prepared for cultures of different strains 4 hours after the onset of sporulation in resuspension medium. The extracts were cleared and incubated with GBP bound to sepharose beads. Samples of the whole cell extracts, as well as the proteins bound to the GBP beads were visualized, following elution, by immunoblotting with anti-σ^G^, anti-σ^E^, anti-σ^K^ and anti-GFP antibodies. Strains in **A**: a wild type strain, a strain producing GFP from the xylose-inducible P_*xylA*_ promoter; strains producing CsfB-GFP from its native promoter region (P_*csfB*_), from the σ^F^-type promoter (P_*sigF*_) or the σ^K^-type promoter (P_*sigK*_), as indicated. The asterisk indicates a likely degradation product of σ^G^. **B**: Ni^2+^-NTA affinity chromatography assay for CsfB-σ interactions. His_6_-CsfB, and untagged σ^E^ and σ^K^, were purified from *E*. *coli* cells and analysed by SDS-PAGE (first three lanes). The three proteins were then individually applied to a column and eluted with an imidazole buffer. The eluted proteins were detected following SDS-PAG by Coomassie-staining. His_6_-CsfB bound to the Ni^2+^-NTA column whereas σ^E^ or σ^K^ did not. σ^E^ but not σ^K^, bound to the column in the presence of His_6_-CsfB. The asterisk indicates a likely degradation product of σ^E^. Molecular weight markers (M, in kDa) are shown on the left side of panels B. The asterisks in A and B indicate likely degradation products of σ^G^ or σ^E^. **C**: colony lift assay (left) and assays in liquid medium (right) for the detection of β-galactosidase activity in yeast strains expressing fusions of CsfB to the GAL4 activation domain (AD) and fusions of σ^F^, σ^E^, σ^G^, σ^K^, and σ^A^ to the GAL4 binding domain (BD), as indicated. Assays in which the BD and AD were expressed from empty vectors were used as negative controls (“-“).

The analysis of the global transcriptional profiling data raised the possibility that CsfB could act as an inhibitor of σ^E^and/or σ^K^. This prompted us to determine whether the anti-sigma factor could form complexes with either sigma factor. To isolate CsfB-GFP and interacting proteins from sporulating cultures of a strain expressing *csfB-gfp*, we used a GFP-binding protein (GBP) coupled to a chromatographic matrix (GFP-Trap beads). As controls, we examined a wild type strain carrying no *gfp* fusion and a strain producing GFP under the control of the xylose-inducible P_*xylA*_ promoter. The extracts were prepared 4 hours after the onset of sporulation, when CsfB-GFP is known to accumulate in the mother cell (Fig. 2B; above). Control experiments also confirmed the accumulation of σ^E^, σ^G^, σ^K^ or GFP in the whole-cell extracts prepared from the various strains (Fig. 4A). The extracts from all strains were incubated with GFP-Trap beads, the bound proteins eluted and identified by immunoblot analysis with antibodies raised against σ^E^, σ^G^, σ^K^ or GFP. We found σ^E^ but not σ^K^, to be pulled down efficiently from extracts of the strain producing CsfB-GFP. By contrast, σ^E^ was not recovered from extracts of the strain containing no GFP fusion or from the strain that produced unfused GFP (Fig. 4A). Thus, retention of σ^E^ by the GFP-trap beads depended on formation of a complex with CsfB-GFP. As expected, CsfB-GFP was able to pull down σ^G^ [20], a property used here as a positive control for the experiment (Fig. 4A). Interestingly, a role for CsfB in inhibiting σ^E^ activity in the forespore soon after asymmetric division was previously suggested [34]. Under the conditions of our experiments, however, σ^E^ is only expected to accumulate in the mother cell and the expression of *csfB* has switched to the mother cell in most sporangia in the population when the cells were harvested for the pull down assays (see above). Therefore, the result reported in Fig. 4A most likely reflects an interaction occurring between σ^E^ and CsfB in the mother cell and not in the forespore.

In agreement with this interpretation, CsfB-GFP produced solely from the P_*sigK*_ promoter, was still able to pull down σ^E^ (Fig. 4A). In addition, this construct could also pull down σ^G^ and conversely CsfB-GFP produced from the P_*sigF*_ was able to pull down σ^E^ (Fig. 4A). Even though these observations could be explained by complex formation after the cells are disrupted in particular for the P_*sigF*_ strain (provided that there was enough free CsfB-GFP), we note that more σ^E^ and less σ^G^ seemed to be pulled-down in the strain where *csfB-gfp* expression is restricted to the mother cell than in the strain producing CsfB-GFP in both compartments (Fig. 4A). Thus, it is possible that the amounts of σ^E^ and σ^G^ pulled-down from the P_*sigK*_-*csfB*-*gfp* carrying strains reflect the levels of both of these proteins in the mother cell. Considering that σ^G^ can accumulate and become active in the mother cell under certain conditions [15,20]; S4 Fig and S5 Fig; see also above), some σ^G^ might be present in the mother cells at hour 4 of sporulation.

### CsfB binds to σ^E^ but not to σ^K^

One of the consequences of inactivating the P_*sigK*_ promoter of *csfB* was the protracted expression of a specific class of σ^E^-dependent genes. A prediction that stems from these results and from the pull-down experiments described above is that CsfB binds directly to σ^E^. To test this hypothesis, we purified His_6_-CsfB, σ^E^ and σ^K^ (both lacking their pro-sequences) overproduced in *E*. *coli* (Fig. 4B, first three lanes and S2A Fig) (Note that in addition to σ^E^, which has a molecular weigth of 29 kDa, a stable proteolytic fragment of about 20 kDa accumulated in the preparations; Fig. 4B). We then asked whether His_6_-CsfB immobilized onto a Ni^2+^-NTA column could capture untagged, purified σ^E^ or σ^K^. While His_6_-CsfB bound efficiently to the Ni^2+^-NTA column, neither σ^E^ nor σ^K^ did (Fig. 4B). However, in the presence of His_6_-CsfB, σ^E^ (but not σ^K^), was efficiently retained by the column (Fig. 4B), a result fully consistent with the pull-down experiments described above (note that both σ^E^ and the 20 kDa fragment were retained; Fig. 4B, left lanes).

To confirm these results with a different technique, we also carried out a GAL4-based yeast two-hybrid assay, an approach that had been used before to dissect the interaction between σ^G^ and CsfB [20]. We constructed translational fusions of σ^F^ (as a negative control), σ^G^ (as a positive control), σ^E^, σ^K^, σ^A^ or CsfB to the C-terminus of the GAL4 DNA binding (BD) or activation domains (AD). The various fusion proteins were expressed in different combinations in yeast cells and checked for their ability to interact *in vivo*, as assessed by the expression of a *lacZ* gene preceded by a GAL4-responsive element. As expected, we detected an interaction of CsfB with σ^G^, but not with σ^F^([20]; Fig. 4C). In addition, CsfB interacted with σ^E^ (Fig. 4C), suggesting that the two proteins could indeed establish specific contacts. No interaction was detected between CsfB and σ^K^ or σ^A^ (Fig. 4C).

Thus, both the yeast two-hybrid and the affinity chromatography assays indicate that CsfB and σ^E^ directly interact.

### CsfB inhibits transcription *in vitro* from σ^G^- and σ^E^-dependent promoters

**Fig 5:**
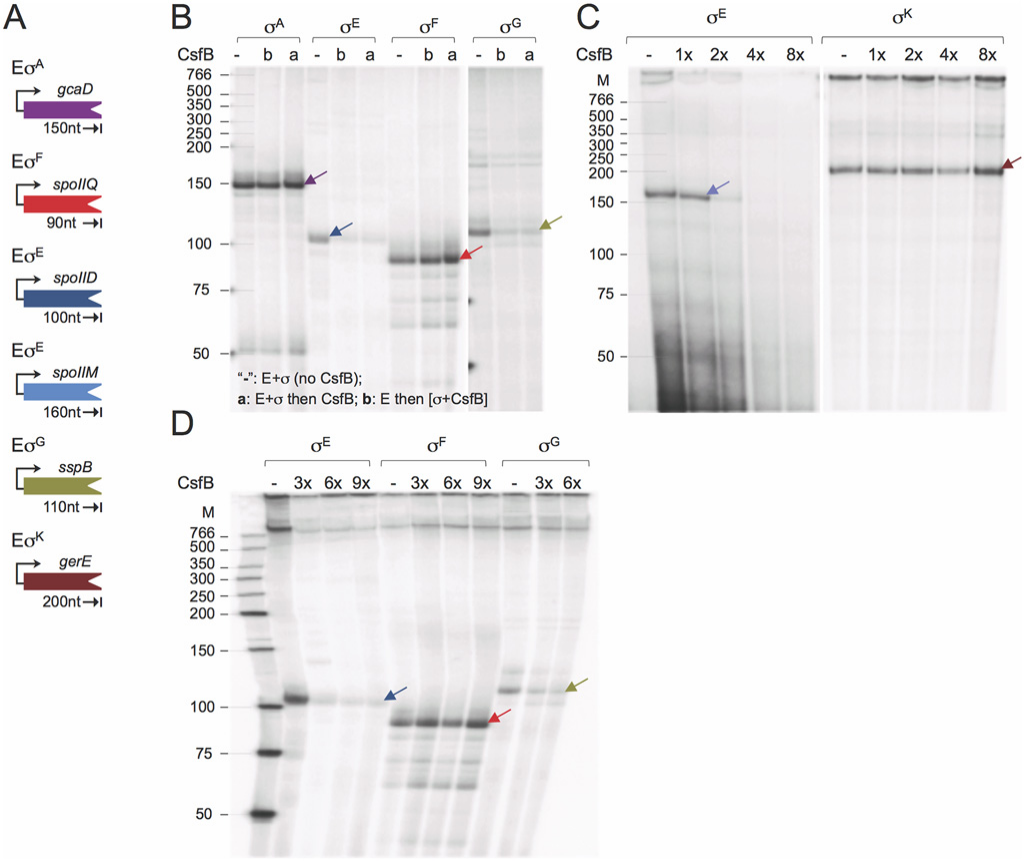
CsfB inhibits *in vitro* transcription by RNA polymerase associated with σ^G^ or σ^E^. **A**: schematic representation of the promoter-containing PCR fragments used as templates for the *in vitro* transcription reactions. The expected size (in nucleotides) for each of the run-off products is indicated. **B**: effect of CsfB on *in vitro* transcription reactions with the indicated RNA polymerase holoenzymes. CsfB (130 nM) was either added to the reaction after mixing core (E; 13 nM) and the sigma subunit (“a”; 130 nM) or together with the sigma subunit to a mixture already containing core (“b”). The symbol “-”refers to a control reaction lacking CsfB. **C** and **D**: effect of the CsfB concentration, shown in molar ratio relative to RNA polymerase (13 nM), on *in vitro* transcription reactions with the indicated holoenzymes. CsfB was added at the same molar concentration as sigma (130 nM, 1x) or 2, 3, 4 and 8-fold excess and the mixture added to core RNA polymerase (13 nM). In B-D, arrows indicate the position of the expected run-off products, identified with arrows, which maintain the color code for the templates as represented in A. The position of molecular weight markers (in nucleotides) is shown on the left side of the panels.

While CsfB binds to both σ^G^ [20] and σ^E^ ([34]; see above), no study has shown direct inhibition of transcriptional activity by the anti-sigma factor. To test the ability of purified CsfB to inhibit σ^G^- or σ^E^-directed transcription, core RNA polymerase (E) was purified from *B*. *subtilis*, and reconstituted with σ^F^, σ^E^, σ^G^, σ^K^, or σ^A^ overproduced and purified from *E*. *coli* cells (S2A Fig). As templates for *in vitro* transcription reactions, we used PCR-generated DNA fragments corresponding to promoters of genes known to be under the control of the sigma factors tested, and whose transcriptional start site has been mapped. As such, the *gcaD* gene was used as the template for σ^A^-directed transcription [35,36], *spoIIQ* as a template for Eσ^F^ [27,37], *spoIID*, *spoIIM*, *spoIIIA p1* and *spoIIIA p2* as templates for Eσ^E^ [38,39,40], *sspB* for Eσ^G^ [30], and *gerE* for Eσ^K^ [33] (Fig. 5A and S2B Fig). All forms of RNA polymerase tested directed the production of run-off transcripts of expected sizes (Fig. 5B-D and S2C Fig). No specific transcription products were seen when templates were mixed with core RNA polymerase in the absence of a σ subunit (S2C Fig). CsfB did not inhibit transcription by Eσ^A^, Eσ^F^ (Fig. 5B) or Eσ^K^ (Fig. 5C), but inhibited the σ^E^-directed utilization of the *spoIIM* (Fig. 5B), *spoIID* (Fig. 5C and D), *spoIIIA p1* and *spoIIIA p2* promoters (S2C Fig). CsfB also inhibited the σ^G^-directed transcription of *sspB* (Fig. 5B and D). Inhibition of Eσ^A^, Eσ^F^or Eσ^K^ by CsfB was not observed even at molar ratios higher than those that inhibited Eσ^E^ (Fig. 5C and D). Interestingly, inhibition of Eσ^E^, and to some extent of Eσ^G^, required molar ratios higher than 1 (Fig. 5B and D; S2C Fig). One possibility is that active CsfB is a dimer (or a higher order multimeric form), in agreement with the results of a previous study [21].

In total, the run-off transcription assays show that CsfB inhibits transcription by the RNA polymerases that contain the σ subunit to which it can bind *in vitro*, *i*.*e*., σ^E^ or σ^G^.

### Mapping the interaction between CsfB and σ^E^

**Fig 6:**
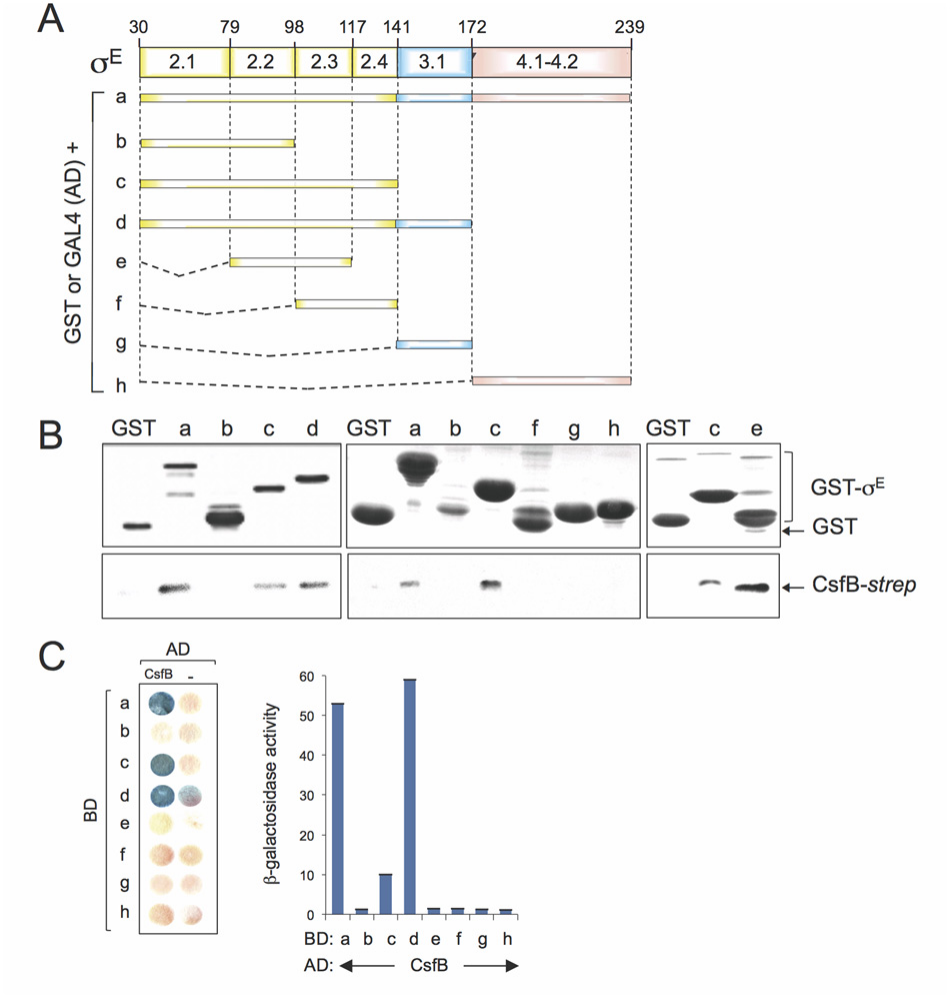
Mapping the region in σ^E^ involved in the interaction with CsfB. **A**: schematic representation of the *B*. *subtilis* σ^E^ protein, with the various regions within domains 2 and 4, represented. The full-length protein (*a*), or the indicated fragments (*b* to *h*) were fused to either the binding domain of GAL4 or to GST (below). The numbers refer to the residue number in the primary structure of σ^E^. **B**: GST pull-down assays with purified CsfB-*Strep* II tag. GST and the various GST-σ^E^ fusions (as depicted in A) were bound to glutathione beads and incubated with purified CsfB-*Strep* II (100 nM). Bound proteins were detected, following elution, with an anti-*Strep* II tag antibody. The amounts of GST or the various GST fusion proteins bound to the glutathione beads were estimated by immunoblot with an anti-GST antibody. **C**: colony lift assay (left) and quantitative assays in liquid cultures (right) for the detection of β-galactosidase activity in yeast strains expressing fusions of CsfB to the GAL4 activation domain (AD) and fusions of different σ^E^ segments (as depicted in C) to the GAL4 binding domain (BD), as indicated. Assays in which the BD and AD were expressed from empty vectors were used as negative controls (“-“).

To map the region(s) involved in the CsfB-σ^E^ interaction, we performed GST-pull down and yeast two-hybrid assays, two techniques that were used before to map the interaction between CsfB and region 2.1 of σ^G^ [20]. For that purpose, full-length mature σ^E^ or different fragments of the sigma factor were fused to either GST or to GAL4 (Fig. 6A). For the GST pull-down assays, we used a CsfB-*StrepII*-tagged protein [20] and a GST-σ^E^ fusion protein, lacking its pro region (sigma fragment *a*, Fig. 6A) or GST fusions to different fragments of σ^E^ (fragments *b* to *h*; Fig. 6A), overproduced and partially purified from *E*. *coli*. We incubated CsfB-*StrepII*-tag with GST or the GST-σ^E^ fusion proteins immobilized on glutathione beads. After washing, the presence of CsfB in the elution samples was assessed by immunoblotting with an anti-*Strep*II tag antibody. The CsfB protein was retained by GST-σ^E^ (fragment *a*) but not by GST alone (Fig. 6B, left panel). GST fusions to σ^E^ fragments corresponding to regions 2.1 through 2.4 (fragment *c*, GST-σ^E 30–141^), 2.1 through 3.1 (fragment *d*, GST-σ^E 30–172^), and 2.2/2.3 (fragment *e*, GST-σ^E 79–117^) pulled down CsfB, whereas fragments encompassing regions 2.1/2.2 (sigma fragment *b*; GST-σ^E 30–98^), 2.3/2.4 (fragment *f*, GST-σ^E 98–141^), 3.1 (fragment *g*, GST-σ^E 141–172^), and 4.1/4.2 (fragment *h*, GST-σ^E 172–239^), did not (Fig. 6B). With the exception of fragment *e* (2.2/2.3, σ^E 79–117^), the same fragments also showed an interaction with CsfB in yeast two-hybrid assays (Fig. 6C). The lack of interaction of fragment *e* in the yeast two-hybrid assay may be due to the topology and/or stability of the GAL4 fusion.

Region 2.1 was previously implicated in the interaction of CsfB with σ^G^ [20]. However, this region does not appear to be sufficient to mediate the interaction of CsfB with σ^E^. In contrast, because σ^E 79–117^ (regions 2.2/2.3) interacted with CsfB, but neither σ^E 30–98^ (regions 2.1/2.2) nor σ^E 98–141^ (regions 2.3/2.4) did, it is likely that residues at the end of region 2.2 and/or at the beginning of region 2.3 of σ^E^ are necessary for the interaction. In any event, our results show that CsfB and σ^E^ interact directly, and that the region required for the interaction differs from that found in σ^G^ [20,22].

### A single residue in region 2.3 allows CsfB to discriminate between σ^E^ and σ^K^

**Fig 7:**
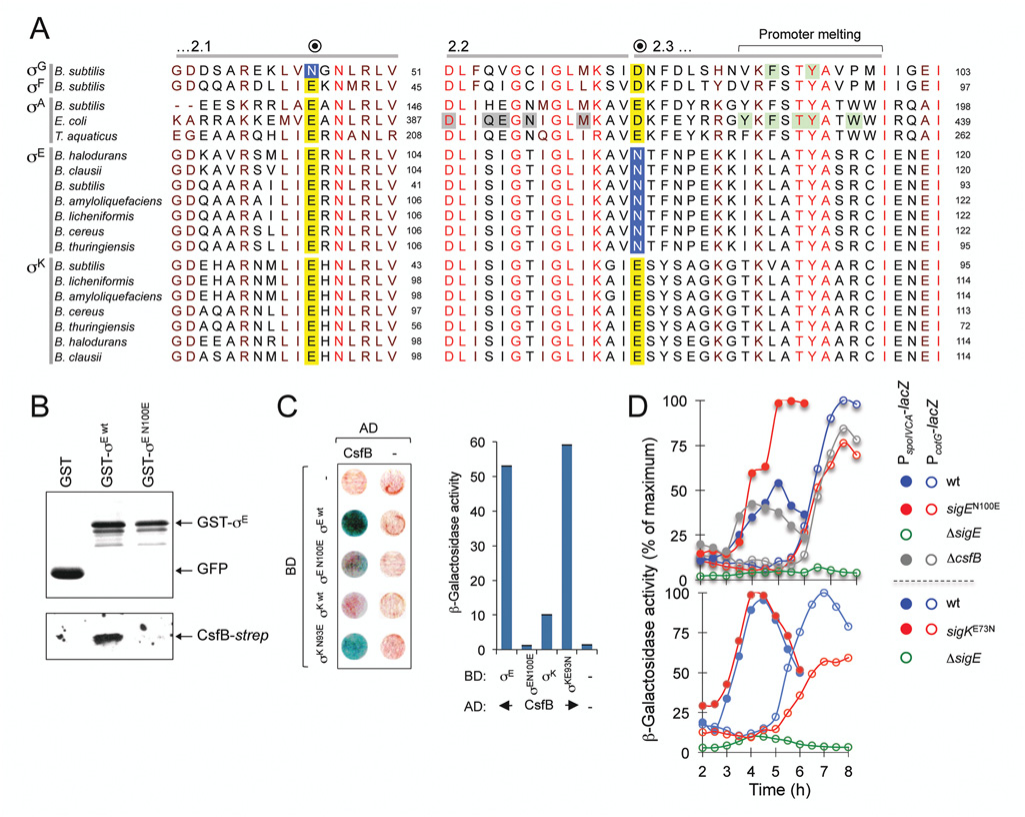
A residue in σ^E^ involved in the interaction with CsfB. **A**: alignment of regions 2.1, 2.2 and the beginning of region 2.3 from the σ^A^, σ^F^, σ^G^, σ^E^, and σ^K^ proteins of the indicated species (the sequence of σ^A^ from *T*. *thermophilus* is identical to that of *T*. *aquaticus* for the segments represented). The residue in *B*. *subtilis* σ^G^ important for the interaction with CsfB is shown in a blue box. Note that the homologous position in σ^F^ and in the other sigma proteins is invariantly occupied by a glutamic acid (yellow box). The residue herein identified in region 2.3 of *B*. *subtilis* σ^E^ (N100) that is important for binding by CsfB is shown in a blue box. This residue is conserved among *Bacillus* orthologues of σ^E^ whereas in other sigma factors the homologous position is occupied by and acidic residue (yellow box). Residues in region 2.2. of *E*. *coli* σ^A^ implicated in core binding are highlighted in grey; residues, at the end of region 2.3, involved in promoter meting are also indicated (reviewed by [55]). The residues boxed in green were shown to affect sporulation in *B*. *subtilis* [20] and promoter melting in *E*. *coli* [68,69]. The accession numbers of the aligned sequences are given in the Material and Methods section. **B**: GST pull-down assays to investigate the role of E100 of σ^E^ in the interaction with CsfB. GST, GST-σ^E^ and GST-σ^E N100E^ fusions were bound to glutathione beads and incubated with purified CsfB-*Strep* II (100 nM). Protein complexes were captured on glutathione sepharose beads, and visualized, following elution, by immunoblotting with anti-GST, or anti-*Strep* tag antibodies. **C**: colony lift assays (left) and assays in liquid medium (right) for the detection of β-galactosidase activity in yeast strains expressing fusions of CsfB to the GAL4 activation domain (AD) and fusions of σ^E wt^, or σ^E N100E^, σ^K wt^, or σ^K E93N^, to the GAL4 binding domain (BD), as indicated. Assays in which the BD and AD were produced from empty vectors were used as negative controls (“-“). **D**: effect of the *sigEN100E* and *sigKE73N* alleles on σ^E^- and σ^K^-dependent gene expression. The figure shows the expression of P_*spoIVCB*_-*lacZ* (σ^E^-dependent) and P_*cotG*_-*lacZ* (σ^K^-dependent) transcriptional fusions during sporulation. Samples were withdrawn from cultures of the represented strains, at the indicated times, in hours after the onset of sporulation in re-suspension (denoted as T0), and assayed for β-galactosidase activity (shown in Miller units).

The N45E substitution in σ^G^ results in loss of CsfB binding, whereas the E39N substitution in σ^F^ is sufficient for conferring CsfB binding ability [20]. However, the residue homologous to N45 in σ^E^ corresponds to a glutamate, E64 (Fig. 7A). Together with the mapping experiments described in the preceding section, this observation suggests that the interaction of CsfB with σ^E^ differs from that with σ^G^. To more precisely delineate the determinants for CsfB binding to σ^E^, we sought to identify residues that are necessary for the interaction. An inspection of the amino acid sequence of σ^E^ in regions 2.2 and 2.3 revealed an asparagine residue located at position 100 (*i*.*e*., at the beginning of region 2.3) and conserved among all of *Bacillus* σ^E^ orthologues. Strikingly, the position homologous to N100 in σ^K^ is occupied by an aspartate (E93) in *B*. *subtilis* and invariably (with the exception of the σ^E^ proteins) by an acidic residue in all other *Bacillus* sigma factors (Fig. 7A). N100 in σ^E^ is located in the vicinity of several residues involved in promoter melting (Fig. 7A). By contrast, E64 in σ^E^ and N45 in σ^G^ are located at the beginning of a helix within which several contacts are established with the β´ subunit of RNA polymerase (S6 Fig). Thus, depending on which sigma factor is present in the complex, CsfB appears to bind to two distinct functional surfaces of the RNA polymerase holoenzyme.

We wanted to test whether the CsfB anti-sigma factor discriminated between σ^E^ and σ^K^ by differentiating an asparagine from an acidic residue at the beginning of region 2.3. We constructed GST and GAL4 fusions to forms of σ^E^ bearing the single amino acid substitution N100E and used them in pull-down and yeast two-hybrid experiments. We found that GST-σ^E N100E^ did not pull-down CsfB-*strepII* partially purified from *E*. *coli* cells (Fig. 7B) and showed a much-reduced interaction (when compared to the wild type sigma factor) with the anti-sigma factor in a yeast two-hybrid assay (Fig. 7C). By contrast, a GAL4 fusion to σ^K^ bearing an E93N substitution interacted strongly with the anti-sigma factor in the yeast two-hybrid assay (Fig. 7C). To determine whether the N100E and E93N substitutions in σ^E^ or σ^K^ affected their activity *in vivo*, the corresponding mutations were first transferred to the *sigE* and *spoIVCB* genes (*spoIVCB* codes for the N-terminal half of σ^K^; see the Material and Methods section for details). We then used P_*spoIVCA*_- (σ^E^-dependent promoter) and P_*cotG*_-*lacZ* (σ^K^-dependent promoter) transcriptional fusions to monitor the activity of σ^E^ and σ^K^ respectively, during sporulation. Consistent with loss of CsfB regulation, the N100E substitution increased expression of *spoIVCA-lacZ*, whereas the E93N replacement reduced expression of *cotG-lacZ* (Fig. 7D). Expression of P_*spoIVCA*_-*lacZ* or P_*cotG*_-*lacZ* in a Δ*csfB* mutant did not differ much from the wild type (Fig. 7D), presumably because premature activation of pro-σ^K^ somehow compensates for increased activity of σ^E^ (see also above). In any event, the N100E substitution is sufficient to render σ^E^ refractory to CsfB, whereas the E93N substitution suffices to make σ^K^ susceptible to CsfB.

### The N100E substitution in σ^E^ affects the assembly of the spore surface layers

Assembly of a protective protein shell called the coat that forms the spore surface, involves the timely production of over 80 components under the control of σ^E^ or σ^K^ [41,42]. Since the N100E substitution increases the activity of σ^E^ and reduces that of σ^K^ we wanted to examine the coat protein composition in spores of the strain producing σ^E N100E^. Proteins were extracted from purified spores by treatment with a buffer containing SDS and the reducing agent DTT, or by NaOH [43]. The first treatment releases about 80 proteins from wild type spores (S7A Fig, top) whereas NaoH extraction produces a much smaller collection of extractable proteins (S7A Fig, bottom). We found that several proteins were more extractbale from N100E spores than from wild type spores, among which the species labelled *a-e* (SDS/DTT extraction) and *f-k* (NaOH extraction) in S7A Fig (bottom panel). For example, the outer coat proteins CotA (in band *a*), CotB (in *b*), and Tgl (in *d*), as well as the inner coat proteins CotN (in bands *h* and *i*), YaaH (in *c*) and YybI (in band *d*) were more extractable from σ^E N100E^ spores (S7A Fig, see also S1 Text). Synthesis of YaaH, CotN and YybI is under σ^E^ control, whereas production of CotA, CotB, and Tgl is mainly controlled by σ^K^ [9,29,41,44,45,46]. Thus, the N100E substitution has a strong and global impact on the assembly of the spore surface layers, affecting the assembly of proteins from both the inner and outer coat layers, which are produced at different periods (controlled by σ^E^ or by σ^K^) during spore coat assembly.

## Discussion

A key finding of our study is that expression of *csfB* switches from the forespore to the mother cell midway into the process of sporulation. The regulatory region of *csfB* includes two promoters: a σ^F^-type promoter (P_*sigF*_) utilized by σ^G^ in pre-divisonal cells and by σ^F^ in the forespore [14,17,20], and a σ^K^-type promoter (P_*sigK*_), located further upstream, active in the mother cell following engulfment completion. Activation of *csfB* transcription in the mother cell coincides with a decline in forespore-specific expression (Fig. 2). It has been hypothesized that a reduction in the levels or activity of CsfB in the forespore could be a factor promoting σ^G^ activity following engulfment completion [14,17,20]. SpoIIAB also disappears from the forespore following engulfment completion [47]. It remains unclear how CsfB activity is turned off in the forespore during the late stages of sporulation, since σ^G^ can utilize P_*sigF*_ *in vitro* and *in vivo* ([20]; this work). However, it has been suggested that Zn^2+^-depletion in the forespore, following engulfment completion, could render CsfB unstable [21]. If so, then the decrease in the CsfB-GFP signal in the engulfed forespore (Fig. 1B) could represent protein degradation.

### Enforcing the transition from early to late stages of development

**Fig 8:**
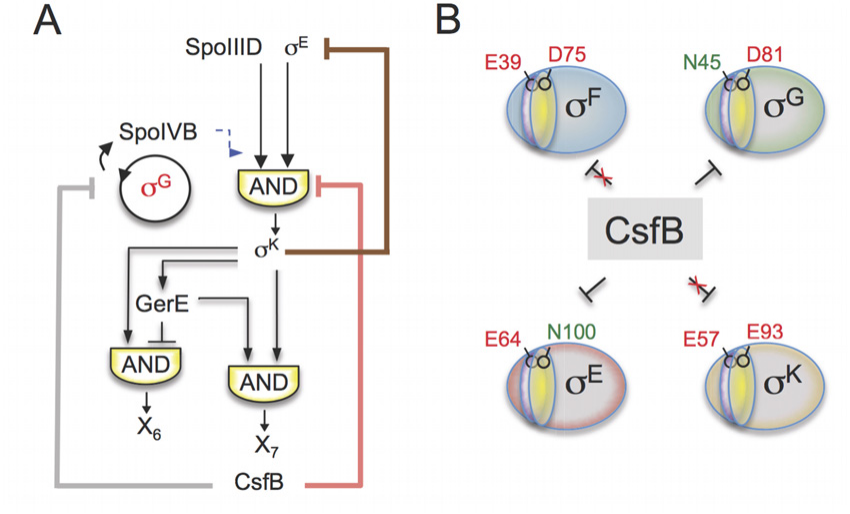
Model for the functions of CsfB. **A**: following engulfment completion, two negative feedback loops are established that act to limit the level (brown line) and the activity of σ^E^ (red line). The loop that acts at the level of σ^E^ activity involves the σ^K^-dependent production of CsfB (this work), and restricts mainly the expression of the σ^E^-and SpoIIID-dependent gene class. Both feedback loops promote proper switching from the early (pre-engulfment, σ^E^-dependent) to late (post-engulfment completion, σ^K^-dependent) stages in development. Together with LonA and SpoIIAB, CsfB also antagonizes σ^G^ in the mother cell (grey line). Minimizing the chances of σ^G^ becoming active in the mother cell prevents the premature, forespore-independent activation of σ^K^. Thus, the action of CsfB superimposes both onto the transcriptional and cell-cell signaling networks. **B**: the panel represents the residues that allow binding of CsfB to σ^G^ (N45) and σ^E^ (N100), and that are sufficient to render σ^F^ (E39) and σ^K^ (E93) resistant to the anti-sigma factor. N45 in σ^G^ and E39 in σ^F^ are located in conserved region 2.1 (purple sector) whereas N100 in σ^E^ and E93 in σ^E^ are located in region 2.3 (yellow).

Genome-wide transcriptional profiling analysis showed that inactivation of P_*sigK*_, which abolishes CsfB accumulation in the mother cell, caused increased transcription of σ^E^-controlled genes that either rely solely on σ^E^ for expression or are dependent on the type I coherent FFL formed by σ^E^ and the ancillary transcriptional activator SpoIIID [9]. Increased σ^E^ activity was also observed using a σ^E-^responsive *lacZ* reporter in a strain expressing the CsfB-resistant form of σ^E^ (σ^E N100E^). By contrast, σ^E^-dependent genes repressed by GerR or SpoIIID (*i*.*e*., the output of the type I incoherent FFLs generating pulses X_2_ and X_3_ in Fig. 1B) did not show increased expression in the P_*sigF*_-*csfB* strain, most likely because their transcription has already been switched off at the time of analysis due to the action of the two repressors (Fig. 3A). In general, these observations are consistent with an increase in the activity of σ^E^ in the absence of CsfB and with the properties of the FFLs formed by σ^E^ [3]. They also support a model in which the appearance of CsfB in the mother cell promotes the transition from early to late cell-type specific gene expression (*i*.*e*., the σ^E^ to σ^K^ switch) (Fig. 8A).

While a previously described negative feedback loop driven by σ^K^ reduces the levels of σ^E^ [11,12,48], the feedback loop revealed in this work, and similarly initiated by σ^K^, uses CsfB to limit the activity of σ^E^ in the mother cell. Thus two partially redundant feedback loops evidently function to promote proper switching from σ^E^- to σ^K^-dependent transcription following engulfment completion, and thus the developmental transition from early to late stages of morphogenesis (Fig. 8A).

### σ^G^ and the activity of σ^K^

Interference with *csfB* expression in the mother cell, as when P_*sigK*_ is inactivated, also leads to increased expression of several genes under σ^K^ control (Fig. 3). Because CsfB does not bind to σ^K^ and does not inhibit σ^K^-directed transcription *in vitro*, it is unlikely that the increased activity of σ^K^ is due to the loss of a direct interaction with CsfB. Instead, it is more likely to be a consequence of the ectopic activation of σ^G^ in the mother cell. Previous work has shown that two negative regulators of σ^G^, the anti-sigma factor SpoIIAB and the LonA protease, effectively counteract σ^G^ in the mother cell, when its synthesis is artificially induced by driving expression of *sigG* from a σ^E^-dependent promoter [15]. Under the same genetic conditions, we showed that CsfB contributes, along with LonA and SpoIIAB, to the inhibition of σ^G^ activity in the mother cell (S5 Fig). In the P_*sigF*_-*csfB* mutant, our transcriptional profiling data indicate that most of the genes in the σ^G^ regulon show increased expression. This increased expression is due in part to increased activity of σ^G^ in the mother cell of P_*sigF*_ sporangia. Not only transcription from the σ^G^-dependent P_*sspE*_ promoter fused to *cfp* is detected in the mother cell prior to engulfment completion (S4 Fig), but FISH experiments also show accumulation of the *sspE* transcript in the mother cell (Fig. 3). Therefore, CsfB is one of several redundant mechanisms that act to silence σ^G^ in the mother cell.

In wild type cells, it is the activity of σ^G^ in the forespore following engulfment completion that triggers σ^K^ activation in the mother cell, via the signalling protein SpoIVB. Mutations that bypass this signalling pathway result in premature activity of σ^K^ and defects in spore morphogenesis [25]. We have shown that the production of σ^G^ in the mother cell uncouples pro-σ^K^ processing from engulfment completion and leads to premature activation of σ^K^ because SpoIVB can activate processing of pro-σ^K^ directly in the cell where it is produced (S5 Fig). Accordingly, processing of pro-σ^K^ in the P_*sigF*_ mutant is detected earlier than in the wild type (Fig. 3C). By contrast, in the σ^E N100E^ strain, expression of a σ^K^-dependent *lacZ* fusion is reduced and delayed, presumably because pro-σ^K^ processing remains strictly dependent on the post-engulfment, σ^G^-dependent SpoIVB signalling from the forespore. Therefore, the silencing of σ^G^ in the mother cell by CsfB, along with the contributions of SpoIIAB and LonA, prevents premature activity of σ^K^.

### Discrimination by CsfB of the σ^F^/ σ^G^ and σ^E^/ σ^K^ pairs

We have previously shown that N45, an asparagine residue in region 2.2 of σ^G^, is a critical determinant for CsfB binding [20] (Fig. 1C). Not only is this residue conserved among *Bacillus* orthologues of σ^G^ but a glutamic acid residue, E39, is found at the homologous position of the CsfB-resistant σ^F^ protein in *B*. *subtilis*, and an acidic residue is invariably found at the equivalent position in σ^F^ orthologues of other *Bacillus* species. Importantly, the N45E substitution renders σ^G^ refractory to CsfB binding and conversely, the E39N variant of σ^F^ is susceptible to the anti-sigma factor. Residue N45 of σ^G^ is most likely involved in a direct contact with the β´ subunit of RNA polymerase, suggesting that CsfB, like other anti-sigma factors interferes with the σ/β ´ interaction [20]. Our *in vitro* transcription assays support this model, as CsfB is sufficient to inhibit σ^G^- and σ^F^-directed transcription at *bona fide* promoters *in vitro* (Fig. 5).

Our results now implicate a region encompassing regions 2.2 and 2.3 of σ^E^ in CsfB binding (Fig. 6), and an asparagine residue, N100, at the beginning of region 2.3, was found to be a key determinant (Fig. 7A-C). In a striking parallel with the σ^F^/σ^G^ pair, a homologous asparagine is found in all known orthologues of σ^E^ from related organisms (Fig. 7A). Despite the high degree of similarity between the σ^E^ and σ^K^ proteins across sporeformers [4,5,6,7], the homologous residue in the σ^K^ protein of *B*. *subtilis* is a glutamic acid, E93, and an acidic residue is invariably found among *Bacillus* orthologues of σ^K^ (Fig. 7A). Also reminiscent of the σ^F^ /σ^G^ pair, the N100E substitution makes σ^E^ refractory to CsfB, whereas the E73N variant of σ^K^ becomes susceptible to the anti-sigma factor (Fig. 7B to D).

Thus, binding of CsfB to any of the four sigma factors of sporulation is favored by the presence of a conserved asparagine in region 2.1 (for σ^F^/σ^G^) or 2.3 (σ^E^/σ^K^) and hindered by a glutamic acid at either position (Fig. 8B). Resistance to CsfB binding requires both positions to be occupied by acidic residues. This discrimination is essential for the control of gene expression during sporulation: it allows σ^G^ activity to be inhibited in the forespore prior to engulfment completion, while allowing σ^F^-dependent transcription; it also enables CsfB to antagonize σ^E^ and σ^G^ in the mother cell thus enforcing the cell type-specificity of σ^G^ and facilitating the timely switching from σ^E^ to the CsfB-immune σ^K^. Making σ^F^ or σ^K^ susceptible to CsfB or making σ^E^ or σ^G^ resistant to the anti-sigma factor interferes with proper temporal control and compartmentalization of the forespore and mother cell lines of gene expression.

The strict conservation of the discriminating residues in regions 2.1 or 2.3 of the sigma factors among *Bacillus* species (Fig. 7A) underscores the importance of CsfB for the activity of the cell type-specific sigma factors in sporeforming organisms closely related to *B*. *subtilis*.

### Binding of CsfB to σ^F^/σ^G^ and to σ^E^/ σ^K^

The crystal structure of the σ^70^-containing RNA polymerase holoenzyme from *Thermus aquaticus* shows that residue E189, the homologue of N45 in σ^G^, is involved in a direct contact with K159 in the β´ subunit [49,50,51,52]. We have argued that an asparagine residue could also contribute to the interaction with this site of β´, consistent with the view that one mechanism by which anti-sigma factors function is by occluding sigma-core binding interfaces [53,54]. Occluding a β´-binding surface is consistent with a role for CsfB in preventing the ectopic activity of σ^G^ in pre-divisional cells [20] or the premature activation of σ^G^ in the forespore [14]. In contrast, N100 in σ^E^ is located in the beginning of region 2.3, just upstream of a motif containing several conserved residues involved in promoter melting [55] (Fig. 7A and S6 Fig). This suggests that CsfB may interfere with σ^E^ function at a step during transcription initiation subsequent to closed complex formation. In contrast to σ^G^, whose ectopic or premature activation has to be prevented, σ^E^ is already engaged in transcription when CsfB appears in the mother cell. Therefore, the most effective mechanism for antagonizing σ^E^ activity may not be targeting a core-binding surface but rather a functional region that prevents the activity of the σ^E^-containing RNA polymerase holoenzyme. We note, however, that N100 is also close to several residues in region 2.2 that have been implicated in core binding in *E*. *coli* σ^70^, and are conserved in *B*. *subtilis* σ^A^ (highlighted in grey in Fig. 7A) (reviewed by [55]). Thus, binding of CsfB in the vicinity of N100 could also possibly occlude core-binding sites in region 2.2.

### Concluding remarks

The function of CsfB affects both the transcriptional and the cell-cell signalling networks that control spore differentiation (Fig. 8A). In the forespore, CsfB contributes to the inhibition of σ^G^ during early stages of development [14]. The σ^K^-dependent expression of *csfB* in the mother cell limits the activity of σ^E^ and prevents the ectopic activation of σ^G^ These activities of CsfB are each one part of redundant mechanisms that work to the same end. σ^K^ activity blocks expression of *sigE* by an unknown mechanism [11,12], and ectopic activity of σ^G^ is limited by SpoIIAB and LonA in the mother cell. Our discovery that CsfB binds and inhibits the activity of σ^E^ also leads us to propose that in the forespore, CsfB binding to σ^E^ explains how CsfB functions as one of the several partially redundant mechanisms described by Piggot and co-workers [34] that prevent ectopic activity of σ^E^ in the forespore (see also Fig. 1B). That several of the major roles for CsfB (*i*.*e*., preventing ectopic expression of σ^G^ and σ^E^, and facilitating the transition between σ^E^ and σ^K^) involve partially redundant mechanisms speaks to the importance of controlling these processes, and provides an explanation for why CsfB is highly conserved among sporeformers [6,21]. Moreover, these redundancies may also explain why the phenotype of a *csfB* mutant is relatively mild. The original work on CsfB found no decernable phenotype [23], whereas subsequent work from Stragier’s group [22] described a small but significant germination defect, but only when a Δ*csfB* mutation was combined with an allele causing premature transcription of the gene for σ^G^ [22]. The P_*sigF*_ and P_*sigK*_ strains formed spores with the same efficiency (77% and 88%, respectively) as the wild type strain (83%). However, we did observe a difference in the protein composition of the spore surface layers between σ^E N100E^ and wild type spores (S7A Fig). It is unknown whether this phenotype provides some of the selective pressure for maintaining CsfB during the evolution of sporeformers, but we note the important role of the spore surface layers in mediating many of the environmental interactions of spores, including with cells of host organisms [42]. On the other hand, the evolution of redundant mechanisms to control key steps in development may have promoted the stabilization or canalization of the cell type-specific patterns of gene expression that led to the establishment of the endospore differentiation pathway [56].

The role of CsfB in favouring the switch from early to late stages in spore development is likely to be conserved among *Bacillus* species and other sporeformers. Importantly, at least in *Bacillus* species, the actions of the CsfB anti-sigma rely strongly on its ability to discriminate between the highly similar σ^F^/σ^G^ and σ^E^/σ^K^ pairs (Fig. 8B). Interestingly, CsfB is not found in some Clostridia, a more distantly related class of sporeforming organisms, including the human intestinal pathogen *C*. *difficile*. The gene regulatory network for sporulation has been recently characterized in detail for *C*. *difficile*. It is interesting to note that in this organism, σ^G^ is active prior to engulfment completion and that σ^E^ remains active until the late stages of sporulation [57,58,59]. The looser temporal control of σ factor function in *C*. *difficile* and presumably also in other Clostridia may stem, at least in part, from the absence of a CsfB orthologue. Moreover, it may be interesting to investigate whether this looser regulation results in greater heterogeneity of morphology among spores of certain *Clostridial* species than among spores of *Bacillus* species.

## Material and Methods

### Strains and general techniques

Except for strain MBS3656 ([60]; see below), all other *B*. *subtilis* strains used in this work are congenic derivatives of the Spo^+^ strains MB24 (*trpC2 metC3*) or PY79 (prototrophic). Their construction is detailed in S1 Text, and they are listed, with their genotypes, in S1 Table. All plasmids used in this work are described in S1 Text. Primers used for plasmid construction, mutagenesis or sequencing are listed, with their sequences, in S2 Table. LB medium was used for routine growth or maintenance of *E*. *coli* and *B*. *subtilis*, and sporulation was induced by growth and exhaustion in Difco sporulation medium (DSM) or by the re-suspension method [43].

### Purification of RNA polymerase, CsfB and the sigma factors σ^A^, σ^F^, σ^E^, σ^G^ and σ^K^

The strains for sigma factor and CsfB expression were cultured in LB at 37°C with Ampicillin (100μg/ml). Arabinose was added to final concentration of 0.5% at an OD_600_ of 0.6. The culture was allowed to incubate for another 1–2 hours before cells were harvested and stored at -80°C. Sigma factors and CsfB were purified using Qiagen Ni-NTA. The Ni-NTA-affinity purified protein was analyzed on a 10% SDS-PAGE. Fractions containing the sigma factor or CsfB were pooled and dialyzed against dialysis buffer (50mM Tris, 100mM NaCl, 3mM β-mercapatoenthanol). The dialyzed protein was chromatographed through a GE HiLoad 16/60 Superdex 75 column, fractions (1 ml) collected, and those containing the desired protein pooled and dialyzed against 1X *in vitro* transcription reaction buffer (50mM Tris, 100mM KCl, 10% Glycerol, 10mM DTT). Protein concentration was measured using a Bradford assay (Bio-Rad, Hercules, CA). Protein aliquots were stored at -20°C.

RNA polymerase (RNAP) was purified from *B*. *subtilis* strain MH5636 [60] cultured in LB with chloramphenicol (5 μg/ml) to an OD_600_ of 1.0. Cells was harvested by centrifugation and lysed by treatment with lysozyme (5mg/ml) for 30 minutes at 4°C followed by passage twice through a French pressure cell at a pressure of 20,000 psi. Core RNA polymerase was purified essentially as described by Burgess and colleagues [55]. Briefly the cell lysate supernatant was first purified using Qiagen Ni-NTA affinity column. Fractions containing RNAP were pooled and subjected to chromatography on a HiLoad 16/60 Superdex 200 gel filtration column. Fractions containing RNA polymerase were then purified by ion-exchange exchange chromatography on a GE Mono Q 5/50 GL column. The purity of RNAP was assayed on a 4–20% SDS-PAGE. The concentration of purified RNAP was determined using a Bradfors assay (Bio-Rad, Hercules, CA). RNAP aliquots were at -20°C.

### *In vitro* transcription assays

Core RNAP (13 nM) was incubated in transcription reaction buffer (40 mM Tris/HCl (pH 8), 50 mM KCl, 10 mM MgCl2, 10mM DTT, 50 mg/ml acetylated BSA, 0.5 ml RNase-inhibitor [61] on ice for 30 minutes with 130 nM sigma factor and 1.0 μg of a DNA template purified through a CsCl/ethidium bromide density gradient and cleaved with a restriction enzyme (BamHI or HindIII). After 10 min at 37°C, ATP, CTP, and GTP were added to a final concentration of 1.0 mM and 5 μCi UTP was added to 50 μl transcription reactions. The mixture was incubated for 10 minutes at 37^7^°C after which 0.2 mM unlabeled UTP was added, followed by a further incubation for 10 minutes at 37°C. Finally, the reaction mixture was extracted with phenol-chloroform and the nucleic acids precipitated and electrophoretically resolved through 10%(w/v) polyacrylamide gels containing 7M urea. In the reactions containing CsfB, the anti-sigma factor was added at an amount equal to sigma (130 nM) or 2, 3, 4 or 8-fold excess (indicated as 1x to 8x in Fig. 6C and D), and incubated with sigma for 20 min at room temperature. RNAP was added and the reactions conducted as described above, and incubated with sigma for 20 min at room temperature. RNAP was added and the reactions conducted as described above.

### Transcriptional profiling of sporulating cells

These analyses were carried out as previously described in Cozy et al. [62] and Winkelman et al. [63]. Briely, the arrays obtained form Agilent include 15,744 probes covering the annotated protein-coding genes of *B*. *subtilis* [64] and the small non-coding RNAs reported in Rasmussen et al. [65] and Irnov et al [66]. The arrays were designed using the Agilent eArray application. Strains AH6825 (P_*csfB*_-*csfB*) and AH6827 (P_*sigF*_-*csfB*) were sporulated by resuspension in Sterlini-Mandelstam medium and samples collected at hour 3 of sporulation. Cells pellets were recovered by centrifugation after mixing with an equal volume of cold methanol. Total RNA was recovered using a hot acid-phenol protocol followed by clean-up using the Qiagen RNeasy kit. cDNA was synthesized from the purified total RNA and labelled using the Agilent Fairplay III kit. After hybridization and washes using standard protocols, the arrays were scanned in an Agilent Technologies DNA microarray scanner with Surescan high-resolution technology. Processed signal from the Agilent software was subjected to standard lowess normalization using Bioconductor run in R and the geometric mean of the probes was used to give the final value for each gene and nc-RNA.

### Fluorescence *in situ* hybridization (FISH)

For RNA-FISH, we used the following protocol: cells growing in Re-suspension medium were fixed in Histochoice solution (Ameresco) for 15 min at room temperature and 30 min on ice. The samples were centrifuged three times at 3.000xg for 2 min and washed in 1xDEPC-treated PBS. The cell pellets were resuspended in 100 μl GTE buffer (50 mM glucose, 20 mM Tris-HCl pH 7.5, 10 mM EDTA pH 8). Sixteen microlitres of a 10 mg/ml lysozyme solution (GTE, 4 mM vanadyl ribonucleoside complex (VRC), 10 mg/ml lysozyme) were added to 48 μl of cell suspension. The mixture was immediately placed onto poly-L-lysine-coated multi-well slides, and incubated for 10 min at room temperature. The excess liquid was aspirated and the slides were left 1 min to dry before putting them in -20°C methanol for 10 min. Next, the slides were dipped in -20°C acetone for 30 s. Once the slides were dry, they were incubated at 37°C for 30–60 min in a 40% formamide solution (40% formamide, 2x DEPC-treated saline-sodium citrate buffer (SSC)). LNA probe was added to the hybridization solution I (80% formamide, 1 mg/ml *E*. *coli* tRNA, 2x DEPC-treated SSC, 70 μg/ml calf-thymus DNA) at a final concentration of 250 nM, and incubated at 80°C for 5 min before mixing with the hybridization solution II (20% dextran sulphate, 4mM VRC, 40U RNase inhibitor, 0.2% RNase-free BSA, 2x DEPC-treated SSC) in a 1:1 ratio. The hybridization solution (25 μl) was added to each well of the slide and hybridized for 2 h. The slides were then washed twice in 50% formamide and 2x DEPC-treated SSC solution for 30 min and briefly rinsed five times in DEPC-treated PBS. DAPI was added to a final concentration of 10 μg/ml in SlowFade solution (Invitrogen) were added to each well and the slide was covered and sealed using clear nail polish. The slides were either visualized immediately or stored in the dark at -20°C. The sequence of the probes, which were labeled with Cy3, is given S2 Table.

### Fluorescence microscopy

Samples (0.6 ml) of cultures were collected, resuspended in 0.2 ml of phosphate-buffered saline (PBS) and the membrane dye FM4–64 (Molecular Probes) added to a final concentration of 10 μg ml^−1^. Cells were observed on slides padded with 1.7% agarose. Images were acquired on Leica fluorescence microscopes DMR2A and DM6000B equipped with a Cool Snap HQ camera (Roper Scientific, Arizona, USA) and an iXonEM+ 885 camera (Andor Technology, Connecticut, USA), respectively, or on a Nikon E1000 microscope equipped with an Orca-ER camera (Hamamatsu Corporation, New Jersey, USA), using 63x or 100x lens objective plus an additional 1.6X optavar, phase-contrast optics and standard filters for visualization of GFP and FM4–64. Images were acquired using Metamorph (Molecular Devices, Berks, UK) and processed for publication using Photoshop (Adobe).

### Accession numbers

The Genbank accession numbers for the sequences represented in Fig. 8A and S3C Fig, are as follows. For σ^G^, CAB13407.1 (*B*. *subtilis*) and for σ^F^, CAB14277.1 (*B*. *subtilis*). For σ^A^, NP_390399.2 (*B*. *subtilis*), P00579.2 (*E*. *coli*), AAG36964.1 (*T*. *aquaticus*); WP_011172619.1 (*T*. *thermophilus*). For σ^E^, NP_243422.1 (*B*. *halodurans*), YP_175847.1 (*B*. *clausii*), NP_389415.2 (*B*. *subtilis*), ABS73878.1 (*B*. *amyloliquefaciens*); YP_006713129.1 (*B*. *licheniformis*); YP_085245.1 (*B*. *cereus*), ABY76243.1 (*B*. *thuringiensis*). For σ^K^, NP_242151.1 (*B*. *halodurans*), YP_175113.1 (*B*. *clausii*), WP_003237137.1 (*B*. *subtilis*), WP_015240253.1 (*B*. *amyloliquefaciens*); YP_079918.1 (*B*. *licheniformis*); YP_085663.1 (*B*. *cereus*), ABY76244.1 (*B*. *thuringiensis*). For the β´subunit of RNAP polymerase: WP_003225772.1 (*B*. *subtilis*); YP_491473.1 (*E*. *coli*); WP_003043700.1 (*T*. *aquaticus*); YP_005641350.1 (*T*. *thermophilus*).

## Supporting Information

S1 Fig *csfB* expression during sporulation. **A**: immunoblot analysis of CsfB-GFP accumulation during sporulation. Samples from sporulating cultures were collected at the time of ressuspension in resuspension medium and at hourly intervals thereafter, as indicated by the numbers above the lanes. Whole cell extracts were prepared and the proteins (30 μg samples) were resolved on 15% SDS-PAGE gels and subject to immunoblot analysis with an anti-GFP antibody. Arrows mark the position of CsfB-GFP. **B**: expression of transcriptional *csfB*-*lacZ* fusions inserted at the *amyE* locus during sporulation. The following strains were included in the analysis: a wild type (bearing no *lacZ* fusion, to estimate background levels), a fusion of the σ^F^-type *csfB* promoter to *lacZ* in the wild type background and in a *sigG* deletion mutant, a fusion of the σ^K^-type *csfB* promoter to *lacZ* in the wild type background and in a *sigK* deletion mutant. Cultures of the various strains were induced to sporulate by the resuspension method, and samples withdrawn at the indicated times, in hours, after the onset of sporulation (denoted as T0), and assayed for β-galactosidase activity (shown in Miller units).
(TIF)

S2 Fig Purification of proteins for *in vitro* transcription assays. **A**: shown is a 15% SDS-PAGE gel separation of core RNA polymerase purified from *B*. *subtilis* cells (E), and CsfB, σ^E^, σ^F^, σ^G^, σ^K^, and σ^A^ (arrows) overproduced and purified from *E*. *coli*. Asterisks indicate possible degradation products. The position of molecular weight markers (M, in kDa) is shown on the left side of the panel. **B**: schematic representation of PCR products bearing the control regions and part of the coding sequence of the *spoIID* and *spoIIIA* genes, used as templates for *in vitro* transcription reactions with RNA polymerase containing σ^E^ and σ^K^, respectively. The size of the expected run-off products is indicated in nucleotides. **C**: *in vitro* transcription reactions with RNA polymerase containing σ^E^ and the *spoIID*, *spoIIIA P*_*1*_ or *spoIIIA P*_*2*_ templates in the absence (“-“) or in the presence of varying concentrations of CsfB (indicated as molar ratio relative to RNA polymerase). The arrows, color-coded as the templates in panel B, point to the position of the resulting run-off products. The position of molecular weight markers (in nucleotides) is shown on the left side of the panel.
(TIF)

S3 Fig Mutations in the σ^F^-dependent *csfB* promoter leads to σ^G^ activity in pre-divisional sporangia. **A**: shows the effect of *csfB* mutations on the activity of σ^G^, monitored by means of an *sspE-lacZ* transcriptional fusion. Cells harbouring the indicated mutations were grown in Difco sporulation medium (DSM) and samples collected at the indicated times, in hours, before or after the onset of stationary phase (or T0), and assayed for β-galactosidase activity (shown in Miller units). **B**: shows the effect of *csfB* mutations on the activity of σ^G^, monitored by means of an *sspE-cfp* transcriptional fusion. Cells were grown in DSM, samples collected 2 hours after T0, stained with the membrane dye FM4–64 and examined by fluorescence microscopy. Cells showing no signs of asymmetric septation and a strong CFP signal are indicated by yellow arrows. The numbers in the CFP panels indicate the percentage of cells with a similar pattern of CFP fluorescence. Scale bar, 1 μm.
(TIF)

S4 Fig In a P_*sigF*_-*csfB* mutant, expression of *sspE* takes place in the mother cell, prior to engulfment completion. **A**: strains carrying a transcriptional fusion of the σ^G^-dependent P_*sspE*_ promoter to *cfp* in either the wild type or P_*sigF*_-*csfB* backgrounds were indiced to sporulate by the re-suspension method. Samples were collected 3 hours after re-suspension, the cells stained with the membrane dye FM4–64 and examined by fluorescence microscopy. Yellow or white arrows point the fluorescence signal in the forespore (following engulfment completion) or in the mother cell (prior to engulfment completion, as judged from the absence of forespore labeling by the FM4–64 dye). **B**: quantification of the fluorescence signal obtained for the wild type or P_*sigF*_-*csfB* strains expressing P_*sspE*_-*cfp*. The signal was only measured in the mother cell, in cells that had not completed forespore engulfment. Fluorescence intensity is shown in arbitrary units.
(TIF)

S5 Fig CsfB concurs with SpoIIAB and LonA to antagonize σ^G^ in the mother cell. **A**: shows the effect of insertional mutations in *lonA* or *csfB*, or a point mutation in the gene for σ^G^ (*sigG E156K*) on the activity of σ^G^ (monitored by means of an *sspE-lacZ* fusion) when produced in the mother cell from the *spoIID* promoter. **B**: the panel illustrates the expression of a fusion of the σ^K^-dependent *gerE* promoter to *lacZ*, during sporulation, in strains expressing either *sigG* or *sigG E156K* from the mother cell-specific *spoIID* promoter. In A and B, cultures were grown in DSM, samples withdrawn at the indicated times in hours after the onset of sporulation (denoted as T0), and assayed for β-galactosidase activity (in Miller units). All strains, in A and B, carry an in-frame deletion of the *sigG* gene and a copy of *sigG* or *sigG E156K* under P_*spoIID*_ or P_*sigG*_ control, inserted at the *amyE* locus. **C**: illustrates the activation of the pro-σ^K^ activation complex (red circle) by SpoIVB, produced in the mother cell, and the role of SpoIIAB, LonA and CsfB in reducing the potential for σ^G^ activity in the mother cell. The composite negative feedback loop established by CsfB and σ^G^ is similar to the one that limits the activity of the sigma factor in pre-divisional cells [20]. Transcriptional and protein-protein interactions are shown in black and red, respectively.
(TIF)

S6 Fig Crystal structure of σ^A^-containing RNA polymerase. **A**: The figure shows the crystal structure of σ^A^-containing RNA polymerase holoenzyme from *T*. *thermophilus* (pdb: 1IW7), with the residues homologous to N45 in *B*. *subtilis* σ^G^ (E202) and E100 in σ^E^ (E240) highlighted in stick representation. The two α subunits are represented in magenta and purple, β is shown in grey, β´ in green, and σ in yellow. **B**: expansion of the region encircled in A. Note that E202 forms a salt bridge with R159 in β´, whereas E240 is surface exposed. The image was rotated 180° along the *y* axis to generate the bottom panel. The images were rendered with Pymol (www.pymol.org). **C**: The figure shows an alignment of the primary structures of a segment of the β´subunit of RNA polymerase from *B*. *subtilis*, *E*. *coli*, *T*. *aquaticus* and *T*. *thermophilus*. E202 in *T*. *thermophilus* and *T*. *aquaticus* σ^A^ (in region 2.1) and presumably N45 in *B*. *subtilis* σ^G^ make a contact with the Arg residue marked by a dot in the β´ subunit of RNA polymerase. Note that this residue is not conserved in the β´ subunit from *E*. *coli* (see also [20]). The *T*. *aquaticus* holoenzyme is not show in panels A and B but is highly similar to the *T*. *thermophilus* enzyme, and the relevant residues in σ and β´are identical. The alignment was produced with ClustalW (www.ebi.ac.uk) and the accession numbers given at the end of the Material and Methods section.
(TIF)

S7 Fig The N100E mutation in *sigE* affects the assembly of the spore surface layers. **A**: spores of the indicated strains were purified on density gradients and proteins extracted by treatment with an SDS/DTT-containing buffer (top) or NaOH (bottom). The red arrows indicate the position of proteins that are more extractable from spores produced by the strain producing the N100E form of σ^E^ (see also S1 Text). **B**: spores of the indicated strains, identified by the color code used in panel A, were induced to germinate by exposure to L-Alanine (10 mM; close symbols). Spores in control samples (open symbols) were heat activated but not exposed to L-Alanine. The rate and extent of germination monitored by following the drop in the optical density of the suspension (OD) at 580 nm, over time, and expressed as the percentage of the initial OD_580_.
(TIF)

S1 Text Supplemental materials and methods and supplemental results and discussion. (DOCX)

S1 Table *Bacillus subtilis* strains used in this work (PDF). (DOCX)

S2 Table Oligonucleotides used in this work (PDF). (DOCX)

S3 Table Transcriptional profiling of sporulating cells (PDF). (PDF)
