# Supplementary figures and images for: Dual-Specificity Anti-sigma Factor Reinforces Control of Cell-Type Specific Gene Expression in Bacillus subtilis
Source: PLoS Genet. 2015 Apr 2;11(4):e1005104. doi: 10.1371/journal.pgen.1005104 (PMC4383634; doi:10.1371/journal.pgen.1005104)

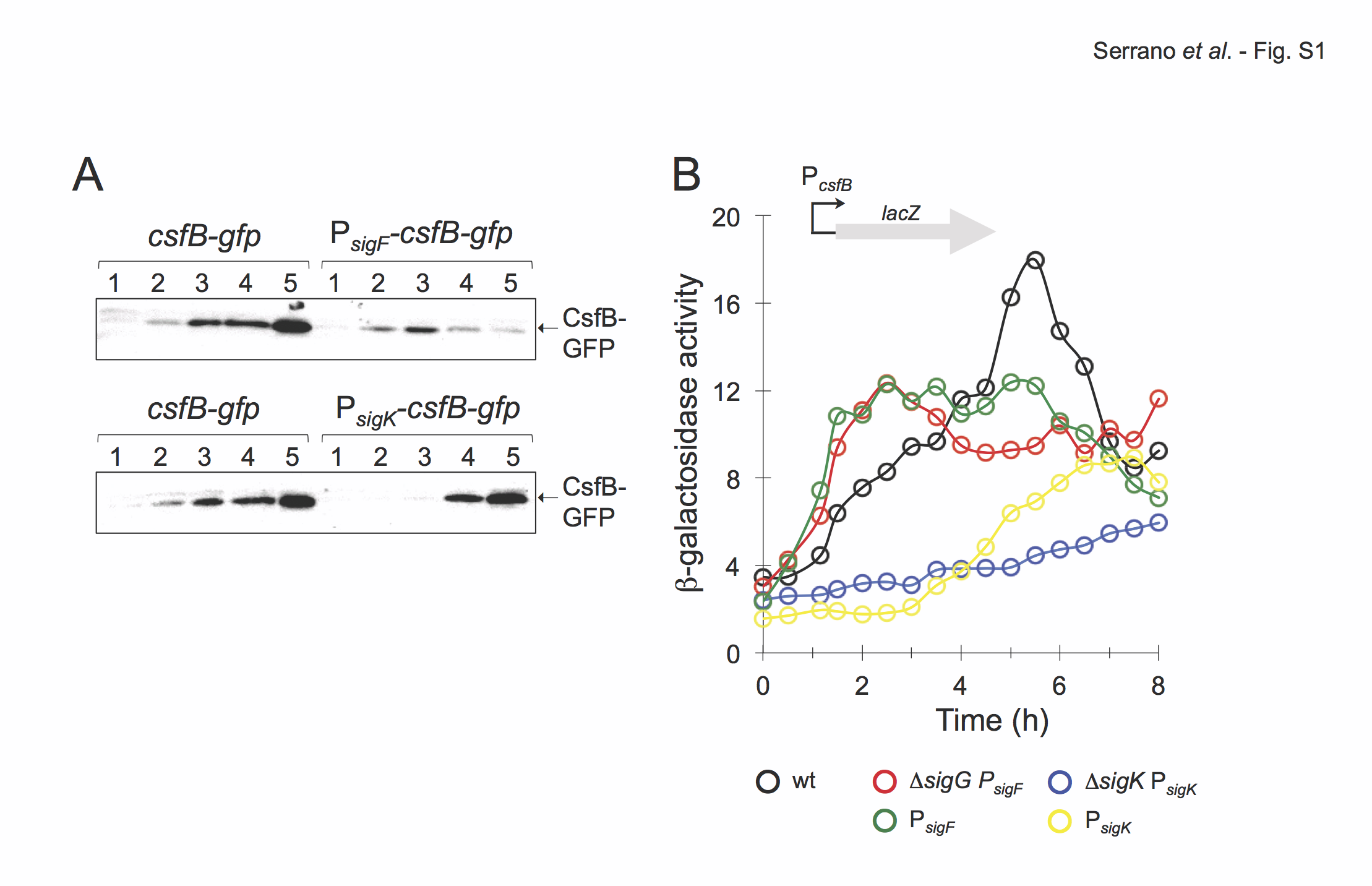

Supplement: S1 Fig — A: immunoblot analysis of CsfB-GFP accumulation during sporulation. Samples from sporulating cultures were collected at the time of ressuspension in resuspension medium and at hourly intervals thereafter, as indicated by the numbers above the lanes. Whole cell extracts were prepared and the proteins (30 μg samples) were resolved on 15% SDS-PAGE gels and subject to immunoblot analysis with an anti-GFP antibody. Arrows mark the position of CsfB-GFP. B: expression of transcriptional csfB-lacZ fusions inserted at the amyE locus during sporulation. The following strains were included in the analysis: a wild type (bearing no lacZ fusion, to estimate background levels), a fusion of the σF-type csfB promoter to lacZ in the wild type background and in a sigG deletion mutant, a fusion of the σK-type csfB promoter to lacZ in the wild type background and in a sigK deletion mutant. Cultures of the various strains were induced to sporulate by the resuspension method, and samples withdrawn at the indicated times, in hours, after the onset of sporulation (denoted as T0), and assayed for β-galactosidase activity (shown in Miller units). (TIF) [file pgen.1005104.s001.tif]

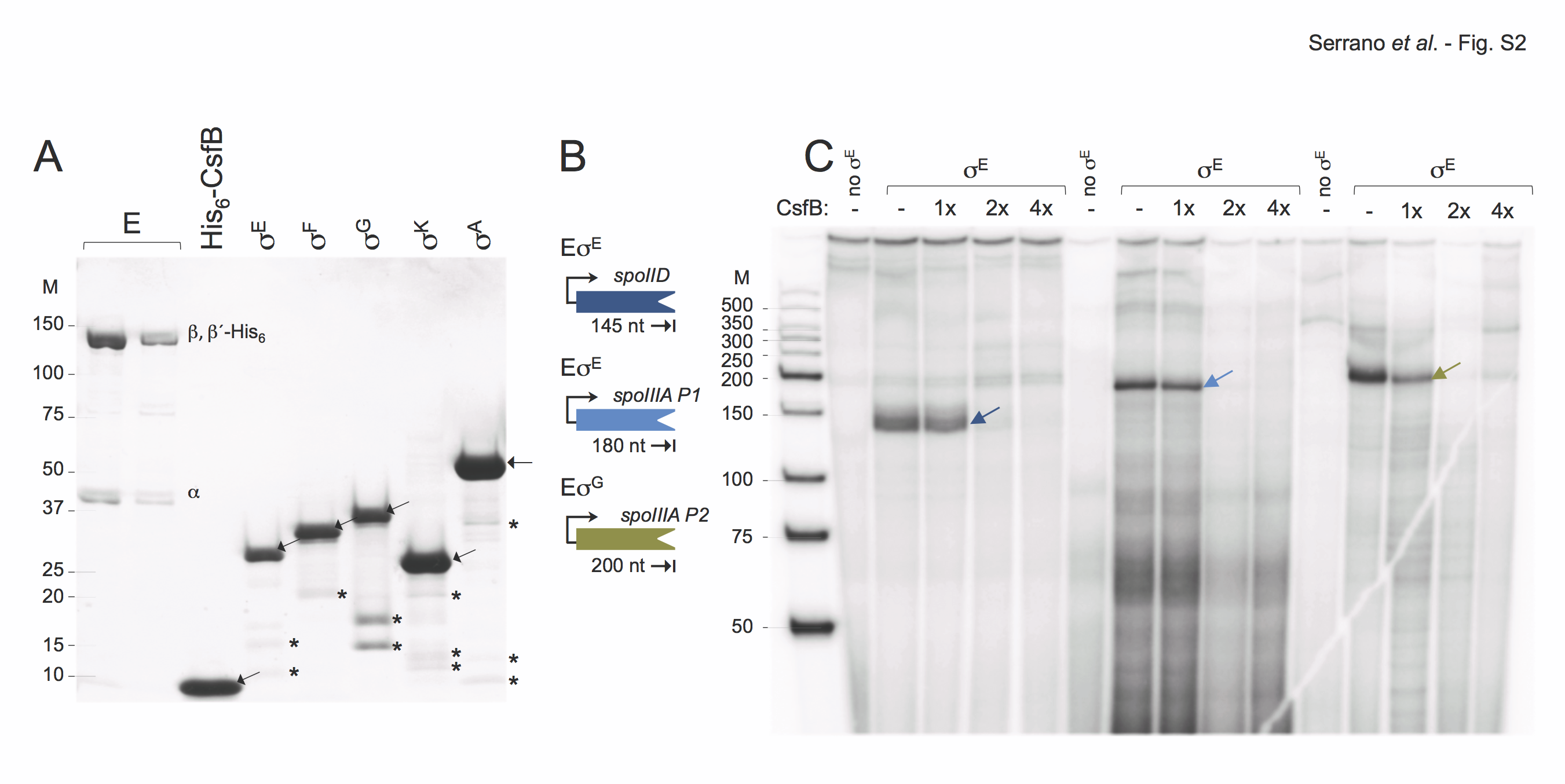

Supplement: S2 Fig — A: shown is a 15% SDS-PAGE gel separation of core RNA polymerase purified from B. subtilis cells (E), and CsfB, σE, σF, σG, σK, and σA (arrows) overproduced and purified from E. coli. Asterisks indicate possible degradation products. The position of molecular weight markers (M, in kDa) is shown on the left side of the panel. B: schematic representation of PCR products bearing the control regions and part of the coding sequence of the spoIID and spoIIIA genes, used as templates for in vitro transcription reactions with RNA polymerase containing σE and σK, respectively. The size of the expected run-off products is indicated in nucleotides. C: in vitro transcription reactions with RNA polymerase containing σE and the spoIID, spoIIIA P 1 or spoIIIA P 2 templates in the absence (“-“) or in the presence of varying concentrations of CsfB (indicated as molar ratio relative to RNA polymerase). The arrows, color-coded as the templates in panel B, point to the position of the resulting run-off products. The position of molecular weight markers (in nucleotides) is shown on the left side of the panel. (TIF) [file pgen.1005104.s002.tif]

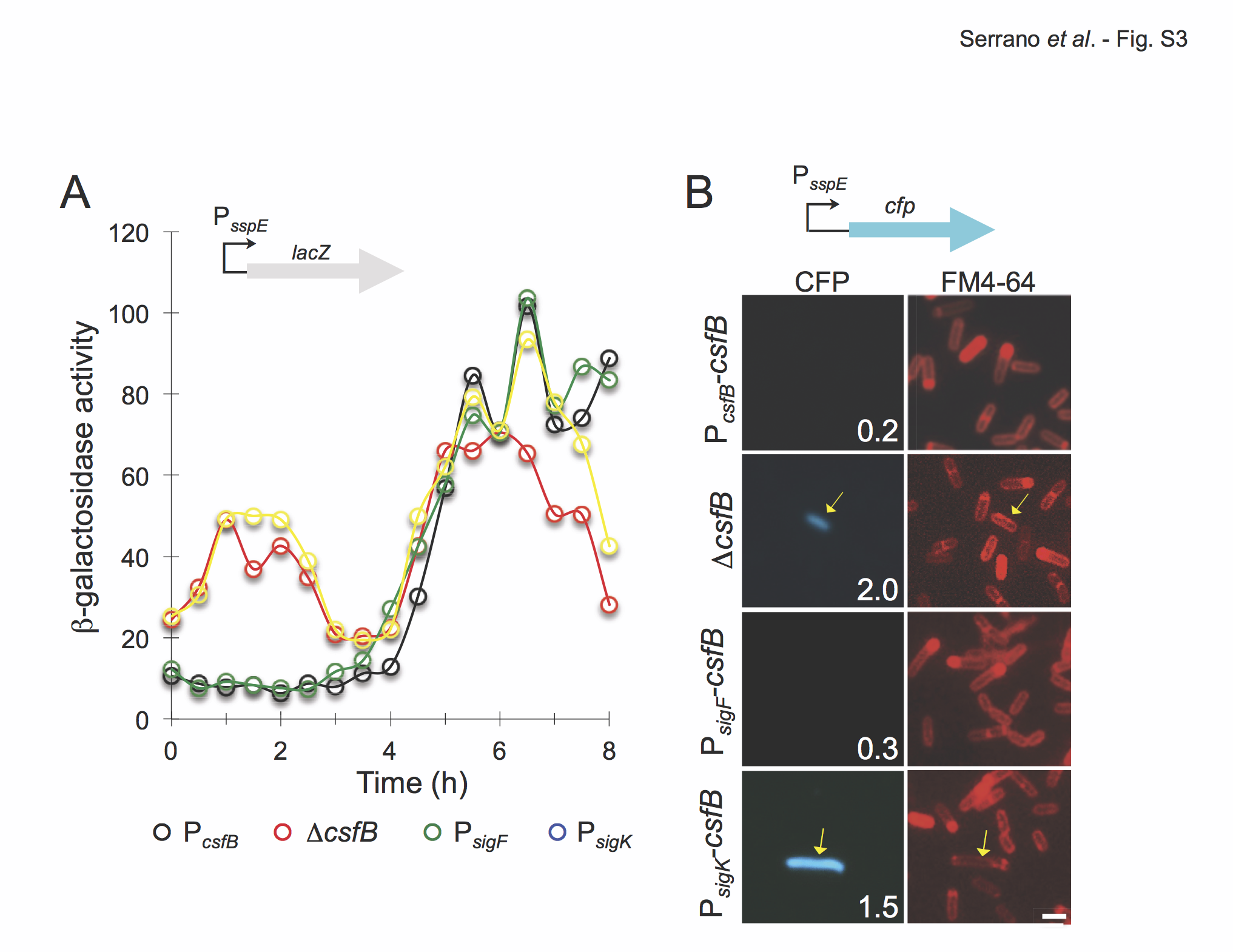

Supplement: S3 Fig — A: shows the effect of csfB mutations on the activity of σG, monitored by means of an sspE-lacZ transcriptional fusion. Cells harbouring the indicated mutations were grown in Difco sporulation medium (DSM) and samples collected at the indicated times, in hours, before or after the onset of stationary phase (or T0), and assayed for β-galactosidase activity (shown in Miller units). B: shows the effect of csfB mutations on the activity of σG, monitored by means of an sspE-cfp transcriptional fusion. Cells were grown in DSM, samples collected 2 hours after T0, stained with the membrane dye FM4–64 and examined by fluorescence microscopy. Cells showing no signs of asymmetric septation and a strong CFP signal are indicated by yellow arrows. The numbers in the CFP panels indicate the percentage of cells with a similar pattern of CFP fluorescence. Scale bar, 1 μm. (TIF) [file pgen.1005104.s003.tif]

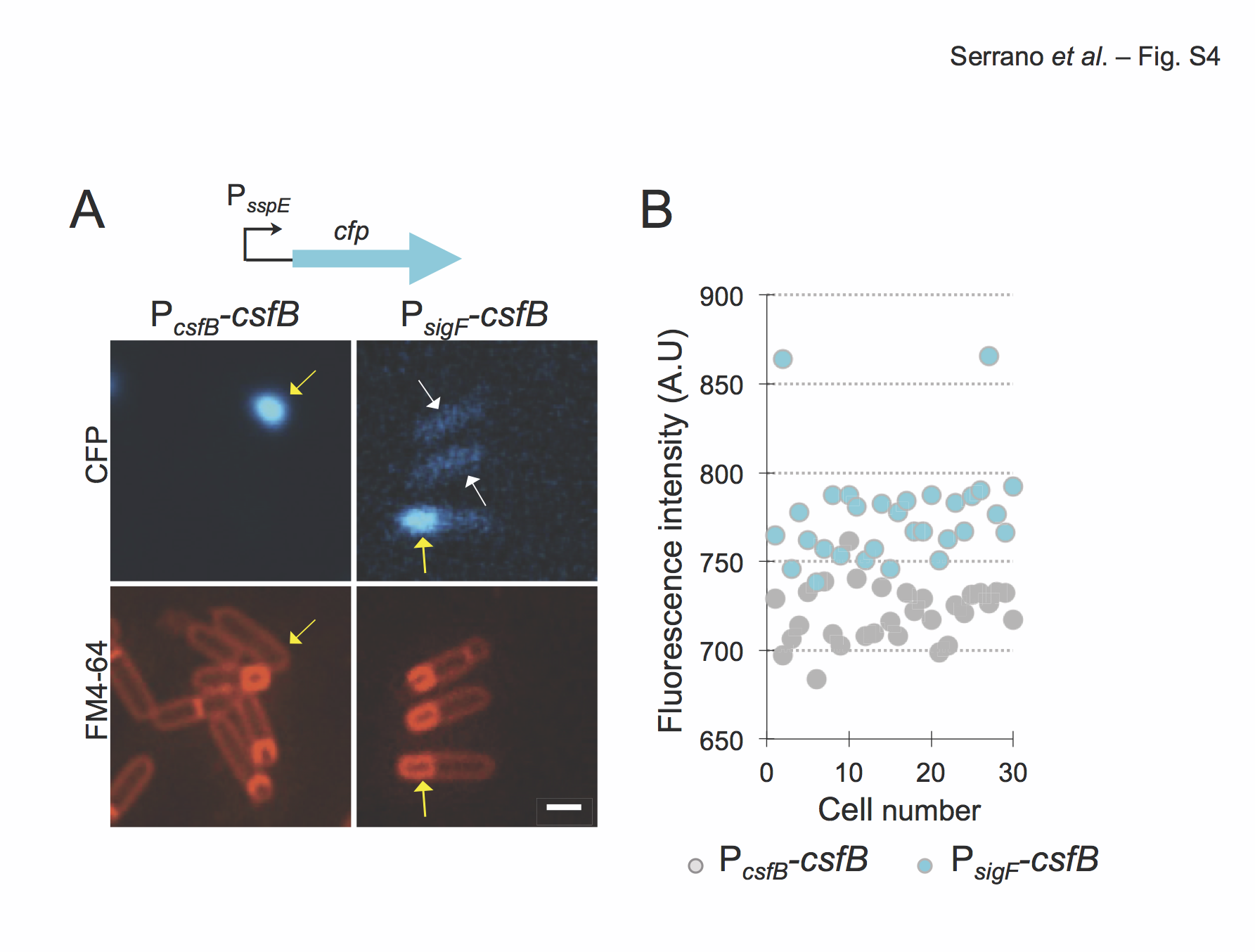

Supplement: S4 Fig — A: strains carrying a transcriptional fusion of the σG-dependent PsspE promoter to cfp in either the wild type or PsigF-csfB backgrounds were indiced to sporulate by the re-suspension method. Samples were collected 3 hours after re-suspension, the cells stained with the membrane dye FM4–64 and examined by fluorescence microscopy. Yellow or white arrows point the fluorescence signal in the forespore (following engulfment completion) or in the mother cell (prior to engulfment completion, as judged from the absence of forespore labeling by the FM4–64 dye). B: quantification of the fluorescence signal obtained for the wild type or PsigF-csfB strains expressing PsspE-cfp. The signal was only measured in the mother cell, in cells that had not completed forespore engulfment. Fluorescence intensity is shown in arbitrary units. (TIF) [file pgen.1005104.s004.tif]

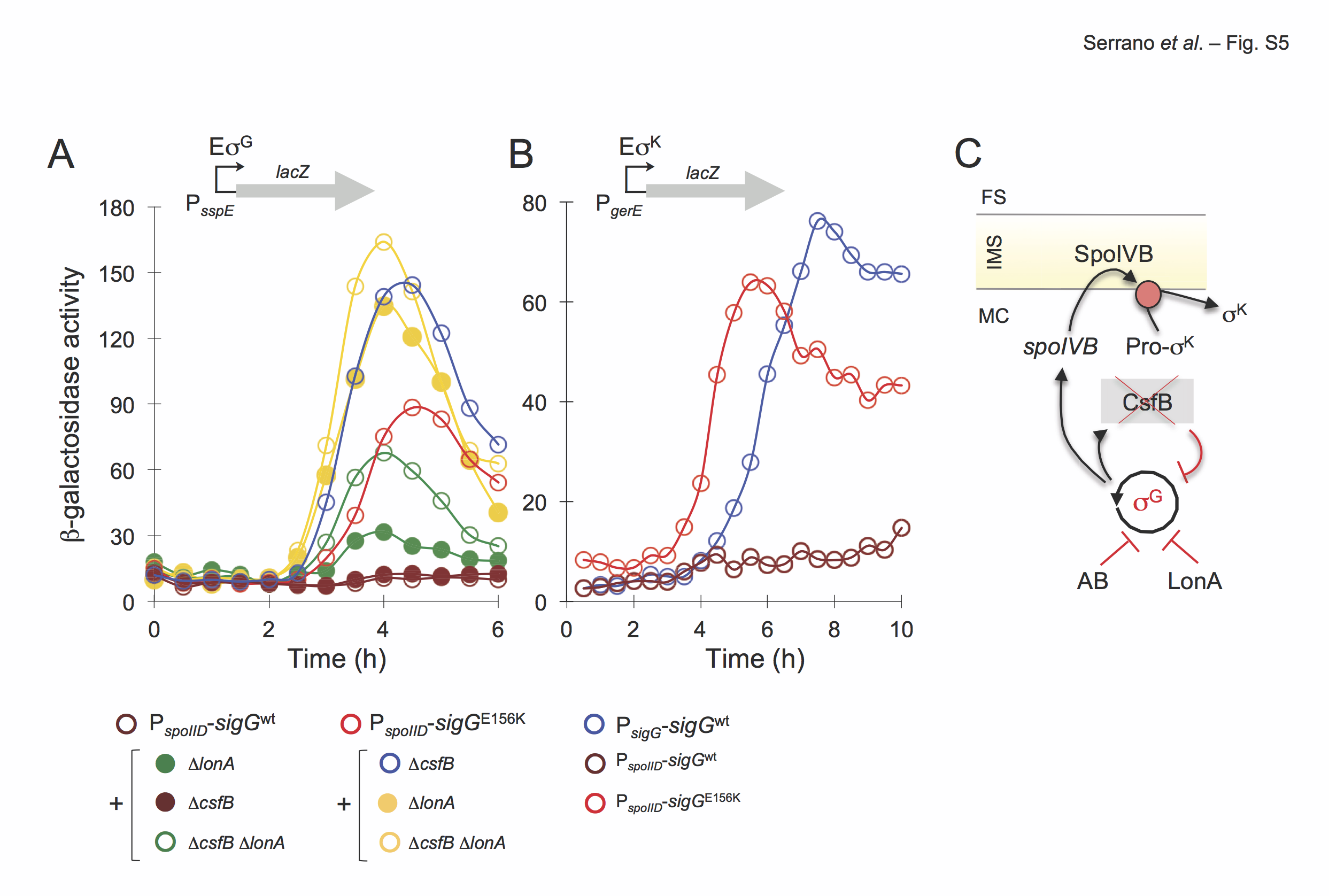

Supplement: S5 Fig — A: shows the effect of insertional mutations in lonA or csfB, or a point mutation in the gene for σG (sigG E156K) on the activity of σG (monitored by means of an sspE-lacZ fusion) when produced in the mother cell from the spoIID promoter. B: the panel illustrates the expression of a fusion of the σK-dependent gerE promoter to lacZ, during sporulation, in strains expressing either sigG or sigG E156K from the mother cell-specific spoIID promoter. In A and B, cultures were grown in DSM, samples withdrawn at the indicated times in hours after the onset of sporulation (denoted as T0), and assayed for β-galactosidase activity (in Miller units). All strains, in A and B, carry an in-frame deletion of the sigG gene and a copy of sigG or sigG E156K under PspoIID or PsigG control, inserted at the amyE locus. C: illustrates the activation of the pro-σK activation complex (red circle) by SpoIVB, produced in the mother cell, and the role of SpoIIAB, LonA and CsfB in reducing the potential for σG activity in the mother cell. The composite negative feedback loop established by CsfB and σG is similar to the one that limits the activity of the sigma factor in pre-divisional cells [20]. Transcriptional and protein-protein interactions are shown in black and red, respectively. (TIF) [file pgen.1005104.s005.tif]

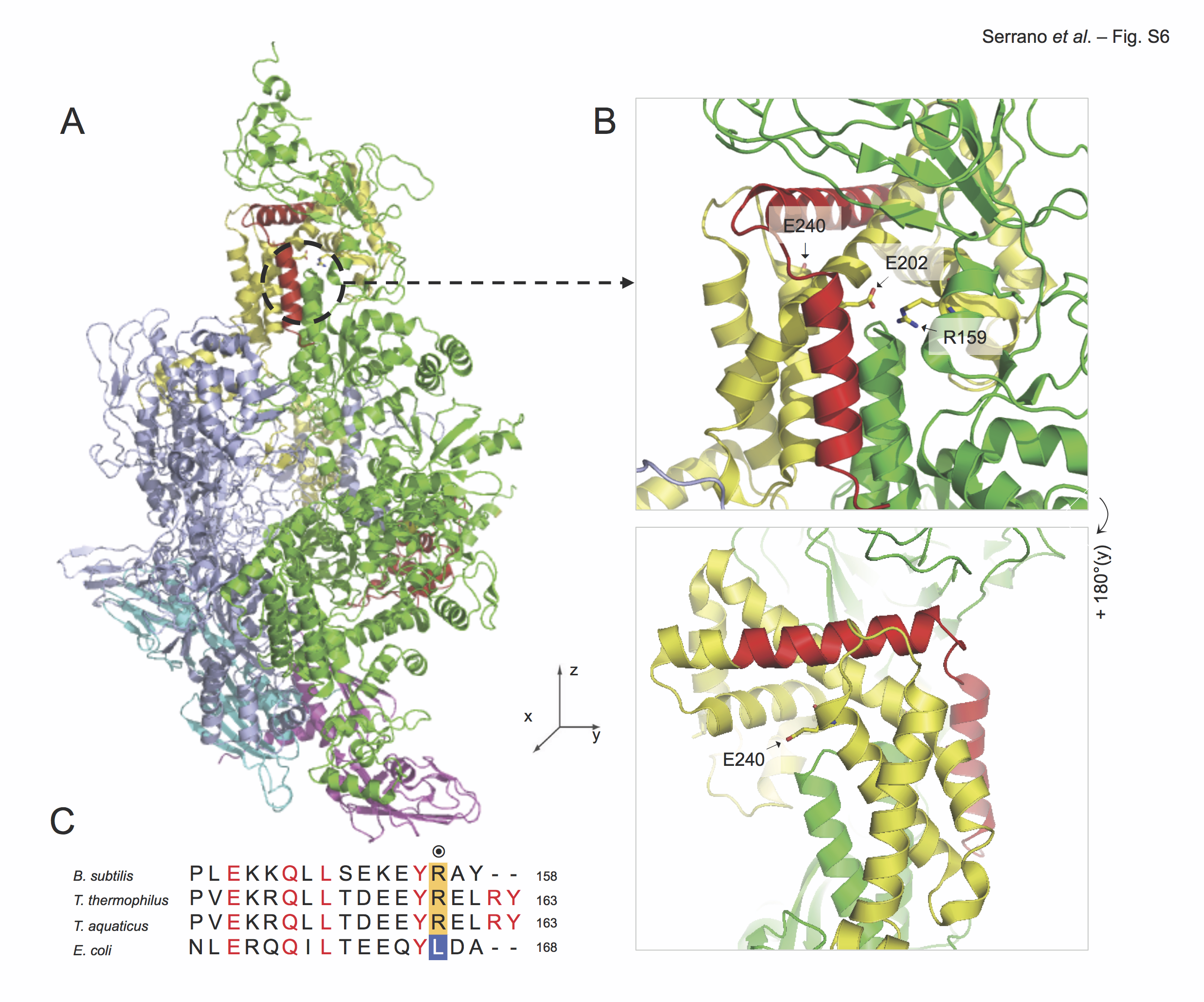

Supplement: S6 Fig — A: The figure shows the crystal structure of σA-containing RNA polymerase holoenzyme from T. thermophilus (pdb: 1IW7), with the residues homologous to N45 in B. subtilis σG (E202) and E100 in σE (E240) highlighted in stick representation. The two α subunits are represented in magenta and purple, β is shown in grey, β´ in green, and σ in yellow. B: expansion of the region encircled in A. Note that E202 forms a salt bridge with R159 in β´, whereas E240 is surface exposed. The image was rotated 180° along the y axis to generate the bottom panel. The images were rendered with Pymol (www.pymol.org). C: The figure shows an alignment of the primary structures of a segment of the β´subunit of RNA polymerase from B. subtilis, E. coli, T. aquaticus and T. thermophilus. E202 in T. thermophilus and T. aquaticus σA (in region 2.1) and presumably N45 in B. subtilis σG make a contact with the Arg residue marked by a dot in the β´ subunit of RNA polymerase. Note that this residue is not conserved in the β´ subunit from E. coli (see also [20]). The T. aquaticus holoenzyme is not show in panels A and B but is highly similar to the T. thermophilus enzyme, and the relevant residues in σ and β´are identical. The alignment was produced with ClustalW (www.ebi.ac.uk) and the accession numbers given at the end of the Material and Methods section. (TIF) [file pgen.1005104.s006.tif]

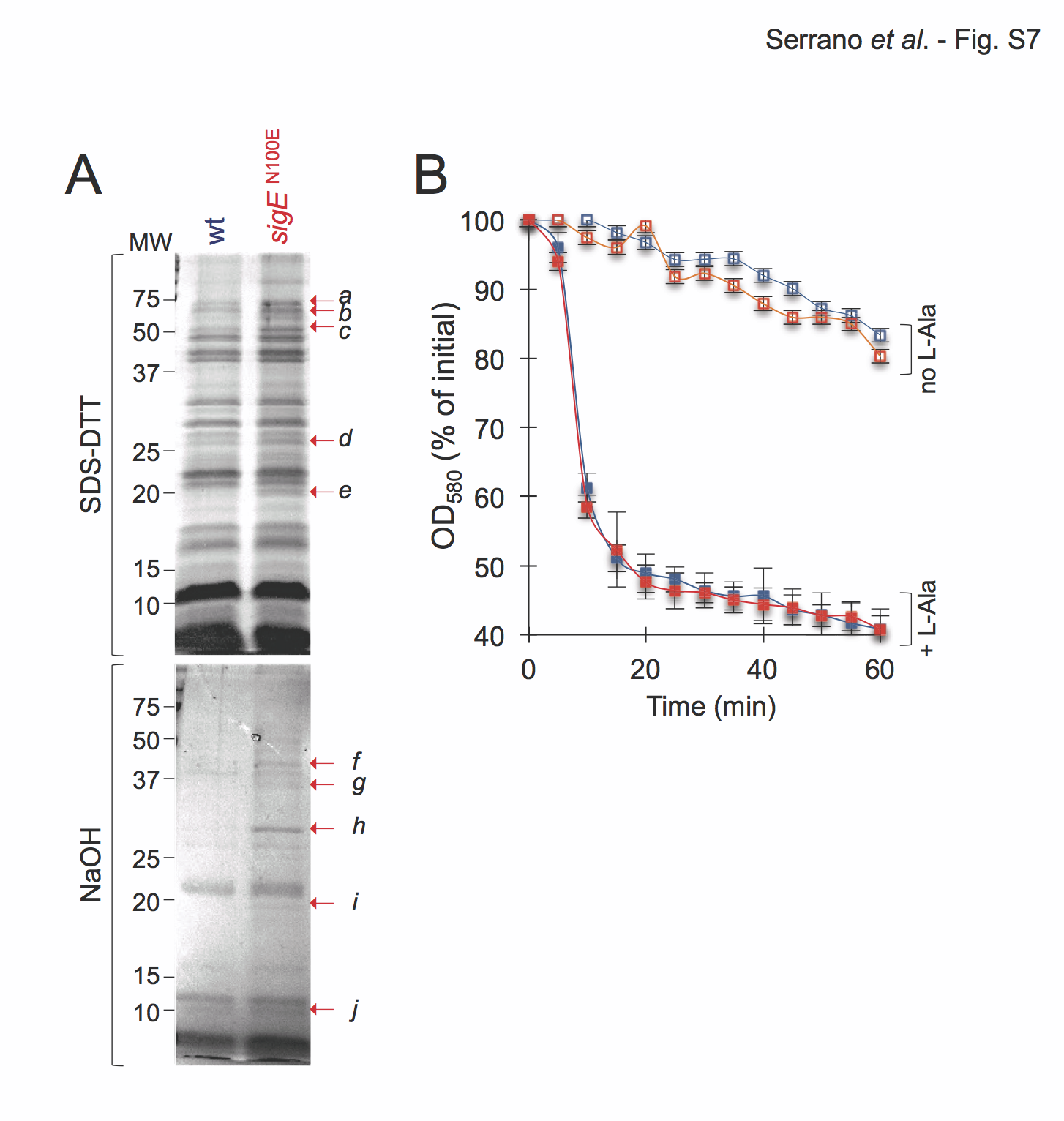

Supplement: S7 Fig — A: spores of the indicated strains were purified on density gradients and proteins extracted by treatment with an SDS/DTT-containing buffer (top) or NaOH (bottom). The red arrows indicate the position of proteins that are more extractable from spores produced by the strain producing the N100E form of σE (see also S1 Text). B: spores of the indicated strains, identified by the color code used in panel A, were induced to germinate by exposure to L-Alanine (10 mM; close symbols). Spores in control samples (open symbols) were heat activated but not exposed to L-Alanine. The rate and extent of germination monitored by following the drop in the optical density of the suspension (OD) at 580 nm, over time, and expressed as the percentage of the initial OD580. (TIF) [file pgen.1005104.s007.tif]
