# Supplementary material for: Dual-Specificity Anti-sigma Factor Reinforces Control of Cell-Type Specific Gene Expression in Bacillus subtilis
Source: PLoS Genet. 2015 Apr 2;11(4):e1005104. doi: 10.1371/journal.pgen.1005104 (PMC4383634; doi:10.1371/journal.pgen.1005104)
Supplement: S1 Text — (DOCX) [file pgen.1005104.s008.docx]

**Supplemental Material**

**Supplemental Materials and Methods**

**P*_csfB_-lacZ* fusions.** To construct a P*_csfB_-lacZ* transcriptional fusion to be integrated at the *amyE* locus the upstream region was amplified from chromosomal DNA of a wild type *B. subtilis* strain using the primer csfB12D and csfB578R (all primers used are listed in S2 Table). The resulting 266 bp *csfB* fragment was cloned between the *Sma*I and *Bam*HI sites of pSN32 [70], to yield pMS384.

**Mutagenesis of the *csfB* promotor.** First a copy of the *csfB* gene including 150 bp upstream of the start codon was PCR-amplified with primers csfB12D and csfB780R. The resulting 468 pb product was digested with *Hind*III and *Bam*HI and inserted between the same sites of the *amyE* integrational vector pDG364 [43], to yield pMS413. We then used primers PsigFcsfBD and PsigFcsfBR to introduce point mutations in the -10 region of the σ^F^ promoter, and primers PsigKcsfBD and PsigKcsfBR to introduce point mutations into the -10 region of the σ^K^ promoter (S2 Table), using pMS413 as the template. Plasmid pMS418 (mutations in the σ^F^promoter) and pMS425 (mutations in the σ^K^ promoter) were the result. The same point mutations were also introduced by PCR into pMS261, which carries a *csfB-gfp* translational fusion [20], and into pMS384 (carrying a P*_csfB_*-*lacZ* fusion; above), to give pMS419/pJB6 (mutations in the σ^F^ promoter) and pMS426/pJB8 (mutations in the σ^K^ promoter).

A fusion of *csfB-gfp* to the *spoIIQ* promoter was constructed as follows. Initially, the *csfB* gene fused to *gfp* and the *spoIIQ* promoter, were amplified separately from chromosomal DNA of the AH6507 strain. The following primers were used (S2 Table): for *csfB*, *csfB-spoIIQ*, and *gfpR*; for *spoIIQ*, primers *spoIIQ*-152D and *spoIIQ*-500R. The 911-bp c*sfB* fragment was mixed with the 350-bp *spoIIQ* fragment and the resulting fragments of 1261 bp were amplified using primers *spoIIQ*-152D and *gfpR*. The P*spoIIQ*-*csfB-gfp* fragment was digested with HindIII and BamHI and cloned between the same sites of pDG364 to yield pMS269.

**Mutagenesis of the *sigE* gene.** To create an in-frame deletion of the *sigE* gene, a 668 pb DNA fragment was first amplified with primers sigE288D and sigE956R from chromosomal DNA of a wild type *B. subtilis* strain (S2 Table). The PCR product was digested with *Bam*HI and *Eco*RI and inserted between the same sites of pGEX-4T-2 (GE Healthcare) yielding pMS427. pMS427 was cleaved with *Hind*III and autoligated, to produce pMS468. Sequencing confirmed the in-frame deletion of codons 109 to 175 of *sigE*. Competent cells of strain PY79 were cotransformed with pMS468 and chromosomal DNA from strain AH6931 (Δ*amyE*::*neo*), with selection for neomycin resistance. Spo^−^ congressants appeared at a frequency of about 3%. One, shown by PCR to carry a deletion of the *sigE* gene (referred to as Δ*sigE*) was named AH6933 (Table S1). We used pMS427, containing the *sigE* gene (above) and *sigE*-specific primers to convert residue the asparagine codon 100 into a glutamate codon (N100E), present at the homologous position of σ^K^. The mutation was then transferred to the *sigE* locus by congression. One Spo^+^ congressant was chosen and the presence of the desired mutation was confirmed by PCR and sequencing.

**Mutagenesis of the *sigK* gene.** We first constructed a *spoIVCB* allele associated to a spectinomicin resistance determinant. First, a 606 bp fragment of *spoIVCA* was amplified with primers spoIVCA1914D and spoIVCA2520R from chromosomal DNA of a wild type *B. subtilis* strain (S2 Table). The PCR product was digested with *Bam*HI and *Hind*III and inserted between the same sites of pAH256 [71], yielding pMS475. Next, a 522 bp fragment of *spoIVCB* was amplified with primers spoIVCB2488D and spoIVCB3010R (S2 Table). The PCR product was digested with *EcoR*I and *Bgl*II and inserted between the same sites of pMS475 to give pMS477. We used pMS477, containing the *spoIVCB* gene (see above) and *sigK*-specific primers to convert residue to replace the glutamate codon 73 to an asparagine codon (found at the homologous position of σ^E^), to produce pMS479. The mutation was transferred to the *spoIVCB* gene by congression. A Spo^+^ congressant was identified and the presence of the mutation verified by PCR and sequencing.

**Yeast two-hybrid analysis.** The coding regions of *sigA*, *sigE* and *sigK* were amplified with primers sigA193D and sigA1343R; sigE278D and sigE956R; and sigK249D, sigKR, sigKD and sigK655R (S2 Table). We used primers sigEN100ED and sigEN100ER and sigKE93ND and sigKE93NR (S2 Table), to convert codon 100 of *sigE* into an aspartate codon and codon 93 of *sigK* into a asparagine codon by PCR (Quickchange, Stratagene). Primers sigE278D or sigEY2H3.1D or sigEY2H4D or sigEY2H292D or sigEY2H2.2D and sigE956R or sigE495R or sigE3.1R or sigE421R or sigE2.3R were used to PCR amplify different segments of the coding region of *sigE* (S2 Table). The PCR products were digested with *Nco*I and *Eco*RI and inserted between the same sites of pAS2-1 (Clontech) yielding plasmids pJB18, pJB10 and pJB11, respectively. Mating of *Sacharomyces cerevisiae* strains and detection of β-galactosidase activity were performed as described before [72].

**CsfB-His_6_ pull-down experiments with purified σ^E^ and σ^K^.** His_6_-tagged sigma factors were purified on a 1 ml HisTrap FF column (GE Healthcare) using an AKTAexplorer 10 (GE Healthcare). These His_6_-tagged proteins were dialyzed at 4°C in 50 mM Tris, 100 mM NaCl, 2 mM β-mercaptoethanol, pH 7.5, and then incubated with 100 units of thrombin protease (GE Healthcare) at room temperature for 2 hours to remove the His_6_ tags. The untagged proteins were separated from the His_6_-tags by chromatography through a 1 ml HiTrap Benzamidine FF and 1 ml HisTrap columns (GE Healthcare). To examine their interaction with CsfB these untagged sigma factors were chromatographed through 1ml HisTrap columns alone or after incubation with His_6_-tagged CsfB, as indicated (Fig 5).

**GBP Pull-down experiments.** *B. subtilis* strains carrying a *csfB-gfp* translational fusion were grown in DSM to 6 hours after the onset of sporulation, before the cells were harvested. The cell pellets were resuspended in 1-ml portions of buffer A [100 mM NaCl, 10 mM Tris-HCl (pH 8.0), 10% glycerol] per 100 ml of culture and lysed in a French pressure cell (18,000 lb/in^2^). The lysate was cleared by centrifugation. One milliliter of cleared lysate was bound to 50 µl of a 50% slurry of GFP-Trap beads (ChromoTek) at room temperature for 30 min. The beads were washed three times in buffer B (same as A but with 200 mM NaCl) and resuspended in a final volume of 100 μl. The samples were subjected to SDS-PAGE and immunoblotting.

**GST Pull-Down experiments.** Primers sigE288D or sigE3.1D or sigE4D or sigE292D or sigE2.2D and sigE956R or sigE495R or sigE3.1R or sigE421R or sigE2.3R were used to PCR amplify the different segments of the coding region of *sigE* from chromosomal DNA of a wild type *B. subtilis* strain (S2 Table). We used primers sigEN100ED and sigEN100ER (S2 Table), to convert the asparagine codon 100 of *sigE* into a glutamate codon by PCR (Quickchange; Stratagene). The PCR products were digested with *Bam*HI and *Eco*RI and cloned between the *Bam*HI and *Eco*RI sites of pGex4T-2 (GE Healthcare) to create plasmids which bear in-frame N-terminal GST fusions to the different forms of σ^E^. Derivatives of BL21 (DE3) bearing each of these plasmids or pGex4T-3 (GST-alone) were grown to mid-log phase (O.D._600_≈0.6) in LB, and induced with 1 mM IPTG for 3 h before the cells were harvested. The cell pellets were resuspended in 1-ml portions of buffer A [100 mM NaCl, 10 mM Tris-HCl (pH 8.0), 10% glycerol] per 50 ml of induced culture and lysed in a French pressure cell (18,000 lb/in^2^). The lysate was cleared by centrifugation. One milliliter of cleared lysate was bound to 50 μl of a 50% slurry of glutathione Sepharose beads (GEHealthcare) at room temperature for 30 min. The beads were washed three times in buffer B (same as A but with 200 mM NaCl). Next, 100 nM of purified CsfB-*Strep* II tag was incubated for 30 min at room temperature with the glutathione Sepharose beads complexed with the GST fusion proteins or with glutathione Sepharose beads alone. The mixtures were washed three times with buffer B (above) and resuspended in a final volume of 30 μl. The samples were subjected to SDS-PAGE and immunoblotting (below). An anti-*Strep* II tag polyclonal antibody was used at a 1:1000 dilution (IBA GmbH).

**Immunoblot analysis.** σ^A^, σ^E^, σ^G^, and σ^K^ were immunodetected using rabbit polyclonal antibodies of established specificity, as previously described [20,73].

**Spore purification and extraction and analysis of spore coat proteins.** Spores

were purified from 24-hour cultures grown on resuspension medium using metrizoic acid gradients as described previously [43]. SDS/DTT or NaOH extraction extraction and analysis of the spore coat proteins were performed as described before [43].

**Spore resistance and germination assays**. Spores were purified on metrizoic acid gradients as outlined above and assayed for heat and lysozyme resistance as described before. The efficiency of sporulation is defined as the ratio between the heat resistant and the total cell count in colony forming units per ml of culture (CFU/ml) x 100 [43]. For germination assays, purified spores were heat activated by incubation for 10 min at 80ºC, and diluted in 10 mM Tris-HCl (pH 8.0) buffer to an OD_580_ of 1. After 15 min at 37°C, germination was induced by addition of 10 mM L-alanine. Germination was monitored at 5-min intervals, by monitoring the decrease in the OD_580_ of the suspension, until a constant reading was reached [43].

β**-Galactosidadse assays.** β-Galactosidadse enzyme activity was assayed with the substrate *o*-nitro-β-D-galactopyranoside (ONPG), and expressed in Miller units as described before [20].

**Supplemental Results and Discussion**

**Mutations in the σ^F^-dependent promoter of *csfB* lead to the accumulation of pre-divisional sporangia with levels of σ^G^ activity**

In an earlier study, we have shown that CsfB is part of a composite negative feedback loop limiting σ^G^ activity in pre-divisional sporangia. The composite negative feedback loop involves the σ^G^-dependent transcription of *csfB*, and the inhibition of σ^G^ activity by CsfB, along with the SpoIIAB anti-sigma factor and the LonA protease [20,31,32] (Fig 1C). Deletion of *csfB* thus results in a sub-population of cells with high levels of σ^G^ activity [20]. Transcription of *csfB* by σ^G^-containing RNA polymerase in pre-divisional sporangia relies on the same promoter that is recognized by σ^F^ in the forespore, prior to engulfment completion, as we now show (Fig 2A and D). In this work, we also show that *csfB* has a second promoter, utilized by the σ^K^-containing RNA polymerase holoenzyme, which is activated in the mother cell following engulfment completion (Fig 2). One prediction then was that point mutations in the -10 region of the σ^F^ promoter, that we show eliminate *csfB* transcripton in the forespore (Fig 2A and C), would also results in the accumulation of pre-divisional sporangia with high levels of σ^G^ activity. In contrast, mutations in the -10 region of the σ^K^*-*dependent promoter, which we show in this work to abolish expression of *csfB-gfp* specifically in the mother cell (Fig 2A and C), should not lead to pre-divisional sporangia with high levels of σ^G^ activity. To test this, a copy of the *csfB* gene under the control of its normal regulatory region (P*_csfB_*-*csfB*), under the control of the σ^F^-dependent promoter (P*_sigF_*-*csfB*) or under the control of the σ^K^-dependent promoter (P*_sigK_*-*csfB*) were transferred to the *amyE* locus of a strains bearing a *csfB* deletion and a reporter for σ^G^ activity, either P_sspE_-*lacZ*, inserted at the *sspE* locus, or P*_sspE_*-*cfp*, inserted at the non-essential *yycR* locus (S1 Table). Cells were induced to sporulate in DSM medium, as in our original study showing the effect of CsfB in the control of σ^G^ activity in pre-divisional sporangia, and samples taken to measure β-galactosidase activity and CFP accumulation. Deletion of *csfB* caused increased expression of *sspE-lacZ* prior to hour 3 of sporulation in agreement with earlier results [20] (S3 Fig). Expression of P*_sigK_*-*csfB*, but not of P*_sigF_*-*csfB* or P*_csfB_*-*csfB*, also caused increased expression of *sspE-lacZ* before hour 3 of sporulation in DSM (S3A Fig). The increased expression of the σ^G^ reporter in the *csfB* deletion mutant was shown previously to be due to small population of pre-divisional cells (about 2%), with high levels of σ^G^ activity [20]. We now found about 2% of the cells in the *csfB* mutant with high levels of CFP, in agreement with our previous study [20] (S3B Fig, yellow arrows). Moreover, these cells, as judged from staining with the FM4-64 membrane dye, did not show signs of asymmetric division (S3B Fig, yellow arrows). Expression of P*_sigK_*-*csfB* resulted in about 1.5% of pre-divisional cell with high levels of σ^G^ activity (S3B Fig). In contrast, only 0.3% of pre-divisional cells with high levels of CFP were found for the P*_sigF_*-*csfB*-expressing strain, close to the level (0.2%) observed for the strain expressing P*_csfB_*-*csfB* from the *amyE* locus (S3B Fig, yellow arrows).

Together, these results are in agreement with our finding that the σ^F^-dependent promoter of *csfB* is utilized by both by σ^F^ and σ^G^, and that this promoter is responsible for the synthesis of CsfB in pre-divisional sporangia under the control of σ^G^, and in the forespore soon after asymmetric division, under the control of σ^G^ ([20]; this work). Morever, the results also show that the *csfB* σ^K^-dependent promoter has no role in the control of σ^G^ activity in pre-divisional sporangia.

**SpoIIAB, LonA and CsfB contributions to the inhibition of σ^G^ in the mother cell**

As previously shown, when the *sigG* gene is placed under the control of a σ^E^-controlled promoter, SpoIIAB and LonA are also important for the inhibition of σ^G^ activity in the mother cell [15,20,32]. Thus, to compare the respective contributions of CsfB, SpoIIAB and LonA in the inhibition of σ^G^ activity in the mother cell, we expressed *sigG* from the σ^E^-controlled P*_spoIID_* promoter [40] and examined the effect of a *csfB* deletion, alone or in combination with a *lonA* deletion, and with or without a mutation (E156K) that makes σ^G^ refractory to the action of SpoIIAB [15]. An *sspE-lacZ* fusion was used as a reporter for σ^G^ activity [15,20] in a strain carrying the P*_spoIID_*-*sigG wt* allele at the non-essential *amyE* locus (as well as an in-frame deletion of the wild type *sigG* gene) (S5A Fig). In agreement with previous results, expression of the wild type *sigG* gene in a *lonA* background or of *sigG E156K* in a *lonA*^+^ strain, resulted in higher levels of *sspE-lacZ* expression, an effect that was further increased when the two genetic perturbations were combined (in a *sigG E156K*/*lonA* strain) (S5A Fig). In a P*_spoIID_*-*sigG wt* strain, deletion of *csfB* did not increase *sspE-lacZ* expression above background levels (S5A Fig). However, in a *lonA csfB* double mutant *sspE-lacZ* expression increased to the level of the P*_spoIID_*-*sigG E156K* strain, while the P*_spoIID_*-*sigG E156K*/*csfB* combination increased *sspE-lacZ* expression to the level of the *sigG E156K*/*lonA* strain (S5A Fig). A triple mutant (P*_spoIID_*-*sigG E156K*/*csfB/lonA*) exhibited only a small additional increase in *sspE-lacZ* expression over the P*_spoIID_*-*sigG E156K*/*csfB* strain (S5A Fig). Thus, in the mother cell, SpoIIAB plays the leading role in σ^G^ inhibition, while LonA and CsfB appear to have largely redundant supportive contributions (S5C Fig). This contrasts with the situation observed in pre-divisional cells, where CsfB is the major contributor to σ^G^ inhibition ([20]; see also above). Nevertheless, these results demonstrate the ability of CsfB to act as an inhibitor of σ^G^ activity in the mother cell (S5C Fig).

**σ^G^ can activate σ^K^ from the mother cell**

Activation of σ^G^ in the mother cell has important consequences on the temporal control of σ^K^ activity. Expression of *gerE-lacZ*, a reporter for σ^K^ activity, was monitored throughout sporulation in a wild type strain (S5B Fig, P*_sigG_*-*sigG wt*) and in strains deleted for *sigG* at its native locus, but expressing either P*_spoIID_*-*sigG wt* or P*_spoIID_*-*sigG E156K*. In the wild type strain, expression of *gerE-lacZ* commenced at around hour 4 of sporulation (S5B Fig). Activation of σ^K^ requires pro-σ^K^ processing, which in turn depends on the activity of σ^G^ in the forespore [25,33]. The results presented in figure S5B show that expression of σ^G E156K^ triggers pro-σ^K^ processing, resulting in premature activation of σ^K^ in the mother cell. Possibly then, the augmented transcription of σ^K^-controlled genes in the P*_sigF_* strain as noted by transcriptional profiling (see above) results in part from premature processing of pro-σ^K^ (S5C Fig), again underscoring the need for strict inhibition of σ^G^ activity in the mother cell.

**The N100E substitution in σ^E^ affects the assembly of the inner and outer spore coat**

Spores produced by the wild type and the σ^E N100E^ strain were purified to over 99% purity on density gradients and the spore coat proteins extracted by either boiling in a buffer containing SDS and DTT, or by a NaOH treatment [42]. During both treatments, the spores remain phase bright [42]. The extracted proteins were resolved by SDS-PAGE (on 15% gels), and bands showing increased extractability in the mutant relative to the wild type spores (*a*-*e* for the SDS/DTT treatment, and *f-k* for the NaOH treatment; S7A Fig) were excised and the proteins identified by mass spectrometry (at the Emory Proteomics Core). Following SDS/DTT treatment of spores of the σ^E N100E^ strain the most abundant species found in bands *a* to *e* were (the predicted molecular masses are given) (S7A Fig, top): CotA (58 kDa, in *a*), CotB (in *b*, which normally migrates just under CotA), the YaaH (48.5 kDa) and YpeB proteins (51 kDa) (in *c*), the YybI protein (29.9 kDa), CotE (24 kDa) and Tgl (28 kDa, in *d*), and fragments of YaaH and YpeB (in *e*). When the spore coat proteins were extracted using NaOH, the most abundant proteins found in bands *f* through *k* were (S7A Fig, bottom): in band *f*, YhfE (a putative glycsyltransferase of 38.6 kDa) and the alkaline phosphatase PhoB (50.3 kDa); in band *g*, a 35 kDa fragment of the subtilisin-like protease Vpr (predicted mass of 85.4 kDa) and the reticulline oxidase-like protein YvdP (of 49.9 kDa; possibly a fragment); in band *h*, possibly a multimeric form of CotN (predicted mass of 9.6 kDa); in band *i*, CotE (at 20 kDa); in band *j*, CotN (possibly the monomeric form).

While CotA, CotB, YvdP and Tgl are outer coat proteins, CotE is at the inner coat/outer coat interface and CotN, YaaH and YybI are associated with the spore inner coat [41,44,45,46,74]. CotA, Tgl, YaaH and YpeB are enzymes. CotA is a laccase, whereas Tgl is a transglutaminase that cross-links several spore coat proteins, and both YaaH and YpeB are involved in degradation of the spore cortex peptidoglycan during germination [41,45,75]. The increased extractability of YaaH and YpeB does not however seem to affect the germination of spores of the σ^E N100E^ strain (Fig S7B).

Production of CotE, YaaH, YhfE, PhoB and YybI is under σ^E^ control, whereas production of CotA, CotB, Tgl, YvdP, CotN and CotU is mainly controlled by σ^K^ [9,29,44,45,46]. Both Vpr, which may be produced only in pre-divisional sporangia, and PhoB are abundant in the extracellular proteome [76,77]. It is not presently known whether Vpr and PhoB accumulate in the extracellular medium and associate with the spore following its release. If so, the apparent increased extractability of Vpr and PhoB could reflect their increased association with the released spores, with the implication that spores of the σ^E N100E^ strain may have an altered surface. YpeB is produced inside the developing spore, under the control of σ^G^ [8,29,75]. We do not presently know why the YpeB protein shows increased extractability from spores of the σ^E N100E^ strain.

**Supplemental References**

70. Mota LJ, Tavares P, Sa-Nogueira I (1999) Mode of action of AraR, the key regulator of L-arabinose metabolism in Bacillus subtilis. Mol Microbiol 33: 476-489.

71. Henriques AO, Melsen LR, Moran CP, Jr. (1998) Involvement of superoxide dismutase in spore coat assembly in Bacillus subtilis. J Bacteriol 180: 2285-2291.

72. Zilhao R, Serrano M, Isticato R, Ricca E, Moran CP, Jr., et al. (2004) Interactions among CotB, CotG, and CotH during assembly of the Bacillus subtilis spore coat. J Bacteriol 186: 1110-1119.

73. Fujita M, Losick R (2005) Evidence that entry into sporulation in Bacillus subtilis is governed by a gradual increase in the level and activity of the master regulator Spo0A. Genes Dev 19: 2236-2244.

74. Abhyankar W, Beek AT, Dekker H, Kort R, Brul S, et al. (2011) Gel-free proteomic identification of the Bacillus subtilis insoluble spore coat protein fraction. Proteomics 11: 4541-4550.

75. Boland FM, Atrih A, Chirakkal H, Foster SJ, Moir A (2000) Complete spore-cortex hydrolysis during germination of Bacillus subtilis 168 requires SleB and YpeB. Microbiology 146 ( Pt 1): 57-64.

76. Lanigan-Gerdes S, Dooley AN, Faull KF, Lazazzera BA (2007) Identification of subtilisin, Epr and Vpr as enzymes that produce CSF, an extracellular signalling peptide of Bacillus subtilis. Mol Microbiol 65: 1321-1333.

77. Tjalsma H, Antelmann H, Jongbloed JD, Braun PG, Darmon E, et al. (2004) Proteomics of protein secretion by Bacillus subtilis: separating the "secrets" of the secretome. Microbiol Mol Biol Rev 68: 207-233.
