# Supplementary material for: Dual-Specificity Anti-sigma Factor Reinforces Control of Cell-Type Specific Gene Expression in Bacillus subtilis
Source: PLoS Genet. 2015 Apr 2;11(4):e1005104. doi: 10.1371/journal.pgen.1005104 (PMC4383634; doi:10.1371/journal.pgen.1005104)
Supplement: S1 Table — (DOCX) [file pgen.1005104.s009.docx]

**Table S1 *Bacillus subtilis* strains used in this work.**

| **Strain** | Relevant Properties | Origin/Reference |
| --- | --- | --- |
| MB24 | *trpC2 metC3* | Laboratory stock |
| MH5636 | *trpC2 pheA1 rpoC::His_10_/*Cm^r^ | BGCS* |
| PY79 | Prototrophic | Laboratory stock |
| AH77 | *trpC2 metC3* Δ*sigK::erm* | “ |
| AH1042 | *trpC2 metC3* Δ*sspE::*P*_sspE_-lacZ* | “ |
| AH3795 | *trpC2* Δ*sigG* | “ |
| AH6507 | *trpC2 metC3* Δ*csfB::km* Δ*sspE::*P*_sspE_-lacZ* Δ*amyE::P_csfB_-csfB-gfp* | This work |
| AH6521 | *trpC2 metC3* Δ*csfB::km* Δ*sspE::*P*_sspE_-lacZ* Δ*amyE::P_spoIIQ_-csfB-gfp* | “ |
| AH6524 | *trpC2 metC3* Δ*csfB::km* Δ*sigK::erm* Δ*amyE::P_csfB_-csfB-gfp* | “ |
| AH6598 | *trpC2* Δ*sigG* Δ*sspE::sspE-lacZ* Δ*amyE::P_spoIID_-sigG wt* | [15] |
| AH6599 | *trpC2* Δ*sigG* Δ*sspE::sspE-lacZ* Δ*amyE::P_spoIID_-sigG E156K* | “ |
| AH6600 | *trpC2* Δ*sigG* Δ*sspE::sspE-lacZ* Δ*lonA::cat* Δ*amyE::P_spoIID_-sigG wt* | “ |
| AH6601 | *trpC2* Δ*sigG* Δ*sspE::sspE-lacZ* Δ*lonA::cat* Δ*amyE::P_spoIID_-sigG E156K* | “ |
| AH6602 | *trpC2* Δ*sigG* Δ*sspE::sspE-lacZ* Δ*lonA::cat* Δ*amyE::P_spoIID_-sigG wt* Δ*csfB::km* | This work |
| AH6603 | *trpC2* Δ*sigG* Δ*sspE::sspE-lacZ* Δ*lonA::cat* Δ*amyE::P_spoIID_-sigG E156K* Δ*csfB::km* | “ |
| AH6604 | *trpC2* Δ*sigG* Δ*sspE::sspE-lacZ* Δ*amyE::P_spoIID_-sigG wt* Δ*csfB::km* | “ |
| AH6605 | *trpC2* Δ*sigG* Δ*sspE::sspE-lacZ* Δ*amyE::P_spoIID_-sigG E156K* Δ*csfB::km* | “ |
| AH6728 | *trpC2* Δ*csfB::km* Δ*amyE::P_sigK_-csfB-gfp* | “ |
| AH6741 | *cotG-lacZΩcotG* | Laboratory stock |
| AH6769 | Δ*csfB::tet* Δ*sspE::*P*_sspE_-lacZ* | This work |
| AH6770 | Δ*csfB::tet* Δ*amyE::P_csfB_-csfB* Δ*sspE::*P*_sspE_-lacZ* | “ |
| AH6771 | Δ*csfB::tet* Δ*amyE::P_sigK_-csfB* Δ*sspE::*P*_sspE_-lacZ* | “ |
| AH6772 | Δ*csfB::tet* Δ*amyE::P_sigF_-csfB* Δ*sspE::*P*_sspE_-lacZ* | “ |
| AH6792 | *trpC2* Δ*csfB::km* Δ*amyE::P_sigF_-csfB-gfp* | This work |
| AH6804 | *spoIVCA-lacZΩspoIVCA* | Laboratory stock |
| AH6818 | Δ*csfB::tet* | [19] |
| AH6825 | Δ*csfB::tet* Δ*amyE::P_csfB_-csfB* | This work |
| AH6826 | Δ*csfB::tet* Δ*amyE::P_sigK_-csfB* | “ |
| AH6827 | Δ*csfB::tet* Δ*amyE::P_sigF_-csfB* | “ |
| AH6886 | Δ*csfB::tet* Δ*yycR::*P*_sspE_-cfp* | “ |
| AH6889 | Δ*csfB::tet* Δ*amyE::P_csfB_-csfB* Δ*yycR::*P*_sspE_-cfp* | “ |
| AH6890 | Δ*csfB::tet* Δ*amyE::P_sigK_-csfB* Δ*yycR::*P*_sspE_-cfp* | “ |
| AH6891 | Δ*csfB::tet* Δ*amyE::P_sigF_-csfB* Δ*yycR::*P*_sspE_-cfp* | “ |
| AH6931 | Δ*amyE::neo* | “ |
| AH6932 | *spoIVCA-lacZΩspoIVCA* | “ |
| AH6933 | Δ*sigE* | “ |
| AH6934 | Δ*sigE*  Δ*amyE::neo spoIVCA-lacZΩspoIVCA* | “ |
| AH6936 | *sigE N100E*  Δ*amyE::neo spoIVCA-lacZΩspoIVCA* | “ |
| AH6940 | Δ*sigE*  Δ*amyE::neo cotG-lacZΩcotG* | “ |
| AH6943 | *sigE N100E*  Δ*amyE::neo cotG-lacZΩcotG* | “ |
| AH6952 | *spoIVCA-Sp-spoIVCB* | “ |
| AH6953 | *spoIVCA-Sp-spoIVCB E73N* | “ |
| AH6954 | *spoIVCA-Sp-spoIVCB spoIVCA-lacZΩspoIVCA* | “ |
| AH6955 | *spoIVCA-Sp-spoIVCB cotG-lacZΩcotG* | “ |
| AH6956 | *spoIVCA-Sp-spoIVCB E73N spoIVCA-lacZΩspoIVCA* | “ |
| AH6957 | *spoIVCA-Sp-spoIVCB E73N cotG-lacZΩcotG* | “ |
| AH9284 | *trpC2 metC3* Δ*amyE::P_xylA_-gfp* | “ |
| AH9538 | *trpC2 metC3* Δ*amyE*::P*_csfB_-lacZ* | “ |
| AH9551 | *trpC2 metC3* Δ*amyE*::P*_sigF_-lacZ* | “ |
| AH9577 | *trpC2 metC3* Δ*amyE*::P*_sigK_-lacZ* | “ |
| AH9591 | *trpC2 metC3* Δ*sigK::erm* Δ*amyE*::P*_sigK_-lacZ* | “ |
| AH9593 | *trpC2 metC3* Δ*sigG* Δ*amyE*::P*_sigF_-lacZ* | “ |
| MO3632 | Δ*csfB::km* | [22] |

**Bacillus* Genetic Stock Center.
