# Supplementary material for: Dual-Specificity Anti-sigma Factor Reinforces Control of Cell-Type Specific Gene Expression in Bacillus subtilis
Source: PLoS Genet. 2015 Apr 2;11(4):e1005104. doi: 10.1371/journal.pgen.1005104 (PMC4383634; doi:10.1371/journal.pgen.1005104)
Supplement: S2 Table — (DOCX) [file pgen.1005104.s010.docx]

**Table S2 Oligonucleotides used in this work.**

| **Primer** | Sequence (5’ 🡪 3’) |
| --- | --- |
| csfB12D | CGCCGCCAAGCAAGCTTAAACCCAGC |
| csfB578R | CGTATAAAGATGGATCCTCTATTCTTCTC |
| csfB780R | ATTATTAGGATCCACTACGTTCAATCC |
| csfBspoIIQ | GTTGCTGAGGTGATGAAACAATGGACGAAACAGTTAAACTTAATC |
| gfpR | GGCGGATCCTTATTTGTATAGTTCATCCATGC |
| PsigFcsfBD | CCGTTTTAAAGGGCGGCCAACTGTACGGAGG |
| PsigFcsfBR | CCTCCGTACAGTTGGCCGCCCTTTAAAACGG |
| PsigKcsfBD | GGGCTTATATAGCCCCGCTACCGATTTAAAGTTTCAAAAAGG |
| PsigKcsfBR | CCTTTTTGAAACTTTAAATCGGTAGCGGGGCTATATAAGCCC |
| sigA193D | GATCCATGGCTGATAAACAAACCC |
| sigA1343R | GAACGGAATTCAAGACCCGTTCC |
| sigE278D | CTATTCCATGGGCGGGAGTGAAGCCCTGCC |
| sigE288D  sigE292D  sigEY2H292D | CGCGGATCCGGCGGGAGTGAAGCCCTGCC  GAAGGATCCGCTGTTAATACATTTAATCCAG  CTTTCCATGGCTGTTAATACATTTAATCCAG |
| sigE421R  sigE495R | ATCGAATTCTTACGGTTCATAAAAGGAAACC  CGCGAATTCTTAAGCTTTGATTAGACCGATGGTACCG |
| sigE956R  sigEY2H2.2D  sigE2.2D  sigE2.3R  sigE4D  sigEY2H4D  sigE3.1D  sigEY2H3.1D  sigE3.1R  sigEN100ED  sigEN100ER | CCCTGTGAATTCAAGGGGTTCTAACC  CTTTCCATGGAAAATACGGGAATTAATATAGAGG  GAAGGATCCGAAAATACGGGAATTAATATAGAGG  ATCGAATTCTTATATACACCGGGAGGCATAGG  GAAGGATCCGTCGATAAAAAGCTTTTG  TAACCCATGGTCGATAAAAAGCTTTTG  TTTGGATCCGAACCGCTTAATATTGATTGGG  CTTTCCATGGAACCGCTTAATATTGATTGGG  GACGAATTCTTAGTTAGCTTCTATGTCTTTAG  CTAATCAAAGCTGTTGAAACATTTAATCCAG  CTGGATTAAATGTTTCAACAGCTTTGATTAG |
| sigK249D | GTCTCCATGGTATCTTACGTGAAAAACAATGCC |
| sigKR | CTCATTTTCAATACACCTCGC |
| sigKD | GCGAGGTGTATTGAAAATGAGATCCTCATGCATTTGCGCGC |
| sigK655R  sigKE93ND  sigKE93NR | TAGAGAATTCCGGAGTATGAGATCCGGC  CTGATCAAAGGAATTAATAGCTATTCCGCCGG  CCGGCGGAATAGCTATTAATTCCTTTGATCAG |
| spoIIQ152D | GTTTCAAAGCTTGATCCAGGCAGCG |
| spoIIQ500R | TGTTTCATCACCTCAGCAAC |
| spoIVCA1914D | CGGAAAAGCTTAAATACCATGACAGGG |
| spoIVCA2520R | GGGTGCATGGATCCCTCTTTAATACG |
| spoIVCB2488D | ATTGAATTCGTATTAAAGAGGGG |
| spoIVCB3010R | TTGAAGATCTAGTATGCTGCTTACC |
| sigA-fwd-pET14b | ccgctcgagatggctgataaacaaacccacgaga |
| SigA-rev-pET14b | cgcggatccttattcaaggaaatctttcaaacgtttacttctgct |
| csfB-fwd invitro | ttaatgagctgggttttttgtttactc |
| csfB-rev invitro | ttcgtataaagatgaatgcctctattct |
| gcaD fwd | aggactgctgaaagggctgacataa |
| gcaD rev | gatttcattctcgttccttgtccagccgc |
| sspB fwd invitro | gactagcttagcctaaacggctaa |
| sspB-rev-invitro | aactgatgctttttcatatttatgactagcttagcct |
| spoIIQ-fwd invitro | ctgtgcaaactacatctaaaaaagtttttttggata |
| spoIIQ-rev-invitro | tttcttcctctctcattgtttcatcacc |
| spoIID-fwd-invitro | gcaaaatagcaaaaaagaatacgtaaatgacaaat |
| spoIID-rec-invitro | ttgtttcatattcagctgcctcctg |
| gerE-fwd-invitro | gcatctatgtgaaaacaagcgcagtc |
| gerE-rev-invitro | ctccttcaagtattgtaaccctccttgcta |
