# Supplementary material for: Dual-Specificity Anti-sigma Factor Reinforces Control of Cell-Type Specific Gene Expression in Bacillus subtilis
Source: PLoS Genet. 2015 Apr 2;11(4):e1005104. doi: 10.1371/journal.pgen.1005104 (PMC4383634; doi:10.1371/journal.pgen.1005104)
Supplement: S3 Table — (PDF) [file pgen.1005104.s011.pdf]

Table S3 - Transcriptional profiling of sporulating cells

(dark green)	**<0.4**
(light green)	**0.4-0.5**
(red)	**≥2.5**
(orange)	**2-2.5**

Green: up-regulated in the PsigF strain
Red: down-regulated in the PsigF strain

Strains (see Table S1):
AH6825: PcsfB-csfB (abbreviated to PcsfB)
AH6827: PsigF-csfB (abbreviated to PsigF)

		Replicate 1			Replicate 2			
Locus tag	Name	log-ratio	Fold (PcsfB/PsigF)	p-value	log-ratio	Fold (PcsfB/PsigF)	p-value	Fold (Median)
BSU_misc_RN	BSU_misc_RN	0,95510505	1,938720797	1,02E-05	-0,1518756	0,90007954	0,574706862	1,419400169
BSU_misc_RN	BSU_misc_RN	-1,20579809	0,433529452	2,78E-06	-0,20439521	0,867902442	0,524493777	0,650715947
BSU_misc_RN	BSU_misc_RN	2,344016701	5,077142318	8,86E-16	-2,31814091	0,200525705	3,17E-14	2,638834012
BSU_misc_RN	BSU_misc_RN	0,485970124	1,400527318	0,000195335	-0,65153759	0,636601476	0,007023152	1,018564397
BSU_misc_RN	BSU_misc_RN	-0,19567639	0,873163426	0,122483492	-0,82312289	0,565217137	0,003003931	0,719190282
BSU_misc_RN	BSU_misc_RN	1,821449768	3,53436189	9,30E-13	-0,29280336	0,816314305	0,094766051	2,175338098
BSU_misc_RN	BSU_misc_RN	-0,10611916	0,929083932	0,684194739	-0,2103263	0,86434172	0,577587432	0,896712826
BSU_misc_RN	BSU_misc_RN	1,105369025	2,151539049	4,63E-07	-0,22623967	0,854860152	0,314756152	1,5031996
BSU_misc_RN	BSU_misc_RN	1,449837404	2,731772618	8,22E-11	-0,51011743	0,702165283	0,037896942	1,716968951
BSU_misc_RN	BSU_misc_RN	1,7597493	3,386392738	8,27E-12	-0,70856141	0,611930026	0,002740554	1,999161382
BSU_misc_RN	BSU_misc_RN	1,674827343	3,192811417	5,31E-12	-1,52230312	0,348129718	1,34E-09	1,770470568
BSU_misc_RN	Scr	3,008143921	8,045287186	6,01E-18	-2,06474917	0,239027884	2,13E-12	4,142157535
BSU_misc_RN	BSU_misc_RN	1,168141687	2,247220495	2,46E-09	-0,818951	0,566853959	0,000604992	1,407037227
BSU_misc_RN	BSU_misc_RN	1,840373012	3,581026046	2,58E-12	0,028496567	1,019948683	0,473677333	2,300487364
BSU_misc_RN	BSU_misc_RN	1,677835911	3,19947659	3,85E-08	0,206437381	1,153835359	0,121201634	2,176655975
BSU_misc_RN	BSU_misc_RN	0,942876885	1,922357802	4,35E-05	-0,82061033	0,566202361	0,000229298	1,244280082

BSU_misc_RN	BSU_misc_RN	0,769758537	1,704984397	1,70E-06	-0,69639032	0,617114322	0,014107195	1,161049359
BSU_misc_RN	BSU_misc_RN	1,208092278	2,310319338	1,03E-06	-0,12372454	0,91781511	0,535105263	1,614067224
BSU_misc_RN	BSU_misc_RN	-1,65089632	0,318442254	0,101798709	0,353241732	1,277427782	0,240726541	0,797935018
BSU_misc_RN	BSU_misc_RN	-1,44473406	0,367359871	4,22E-06	0,2224789	1,166736602	0,000309115	0,767048236
BSU_misc_RN	BSU_misc_RN	-0,32470214	0,798463221	0,097289419	-0,26186808	0,834007302	0,612362003	0,816235262
BSU_misc_RN	BSU_misc_RN	0,057645684	1,040765961	0,053093596	0,429906217	1,347146002	0,000572421	1,193955982
BSU_misc_RN	BSU_misc_RN	0,623731185	1,540855085	0,000527707	-0,05042941	0,965648869	0,664536569	1,253251977
BSU_misc_RN	BSU_misc_RN	3,028743118	8,160984026	3,90E-19	-1,07467781	0,474777079	6,54E-06	4,317880553
BSU_misc_RN	BSU_misc_RN	-0,76329082	0,58915093	0,002434802	0,429997631	1,347231364	0,016534989	0,968191147
BSU_misc_RN	RnpB	0,869059842	1,826472259	8,07E-05	-1,13347393	0,455816821	2,70E-06	1,14114454
BSU_misc_RN	BSU_misc_RN	1,48173783	2,792849496	2,53E-10	-0,46703123	0,723451781	0,02912495	1,758150638
BSU_misc_RN	BSU_misc_RN	1,022012923	2,030750393	4,03E-07	-0,69225938	0,618883866	0,00327681	1,324817129
BSU_misc_RN	BSU_misc_RN	0,355466821	1,279399496	0,107613094	-0,48952753	0,712258316	0,038286059	0,995828906
BSU_misc_RN	BSU_misc_RN	0,33359816	1,260152347	0,071602739	-0,25763947	0,836455406	0,602925133	1,048303876
BSU_misc_RN	BSU_misc_RN	-0,72501305	0,604991574	0,002258341	-0,55610768	0,68013466	0,01363614	0,642563117
BSU_misc_RN	BSU_misc_RN	4,308545435	19,81533476	4,68E-21	-1,34164843	0,39456956	6,02E-07	10,10495216
BSU_misc_RN	BSU_misc_RN	0,633905161	1,551759696	0,000874157	-0,08296576	0,944114823	0,588347537	1,24793726
BSU_misc_RN	BSU_misc_RN	0,840157923	1,790246099	7,79E-05	-0,01465799	0,989891294	0,131857041	1,390068696
BSU_misc_RN	BSU_misc_RN	0,097303977	1,069772466	0,511053974	-0,53053405	0,692298415	0,024582326	0,88103544
BSU_misc_RN	BSU_misc_RN	0,467225532	1,382448304	0,030469849	-0,67621995	0,625802813	0,044602668	1,004125558
BSU_misc_RN	BSU_misc_RN	0,807130811	1,749728173	0,000100891	-0,72026927	0,606984142	0,00244511	1,178356157
BSU_misc_RN	BSU_misc_RN	0,484668064	1,399263885	0,008769115	-0,28376481	0,821444602	0,536272548	1,110354244
BSU_misc_RN	BSU_misc_RN	0,944929756	1,925095152	0,000205157	-0,42791622	0,74333466	0,076015958	1,334214906
BSU_misc_RN	BSU_misc_RN	1,49634883	2,821277997	1,00E-07	-0,413718	0,750686269	0,115972576	1,785982133
BSU_misc_RN	BSU_misc_RN	0,701612024	1,62632098	0,00090716	0,11108936	1,080043455	0,31389615	1,353182218
BSU_misc_RN	BSU_misc_RN	0,708023388	1,633564465	3,38E-06	-0,10365956	0,930669247	0,806299597	1,282116856
BSU_misc_RN	BSU_misc_RN	1,270087092	2,411761244	5,50E-09	-1,38593029	0,382642681	2,32E-08	1,397201962
BSU_misc_RN	BSU_misc_RN	-0,94060592	0,521014012	0,00025751	-0,51435269	0,700106988	0,018167262	0,6105605
**SpoVT depen**	BSU_misc_RN	-0,33809591	0,791084711	0,226481617	0,008635052	1,00600331	0,862489125	0,89854401

BSU_misc_RN	BSU_misc_RN	-0,48995489	0,712047363	0,000250909	-1,83403912	0,280478265	4,67E-11	0,496262814
BSU_misc_RN	SsrA	1,989503345	3,971002709	1,69E-12	-0,42453365	0,745079538	0,846747853	2,358041123
BSU_misc_RN	BSU_misc_RN	1,534598577	2,897078098	1,73E-05	-0,04082072	0,972101785	0,104472009	1,934589941
BSU_misc_RN	BSU_misc_RN	0,491541931	1,405946728	0,009649548	-0,07697344	0,948044422	0,762628113	1,176995575
BSU_misc_RN	BSU_misc_RN	-0,27392672	0,827065378	0,043247022	-1,2970384	0,40696076	5,60E-08	0,617013069
BSU_misc_RN	BSU_misc_RN	-0,29880284	0,812926687	0,270440107	-0,72119651	0,606594149	0,002185531	0,709760418
BSU_misc_RN	BSU_misc_RN	1,310088948	2,47956827	9,20E-09	-0,73167191	0,602205624	0,00144218	1,540886947
BSU_misc_RN	BSU_misc_RN	1,594103357	3,01906822	1,19E-10	-0,41213598	0,751509904	0,122156854	1,885289062
BSU_misc_RN	BSU_misc_RN	1,899247025	3,730184592	2,11E-12	-0,0553976	0,962329195	0,593482337	2,346256893
BSU_misc_RN	BSU_misc_RN	0,667620856	1,588451296	0,127577872	2,096888248	4,277856973	0,003510648	2,933154135
BSU_misc_RN	BSU_misc_RN	0,078496185	1,055916816	0,920871745	0,144481988	1,105333702	0,02059496	1,080625259
BSU_misc_RN	BSU_misc_RN	0,420179425	1,33809396	0,002267264	-1,05089578	0,482668381	2,06E-05	0,91038117
BSU_misc_RN	BSU_misc_RN	-0,00092421	0,999359594	0,411491869	0,115880183	1,083635966	0,550969803	1,04149778
BSU_rRNA_1	RrnO-16S	3,040336981	8,226831989	2,78E-19	-3,53298353	0,086390499	6,76E-18	4,156611244
BSU_rRNA_14	RrnI-23S	2,57785813	5,970526382	3,65E-17	-3,06859536	0,119195745	2,66E-15	3,044861063
BSU_rRNA_15	RrnI-5S	3,827971388	14,20149975	2,40E-20	-1,05129619	0,482534437	0,013969237	7,342017093
BSU_rRNA_18	RrnD-5S	4,518934866	22,92635132	1,39E-21	-1,06448845	0,478142168	0,014480599	11,70224675
BSU_rRNA_2	RrnO-23S	2,824865272	7,085478435	1,97E-17	-3,21238657	0,107888532	9,66E-16	3,596683483
BSU_rRNA_23	RrnI-16S	2,811391544	7,019613236	2,12E-17	-3,42355832	0,093197927	1,20E-16	3,556405582
BSU_rRNA_25	RrnG-16S	2,914831171	7,541393718	7,39E-18	-3,59239247	0,082905265	4,56E-17	3,812149491
BSU_rRNA_29	RrnH-23S	2,755198181	6,751453742	1,27E-17	-3,20357334	0,108549626	2,84E-16	3,430001684
BSU_rRNA_3	RrnO-5S	3,754671243	13,49797651	1,79E-20	-1,09076713	0,469511652	0,011832874	6,983744081
BSU_rRNA_4	RrnA-16S	2,907026473	7,500706405	1,39E-17	-3,42115256	0,093353468	4,07E-17	3,797029937
BSU_rRNA_5	RrnA-23S	2,861915551	7,269799376	8,73E-18	-3,12113017	0,114933386	1,39E-15	3,692366381
BSU_rRNA_8	RrnJ-5S	4,081366402	16,92831417	7,57E-21	-1,03717837	0,487279566	0,014352622	8,707796866
BSU_tRNA_1	TrnY-Phe	3,006021185	8,033458312	1,65E-18	-0,45630787	0,728849139	0,076467435	4,381153726
BSU_tRNA_10	TrnSL-Met1	3,272254273	9,661547413	3,09E-19	-1,37610956	0,385256297	2,05E-07	5,023401855
BSU_tRNA_12	TrnJ-Val	3,65269942	12,57685606	1,44E-19	0,816261574	1,760837254	1,47E-06	7,168846657
BSU_tRNA_13	TrnJ-Thr	3,950485741	15,46018567	1,08E-20	-1,47785424	0,359022399	9,06E-09	7,909604037

BSU_tRNA_14	TrnJ-Lys	2,08223455	4,234625979	2,11E-14	-0,08157265	0,945026931	0,766461028	2,589826455
BSU_tRNA_15	TrnJ-Leu1	4,142582769	17,6620729	2,49E-20	-1,43745814	0,36921725	1,28E-06	9,015645075
BSU_tRNA_16	TrnJ-Gly	2,987051598	7,928520067	5,08E-19	-0,98754694	0,504334583	3,36E-05	4,216427325
BSU_tRNA_17	TrnJ-Leu2	2,930205239	7,622188247	3,13E-18	-0,5790822	0,669389488	0,008578733	4,145788867
BSU_tRNA_18	TrnJ-Arg	2,831145998	7,116392077	1,82E-17	-0,62500343	0,648418237	0,004297021	3,882405157
BSU_tRNA_19	TrnJ-Pro	2,922687987	7,582575652	5,08E-18	-0,38043021	0,768208479	0,177098831	4,175392065
BSU_tRNA_21	TrnE-Met	4,473568918	22,21664277	1,91E-21	-1,51712521	0,349381418	1,66E-08	11,28301209
BSU_tRNA_22	TrnE-Asp	5,06748376	33,53239816	2,95E-22	-1,62626077	0,323926687	5,74E-10	16,92816242
BSU_tRNA_23	TrnI-Asn	2,895872739	7,442940687	6,48E-18	-0,63544858	0,643740623	0,008333633	4,043340655
BSU_tRNA_24	TrnI-Thr	3,761797998	13,56482	9,11E-20	-0,93841303	0,521806554	1,91E-05	7,043313276
BSU_tRNA_29	TrnD-Asn	2,373609262	5,182360088	1,10E-15	-0,5948534	0,662111731	0,013868816	2,922235909
BSU_tRNA_30	TrnD-Ser	3,222290391	9,332673285	1,87E-19	-1,64805275	0,319070527	3,35E-10	4,825871906
BSU_tRNA_35	TrnD-Phe	2,845668386	7,188388517	5,08E-18	-0,42317485	0,745781623	0,097981537	3,96708507
BSU_tRNA_38	TrnD-Trp	4,34673406	20,34685712	1,42E-21	-1,26917568	0,414896767	3,51E-07	10,38087694
BSU_tRNA_39	TrnD-His	4,001360527	16,01509585	5,43E-21	-1,21250904	0,431517496	4,92E-07	8,223306672
BSU_tRNA_40	TrnD-Gln	5,527728934	46,13305518	2,32E-22	-1,8562775	0,276187993	5,59E-11	23,20462159
BSU_tRNA_42	TrnD-Cys	3,600007595	12,12579637	7,26E-20	-1,53943696	0,344019688	7,81E-09	6,234908028
BSU_tRNA_43	TrnD-Leu1	3,528645503	11,54059341	4,44E-20	-0,47910026	0,71742491	0,035500591	6,12900916
BSU_tRNA_44	TrnD-Leu2	5,137047672	35,18887953	2,84E-22	-2,6435875	0,160029801	3,17E-16	17,67445466
BSU_tRNA_45	TrnS-Asn	2,45480841	5,482403109	1,04E-16	-0,71702798	0,608349383	0,002537899	3,045376246
BSU_tRNA_46	TrnS-Ser	3,449706333	10,92609777	1,11E-19	-1,23231006	0,425635367	4,34E-07	5,675866571
BSU_tRNA_48	TrnS-Gln	4,644434634	25,01002568	5,48E-22	-1,92678996	0,263013733	1,29E-11	12,63651971
BSU_tRNA_5	TrnO-Ile	2,943016146	7,69017349	7,91E-18	-0,26879097	0,830014838	0,42657682	4,260094164
BSU_tRNA_50	TrnS-Leu1	4,061263229	16,69406315	8,55E-21	-0,93085764	0,524546422	6,75E-05	8,609304788
BSU_tRNA_51	TrnS-Leu2	3,985634694	15,84147421	9,43E-21	-2,24304731	0,211239669	8,11E-14	8,02635694
BSU_tRNA_55	TrnB-Leu1	3,865751096	14,57830512	1,32E-20	-1,73660085	0,300075856	8,74E-10	7,439190486
BSU_tRNA_6	TrnO-Ala	2,830959904	7,115474188	1,19E-17	1,272897231	2,416463546	4,77E-12	4,765968867
BSU_tRNA_60	TrnB-Ala	2,885173813	7,387948518	9,85E-18	1,230405424	2,346329168	7,09E-12	4,867138843
BSU_tRNA_62	TrnB-Met3	2,593748064	6,03664961	9,30E-18	-0,74342738	0,597318629	0,001723058	3,31698412

BSU_tRNA_63	TrnB-Ser1	3,501975828	11,32921369	3,57E-20	-1,6410918	0,320613749	2,50E-10	5,824913718
BSU_tRNA_67	TrnB-His	3,572935224	11,90037577	1,64E-20	-0,98440088	0,505435575	2,58E-05	6,202905674
BSU_tRNA_68	TrnB-Gly2	3,189153907	9,120759135	2,53E-18	-1,19450373	0,436936725	1,42E-05	4,77884793
BSU_tRNA_69	TrnB-Ile2	2,850771392	7,213859834	1,99E-17	-0,26864218	0,830100446	0,344190547	4,02198014
BSU_tRNA_7	TrnSL-Ser1	3,808410554	14,01024767	1,85E-20	-1,62119623	0,325065819	3,59E-10	7,167656743
BSU_tRNA_71	TrnB-Ser2	3,252963932	9,533222257	2,70E-19	-1,13484129	0,455385009	2,56E-06	4,994303633
BSU_tRNA_73	TrnSL-Gln1	3,980777674	15,7882315	1,88E-20	-1,21873919	0,429658044	4,91E-06	8,108944773
BSU_tRNA_74	TrnSL-Gln2	4,694999865	25,90214851	9,10E-22	-1,83631854	0,280035466	2,45E-11	13,09109199
BSU_tRNA_76	TrnSL-Thr1	4,435794487	21,64248848	2,67E-21	-1,56012574	0,339121525	2,15E-09	10,990805
BSU_tRNA_77	TrnSL-Tyr1	3,311274573	9,926427393	2,66E-19	-1,76896839	0,293418474	9,21E-11	5,109922934
BSU_tRNA_80	TrnE-Gly	2,720602432	6,591479996	1,50E-16	-1,1188999	0,460444797	5,00E-05	3,525962396
BSU_tRNA_81	TrnQ-Arg	3,977754611	15,75518305	8,04E-21	-1,6425179	0,32029698	1,42E-10	8,037740015
BSU_tRNA_83	TrnSL-Val2	2,906064577	7,495707083	7,39E-18	0,04903034	1,034569339	0,012276843	4,265138211
BSU_tRNA_84	TrnSL-Arg2	3,47969545	11,15559416	5,89E-20	-1,00100194	0,499652876	1,06E-05	5,827623519
BSU_tRNA_85	TrnSL-Arg1	3,428231046	10,76466148	6,47E-13	-1,67511062	0,313142099	2,44E-06	5,538901789
BSU_tRNA_86	TrnSL-Ala1	3,630570837	12,38541957	3,78E-19	1,118772091	2,171620625	1,26E-09	7,278520095
BSU00010	DnaA	0,327950127	1,255228598	0,008859793	0,628213786	1,545650124	0,002167312	1,400439361
BSU00020	DnaN	-1,30760086	0,403992144	1,76E-06	-1,30400551	0,40500019	6,54E-06	0,404496167
BSU00030	YaaA	-0,31251034	0,805239393	0,008732419	-0,02044919	0,985925684	0,188626147	0,895582538
BSU00040	RecF	0,142914265	1,10413323	0,125797641	0,355090474	1,279065791	0,015695723	1,191599511
BSU00050	RemB	-0,00584427	0,995957253	0,510003481	0,717159514	1,643942129	0,000463505	1,319949691
BSU00060	GyrB	0,504442241	1,418574816	0,018448844	0,032809232	1,023002189	0,290783171	1,220788503
BSU00070	GyrA	0,588662586	1,503851993	0,002818199	0,328128429	1,255383741	0,060630835	1,379617867
**BSU00080**	**YaaC**	**-2,64411604**	**0,159971184**	**1,59E-14**	**-1,18316534**	**0,440384214**	**1,57E-06**	**0,300177699**
BSU00090	GuaB	-0,20927475	0,864971946	0,198265028	0,149660606	1,109308477	0,260638747	0,987140211
BSU00100	DacA	0,551834118	1,465948192	0,017245899	0,604891026	1,52086387	0,001807762	1,493406031
BSU00110	PdxS	-0,36695661	0,775416527	0,009097402	0,615891074	1,532504249	0,000976483	1,153960388
BSU00120	PdxT	-0,45933259	0,727322652	0,081144273	0,656719192	1,576493468	0,002104444	1,15190806
BSU00130	SerS	-0,32883177	0,796180936	0,067702893	0,11627685	1,083933951	0,127274312	0,940057444

BSU00140	Dck	1,027238535	2,038119348	0,000545591	0,384249272	1,30518044	0,114971197	1,671649894
BSU00150	Dgk	-0,29282233	0,816303567	0,169602481	0,094178404	1,067457332	0,300808664	0,94188045
BSU00160	YaaH	-0,1027595	0,931250048	0,274250323	-0,65357034	0,635705142	0,016355244	0,783477595
BSU00170	YaaI	0,258812581	1,196493518	0,177190064	0,818297238	1,763323575	0,036709036	1,479908546
BSU00180	YaaJ	0,001975259	1,001370083	0,004383864	-0,03753095	0,974320991	0,220381862	0,987845537
BSU00190	DnaX	-0,49144503	0,711312278	0,037454637	-0,11108681	0,92589031	0,584585008	0,818601294
BSU00200	YaaK	-0,42443797	0,745128953	0,066571069	-0,2342703	0,850114871	0,407928862	0,797621912
BSU00210	RecR	-1,15581074	0,448813898	9,94E-07	-0,46549973	0,724220174	0,100900546	0,586517036
BSU00220	YaaL	-0,36274441	0,777683797	0,004189137	-0,49860341	0,707791624	0,027224603	0,742737711
BSU00230	BofA	1,338965983	2,529699437	8,98E-09	-0,17645848	0,884872513	0,574755386	1,707285975
BSU00240	Gin	1,543763762	2,915541306	5,95E-11	-0,0654964	0,955616453	0,133521447	1,935578879
**BSU00250**	**XpaC**	**1,863284165**	**3,638349576**	**1,37E-12**	**2,948117282**	**7,717412829**	**4,24E-18**	**5,677881203**
**BSU00260**	**YaaN**	**1,28798092**	**2,441860732**	**2,19E-08**	**2,660539502**	**6,322694451**	**6,01E-17**	**4,382277592**
**BSU00270**	**YaaO**	**0,96783196**	**1,955899117**	**1,92E-06**	**2,47822516**	**5,572115488**	**1,82E-16**	**3,764007303**
BSU00280	Tmk	0,739051511	1,669078155	1,47E-06	2,457835476	5,493918373	5,84E-16	3,581498264
BSU00290	YaaQ	0,670404192	1,591518792	0,000871477	1,578222587	2,98601743	3,34E-11	2,288768111
BSU00300	YaaR	0,58592249	1,500998453	0,000296208	1,663330222	3,167468391	1,98E-11	2,334233422
BSU00310	HolB	0,815490965	1,759896962	3,20E-05	1,663354473	3,167521634	2,14E-11	2,463709298
**BSU00320**	**YaaT**	**1,580246219**	**2,990208781**	**3,88E-09**	**1,455665769**	**2,742831068**	**6,50E-10**	**2,866519925**
**BSU00330**	**YabA**	**0,926476284**	**1,900628126**	**1,90E-05**	**1,715992861**	**3,285226547**	**8,69E-11**	**2,592927336**
BSU00340	YabB	0,401487909	1,320869474	0,004087282	1,317543373	2,492413391	1,53E-08	1,906641432
BSU00350	YazA	0,334059931	1,260555755	0,126267102	0,895348274	1,860058858	4,52E-06	1,560307307
BSU00360	YabC	1,078328262	2,111587838	1,79E-05	1,089067837	2,127365374	2,23E-05	2,119476606
BSU00370	AbrB	1,148854567	2,21737775	9,20E-07	0,55861035	1,472849838	0,014314852	1,845113794
BSU00380	MetS	0,013470598	1,009380834	0,152322957	0,803454667	1,74527535	5,59E-05	1,377328092
BSU00390	YabD	0,319495799	1,247894352	0,078728507	0,982357284	1,975690942	2,44E-05	1,611792647
BSU00400	YabE	1,429227201	2,693024213	2,47E-09	0,143913055	1,104897896	0,269764228	1,898961054
BSU00410	RnmV	-0,29498455	0,815081062	0,264770583	0,518246518	1,432213447	0,014642743	1,123647255
BSU00420	KsgA	-0,3620643	0,778050502	0,047523456	-0,01433937	0,99010994	0,282427557	0,884080221

**BSU00430**	**YabG**	**-2,33692558**	**0,197931676**	**5,27E-06**	**-1,34693794**	**0,393125556**	**0,000472927**	**0,295528616**
BSU00440	Veg	0,103206866	1,074158483	0,033187203	-1,1481857	0,451192284	4,83E-07	0,762675383
**BSU00450**	**SspF**	**-1,93982608**	**0,26064786**	**2,10E-09**	**-2,92378876**	**0,131780722**	**4,82E-17**	**0,196214291**
BSU00460	IspE	0,844348615	1,795453906	9,57E-05	-0,38993931	0,763161708	0,088937375	1,279307807
BSU00470	PurR	0,725267041	1,653206615	0,000105414	-0,83615626	0,560133934	0,000444377	1,106670274
BSU00480	YabJ	1,060829403	2,08613049	1,25E-06	-0,49020713	0,711922877	0,060182112	1,399026684
BSU00490	SpoVG	2,280255711	4,857640454	8,64E-16	0,425505219	1,343042742	0,007095852	3,100341598
BSU00500	GcaD	-0,64001511	0,641706228	0,00095453	0,350017351	1,274575957	0,062047164	0,958141092
BSU00510	Prs	-0,97157659	0,509948482	0,000621691	0,399196827	1,318773522	0,040621116	0,914361002
BSU00520	Ctc	0,609551805	1,525785127	0,001732392	0,218817844	1,163779585	0,163296859	1,344782356
BSU00530	SpoVC	-0,5663738	0,675312047	0,013163637	0,496724829	1,41100669	0,013689907	1,043159369
BSU00540	Fin	-0,99428541	0,501984454	1,13E-05	-0,5862416	0,666075858	0,027800595	0,584030156
BSU00550	Mfd	-0,854096	0,553211865	0,000260161	0,134562532	1,09775989	0,236271404	0,825485877
**BSU00560**	**SpoVT**	**-2,05369205**	**0,240866882**	**1,51E-12**	**-1,33048951**	**0,397633302**	**9,11E-07**	**0,319250092**
BSU00570	YabM	-0,85731038	0,551980659	3,33E-07	0,095944131	1,068764602	0,214655595	0,81037263
BSU00580	YabN	-0,4669268	0,72350415	0,01207501	0,261168415	1,198448916	0,143358662	0,960976533
BSU00590	YabO	-0,89656066	0,537165792	0,000637116	-0,43379429	0,740312203	0,052881429	0,638738998
BSU00600	YabP	1,113671261	2,163956144	1,26E-05	0,483726019	1,398350498	0,010557448	1,781153321
BSU00610	YabQ	0,464146459	1,379500958	0,055208463	0,498676893	1,412917171	0,002747125	1,396209064
BSU00620	DivIC	0,432869437	1,349915813	0,05206761	0,577862832	1,49263646	0,002095574	1,421276137
BSU00630	YabR	0,770313337	1,705640189	7,00E-06	0,112789931	1,081317303	0,479327106	1,393478746
BSU00640	SpoIIE	1,149578825	2,218491191	1,51E-07	0,970554444	1,959593544	4,88E-07	2,089042368
BSU00650	YabS	0,789611688	1,728609132	3,40E-05	0,500738029	1,414937207	0,002517572	1,571773169
BSU00660	YabT	1,218360016	2,326820654	1,36E-05	0,350397592	1,274911932	0,02464638	1,800866293
BSU00670	TilS	-0,38866665	0,763835223	0,098110031	-0,13178442	0,912701861	0,418930395	0,838268542
BSU00680	HprT	-0,80334213	0,573020188	0,001057867	-0,0809323	0,94544648	0,606077121	0,759233334
BSU00690	FtsH	-0,21190089	0,86339887	0,117305561	0,001933027	1,00134077	0,127313339	0,93236982
BSU00700	CoaX	-0,60561841	0,657189614	0,000518426	-0,26908219	0,829847307	0,004368647	0,743518461
BSU00710	YacC	-0,9570883	0,515095449	1,78E-05	-0,0895716	0,939801776	0,376637384	0,727448613

BSU00720	YacD	-0,30677115	0,808449099	5,34E-06	0,249854794	1,189087428	0,000885374	0,998768263
BSU00730	CysK	-1,17259337	0,443623172	5,18E-08	-0,87319222	0,545937529	2,94E-05	0,49478035
BSU00740	PabB	-0,40553979	0,75495377	0,15625735	0,73038751	1,659084665	0,003010256	1,207019217
BSU00750	PabA	-0,37696203	0,770057444	0,162833359	0,28175745	1,215674883	0,016163986	0,992866164
BSU00760	PabC	-0,54255536	0,686553779	0,005546578	0,808533808	1,751430581	2,65E-05	1,21899218
BSU00770	Sul	-0,03084121	0,978849381	0,198718771	0,738344936	1,668260907	0,000319639	1,323555144
BSU00780	FolB	-0,24548596	0,843531611	0,444099675	0,821207435	1,766884133	0,000148659	1,305207872
BSU00790	FolK	-0,24730865	0,842466576	0,42577817	0,585972278	1,501050254	0,002130983	1,171758415
BSU00800	YazB	0,118292902	1,085449722	0,112454266	0,518716792	1,43268038	0,005869815	1,259065051
BSU00810	YacF	-0,24152694	0,845849595	0,410332387	0,539653199	1,453623048	0,004549061	1,149736321
BSU00820	LysS	-0,07540538	0,949075404	0,010039109	0,069754438	1,049538026	0,292387006	0,999306715
BSU00830	CtsR	0,313683259	1,242876768	0,070997779	0,541958362	1,455947528	0,00405567	1,349412148
BSU00840	McsA	0,116062918	1,083773231	0,050425836	0,714239187	1,6406178	0,000461346	1,362195516
BSU00850	McsB	-0,3574956	0,780518321	0,080545878	0,713950956	1,64029006	0,000792707	1,210404191
BSU00860	ClpC	-0,2718464	0,828258841	0,054024695	0,284437099	1,217934966	0,027191774	1,023096904
BSU00870	RadA	-0,14082767	0,906998665	0,668185603	0,601058403	1,516828949	0,002363423	1,211913807
BSU00880	DisA	0,221551601	1,165986917	0,037732756	0,828076421	1,775316712	7,69E-05	1,470651815
BSU00890	YacL	0,175360406	1,129246472	0,03658509	0,520839139	1,434789546	0,00323768	1,282018009
BSU00900	IspD	-0,51796455	0,698356425	0,041938596	1,00998363	2,013888248	3,39E-05	1,356122337
BSU00910	IspF	-0,56166431	0,677520118	0,020936391	0,758961066	1,692271524	0,000311526	1,184895821
BSU00920	GltX	-0,36982268	0,773877605	0,039681975	0,135041197	1,098124171	0,302489373	0,936000888
BSU00930	CysE	-0,87221204	0,546308571	8,02E-05	0,28550689	1,218838426	0,127694394	0,882573499
BSU00940	CysS	-1,06062752	0,479423484	3,57E-05	0,044911858	1,03162015	0,47870935	0,755521817
BSU00950	MrnC	-0,86873815	0,547625623	0,001354779	0,350305234	1,274830317	0,039064898	0,91122797
BSU00960	YacO	-0,04213577	0,971216094	0,063932682	0,56143566	1,475737028	0,002402709	1,223476561
BSU00970	YacP	-0,16138331	0,8941673	0,550215608	0,458016067	1,373651531	0,01395334	1,133909416
BSU00980	SigH	0,849885343	1,802357678	5,94E-05	0,897076681	1,86228862	6,35E-06	1,832323149
BSU00990	RpmGB	1,279123854	2,42691546	3,04E-09	0,977099574	1,968503904	6,94E-06	2,197709682
BSU01000	SecE	1,940383505	3,838076599	6,61E-13	0,604061973	1,519990148	0,002586905	2,679033374

BSU01010	NusG	-0,16569917	0,89149637	0,560872531	0,232535364	1,174897878	0,16360387	1,033197124
BSU01020	RplK	0,155468818	1,113783495	0,198216422	0,463898562	1,379263941	0,000948828	1,246523718
BSU01030	RplA	-0,20092281	0,869993898	0,076151904	0,655836606	1,575529323	7,85E-05	1,222761611
BSU01040	RplJ	-0,44323873	0,735481661	0,020361382	0,404619275	1,323739531	0,009230782	1,029610596
BSU01050	RplL	0,145403269	1,106039775	0,000545586	0,117726308	1,085023514	0,316164851	1,095531645
BSU01060	YbxB	-0,14328332	0,90545615	0,375084282	0,831759275	1,779854459	0,000277833	1,342655305
BSU01070	RpoB	0,097525477	1,069936723	0,001428367	0,97082673	1,959963422	1,44E-08	1,514950072
BSU01080	RpoC	0,064674164	1,045848706	0,407631966	0,584068489	1,499070765	0,000144488	1,272459736
BSU01090	YbxF	0,168633401	1,123993273	0,004358029	0,602356086	1,518193928	4,66E-05	1,3210936
BSU01100	RpsL	-0,21857372	0,859414653	0,018492001	0,553072015	1,467206581	4,40E-05	1,163310617
BSU01110	RpsG	-0,12058426	0,919815069	0,244946143	0,741073559	1,671419137	4,32E-06	1,295617103
BSU01120	FusA	0,09437221	1,067600739	0,402839205	1,152402768	2,222837937	4,82E-10	1,645219338
BSU01130	TufA	0,47819403	1,392998814	0,029053264	0,311000892	1,240568064	0,000298849	1,316783439
BSU01140	YbaC	-0,11062399	0,926187386	0,02634984	0,044837226	1,031566785	0,350496703	0,978877086
BSU01150	RpsJ	-0,81595695	0,568031582	0,00012567	0,467483014	1,382695055	0,001201254	0,975363319
BSU01160	RplC	-0,7902309	0,578251538	0,002087809	0,642643083	1,561186706	2,27E-05	1,069719122
BSU01170	RplD	-0,43799403	0,73816026	0,008906192	0,650998813	1,570254945	9,93E-06	1,154207603
BSU01180	RplW	-0,72238975	0,606092649	0,003418658	0,544120046	1,458130704	4,76E-05	1,032111677
BSU01190	RplB	-0,42885475	0,742851248	0,019107569	0,700487361	1,625053664	3,64E-06	1,183952456
BSU01200	RpsS	-0,43199762	0,741234726	0,025963272	0,607569934	1,523690552	4,94E-05	1,132462639
BSU01210	RplV	-0,57797719	0,669902393	0,004098528	0,594703425	1,510162109	4,85E-05	1,090032251
BSU01220	RpsC	-0,58315454	0,667502648	0,00887879	0,715539326	1,642096971	2,54E-06	1,15479981
BSU01230	RplP	-0,63142636	0,64553787	0,001626483	0,804736862	1,746827154	6,51E-06	1,196182512
BSU01240	RpmC	-0,46061081	0,72667853	0,00478095	0,767780215	1,702648009	2,56E-05	1,21466327
BSU01250	RpsQ	-0,60979103	0,655291614	0,008213112	0,601916693	1,517731611	5,64E-05	1,086511612
BSU01260	RplN	-0,52004944	0,697347936	0,002206273	0,819972567	1,765372424	3,97E-07	1,23136018
BSU01270	RplX	-0,5293373	0,692872933	0,004357058	0,66602602	1,586696303	1,81E-05	1,139784618
BSU01280	RplE	-0,49663791	0,708756559	0,001571148	0,891580766	1,855207768	3,04E-07	1,281982164
BSU01290	RpsN	-0,49604159	0,709049576	0,020033023	0,561790602	1,476100144	0,000254059	1,09257486

BSU01300	RpsH	-0,62759565	0,647254209	0,001661472	0,806076753	1,748450258	3,77E-06	1,197852234
BSU01310	RplF	0,142335045	1,103690027	0,043778163	0,94914716	1,930730981	3,34E-07	1,517210504
BSU01320	RplR	-0,7480853	0,595393221	0,000372988	0,857285693	1,811626682	9,26E-07	1,203509952
BSU01330	RpsE	-0,90226758	0,535045105	5,47E-05	0,99450391	1,992395297	3,06E-07	1,263720201
BSU01340	RpmD	-0,95881053	0,514480917	5,27E-05	0,987086099	1,982177418	2,85E-07	1,248329168
BSU01350	RplO	-0,39632021	0,759793771	0,006748681	1,163555001	2,240087368	9,72E-09	1,499940569
BSU01360	SecY	-0,66015859	0,632808732	0,000258739	1,091567109	2,131053939	5,13E-09	1,381931336
BSU01370	Adk	-0,68041075	0,623987593	0,000727898	1,151806844	2,221919954	5,15E-09	1,422953774
BSU01380	Map	-0,77562203	0,584136714	7,27E-05	0,971163167	1,960420539	1,39E-07	1,272278626
BSU01389	YbzG	0,277488919	1,212083357	0,062768753	0,816522888	1,761156222	2,16E-06	1,48661979
BSU01390	InfA	-0,58727231	0,665600164	0,008826152	0,785132348	1,723250398	3,74E-06	1,194425281
BSU01400	RpmJ	-0,90969031	0,532299344	3,30E-05	0,803263597	1,745044222	1,86E-05	1,138671783
BSU01410	RpsM	0,243330016	1,183721767	0,024173236	0,686805699	1,609715465	1,14E-05	1,396718616
BSU01420	RpsK	-0,41807757	0,748421253	0,031699818	0,606116612	1,522156411	9,37E-05	1,135288832
BSU01430	RpoA	0,071224007	1,050607659	0,168107318	0,782875024	1,720556208	4,07E-06	1,385581934
BSU01440	RplQ	-0,21437003	0,861922447	0,041711308	0,450589618	1,36659866	0,003613019	1,114260554
BSU01450	YbxA	-0,00158915	0,998899093	0,253568364	0,907779488	1,876155606	1,02E-05	1,437527349
BSU01460	YbaE	-0,34915247	0,785045146	0,029479846	1,435991578	2,705680667	1,29E-09	1,745362907
BSU01470	YbaF	0,103323123	1,074245045	0,102867697	1,487245585	2,803532094	1,21E-09	1,93888857
BSU01480	TruA	0,270192579	1,205968796	0,030615094	1,000090675	2,000125706	1,84E-06	1,603047251
BSU01490	RplM	0,512995083	1,427009642	0,018920967	-0,19043045	0,876344213	0,850939822	1,151676927
BSU01500	RpsI	0,619568526	1,536415609	2,17E-05	-0,67782444	0,625107217	0,001434475	1,080761413
BSU01510	YbaJ	0,595670108	1,511174338	0,000693817	0,324866864	1,252548845	0,06621735	1,381861591
**BSU01520**	**YbaK**	**-2,50514054**	**0,176147934**	**6,48E-12**	**-1,93444587**	**0,261621703**	**1,23E-10**	**0,218884819**
BSU01530	CwlD	-0,40099965	0,757333343	0,085932176	-0,66073847	0,632554428	0,000359284	0,694943885
BSU01540	SalA	-0,36856472	0,774552688	0,135487921	-0,33716324	0,791596292	0,37260685	0,78307449
**BSU01550**	**GerD**	**-1,71918449**	**0,303720355**	**1,78E-10**	**-1,84218755**	**0,278898572**	**5,52E-12**	**0,291309464**
BSU01560	KbaA	0,114126373	1,082319446	0,035203501	-0,55941162	0,678578853	0,024637591	0,88044915
BSU01570	PdaB	0,484861973	1,39945197	0,025523673	-0,19857562	0,871410486	0,460231972	1,135431228

BSU01580	YbaR	0,805229369	1,747423587	4,12E-05	0,371617116	1,29380224	0,0273644	1,520612913
BSU01590	YbaS	0,839715877	1,789697646	2,63E-05	-0,08607382	0,942083065	0,446348889	1,365890356
BSU01600	YbbA	-0,43329703	0,740567409	0,057037696	0,802786333	1,744467032	6,53E-05	1,24251722
BSU01610	FeuC	-0,19269073	0,87497231	0,052626762	1,154346069	2,225834103	3,62E-07	1,550403207
BSU01620	FeuB	0,375000596	1,29684009	0,001423075	1,488355581	2,805689933	1,33E-09	2,051265012
BSU01630	FeuA	-0,41020614	0,752515842	0,106593299	1,134475438	2,195387242	2,37E-07	1,473951542
BSU01640	Btr	0,000440451	1,000305344	0,618021012	0,718545302	1,645521985	0,000326982	1,322913664
**BSU01650**	**YbbC**	**-1,44097147**	**0,368319206**	**1,82E-09**	**-1,54257055**	**0,343273275**	**6,68E-09**	**0,355796241**
**BSU01660**	**NagZ**	**-1,71642439**	**0,304301977**	**5,10E-10**	**-1,3748559**	**0,385591218**	**1,79E-07**	**0,344946597**
BSU01670	AmiE	-2,03035577	0,244794701	6,62E-13	-0,79959403	0,57451082	0,001526715	0,409652761
BSU01680	MurP	-1,87337269	0,272934621	2,25E-11	-0,7586727	0,591039844	0,009941627	0,431987233
BSU01690	MurR	-1,74243942	0,298863908	3,84E-12	-0,97635453	0,50826242	9,22E-05	0,403563164
**BSU01700**	**MurQ**	**-2,12160253**	**0,229791521**	**8,99E-13**	**-1,44864145**	**0,366366259**	**2,46E-08**	**0,29807889**
BSU01710	YbbJ	0,375669058	1,297441111	0,304206037	-0,28083913	0,823112125	0,157931072	1,060276618
BSU01720	YbbK	0,694113363	1,617889821	0,001443422	-0,1815405	0,881760956	0,449353404	1,249825388
BSU01730	SigW	0,970100963	1,958977684	0,000239223	0,393749091	1,313803115	0,012701772	1,636390399
BSU01740	RsiW	1,161231667	2,236482806	2,07E-07	0,258669708	1,196375033	0,161763678	1,71642892
BSU01750	YbbP	-0,87558738	0,545031917	3,96E-05	-0,38171967	0,767522172	0,130152468	0,656277044
BSU01760	YbbR	-0,90125646	0,535420223	0,00059516	-0,42499584	0,74484088	0,083118834	0,640130552
BSU01770	GlmM	-0,4561704	0,72891859	0,064806953	-0,58815358	0,665193705	0,013013292	0,697056147
BSU01780	GlmS	0,809278274	1,752334597	7,12E-05	-0,14028745	0,907338356	0,429229011	1,329836476
BSU01800	AlkA	-0,98512372	0,5051824	0,000266053	-0,48451268	0,714738455	0,037782906	0,609960427
BSU01810	AdaA	-1,2889026	0,40926222	4,64E-07	0,220943743	1,16549575	0,433009217	0,787378985
BSU01820	AdaB	-1,25526732	0,418915938	0,000736779	-0,19833261	0,871557281	0,183277805	0,645236609
BSU01830	NdhF	0,372714068	1,294786356	0,432376846	-0,45586888	0,729070949	0,08994653	1,011928653
BSU01845	YbcC	-1,3654159	0,388122533	0,002014664	0,892017592	1,855769582	0,037953691	1,121946058
BSU01860	YbcF	-0,1623364	0,893576777	0,143157136	0,003259371	1,002261778	0,161975908	0,947919278
BSU01870	YbcH	-0,54697558	0,684453492	0,038456877	0,214286019	1,160129631	0,484778227	0,922291561
BSU01880	YbcI	1,297603327	2,458201753	4,95E-08	0,086189854	1,061562898	0,567089445	1,759882326

BSU01889	YbzH	0,061499628	1,043549928	0,729759572	-0,18613232	0,878958942	0,095891773	0,961254435
BSU01890	YbcL	0,614280779	1,530794666	0,134673637	0,518528834	1,432493739	0,159468922	1,481644203
BSU01900	YbcM	-0,32390221	0,798906065	0,671594926	0,4226783	1,340413666	0,423049032	1,069659865
BSU01910	SkfA	2,165032223	4,484764479	5,91E-13	0,226038422	1,169618813	0,003335838	2,827191646
BSU01920	SkfB	0,190210411	1,140930104	0,482618352	1,419901388	2,675672214	4,52E-10	1,908301159
BSU01935	SkfC	-0,37074263	0,773384296	0,057131111	1,675666275	3,194668589	8,51E-12	1,984026442
BSU01950	SkfE	-0,42970219	0,742415024	0,035716071	1,527996696	2,883851138	7,03E-11	1,813133081
BSU01960	SkfF	0,00785086	1,005456635	0,49230477	1,419726044	2,675347035	9,03E-10	1,840401835
BSU01970	SkfG	0,902845706	1,869750417	0,000361278	1,248535574	2,376001215	6,37E-08	2,122875816
BSU01980	SkfH	0,162488493	1,119216001	0,122541765	1,06743359	2,095702005	2,54E-07	1,607459003
BSU01990	YbdG	-0,23988518	0,846812706	0,238676672	1,183193598	2,270788905	3,04E-07	1,558800805
BSU02000	YbdJ	0,518330171	1,432296495	0,005652011	1,06575034	2,093258292	2,61E-06	1,762777393
BSU02010	YbdK	0,350803228	1,275270443	0,085207584	0,929995477	1,905270023	9,69E-05	1,590270233
BSU02019	YbzI	-0,13885098	0,908242226	0,084926482	0,252968488	1,191656545	0,350893881	1,049949386
BSU02030	PrkD	-1,59670771	0,33063063	1,31E-07	1,90334835	3,740803929	0,011837467	2,035717279
BSU02040	YbdN	0,185991607	1,13759861	0,155529129	0,156179276	1,114332114	0,244273397	1,125965362
BSU02050	YbdO	0,017469798	1,012182754	0,141201171	-0,35018156	0,784485364	0,207670053	0,898334059
BSU02060	YbxG	2,114456447	4,330268392	4,24E-13	0,916789654	1,887909559	3,94E-06	3,109088976
**BSU02070**	**CsgA**	**-2,85846887**	**0,137884399**	**1,90E-17**	**-1,68418918**	**0,311177751**	**2,76E-10**	**0,224531075**
**BSU02080**	**YbxH**	**-2,63069408**	**0,161466404**	**6,28E-16**	**-1,66919868**	**0,31442794**	**8,78E-11**	**0,237947172**
BSU02090	YbxI	1,025749927	2,03601745	0,002835698	-0,15783135	0,89637148	0,106476849	1,466194465
BSU02100	CypC	0,482419851	1,397085051	0,000257087	0,203622822	1,151586532	0,12597486	1,274335792
BSU02110	YbyB	1,806245428	3,497309385	4,22E-12	-0,09343514	0,937288351	0,818051473	2,217298868
BSU02120	YbeC	0,654884872	1,574490303	3,24E-05	0,933101549	1,909376421	5,96E-06	1,741933362
BSU02130	GlpQ	-0,90100338	0,535514158	0,000732739	1,285860839	2,438274983	5,09E-08	1,486894571
BSU02140	GlpT	-1,01006273	0,496524658	4,67E-05	1,680259436	3,204855779	2,80E-11	1,850690219
BSU02150	YbeF	0,622932856	1,540002674	0,064487122	-1,12398944	0,458823301	4,46E-06	0,999412987
BSU02160	YbfA	-0,31407922	0,804364203	0,002638659	0,55562957	1,469809896	0,019204143	1,137087049
BSU02170	YbfB	0,845041668	1,796316627	0,000702229	0,59053864	1,505808847	0,003085165	1,651062737

BSU02180	YbfE	-0,20658457	0,866586357	0,433292354	-0,87372094	0,54573749	5,86E-05	0,706161923
BSU02190	YbfF	0,314960691	1,243977758	0,038768815	0,668239004	1,589132042	0,000579999	1,4165549
**BSU02200**	**YbfG**	**1,681840931**	**3,208370897**	**2,63E-10**	**1,183397666**	**2,271110129**	**8,52E-09**	**2,739740513**
BSU02210	YbfH	0,51203667	1,426061964	0,001033404	0,348663153	1,273380126	0,076247478	1,349721045
BSU02220	YbfI	0,073891735	1,052552165	0,266169893	0,762656299	1,696611562	8,15E-05	1,374581863
BSU02230	PurT	-0,37383953	0,771725923	0,32508275	-0,15406585	0,898714104	0,095383785	0,835220013
**BSU02240**	**Mpr**	**-2,07045288**	**0,238084749**	**1,26E-13**	**-1,1399409**	**0,453778165**	**9,01E-05**	**0,345931457**
**BSU02250**	**YbfJ**	**-1,8680142**	**0,273950245**	**1,83E-09**	**-1,38670325**	**0,382437725**	**1,25E-07**	**0,328193985**
BSU02260	YbfK	0,464400285	1,379743688	0,00878448	-0,20337108	0,868518763	0,185424587	1,124131225
BSU02270	PssA	0,404608857	1,323729973	0,012036559	0,444224118	1,360582198	0,043462195	1,342156085
BSU02280	YbfM	0,009384901	1,006526322	0,102062242	0,630858576	1,548486254	0,002628633	1,277506288
BSU02290	Psd	0,080027646	1,057038296	0,147052685	0,725717144	1,653722475	0,000868076	1,355380385
BSU02300	YbfN	-0,54071028	0,687432383	0,694267029	-0,30578845	0,80899997	0,82242285	0,748216176
BSU02310	YbfO	0,72845446	1,656863166	0,000751635	0,335567161	1,261873387	0,104365037	1,459368276
BSU02320	YbfP	-0,50782701	0,703280926	0,027187486	0,353417153	1,277583117	0,148905342	0,990432021
BSU02330	YbfQ	-0,05452836	0,962909181	0,626065997	0,149871001	1,109470264	0,233734462	1,036189723
BSU02340	GltP	1,344202558	2,538898214	1,38E-09	0,614773233	1,531317282	0,000325847	2,035107748
BSU02350	GamP	-1,37844568	0,384632966	0,114419726	2,252327505	4,764508863	0,001047727	2,574570915
BSU02360	GamA	-2,24836608	0,210462328	4,25E-06	1,183910552	2,271917664	0,000319865	1,241189996
BSU02370	YbgA	-0,3387844	0,790707273	0,098438109	0,310967459	1,240539316	0,271964272	1,015623294
BSU02380	YbgB	0,126085721	1,091328719	0,001521603	-0,27140995	0,828509445	0,190692267	0,959919082
BSU02390	YbgE	-0,44788876	0,733114902	0,010786445	-0,07076571	0,952132518	0,371647292	0,84262371
BSU02400	YbgF	0,656500988	1,576255045	0,000800689	0,761810621	1,695617335	0,000391303	1,63593619
BSU02410	YbgG	-0,78903386	0,578731527	0,012494531	0,056779999	1,04014164	0,673135567	0,809436583
BSU02420	GlnT	-0,61159843	0,65447118	0,141638787	0,408305389	1,327126036	0,128015674	0,990798608
BSU02430	GlsA	-1,17466504	0,442986598	0,405339625	0,516686109	1,430665212	0,584625891	0,936825905
BSU02440	GlnK	-0,15236356	0,899775156	0,3289	0,911959867	1,881599871	0,016700955	1,390687514
BSU02450	GlnL	-0,07433864	0,949777418	0,519429095	0,88264099	1,843747368	0,045600958	1,396762393
BSU02460	YcbC	0,049306161	1,034767151	4,05E-05	1,472882696	2,775759735	1,77E-05	1,905263443

BSU02470	YcbD	-0,2701378	0,829240335	0,13080244	1,228725012	2,343597818	1,09E-05	1,586419077
BSU02480	YcbE	0,32676439	1,254197362	0,001266318	1,809616481	3,505490879	2,15E-09	2,37984412
BSU02490	YcbF	-0,26031005	0,834908468	0,030572994	1,476988357	2,783670318	2,30E-09	1,809289393
BSU02500	YcbG	0,148797401	1,108644945	0,295764531	0,912430833	1,882214218	6,39E-05	1,495429581
BSU02510	YcbH	0,129363015	1,09381065	0,140807332	1,076916223	2,109522131	4,75E-06	1,60166639
BSU02520	YcbJ	-1,20131769	0,434877902	2,56E-07	-0,09838635	0,934077169	0,731018403	0,684477535
BSU02530	RtpA	-0,663225	0,631465142	1,61E-07	-0,26478379	0,832323465	0,330779352	0,731894303
BSU02540	YcbK	0,345663087	1,270734899	0,138398877	0,073310613	1,052128279	0,354928633	1,161431589
BSU02550	YcbL	-0,72685321	0,604220398	0,000749013	0,403161065	1,322402232	0,065314691	0,963311315
BSU02560	YcbM	-0,68115992	0,623663648	0,031425022	0,708598306	1,634215575	0,018180864	1,128939611
BSU02570	YcbN	-0,56658757	0,675211991	0,014353508	0,731416901	1,660268877	0,000793548	1,167740434
BSU02580	YcbO	0,446119283	1,362370672	0,019810026	0,391618351	1,31186417	0,09629813	1,337117421
BSU02590	YcbP	0,53656872	1,450518524	0,000131634	-1,04294837	0,485334605	5,99E-05	0,967926565
**BSU02600**	**CwlJ**	**-2,04654813**	**0,242062561**	**1,69E-12**	**-1,88491864**	**0,27075903**	**1,29E-11**	**0,256410796**
BSU02610	YcbR	-0,90739819	0,533145718	0,000188073	-0,81903497	0,566820968	0,000113393	0,549983343
BSU02619	YczK	-1,98869377	0,251966918	0,022388369	-0,37257552	0,772402361	0,511549742	0,51218464
BSU02620	PhoD	-1,78629994	0,289914634	0,047325736	0,695638183	1,619600713	0,366063931	0,954757673
BSU02630	TatAD	-1,28064966	0,411610114	0,094757133	-0,14771686	0,902677865	0,080085152	0,65714399
BSU02640	TatCD	1,697355849	3,2430603	1,02E-07	-0,83229548	0,561634911	0,000414439	1,902347605
BSU02650	Pcp	-0,53600074	0,689680106	0,010157923	-0,40250598	0,756543015	0,010834848	0,72311156
BSU02660	YcbU	-0,19941809	0,870901769	0,125477887	0,078626763	1,056012391	0,364103403	0,96345708
BSU02670	LmrB	-1,73649808	0,300097232	4,08E-08	-0,37154003	0,772956949	0,085652625	0,536527091
BSU02680	LmrA	-1,78448966	0,290278643	1,03E-10	-0,889323	0,539867399	0,000234101	0,415073021
BSU02690	AnsZ	-1,83817799	0,279674769	2,89E-11	-0,94561387	0,519208579	2,90E-05	0,399441674
BSU02700	Lip	0,404045404	1,323213083	0,014179847	0,57149087	1,486058459	0,001435389	1,404635771
**BSU02710**	**YczC**	**-2,37361566**	**0,192961421**	**2,57E-15**	**-2,23046228**	**0,213090432**	**4,02E-14**	**0,203025927**
BSU02720	YccF	-0,15017112	0,901143572	0,123399037	0,033178601	1,023264139	0,390626859	0,962203855
BSU02730	NatK	-1,0631991	0,47856968	1,78E-05	0,391580135	1,31182942	0,122514987	0,89519955
BSU02740	NatR	-0,83244135	0,561578126	0,00184271	-0,04027965	0,972466425	0,130492281	0,767022276

BSU02750	NatA	-1,7475991	0,297796954	0,00327769	0,90307731	1,870050603	0,007863744	1,083923779
BSU02760	NatB	-0,51092273	0,701773447	0,000241366	-0,08765489	0,94105119	0,041752601	0,821412318
BSU02770	YccK	-0,07819664	0,947240956	0,07402358	-0,387887	0,76424812	0,065339232	0,855744538
**BSU02780**	**YcdA**	**-2,18567984**	**0,219808664**	**1,86E-14**	**-1,89300448**	**0,269245758**	**3,43E-11**	**0,244527211**
BSU02790	YcdB	0,254736591	1,193117882	0,17605687	0,933561622	1,909985416	0,002186919	1,551551649
BSU02800	YcdC	-0,14535537	0,904156638	0,05765017	0,468018694	1,383208552	0,007177382	1,143682595
BSU02810	CwlK	0,390857507	1,311172505	0,087352098	-0,02939928	0,9798282	0,344925479	1,145500353
BSU02820	RapJ	1,243974321	2,368501066	2,58E-06	0,774960644	1,711143372	0,000614587	2,039822219
BSU02830	YcdF	1,051165312	2,072202958	3,39E-05	1,328950014	2,512197719	1,93E-05	2,292200339
BSU02840	YcdG	0,870825448	1,82870891	0,002015857	0,865769943	1,822311946	0,000560743	1,825510428
BSU02850	ZnuA	-0,2606076	0,834736289	0,152690238	-0,91295194	0,531097286	0,000214075	0,682916787
BSU02860	ZnuC	-0,85531887	0,552743145	0,001290492	-0,17035849	0,888621843	0,517908526	0,720682494
BSU02870	ZnuB	-0,7931416	0,577086063	0,000704228	-0,18090636	0,882148623	0,217419939	0,729617343
BSU02880	YceB	-0,79071594	0,578057158	4,71E-05	-1,54647927	0,342344498	1,23E-09	0,460200828
BSU02890	YceC	0,445383494	1,361676026	0,009933169	1,284888836	2,43663277	2,68E-08	1,899154398
BSU02900	YceD	0,351457863	1,275849239	0,11959732	1,464399797	2,759486442	6,38E-10	2,01766784
BSU02910	YceE	0,591777483	1,507102441	0,015668448	1,361717118	2,569908721	1,23E-09	2,038505581
BSU02920	YceF	0,82590321	1,772644468	1,24E-05	1,234533829	2,353053021	8,01E-08	2,062848744
BSU02930	YceG	-0,17748436	0,884243514	0,024844787	1,263099706	2,400108633	3,62E-07	1,642176073
BSU02940	YceH	0,040711932	1,028621299	0,002964698	0,584492316	1,499511219	0,003800818	1,264066259
BSU02950	NiaP	0,935771252	1,912912988	9,13E-08	0,052008606	1,036707285	0,321520585	1,474810137
BSU02960	YceJ	-0,64219968	0,640735274	0,350087181	-0,67893019	0,624628287	0,077213487	0,632681781
BSU02970	YceK	-0,80681881	0,571640956	0,002724113	-1,17120404	0,444050592	1,37E-06	0,507845774
BSU02980	OpuAA	-0,79865903	0,574883275	0,001736555	0,508883287	1,422948341	0,003690478	0,998915808
BSU02990	OpuAB	-0,7986942	0,574869263	0,000163162	0,280950403	1,214995022	0,050940541	0,894932143
BSU03000	OpuAC	-1,07479558	0,474738323	5,20E-05	0,351119868	1,275550368	0,057439903	0,875144345
BSU03010	AmhX	0,782065255	1,71959075	0,000406928	0,492960574	1,407329915	0,002507246	1,563460332
**BSU03020**	**YcgA**	**2,065942044**	**4,187072921**	**2,99E-13**	**1,120902521**	**2,174829829**	**4,96E-08**	**3,180951375**
BSU03030	YcgB	0,603615783	1,519520126	0,001662424	1,110741843	2,15956665	1,48E-07	1,839543388

BSU03040	AmyE	-1,79566985	0,288037818	0,05315514	0,012921895	1,008997007	0,699324572	0,648517413
BSU03050	Ldh	-0,23957896	0,846992467	0,054866583	0,154270005	1,112858376	0,19158367	0,979925422
BSU03060	LctP	0,245172575	1,18523454	0,629621445	0,446610192	1,362834327	0,339236111	1,274034434
BSU03070	Mdr	-1,02011977	0,493075415	1,66E-05	-0,5391582	0,688172334	0,006321756	0,590623875
BSU03080	YcgE	0,012048956	1,008386672	0,63714422	0,326320958	1,253811926	0,128715466	1,131099299
BSU03090	YcgF	1,259270324	2,393746412	2,33E-08	0,411522122	1,33008839	0,043065238	1,861917401
BSU03100	YcgG	0,37178271	1,293950753	0,045720963	-0,08443001	0,943157092	0,809339823	1,118553923
**BSU03110**	**YcgH**	**-2,94930438**	**0,129470527**	**5,88E-18**	**-1,71794801**	**0,303980775**	**1,55E-10**	**0,216725651**
**BSU03120**	**YcgI**	**-2,84525574**	**0,139153033**	**1,82E-15**	**-1,11213109**	**0,462610178**	**5,78E-06**	**0,300881605**
BSU03130	NadE	-0,55905192	0,678748062	0,022269418	-0,20176691	0,869485028	0,266330415	0,774116545
BSU03140	TmrB	-0,25314613	0,839064642	0,244505801	-0,88433219	0,54173823	0,000887445	0,690401436
BSU03150	AroK	1,082023819	2,117003742	1,05E-05	-0,02659093	0,981737393	0,630792454	1,549370567
BSU03160	YcgJ	0,596988256	1,512555684	0,06318288	-0,59113811	0,663819028	0,044777802	1,088187356
BSU03170	YcgK	0,653052626	1,572491944	0,007718858	0,091684692	1,065613816	0,616456505	1,31905288
BSU03180	Cah	-1,0903362	0,469651916	0,000126986	-0,53094976	0,692098958	0,033629416	0,580875437
**BSU03190**	**YcgL**	**-2,65402966**	**0,158875695**	**1,16E-16**	**-1,34353483**	**0,394053978**	**4,21E-08**	**0,276464837**
**BSU03200**	**PutB**	**-2,67680443**	**0,156387333**	**1,51E-16**	**-1,0140935**	**0,495139347**	**1,39E-05**	**0,32576334**
BSU03210	PutC	-2,36533401	0,194072282	1,72E-14	-0,67250532	0,627416199	0,003311059	0,410744241
BSU03220	PutP	-0,69190061	0,619037792	0,002256726	-0,39434559	0,760834416	0,023054704	0,689936104
BSU03230	PutR	-0,44359886	0,735298089	0,00754078	-0,08870198	0,940368434	0,676507996	0,837833262
BSU03240	YcgQ	0,102451073	1,073595904	0,104288238	0,259405099	1,196985022	0,28054805	1,135290463
BSU03250	YcgR	-0,33691814	0,791730788	0,137493401	0,12833777	1,093033615	0,619909126	0,942382201
BSU03260	YcgS	-1,31141566	0,402925311	2,09E-05	-0,66004039	0,63286058	0,00089872	0,517892945
BSU03270	YcgT	0,300882684	1,231897894	0,000198881	0,572841788	1,487450634	0,001509821	1,359674264
BSU03280	NasF	0,759728923	1,693172455	0,000258237	-0,49101689	0,7115234	0,01971693	1,202347927
BSU03290	NasE	-0,16451009	0,892231449	0,18629464	-0,30161525	0,811343504	0,182872798	0,851787477
BSU03300	NasD	-0,21007701	0,864491084	0,120213895	-0,34175642	0,789080056	0,028923974	0,82678557
BSU03310	NasC	-1,5945488	0,331125771	6,30E-06	-0,24209175	0,845518517	0,214612117	0,588322144
BSU03320	NasB	-1,39876698	0,379253137	2,55E-06	-0,48028335	0,716836822	0,089980037	0,548044979

BSU03330	NasA	-0,10192924	0,931786129	0,327152869	-0,00965959	0,993326847	0,567338023	0,962556488
BSU03340	FolE2	-0,21993887	0,858601816	0,741517521	0,080935539	1,057703703	0,776822288	0,95815276
BSU03350	YciB	0,818870956	1,764024938	4,92E-06	0,017073312	1,011904621	0,741874227	1,387964779
BSU03359	YczL	1,631798806	3,098991517	3,51E-08	0,080935539	1,057703703	0,35831432	2,07834761
BSU03360	YciC	-0,34233064	0,788766051	0,736958637	0,092514978	1,066227264	0,630959234	0,927496658
BSU03370	YckA	-0,903729	0,53450339	0,000906266	-0,1565297	0,897180581	0,444933372	0,715841985
BSU03380	YckB	-1,33751388	0,39570196	1,27E-07	0,003058635	1,002122333	0,785828324	0,698912147
BSU03390	YckC	-0,97945098	0,507172709	5,97E-05	-1,14398031	0,452509408	5,63E-06	0,479841059
**BSU03400**	**YckD**	**-2,02875739**	**0,245066063**	**1,32E-13**	**-2,08310698**	**0,236005603**	**1,22E-13**	**0,240535833**
BSU03410	YckE	-1,27220937	0,414025242	6,03E-07	-0,864825	0,549113011	4,62E-05	0,481569127
BSU03420	Nin	-1,21465684	0,430875557	2,43E-07	-0,49895122	0,707621005	0,029610687	0,569248281
BSU03430	NucA	-1,32664525	0,398694263	2,42E-08	-0,96769999	0,511320583	0,000160136	0,455007423
BSU03440	TlpC	-0,20721905	0,866205328	0,06356566	-0,04642601	0,968332208	0,687452266	0,917268768
BSU03450	HxlB	-0,39437521	0,760818797	0,116392777	-1,03104771	0,489354644	2,73E-06	0,62508672
BSU03460	HxlA	-1,2937969	0,407876164	3,24E-07	-0,68476377	0,622107684	0,000994216	0,514991924
BSU03470	HxlR	-0,28914689	0,818385854	0,043728578	-0,07107637	0,951927514	0,549856695	0,885156684
BSU03480	SrfAA	0,131171442	1,095182608	0,511979367	1,121573111	2,175840963	7,47E-11	1,635511786
BSU03490	SrfAB	0,428582312	1,345910346	0,013825783	0,799562373	1,740573061	4,06E-09	1,543241704
BSU03500	ComS	-0,06830114	0,953760449	0,413656263	0,932540698	1,908634291	1,82E-09	1,43119737
BSU03510	SrfAC	0,771065124	1,706529229	2,09E-05	0,999212037	1,998907949	3,93E-10	1,852718589
BSU03520	SrfAD	1,028956562	2,040547879	3,86E-05	0,589929367	1,505173053	3,68E-05	1,772860466
BSU03530	YcxA	-0,1077236	0,928051264	0,383276766	-0,28347293	0,821610809	0,312002761	0,874831037
BSU03540	YcxB	-0,28031716	0,823409979	0,021858313	-0,60794913	0,656128764	0,00779378	0,739769372
BSU03550	YcxC	-1,17668185	0,442367759	2,18E-05	-0,63627257	0,643373059	0,001965965	0,542870409
BSU03560	YcxD	0,039856337	1,028011453	0,444084068	0,458544855	1,374155105	0,101015719	1,201083279
BSU03580	YczE	-0,98957344	0,50362666	7,20E-05	-0,71525365	0,609098032	0,002975633	0,556362346
**BSU03590**	**TcyC**	**-2,00200382**	**0,249653006**	**1,97E-13**	**-1,11242924**	**0,462514584**	**1,23E-06**	**0,356083795**
**BSU03600**	**TcyB**	**-1,84374264**	**0,278598107**	**2,03E-11**	**-1,1955097**	**0,436632161**	**5,32E-07**	**0,357615134**
**BSU03610**	**TcyA**	**-2,45977539**	**0,181774863**	**3,12E-15**	**-1,66122458**	**0,316170664**	**2,43E-10**	**0,248972764**

BSU03620	BsdA	-1,07460584	0,474800763	0,012574268	-0,1260645	0,916327675	0,323012829	0,695564219
BSU03630	BsdB	-1,01066515	0,496317368	0,004017785	0,283063693	1,216776076	0,039668879	0,856546722
BSU03640	BsdC	-1,06952009	0,476477473	0,005717916	0,158412509	1,116058391	0,434551034	0,796267932
BSU03651	BsdD	0,528730734	1,442659402	0,001281515	-0,23597176	0,849112867	0,176808752	1,145886134
BSU03652	YclD	-0,67751046	0,625243276	0,20522778	-0,24940135	0,841245418	0,412109034	0,733244347
BSU03660	YclE	-0,30440181	0,809777908	0,302147401	0,087963667	1,062868906	0,535327973	0,936323407
BSU03670	YclF	0,235375198	1,177212852	0,055106279	-0,28181615	0,822554887	0,054276113	0,999883869
**BSU03680**	**YclG**	**-2,34815154**	**0,196397498**	**1,76E-09**	**-1,24382507**	**0,422251639**	**1,01E-05**	**0,309324569**
**BSU03690**	**YczF**	**-3,3372235**	**0,098945403**	**1,63E-17**	**-1,24909468**	**0,420712129**	**1,34E-07**	**0,259828766**
**BSU03700**	**GerKA**	**-3,1864836**	**0,109843119**	**1,06E-17**	**-0,94071876**	**0,520973264**	**1,93E-05**	**0,315408191**
**BSU03710**	**GerKC**	**-3,1181815**	**0,115168534**	**8,23E-18**	**-0,85984352**	**0,551012321**	**0,000989566**	**0,333090428**
**BSU03720**	**GerKB**	**-2,53309731**	**0,172767372**	**3,59E-11**	**-0,89613389**	**0,537324719**	**0,000134687**	**0,355046046**
**BSU03730**	**YclH**	**-2,3567714**	**0,195227556**	**0,000421686**	**-1,08011468**	**0,472991224**	**0,074135593**	**0,33410939**
BSU03740	YclI	-1,78325284	0,290527607	0,001916665	-0,95120344	0,517200853	0,083168038	0,40386423
BSU03750	YclJ	0,025840885	1,01807291	0,046296728	0,582347176	1,497283256	0,01584433	1,257678083
BSU03760	YclK	0,017494231	1,012199896	0,118091852	0,37474745	1,296612558	0,006869147	1,154406227
BSU03770	RapC	-0,78083751	0,582028816	7,96E-06	-0,65109925	0,636794926	0,050914494	0,609411871
BSU03780	PhrC	-0,09372429	0,937100515	0,185898341	-0,94665133	0,518835346	0,000661909	0,72796793
BSU03789	YczN	0,575057311	1,489736642	0,004380152	-0,65653169	0,6344016	0,003541253	1,062069121
BSU03790	YclM	1,206351577	2,307533475	3,00E-06	0,583941434	1,498938752	0,001016545	1,903236114
BSU03800	YclN	-0,17701293	0,884532511	0,0316493	0,928897375	1,903820386	2,26E-05	1,394176448
BSU03810	YclO	-0,20566391	0,867139548	0,033688336	0,564669055	1,479048186	0,00351538	1,173093867
BSU03820	YclP	-0,33394582	0,79336364	0,225310822	0,704080533	1,629106062	0,000257118	1,211234851
BSU03830	YclQ	-0,04786994	0,967363532	0,090573085	0,155007656	1,113427527	0,260187026	1,040395529
**BSU03840**	**YcnB**	**-1,89876378**	**0,268173059**	**7,92E-09**	**-1,09469351**	**0,468235587**	**2,85E-06**	**0,368204323**
**BSU03850**	**YcnC**	**-2,23441989**	**0,212506682**	**5,61E-10**	**-1,33600681**	**0,396115535**	**1,72E-07**	**0,304311108**
**BSU03860**	**YcnD**	**-2,38703288**	**0,191175178**	**9,33E-14**	**-1,64435758**	**0,319888808**	**1,03E-10**	**0,255531993**
**BSU03870**	**YcnE**	**-2,11371029**	**0,231052036**	**4,84E-12**	**-1,60166754**	**0,32949591**	**4,55E-10**	**0,280273973**
BSU03880	YczG	-0,04872943	0,966787395	0,223146426	-1,13492458	0,455358722	9,46E-07	0,711073058

BSU03890	GabR	-0,79390179	0,576782066	0,001226238	0,107905163	1,077662302	0,341037656	0,827222184
BSU03900	GabT	-1,83758611	0,279789532	4,10E-06	-0,08739874	0,94121829	0,33571953	0,610503911
BSU03910	GabD	0,411531122	1,330096688	0,002367071	-0,1125898	0,924926227	0,445856303	1,127511458
**BSU03920**	**GlcU**	**-3,43417923**	**0,092514337**	**1,09E-19**	**-1,81431365**	**0,284339484**	**2,00E-10**	**0,188426911**
**BSU03930**	**Gdh**	**-3,00536783**	**0,124535777**	**1,79E-18**	**-1,63145739**	**0,322761994**	**6,78E-10**	**0,223648886**
BSU03940	YcnI	0,847717222	1,799651081	3,66E-05	0,223510227	1,167570955	0,292065313	1,483611018
BSU03950	YcnJ	0,728145822	1,656508747	0,000506965	0,380589899	1,301874066	0,016138708	1,479191407
BSU03960	YcnK	-0,15671336	0,897066377	0,081893195	0,400718638	1,32016535	0,015284463	1,108615864
BSU03970	YcnL	-0,8886885	0,540104883	0,00198072	-1,53976903	0,343940513	5,18E-09	0,442022698
BSU03981	MtlA	-0,19311695	0,874713858	0,002535398	0,458679984	1,374283821	0,045113268	1,124498839
BSU03982	MtlF	-0,45872628	0,727628379	0,0620021	0,573111961	1,487729213	0,062079222	1,107678796
BSU03990	MtlD	0,397710642	1,317415694	0,026116224	0,687853954	1,610885502	0,087749993	1,464150598
BSU04000	YcsA	0,683219118	1,605718637	0,003662906	1,134863909	2,195978469	6,25E-06	1,900848553
BSU04010	SipU	-0,28524616	0,82060158	0,111831491	0,407638141	1,326512381	0,030572211	1,07355698
BSU04020	YczH	0,151083485	1,110403088	0,181126764	-0,72070258	0,606801864	0,003781863	0,858602476
BSU04030	YcsD	1,471440189	2,772985725	3,95E-07	-0,48119355	0,716384708	0,045649378	1,744685216
BSU04039	YczO	2,040006097	4,112472686	4,31E-12	-0,49836054	0,707910783	0,023441542	2,410191734
BSU04040	YcsE	-0,3798427	0,768521381	0,040779085	0,272660301	1,208033365	0,193481845	0,988277373
**BSU04050**	**YcsF**	**-2,81546995**	**0,14205584**	**3,11E-12**	**-1,09955312**	**0,466661024**	**5,97E-05**	**0,304358432**
**BSU04060**	**YcsG**	**-2,3253869**	**0,199521082**	**1,85E-14**	**-1,0911938**	**0,469372818**	**3,25E-05**	**0,33444695**
BSU04070	YcsI	-3,23957323	0,105874479	2,18E-17	-0,58626242	0,666066249	0,003829334	0,385970364
BSU04080	KipI	-1,35603441	0,390654623	1,06E-09	-0,67425757	0,62665462	0,001965267	0,508654621
BSU04090	KipA	-0,89342884	0,538333144	1,58E-06	-0,15984814	0,895119286	0,143688749	0,716726215
BSU04100	KipR	-1,38363478	0,383252	2,47E-07	-0,77883116	0,582838807	0,000792358	0,483045403
BSU04110	LipC	-0,92900798	0,525219368	0,000270953	-1,80984835	0,285220909	6,77E-11	0,405220139
BSU04120	YczI	0,101874911	1,073167233	0,284708307	-0,95591625	0,515514085	3,17E-05	0,794340659
BSU04130	YczJ	0,349057171	1,27372795	0,046654831	0,237886544	1,179263849	0,191766959	1,226495899
BSU04140	PbpC	-0,32010659	0,801010697	0,189635291	-0,00189832	0,998685048	0,57259741	0,899847873
BSU04150	YcsN	0,474204637	1,389152164	0,005101194	0,628351965	1,545798171	0,000881678	1,467475168

BSU04160	MtlR	0,40725584	1,326160914	3,46E-05	0,459689661	1,375245956	0,026892757	1,350703435
**BSU04170**	**YdaB**	**-2,24804838**	**0,21050868**	**4,28E-11**	**-1,34487889**	**0,393687036**	**1,47E-07**	**0,302097858**
BSU04180	YdaC	0,596834332	1,512394316	0,729729509	0,080935539	1,057703703	1	1,28504901
BSU04190	YdaD	1,877027997	3,67317593	2,58E-11	-0,14441517	0,904746066	0,517816849	2,288960998
BSU04200	YdaE	0,264577069	1,201283838	0,461029964	0,270400133	1,206142306	0,158630368	1,203713072
BSU04210	YdaF	-0,00606056	0,995807951	0,004696916	0,160864125	1,117956555	0,330742912	1,056882253
BSU04220	YdaG	-0,29544173	0,814822808	0,266084906	-0,14114953	0,906796337	0,668391108	0,860809573
**BSU04230**	**YdaH**	**2,412471287**	**5,323855031**	**9,91E-11**	**1,092103278**	**2,131846079**	**0,002051292**	**3,727850555**
BSU04240	YdzA	-0,53376086	0,690751714	0,159074631	-1,94664438	0,259418922	8,01E-09	0,475085318
BSU04250	LrpC	0,626907031	1,544250749	0,000121304	-0,02944114	0,979799772	0,158959929	1,262025261
BSU04260	TopB	0,084868957	1,060591401	0,292029957	0,080935539	1,057703703	1	1,059147552
BSU04270	YdaJ	0,078214489	1,055710661	1	0,080935539	1,057703703	0,929249138	1,056707182
BSU04280	YdaK	-0,59644656	0,661380968	0,73165949	0,080935539	1,057703703	0,972294228	0,859542336
BSU04290	YdaL	0,360322722	1,283713024	0,331861319	0,101305129	1,072743477	0,487579938	1,178228251
BSU04300	YdaM	0,059383361	1,042020284	1	0,080935539	1,057703703	0,88310496	1,049861994
BSU04310	YdaN	-0,01056425	0,992704162	0,97644879	0,080935539	1,057703703	0,944621536	1,025203933
BSU04320	YdaO	0,973864964	1,964095343	0,017736687	-0,06172425	0,958118332	0,579287172	1,461106838
BSU04330	MutT	0,064757112	1,045908838	0,944630907	0,080935539	1,057703703	0,886624805	1,051806271
BSU04340	YdaP	-0,00241597	0,998326781	0,995746954	0,080935539	1,057703703	0,706225183	1,028015242
BSU04359	YdzK	0,159482064	1,116886097	1	0,080935539	1,057703703	0,96984642	1,0872949
BSU04360	MntH	0,077261945	1,055013855	0,668138886	0,080935539	1,057703703	1	1,056358779
BSU04370	YdaS	3,237747915	9,433204313	1,17E-18	0,424435494	1,342047276	0,049675192	5,387625794
BSU04380	YdaT	1,182690962	2,269997899	1,65E-05	0,808961931	1,7519504	0,007834282	2,010974149
BSU04390	YdbA	-1,01735688	0,494020605	0,000307796	-0,44025786	0,73700287	0,033475564	0,615511738
BSU04400	GsiB	2,143081422	4,417044668	4,53E-14	0,151289283	1,110561496	0,297782463	2,763803082
BSU04410	YdbB	0,578925902	1,493736736	0,014173034	-0,74813087	0,595374414	0,002689485	1,044555575
BSU04420	YdbC	0,12593729	1,091216444	0,370267954	-0,32782582	0,796736284	0,132854578	0,943976364
BSU04430	YdbD	1,487544051	2,804112151	1,57E-10	0,645003212	1,563742767	0,000570022	2,183927459
BSU04440	DctB	-0,33986519	0,790115138	0,021033011	0,496497215	1,410784094	0,019816667	1,100449616

BSU04450	DctS	-0,34248563	0,788681319	0,269528414	1,055549362	2,078509529	2,89E-05	1,433595424
BSU04460	DctR	0,773164462	1,709014292	0,026678463	1,016108646	2,022456462	0,000233623	1,865735377
BSU04470	DctP	0,361859453	1,285081139	0,110668217	0,558264425	1,472496725	0,003970269	1,378788932
BSU04480	YdbI	-0,02859599	0,980373923	0,401364391	-0,93938742	0,521454248	0,000305355	0,750914085
BSU04490	YdbJ	-0,68940866	0,620107972	0,007465787	0,320568297	1,24882238	0,18475625	0,934465176
BSU04500	YdbK	-0,36910828	0,774260916	0,032147806	0,251547655	1,190483525	0,047416508	0,98237222
BSU04510	YdbL	0,625933455	1,543208992	0,001853518	-1,13933672	0,453968243	1,13E-06	0,998588617
BSU04520	YdbM	0,476371105	1,391239795	0,023229138	-0,57399883	0,671752259	0,029379663	1,031496027
BSU04530	FbpB	-0,08049808	0,945731081	0,438379596	0,207923095	1,155024213	0,194996026	1,050377647
BSU04540	YdbO	-1,00140192	0,499514366	3,14E-08	-0,67402517	0,626755576	0,004051391	0,563134971
BSU04550	YdbP	0,040396054	1,028396107	0,370897365	-0,25863647	0,835877557	0,028009506	0,932136832
BSU04560	Ddl	-1,14396262	0,452514956	6,34E-06	-0,62931478	0,646483397	0,016269914	0,549499176
BSU04570	MurF	-1,22630329	0,427411227	2,51E-06	-0,65876824	0,633418872	0,004901415	0,53041505
BSU04580	CshA	0,322277947	1,250303161	0,12727878	-0,7183299	0,60780064	0,000673514	0,9290519
BSU04590	YdbS	-0,16705132	0,89066122	0,319529473	-0,05598128	0,961939939	0,694637905	0,92630058
BSU04600	YdbT	-0,29066093	0,817527448	0,085482249	-0,73201672	0,602061713	0,007789083	0,709794581
BSU04610	YdcA	1,682024096	3,208778258	3,01E-11	-0,19268687	0,874974652	0,531030291	2,041876455
BSU04620	AcpS	0,194845336	1,144601447	0,319669962	0,259384994	1,196968341	0,235088542	1,170784894
BSU04630	YdcC	0,631375037	1,549040686	0,004326212	0,458246178	1,373870647	0,001960102	1,461455667
BSU04640	Alr	-0,44396667	0,735110652	0,099856475	0,144562854	1,105395661	0,305556347	0,920253156
BSU04650	NdoAI	-0,32477653	0,798422051	0,061304793	-0,39357402	0,761241426	0,484620832	0,779831739
BSU04660	NdoA	-0,34390852	0,787903846	0,136600956	-0,56037775	0,678124583	0,020660164	0,733014214
BSU04670	RsbR	-0,01891128	0,986977237	0,547900444	0,744815545	1,675760002	0,000145645	1,331368619
BSU04680	RsbS	0,939244873	1,917524316	1,07E-05	0,585848872	1,500921862	0,001150189	1,709223089
BSU04690	RsbT	0,487712678	1,402219962	0,001972079	0,963259953	1,94971054	3,76E-06	1,675965251
BSU04700	RsbU	0,247937378	1,187508119	0,100307488	1,221580005	2,332019748	3,16E-08	1,759763934
**BSU04710**	**RsbV**	**1,656764277**	**3,153085469**	**5,82E-11**	**1,038888774**	**2,054644467**	**5,32E-07**	**2,603864968**
BSU04720	RsbW	1,387416527	2,616097893	1,35E-09	1,074409596	2,105860099	8,52E-07	2,360978996
BSU04730	SigB	1,207479698	2,309338566	1,15E-07	1,105345276	2,151503632	7,76E-07	2,230421099

BSU04740	RsbX	1,574074876	2,977445036	1,52E-12	0,718893283	1,645918936	0,00019671	2,311681986
BSU04750	YdcF	1,130455701	2,189278816	3,36E-05	-0,01211129	0,991640233	0,262041261	1,590459524
BSU04760	YdcG	1,224955903	2,33748305	2,73E-05	0,293029026	1,225209982	0,262655298	1,781346516
BSU04770	YdcH	1,013455123	2,018740013	0,00243718	0,021065778	1,01470881	0,671613988	1,516724412
BSU04780	YdcI	-0,39628988	0,759809744	0,00523388	-1,37100305	0,386622352	7,72E-07	0,573216048
BSU04790	YdcK	0,458060124	1,373693481	0,005949292	0,241227689	1,181998077	0,161878191	1,277845779
BSU04800	Int	1,818113249	3,526197421	2,24E-10	0,043351036	1,030504665	0,506497923	2,278351043
BSU04810	ImmA	0,152255658	1,111305644	1	0,080935539	1,057703703	1	1,084504674
BSU04820	ImmR	0,99966604	1,999537086	0,000184552	-0,07548101	0,949025651	0,477185934	1,474281368
BSU04830	Xis	0,803802991	1,74569678	0,319600814	0,080935539	1,057703703	0,895043752	1,401700242
BSU04839	YdzL	0,137350077	1,099883009	0,996157421	0,080935539	1,057703703	0,97201296	1,078793356
BSU04840	YdcO	1,487681951	2,804380195	9,96E-10	0,037232524	1,026143519	0,642284123	1,915261857
BSU04850	YdcP	2,116055805	4,33507155	6,22E-11	0,078159132	1,055670154	0,386903436	2,695370852
BSU04860	YdcQ	1,242534636	2,366138685	0,000288719	-0,35242915	0,783264162	0,055298527	1,574701423
BSU04870	NicK	0,083991422	1,059946481	1	0,080935539	1,057703703	1	1,058825092
BSU04880	YdcS	0,290081696	1,222709515	0,933551456	0,080935539	1,057703703	1	1,140206609
BSU04890	YdcT	0,543571053	1,457575943	0,001050964	-0,50550554	0,7044135	0,235758846	1,080994721
BSU04900	YddA	0,838568918	1,788275382	0,138131356	-0,29409547	0,815583518	0,443045201	1,30192945
BSU04910	YddB	1,136143559	2,197927135	0,003196015	-0,06265656	0,95749937	0,613970762	1,577713253
BSU04920	YddC	1,307451817	2,475039959	0,000597566	-0,12796981	0,915118317	0,312242289	1,695079138
BSU04930	YddD	0,124204442	1,08990655	6,26E-07	0,080935539	1,057703703	0,842285573	1,073805127
BSU04940	ConE	0,305435848	1,235791922	0,671058327	-0,91785424	0,52929567	0,347721862	0,882543796
BSU04950	YddF	0,382175485	1,30330567	0,170053588	0,080935539	1,057703703	0,270669456	1,180504686
BSU04960	YddG	0,114144773	1,08233325	0,682553093	-0,3626172	0,777752375	0,947041791	0,930042813
BSU04970	CwlT	1,144735069	2,211055239	1,55E-05	-0,04772948	0,967457722	0,757903031	1,58925648
BSU04980	YddI	0,147612197	1,107734544	1	0,080935539	1,057703703	1	1,082719124
BSU04990	YddJ	2,029822423	4,08354584	2,43E-13	-0,2155264	0,861231864	0,429343202	2,472388852
BSU05000	YddK	0,788876701	1,727728709	0,001578983	0,021157952	1,014773642	0,351234663	1,371251175
BSU05010	RapI	0,089361186	1,063898992	1	0,080935539	1,057703703	0,753564845	1,060801348

BSU05020	PhrI	0,721383642	1,648762551	1	0,080935539	1,057703703	0,778006632	1,353233127
BSU05030	YddM	0,256508974	1,194584555	0,133935389	-0,55782372	0,679326145	0,4294486	0,93695535
BSU05040	YddN	-0,54080341	0,687388008	0,007292198	-0,0202287	0,986076377	0,725588636	0,836732192
BSU05050	LrpA	-1,32120927	0,400199351	3,48E-06	-0,00124894	0,999134679	0,782473169	0,699667015
BSU05060	LrpB	-0,16673352	0,890857436	0,683743278	0,178866862	1,131994432	0,38366126	1,011425934
BSU05070	YddQ	-1,4691683	0,361190462	4,35E-06	-0,51940418	0,697659901	0,01394247	0,529425182
BSU05080	YddR	-1,33097348	0,397499933	0,11335394	-0,32819311	0,79653347	0,315034719	0,597016702
BSU05090	YddS	1,102715696	2,147585688	0,123300555	0,515698908	1,429686578	0,010765016	1,788636133
BSU05099	YdzM	0,989913311	1,986065649	9,23E-05	0,959072932	1,944060251	0,000241899	1,96506295
BSU05100	YddT	0,760675131	1,694283304	0,000637109	0,677817816	1,599718231	0,005021596	1,647000768
BSU05109	YdzN	-0,46176152	0,726099157	0,440590645	-0,17850324	0,883619252	0,175174791	0,804859204
BSU05110	YdeA	0,655550743	1,575217171	0,074748592	0,792285194	1,731815445	0,0039354	1,653516308
BSU05120	CspC	2,292889784	4,900366947	4,43E-15	-0,38012379	0,768371659	0,096371307	2,834369303
BSU05130	YdeB	-0,01039519	0,9928205	0,089590573	0,103939135	1,074703832	0,213871657	1,033762166
BSU05140	YdzE	1,005056722	2,007022405	7,72E-05	-0,91031057	0,53207054	0,000305667	1,269546472
BSU05150	YdeC	-1,74054617	0,299256363	6,72E-07	-0,23930006	0,847156221	0,007691228	0,573206292
BSU05160	YdeD	-0,67385643	0,626828887	0,067897096	-0,25573489	0,837560382	0,102026576	0,732194634
BSU05170	YdeE	-1,69957315	0,307877182	2,52E-10	-0,75548574	0,592346915	0,000545707	0,450112049
BSU05180	YdeF	-0,69599718	0,617282509	0,060079694	-0,21755798	0,860019944	0,455530494	0,738651227
BSU05190	YdeG	-0,14711273	0,903055947	0,071259868	0,096905432	1,069476982	0,115477451	0,986266464
BSU05200	YdeH	0,32100705	1,24920223	0,027785617	-0,20186678	0,869424843	0,502647507	1,059313536
BSU05210	YdeI	-0,34795881	0,78569495	0,043657998	0,065236649	1,046256546	0,149708755	0,915975748
BSU05220	YdeJ	-0,00706662	0,995113769	0,042188538	0,679228873	1,601283633	0,006050161	1,298198701
BSU05230	YdeK	-0,40066201	0,757510607	0,102587738	-1,15110578	0,450279974	6,54E-06	0,603895291
BSU05240	YdeL	-1,4671151	0,361704862	4,52E-06	-0,15570874	0,897691265	0,030466946	0,629698064
BSU05250	YdeM	-0,05828488	0,960405203	0,093813315	0,390159129	1,310537948	0,080891205	1,135471576
BSU05260	YdeN	0,649673073	1,568812649	0,001654947	0,07128434	1,050651596	0,65232137	1,309732123
BSU05270	YdzF	1,180060774	2,265863218	6,95E-05	0,022836218	1,0159548	0,337216548	1,640909009
BSU05280	YdeO	0,684973304	1,607672232	0,000395203	-0,19912003	0,871081717	0,325010614	1,239376974

BSU05290	YdeP	0,879154011	1,839296431	0,000289777	-0,12566232	0,916583155	0,037042382	1,377939793
BSU05300	YdeQ	-0,00451814	0,996873162	0,406066144	-0,00590318	0,995916587	0,397147995	0,996394874
BSU05310	YdeR	-0,62878715	0,646719872	0,127438931	-1,08035059	0,472913885	0,058515007	0,559816879
BSU05320	YdeS	0,78002455	1,717160094	0,000223603	-0,36294426	0,777576079	0,006584958	1,247368086
BSU05329	YdzO	-0,35740853	0,780565426	0,052530097	0,150113288	1,109656605	0,243300125	0,945111016
BSU05330	AseR	-1,14847096	0,451103079	0,000685211	0,846203914	1,797764335	0,003553751	1,124433707
BSU05340	AseA	-0,31034989	0,80644615	0,040849996	0,750592481	1,682483645	0,002835988	1,244464897
BSU05350	YdfB	0,082098946	1,058556993	0,081758117	0,161573373	1,118506293	0,350603449	1,088531643
BSU05360	YdfC	0,070490414	1,050073573	0,141273739	0,080935539	1,057703703	0,922377019	1,053888638
BSU05370	YdfD	0,013111137	1,009129368	5,51E-06	-0,06062689	0,958847386	0,593962483	0,983988377
BSU05380	YdfE	-1,00891507	0,496919801	0,35423503	0,010534003	1,007328336	0,673340086	0,752124069
BSU05390	YdfF	0,227603516	1,170888351	0,06531946	0,305138693	1,235537409	0,258014696	1,20321288
BSU05400	YdfG	1,051613049	2,072846161	0,000200162	0,457810759	1,373456063	0,00563382	1,723151112
BSU05408	YdzP	-1,11788113	0,460770058	0,141817028	0,080935539	1,057703703	0,81422378	0,75923688
BSU05409	YdzQ	-1,62579642	0,324030962	0,109253198	0,579456437	1,494286141	0,344302304	0,909158552
BSU05410	YdfH	-1,33439377	0,39655867	4,47E-06	0,287594151	1,220603093	0,153097163	0,808580882
BSU05420	YdfI	-0,68370356	0,622565027	0,049121776	-0,19252721	0,87507149	0,310761493	0,748818258
BSU05430	YdfJ	-1,37686296	0,385055161	2,96E-05	1,446011716	2,724538194	1,45E-05	1,554796678
BSU05440	Nap	0,403741896	1,32293474	0,14497636	0,300444594	1,231523873	0,332238811	1,277229307
BSU05450	YdfK	-0,95841168	0,514623172	0,244932166	0,787851572	1,726501482	0,041781886	1,120562327
BSU05460	YdfL	-0,18695462	0,878458098	0,181854553	0,157953587	1,115703428	0,425213402	0,997080763
BSU05470	YdfM	0,298932519	1,2302338	0,03324422	-0,32663102	0,79739639	0,217031608	1,013815095
BSU05480	MhqN	-0,38689426	0,764774188	0,018373948	0,618258481	1,535021094	0,029556563	1,149897641
BSU05490	MhqO	-0,56760781	0,674734664	0,006219389	0,291361531	1,223794679	0,126310281	0,949264671
BSU05500	MhqP	-1,6808829	0,311891707	5,97E-06	-0,59117785	0,663800742	0,005526848	0,487846225
BSU05510	YdfQ	-1,52578305	0,347291004	0,033341452	-0,86799418	0,547908094	0,138998841	0,447599549
BSU05520	YdzH	0,178584733	1,131773084	0,469750426	-0,23284313	0,85095625	0,493369188	0,991364667
**BSU05529**	**YdzR**	**-2,52414437**	**0,17384285**	**2,65E-15**	**-1,76938254**	**0,293334254**	**1,65E-10**	**0,233588552**
**BSU05530**	**YdfR**	**-3,17732694**	**0,110542501**	**1,06E-17**	**-1,86511774**	**0,274500801**	**3,96E-12**	**0,192521651**

**BSU05540**	**YdfS**	**-2,34723523**	**0,196522278**	**6,97E-11**	**-1,13723022**	**0,454631571**	**3,09E-06**	**0,325576925**
**BSU05550**	**CotP**	**-3,35964636**	**0,097419449**	**1,29E-18**	**-2,15772776**	**0,224108961**	**1,05E-13**	**0,160764205**
**BSU05560**	**YdgA**	**-3,26579958**	**0,103967205**	**1,79E-18**	**-2,72358999**	**0,151397156**	**2,88E-16**	**0,12768218**
**BSU05570**	**YdgB**	**-2,94409249**	**0,129939099**	**1,40E-17**	**-2,48704236**	**0,178371576**	**8,91E-16**	**0,154155337**
BSU05580	YdgC	-0,06541911	0,955667652	0,520883162	0,43213257	1,34922651	0,04735454	1,152447081
BSU05590	YdgD	0,27237547	1,207794886	0,111193025	0,310232284	1,239907317	0,098183485	1,223851102
BSU05600	YdgE	0,589798606	1,505036636	0,007940514	0,371533439	1,293727201	0,042464028	1,399381918
BSU05610	VmlR	-0,86434839	0,549294446	0,003470633	-0,61618414	0,6523942	0,000343734	0,600844323
BSU05620	YdgF	1,234255922	2,352599795	9,20E-09	0,668492706	1,589411519	0,003049616	1,971005657
BSU05630	DinB	0,014241727	1,009920498	0,564065532	-0,45303112	0,73050643	0,017596845	0,870213464
BSU05640	YdgG	-1,45730368	0,364173115	1,05E-06	0,069049502	1,049025321	0,298951198	0,706599218
BSU05650	YdgH	-0,14078838	0,907023366	0,005458911	-0,26419485	0,832663307	0,240714939	0,869843336
BSU05660	YdgI	-0,63896434	0,642173776	0,002439388	-0,29435113	0,815439005	0,180131014	0,72880639
BSU05670	YdgJ	0,737990545	1,667851157	0,002695679	0,115924296	1,083669101	0,271720431	1,375760129
BSU05680	YdgK	-0,42537629	0,744644484	0,039450724	-0,25925654	0,835518372	0,365946498	0,790081428
BSU05690	YdhB	-0,5756817	0,670969131	6,66E-06	-0,46705117	0,723441783	0,029247591	0,697205457
BSU05700	YdhC	-0,40155211	0,757043387	0,004877812	-0,21102962	0,863920453	0,337913389	0,81048192
BSU05710	YdhD	-1,29480497	0,407591264	5,31E-07	-0,57837964	0,669715545	0,008847399	0,538653405
BSU05720	YdhE	-0,29262178	0,816417053	0,013079749	-0,05631856	0,961715075	0,168997411	0,889066064
BSU05730	YdhF	-0,71861921	0,60767877	0,00608935	-0,76878018	0,586913508	0,001682678	0,597296139
BSU05740	PhoB	-0,66094102	0,632465629	0,001103396	-0,75184206	0,593844842	0,000950634	0,613155235
BSU05750	Fra	0,296942381	1,228537914	0,077496345	0,369870176	1,292236541	0,033766111	1,260387227
BSU05760	YdhH	0,739732149	1,669865783	0,001926484	0,251052675	1,190075147	0,164873872	1,429970465
BSU05770	YdhI	0,279440483	1,213724078	0,449074903	1,053824626	2,076026164	0,00162684	1,644875121
BSU05780	YdhJ	0,476371593	1,391240266	0,042124574	0,382899658	1,303960039	0,109383216	1,347600152
BSU05790	YdhK	0,195617696	1,145214384	0,032203346	0,608808536	1,524999253	0,010753359	1,335106818
BSU05800	PbuE	-0,11316167	0,924559668	0,248855722	0,092938174	1,066540074	0,240258438	0,995549871
BSU05810	GmuB	-1,4902023	0,355962632	4,69E-05	1,021420528	2,029916703	6,17E-05	1,192939667
BSU05820	GmuA	-1,05672754	0,480721243	0,000103406	0,960764742	1,946341336	0,000116103	1,213531289

BSU05830	GmuC	-1,32533776	0,399055757	2,72E-06	1,278023462	2,425065075	3,02E-06	1,412060416
BSU05840	GmuD	-0,92360597	0,527189678	0,00014731	0,356777531	1,280562379	0,143138367	0,903876028
BSU05850	GmuR	-0,96810285	0,511177821	0,000219637	0,341388917	1,266975755	0,042924707	0,889076788
BSU05860	GmuE	-0,82228538	0,56554535	0,000328737	0,434371336	1,351321857	0,028020592	0,958433604
BSU05870	GmuF	-1,04457477	0,484787777	1,82E-05	0,487747001	1,402253322	0,007349491	0,943520549
BSU05880	GmuG	-1,03470532	0,488115571	0,000303584	-0,27307416	0,827554276	0,293104427	0,657834924
BSU05900	ThiL	-0,71378734	0,609717414	0,006858355	0,771057975	1,706520772	0,000353488	1,158119093
BSU05910	YdiB	-0,00768565	0,99468688	0,161839719	0,474535023	1,389470325	0,012204761	1,192078602
BSU05920	YdiC	-0,73579603	0,600486607	0,005494497	0,660244993	1,58035097	0,000631081	1,090418789
BSU05930	YdiD	-0,5141844	0,700188659	0,042393905	0,597659336	1,513259425	0,001375499	1,106724042
BSU05940	Gcp	-0,71231298	0,610340834	0,006215495	0,436580085	1,3533923	0,031909544	0,981866567
BSU05950	YdiF	0,291244944	1,223695785	0,000662257	-0,13154032	0,912856304	0,332273721	1,068276045
BSU05960	YdiG	-0,46322411	0,725363417	0,062442349	-0,06549004	0,955620665	0,617041913	0,840492041
BSU05970	Rex	-0,11608205	0,922690016	0,470552	-0,19624511	0,872819287	0,483615407	0,897754651
BSU05980	TatAY	0,218605545	1,163608343	0,017536487	0,213454604	1,159461249	0,042170848	1,161534796
BSU05990	TatCY	0,523182783	1,437122242	0,004121936	-0,11553079	0,923042646	0,719332914	1,180082444
BSU06000	YdiK	1,222007426	2,332710747	2,80E-06	-0,68722438	0,621047541	0,009700718	1,476879144
BSU06010	YdiL	0,299755536	1,230935814	0,006002192	-0,00150067	0,998960356	0,715243467	1,114948085
BSU06020	GroES	0,156191074	1,114341227	0,287522245	0,424033263	1,341673158	0,001416491	1,228007192
BSU06030	GroEL	0,573231409	1,487852396	0,062438293	0,327163818	1,25454465	0,009610491	1,371198523
BSU06040	YdiM	-0,29872203	0,812972225	0,161430814	1,135978802	2,197676143	1,14E-06	1,505324184
BSU06048	YdzU	-0,98268587	0,506036774	0,00215116	1,048859676	2,068893922	5,69E-06	1,287465348
BSU06060	YdiO	0,613966221	1,530460936	9,42E-06	1,373192257	2,590431182	1,91E-09	2,060446059
BSU06070	YdiP	0,916289553	1,887255241	6,67E-08	1,183103253	2,270646708	6,33E-07	2,078950975
BSU06090	YdiR	0,034149092	1,023952713	0,252931916	1,078542233	2,111901038	7,68E-06	1,567926876
BSU06100	YdiS	-0,2294942	0,852933876	0,326186303	0,684292805	1,606914094	0,000847855	1,229923985
BSU06110	YdjA	0,715720164	1,642302818	3,38E-06	0,160707234	1,117834986	0,321205412	1,380068902
BSU06120	YdjB	1,01728497	2,024106176	0,222710104	0,463442404	1,378827907	0,428512327	1,701467041
BSU06130	YdjC	0,440606252	1,357174522	1	0,080935539	1,057703703	0,99127651	1,207439113

BSU06140	GutR	-0,17460344	0,886011031	0,43984016	0,159518152	1,116914036	0,393176508	1,001462533
BSU06150	GutB	-0,097925	0,934375918	0,109363537	0,718887724	1,645912595	0,029066749	1,290144256
BSU06160	GutP	-0,10998397	0,926598355	0,398293681	0,51335721	1,427367877	0,017837479	1,176983116
BSU06170	FruC	0,086042784	1,061454687	0,148724514	0,022984061	1,016058917	0,468568655	1,038756802
BSU06180	PspA	0,644455307	1,563149005	0,000151532	0,375429632	1,297225809	0,130693848	1,430187407
BSU06190	YdjG	-0,02130377	0,985341847	0,70196373	1,045224081	2,063686868	0,000406906	1,524514358
BSU06200	YdjH	0,112321327	1,080966135	0,421298629	0,7788992	1,715821174	0,03735367	1,398393655
BSU06210	YdjI	0,427705886	1,345092964	0,090718881	0,036180963	1,025395849	0,59095625	1,185244406
**BSU06220**	**YdjJ**	**1,890387119**	**3,707346909**	**5,00E-05**	**1,090493902**	**2,129469257**	**0,001080653**	**2,918408083**
BSU06230	IolT	-0,08659428	0,94174327	0,693089992	0,431661281	1,348785826	0,048706778	1,145264548
BSU06240	BdhA	1,265872026	2,404725178	9,39E-09	0,440995839	1,357541065	0,036568373	1,881133121
BSU06250	YdjM	1,435595254	2,70493749	4,35E-09	0,586847848	1,501961516	0,002983974	2,103449503
BSU06260	YdjN	0,57060894	1,485150298	0,001464107	0,494648339	1,408977271	0,0045174	1,447063784
BSU06269	YdzJ	-0,29274698	0,816346206	0,168982559	-1,16165411	0,446999739	5,95E-07	0,631672973
BSU06270	YdjO	-1,20968116	0,432364161	3,80E-08	-1,30402671	0,404994239	4,70E-06	0,4186792
BSU06280	YdjP	-1,43975406	0,368630142	2,73E-07	-1,00268739	0,499069489	0,000640563	0,433849815
**BSU06290**	**YeaA**	**-1,64123612**	**0,320581679**	**1,06E-10**	**-1,19796927**	**0,435888404**	**4,98E-05**	**0,378235041**
**BSU06300**	**CotA**	**-2,24189419**	**0,211408576**	**5,38E-15**	**-2,11897085**	**0,230211075**	**6,51E-14**	**0,220809826**
BSU06310	GabP	0,722714646	1,650284371	0,00028615	0,792342307	1,731884005	0,000403676	1,691084188
BSU06319	YdzX	0,026202518	1,018328137	0,987072931	0,080935539	1,057703703	0,872865056	1,03801592
BSU06320	YeaB	0,795577888	1,735772519	0,000467207	0,461929067	1,377382323	0,028518731	1,556577421
BSU06330	YeaC	0,399386716	1,318947112	0,072252178	1,358130279	2,56352733	3,51E-08	1,941237221
BSU06340	YeaD	0,462391357	1,377823755	0,000267946	1,21015076	2,313618125	4,31E-06	1,84572094
BSU06350	YebA	-1,49157315	0,355624557	3,59E-07	0,348389809	1,273138884	0,056970032	0,81438172
BSU06360	GuaA	0,276601631	1,211338129	0,062941722	0,153752685	1,1124594	0,21307659	1,161898764
BSU06370	PbuG	-0,55590824	0,680228689	0,00284964	-0,57056238	0,673354254	0,009900181	0,676791471
BSU06380	YebC	0,662122077	1,582408496	0,000198576	-0,19430368	0,873994629	0,311482204	1,228201562
BSU06390	YebD	-0,01280323	0,991164743	0,192279512	-1,2345732	0,424968201	1,95E-07	0,708066472
BSU06400	YebE	-0,54815589	0,68389375	0,028454153	-0,30044816	0,812000115	0,190388149	0,747946933

BSU06410	YebG	-0,91693453	0,529633202	0,000651932	-0,24853924	0,841748273	0,245952314	0,685690738
BSU06420	PurE	-0,92352198	0,527220372	2,51E-06	0,431704791	1,348826504	0,02981522	0,938023438
BSU06430	PurK	-1,67891672	0,312317059	4,81E-11	0,583252	1,498222611	0,001242923	0,905269835
BSU06440	PurB	-1,39108322	0,38127842	2,96E-07	0,683683757	1,606235863	0,000628401	0,993757141
BSU06450	PurC	-1,28132079	0,411418683	4,85E-08	1,065009937	2,09218429	4,57E-07	1,251801487
BSU06460	PurS	-1,07670931	0,474109001	2,37E-05	1,015743093	2,021944073	6,35E-07	1,248026537
BSU06470	PurQ	-1,34319307	0,394147337	4,53E-07	1,140582983	2,204700956	4,53E-07	1,299424147
BSU06480	PurL	-0,7696526	0,5865587	0,00051814	0,8370832	1,786434725	3,10E-05	1,186496712
BSU06490	PurF	-0,80004582	0,574330937	0,00263824	0,81052618	1,753850991	6,15E-05	1,164090964
BSU06500	PurM	-0,3418005	0,789055947	0,058074047	1,024117158	2,033714494	7,26E-07	1,41138522
BSU06510	PurN	-0,86389823	0,549465867	0,001039502	1,161857885	2,237453788	1,09E-07	1,393459827
BSU06520	PurH	-0,65579891	0,634723906	0,008883475	0,999008386	1,998625804	3,03E-06	1,316674855
BSU06530	PurD	-0,22454401	0,855865498	0,397976491	0,907459157	1,875739077	6,79E-05	1,365802287
BSU06540	YezC	-0,22351236	0,856477733	0,209011428	-0,79550157	0,576142834	0,003186232	0,716310284
BSU06550	YecA	1,807217355	3,499666275	9,12E-09	-0,08434523	0,943212517	0,146377935	2,221439396
**BSU06559**	**YezF**	**-1,35756775**	**0,390239644**	**1,31E-08**	**-2,04984937**	**0,241509296**	**5,82E-14**	**0,31587447**
BSU06560	YerA	0,993390896	1,99085879	3,91E-05	0,741369497	1,671762028	0,000126083	1,831310409
BSU06570	YerB	-0,00805554	0,994431887	0,6024303	0,021243864	1,014834073	0,322324869	1,00463298
BSU06580	YerC	0,616737494	1,533403623	6,80E-05	-0,05320924	0,963790012	0,868295166	1,248596818
BSU06590	YerD	1,679517295	3,203207584	9,15E-06	0,681117333	1,603381055	0,005146232	2,403294319
BSU06600	PcrB	-0,43616761	0,739095345	0,008514998	0,276675022	1,211399752	0,130276257	0,975247549
BSU06610	PcrA	-1,06931369	0,476545646	9,20E-06	0,292428636	1,224700206	0,085592557	0,850622926
BSU06620	LigA	-0,51168504	0,701402734	0,003393361	-0,12413533	0,917553807	0,811191141	0,809478271
BSU06630	YerH	-0,14788533	0,902572466	0,106755214	-0,26834696	0,830270324	0,307228583	0,866421395
BSU06640	YerI	0,767013	1,701742793	0,001015158	-0,0064075	0,995568505	0,445170616	1,348655649
BSU06650	SapB	-0,05767085	0,960814048	0,012646726	-0,84212742	0,557820393	0,001487205	0,75931722
BSU06660	OpuE	2,374639368	5,186061693	2,36E-12	0,528937201	1,442865879	0,008543761	3,314463786
BSU06670	GatC	-0,15025729	0,90108975	0,215037536	0,64685502	1,565751238	0,000595603	1,233420494
BSU06680	GatA	-0,19752011	0,872048263	0,308746187	0,440227435	1,356818208	0,010353078	1,114433236

BSU06690	GatB	0,761271471	1,694983784	0,000791041	0,222783352	1,166982844	0,119130732	1,430983314
BSU06700	YerO	-0,12303501	0,918253878	0,103115804	-0,37164327	0,772901636	0,024475586	0,845577757
BSU06710	SwrC	0,573339739	1,487964121	0,005453278	0,14749468	1,107644315	0,270321957	1,297804218
BSU06720	DgkB	-0,16936361	0,889234844	0,391837836	0,136995616	1,099612808	0,250311461	0,994423826
BSU06730	RlmCD	-1,04273288	0,4854071	0,000200399	-0,27771306	0,824897595	0,181958307	0,655152348
BSU06740	YefB	-1,74689901	0,297941499	0,095607428	0,213853924	1,159782218	0,251651882	0,728861859
BSU06750	YefC	0,428238193	1,345589351	0,168057814	1,274119651	2,418511925	4,56E-07	1,882050638
BSU06760	YeeA	0,027614918	1,01932557	0,259764367	0,922041859	1,894795117	0,000279616	1,457060343
BSU06770	YeeB	-0,10387459	0,930530543	0,028102521	0,807642916	1,750349374	0,000188453	1,340439958
BSU06780	YeeC	0,485087004	1,399670272	0,044480591	1,392871193	2,626007784	9,09E-09	2,012839028
BSU06790	YeeD	-1,65419101	0,317715855	1,83E-08	-0,85886978	0,551384349	0,00021797	0,434550102
BSU06800	YezA	-1,19893058	0,435598055	3,00E-05	-0,72032499	0,606960699	0,002011797	0,521279377
BSU06811	YezG	-0,79119196	0,577866459	0,000805217	0,218750897	1,163725582	0,296067432	0,870796021
BSU06812	YeeF	-0,94311199	0,520109758	8,73E-06	-0,3987103	0,758536077	0,013585482	0,639322918
BSU06820	YeeG	1,438961577	2,711256445	2,41E-06	-0,15168381	0,900199203	0,266725108	1,805727824
BSU06830	RapH	0,074189983	1,052769781	0,010063037	1,101256718	2,145414962	3,97E-06	1,599092371
BSU06839	PhrH	0,17397303	1,128161047	0,229340767	0,888413434	1,851139262	3,56E-05	1,489650154
BSU06840	YeeI	0,360986605	1,284303884	0,015788931	0,351121327	1,275551658	0,052429503	1,279927771
**BSU06850**	**YeeK**	**-1,86087406**	**0,275309432**	**1,24E-11**	**-1,97403357**	**0,254540378**	**8,03E-13**	**0,264924905**
BSU06860	YezE	0,808243762	1,751078501	0,001079349	-0,17382233	0,886490866	0,405742693	1,318784684
BSU06870	YesE	-0,42354612	0,745589726	0,095177746	0,089660908	1,064120042	0,421850431	0,904854884
BSU06880	YesF	-0,17402184	0,886368284	0,308270316	-0,90676641	0,533379243	0,009505372	0,709873763
BSU06890	CotJA	-1,40560929	0,377458702	3,63E-09	-1,00067749	0,499765254	0,005503735	0,438611978
BSU06900	CotJB	-1,38865779	0,381919956	3,47E-08	-0,80347176	0,572968701	0,047098886	0,477444328
BSU06910	CotJC	-1,29039026	0,408840421	1,11E-06	-0,77137623	0,58585834	0,079991859	0,49734938
BSU06920	YesJ	-1,10052722	0,466346044	4,13E-05	-0,83784003	0,559480586	0,005158105	0,512913315
BSU06930	YesK	0,219273906	1,164147535	0,000741559	-0,22849715	0,853523543	0,50427373	1,008835539
BSU06940	YesL	1,542515412	2,913019607	8,00E-09	0,614343195	1,530860895	0,001939339	2,221940251
BSU06950	YesM	-0,23107065	0,852002371	0,036314479	1,363017756	2,572226624	1,76E-08	1,712114498

BSU06960	YesN	0,906142969	1,874028598	1,21E-05	0,309951677	1,239666176	0,070341059	1,556847387
BSU06970	YesO	0,014298075	1,009959943	0,20267144	1,258076848	2,391766993	1,09E-06	1,700863468
BSU06980	YesP	-0,07025555	0,952469269	0,451311898	0,857195681	1,811513655	5,54E-05	1,381991462
BSU06990	YesQ	0,255455746	1,193712776	0,009357622	0,615612668	1,53220854	0,003133513	1,362960658
BSU07000	YesR	-0,26706609	0,831007794	0,123655326	0,595000485	1,510473094	0,017960105	1,170740444
BSU07010	YesS	-0,49100425	0,711529633	0,064069888	0,855971431	1,809977083	7,63E-05	1,260753358
BSU07020	YesT	-0,73127761	0,602370236	0,004175586	0,8629688	1,81877717	0,000118723	1,210573703
BSU07030	YesU	-0,95223775	0,51683019	0,000642389	0,886538524	1,848735106	0,000429703	1,182782648
BSU07040	YesV	0,123722516	1,089542533	0,01223817	0,663075103	1,58345416	0,001615676	1,336498346
BSU07050	YesW	-0,20954901	0,864807531	0,408295637	0,574364778	1,489021699	0,001845007	1,176914615
BSU07060	YesX	-0,51044455	0,702006089	0,052569181	0,673814897	1,595285785	0,0036633	1,148645937
BSU07070	YesY	0,539458292	1,453426678	0,026876641	0,788992361	1,727867226	0,000195607	1,590646952
BSU07080	YesZ	-0,48951469	0,712264659	0,083541257	1,053842752	2,076052248	0,000229368	1,394158453
BSU07090	YetA	0,377915051	1,299462545	0,083071295	0,966586495	1,954211336	0,003380982	1,626836941
BSU07100	LplA	0,35590161	1,279785131	1,45E-06	0,478749366	1,393535124	0,000858875	1,336660127
BSU07110	LplB	-0,36094044	0,778656838	0,040805904	0,801689688	1,743141503	0,012293115	1,260899171
BSU07120	LplC	-0,63548849	0,643722816	1,95E-05	0,776153033	1,712558217	0,000613271	1,178140517
BSU07130	LplD	-2,00390328	0,249324527	2,83E-12	-0,48952543	0,712259356	0,022442725	0,480791941
BSU07140	YetF	-0,63795424	0,642623551	0,006879319	-0,73424195	0,601133802	0,00145022	0,621878676
BSU07150	HmoA	0,389901123	1,310303597	0,008540516	-0,32722651	0,797067321	0,233191095	1,053685459
BSU07160	YetH	-0,83971163	0,558755244	0,000491066	-0,51695175	0,698846859	0,016649608	0,628801052
BSU07190	YezD	-2,07405521	0,237491007	1,77E-11	-0,52896459	0,693051956	0,015740976	0,465271481
BSU07200	YetJ	0,630590947	1,548199026	8,71E-05	0,045521071	1,032055869	0,608027474	1,290127447
BSU07210	YetK	1,151303306	2,221144582	8,25E-07	0,275725805	1,210602978	0,10795669	1,71587378
BSU07220	YetL	0,629157754	1,546661788	0,005814957	-0,541866	0,686881912	0,009861294	1,11677185
BSU07230	YetM	-0,23365279	0,850478817	0,173141383	-0,57337766	0,672041551	0,124055848	0,761260184
BSU07240	YetN	-0,39565811	0,760142546	0,026730435	-0,15575041	0,897665338	0,191034436	0,828903942
**BSU07250**	**YetO**	**-3,13039858**	**0,114197377**	**7,70E-18**	**-1,15902145**	**0,447816177**	**4,91E-07**	**0,281006777**
BSU07260	YfnI	0,710298866	1,636143022	0,000513873	0,792401997	1,731955661	0,000235219	1,684049342

BSU07270	YfnH	-0,83226599	0,561646393	0,10648524	1,283420042	2,43415332	0,032944437	1,497899857
BSU07280	YfnG	-1,13490893	0,455363661	0,018777412	1,005216412	2,007244572	0,023719989	1,231304117
BSU07290	YfnF	-0,46226956	0,725843507	0,005743347	0,378657867	1,300131786	0,316131912	1,012987647
BSU07300	YfnE	-1,58028795	0,334415137	1,49E-07	-0,11703041	0,92208368	0,271981355	0,628249409
BSU07310	YfnD	-1,72563637	0,302365121	1,19E-08	-0,20601088	0,866931024	0,26950619	0,584648072
BSU07320	YfnC	-1,5712506	0,336516559	2,80E-09	-1,02579984	0,491137933	4,78E-05	0,413827246
BSU07330	YfnB	-0,32391448	0,798899275	0,408109144	-0,2346922	0,849866298	0,245920619	0,824382786
BSU07340	YfnA	-0,68860539	0,620453333	0,010644127	-0,95242997	0,516761335	4,40E-05	0,568607334
BSU07350	YfmT	0,333061396	1,259683586	0,018662597	-0,65655858	0,634389775	0,009667397	0,947036681
BSU07360	YfmS	0,77212322	1,707781284	0,006296458	-0,41282185	0,751152714	0,03150626	1,229466999
BSU07370	YfmR	1,271657639	2,414388164	1,31E-08	0,662535273	1,582861771	0,002039944	1,998624968
BSU07380	YfmQ	0,50916688	1,42322808	0,000228809	-0,18839764	0,877579883	0,181895299	1,150403982
BSU07390	YfmP	0,363209159	1,286283953	0,299355954	-0,70921634	0,611652292	0,029272993	0,948968122
BSU07400	YfmO	0,683369692	1,605886235	0,027288364	0,689182362	1,61236946	0,000675265	1,609127848
BSU07410	YfmN	-0,51789034	0,698392348	0,802813769	0,080935539	1,057703703	1	0,878048026
BSU07420	YfmM	0,06589552	1,046734475	0,007179859	-0,64280982	0,640464351	0,000263552	0,843599413
BSU07430	YfmL	-1,03280407	0,488759258	1,22E-05	-0,98967919	0,503589744	6,67E-05	0,496174501
BSU07440	YfmK	0,030341369	1,021253745	0,261625499	-0,3412872	0,789336737	0,133591302	0,905295241
BSU07450	YfmJ	1,456580898	2,74457145	2,71E-08	0,5825282	1,497471142	0,001085818	2,121021296
BSU07460	YfmI	0,899338479	1,86521053	6,00E-05	1,17596848	2,259445065	3,23E-07	2,062327797
BSU07480	YfmG	0,721024076	1,648351678	3,10E-05	0,71009218	1,635908639	0,000212206	1,642130158
BSU07490	YfmF	-0,3328143	0,793986126	0,032376306	-0,14617226	0,903644825	0,455490204	0,848815476
BSU07500	YfmE	0,970172936	1,959075416	0,000104942	0,091825057	1,065717498	0,061457539	1,512396457
BSU07510	YfmD	1,339534172	2,530695926	5,99E-09	0,516732736	1,430711452	0,020759804	1,980703689
BSU07520	YfmC	0,398794421	1,318405733	0,001139116	0,17219676	1,126772891	0,330195802	1,222589312
BSU07530	YfmB	0,550381842	1,464473251	0,002163537	0,376704888	1,298372985	0,041183987	1,381423118
BSU07540	YfmA	-0,20377111	0,868277973	0,160855124	-0,71979085	0,607185462	0,003912741	0,737731717
BSU07550	YflT	1,094316353	2,135118812	9,84E-07	0,28440526	1,217908087	0,046365561	1,676513449
BSU07560	Pel	-0,85366422	0,553377456	1,35E-05	-0,66726017	0,629701423	0,05516375	0,59153944

BSU07570	YflS	-0,57502633	0,671273999	6,32E-06	-1,18682587	0,43926825	9,48E-07	0,555271125
BSU07580	CitS	0,502312271	1,416482003	0,009320193	1,150467025	2,219857434	4,54E-07	1,818169718
BSU07590	CitT	0,541141121	1,455123013	0,005671834	0,882788025	1,843935287	5,41E-06	1,64952915
BSU07600	YflP	0,56223984	1,476559855	0,002481218	0,938320737	1,916296416	0,000187266	1,696428135
BSU07610	CitM	0,501867384	1,416045266	0,08556699	0,731806658	1,660717473	0,007687853	1,538381369
**BSU07620**	**YflN**	**-2,12552507**	**0,229167591**	**9,80E-09**	**-1,43108433**	**0,370852056**	**4,61E-08**	**0,300009823**
BSU07630	Nos	-0,3207271	0,800666249	0,060965463	0,03817783	1,026816106	0,589821672	0,913741177
BSU07640	YflL	0,345611841	1,270689762	0,043607759	-0,88887427	0,540035342	0,000164831	0,905362552
BSU07650	YflK	0,306175513	1,236425671	0,001206332	0,21598219	1,161494394	0,129092845	1,198960032
BSU07660	YflJ	-0,75638086	0,591979509	0,004005029	-1,66581611	0,315166017	3,46E-10	0,453572763
BSU07670	YflI	0,581848526	1,496765828	0,033805711	-0,68163137	0,623459881	0,004409459	1,060112855
BSU07680	YflH	-0,16367131	0,892750342	0,121919604	-0,39473774	0,760627636	0,036256636	0,826688989
BSU07690	YflG	-0,34773397	0,785817409	0,031233337	-0,46556199	0,724188922	0,017969821	0,755003166
BSU07700	NagP	-0,75306756	0,593340613	0,000603287	-0,98791619	0,504205518	0,000135252	0,548773065
BSU07710	LtaS	0,707329767	1,632779265	0,000622544	0,730066916	1,658716025	0,000431235	1,645747645
BSU07720	YflD	-0,87751373	0,54430465	0,000361351	0,080935539	1,057703703	0,197169202	0,801004177
BSU07735	YflB	-0,76502759	0,588442117	0,005336269	-0,62649842	0,647746664	0,001172558	0,618094391
**BSU07750**	**YflA**	**1,655284532**	**3,149853066**	**2,49E-11**	**1,356504402**	**2,560639935**	**1,29E-08**	**2,855246501**
BSU07760	YfkT	-0,91624462	0,529886539	0,000225622	-1,33381424	0,396718	4,07E-07	0,46330227
BSU07770	YfkS	-1,51726301	0,349348049	9,11E-10	-0,85728026	0,551992184	9,60E-05	0,450670117
**BSU07780**	**YfkR**	**-2,23307643**	**0,212704664**	**6,06E-12**	**-1,16575132**	**0,445732075**	**7,96E-07**	**0,329218369**
**BSU07790**	**YfkQ**	**-2,40457049**	**0,188865292**	**1,82E-15**	**-1,28662736**	**0,409908166**	**2,55E-08**	**0,299386729**
BSU07800	TreP	0,220164904	1,164866726	0,37867631	0,248659084	1,188102318	0,106435446	1,176484522
BSU07810	TreA	-1,27596105	0,41294998	0,071766618	0,47283172	1,387830829	0,534333812	0,900390404
BSU07820	TreR	0,137583376	1,100060886	0,244048737	-0,05235338	0,964361938	0,601027049	1,032211412
BSU07830	HypO	-0,85677296	0,552186317	0,001243663	-0,62456526	0,648615202	0,007877117	0,600400759
BSU07840	YfkN	0,132085312	1,095876568	0,402988395	0,485903226	1,400462377	0,006870747	1,248169472
BSU07850	YfkM	1,722470449	3,30001012	3,26E-12	0,142713914	1,103979906	0,367298779	2,201995013
BSU07860	YfkL	0,317857127	1,246477747	0,017898722	0,363952329	1,286946722	0,057114747	1,266712235

BSU07870	YfkK	0,107518096	1,077373209	0,206818403	0,107775709	1,077565606	0,366387117	1,077469408
BSU07880	YfkJ	-0,91405035	0,530693085	8,16E-05	-0,35594254	0,781358999	0,175690246	0,656026042
BSU07890	YfkI	-0,69180643	0,619078203	0,018482766	-0,3416924	0,789115074	0,120827348	0,704096639
BSU07900	YfkH	0,040143149	1,028215845	0,461754728	-0,36084429	0,778708734	0,046062387	0,903462289
BSU07910	YfkF	1,108903405	2,15681645	3,05E-06	-0,66794667	0,629401854	0,008233556	1,393109152
BSU07920	ChaA	0,084719233	1,060481338	0,005561211	-0,63641762	0,643308376	0,00877631	0,851894857
**BSU07930**	**YfkD**	**-1,59017239**	**0,332131763**	**1,26E-09**	**-1,98208266**	**0,253124199**	**2,16E-12**	**0,292627981**
BSU07940	YfkC	-2,06211222	0,239465177	3,92E-08	-0,70673518	0,612705122	0,001217767	0,426085149
BSU07955	YfkA	-1,44297684	0,367807591	4,54E-08	0,072900847	1,051829487	0,295967561	0,709818539
BSU07970	YfjT	-0,3274598	0,796938446	0,210655758	-0,51455551	0,700008569	0,022365361	0,748473507
**BSU07980**	**PdaA**	**-2,84888974**	**0,138802962**	**8,94E-17**	**-1,97909973**	**0,253648102**	**2,05E-13**	**0,196225532**
BSU07990	YfjR	0,34389832	1,269181432	0,06984579	0,221829469	1,166211512	0,192802149	1,217696472
BSU08000	YfjQ	-1,58595931	0,333103101	8,16E-09	-0,63594068	0,643521085	0,004294802	0,488312093
BSU08010	YfjP	0,214020578	1,159916199	0,201585916	0,610530472	1,52682051	0,004834603	1,343368354
BSU08020	YfjO	0,08232572	1,058723398	0,417733676	-0,40262528	0,75648046	0,081633597	0,907601929
BSU08029	YfzA	1,663185881	3,167151502	4,76E-11	0,434996885	1,351907914	0,044244096	2,259529708
BSU08030	YfjN	0,53203908	1,445971462	0,002716627	0,579111534	1,493928947	0,006809612	1,469950205
BSU08040	YfjM	0,587618735	1,502764286	0,026566706	0,383713608	1,304695924	0,161726931	1,403730105
BSU08050	YfjL	-0,18321196	0,880739967	0,439068715	-0,1578984	0,896329821	0,383818709	0,888534894
BSU08060	AcoA	2,203346757	4,605464774	1,34E-13	0,822990338	1,769069023	1,31E-05	3,187266898
BSU08070	AcoB	2,365276803	5,152515011	3,94E-14	0,698294832	1,622585872	2,10E-05	3,387550442
BSU08080	AcoC	2,019590412	4,054686611	8,68E-13	0,321368353	1,249515114	0,022021678	2,652100863
BSU08090	AcoL	2,05522195	4,156075752	2,49E-13	0,531833478	1,445765408	0,001865292	2,80092058
BSU08100	AcoR	1,133158421	2,193384018	1,68E-08	1,093437359	2,13381834	3,92E-07	2,163601179
BSU08110	SspH	-0,62186743	0,64982924	0,000279162	-0,5455927	0,685109884	0,014717213	0,667469562
BSU08120	YfjF	-0,99797979	0,500700643	2,22E-05	-1,0686485	0,476765419	2,10E-05	0,488733031
BSU08130	YfjE	-1,62457123	0,324306258	4,77E-08	-0,48838317	0,712823514	0,013378373	0,518564886
BSU08140	YfjD	-1,88458271	0,270822084	4,99E-11	-0,35411361	0,782350169	0,080109961	0,526586126
BSU08150	YfjC	-1,4510598	0,365752644	1,31E-09	-0,24401751	0,844390641	0,296967393	0,605071642

BSU08160	YfjB	-1,44826664	0,366461454	3,65E-09	-0,3848034	0,765883361	0,107872776	0,566172407
BSU08170	YfjA	-0,75126306	0,594083217	0,00022391	-0,4783303	0,717807896	0,011451341	0,655945556
BSU08180	MalA	-0,59003886	0,664325011	0,007800157	0,38576936	1,306556362	0,02460644	0,985440686
BSU08190	GlvR	-0,59706652	0,661096819	0,027737072	0,374737828	1,296603909	0,022134164	0,978850364
BSU08200	MalP	0,027310501	1,019110509	0,403751843	0,561531368	1,475834931	0,006975514	1,24747272
BSU08210	YfiB	-0,58913274	0,66474239	0,081357938	0,367891079	1,290465059	0,030031925	0,977603724
BSU08220	YfiC	-0,94239051	0,520369927	0,011022497	0,069028313	1,049009914	0,421586649	0,784689921
**BSU08230**	**CatD**	**-2,28981051**	**0,204502373**	**7,86E-14**	**-1,03006173**	**0,489689195**	**7,10E-06**	**0,347095784**
**BSU08240**	**CatE**	**-2,71626103**	**0,152168218**	**6,99E-13**	**-0,93902859**	**0,521583959**	**0,000178148**	**0,336876088**
BSU08250	YfiF	-0,40185734	0,756883235	0,0055214	0,628859904	1,546342507	0,050595654	1,151612871
BSU08260	YfiG	0,20406206	1,151937194	0,023114664	1,489203635	2,807339678	3,39E-10	1,979638436
BSU08270	YfiH	0,304821207	1,235265542	0,032246465	1,430457043	2,69532089	8,99E-10	1,965293216
BSU08280	YfiI	0,719195939	1,646264262	0,000286435	1,244515739	2,369390089	4,63E-08	2,007827175
BSU08290	YfiJ	-0,74961695	0,594761453	0,000304914	-0,49684017	0,708657203	0,020123215	0,651709328
BSU08300	YfiK	-1,34663447	0,393208259	4,54E-06	-0,29528411	0,814911839	0,169324133	0,604060049
BSU08310	YfiL	-0,76193057	0,589706676	0,53294709	-0,24176432	0,845710435	0,48876702	0,717708556
**BSU08320**	**YfiM**	**-1,59033815**	**0,332093606**	**0,086014621**	**-1,36112743**	**0,389277961**	**0,289631471**	**0,360685784**
BSU08330	YfiN	-0,24045357	0,846479146	0,314442091	-0,33621091	0,792118999	0,116771838	0,819299073
BSU08340	PadR	0,619953449	1,536825592	0,000368598	-0,29458683	0,815305791	0,153499621	1,176065691
BSU08350	LipB	0,15427356	1,112861118	1,50E-05	-0,95999717	0,514057923	0,001981953	0,81345952
BSU08360	YfiQ	-0,67371733	0,626889328	0,020301613	-0,88212931	0,542566053	0,000430943	0,58472769
BSU08370	YfiR	-0,07008542	0,952581598	0,008078605	-0,07541891	0,949066507	0,130682297	0,950824052
BSU08380	YfiS	0,262201948	1,19930778	0,395830265	-0,50313063	0,705574033	0,02312082	0,952440907
BSU08390	YfiT	-0,20858201	0,865387383	0,019018693	-0,14632106	0,903551627	0,582801531	0,884469505
BSU08400	YfiU	-0,94236844	0,520377885	0,000343957	-0,74940606	0,594848399	0,000683825	0,557613142
BSU08410	YfiV	0,207374573	1,154585148	0,040148556	-0,19574848	0,873119796	0,377033986	1,013852472
BSU08425	MprF	0,502982905	1,417140604	0,002298992	0,792124997	1,731623155	0,000987409	1,57438188
BSU08440	YfiY	1,502740054	2,83380415	5,05E-08	0,640627364	1,559006955	0,001062108	2,196405553
BSU08450	YfiZ	0,602047457	1,517869182	8,26E-05	-0,38060028	0,768117922	0,100937488	1,142993552

BSU08460	YfhA	-0,10243594	0,931458925	0,061557244	-0,77257741	0,585370762	0,003592109	0,758414843
BSU08470	YfhB	-0,06237384	0,957687028	0,043433146	0,146897378	1,107185825	0,260233492	1,032436427
BSU08480	YfhC	-0,22074024	0,858125024	0,075238364	-0,43195504	0,741256605	0,093291102	0,799690814
**BSU08490**	**YfhD**	**-1,86383999**	**0,274744025**	**2,97E-12**	**-2,60102564**	**0,164821272**	**6,86E-16**	**0,219782649**
BSU08500	YfhE	-0,32216654	0,79986779	0,034617297	-1,27598604	0,412942828	2,50E-07	0,606405309
BSU08510	YfhF	0,090729382	1,064908431	0,389712846	-0,68434061	0,62229018	0,001855866	0,843599306
BSU08520	YfhG	-0,74033024	0,598602314	0,009330272	-0,40302246	0,756272225	0,090839033	0,677437269
BSU08530	YfhH	-0,31136954	0,805876384	0,228792977	-0,43946935	0,737405788	0,001098542	0,771641086
BSU08540	YfhI	-0,42204831	0,746364201	0,001919737	-1,14673404	0,451646508	8,47E-06	0,599005355
**BSU08550**	**SspK**	**-3,20085**	**0,108754726**	**7,30E-18**	**-2,77924751**	**0,145667657**	**4,89E-17**	**0,127211191**
BSU08560	YfhJ	1,020518895	2,028648474	2,20E-05	-0,24729914	0,842472127	0,203359358	1,435560301
BSU08570	YfhK	1,955785245	3,879270142	3,43E-13	0,853412447	1,806769478	3,14E-05	2,84301981
BSU08580	YfhL	1,052338231	2,073888356	2,07E-06	0,547450655	1,461500836	0,011399893	1,767694596
BSU08590	YfhM	0,984996445	1,979308436	9,08E-07	0,003204744	1,002223828	0,383043221	1,490766132
BSU08600	CsbB	0,647653864	1,566618461	0,000749199	0,631735941	1,549428241	0,004468117	1,558023351
BSU08610	YfhO	-1,17118579	0,444056209	4,89E-05	-0,35958933	0,779386405	0,046854286	0,611721307
BSU08620	YfhP	-0,16678892	0,890823228	0,479558213	-0,74547962	0,596469544	0,000613085	0,743646386
BSU08630	MutY	0,019349465	1,013502372	0,089440109	-0,04984416	0,966040674	0,596922605	0,989771523
**BSU08640**	**YfhS**	**-2,95967387**	**0,128543283**	**1,14E-17**	**-1,9184642**	**0,264535968**	**3,42E-12**	**0,196539625**
BSU08650	FabL	-0,9871543	0,504471858	0,000293038	0,153933705	1,112598993	0,328943171	0,808535425
**BSU08660**	**SspE**	**-2,75305986**	**0,148335945**	**1,21E-17**	**-3,95545877**	**0,064459698**	**2,04E-19**	**0,106397821**
BSU08670	YgaB	0,74981482	1,681576975	8,72E-06	0,676384046	1,598129199	0,000648752	1,639853087
BSU08680	YgaC	0,237465256	1,178919538	0,182200153	0,231607478	1,174142473	0,138637128	1,176531005
BSU08690	YgaD	-0,82786505	0,563362308	0,001017378	0,495141803	1,409459284	0,023364968	0,986410796
BSU08700	YgaE	-1,719863	0,303577548	2,72E-08	-0,73730157	0,599860287	0,006967779	0,451718918
BSU08710	GsaB	0,282332787	1,216159782	0,09678726	0,011102986	1,007725694	0,731750973	1,111942738
BSU08720	YgaF	-0,41596921	0,749515798	0,011323171	0,050528746	1,035644417	0,577991988	0,892580107
BSU08730	PerR	0,464497058	1,379836241	0,008628463	-0,2294213	0,852976975	0,163657919	1,116406608
BSU08740	YgzB	2,189393897	4,561138242	1,54E-13	0,002004907	1,001390661	0,934426473	2,781264452

BSU08750	YgxA	1,421769103	2,679138387	2,50E-07	0,699300277	1,623717081	0,00168893	2,151427734
BSU08760	Spo0M	-0,25118103	0,840208314	0,225914126	-0,07852608	0,947024675	0,263908137	0,893616494
BSU08770	YgzA	-0,51232101	0,701093608	0,001220178	-1,61597732	0,326243866	1,45E-09	0,513668737
BSU08779	YgzC	-0,71614603	0,608721392	0,000285468	-1,42200405	0,37319355	6,15E-09	0,490957471
BSU08780	YgaJ	0,377256508	1,298869519	0,066701281	-0,53555867	0,689891469	0,003670438	0,994380494
BSU08790	ThiC	1,711129492	3,274170592	4,57E-10	-0,28958757	0,818135909	0,324853344	2,046153251
BSU08800	YgaK	-0,11950956	0,920500522	0,68749197	-1,2194634	0,429442416	0,032895667	0,674971469
BSU08810	SenS	1,091907829	2,131557286	6,20E-06	0,368712423	1,291199946	0,153018491	1,711378616
**BSU08820**	**KatA**	**-2,15093347**	**0,225166879**	**9,32E-15**	**-1,23282785**	**0,425482634**	**5,76E-08**	**0,325324756**
**BSU08830**	**SsuB**	**-2,87012293**	**0,136775058**	**7,13E-15**	**-2,54557057**	**0,171280098**	**1,80E-15**	**0,154027578**
**BSU08840**	**SsuA**	**-3,26938145**	**0,103709399**	**2,73E-18**	**-2,3714497**	**0,193251337**	**4,54E-15**	**0,148480368**
**BSU08850**	**SsuC**	**-3,79274092**	**0,072155795**	**1,49E-19**	**-2,66918285**	**0,157215694**	**7,32E-16**	**0,114685745**
**BSU08860**	**SsuD**	**-3,53037356**	**0,086546929**	**1,01E-13**	**-2,16925902**	**0,222324829**	**4,48E-15**	**0,154435879**
BSU08870	YgaN	-1,12166112	0,459564377	4,62E-05	-1,03320937	0,488621968	6,14E-06	0,474093173
BSU08880	YhzA	-1,33310831	0,396912165	0,35705422	-1,19477356	0,43685501	0,172413608	0,416883587
BSU08890	YgaO	-0,01470989	0,989855687	0,495745731	-1,45733824	0,364164389	2,15E-08	0,677010038
BSU08899	YgzD	0,309045183	1,238887497	0,624097431	-0,43406725	0,740172147	0,112910836	0,989529822
BSU08900	YhzB	-0,93877318	0,521676309	0,000593366	-0,77127166	0,585900808	0,000968834	0,553788559
BSU08910	QueG	-0,67112729	0,62801578	0,040992601	-0,15173197	0,900169152	0,351912505	0,764092466
BSU08920	YhbB	-1,54241297	0,343310773	4,05E-09	-1,00095107	0,499670494	0,000134101	0,421490633
BSU08930	CspR	-0,9668571	0,51161941	5,98E-05	-0,56272662	0,677021419	0,007199799	0,594320415
BSU08940	YhbD	-0,39397039	0,761032307	0,100125952	0,802259697	1,743830355	0,002903662	1,252431331
BSU08950	YhbE	-0,20989811	0,86459829	0,175743381	0,384220479	1,305154392	0,062034193	1,084876341
BSU08960	YhbF	-0,17733091	0,884337573	0,174235073	-0,37855576	0,769207239	0,177651135	0,826772406
BSU08970	PrkA	-0,29863959	0,813018685	0,064736197	-0,36329522	0,777386941	0,427814717	0,795202813
BSU08980	YhbH	0,448378751	1,364506012	0,003996326	-0,63910986	0,642109007	0,032781591	1,00330751
BSU08990	YhbI	-2,22392561	0,214058108	0,000868568	-0,15739584	0,896642107	0,572885068	0,555350108
**BSU09000**	**YhbJ**	**-2,83821665**	**0,139833637**	**1,33E-17**	**-1,08453424**	**0,471544475**	**3,93E-06**	**0,305689056**
**BSU09010**	**YhcA**	**-3,26784291**	**0,103820057**	**4,36E-19**	**-1,13719284**	**0,454643352**	**2,66E-06**	**0,279231704**

**BSU09020**	**YhcB**	**-2,57258467**	**0,168102761**	**1,48E-16**	**-1,5543967**	**0,340470877**	**2,17E-09**	**0,254286819**
**BSU09030**	**YhcC**	**-1,97237912**	**0,254832446**	**8,88E-09**	**-1,48265038**	**0,357830835**	**2,89E-07**	**0,30633164**
BSU09040	YhcD	0,063209517	1,044787482	0,954273521	0,080935539	1,057703703	0,876900637	1,051245593
BSU09050	YhcE	-0,1005406	0,932683437	0,657032964	1,078253149	2,111477902	2,10E-05	1,52208067
BSU09060	YhcF	1,266457185	2,405700735	2,15E-08	0,550131082	1,464218727	0,008555133	1,934959731
BSU09070	YhcG	0,160117558	1,117378184	0,104033134	0,938791399	1,916921687	9,71E-06	1,517149935
BSU09080	YhcH	0,256948495	1,194948544	0,098457606	0,756857519	1,689805875	0,000214548	1,442377209
BSU09090	YhcI	0,754546841	1,68710158	0,015906743	0,544831899	1,458850351	0,076678999	1,572975965
BSU09100	CspB	2,100665024	4,289070481	4,35E-14	-0,49037938	0,711837882	0,072218679	2,500454182
BSU09110	YhcJ	1,074000472	2,105262998	0,00011315	0,242186134	1,182783591	0,188519587	1,644023294
BSU09120	YhcK	-1,38317731	0,383373545	3,69E-07	-0,53829199	0,688585644	0,049485674	0,535979595
BSU09130	TcyP	-1,81190595	0,28481441	4,79E-10	0,315085078	1,244085017	0,14388392	0,764449713
**BSU09140**	**YhcM**	**-2,02361643**	**0,245940898**	**7,22E-13**	**-2,15959998**	**0,223818319**	**3,92E-14**	**0,234879609**
**BSU09150**	**YhcN**	**-2,46934294**	**0,18057337**	**1,80E-16**	**-2,18051478**	**0,220597023**	**9,48E-14**	**0,200585197**
BSU09165	YhcO	-0,84548997	0,556521771	0,002370757	-0,60685781	0,656625276	4,20E-05	0,606573524
**BSU09180**	**YhcQ**	**-3,11732295**	**0,115237091**	**9,19E-19**	**-2,55894903**	**0,169699118**	**2,64E-15**	**0,142468105**
BSU09190	YhcR	0,375471083	1,297263081	0,013235765	0,799740433	1,740787899	0,000106034	1,51902549
BSU09200	YhcS	0,428689593	1,346010433	0,00028709	0,488425269	1,402912732	0,022671331	1,374461583
**BSU09210**	**YhcT**	**-1,80167227**	**0,286841909**	**2,19E-11**	**-1,52827503**	**0,346691644**	**1,13E-09**	**0,316766777**
BSU09220	YhcU	0,118655643	1,085722674	0,065717889	-0,26401371	0,832767859	0,446589299	0,959245267
**BSU09230**	**YhcV**	**-2,74434044**	**0,149235179**	**1,79E-16**	**-3,26535989**	**0,103998895**	**7,95E-19**	**0,126617037**
BSU09240	YhcW	-0,73153346	0,60226342	0,000351199	-0,39878807	0,758495189	0,097551159	0,680379305
BSU09250	YhcX	-0,71966584	0,607238075	0,000793544	-0,75809472	0,591276681	0,003445414	0,599257378
BSU09260	YhxA	-0,11785804	0,921554861	0,654833469	0,310573814	1,240200877	0,082933449	1,080877869
BSU09270	GlpP	-0,16344908	0,892887874	0,087095094	0,266039964	1,202502559	0,116990189	1,047695217
BSU09280	GlpF	0,709083375	1,63476513	0,000154975	1,324740621	2,50487849	1,91E-09	2,06982181
BSU09290	GlpK	1,435950592	2,705603801	1,86E-08	0,882151683	1,843122146	2,84E-06	2,274362973
BSU09300	GlpD	0,476887663	1,391738018	0,038955836	0,421734726	1,339537274	0,079334477	1,365637646
BSU09310	PgcA	-0,08302699	0,944074759	0,046802237	-0,67934807	0,624447389	0,005609387	0,784261074

BSU09320	YhcY	-0,1523888	0,899759417	0,360150908	0,061887512	1,043830536	0,493353492	0,971794976
BSU09330	YhcZ	-0,33782772	0,791231784	0,239442261	-0,12138659	0,919303673	0,261371412	0,855267729
BSU09340	YhdA	-0,92879948	0,525295279	3,53E-05	-0,11130328	0,925751396	0,45460492	0,725523337
**BSU09350**	**YhdB**	**-3,17079224**	**0,11104434**	**5,41E-18**	**-3,06942538**	**0,119127188**	**3,87E-18**	**0,115085764**
**BSU09360**	**YhdC**	**-2,21167498**	**0,21588352**	**1,43E-13**	**-1,21001355**	**0,432264556**	**5,62E-07**	**0,324074038**
BSU09370	LytF	0,102991871	1,07399842	0,034787151	-1,37132654	0,386535671	4,33E-09	0,730267046
BSU09380	NsrR	0,196087216	1,14558715	0,142755579	-0,66087186	0,632495945	0,006492747	0,889041548
BSU09390	YgxB	1,397828024	2,635045774	9,97E-10	0,688276212	1,611357056	0,001269186	2,123201415
BSU09400	SpoVR	-0,2760089	0,825872568	0,070291195	-0,1654652	0,891640961	0,849299438	0,858756765
**BSU09410**	**PhoA**	**-1,8672678**	**0,274092015**	**1,02E-09**	**-2,01987321**	**0,246579845**	**6,31E-13**	**0,26033593**
BSU09420	LytE	0,949586105	1,931318502	0,000210526	-0,01888834	0,986992933	0,197869425	1,459155718
BSU09430	CitR	-0,59036996	0,664172566	0,071111851	0,080695758	1,057527924	0,30053343	0,860850245
BSU09440	CitA	-0,14352601	0,905303845	0,14493688	-0,03964684	0,972893075	0,050971722	0,93909846
BSU09450	YhdF	0,530843447	1,44477361	0,009395614	-0,01058747	0,992688187	0,166804236	1,218730899
BSU09460	BcaP	0,692572547	1,616162818	0,000677353	-0,6035042	0,658153408	0,033112171	1,137158113
BSU09470	YhdH	0,871989068	1,830184468	3,59E-06	0,402711366	1,321990094	0,06607135	1,576087281
BSU09480	YhdI	-0,69340182	0,618393979	0,007423064	-0,14041458	0,907258406	0,052537381	0,762826192
BSU09490	YhdJ	-0,76770416	0,587351415	0,000257646	0,113781019	1,08206039	0,585567049	0,834705903
BSU09500	YhdK	0,596827135	1,51238677	0,002055758	-0,18964201	0,876823272	0,166509751	1,194605021
BSU09510	YhdL	-0,04351963	0,970284929	0,572300244	-0,1615693	0,894052031	0,335599386	0,93216848
BSU09520	SigM	-0,25342182	0,838904318	0,11799441	0,179163431	1,132227155	0,000275835	0,985565736
**BSU09530**	**YhdN**	**2,069847048**	**4,198421601**	**6,45E-13**	**1,006286751**	**2,008734305**	**1,02E-05**	**3,103577953**
BSU09540	PlsC	1,141631846	2,206304392	4,62E-08	-0,00744979	0,994849507	0,803208388	1,60057695
BSU09550	YhdP	-0,38315544	0,766758715	0,070183234	0,411690453	1,330243591	0,009481131	1,048501153
BSU09560	CueR	-0,28443939	0,821060597	0,01142089	0,725580942	1,653566357	0,000529003	1,237313477
BSU09570	YhdR	0,095082522	1,068126503	0,07931415	0,135407138	1,098402746	0,304967485	1,083264625
BSU09590	YhdT	-0,36754026	0,775102892	0,020637353	-0,32027717	0,800915994	0,378342464	0,788009443
BSU09600	YhdU	1,001659511	2,002301895	9,95E-07	0,170333447	1,125318547	0,12207549	1,563810221
BSU09610	YhdV	1,837941829	3,574996499	2,39E-11	0,66103846	1,581220385	0,001944782	2,578108442

BSU09620	YhdW	-0,35515949	0,781783215	0,225838457	-0,01390607	0,990407351	0,790311486	0,886095283
BSU09630	YhdX	0,827263372	1,774316492	1,40E-05	-0,74640445	0,596087305	0,00010781	1,185201899
BSU09640	YhdY	-0,75276986	0,59346306	0,000359208	-0,03216726	0,977950084	0,835998868	0,785706572
BSU09650	SrtN	-0,10427448	0,930272655	0,044297086	-0,28964616	0,818102683	0,176989686	0,874187669
BSU09660	YheN	1,077527495	2,110416128	1,26E-05	0,141409353	1,102982081	0,574007812	1,606699104
BSU09670	Dat	0,329663187	1,256719945	0,00073134	-0,31451196	0,804122969	0,558768049	1,030421457
BSU09680	NhaC	0,975993592	1,966995412	2,88E-08	-0,05500453	0,96259142	0,68913431	1,464793416
BSU09690	NhaX	2,761560957	6,781295709	4,96E-17	0,49329075	1,407652035	0,005808104	4,094473872
BSU09700	YheJ	2,466070869	5,525369207	1,24E-16	-1,5599082	0,339172663	1,25E-09	2,932270935
BSU09710	BmrC	-1,40587328	0,377389639	1,19E-07	0,584013351	1,499013474	0,003136671	0,938201557
BSU09720	BmrD	-0,81553392	0,568198168	0,000881725	0,277065356	1,211727552	0,136162394	0,88996286
BSU09730	YheG	0,173547944	1,127828687	0,128334755	0,174034481	1,128209102	0,269891535	1,128018894
**BSU09740**	**YheF**	**-1,69564144**	**0,30871737**	**1,61E-07**	**-2,01173717**	**0,247974355**	**3,95E-10**	**0,278345863**
**BSU09750**	**SspB**	**-3,46610832**	**0,090489342**	**1,62E-19**	**-2,80945076**	**0,142649761**	**1,91E-16**	**0,116569552**
BSU09760	YheE	0,091491067	1,065470808	0,290163775	-0,76247952	0,589482333	0,000795789	0,827476571
**BSU09770**	**YheD**	**-2,58764953**	**0,166356538**	**1,66E-16**	**-1,020961**	**0,492787989**	**0,000476043**	**0,329572263**
**BSU09780**	**YheC**	**-2,64979108**	**0,159343151**	**3,29E-17**	**-0,8127941**	**0,569278258**	**0,030256822**	**0,364310705**
BSU09790	YheB	-0,86092219	0,550600496	1,15E-06	-0,37331488	0,772006619	0,001782936	0,661303557
**BSU09800**	**YheA**	**-1,8853616**	**0,27067591**	**2,96E-12**	**-1,3564909**	**0,390531034**	**6,50E-07**	**0,330603472**
BSU09810	YhaZ	-1,32722711	0,398533497	2,52E-07	-0,47493813	0,719497646	0,04110951	0,559015571
BSU09830	YhaX	0,110556417	1,079644553	0,232996945	-0,66728485	0,629690652	0,001488636	0,854667602
BSU09840	HemZ	-0,85159133	0,554173133	0,00124807	-0,41363631	0,750728778	0,102030817	0,652450955
BSU09850	YhaU	0,052435618	1,037014178	0,217086631	-1,01377776	0,495247721	2,66E-05	0,766130949
BSU09860	YhaT	-0,6863495	0,621424273	0,006427282	-0,73384884	0,601297625	0,002376746	0,611360949
BSU09870	YhaS	-0,48547376	0,714262478	0,049076177	-1,31933357	0,400720003	1,25E-07	0,557491241
BSU09880	YhaR	1,209573473	2,312692528	4,67E-08	0,598696489	1,514347697	0,003335214	1,913520113
BSU09889	YhzD	-0,41914826	0,747866021	0,088547726	-1,45110254	0,365741809	5,14E-09	0,556803915
BSU09890	YhaQ	-0,90337929	0,534632968	8,43E-05	-0,00680922	0,995291328	0,752659418	0,764962148
BSU09900	YhaP	-0,44052629	0,736865753	0,075612533	-0,52047479	0,697142365	0,028592209	0,717004059

BSU09910	YhaO	-0,32226243	0,799814628	0,211992764	0,259407598	1,196987095	0,046792224	0,998400861
BSU09920	SbcE	-1,36114738	0,389272577	2,91E-08	-0,52301109	0,695917845	0,073017572	0,542595211
BSU09930	YhaM	0,091729805	1,065647138	0,064929513	-0,44827275	0,732919802	0,121608149	0,89928347
BSU09940	YhaL	1,710418432	3,272557253	2,21E-11	-0,6580056	0,633753801	0,021930373	1,953155527
BSU09950	PrsA	0,291000155	1,223488172	0,012536519	-0,08412783	0,94335466	0,898241517	1,083421416
BSU09958	SscA	-0,54579188	0,6850153	0,039962155	-1,35672407	0,390467922	3,03E-07	0,537741611
BSU09965	YhaJ	-0,84385388	0,557153251	0,000278558	-1,07639022	0,474213877	4,74E-07	0,515683564
BSU09980	YhaI	0,917468384	1,888797954	3,05E-05	0,024812183	1,01734724	0,511179468	1,453072597
BSU09990	ScoC	-0,18531177	0,879459	0,324486057	-0,40748283	0,753937675	0,119025622	0,816698338
BSU10000	YhaH	1,06100361	2,086382408	2,82E-07	0,032215832	1,022581501	0,488636548	1,554481955
BSU10009	YhzF	1,047012532	2,066246724	2,75E-06	-0,63522918	0,643838531	0,004873752	1,355042628
BSU10010	TrpP	0,914645251	1,885105481	1,16E-05	-0,73481017	0,600897085	0,004254838	1,243001283
BSU10020	SerC	-0,08536835	0,942543855	0,313434154	0,147683911	1,107789608	0,330956622	1,025166732
BSU10030	Hit	-0,10765988	0,928092254	0,070158103	-0,38541614	0,765558147	0,083713748	0,846825201
BSU10040	EcsA	-0,39794602	0,758938021	0,074927107	-0,13610983	0,909969549	0,728083408	0,834453785
BSU10050	EcsB	-0,15377271	0,898896731	0,628369902	-0,16786621	0,890158278	0,507479838	0,894527505
BSU10060	EcsC	-0,23988126	0,846815007	0,154326087	-0,04904033	0,96657908	0,262274739	0,906697043
BSU10070	YhaA	0,471115228	1,386180595	2,69E-05	0,175278077	1,129182032	0,118893208	1,257681313
**BSU10080**	**YhfA**	**-2,48598739**	**0,178502058**	**4,94E-15**	**-2,31305518**	**0,201233838**	**2,17E-14**	**0,189867948**
BSU10090	YhgB	-1,08249909	0,472210134	0,000943955	-0,43645018	0,738950599	0,004508284	0,605580367
BSU10100	HmoB	0,207099977	1,154365411	0,116343568	-0,77336728	0,585050359	0,002353856	0,869707885
BSU10110	PbpF	-2,2575533	0,209126341	4,13E-14	-0,63606371	0,643466208	0,016026451	0,426296274
BSU10120	HemE	-1,20356713	0,434200373	2,66E-06	-0,16276093	0,893313872	0,642499821	0,663757123
BSU10130	HemH	-0,81417128	0,56873509	0,00107539	-0,183077	0,880822363	0,640017569	0,724778727
BSU10140	HemY	-0,13798011	0,908790648	0,067724237	-0,26317977	0,833249373	0,146959248	0,871020011
BSU10150	YhgD	0,129825354	1,094161239	0,111226695	0,164236502	1,120572896	0,250512803	1,107367068
BSU10160	YhgE	-0,96006738	0,514032905	0,000875856	0,138743664	1,100945966	0,541744064	0,807489436
BSU10170	FabHB	-0,3817675	0,767496725	0,010093113	0,340105284	1,265848969	0,126922767	1,016672847
BSU10180	YhfC	0,300620005	1,231673617	0,056649115	0,523560499	1,437498548	0,000394198	1,334586082

BSU10200	YhfE	0,075021652	1,053376845	0,232580347	1,085027785	2,121416339	7,89E-07	1,587396592
BSU10210	YhfF	0,744147849	1,67498462	0,000216372	0,163570627	1,120055815	0,046600399	1,397520217
BSU10220	GltT	1,755654848	3,376795585	4,74E-11	0,752091487	1,684232707	0,000473037	2,530514146
BSU10230	YhfH	1,160829444	2,235859362	8,93E-07	-0,50987172	0,702284882	0,004319815	1,469072122
BSU10240	YhfI	-0,14427396	0,904834622	0,302833997	0,205419016	1,153021182	0,06642617	1,028927902
BSU10250	LplJ	-0,42025421	0,747292936	0,05400709	0,297297991	1,228840773	0,141854236	0,988066855
BSU10260	YhfK	0,269151654	1,205098986	0,080298094	-0,99199532	0,50278192	6,32E-05	0,853940453
BSU10270	LcfB	1,539991595	2,907928093	4,98E-11	0,864901447	1,821215253	2,27E-05	2,364571673
**BSU10280**	**YhfM**	**-1,47575089**	**0,35954621**	**2,92E-09**	**-1,1875662**	**0,439042894**	**2,44E-07**	**0,399294552**
BSU10290	YhfN	0,783470843	1,721266929	2,14E-05	0,707570058	1,633051239	9,25E-05	1,677159084
BSU10300	AprE	0,649198378	1,568296542	0,001050484	0,351214258	1,275633825	0,091596434	1,421965184
BSU10310	YhfO	-0,05464794	0,962829373	0,38830704	0,082688952	1,05898999	0,513876007	1,010909681
BSU10320	YhfP	0,517816516	1,431786633	0,010040744	-0,2271449	0,854323931	0,337619635	1,143055282
BSU10330	YhfQ	0,288836299	1,221654474	0,039270561	0,522630113	1,436571812	0,003372064	1,329113143
BSU10340	YhfR	0,44976386	1,365816682	0,000380525	0,63469956	1,552614386	0,000794498	1,459215534
BSU10350	YhfS	0,672343753	1,593659871	2,74E-05	1,036868982	2,051769951	4,12E-06	1,822714911
BSU10360	YhfT	-0,00326648	0,997738413	0,196999089	1,015888629	2,022148053	6,21E-06	1,509943233
BSU10370	YhfU	0,269362786	1,20527536	0,191514675	0,545992178	1,460024095	0,002280216	1,332649728
BSU10380	HemAT	-0,77494651	0,584410293	0,000768317	-1,08387626	0,471759588	5,13E-06	0,528084941
BSU10390	YhfW	-1,20175538	0,434745989	4,41E-07	-0,58791112	0,665305509	0,009344086	0,550025749
**BSU10400**	**YhxC**	**-1,44521315**	**0,367237897**	**2,45E-08**	**-1,28369439**	**0,410742351**	**5,49E-07**	**0,388990124**
BSU10410	YhzC	0,573309414	1,487932844	0,000135816	-1,54320157	0,343123165	1,74E-09	0,915528005
BSU10420	ComK	-0,48029035	0,716833342	0,004439014	-1,06728831	0,477215129	3,36E-05	0,597024236
BSU10430	YhxD	0,677522182	1,599390454	0,000360414	0,144798081	1,105575906	0,262082625	1,35248318
BSU10440	YhjA	0,700908202	1,625527769	7,16E-05	-0,26196069	0,833953769	0,10782584	1,229740769
BSU10450	YhjB	1,589741351	3,009953817	2,55E-10	0,688170909	1,611239446	0,002115529	2,310596631
BSU10460	YhjC	0,935024665	1,911923322	2,50E-05	0,37579372	1,297553226	0,023891333	1,604738274
BSU10470	YhjD	-1,92256041	0,263785943	8,61E-09	-0,30822509	0,807634763	0,072199764	0,535710353
BSU10480	YhjE	-0,44897122	0,732565053	0,0455622	-0,47765646	0,718143244	0,083211991	0,725354148

BSU10490	SipV	-0,70784215	0,61223518	0,012726322	-0,51003728	0,70220429	0,025022321	0,657219735
BSU10500	YhjG	-0,42568625	0,744484518	0,151645895	0,455029326	1,37081067	0,053697256	1,057647594
BSU10510	YhjH	-0,28832897	0,818849957	0,00285648	0,885860451	1,847866395	0,009753187	1,333358176
BSU10520	GlcP	0,078205309	1,055703943	0,507592085	0,00796934	1,00553921	0,456565862	1,030621577
BSU10530	NtdC	-1,03763323	0,487125958	0,002016591	-0,37163257	0,772907373	0,104691247	0,630016665
BSU10540	NtdB	-0,15136154	0,900400311	0,192181999	0,310715656	1,240322816	0,333404237	1,070361563
BSU10550	NtdA	0,563678105	1,478032615	0,012415915	0,307543944	1,237599007	0,218798176	1,357815811
BSU10560	NtdR	-0,03084376	0,978847651	0,055444689	0,074921479	1,053303707	0,383547971	1,016075679
BSU10570	YhjN	0,614485838	1,531012262	0,002220979	-1,05363571	0,481752577	3,06E-05	1,00638242
BSU10580	YhjO	-0,16092438	0,894451786	0,967608865	0,080935539	1,057703703	0,60004838	0,976077745
BSU10590	YhjP	0,386491374	1,307210406	0,164873666	0,031891023	1,022351302	0,63630193	1,164780854
**BSU10600**	**YhjQ**	**-1,90780681**	**0,266497368**	**7,39E-11**	**-1,75600149**	**0,296067597**	**1,84E-10**	**0,281282482**
**BSU10610**	**YhjR**	**-1,76740095**	**0,293737436**	**2,85E-11**	**-2,28018045**	**0,205872003**	**2,25E-14**	**0,249804719**
BSU10620	AddB	1,067842038	2,096295413	1,55E-06	1,149848941	2,2189066	2,14E-05	2,157601006
BSU10630	AddA	-0,02819347	0,980647491	0,44447059	1,049606422	2,069965069	7,31E-05	1,52530628
BSU10640	SbcD	0,722117736	1,649601713	0,003576757	0,780046457	1,717186168	6,73E-07	1,683393941
BSU10650	SbcC	-1,74837312	0,297637226	4,96E-09	-0,85831731	0,551595538	8,34E-05	0,424616382
BSU10660	YisB	-1,2689406	0,414964378	3,42E-07	-1,05115786	0,482580707	8,61E-07	0,448772542
**BSU10670**	**GerPF**	**-1,74290659**	**0,298767145**	**1,88E-10**	**-2,66836281**	**0,157305082**	**1,39E-15**	**0,228036114**
**BSU10680**	**GerPE**	**-1,66181183**	**0,316041994**	**1,74E-11**	**-2,4834532**	**0,178815884**	**1,97E-15**	**0,247428939**
**BSU10690**	**GerPD**	**-2,41860521**	**0,187036895**	**2,14E-15**	**-2,35860849**	**0,194979116**	**5,08E-15**	**0,191008005**
**BSU10700**	**GerPC**	**-2,73307819**	**0,150404727**	**2,22E-17**	**-2,28395379**	**0,205334252**	**1,73E-14**	**0,177869489**
**BSU10710**	**GerPB**	**-2,67270955**	**0,156831846**	**4,25E-15**	**-2,13219214**	**0,22811099**	**4,51E-14**	**0,192471418**
**BSU10720**	**GerPA**	**-2,50284142**	**0,176428872**	**9,47E-16**	**-2,47634324**	**0,179699309**	**2,18E-15**	**0,17806409**
BSU10730	YisI	-0,76173121	0,589788172	0,00191205	-0,70387138	0,613922572	0,002020831	0,601855372
**BSU10740**	**YisJ**	**-2,37757416**	**0,192432694**	**1,07E-12**	**-1,95300375**	**0,258277925**	**1,35E-12**	**0,22535531**
BSU10750	YisK	0,993076497	1,99042498	9,91E-07	0,8774332	1,837103871	1,08E-05	1,913764426
BSU10760	YisL	1,562079407	2,952791331	2,94E-08	0,737637356	1,667442897	0,002017559	2,310117114
BSU10770	WprA	1,054748399	2,077355892	1,06E-06	0,96126012	1,947009766	1,86E-05	2,012182829

BSU10780	YisN	0,718542325	1,64551859	0,000574422	-0,07756357	0,947656706	0,828225352	1,296587648
**BSU10790**	**AsnO**	**-2,60988374**	**0,163812376**	**3,48E-16**	**-1,19289843**	**0,437423178**	**3,19E-06**	**0,300617777**
BSU10800	YizA	-0,6115969	0,654471875	0,007241655	0,009024646	1,006275014	0,767628801	0,830373444
BSU10810	YisP	0,125655942	1,091003661	0,387609488	0,323568357	1,251421987	0,046667129	1,171212824
BSU10820	YisQ	-0,7657389	0,588152061	0,531652552	0,055214391	1,039013494	0,384967574	0,813582777
BSU10830	YisR	-0,79198054	0,577550681	0,000984696	0,224225958	1,168150339	0,17258824	0,87285051
BSU10840	DegA	-0,19084442	0,87609279	0,520767352	-0,09126115	0,938701813	0,704932524	0,907397301
BSU10850	IolX	0,462411766	1,377843247	0,068059427	0,362245689	1,285425225	0,351539173	1,331634236
BSU10860	YisT	0,64144942	1,559895539	0,005243616	0,238079092	1,17942125	0,262490314	1,369658395
BSU10870	YisU	0,145744835	1,106301668	0,187539681	0,219597142	1,164408392	0,592581927	1,13535503
BSU10880	YisV	0,02031893	1,014183656	0,000490253	0,713272782	1,639519184	0,001657963	1,32685142
BSU10890	YisX	-0,44459331	0,734791423	0,039612854	-0,03420409	0,97657037	0,587416002	0,855680896
BSU10900	YisY	-1,01738523	0,494010896	9,07E-07	-0,80797268	0,571183941	0,000143803	0,532597418
**BSU10910**	**YisZ**	**-1,73622962**	**0,300153081**	**4,20E-12**	**-2,74480887**	**0,149186731**	**1,33E-16**	**0,224669906**
**BSU10920**	**YitA**	**-2,77914143**	**0,145678368**	**2,82E-16**	**-2,73681811**	**0,150015335**	**1,13E-16**	**0,147846851**
**BSU10930**	**YitB**	**-3,16007606**	**0,111872236**	**4,96E-16**	**-2,84397487**	**0,139276633**	**2,50E-16**	**0,125574434**
**BSU10940**	**YitC**	**-2,2036192**	**0,217092351**	**1,75E-10**	**-1,78683966**	**0,289806196**	**1,62E-09**	**0,253449274**
**BSU10950**	**YitD**	**-1,75002554**	**0,297296515**	**4,09E-10**	**-1,4815551**	**0,3581026**	**5,04E-10**	**0,327699558**
BSU10960	YitE	1,15777093	2,231124361	1,03E-06	0,056695413	1,040080658	0,588713711	1,635602509
**BSU10970**	**YitF**	**-3,03840333**	**0,121716501**	**7,58E-18**	**-2,16609539**	**0,222812891**	**7,28E-14**	**0,172264696**
**BSU10980**	**YitG**	**-3,66340546**	**0,07892327**	**4,72E-20**	**-1,75844937**	**0,295565674**	**1,57E-10**	**0,187244472**
BSU10990	YitH	-0,74189247	0,597954464	0,003934988	0,196375931	1,145816431	0,341347987	0,871885447
BSU11000	YitI	-0,64563281	0,639212351	0,014536584	-0,12260213	0,918529443	0,680078196	0,778870897
BSU11010	YitJ	-0,63550271	0,643716471	0,007294114	-0,14543591	0,904106163	0,602375659	0,773911317
BSU11020	YitK	0,465981397	1,381256637	0,004043008	-0,02886306	0,980192448	0,428107484	1,180724543
BSU11030	YitL	-0,34018317	0,789941014	0,172258279	0,029476341	1,020641594	0,549526023	0,905291304
BSU11040	YitM	-0,17004929	0,888812315	0,162834785	0,484816708	1,399408062	0,001939402	1,144110189
BSU11055	YitO	-1,29718397	0,4069197	9,20E-07	1,17864315	2,263637825	3,97E-07	1,335278762
BSU11070	YitP	-0,37964504	0,768626679	0,002046499	0,748968824	1,680591188	0,000327393	1,224608933

BSU11079	YizB	-0,50065825	0,706784228	0,014209225	-0,53131264	0,691924897	0,023074482	0,699354562
BSU11080	YitQ	-0,22170238	0,857552927	0,006344353	-0,38229217	0,767217659	0,110213641	0,812385293
BSU11090	YitR	0,335436187	1,261758834	0,118657129	-0,98095422	0,506644527	0,000139367	0,88420168
BSU11100	NprB	-0,08117467	0,945287662	0,053396905	0,936922265	1,91443976	0,00013247	1,429863711
BSU11110	YitS	-0,49694238	0,708606997	0,013079771	-0,93090305	0,524529913	0,000192417	0,616568455
BSU11120	YitT	-1,16624728	0,445578869	7,29E-06	-0,87387938	0,545677559	0,000145651	0,495628214
BSU11130	Ipi	-0,56521199	0,6758561	0,010880455	-0,39647835	0,75971049	0,015230432	0,717783295
**BSU11139**	**YizC**	**-1,96636913**	**0,255896243**	**1,60E-12**	**-2,50184635**	**0,176550603**	**6,17E-16**	**0,216223423**
BSU11140	YitU	-0,71233742	0,610330494	0,003260761	-0,28208035	0,822404267	0,288325936	0,716367381
BSU11150	YitV	-1,48166103	0,358076309	4,22E-08	-0,64224971	0,640713055	0,002207618	0,499394682
BSU11160	YitW	-0,12168691	0,919112324	0,020069136	-0,81600085	0,568014298	0,00111315	0,743563311
BSU11170	YitY	-0,83299949	0,561360909	0,00025198	-0,1280918	0,915040943	0,134363708	0,738200926
BSU11180	YitZ	0,804574144	1,746630144	0,000150672	-0,26826431	0,83031789	0,105391062	1,288474017
BSU11190	ArgC	-0,25793805	0,836282308	0,072682405	-0,73374395	0,601341342	0,003894101	0,718811825
BSU11200	ArgJ	0,447528558	1,363702133	0,026328128	-0,86244431	0,550019888	0,002030085	0,956861011
BSU11210	ArgB	-0,01886758	0,987007137	0,201571836	-0,42743107	0,743584671	0,181160996	0,865295904
BSU11220	ArgD	-0,13689649	0,909473501	0,226331132	-0,38260661	0,76705046	0,262672005	0,83826198
BSU11230	CarA	-0,20263536	0,868961788	0,041522884	-0,32094625	0,800544638	0,222831447	0,834753213
BSU11240	CarB	0,093305445	1,06681162	0,115850783	-0,39704055	0,759414498	0,120396028	0,913113059
BSU11250	ArgF	0,323904416	1,251713525	0,197724829	-0,49926971	0,707464809	0,054322563	0,979589167
**BSU11260**	**YjzC**	**-1,30313424**	**0,405244852**	**5,61E-09**	**-2,92509162**	**0,131661768**	**1,01E-17**	**0,26845331**
BSU11270	YjzD	1,397604017	2,634636663	6,72E-10	-0,38273831	0,766980441	0,172861262	1,700808552
BSU11280	YjaU	-0,58022906	0,668857573	0,00764746	-0,30646385	0,808621319	0,095549843	0,738739446
BSU11290	YjaV	-0,96017249	0,513995458	0,000190293	-1,16632663	0,445554363	8,78E-07	0,47977491
BSU11300	Med	-0,44669418	0,733722191	0,00317268	-0,32568708	0,79791829	0,028933927	0,765820241
BSU11310	ComZ	0,086171874	1,061549668	0,10594909	0,097446777	1,069878359	0,139629011	1,065714013
**BSU11320**	**YjzB**	**-1,28648873**	**0,409947558**	**4,00E-07**	**-1,60823107**	**0,328000275**	**2,19E-08**	**0,368973917**
BSU11330	FabHA	0,115146327	1,083084893	0,14382945	0,613606193	1,530079053	0,00295538	1,306581973
BSU11340	FabF	0,404868142	1,323967898	0,000168236	0,058415731	1,041321625	0,286385861	1,182644761

BSU11350	YjaZ	0,070297843	1,049933419	0,288592298	0,608240326	1,524398746	0,012187249	1,287166082
BSU11360	AppD	-0,18797433	0,877837417	0,214363486	0,056758698	1,040126283	0,276130873	0,95898185
BSU11370	AppF	0,194509148	1,144334754	0,256374847	-0,16193264	0,893826893	0,326097457	1,019080823
BSU11390	AppB	0,250647317	1,189740815	0,087524218	0,856533769	1,810682719	1,52E-05	1,500211767
BSU11400	AppC	0,56550962	1,479910183	1,41E-05	0,75749386	1,690551375	0,000754002	1,585230779
BSU11410	YjbA	0,577120413	1,491868539	0,002434138	-0,15389398	0,898821178	0,323597044	1,195344858
BSU11420	TrpS	-0,41307088	0,751023064	0,034411936	-0,30580273	0,808991963	0,28928573	0,780007513
BSU11430	OppA	0,056051952	1,039616872	0,00834489	-0,02663642	0,981706439	0,324659792	1,010661655
BSU11440	OppB	0,984730595	1,978943735	4,94E-06	1,109216086	2,157283956	2,06E-07	2,068113846
BSU11450	OppC	0,836826692	1,786117129	0,000126965	1,37089173	2,586303762	3,23E-09	2,186210446
BSU11460	OppD	0,813578912	1,757566056	1,87E-05	1,272945962	2,416545169	5,06E-08	2,087055613
BSU11470	OppF	1,980516986	3,946344727	2,95E-13	0,803161663	1,74492093	0,00073315	2,845632829
BSU11480	YjbB	0,727661273	1,65595248	0,000279738	0,454653246	1,370453375	0,010546264	1,513202928
BSU11490	YjbC	0,417318028	1,335442656	0,061611863	0,0867601	1,061982579	0,30108481	1,198712617
BSU11500	Spx	0,121768925	1,088068153	0,12760957	-0,3888006	0,763764302	0,161170432	0,925916227
BSU11510	YjbE	1,287070076	2,440319551	2,52E-08	0,688483346	1,611588422	0,001797207	2,025953987
BSU11520	MecA	0,494226036	1,408564898	0,012722951	-0,43977915	0,737247461	0,060080031	1,072906179
BSU11530	CoiA	0,284659693	1,218122895	0,001772919	-0,43888593	0,737704055	0,043994809	0,977913475
BSU11540	PepF	-0,3914455	0,762365372	0,000384732	-0,17653874	0,884823286	0,074698585	0,823594329
BSU11549	YizD	1,412353567	2,661710318	3,27E-11	-2,03811794	0,243481161	3,04E-13	1,452595739
BSU11550	YjbH	0,575042605	1,489721456	0,000416322	-0,53952743	0,687996232	0,00370627	1,088858844
BSU11560	YjbI	-0,03619483	0,975223755	0,009875036	-0,18479446	0,879774411	0,255717977	0,927499083
BSU11570	CwlQ	1,199816109	2,297103894	5,62E-07	-0,20324175	0,868596625	0,016035195	1,58285026
BSU11580	YjbK	-0,3408877	0,789555344	0,040305695	-0,29592065	0,814552361	0,162983002	0,802053853
BSU11590	YjbL	-1,19697285	0,436189562	1,33E-06	-0,09557953	0,935896224	0,466779199	0,686042893
BSU11600	YjbM	-0,73429428	0,601111999	0,003264586	-0,15128991	0,90044502	0,483766978	0,750778509
BSU11610	NadF	-0,18969368	0,876791864	0,012024841	-0,25092269	0,840358784	0,358193749	0,858575324
BSU11620	YjbO	-0,35295023	0,782981307	0,203302238	-0,09217993	0,938104189	0,788251663	0,860542748
BSU11630	PrpE	-1,18345266	0,440296519	5,50E-06	-1,01270682	0,49561549	5,73E-06	0,467956004

BSU11640	YjbQ	-2,24918977	0,210342201	1,99E-11	-0,52875024	0,693154931	0,027782657	0,451748566
**BSU11650**	**TenA**	**1,497127389**	**2,822800927**	**1,07E-09**	**1,127061437**	**2,184134101**	**1,25E-06**	**2,503467514**
BSU11660	TenI	1,314738024	2,487571552	1,13E-07	0,990335461	1,98664688	3,35E-07	2,237109216
**BSU11670**	**ThiO**	**1,619981464**	**3,07371087**	**2,35E-10**	**1,068425148**	**2,097142867**	**3,41E-07**	**2,585426869**
**BSU11680**	**ThiS**	**1,447007514**	**2,726419414**	**6,51E-11**	**1,296488347**	**2,456302676**	**2,27E-08**	**2,591361045**
**BSU11690**	**ThiG**	**1,628984651**	**3,092952442**	**2,39E-11**	**1,043066145**	**2,06060238**	**7,53E-07**	**2,576777411**
BSU11700	ThiF	1,921437081	3,788001969	2,85E-13	0,907119238	1,875297179	4,30E-05	2,831649574
BSU11710	ThiD	1,927838997	3,804848464	6,72E-14	0,94960171	1,931339392	3,44E-05	2,868093928
BSU11720	FabI	0,541383143	1,455367141	9,58E-05	0,048825706	1,034422604	0,637468637	1,244894872
BSU11730	CotO	-0,32758772	0,796867787	0,025618221	-2,44048784	0,184221348	6,83E-14	0,490544568
**BSU11740**	**CotZ**	**-1,14815872**	**0,451200723**	**1,06E-06**	**-1,37171903**	**0,386430525**	**3,31E-08**	**0,418815624**
**BSU11750**	**CotY**	**-1,17863024**	**0,441770736**	**9,18E-07**	**-1,54591223**	**0,342479079**	**1,39E-09**	**0,392124907**
**BSU11760**	**CotX**	**-1,75540437**	**0,296190162**	**3,14E-10**	**-1,49299564**	**0,355274085**	**2,64E-09**	**0,325732124**
**BSU11770**	**CotW**	**-2,26188568**	**0,208499282**	**7,61E-14**	**-2,12233841**	**0,229674341**	**5,47E-13**	**0,219086811**
**BSU11780**	**CotV**	**-2,31222381**	**0,201349835**	**2,32E-14**	**-1,94892142**	**0,259009799**	**9,41E-13**	**0,230179817**
BSU11790	YjcA	0,197270121	1,146526834	0,129480248	-0,58011725	0,668909414	0,009831983	0,907718124
BSU11799	YjzK	-1,3849824	0,382894171	4,05E-08	-0,70818595	0,6120893	0,001022126	0,497491736
BSU11809	YjcZ	-0,97893999	0,507352377	0,00010902	-1,14327271	0,452731403	6,05E-06	0,48004189
**BSU11810**	**SpoVIF**	**-1,90045125**	**0,267859571**	**2,70E-13**	**-1,94016847**	**0,260586009**	**6,57E-12**	**0,26422279**
BSU11820	YjcD	-0,27176991	0,828302753	0,069280284	-0,52463357	0,695135644	0,034186073	0,761719199
BSU11839	YjzE	1,173808769	2,256065211	3,73E-08	-0,07769226	0,947572177	0,702882859	1,601818694
BSU11840	YjcF	0,105428842	1,075814129	0,041392985	-0,42170029	0,746544265	0,132709818	0,911179197
BSU11850	YjcG	-0,17242077	0,887352498	0,296644143	-0,59457801	0,662238131	0,089839611	0,774795315
BSU11860	YjcH	0,405307208	1,324370892	0,154944767	-0,12543546	0,916727298	0,716089321	1,120549095
BSU11870	MetI	-0,7833994	0,580996185	8,40E-05	0,508973192	1,423037018	0,003321059	1,002016602
BSU11880	MetC	-0,86068769	0,550689996	0,001620822	0,351545342	1,275926604	0,055036601	0,9133083
BSU11890	YjcK	0,111459502	1,08032059	0,203913989	0,782603424	1,720232329	0,000165878	1,40027646
BSU11900	YjcL	1,178822108	2,263918633	4,62E-09	1,105926623	2,152370774	9,94E-08	2,208144704
BSU11910	YjcM	0,32676506	1,254197944	0,009600727	-0,08902203	0,940159843	0,284290796	1,097178894

BSU11920	YjcN	1,025209095	2,03525434	1,42E-05	-0,46796401	0,722984183	0,064028421	1,379119261
BSU11928	YjzF	-0,00141976	0,999016383	0,098399089	0,422176369	1,339947401	0,198378678	1,169481892
BSU11929	YjzG	0,450200733	1,366230337	0,018430487	-0,21899285	0,859165011	0,328386718	1,112697674
BSU11930	YjcO	0,602620798	1,518472518	0,00204102	0,360493918	1,283865364	0,138662469	1,401168941
BSU11940	YjcP	1,33892745	2,529631873	6,03E-09	-0,5233252	0,695766346	0,015690555	1,612699109
BSU11950	YjcQ	1,212024089	2,316624293	6,01E-05	-0,0461586	0,968511712	0,50352822	1,642568003
BSU11960	YjcR	0,596084495	1,511608456	0,028206275	-0,07523567	0,949187054	0,855045192	1,230397755
BSU11970	YjcS	-1,22139472	0,428867913	1,02E-06	-0,1353982	0,910418516	0,350691211	0,669643214
BSU11980	YjdA	-0,97035536	0,510380331	0,000358014	-0,24043664	0,846489081	0,151417196	0,678434706
BSU11990	YjdB	1,576271384	2,981981658	7,06E-09	-0,00577389	0,996005843	0,846027965	1,98899375
BSU12000	ManR	-1,32825362	0,398250033	0,014305755	0,502776417	1,416937788	0,024077153	0,90759391
BSU12010	ManP	-1,14350874	0,452657344	6,12E-06	0,399582512	1,319126126	0,017826806	0,885891735
BSU12020	ManA	-0,68541967	0,621824918	0,006654116	0,394802919	1,314763143	0,024890173	0,968294031
BSU12030	YjdF	-0,19726939	0,872199829	0,189575964	0,216695805	1,162069057	0,131813426	1,017134443
**BSU12040**	**YjdG**	**-1,59601713**	**0,330788932**	**6,92E-11**	**-1,4286455**	**0,371479499**	**1,13E-08**	**0,351134215**
**BSU12050**	**YjdH**	**-2,00751918**	**0,248700414**	**1,68E-13**	**-1,72352097**	**0,302808799**	**3,06E-10**	**0,275754607**
BSU12060	YjdI	0,595306166	1,51079317	0,00068404	-0,22289691	0,856843181	0,136680506	1,183818175
BSU12069	YjzH	-0,17230773	0,887422031	0,264596118	-0,77681698	0,583653087	0,000743658	0,735537559
BSU12070	YjdJ	-0,59344105	0,662760236	0,014304733	-0,56064155	0,678000598	0,015500041	0,670380417
BSU12080	CtaO	-0,64076851	0,641371206	0,015066466	-0,63432388	0,644242669	0,0002868	0,642806937
**BSU12090**	**CotT**	**-3,28422749**	**0,10264765**	**1,56E-18**	**-2,95612031**	**0,128860294**	**1,32E-17**	**0,115753972**
BSU12100	YjeA	-1,98260128	0,253033223	0,002750347	-0,79361327	0,576897426	0,006806467	0,414965324
**BSU12110**	**YjfA**	**-1,87289627**	**0,273024766**	**2,14E-13**	**-1,09291186**	**0,46881419**	**8,21E-05**	**0,370919478**
BSU12120	YjfB	0,591880795	1,507210369	0,000189612	-1,29855297	0,40653375	1,19E-07	0,95687206
BSU12130	YjfC	-0,95735138	0,515001528	0,201149013	-1,10480424	0,464965559	0,019188312	0,489983543
BSU12140	YjgA	1,040659166	2,057167357	0,001140596	0,125898531	1,091187128	0,000252284	1,574177242
BSU12150	YjgB	0,173231625	1,127581431	0,129085752	-0,00274715	0,998097635	0,460599601	1,062839533
BSU12160	YjgC	0,712213488	1,638315814	0,001506407	0,623294783	1,540389061	0,006405869	1,589352437
BSU12170	YjgD	1,057336381	2,081085706	1,83E-06	0,394095105	1,314118254	0,028537276	1,69760198

BSU12180	YjhA	-0,78860076	0,578905288	0,000198261	-0,25170607	0,839902597	0,304376887	0,709403943
BSU12190	YjhB	0,469607515	1,3847327	0,000518241	-0,17470154	0,88595078	0,190809916	1,13534174
BSU12200	YjiA	0,045643169	1,032143217	0,421187744	-0,70394042	0,613893196	0,002043676	0,823018206
BSU12210	YjiB	0,169019756	1,124294319	0,060216659	0,488196778	1,402690559	0,000628568	1,263492439
BSU12220	YjiC	0,693359745	1,617044908	3,85E-05	0,739222758	1,669276285	0,001379301	1,643160597
BSU12229	YjzI	0,551614803	1,465725359	1,29E-05	-0,31087418	0,806153133	0,340704707	1,135939246
BSU12230	YjjA	0,382470729	1,303572415	0,003490441	-0,2736294	0,827235844	0,162495269	1,06540413
BSU12240	YjkA	0,994907445	1,992952665	0,00034622	0,810945693	1,754361058	0,004024686	1,873656861
BSU12250	YjkB	-0,04170709	0,971504718	0,040504492	0,784632602	1,722653571	0,006306637	1,347079144
BSU12260	YjlA	0,745455897	1,676503968	0,001915888	0,324049459	1,251839374	0,11527391	1,464171671
BSU12270	YjlB	0,469941044	1,385052866	0,024267197	-0,02570043	0,982343554	0,756071517	1,18369821
BSU12280	YjlC	-0,52313019	0,695860396	0,017771728	0,191647281	1,142066993	0,334636053	0,918963695
BSU12290	Ndh	0,116251018	1,083914544	0,347867171	0,050620881	1,035710559	0,559349144	1,059812551
BSU12300	UxaC	0,718296915	1,645238703	0,015489263	1,633936672	3,103587181	2,06E-06	2,374412942
BSU12310	YjmB	-0,72891751	0,603356457	0,036892481	0,987207609	1,982344372	0,00280498	1,292850414
BSU12320	YjmC	-0,02429269	0,983302565	0,507741659	0,374565985	1,296449477	0,002531618	1,139876021
BSU12330	YjmD	0,054816284	1,038726821	0,199269598	0,895756766	1,8605856	3,68E-06	1,449656211
BSU12340	UxuA	0,114132882	1,082324329	0,336179074	1,015987216	2,022286242	1,25E-06	1,552305286
BSU12350	YjmF	0,274837636	1,209857921	0,236805244	0,643464325	1,562075652	0,000327943	1,385966786
BSU12360	ExuT	0,32561279	1,253196625	0,001878448	0,681183776	1,6034549	0,000714043	1,428325763
BSU12370	ExuR	0,243732018	1,184051653	0,151636154	0,464718006	1,380047579	0,013823838	1,282049616
BSU12380	UxaB	-0,89491547	0,537778705	0,002012653	0,143882762	1,104874696	0,313520047	0,8213267
BSU12390	UxaA	-0,26111016	0,834445565	0,330087643	0,015697809	1,010940304	0,448589477	0,922692934
BSU12400	YjnA	-1,01967493	0,493227475	4,23E-05	-1,56471085	0,338045454	2,37E-09	0,415636465
BSU12410	YjoA	-0,75904556	0,590887111	0,000185777	-0,71058774	0,611071142	0,001015558	0,600979127
BSU12420	YjoB	-0,40194601	0,756836721	0,499978507	-0,42997197	0,742276209	0,292962394	0,749556465
BSU12430	RapA	0,239393622	1,180496383	0,312588026	0,748112922	1,679594445	3,38E-08	1,430045414
BSU12440	PhrA	0,509332516	1,42339149	0,015027795	0,440612464	1,357180365	5,50E-05	1,390285928
BSU12450	YjpA	0,084977641	1,060671303	0,136081765	-0,37704287	0,770014292	0,06295319	0,915342797

**BSU12460**	**XlyB**	**2,544853036**	**5,835486898**	**1,40E-11**	**1,753777255**	**3,372403717**	**3,06E-09**	**4,603945307**
BSU12470	YjqA	0,634790334	1,552712079	0,010653879	0,506239835	1,420343458	0,248717563	1,486527768
BSU12480	YjqB	0,292484861	1,224747936	0,488319687	1,011735109	2,01633466	0,001741126	1,620541298
BSU12490	YjqC	0,126724686	1,091812172	0,170026756	-0,35880333	0,779811143	0,062683187	0,935811658
BSU12500	XkdA	-0,07037411	0,952390999	0,00050927	-0,1561017	0,897446783	0,130978317	0,924918891
BSU12510	Xre	-0,08400034	0,943438028	0,139124474	-0,33752865	0,791395818	0,135182576	0,867416923
BSU12519	YjzJ	1,522550317	2,872984714	3,09E-07	0,332047925	1,258798989	0,092288007	2,065891852
BSU12520	XkdB	0,487534505	1,402046798	0,232598997	1,387896537	2,61696846	0,000711157	2,009507629
BSU12530	XkdC	1,824244885	3,541216098	2,54E-05	3,552373728	11,73197281	1,80E-13	7,636594456
BSU12539	YkzK	1,760243189	3,387552227	8,91E-07	1,846353342	3,595901099	1,46E-09	3,491726663
BSU12540	XkdD	-0,27604147	0,825853928	0,128948862	1,189183907	2,280237199	2,50E-05	1,553045564
BSU12550	XtrA	1,752615597	3,369689351	2,33E-11	0,183784545	1,13585962	0,222559395	2,252774486
BSU12560	Xpf	0,819043739	1,764236217	0,065694755	1,520449581	2,868804351	1,47E-06	2,316520284
BSU12570	XtmA	1,986740356	3,96340489	1,03E-12	2,838234597	7,151444103	1,16E-17	5,557424496
BSU12580	XtmB	1,807981003	3,501519211	1,00E-11	3,13067921	8,758472065	2,16E-17	6,129995638
BSU12590	XkdE	1,801748426	3,486424949	1,06E-11	2,895557831	7,441316238	1,63E-17	5,463870594
BSU12600	XkdF	2,622536415	6,158318208	3,84E-16	3,438785411	10,84370159	1,94E-19	8,501009897
BSU12610	XkdG	2,036466131	4,10239421	8,79E-13	3,450754403	10,93403811	8,42E-20	7,518216157
BSU12619	YkzL	2,090482621	4,258905212	9,01E-13	3,760336934	13,55108942	4,00E-20	8,904997315
BSU12620	XkdH	2,277183848	4,847308314	1,86E-14	3,448032282	10,91342687	7,55E-20	7,880367594
BSU12630	XkdI	1,99753291	3,993165618	1,30E-12	3,568916039	11,86726881	4,00E-20	7,930217212
BSU12640	XkdJ	2,13391114	4,389057406	9,17E-14	3,418068637	10,68910115	3,67E-19	7,53907928
BSU12649	YkzM	1,990014118	3,972408855	5,30E-13	3,563357842	11,82163635	7,80E-20	7,897022602
BSU12650	XkdK	2,002392921	4,006640091	5,96E-14	3,469600834	11,07781031	1,13E-19	7,542225198
BSU12660	XkdM	2,291307813	4,894996448	9,03E-14	3,367621397	10,32179084	2,06E-19	7,608393645
BSU12671	XkdN	2,172344527	4,507553226	4,84E-14	1,852347625	3,610872865	5,13E-13	4,059213045
BSU12672	XkzB	1,653814914	3,146646066	6,12E-07	3,874127141	14,66319048	1,44E-19	8,904918275
BSU12680	XkdO	1,527226375	2,882311729	3,85E-10	3,030196105	8,16920737	5,19E-18	5,525759549
BSU12690	XkdP	1,661529742	3,163517864	1,44E-09	3,556020468	11,76166556	3,31E-19	7,462591711

BSU12700	XkdQ	1,514109044	2,856223835	2,44E-07	3,313124159	9,939161589	3,00E-19	6,397692712
BSU12710	XkdR	1,522646048	2,87317536	6,76E-10	3,318180663	9,974058487	3,15E-19	6,423616924
BSU12720	XkdS	1,728603443	3,314068547	5,65E-11	3,448858172	10,9196762	2,24E-19	7,116872375
BSU12730	XkdT	1,682167073	3,209096278	5,46E-10	3,375458653	10,37801523	4,35E-19	6,793555754
BSU12740	XkdU	1,12493914	2,180923461	0,000109966	3,188533943	9,116840543	6,47E-19	5,648882002
BSU12749	XkzA	1,398230173	2,635780393	1,12E-08	3,330465287	10,05935074	2,83E-19	6,347565566
BSU12750	XkdV	0,699654969	1,624116327	0,000217551	3,191985679	9,13867926	1,27E-18	5,381397794
BSU12760	XkdW	1,267267632	2,407052539	3,43E-07	3,31459312	9,949286858	7,42E-19	6,178169698
BSU12770	XkdX	1,897585426	3,725890887	5,59E-10	3,947237826	15,42541957	2,34E-20	9,575655227
BSU12780	XepA	2,108982433	4,313869199	3,13E-11	3,569323617	11,87062192	1,20E-19	8,092245559
BSU12790	XhlA	2,526810186	5,76296072	4,45E-17	3,303240265	9,871301181	3,46E-19	7,817130951
BSU12800	XhlB	2,721399389	6,595122189	2,64E-17	2,984667117	7,91542666	4,92E-18	7,255274424
BSU12810	XlyA	2,868137139	7,301217912	2,67E-17	2,371330874	5,174182263	6,44E-15	6,237700088
BSU12820	SpoIISB	-0,82508931	0,564447259	0,000640499	-0,76314665	0,589209809	0,000605365	0,576828534
BSU12830	SpoIISA	-1,11605192	0,461354643	2,87E-05	-0,77023211	0,586323138	0,003330065	0,523838891
BSU12840	Pit	-1,37090696	0,386648103	1,55E-07	-0,5465562	0,684652487	0,005456953	0,535650295
BSU12850	YkaA	-1,53853701	0,344234355	1,16E-08	-0,13218956	0,912445589	0,641543023	0,628339972
BSU12860	SteT	-1,3683797	0,387326012	0,000411566	0,006827611	1,004743756	0,810767676	0,696034884
BSU12870	MhqA	-0,88163078	0,542753574	0,000461007	-0,94773325	0,518446401	3,02E-05	0,530599988
BSU12880	YkcB	-0,46823096	0,722850416	0,176106274	0,396315811	1,316142602	0,084489705	1,019496509
BSU12890	YkcC	-1,2293176	0,426519142	0,265340115	0,716634462	1,643343946	0,453391321	1,034931544
BSU12900	HtrA	-0,22571491	0,855171151	0,077806349	-0,45279857	0,730624195	0,085755452	0,792897673
BSU12910	ProG	0,481309915	1,396010618	0,092928308	0,013722685	1,009557221	0,33565163	1,20278392
BSU12920	DppA	0,280429212	1,21455617	0,004605097	1,259923996	2,394831241	1,39E-10	1,804693705
BSU12930	DppB	0,413315248	1,331742585	0,091698355	1,351665734	2,552066166	2,07E-11	1,941904376
BSU12940	DppC	0,333436308	1,260010982	0,030357563	1,5104061	2,848902208	1,82E-12	2,054456595
BSU12950	DppD	0,316050935	1,244918187	0,010359325	1,043127121	2,060689474	2,21E-09	1,652803831
BSU12960	DppE	0,523665959	1,437603632	0,029019682	0,581047139	1,495934636	1,98E-05	1,466769134
BSU12970	YkfA	-0,25597013	0,837423825	0,164246434	0,380752388	1,302020703	0,002495718	1,069722264

BSU12980	YkfB	-0,22621352	0,854875648	0,099347374	0,431572248	1,348702591	0,000167132	1,101789119
BSU12990	YkfC	0,491484735	1,40589099	0,068820242	0,687411006	1,61039099	1,19E-05	1,50814099
BSU13000	YkfD	1,171274252	2,25210525	7,65E-07	0,748464537	1,680003847	3,95E-05	1,966054549
BSU13010	YkgB	0,89318322	1,857269561	1,09E-05	-0,56894827	0,674108038	0,01595133	1,265688799
BSU13020	YkgA	0,37520161	1,297020794	0,104917689	-0,20817891	0,86562921	0,279059352	1,081325002
BSU13030	YkhA	-0,71458033	0,609382373	0,006685691	-0,11188068	0,925380959	0,469920139	0,767381666
BSU13040	Hmp	-0,52934227	0,692870544	0,02892477	-1,34187045	0,394508846	1,57E-08	0,543689695
BSU13050	YkzH	-1,43320316	0,3703078	0,131524281	-0,8449401	0,556733923	0,199221398	0,463520862
**BSU13060**	**YkjA**	**-3,43818907**	**0,092257559**	**9,31E-18**	**-1,66427085**	**0,315503771**	**2,77E-11**	**0,203880665**
BSU13070	YkkA	-0,24407801	0,844355232	0,116813772	0,13931897	1,10138508	0,450958065	0,972870156
BSU13080	YkkB	-0,07537129	0,949097831	0,754752976	0,545191671	1,459214197	0,033812073	1,204156014
BSU13090	YkkC	-0,74033639	0,598599762	0,034019009	0,038229704	1,026853027	0,096779869	0,812726394
BSU13100	YkkD	-0,91975959	0,528597098	0,000327003	0,05225718	1,036885924	0,20370319	0,782741511
BSU13110	YkkE	0,123718814	1,089539737	0,025373009	-0,00716216	0,995047872	0,85061906	1,042293804
BSU13120	ProB	-0,0303337	0,979193779	0,112152798	0,354363498	1,278421431	0,101363798	1,128807605
BSU13130	ProA	0,328472018	1,255682756	0,073402422	0,08527962	1,060893341	0,329220296	1,158288048
BSU13140	OhrA	1,070520315	2,100190675	2,97E-06	-0,00522402	0,996385535	0,012299559	1,548288105
BSU13150	OhrR	-0,0570213	0,961246739	0,320350164	-0,18085475	0,882180177	0,423184339	0,921713458
BSU13160	OhrB	1,125063943	2,181112134	2,74E-07	-0,27670456	0,825474437	0,29819309	1,503293286
BSU13169	YkzN	0,525757868	1,43968967	0,003803145	0,054378128	1,038411401	0,73104267	1,239050535
BSU13170	GuaD	-0,54990221	0,683066428	0,045967669	-1,14460896	0,452312271	9,24E-07	0,56768935
BSU13180	MetE	-1,55625721	0,340032085	7,36E-10	0,292290823	1,224583223	0,067770396	0,782307654
BSU13190	IspA	-0,02434027	0,983270135	0,02864204	-0,08137315	0,945157622	0,734769786	0,964213878
BSU13200	RsbRB	0,119589485	1,086425679	0,535237519	0,326170327	1,253681024	0,060910795	1,170053351
BSU13210	ThiX	2,547199551	5,844985927	1,22E-15	0,144032753	1,104989571	0,019885371	3,474987749
BSU13220	ThiW	1,931472895	3,814444297	5,42E-14	0,713002702	1,639212285	0,002762568	2,726828291
BSU13230	ThiV	2,148875774	4,434820688	1,25E-13	0,321081182	1,249266422	0,006649487	2,842043555
**BSU13240**	**ThiU**	**1,194583503**	**2,288787467**	**3,63E-08**	**1,453268701**	**2,73827758**	**4,93E-06**	**2,513532524**
BSU13250	YkoG	-1,63133093	0,322790286	5,54E-08	-0,0057444	0,996026202	0,308287598	0,659408244

BSU13260	YkoH	-1,25532968	0,41889783	3,98E-06	0,321807902	1,249895866	0,07176263	0,834396848
BSU13270	YkoI	-1,38714555	0,382320496	1,21E-06	-0,22443233	0,855931753	0,177698097	0,619126125
BSU13280	YkoJ	3,547365809	11,69131909	3,47E-20	-0,32001567	0,801061175	0,184664015	6,246190135
**BSU13290**	**YkzD**	**-2,72633754**	**0,151109101**	**4,86E-16**	**-2,2254574**	**0,213830952**	**2,34E-14**	**0,182470026**
BSU13300	MgtE	0,181370563	1,133960638	0,019644575	0,081087817	1,057815351	0,506295637	1,095887995
BSU13310	TnrA	0,885050988	1,846829891	2,24E-05	-0,12597574	0,916384057	0,626264354	1,381606974
BSU13320	YkzB	-0,76528023	0,588339077	0,038896065	-0,53250858	0,691351555	0,034451458	0,639845316
BSU13330	YkoL	-0,94059862	0,52101665	0,447362341	0,080935539	1,057703703	0,768812109	0,789360177
BSU13340	YkoM	1,16549728	2,243105195	1,12E-08	0,552013349	1,466130323	0,00314865	1,854617759
BSU13350	YkoN	-2,09329633	0,234344634	5,96E-11	-0,80492428	0,572392122	0,000129763	0,403368378
**BSU13360**	**YkoP**	**-2,47189104**	**0,180254722**	**6,43E-15**	**-1,23241921**	**0,425603168**	**1,21E-07**	**0,302928945**
BSU13370	YkoQ	-0,7998361	0,574414433	0,000368633	-1,01826462	0,493709866	0,000180675	0,534062149
**BSU13380**	**YkoS**	**-3,7270314**	**0,075518222**	**1,84E-19**	**-1,14134825**	**0,453335721**	**1,01E-05**	**0,264426971**
**BSU13390**	**YkoT**	**-2,66913552**	**0,157220852**	**1,72E-16**	**-1,53871595**	**0,344191662**	**6,91E-10**	**0,250706257**
**BSU13400**	**YkoU**	**-3,04066411**	**0,121525914**	**6,37E-18**	**-2,48945938**	**0,178072991**	**1,00E-15**	**0,149799453**
**BSU13410**	**YkoV**	**-3,3206754**	**0,100086868**	**1,92E-18**	**-2,15108386**	**0,225143407**	**3,60E-14**	**0,162615137**
BSU13420	YkoW	-1,30040469	0,406012291	0,008853383	-0,64795319	0,638185093	0,001902472	0,522098692
BSU13430	YkoX	0,736475573	1,666100668	5,29E-05	-0,50727398	0,703550566	0,054972859	1,184825617
BSU13440	YkoY	-0,14235597	0,906038355	0,25803283	-0,77154751	0,585788791	0,000528131	0,745913573
BSU13450	SigI	0,000773957	1,00053661	0,052442845	-0,09053701	0,939173101	0,592709279	0,969854855
BSU13460	RsgI	-0,36660179	0,775607259	0,105906811	-0,13397325	0,911318176	0,318375717	0,843462718
**BSU13470**	**SspD**	**-2,6751626**	**0,156565408**	**5,14E-17**	**-2,66758149**	**0,157390298**	**2,85E-16**	**0,156977853**
BSU13480	YkrK	-0,70582513	0,613091738	0,00277487	-0,73496218	0,600833776	0,001420955	0,606962757
BSU13490	YkrL	0,993645694	1,991210432	2,44E-06	-0,50682187	0,703771077	0,065629065	1,347490755
BSU13500	KtrD	-1,20861982	0,43268235	2,90E-05	-0,80949935	0,570579829	0,000470123	0,501631089
**BSU13509**	**YkzP**	**-2,22150604**	**0,21441741**	**6,67E-15**	**-3,16757957**	**0,111291895**	**2,54E-18**	**0,162854652**
**BSU13510**	**YkzE**	**-1,88736965**	**0,270299426**	**4,16E-11**	**-2,7805758**	**0,145533602**	**2,00E-16**	**0,207916514**
BSU13520	YkrP	0,40857845	1,327377247	0,001205639	0,115852196	1,083614945	0,23479787	1,205496096
BSU13530	KinE	0,647113154	1,566031415	0,001068522	0,837523501	1,786980016	6,47E-05	1,676505716

BSU13540	Ogt	0,618790126	1,535586866	0,000393612	0,456109007	1,371836938	0,008215478	1,453711902
BSU13550	MtnA	-1,83862594	0,279587944	1,28E-11	-0,09820771	0,934192839	0,395697715	0,606890391
BSU13560	MtnK	-2,18364448	0,220118991	1,32E-13	0,222445444	1,166709545	0,132704436	0,693414268
BSU13570	MtnU	-0,2840945	0,821256903	0,044664477	0,097213516	1,069705391	0,299348487	0,945481147
BSU13580	MtnE	-0,23305618	0,850830597	0,289091089	0,338686099	1,26460436	0,063272444	1,057717478
BSU13590	MtnW	0,274725711	1,209764064	0,00327524	1,184910605	2,273493068	3,38E-07	1,741628566
BSU13600	MtnX	-0,11124061	0,925791608	0,12987041	1,317705273	2,492693107	8,66E-09	1,709242357
BSU13610	MtnB	0,123428938	1,089320841	0,247765396	0,970821977	1,959956965	6,14E-06	1,524638903
BSU13620	MtnD	0,473969548	1,388925819	0,016524463	0,920597936	1,892899657	3,34E-05	1,640912738
BSU13630	YkvA	1,668468867	3,178770512	2,04E-11	-0,02668982	0,981670101	0,322465883	2,080220307
BSU13640	Spo0E	1,759983207	3,386941825	3,20E-12	0,490961106	1,405380812	0,027999271	2,396161318
BSU13650	Eag	1,024696853	2,034531832	9,23E-09	0,526480512	1,44041099	0,045260186	1,737471411
BSU13660	KinD	0,852530775	1,805665647	8,34E-07	0,219921216	1,164669984	0,211294015	1,485167815
BSU13670	MhqR	-0,19841339	0,871508483	0,009047202	-0,63652647	0,643259842	0,001312946	0,757384162
BSU13680	MotB	0,409625542	1,328340993	0,01779215	-0,8894035	0,539837273	0,000273347	0,934089133
BSU13690	MotA	0,03182264	1,022302844	0,248235078	-0,65010729	0,637232923	0,010722576	0,829767884
BSU13700	ClpE	0,385449487	1,306266705	0,420323443	0,932462001	1,908530181	0,013205102	1,607398443
BSU13710	YkvI	1,467695228	2,765796916	3,46E-09	0,532378883	1,446312078	0,000392848	2,106054497
BSU13720	QueC	-0,29427738	0,815480688	0,000132942	0,630900379	1,548531123	0,019626491	1,182005905
BSU13730	QueD	-0,70041592	0,615394769	0,071501987	1,598899974	3,029122608	1,10E-05	1,822258688
BSU13740	QueE	-0,8948822	0,537791105	0,01955897	1,877992521	3,675632478	4,79E-06	2,106711792
BSU13750	QueF	-1,02618511	0,491006794	0,001493548	-0,00484538	0,99664707	0,253089768	0,743826932
BSU13760	YkvN	1,128296019	2,186003967	1,17E-07	0,029432981	1,020610918	0,314579932	1,603307443
BSU13770	YkvO	-0,10067201	0,932598486	0,022698991	0,178804194	1,131945261	0,518897923	1,032271874
BSU13780	YkvP	0,02370137	1,016564228	0,173328763	0,548476211	1,462540131	0,03143774	1,239552179
BSU13789	YkzQ	-0,68882009	0,620361005	0,008532922	0,10445588	1,075088839	0,791305904	0,847724922
BSU13790	YkvQ	0,144291157	1,105187505	0,120853103	1,401314043	2,641420598	0,012969894	1,873304052
BSU13799	YkzR	-0,0235304	0,983822258	0,58414862	0,991676724	1,98849471	0,025878282	1,486158484
BSU13800	YkvR	0,77909292	1,716051583	0,004813223	0,141524107	1,103069817	0,586220019	1,4095607

BSU13810	YkvS	-0,29741675	0,813708095	0,073951869	-1,30356286	0,405124473	5,29E-07	0,609416284
BSU13819	YkzS	0,236160024	1,17785343	0,285647268	-2,09993227	0,233269198	3,93E-11	0,705561314
BSU13820	YkvT	-1,13316926	0,455913091	0,038209507	-0,24995153	0,840924669	0,438511175	0,64841888
BSU13830	YkvU	1,244243757	2,368943445	1,15E-07	0,914490235	1,884902938	3,25E-05	2,126923192
BSU13840	StoA	0,961384945	1,947178231	1,54E-05	-0,10410138	0,930384274	0,72622439	1,438781253
BSU13850	ZosA	-0,69164014	0,619149564	0,011721474	-0,33067585	0,79516389	0,09530166	0,707156727
BSU13860	YkvY	0,659444055	1,579473854	0,003678716	0,449689451	1,36574624	0,009643448	1,472610047
BSU13870	YkvZ	-0,67942181	0,624415471	0,024576165	0,507325331	1,421412539	0,011980539	1,022914005
BSU13880	GlcT	0,268049226	1,204178468	0,141049647	-0,23016022	0,852540205	0,032427416	1,028359337
**BSU13890**	**PtsG**	**-3,42033133**	**0,093406624**	**1,78E-19**	**-1,09904355**	**0,46682588**	**5,97E-06**	**0,280116252**
BSU13900	PtsH	-1,08902706	0,470078284	0,000148884	-0,08005439	0,94602198	0,69481555	0,708050132
BSU13910	PtsI	-0,42478816	0,744948107	0,109790519	-0,10891467	0,927285392	0,798373093	0,836116749
**BSU13920**	**SplA**	**-1,99663046**	**0,250584579**	**1,47E-13**	**-1,66963669**	**0,314332491**	**3,13E-11**	**0,282458535**
**BSU13930**	**SplB**	**-1,84502864**	**0,278349879**	**4,34E-11**	**-1,84890196**	**0,277603573**	**8,52E-12**	**0,277976726**
BSU13940	YkwB	-0,99854586	0,500504222	3,88E-06	-0,62218977	0,649684067	0,022323305	0,575094145
BSU13950	McpC	0,1660663	1,121995041	0,317047582	-0,67141789	0,62788929	0,00098877	0,874942166
BSU13960	YkwC	-0,81374847	0,568901792	0,002567004	-0,5760608	0,670792842	0,017675822	0,619847317
BSU13970	YkwD	-0,24089247	0,846221667	0,100803782	-0,82170417	0,565773235	0,000326959	0,705997451
BSU13980	PbpH	-0,40357913	0,755980468	0,050775867	-0,5631919	0,676803108	0,007364736	0,716391788
BSU13990	KinA	0,395881796	1,315746718	0,012847907	0,434995078	1,351906221	0,016952296	1,333826469
BSU14000	PatA	0,473706537	1,388672633	0,007062913	0,003520219	1,002443009	0,289979824	1,195557821
BSU14009	YkzT	-0,52730653	0,693848923	0,053139973	-0,65650644	0,634412702	0,001401842	0,664130812
BSU14010	CheV	0,418937327	1,336942415	0,046546887	-0,00658883	0,995443383	0,208897592	1,166192899
BSU14020	YkyB	0,392506158	1,312671714	0,06141574	-0,93093509	0,524518263	0,000318965	0,918594989
BSU14030	YkuC	-0,03658402	0,974960705	0,042877436	-0,40365956	0,755938328	0,171222672	0,865449517
BSU14040	Ldt	-1,64375693	0,320022016	1,12E-09	-1,04557158	0,484452934	4,00E-07	0,402237475
BSU14050	YkuE	0,135395112	1,098393591	0,254306123	0,407295439	1,326197315	0,042809561	1,212295453
BSU14060	FadH	0,772542024	1,708277112	7,22E-06	0,609845766	1,52609605	0,002122985	1,617186581
BSU14071	FadG	0,787186295	1,725705515	0,000264309	0,955362614	1,939066947	3,79E-06	1,832386231

BSU14072	YkzU	1,791153309	3,46091452	5,25E-09	0,504511998	1,418643409	0,008003191	2,439778964
BSU14080	YkuH	1,307818497	2,475669102	3,75E-08	1,28046516	2,429172866	7,31E-08	2,452420984
BSU14090	YkuI	-0,07946702	0,946407219	0,198710754	0,742601587	1,673190355	0,006084781	1,309798787
BSU14100	YkuJ	-1,54868634	0,341821172	2,76E-10	-0,26846239	0,830203899	0,403751911	0,586012535
BSU14110	YkuK	-1,36499734	0,388235153	2,13E-08	-0,26444063	0,832521461	0,509373107	0,610378307
BSU14120	AbbA	-0,13159399	0,912822344	0,250917622	0,223018605	1,167173154	0,030339205	1,039997749
BSU14130	YkuL	0,381821228	1,30298568	0,044813944	0,742684915	1,673286999	0,000131873	1,488136339
BSU14140	CcpC	1,199053598	2,295890121	1,70E-06	0,374394813	1,296295665	0,053506312	1,796092893
BSU14150	YkuN	0,335353006	1,261686087	0,004241732	0,259420693	1,19699796	0,014921718	1,229342023
BSU14160	YkuO	-0,18406203	0,880221165	0,054232596	0,181248217	1,133864478	0,035753888	1,007042822
BSU14170	YkuP	-0,46531777	0,724311522	0,025275339	0,075325308	1,053598581	0,153148278	0,888955052
BSU14180	YkuQ	-0,37816704	0,769414522	0,061499101	0,228134517	1,17131939	0,193720188	0,970366956
BSU14190	YkuR	-0,69278005	0,618660552	0,008165761	0,63014731	1,54772302	0,001034439	1,083191786
BSU14200	YkuS	-1,24258537	0,422614635	1,38E-07	-0,86445457	0,54925402	0,001178316	0,485934327
BSU14210	YkuT	0,63306014	1,550851059	0,00036394	-0,48669579	0,713657719	0,045896448	1,132254389
BSU14220	YkuU	-0,37864364	0,769160382	0,0861209	-0,38536369	0,765585976	0,134956749	0,767373179
BSU14230	YkuV	0,108016923	1,077745787	0,297215932	-0,48977055	0,712138348	0,071097787	0,894942067
BSU14240	Rok	1,045533574	2,064129626	3,83E-07	0,219405244	1,16425352	0,084289006	1,614191573
BSU14250	YknT	-0,69024927	0,619746761	0,00066523	-2,09586028	0,233928529	5,65E-12	0,426837645
BSU14260	MobA	0,278619908	1,213033933	0,083683097	0,634862534	1,552789787	0,006202539	1,38291186
BSU14270	MoeB	0,148125988	1,108129115	0,056086868	1,013408139	2,018674271	6,93E-06	1,563401693
BSU14280	MoeA	0,611324652	1,527661231	0,002555069	0,686189801	1,609028412	0,000239358	1,568344821
BSU14290	MobB	0,37760058	1,299179326	0,015505199	0,695701383	1,619671665	0,000385409	1,459425495
BSU14300	MoaE	0,403347528	1,322573159	0,01843604	0,701239447	1,625901035	0,000811232	1,474237097
BSU14310	MoaD	0,539823385	1,453794533	0,001312426	0,542225458	1,456217102	0,005484181	1,455005818
BSU14320	YknU	-2,60383307	0,164500849	3,43E-16	-0,51266633	0,700925819	0,035064853	0,432713334
BSU14330	YknV	-2,12863606	0,228673952	2,59E-13	-0,45103421	0,731518266	0,136638942	0,480096109
BSU14340	YknW	-1,62430552	0,324365993	6,93E-10	-0,30681607	0,808423926	0,480271613	0,56639496
BSU14350	YknX	-1,3667114	0,387774167	8,33E-09	-0,63123254	0,645624601	0,027100384	0,516699384

BSU14360	YknY	-1,70445277	0,30683761	9,56E-09	-0,3488329	0,785219064	0,180984027	0,546028337
BSU14370	YknZ	-1,08399592	0,47172046	1,91E-05	-0,35296215	0,782974838	0,25953725	0,627347649
BSU14380	FruR	-0,98021297	0,506904905	1,44E-06	-0,22916534	0,853128317	0,263564283	0,680016611
BSU14390	FruK	-0,85494671	0,55288575	8,78E-05	-0,34216716	0,788855434	0,087311473	0,670870592
BSU14400	FruA	-0,5310065	0,692071741	0,001807577	-0,67449503	0,626551487	0,002100623	0,659311614
BSU14410	SipT	0,666121256	1,586801049	0,001889392	0,161706575	1,118609568	0,487869809	1,352705308
BSU14420	YkoA	0,895514452	1,860273124	5,90E-06	-1,02211137	0,492395211	0,000148299	1,176334167
BSU14430	YkpA	0,112041051	1,080756154	0,426925605	-0,26435765	0,832569347	0,43743989	0,95666275
BSU14440	YkpB	0,648089937	1,567092062	0,000610381	-0,18439223	0,880019725	0,465346147	1,223555894
BSU14450	AmpS	-0,17418257	0,886269535	0,029942914	-0,35672686	0,780934329	0,078466713	0,833601932
BSU14460	YkpC	-0,48235206	0,715809671	0,041204491	-1,64265858	0,320265749	1,98E-10	0,51803771
BSU14470	MreBH	1,044597405	2,062790641	0,004023614	0,626942885	1,544289128	0,000887681	1,803539884
BSU14480	Abh	1,688445988	3,223093382	1,99E-10	0,031522522	1,022090201	0,627226999	2,122591791
BSU14490	KinC	-0,55863022	0,678946488	0,068653144	0,134884212	1,098004686	0,457306423	0,888475587
BSU14500	YkqA	-0,3073006	0,808152463	0,000594595	0,431090163	1,34825199	0,017186794	1,078202227
BSU14510	KtrC	-0,56636257	0,675317304	0,030459271	-0,31998481	0,801078311	0,164714517	0,738197808
BSU14520	AdeC	0,654289919	1,573841132	0,000362385	0,53790453	1,4518622	0,021666843	1,512851666
BSU14530	RnjA	-0,54643551	0,684709765	0,019730488	-0,15072358	0,900798558	0,686654229	0,792754161
BSU14540	YkzG	-0,32395808	0,798875129	0,008769372	-0,04272762	0,970817746	0,756124234	0,884846437
BSU14550	YkrA	-0,2599557	0,835113564	0,10885461	-0,29181468	0,816873914	0,23722603	0,825993739
BSU14560	YkrB	0,098233803	1,070462163	0,061079246	-0,88686343	0,540788574	5,44E-05	0,805625368
BSU14569	YkzV	0,197243289	1,146505511	0,132319099	-1,81363264	0,284473734	2,92E-12	0,715489622
BSU14570	YkyA	0,257695499	1,195567429	0,165853568	0,332323369	1,259039346	0,119442903	1,227303387
BSU14580	PdhA	-0,73018522	0,602826517	9,30E-05	-0,98426887	0,505481825	7,03E-05	0,554154171
BSU14590	PdhB	-0,78176191	0,581656005	0,003065504	-0,81537987	0,56825884	0,000790841	0,574957422
BSU14600	PdhC	-0,67594797	0,625920805	0,001877959	-0,4347273	0,739833587	0,170078341	0,682877196
BSU14610	PdhD	0,617669208	1,534394239	0,00345035	-0,11404926	0,923991025	0,290419258	1,229192632
BSU14620	Slp	0,821437303	1,767165676	1,99E-05	0,206689296	1,154036853	0,257459386	1,460601265
BSU14629	Sr1	1,331734166	2,517050507	1,65E-08	-0,93175733	0,524219409	0,000118238	1,520634958

BSU14630	SpeA	-0,96444644	0,512475008	0,000236279	-0,64891816	0,637758374	0,002255154	0,575116691
BSU14640	YktA	0,721943194	1,649402151	0,00011198	-0,37337902	0,771972297	0,081874862	1,210687224
BSU14650	YktB	-0,25978315	0,835213452	0,060974091	-0,62705913	0,647494963	0,009146224	0,741354208
BSU14660	YkzI	1,117578041	2,169824021	5,79E-06	-0,12883467	0,91456989	0,152505133	1,542196956
BSU14670	YktC	-0,0096658	0,99332257	0,123983394	0,088690281	1,063404356	0,344646769	1,028363463
BSU14680	YkzC	1,728411259	3,313627104	1,72E-08	-0,07035741	0,952402026	0,708610421	2,133014565
BSU14690	YktD	-0,82101264	0,566044493	0,003485742	-0,30417727	0,809903952	0,0814511	0,687974223
BSU14700	NprE	0,364895929	1,28778873	0,103132606	0,434525405	1,351466176	0,03917056	1,319627453
BSU14710	YlaA	0,890158478	1,853379704	7,57E-05	-0,07066913	0,952196263	0,2568924	1,402787983
BSU14720	YlaB	0,762416459	1,696329533	4,54E-07	-0,60135649	0,659133915	0,053035428	1,177731724
BSU14730	YlaC	-0,44996013	0,732063081	0,000146199	-0,43941375	0,737434207	0,170999179	0,734748644
BSU14740	YlaD	-1,05848818	0,480134938	0,037800189	-0,62899532	0,646626562	0,161086786	0,56338075
BSU14750	YlaE	0,996008136	1,994473751	0,000475431	0,785849268	1,724106947	0,010067357	1,859290349
BSU14760	YlaF	1,754235915	3,373476039	4,01E-12	-0,82941559	0,562757158	0,000381946	1,968116598
BSU14770	YlaG	-0,54294699	0,686367434	0,020650832	0,324512515	1,252241236	0,042017262	0,969304335
BSU14780	YlaH	1,448053188	2,728396255	3,37E-08	-0,06500958	0,95593897	0,585924051	1,842167613
BSU14790	YlaI	0,058051883	1,041059036	0,436899455	-0,88114979	0,542934555	0,000217026	0,791996795
**BSU14800**	**YlaJ**	**-2,74069011**	**0,149613254**	**1,59E-16**	**-1,98626378**	**0,252391674**	**2,07E-12**	**0,201002464**
BSU14810	YlaK	0,231789238	1,174290408	0,011270216	0,098288657	1,070502865	0,173266347	1,122396636
BSU14820	YlaL	0,784186744	1,722121276	1,12E-05	-0,64593218	0,639079725	0,002799556	1,1806005
BSU14830	YlaM	-0,3199393	0,801103583	0,062030189	0,228184366	1,171359863	0,082797821	0,986231723
BSU14840	YlaN	0,757032938	1,690011353	0,000210211	-0,30083737	0,811781085	0,219748692	1,250896219
BSU14850	FtsW	0,296127929	1,227844557	0,004472334	0,402444717	1,321745777	0,022025062	1,274795167
**BSU14860**	**PycA**	**1,753652666**	**3,372112494**	**1,69E-11**	**1,177105372**	**2,261226284**	**9,17E-08**	**2,816669389**
BSU14870	CtaA	0,067290937	1,047747397	0,089688136	-0,70374521	0,613976266	0,001864764	0,830861831
BSU14880	CtaB	0,390010071	1,310402552	0,010552396	-0,0236515	0,983739683	0,358038278	1,147071117
BSU14890	CtaC	0,48318222	1,397823514	0,089494546	0,506759056	1,420854727	0,000317095	1,40933912
BSU14900	CtaD	0,507357193	1,421443931	0,031122974	0,547387964	1,461437329	3,18E-05	1,44144063
BSU14910	CtaE	-0,10012159	0,932954361	0,151958877	0,415842891	1,334077881	0,000423564	1,133516121

BSU14920	CtaF	0,247540991	1,187181891	0,239771307	0,329159045	1,256280868	0,001733417	1,221731379
BSU14930	CtaG	0,294046611	1,226074471	0,064884817	-0,04933501	0,966381666	0,341251341	1,096228069
BSU14940	YlbA	1,130092862	2,18872828	7,01E-08	-0,00252755	0,998249568	0,70925966	1,593488924
BSU14950	YlbB	-0,88429814	0,541751015	0,000879637	-0,11975705	0,920342626	0,558906184	0,731046821
BSU14960	YlbC	-0,14454229	0,904666347	0,449793067	-0,10600719	0,929156045	0,814359551	0,916911196
**BSU14970**	**YlbD**	**-1,91381297**	**0,265390205**	**2,29E-09**	**-1,77967498**	**0,291249005**	**4,65E-09**	**0,278319605**
**BSU14980**	**YlbE**	**-1,83687199**	**0,27992806**	**0,015104583**	**-1,84751538**	**0,277870508**	**0,002013212**	**0,278899284**
BSU14990	YlbF	1,05280051	2,074552992	1,07E-06	-0,34093089	0,789531706	0,237866671	1,432042349
BSU15000	YlbG	1,324828151	2,505030468	7,61E-08	0,170610464	1,125534644	0,246001555	1,815282556
BSU15010	YlbH	0,300563492	1,231625372	0,033105352	0,221180219	1,165686805	0,261668609	1,198656088
BSU15020	YlbI	0,668935425	1,589899337	0,009847175	0,093735752	1,067129862	0,088740056	1,328514599
BSU15030	YlbJ	0,80814956	1,750964168	1,42E-05	0,593241085	1,508632159	0,007066051	1,629798163
BSU15040	YlbK	0,217416033	1,162649335	0,002674433	0,355372354	1,279315725	0,079016721	1,22098253
BSU15050	YlbL	-0,2926357	0,816409175	0,04510557	-0,37541548	0,770883373	0,161197962	0,793646274
BSU15060	YlbM	-0,03509372	0,975968356	0,371983264	0,131916376	1,095748251	0,605654385	1,035858303
BSU15069	YlzH	-1,10209266	0,465840294	2,39E-05	-0,27443536	0,826773834	0,095075346	0,646307064
BSU15070	YlbN	-0,4301394	0,742190068	0,001814203	-0,24625797	0,843080344	0,434386702	0,792635206
BSU15080	RpmF	0,166673682	1,122467507	0,213870802	-0,51035078	0,702051719	0,032944831	0,912259613
BSU15090	GerR	0,387888392	1,308476843	0,027113109	-0,5916977	0,663561598	0,046480757	0,986019221
BSU15100	YlbP	0,622102001	1,539116034	0,000365793	0,063308787	1,044859376	0,53024348	1,291987705
BSU15110	YlbQ	-0,46664043	0,72364778	0,015109947	0,069665039	1,049472991	0,57193447	0,886560385
BSU15120	BshC	-1,17963165	0,441464198	1,60E-06	-0,19366853	0,874379494	0,583184926	0,657921846
BSU15130	MraZ	0,588455075	1,503635701	0,013947351	0,110621108	1,079692965	0,126181868	1,291664333
BSU15140	MraW	0,47440205	1,389342264	0,002116204	-0,16625614	0,891152267	0,496246028	1,140247266
BSU15150	FtsL	0,722475718	1,650011087	0,000871131	-0,20687537	0,866411702	0,643355731	1,258211394
BSU15160	PbpB	1,068271687	2,096919803	5,40E-07	0,411654812	1,330210729	0,031897105	1,713565266
BSU15170	SpoVD	0,763247425	1,69730687	0,000407541	0,250506303	1,189624531	0,144810829	1,443465701
BSU15180	MurE	-0,65218685	0,636315052	0,00693965	0,704417989	1,629487165	0,000318901	1,132901109
BSU15190	MraY	-1,05224286	0,482217909	1,31E-05	0,287543009	1,220559825	0,04213414	0,851388867

BSU15200	MurD	-0,8339564	0,560988694	0,0001691	0,433368888	1,350383226	0,013747424	0,95568596
BSU15210	SpoVE	0,394521169	1,314506402	0,045047352	0,330934174	1,257827579	0,015736823	1,286166991
BSU15220	MurG	-0,21652991	0,860633013	0,295272707	0,699365185	1,623790135	0,001561258	1,242211574
BSU15230	MurB	0,629141591	1,546644461	0,000741555	0,699396205	1,62382505	0,000474197	1,585234755
BSU15240	DivIB	0,18562215	1,137307322	0,21092494	0,717987615	1,644886017	0,000254433	1,391096669
BSU15250	YlxW	1,251595623	2,381046215	4,25E-07	0,551966523	1,466082737	0,002470953	1,923564476
BSU15260	YlxX	-0,01197122	0,991736514	0,008656604	0,479796202	1,394546656	0,003862848	1,193141585
BSU15270	Sbp	1,678646178	3,201274031	4,86E-09	0,016461959	1,011475909	0,137379191	2,10637497
BSU15280	FtsA	1,238866033	2,360129517	1,18E-07	0,684857679	1,60754339	0,000124566	1,983836454
BSU15290	FtsZ	1,024081941	2,033664849	2,00E-05	0,238180171	1,179503885	0,012543203	1,606584367
BSU15300	Bpr	0,352366997	1,276653487	0,009303195	1,376446409	2,59628077	3,18E-09	1,936467128
BSU15310	SpoIIGA	1,171673587	2,252728715	1,79E-05	-0,0284928	0,980444046	0,079272355	1,61658638
BSU15320	SigE	0,679338573	1,601405396	0,000291624	0,509433332	1,423490961	0,001917993	1,512448179
BSU15330	SigG	0,217676908	1,16285959	0,130106428	-0,38068428	0,768073205	0,153580844	0,965466397
BSU15340	YlmA	0,202426013	1,150631613	0,299350377	0,328165677	1,255416153	0,0364845	1,203023883
BSU15350	YlmB	0,788460557	1,72723042	0,000337214	0,190330738	1,141025266	0,012462525	1,434127843
BSU15360	YlmC	0,014204114	1,009894168	0,120785342	-0,10279315	0,931228328	0,614165832	0,970561248
BSU15370	YlmD	0,257646103	1,195526495	0,161645708	0,370983051	1,293233739	0,040374433	1,244380117
BSU15380	YlmE	-0,11809523	0,921403366	0,081869614	0,26878505	1,204792797	0,056910178	1,063098082
BSU15390	SepF	0,009376304	1,006520324	0,272147607	0,083855676	1,059846754	0,335297551	1,033183539
BSU15400	YlmG	0,056358147	1,039837541	0,014106573	0,254321304	1,192774487	0,121579712	1,116306014
BSU15410	YlmH	0,743964378	1,674771623	9,80E-06	0,76229998	1,696192583	0,000872823	1,685482103
BSU15420	DivIVA	0,768602058	1,703618212	0,000163231	0,450526787	1,366539145	0,012145248	1,535078678
BSU15430	IleS	-0,45975976	0,72710733	0,111554134	1,11476227	2,165593211	1,30E-06	1,446350271
**BSU15440**	**YlyA**	**-2,99635745**	**0,125316002**	**1,96E-17**	**-2,80758582**	**0,142834281**	**8,37E-17**	**0,134075142**
BSU15450	LspA	-1,04322103	0,485242886	1,77E-06	-0,11089383	0,926014167	0,376561236	0,705628527
BSU15460	YlyB	-1,04323752	0,485237339	1,59E-05	-0,39073909	0,762738756	0,177095627	0,623988048
BSU15470	PyrR	0,759499505	1,692903227	0,00023883	0,747079768	1,678392072	0,00110973	1,685647649
BSU15480	PyrP	-0,3182942	0,802017601	0,000148901	-0,72020361	0,607011767	0,000522124	0,704514684

BSU15490	PyrB	-1,34645049	0,393258408	0,06909893	1,290601439	2,446300171	0,066053948	1,419779289
BSU15500	PyrC	-1,45488202	0,364784915	0,02148997	0,392698313	1,312846563	0,000270141	0,838815739
BSU15510	PyrAA	-1,15382536	0,449431963	0,06469224	0,745623376	1,676698599	0,172353684	1,063065281
BSU15520	PyrAB	-2,0672101	0,238620501	0,001347749	0,767867701	1,702751262	0,013645075	0,970685882
BSU15530	PyrK	-0,25529743	0,837814392	0,249196892	0,506191956	1,420296322	0,051115436	1,129055357
BSU15540	PyrD	-0,94462019	0,519566316	0,010292659	0,675435306	1,597078589	0,021170722	1,058322453
BSU15550	PyrF	-0,09898622	0,933688866	0,223184637	0,683162271	1,605655368	0,016358244	1,269672117
BSU15560	PyrE	0,426851899	1,344296987	0,001212778	0,372745761	1,2948148	0,37136635	1,319555894
BSU15570	CysH	-1,1957682	0,436553933	2,53E-07	0,639811664	1,558125741	0,000335663	0,997339837
BSU15580	CysP	-0,91932104	0,528757806	3,01E-06	0,602493275	1,518338303	0,000439024	1,023548054
BSU15590	Sat	-1,7528218	0,296720849	8,56E-10	1,043901359	2,061795662	4,02E-07	1,179258255
BSU15600	CysC	-1,05705141	0,480613339	8,27E-06	1,125824394	2,182262112	2,83E-07	1,331437725
BSU15610	YlnD	-0,76617635	0,587973751	0,000276746	1,043902197	2,06179686	5,56E-07	1,324885306
BSU15620	YlnE	0,511157659	1,425193352	0,018422898	0,861550796	1,816990399	6,25E-05	1,621091875
BSU15630	YlnF	0,822085594	1,767959951	6,06E-05	0,608399589	1,524567037	0,000948957	1,646263494
BSU15640	YloA	-0,48769712	0,713162564	0,022019397	-0,2756741	0,826064247	0,338826838	0,769613406
BSU15650	YloB	2,065267748	4,185116398	7,68E-12	0,576098571	1,490812242	0,001554435	2,83796432
BSU15660	YloC	0,167055836	1,122764875	0,312212117	0,620374615	1,537274303	0,001266653	1,330019589
BSU15670	RemA	-0,1199657	0,920209532	0,229156543	0,440232563	1,356823031	0,016163472	1,138516281
BSU15680	Gmk	-0,17927236	0,883148312	0,048131764	0,467302518	1,382522077	0,011838291	1,132835194
BSU15690	YloH	0,58920374	1,504416192	0,016052655	0,139952885	1,101869131	0,264382738	1,303142661
BSU15700	YloI	0,240672353	1,181543179	0,142084752	0,589318843	1,504536224	0,002738088	1,343039702
BSU15710	PriA	0,312392689	1,241765443	0,172533946	0,388414526	1,308954116	0,029523699	1,275359779
BSU15720	Def	-0,1293037	0,914272607	0,391087986	0,285985165	1,219242556	0,078610834	1,066757582
BSU15730	Fmt	-0,638904	0,642200635	0,014971213	0,135777109	1,098684462	0,204631097	0,870442549
BSU15740	YloM	-0,67581941	0,625976581	0,003373343	0,222691754	1,166908754	0,129635859	0,896442667
BSU15750	YloN	0,212984212	1,159083267	0,01809401	0,394396318	1,31439265	0,033774693	1,236737959
BSU15760	PrpC	-0,56111544	0,677777926	0,020118221	0,511391433	1,425424309	0,015707701	1,051601117
BSU15770	PrkC	-0,50519688	0,704564222	0,028674959	0,320534334	1,248792981	0,050767871	0,976678602

BSU15780	CpgA	-0,15445933	0,898469026	0,624963143	0,586834259	1,501947369	0,004262765	1,200208198
BSU15790	Rpe	0,231081194	1,173714233	0,215961389	0,094439177	1,067650297	0,116718326	1,120682265
BSU15800	YloS	-0,57577796	0,670924363	0,070550201	-0,07518091	0,949223084	0,352948465	0,810073724
BSU15810	SpoVM	-0,81191758	0,569624231	0,001169521	-1,35767329	0,390211096	5,79E-08	0,479917663
BSU15820	RpmB	1,10880119	2,156663645	3,86E-09	-0,76894214	0,586847626	0,000854883	1,371755636
BSU15830	YloU	-1,01063857	0,496326512	1,62E-05	-0,08420391	0,943304916	0,862486345	0,719815714
BSU15840	YloV	-0,29681495	0,814047591	0,213068551	-0,4693545	0,722287698	0,045493596	0,768167645
BSU15850	SdaAB	-0,50819072	0,703103646	0,017118255	-0,10002115	0,933019314	0,232465759	0,81806148
BSU15860	SdaAA	-0,09691949	0,935027378	0,231457548	0,171080015	1,125901029	0,371279528	1,030464203
BSU15870	RecG	0,796077925	1,73637424	2,79E-05	1,040021884	2,056258845	5,80E-06	1,896316542
BSU15880	FapR	-0,14990053	0,901312601	0,2039167	0,574882276	1,48955591	0,001199305	1,195434256
BSU15890	PlsX	0,2208693	1,165435611	0,058856555	0,481046721	1,395755965	0,001256771	1,280595788
BSU15900	FabD	0,001541808	1,001069271	0,534984473	0,450340618	1,366362815	0,010896411	1,183716043
BSU15910	FabG	0,258571265	1,1962934	0,020615248	0,641886983	1,56036872	0,001387846	1,37833106
BSU15920	AcpA	0,503449445	1,417598955	0,020440735	0,022512883	1,015727131	0,586654094	1,216663043
BSU15930	Rnc	0,634908958	1,552839755	0,001694147	0,230024012	1,17285447	0,11831755	1,362847112
BSU15940	Smc	-0,5007905	0,70671944	0,081314957	0,191309475	1,14179961	0,145298361	0,924259525
BSU15950	FtsY	-0,31956732	0,801310164	0,089748071	0,449876233	1,365923071	0,021267481	1,083616617
BSU15960	YlqB	0,460268494	1,375797838	0,002911816	-0,51970657	0,697513688	0,004705718	1,036655763
BSU15970	YlxM	-0,31342811	0,804727303	0,029062854	0,347056973	1,271963236	0,043212146	1,038345269
BSU15980	Ffh	-0,55692304	0,679750381	0,015374344	0,35977732	1,283227816	0,048246215	0,981489099
BSU15990	RpsP	-0,50351076	0,705388149	0,068117937	-0,07616692	0,948574558	0,823327194	0,826981354
BSU16000	YlqC	-0,38851609	0,763914938	0,089660476	-0,0959505	0,935655601	0,693343447	0,84978527
BSU16010	YlqD	-0,77576467	0,584078963	9,09E-05	-0,06659157	0,954891305	0,817163359	0,769485134
BSU16020	RimM	-0,19540569	0,873327283	0,119807437	0,017268363	1,012041439	0,730165388	0,942684361
BSU16030	TrmD	-0,20705325	0,866304882	0,040978867	-0,10611798	0,929084692	0,553201059	0,897694787
BSU16040	RplS	0,616285368	1,532923146	0,005607406	-0,18081053	0,882207219	0,691409672	1,207565182
BSU16050	RbgA	0,663014181	1,583387296	0,001119651	0,254922681	1,19327179	0,074932719	1,388329543
BSU16060	RnhB	0,046201145	1,032542486	0,437925427	-0,10907768	0,927180625	0,183352049	0,979861556

BSU16070	YlqG	-0,45209149	0,730982369	0,02967983	-0,47299814	0,720465802	0,033724251	0,725724085
BSU16080	YlqH	-0,29834688	0,813183652	0,128182659	-0,23915968	0,847238656	0,35076127	0,830211154
BSU16090	SucC	0,090266216	1,064566606	0,038134491	0,76072481	1,694341648	0,000177181	1,379454127
BSU16100	SucD	0,234332751	1,176362542	0,0016332	0,62482078	1,542019255	0,002949533	1,359190898
BSU16110	DprA	-0,15655484	0,897164946	0,090057189	0,087494822	1,062523553	0,092816491	0,97984425
BSU16120	TopA	-0,10845243	0,927582542	0,107066671	0,156509586	1,114587274	0,200390081	1,021084908
BSU16130	TrmFO	-1,88403867	0,27092423	3,14E-12	-0,22011125	0,858499236	0,244929333	0,564711733
BSU16140	CodV	-1,50161376	0,353158136	2,54E-08	-0,13080356	0,9133226	0,7535373	0,633240368
BSU16150	ClpQ	-1,23938511	0,423553141	1,54E-07	-0,14401288	0,904998386	0,838050515	0,664275764
BSU16160	ClpY	-0,92847552	0,525413246	0,000258686	-0,26098582	0,834517486	0,455259902	0,679965366
BSU16170	CodY	-0,23857627	0,84758134	0,058133642	-0,36694635	0,77542204	0,02914385	0,81150169
BSU16180	FlgB	0,11464942	1,08271191	0,141391348	0,532128153	1,446060741	0,004819007	1,264386326
BSU16190	FlgC	0,320436104	1,248707957	0,076706269	0,311724145	1,241190143	0,080235587	1,24494905
BSU16200	FliE	0,780833228	1,718122888	0,00028155	0,484599497	1,399197384	0,013787524	1,558660136
BSU16210	FliF	0,027592405	1,019309663	0,027561338	0,735967023	1,665513471	0,002541555	1,342411567
BSU16220	FliG	-0,02396771	0,983524086	0,454000044	0,418215278	1,336273461	0,01325015	1,159898774
BSU16230	FliH	0,088435332	1,063216451	0,196044316	0,767924195	1,70281794	0,001194132	1,383017195
BSU16240	FliI	0,19952823	1,148322785	0,004966117	0,850540202	1,803175979	0,000134775	1,475749382
BSU16250	FliJ	-0,15305889	0,8993416	0,461812434	0,689071566	1,612245637	0,000928457	1,255793618
BSU16260	YlxF	-0,21386246	0,862225742	0,17157187	0,503874894	1,418017065	0,021890735	1,140121403
BSU16270	FliK	0,202264742	1,150502998	0,061705153	0,437000897	1,353787121	0,038222347	1,252145059
BSU16280	YlxG	0,412673658	1,331150469	0,001742196	0,403639984	1,322841291	0,052836044	1,32699588
BSU16290	FlgE	-0,10089859	0,932452027	0,002291981	0,594752095	1,510213057	0,002999517	1,221332542
BSU16299	YlzI	0,370827083	1,293093936	0,00723536	0,735266262	1,664704677	0,000502375	1,478899307
BSU16300	FliL	0,062143039	1,044015434	0,198890869	0,82819847	1,775466907	8,31E-05	1,40974117
BSU16310	FliM	0,209589143	1,156358824	0,030818318	0,949245983	1,930863238	2,58E-05	1,543611031
BSU16320	FliY	0,733970632	1,663210339	1,09E-05	0,645712894	1,564512184	0,001104569	1,613861261
BSU16330	CheY	0,653216778	1,572670874	6,15E-05	0,256221263	1,194346348	0,155499053	1,383508611
BSU16340	FliZ	0,247783599	1,187381548	0,061542522	0,613220912	1,52967049	0,003466997	1,358526019

BSU16350	FliP	0,037890113	1,026611348	0,001547364	0,696505941	1,62057517	0,005408799	1,323593259
BSU16360	FliQ	0,588401142	1,503579491	0,000474568	0,605496468	1,52150225	0,000405651	1,512540871
BSU16370	FliR	0,986734186	1,98169397	6,19E-06	0,374617066	1,296495381	0,022974818	1,639094675
BSU16380	FlhB	0,202017145	1,150305564	0,20281673	0,827822989	1,775004878	3,41E-05	1,462655221
BSU16390	FlhA	-0,48034373	0,716806819	0,029565412	1,090980548	2,130187685	8,77E-06	1,423497252
BSU16400	FlhF	-0,40392452	0,755799507	0,170548964	0,791317553	1,730654276	0,000465709	1,243226891
BSU16410	YlxH	0,09731509	1,069780706	0,401271355	0,913190315	1,883205339	5,45E-05	1,476493022
BSU16420	CheB	-0,16371134	0,892725572	0,410518871	1,259901314	2,39479359	2,23E-07	1,643759581
BSU16430	CheA	0,226949743	1,170357871	0,291121757	1,10449895	2,150241869	2,74E-07	1,66029987
BSU16440	CheW	0,353817417	1,277937621	0,075709362	1,215424482	2,322090954	2,62E-07	1,800014288
BSU16450	CheC	0,749498921	1,681208809	4,56E-05	1,156415404	2,229029028	4,07E-07	1,955118918
BSU16460	CheD	0,459382742	1,374953417	0,00284715	1,244559812	2,369462473	6,43E-08	1,872207945
BSU16470	SigD	0,474864198	1,389787392	0,000435418	1,145627303	2,212423089	6,97E-07	1,80110524
BSU16480	SwrB	-0,23048657	0,852347379	0,402381549	-0,0497013	0,966136342	0,414215734	0,90924186
BSU16490	RpsB	-0,54551715	0,685145762	0,014858499	-0,36348739	0,777283397	0,52457457	0,73121458
BSU16500	Tsf	-0,12847951	0,914795064	0,331860196	-0,5368136	0,689291629	0,036987114	0,802043346
BSU16510	PyrH	-0,45707636	0,728460999	0,010776882	-0,01990276	0,986299181	0,455980078	0,85738009
BSU16520	Frr	-0,2123087	0,863154844	0,048255574	0,213038922	1,159127223	0,242791269	1,011141034
BSU16530	UppS	-0,38125232	0,767770843	0,013040734	0,69335669	1,617041483	0,000289238	1,192406163
BSU16540	CdsA	0,208519807	1,15550204	0,007997859	0,980417687	1,973036557	9,16E-06	1,564269299
BSU16550	IspC	0,084749753	1,060503773	0,135038826	0,602035648	1,517856758	0,001680936	1,289180265
BSU16560	RasP	0,342576442	1,268019069	0,015287707	0,464694691	1,380025276	0,011054587	1,324022173
BSU16570	ProS	1,248653266	2,376195052	3,06E-06	0,903766426	1,870944063	3,18E-05	2,123569558
BSU16580	PolC	1,418162666	2,672449463	7,22E-10	0,400875246	1,320308665	0,040297375	1,996379064
BSU16590	YlxS	0,153393064	1,112182132	0,027454893	-0,11109408	0,925885644	0,653227134	1,019033888
BSU16600	NusA	-0,40550762	0,754970604	0,042791033	0,141208148	1,102828264	0,23840251	0,928899434
BSU16610	YlxR	-0,22753027	0,854095756	0,152764745	0,059155429	1,041855667	0,408258597	0,947975712
BSU16620	YlxQ	-0,14752892	0,90279547	0,379971643	-0,05441407	0,962985469	0,643347384	0,932890469
BSU16630	InfB	-0,13842927	0,90850775	0,123636072	0,167498036	1,123109066	0,349479856	1,015808408

BSU16640	YlxP	0,363791923	1,286803641	0,020274942	-0,45629469	0,728855797	0,051950644	1,007829719
BSU16650	RbfA	0,167098988	1,122798458	0,021018179	-0,536427	0,689476365	0,008067595	0,906137411
BSU16660	TruB	-0,26290858	0,833406015	0,016517198	-0,23788537	0,847987339	0,093907664	0,840696677
BSU16670	RibC	0,247659835	1,187279691	0,011001802	0,2770143	1,211684671	0,123572245	1,199482181
BSU16680	RpsO	0,80082029	1,742091367	0,000348921	-0,14058555	0,907150892	0,710167338	1,324621129
BSU16690	PnpA	-0,03109351	0,978678211	0,614990575	-0,13308321	0,911880572	0,68495365	0,945279392
BSU16700	YlxY	0,211837316	1,1581622	0,026194907	0,604362047	1,520306332	0,000481695	1,339234266
BSU16710	MlpA	-0,13475921	0,910821838	0,618176003	-0,36453204	0,776720778	0,075560135	0,843771308
BSU16720	YmxH	-0,02104416	0,985519168	0,190574269	-0,67058787	0,628250636	0,007047922	0,806884902
**BSU16730**	**SpoVFA**	**-3,06379262**	**0,119593209**	**5,38E-18**	**-1,95935908**	**0,257142668**	**8,52E-13**	**0,188367939**
**BSU16740**	**SpoVFB**	**-2,76067405**	**0,147555127**	**1,21E-15**	**-2,0891903**	**0,235012549**	**7,58E-13**	**0,191283838**
BSU16750	Asd	-0,54744928	0,684228792	0,01029923	-0,25903124	0,835648863	0,262032175	0,759938827
BSU16760	DapG	-0,73903106	0,599141612	0,004381938	-0,06894186	0,953336965	0,716356582	0,776239289
BSU16770	DapA	-0,22555095	0,855268347	0,243067776	-0,24475611	0,843958458	0,342071922	0,849613403
BSU16780	RnjB	1,638725307	3,113905811	7,75E-08	0,228687128	1,171768139	0,055163496	2,142836975
**BSU16790**	**TepA**	**-2,88759726**	**0,135128392**	**4,95E-17**	**-1,60994612**	**0,327610587**	**6,41E-10**	**0,23136949**
**BSU16799**	**YlzJ**	**-2,35909205**	**0,194913774**	**1,90E-14**	**-1,51056893**	**0,350972784**	**2,15E-09**	**0,272943279**
BSU16800	SpoIIIE	-0,83828476	0,559308143	0,000293241	0,053091345	1,037485623	0,495705908	0,798396883
BSU16810	YmfC	-0,03115908	0,978633734	0,004954509	0,423635205	1,341303024	0,008630687	1,159968379
**BSU16825**	**YmfD**	**-3,57399267**	**0,083969391**	**5,57E-18**	**-1,87388044**	**0,272838579**	**7,52E-12**	**0,178403985**
BSU16845	YmfF	-1,85384846	0,276653396	3,79E-12	-0,65704047	0,634177912	0,012541571	0,455415654
BSU16860	YmfH	-1,56710117	0,337485829	1,56E-09	-0,5631521	0,676821779	0,003501703	0,507153804
BSU16870	YmfI	-1,72664982	0,302152793	6,49E-11	-0,5572802	0,679582118	0,0234224	0,490867455
**BSU16880**	**YmfJ**	**-2,63308107**	**0,161199472**	**1,24E-16**	**-1,88393212**	**0,270944239**	**1,02E-11**	**0,216071856**
BSU16910	RodZ	0,047309905	1,033336335	0,219416023	-0,04356633	0,97025352	0,567418279	1,001794927
BSU16920	PgsA	-0,02513416	0,982729211	0,508151227	0,287768514	1,220750623	0,035113663	1,101739917
BSU16930	CinA	0,10470845	1,075277069	0,071842638	0,626061902	1,543346394	0,00062829	1,309311731
BSU16940	RecA	0,42244211	1,340194239	0,030292311	0,591775493	1,507100362	0,000879235	1,4236473
BSU16950	PbpX	-1,79298288	0,288574779	5,37E-10	-0,43184111	0,741315143	0,008787832	0,514944961

BSU16960	Rny	0,732496849	1,661512157	0,001398745	0,667066358	1,587840895	7,71E-05	1,624676526
BSU16970	YmdB	0,246302199	1,186162937	0,012262161	0,833604423	1,782132277	7,71E-05	1,484147607
BSU16980	SpoVS	0,253697144	1,192258562	0,278838829	-1,46545923	0,362120251	4,72E-08	0,777189407
BSU16990	Tdh	0,631612632	1,549295816	0,003100683	0,768441152	1,703428216	0,00164889	1,626362016
BSU17000	Kbl	0,688645053	1,611769069	0,002922434	0,383596072	1,304589635	0,077211899	1,458179352
BSU17010	YmcB	-0,48040302	0,716777363	0,020044698	0,276198943	1,211000065	0,126311126	0,963888714
BSU17020	YmcA	0,424118456	1,341752388	0,010163738	-0,0414866	0,971653207	0,732810695	1,156702797
BSU17030	CotE	-1,24549814	0,421762245	7,91E-06	-1,3556588	0,390756345	2,93E-05	0,406259295
BSU17040	MutS	-0,08630068	0,941934942	0,209002909	0,784937558	1,723017743	0,00028554	1,332476343
BSU17050	MutL	0,6707962	1,591951297	0,002827649	-0,13444083	0,911022864	0,749427755	1,251487081
BSU17060	YmzD	0,143900846	1,104888545	0,233554083	0,16633149	1,1222013	0,359740753	1,113544923
BSU17070	YmcC	-1,01393585	0,495193456	0,000510046	-0,57105186	0,673125836	0,011484769	0,584159646
BSU17080	PksA	0,129541543	1,093946014	0,06799404	-0,3098393	0,806731615	0,167955184	0,950338814
BSU17090	PksB	0,372445113	1,294544997	0,004209759	1,317435907	2,492227739	2,51E-06	1,893386368
BSU17100	PksC	0,121200856	1,087639805	0,00395653	1,137832885	2,200502308	9,08E-08	1,644071056
BSU17110	PksD	-0,10117607	0,932272701	0,604611939	1,566928844	2,962733467	1,79E-10	1,947503084
BSU17120	PksE	-0,18219554	0,881360689	0,46480694	1,346406651	2,542780008	2,67E-08	1,712070349
BSU17130	AcpK	-0,05180639	0,964727646	0,677164553	1,382602539	2,60738304	2,65E-09	1,786055343
BSU17140	PksF	-0,22022822	0,85842963	0,055901995	1,508449293	2,845040704	5,05E-10	1,851735167
BSU17150	PksG	0,529112374	1,443041083	0,000549385	1,308436601	2,476729998	6,53E-08	1,95988554
BSU17160	PksH	0,021187712	1,014794575	0,11054166	1,727647527	3,311873405	1,40E-10	2,16333399
BSU17170	PksI	-0,52279821	0,696020541	0,0318805	1,593455085	3,017711913	2,14E-10	1,856866227
BSU17180	PksJ	-0,34478562	0,787424979	0,01867351	1,399880336	2,638796939	3,43E-09	1,713110959
BSU17190	PksL	-0,50521651	0,704554633	0,046949548	1,338032309	2,528062812	5,45E-09	1,616308723
BSU17200	PksM	-0,58034892	0,668802007	0,023963935	1,358951165	2,564986379	3,29E-09	1,616894193
BSU17210	PksN	-0,72143968	0,606491915	0,00180952	1,309728493	2,478948832	6,38E-09	1,542720373
BSU17220	PksR	0,109138671	1,0785841	0,024417084	1,046681703	2,06577296	2,30E-05	1,57217853
BSU17230	PksS	0,821504017	1,767247397	3,12E-05	0,856411057	1,810528714	0,000692603	1,788888055
BSU17240	YmzB	-0,91891053	0,528908282	5,49E-05	-0,80831613	0,571047979	0,000560808	0,549978131

BSU17250	YmaE	0,643077379	1,561656743	0,012391397	0,906119034	1,873997508	0,000256257	1,717827125
BSU17260	AprX	0,460677251	1,376187697	0,064601047	-0,11503054	0,923362762	0,099583202	1,149775229
BSU17270	YmaC	-0,95718419	0,515061215	0,007864047	-0,37463862	0,771298593	0,013762508	0,643179904
BSU17280	YmaD	-1,5583251	0,33954505	2,55E-08	-0,87061032	0,546915434	0,000688286	0,443230242
BSU17290	EbrB	0,201749458	1,150092148	0,348670758	-0,65048269	0,637067131	0,032894619	0,89357964
BSU17300	EbrA	-0,77626	0,583878461	0,142098676	0,291955572	1,22429869	0,395564552	0,904088575
**BSU17310**	**YmaG**	**-3,65644853**	**0,079304771**	**2,11E-14**	**-2,78824363**	**0,144762152**	**7,21E-16**	**0,112033462**
BSU17320	YmaF	-1,50825231	0,351536814	2,27E-07	-0,66994561	0,628530381	0,004335407	0,490033598
BSU17330	MiaA	-1,63991701	0,320874932	1,47E-09	-0,34654029	0,786467856	0,1597132	0,553671394
BSU17340	Hfq	0,322352992	1,2503682	0,014533079	-0,23309212	0,850809401	0,36496701	1,0505888
BSU17350	YmzC	-1,26987304	0,414696267	3,58E-08	-0,58477599	0,666752859	0,003689264	0,540724563
BSU17360	YmzA	-0,86891873	0,547557079	0,00027923	-0,54237673	0,68663879	0,014563297	0,617097934
BSU17370	NrdI	-0,09930017	0,933485704	0,094561894	0,689529593	1,612757575	0,000310398	1,27312164
BSU17380	NrdE	0,089007948	1,063638533	0,041142339	0,915094549	1,885692649	5,19E-05	1,474665591
BSU17390	NrdF	0,356416122	1,280241625	0,006929631	0,658171609	1,578081384	0,000564892	1,429161505
BSU17400	YmaB	0,817890735	1,762826801	3,21E-05	0,242463808	1,183011262	0,283505834	1,472919031
BSU17410	CwlC	-0,06388907	0,956681718	0,000102852	-0,22725557	0,854258399	0,024222075	0,905470058
**BSU17420**	**SpoVK**	**-1,40305524**	**0,37812752**	**3,16E-08**	**-1,3136403**	**0,402304477**	**4,84E-07**	**0,390215999**
BSU17430	YnbA	-1,36552592	0,388092937	4,65E-08	-0,35632534	0,781151705	0,151315827	0,584622321
BSU17440	YnbB	-1,05585991	0,481010434	0,00019056	0,20448605	1,152275784	0,058891359	0,816643109
BSU17450	GlnR	-0,65809967	0,633712479	0,005076591	0,185859104	1,137494133	0,221451072	0,885603306
BSU17460	GlnA	0,596576528	1,512124081	0,001499957	-0,24476694	0,843952121	0,347868703	1,178038101
BSU17470	YnxB	0,656835918	1,576621024	2,17E-05	-0,36633519	0,775750598	0,128360411	1,176185811
BSU17480	YnzF	-0,58775069	0,665379496	0,000415978	-0,21834405	0,859551476	0,336692566	0,762465486
BSU17490	YnzG	-0,4772644	0,718338428	0,053841731	-0,46403934	0,724953646	0,071342926	0,721646037
BSU17500	YnaB	-0,07276546	0,95081366	0,629012151	0,255248955	1,193541686	0,234981852	1,072177673
BSU17510	YnaC	0,951149396	1,933412396	1,66E-05	0,055953768	1,039546122	0,42063165	1,486479259
BSU17520	YnaD	-0,93942999	0,521438859	0,000247953	-0,14667774	0,903328267	0,519432272	0,712383563
BSU17530	YnaE	-0,30072712	0,811843123	0,078082738	0,641848093	1,560326659	0,002087246	1,186084891

BSU17540	YnaF	0,775236605	1,711470714	0,000428512	0,612392227	1,528792098	0,002741695	1,620131406
BSU17550	YnaG	0,747487798	1,67886683	0,000174449	0,335997878	1,262250175	0,058948264	1,470558503
BSU17559	YnzI	0,307121546	1,23723671	0,04319837	0,725945393	1,653984131	0,001504512	1,445610421
BSU17560	YnaI	1,314692063	2,487492305	2,71E-09	0,174695479	1,12872613	0,264787463	1,808109217
BSU17570	XynP	0,26196113	1,199107606	0,034102455	2,133572242	4,388026512	6,17E-14	2,793567059
BSU17580	XynB	0,470815065	1,385892221	0,022487924	0,852973593	1,806219959	5,06E-06	1,59605609
BSU17590	XylR	0,62119237	1,538145915	0,105526908	0,034838916	1,024442433	0,510753579	1,281294174
BSU17600	XylA	-0,3584488	0,780002796	0,113341816	0,454389949	1,370203285	0,015524892	1,07510304
BSU17610	XylB	0,07592519	1,054036765	0,052819903	0,542624985	1,456620429	0,017575183	1,255328597
BSU17620	YncB	-1,73142689	0,301153955	0,046958986	-0,39687048	0,759504026	0,277837143	0,53032899
BSU17630	YncC	0,668970038	1,589937482	0,006853376	0,358929938	1,28247432	0,0845068	1,436205901
BSU17640	YncD	-0,39875997	0,758509961	0,000191972	-0,78011275	0,582321281	0,000359161	0,670415621
BSU17650	YncE	0,845240779	1,796564559	0,001315276	-0,17887262	0,883393045	0,511395264	1,339978802
BSU17660	YncF	0,671397423	1,592614859	0,006114277	0,353973807	1,278076159	0,079837012	1,435345509
BSU17680	ThyA	-0,05623862	0,961768365	0,136703284	0,772336997	1,708034359	0,001081396	1,334901362
BSU17690	YncM	0,828427872	1,775749245	0,000146803	-0,09268208	0,937777725	0,751171953	1,356763485
BSU17699	YnzK	-1,70965872	0,305732385	0,000796489	0,074309909	1,052857297	0,132277802	0,679294841
BSU17700	CotC	0,315124768	1,244119244	0,343844694	0,813312909	1,757242027	0,005538724	1,500680636
BSU17710	TatAC	2,482176764	5,587398676	1,14E-16	-0,62130163	0,650084144	0,013969673	3,11874141
BSU17720	YndA	-0,32657184	0,797429103	0,046043107	-0,31469181	0,804022728	0,300375663	0,800725915
BSU17730	YndB	1,555083084	2,938506488	6,13E-11	-0,70535403	0,613291972	0,000702143	1,77589923
BSU17740	YnzB	-0,15825422	0,896108779	0,304289904	-0,62947295	0,646412522	0,005500842	0,771260651
BSU17750	YndD	-2,58972355	0,166117556	9,39E-14	-0,49852213	0,707831498	0,011845557	0,436974527
BSU17760	YndE	-2,71743409	0,152044539	9,93E-17	-0,49064076	0,71170893	0,029101647	0,431876735
BSU17770	YndF	-2,39081351	0,190674853	2,36E-08	-0,31588559	0,803357704	0,350973963	0,497016279
BSU17780	YndG	1,172854239	2,254573025	1,48E-05	1,171267333	2,252094449	9,17E-07	2,253333737
BSU17790	YndH	0,427295856	1,344710728	0,089973885	1,462076754	2,755046662	2,27E-08	2,049878695
BSU17800	YndJ	0,27290427	1,208237669	0,470447823	1,391778048	2,624018784	1,53E-07	1,916128226
BSU17810	YndK	-0,4768445	0,71854753	0,083089549	-0,41727103	0,748839777	0,053527413	0,733693654

**BSU17820**	**YndL**	**-1,86110493**	**0,275265378**	**1,18E-08**	**-1,63538426**	**0,321884662**	**1,45E-09**	**0,29857502**
**BSU17830**	**YndM**	**-3,92768476**	**0,065712664**	**2,44E-20**	**-2,19089009**	**0,219016263**	**3,23E-14**	**0,142364463**
BSU17840	FosB	-1,35167998	0,391835501	9,61E-07	-0,8694177	0,547367735	4,22E-05	0,469601618
BSU17850	LexA	1,085466577	2,12206166	9,25E-06	0,473203639	1,38818865	0,03542834	1,755125155
BSU17860	YneA	0,170925089	1,12578013	0,04266904	0,417254646	1,335383988	0,289123723	1,230582059
BSU17870	YneB	1,233840818	2,351922983	1,92E-06	0,66515189	1,585735214	0,038351928	1,968829098
BSU17880	YnzC	0,710600178	1,636484772	0,000196809	-0,19302865	0,874767392	0,325153169	1,255626082
BSU17890	Tkt	-0,32489207	0,798358111	0,233410933	-0,06443034	0,956322855	0,78359758	0,877340483
BSU17900	SirA	1,627108972	3,088933841	2,66E-12	0,847650116	1,799567374	1,73E-05	2,444250607
BSU17910	YneF	1,484704155	2,798597781	2,23E-09	0,24491614	1,185023887	0,192331791	1,991810834
BSU17920	YnzD	1,030742397	2,043075328	3,75E-06	0,301247118	1,232209119	0,14404889	1,637642223
BSU17930	CcdA	0,45459549	1,370398512	0,000239212	-0,00930935	0,993568023	0,420865569	1,181983268
BSU17940	YneI	0,878117889	1,83797595	1,66E-06	0,107606883	1,077439516	0,141319648	1,457707733
BSU17950	YneJ	1,215708766	2,322548568	5,51E-07	-0,34259871	0,788619501	0,171153217	1,555584034
BSU17960	YneK	1,474705602	2,779269241	1,64E-09	0,28694961	1,220057896	0,27102292	1,999663569
**BSU17970**	**CotM**	**-2,02886302**	**0,24504812**	**2,03E-12**	**-2,18411953**	**0,220046521**	**4,40E-14**	**0,23254732**
**BSU17980**	**SspP**	**-2,62853758**	**0,161707939**	**1,55E-16**	**-3,76811141**	**0,073398205**	**3,30E-20**	**0,117553072**
**BSU17990**	**SspO**	**-3,05118441**	**0,120642956**	**4,78E-18**	**-3,35235465**	**0,097913076**	**5,96E-19**	**0,109278016**
BSU18000	CitB	1,706882179	3,264545567	5,01E-11	-0,33957015	0,79027674	0,141800772	2,027411153
BSU18010	YneN	2,385776762	5,226252259	1,04E-15	-0,5700299	0,673602829	0,037078198	2,949927544
BSU18019	YnzL	-0,66515283	0,630621895	0,012771809	-0,89428239	0,538014744	0,000103282	0,58431832
**BSU18020**	**SspN**	**-2,01202824**	**0,247924329**	**3,72E-10**	**-2,33620563**	**0,198030476**	**2,91E-14**	**0,222977403**
**BSU18030**	**Tlp**	**-1,96124427**	**0,256806874**	**1,19E-11**	**-2,24291451**	**0,211259115**	**2,13E-14**	**0,234032994**
BSU18040	YneP	-0,72020443	0,607011424	0,000150276	-0,38728704	0,764566007	0,027815052	0,685788716
BSU18050	YneQ	0,874121449	1,832891579	1,36E-08	-0,43811633	0,738097684	0,085520774	1,285494632
BSU18060	YneR	0,851867634	1,804835855	2,52E-05	0,05840529	1,041314088	0,5806251	1,423074971
BSU18070	PlsY	0,969948624	1,95877084	3,02E-05	0,347418808	1,272282291	0,037279486	1,615526566
BSU18080	YneT	0,145023606	1,105748746	0,02558669	-0,69131304	0,619289959	0,002046891	0,862519352
BSU18090	ParE	-0,51909179	0,697810981	0,022222321	0,671878907	1,593146466	0,000476153	1,145478724

BSU18100	ParC	0,755535675	1,688258329	0,002224495	0,008773494	1,006099851	0,101982045	1,34717909
BSU18110	YnfC	1,220801323	2,3307614	7,47E-08	-0,58041235	0,668772604	0,012868338	1,499767002
BSU18120	AlsT	0,477039218	1,391884228	0,002237342	-0,00591467	0,995908658	0,163270814	1,193896443
BSU18130	BglC	-1,27922321	0,412017292	1,03E-08	-0,74065015	0,59846959	0,000403692	0,505243441
**BSU18140**	**YnfE**	**-2,06880015**	**0,238357653**	**9,41E-09**	**-1,31487908**	**0,401959185**	**1,75E-05**	**0,320158419**
BSU18150	XynC	-0,62076358	0,650326636	0,001895328	-0,54133013	0,687137092	0,067165922	0,668731864
BSU18160	XynD	-1,45184897	0,365552628	1,86E-06	0,140387731	1,102201298	0,453721559	0,733876963
BSU18170	YngA	0,807402946	1,750058254	0,131110539	0,902250587	1,868979296	0,010629838	1,809518775
BSU18180	YngB	0,062916625	1,044575394	0,00506171	0,512461071	1,426481534	0,048878624	1,235528464
BSU18190	YngC	1,587233254	3,004725616	6,96E-11	0,293138291	1,225302779	0,091383785	2,115014197
**BSU18200**	**NrnB**	**-2,4243862**	**0,186288923**	**5,60E-15**	**-1,43957768**	**0,368675211**	**1,88E-08**	**0,277482067**
BSU18210	YngE	1,515546838	2,859071774	8,77E-10	-0,12660467	0,915984653	0,521844971	1,887528213
BSU18220	YngF	0,204934611	1,152634103	0,336389993	0,235827483	1,177581966	0,106858542	1,165108035
BSU18230	YngG	-0,01815542	0,987494475	0,604472413	-0,00599921	0,995850297	0,124195739	0,991672386
BSU18239	YngHB	-0,09574001	0,935792127	0,110420816	0,038665559	1,027163298	0,459555079	0,981477712
BSU18240	YngH	0,036345152	1,025512553	0,69076907	-0,04973287	0,9661152	0,454181267	0,995813877
BSU18250	YngI	0,080182064	1,057151442	0,775047486	0,414812451	1,333125361	0,004306754	1,195138401
BSU18260	YngJ	-0,12425092	0,917480298	0,030823534	0,409472048	1,328199672	0,007423589	1,122839985
BSU18270	YnzE	0,216450715	1,161871657	0,433221589	0,077146383	1,05492935	0,561077827	1,108400504
**BSU18280**	**YngK**	**-2,03058447**	**0,244755898**	**6,20E-10**	**-1,67364006**	**0,313461452**	**1,11E-08**	**0,279108675**
**BSU18290**	**YngL**	**-4,16132405**	**0,055887752**	**2,60E-17**	**-2,44584355**	**0,183538731**	**2,88E-13**	**0,119713242**
BSU18300	PpsE	-0,40621649	0,754599741	0,007289516	0,241559878	1,182270271	0,035793245	0,968435006
BSU18310	PpsD	-0,94859634	0,518136336	0,000161847	0,518066683	1,43203493	0,006291198	0,975085633
BSU18320	PpsC	0,263702928	1,20055619	0,488490881	0,080935539	1,057703703	0,245339756	1,129129946
BSU18330	PpsB	-0,47212538	0,72090178	0,022858501	0,760026327	1,693521528	0,000439048	1,207211654
BSU18340	PpsA	-0,3287182	0,796243615	0,163042399	0,874857118	1,833826459	0,000215166	1,315035037
BSU18350	DacC	1,059300124	2,083920331	1,35E-05	0,433879407	1,350861164	0,026425746	1,717390747
BSU18360	GalM	-0,2229347	0,856820733	0,049110045	0,759686478	1,693122641	6,86E-05	1,274971687
BSU18370	YoeA	1,163395786	2,239840166	1,94E-06	0,514721162	1,428717977	0,00714428	1,834279071

BSU18380	IseA	0,167124544	1,122818347	0,16393467	-1,13471197	0,455425833	7,41E-07	0,78912209
BSU18390	YoeC	0,387356767	1,307994765	0,079645368	0,727738222	1,656040806	0,00320404	1,482017786
BSU18400	YoeD	0,548204542	1,46226475	0,055689446	0,764303618	1,698549918	0,006432105	1,580407334
BSU18410	Ggt	0,401959178	1,321301018	0,064081729	0,08701752	1,062172086	0,43650732	1,191736552
BSU18420	YofA	-0,3122935	0,805360433	0,050255255	-0,12436228	0,917409479	0,554947027	0,861384956
BSU18430	YogA	-2,19820043	0,217909284	1,49E-10	-0,67126063	0,627957738	0,00319928	0,422933511
BSU18440	GltB	-0,55744657	0,679503757	0,000723339	-0,09902845	0,933661531	0,185510488	0,806582644
BSU18450	GltA	-1,07405671	0,474981522	6,23E-06	0,073502029	1,052267884	0,362500061	0,763624703
BSU18460	GltC	0,311497432	1,240995112	0,070843991	-0,41731847	0,748815148	0,047255587	0,99490513
BSU18470	ProJ	-0,92985403	0,52491145	4,89E-06	-0,63701408	0,643042467	0,000763889	0,583976958
BSU18480	ProH	-0,9181753	0,529177893	1,34E-05	-1,0018184	0,499370188	5,52E-05	0,514274041
BSU18490	Rtp	1,262319604	2,398811184	0,001870356	0,286680883	1,21983066	0,414333491	1,809320922
BSU18500	YoxD	-0,03661618	0,974938973	0,203125801	-0,52228365	0,696268832	0,039060874	0,835603903
BSU18510	YoxC	1,653607803	3,146194371	1,16E-10	0,285257957	1,218628138	0,208539372	2,182411254
BSU18520	YoxB	0,881178696	1,84187952	6,92E-06	0,425449311	1,342990697	0,033886222	1,592435108
BSU18530	YoaA	0,793025787	1,732704683	3,02E-05	0,292251286	1,224549664	0,066980293	1,478627174
BSU18540	YoaB	-1,31834198	0,400995519	6,77E-07	-0,55283045	0,681681411	0,001344684	0,541338465
BSU18550	YoaC	-1,0497352	0,48305682	7,61E-08	0,77002858	1,705303566	0,000977841	1,094180193
BSU18560	YoaD	-1,19508861	0,436759621	5,35E-07	0,533212746	1,447148272	0,008619849	0,941953947
BSU18570	YoaE	-0,85689931	0,552137957	0,000575747	-0,27008533	0,829270495	0,351554574	0,690704226
BSU18580	YoaF	-0,07366386	0,950221756	0,035224374	-0,20858241	0,86538714	0,431901644	0,907804448
BSU18590	YoaG	0,258638867	1,196349458	0,08420105	0,125340433	1,090765091	0,31649159	1,143557274
**BSU18600**	**YozQ**	**-2,4268896**	**0,185965951**	**1,04E-14**	**-2,04275599**	**0,242699663**	**1,31E-12**	**0,214332807**
BSU18610	YoaH	-0,96056802	0,513854558	0,00033781	-0,53165113	0,691762576	0,008933951	0,602808567
BSU18620	YoaI	-1,83052473	0,281162339	4,46E-08	-0,51972687	0,697503874	0,018739696	0,489333106
BSU18630	YoaJ	-0,07347818	0,950344057	0,495555146	0,72028445	1,647506834	0,001734265	1,298925446
BSU18640	YoaK	-1,31862085	0,400918014	5,52E-06	-0,42224562	0,74626213	0,017329008	0,573590072
BSU18650	PelB	-2,56021592	0,169550163	2,71E-05	0,448343765	1,364472923	0,386206278	0,767011543
BSU18660	YoaM	-1,6092436	0,327770156	5,97E-06	-0,91712808	0,529562151	5,28E-07	0,428666153

BSU18669	YozS	0,762682453	1,69664232	6,57E-05	-0,07705943	0,947987913	0,575415912	1,322315117
**BSU18670**	**OxdD**	**-2,33932352**	**0,197602963**	**4,89E-13**	**-2,04115527**	**0,242969097**	**1,19E-12**	**0,22028603**
BSU18680	YoaO	0,591251539	1,506553116	0,001897935	0,171705862	1,126389555	0,194390513	1,316471336
BSU18690	YoaP	-0,49636414	0,708891072	0,002419008	-0,28366637	0,821500657	0,211205965	0,765195865
**BSU18700**	**YoaQ**	**-2,42151073**	**0,18666059**	**9,72E-15**	**-1,51982243**	**0,348728838**	**1,08E-09**	**0,267694714**
BSU18709	YozT	-0,09742713	0,934698426	0,022372133	-0,9691111	0,510820702	1,25E-05	0,722759564
**BSU18710**	**YozF**	**-2,64399241**	**0,159984894**	**7,06E-17**	**-1,4311884**	**0,370825304**	**7,33E-09**	**0,265405099**
**BSU18720**	**YoaR**	**-2,43369536**	**0,185090742**	**2,05E-15**	**-1,36957566**	**0,387005061**	**2,36E-08**	**0,286047902**
BSU18730	YoaS	-0,57609866	0,670775237	0,031498597	0,366032187	1,288803384	0,086184548	0,979789311
BSU18740	YozG	-0,08260452	0,944351253	0,085628168	0,23387543	1,175989704	0,306703539	1,060170479
BSU18750	YoaT	-0,33178857	0,794550834	0,048714204	-0,2152627	0,861389295	0,35928684	0,827970065
**BSU18760**	**YoaU**	**-2,29967921**	**0,203108256**	**1,63E-11**	**-1,1443872**	**0,452381802**	**1,74E-05**	**0,327745029**
BSU18770	YoaV	-0,62357783	0,649059289	0,010951324	-0,74749588	0,59563652	0,002140331	0,622347904
BSU18780	YoaW	-0,72926146	0,60321263	0,001213892	-0,97080516	0,510221233	1,11E-05	0,556716931
BSU18790	YoaZ	0,120817864	1,087351107	0,138989243	-0,07729361	0,947834048	0,33983269	1,017592578
BSU18800	PenP	0,349102282	1,273767778	0,013428876	0,577934293	1,492710397	0,001405614	1,383239087
BSU18810	YobA	-1,09549958	0,467974044	0,006958154	0,781360515	1,718750955	0,009471485	1,0933625
BSU18820	YobB	0,713161923	1,639393205	3,64E-05	1,223784518	2,335585918	1,08E-07	1,987489562
BSU18830	Pps	-0,26231081	0,833751405	0,001474562	0,021201675	1,014804397	0,472534122	0,924277901
BSU18840	XynA	0,683337205	1,605850073	4,55E-05	0,130239447	1,094475339	0,196477898	1,350162706
BSU18849	YozV	1,359625964	2,566186394	0,044523153	0,030447331	1,021328756	0,483000956	1,793757575
BSU18850	YobD	0,72553766	1,65351675	0,000807218	0,467785636	1,382985122	0,041554495	1,518250936
BSU18860	YozH	0,712154641	1,638248988	0,124039742	1,131267639	2,190511273	0,0019326	1,914380131
BSU18870	YozI	0,406987202	1,325913998	0,227068919	0,645534101	1,564318307	0,013696358	1,445116152
BSU18880	YobE	0,083849013	1,059841859	0,150377029	-0,27890415	0,824216843	0,115344879	0,942029351
BSU18890	YobF	-0,07416302	0,949893043	0,710751879	0,305211525	1,235599785	0,049010523	1,092746414
BSU18898	YozW	-1,22818648	0,426853679	2,04E-05	-0,10644372	0,928874941	0,452057451	0,67786431
BSU18899	YozX	-1,60404241	0,328953961	0,104392875	-0,73114027	0,60242758	0,1402866	0,46569077
BSU18900	YozJ	0,299463209	1,23068642	0,001341952	-0,08116219	0,945295841	0,450611783	1,087991131

BSU18908	YozY	1,766440431	3,402135097	1,14E-11	0,450341825	1,366363958	0,012566512	2,384249527
BSU18910	RapK	0,923626856	1,896877951	1,40E-07	-0,04680175	0,968080046	0,37365117	1,432478999
BSU18920	PhrK	0,821713202	1,767503659	2,57E-06	0,008933937	1,006211746	0,768794116	1,386857703
BSU18930	YobH	1,387173461	2,615657169	4,09E-06	-0,78797699	0,579155639	0,063574039	1,597406404
BSU18940	YozK	-0,63846191	0,642397459	0,526619915	-0,80351159	0,572952883	0,700265248	0,607675171
BSU18950	YozL	-0,51144768	0,70151814	0,521793366	0,142151393	1,103549538	0,817295372	0,902533839
BSU18960	YozM	1,128221297	2,185890749	0,018083569	0,723533291	1,651221077	0,011428607	1,918555913
BSU18970	YobI	0,420963086	1,338821001	0,040254207	0,30753819	1,23759407	0,090555785	1,288207536
BSU18980	YobJ	1,507018313	2,842220164	1,01E-09	0,012821537	1,008926821	0,600504453	1,925573492
BSU18990	YobK	1,023695451	2,033120115	6,30E-06	0,723487816	1,651169029	0,003221955	1,842144572
BSU19000	YobL	0,062272681	1,044109254	0,000165057	0,353067957	1,277273922	0,042801423	1,160691588
BSU19010	YobM	0,381881231	1,303039873	0,027751626	0,537166365	1,451119535	0,032097427	1,377079704
BSU19020	YobN	0,278581959	1,213002025	0,011192264	0,682137936	1,604515733	0,000145963	1,408758879
BSU19030	YobO	0,564399352	1,478771713	0,001517581	0,990705252	1,987156161	1,24E-06	1,732963937
BSU19040	CsaA	-0,32583152	0,797838409	0,227465364	-0,03657679	0,97496559	0,753898547	0,886402
BSU19050	YobQ	-0,46506269	0,724439598	0,042387478	0,683089965	1,605574896	0,001344515	1,165007247
BSU19060	YobR	-0,05615275	0,961825613	0,036144606	0,173840978	1,12805779	0,064675178	1,044941701
BSU19070	YobS	-0,7844944	0,580555381	0,010430307	0,463403988	1,378791192	0,012405069	0,979673287
BSU19080	YobT	-0,55679244	0,67981192	0,020990034	0,241747235	1,182423818	0,332851	0,931117869
BSU19090	YobU	-1,36202681	0,389035358	0,2954447	1,126068508	2,182631396	0,128852486	1,285833377
BSU19100	YobV	-1,16250817	0,446735196	0,000676158	-1,37541283	0,385442395	1,65E-05	0,416088795
BSU19110	YobW	0,178631068	1,131809434	0,148391081	-0,8756271	0,54501691	0,000381374	0,838413172
BSU19120	CzrA	-0,14645472	0,903467923	0,392759861	-0,75731803	0,591595083	0,000404046	0,747531503
BSU19130	YocA	0,522749064	1,436690263	0,0274844	0,216960314	1,162282135	0,147783845	1,299486199
BSU19140	YozB	1,680909719	3,206300667	6,51E-10	0,211094233	1,157565823	0,10510037	2,181933245
BSU19150	YocB	2,085184368	4,243293199	6,39E-15	0,992162105	1,989163832	5,45E-06	3,116228515
BSU19160	YocC	-0,94063814	0,521002375	5,20E-07	-0,07452543	0,949654459	0,131412485	0,735328417
BSU19170	YocD	0,078553042	1,055958431	0,133754497	-0,2651728	0,832099066	0,118349919	0,944028749
BSU19180	Des	0,262418279	1,19948763	0,001869624	-1,31449781	0,402065426	4,08E-08	0,800776528

BSU19190	DesK	-0,55836483	0,679071398	0,111813585	-0,32697325	0,797207258	0,113585165	0,738139328
BSU19200	DesR	-0,2493045	0,841301898	0,003005294	-0,19134454	0,87578914	0,274178442	0,858545519
BSU19210	YocH	-0,04895512	0,966636168	0,029549525	-0,55047056	0,682797385	0,62150798	0,824716776
BSU19220	YocI	-0,36694134	0,775424735	0,058881881	0,213782266	1,159724614	0,098463078	0,967574674
BSU19230	AzoR1	-0,73337887	0,601493535	0,007263753	-0,96052341	0,513870448	0,000611375	0,557681992
BSU19240	YocK	0,303595235	1,234216285	0,061642019	-0,80729275	0,571453196	0,000453051	0,90283474
BSU19250	YocL	-1,05196086	0,482312178	1,23E-05	-0,87109082	0,546733311	0,000325759	0,514522744
**BSU19259**	**YoyB**	**-1,71747224**	**0,304081037**	**4,74E-11**	**-1,34887161**	**0,392598998**	**1,27E-07**	**0,348340017**
BSU19260	YocM	-0,14454397	0,904665294	0,533390169	-0,8650008	0,549046103	0,000561869	0,726855698
**BSU19270**	**YozN**	**-1,22971904**	**0,426400478**	**9,30E-09**	**-1,42677894**	**0,371960432**	**2,59E-07**	**0,399180455**
**BSU19280**	**YocN**	**-1,06368441**	**0,478408718**	**1,04E-06**	**-1,57323335**	**0,33605439**	**8,74E-08**	**0,407231554**
BSU19290	YozO	0,541612528	1,455598559	0,003061972	-0,72867828	0,603456515	0,000643353	1,029527537
BSU19300	YozC	0,629066842	1,546564328	0,000458559	-0,5343789	0,690455863	0,012834657	1,118510096
BSU19310	DhaS	1,015328326	2,021362858	3,74E-06	0,681451673	1,603752677	8,60E-06	1,812557768
BSU19320	SqhC	-1,8622412	0,275048664	1,13E-12	-0,7049256	0,613474123	0,014372916	0,444261394
BSU19330	SodF	-2,03289758	0,24436379	2,37E-13	-0,66860505	0,629114688	0,006453016	0,436739239
BSU19340	YocR	0,680138436	1,602293499	0,000101748	-0,54559196	0,685110233	0,002209035	1,143701866
BSU19350	YocS	1,550449863	2,929084601	1,51E-10	0,47406901	1,389021577	0,011537301	2,159053089
BSU19360	OdhB	0,710763264	1,636669776	0,001644469	-0,10543554	0,929524282	0,470180598	1,283097029
BSU19370	OdhA	0,941824088	1,920955486	1,93E-05	0,465455172	1,380752914	0,000703589	1,6508542
BSU19380	YojO	-0,30568442	0,809058304	0,019339768	-0,3018164	0,811230386	0,189762694	0,810144345
BSU19390	YojN	-0,83732092	0,559681932	0,000443808	0,135369135	1,098373813	0,473189777	0,829027873
BSU19400	YojM	0,610479682	1,526766759	0,000350998	-0,23907827	0,847286469	0,423234964	1,187026614
BSU19410	CwlS	0,665705876	1,586344243	0,000551918	0,277639721	1,21221006	0,107970139	1,399277152
BSU19420	YojK	-0,89234821	0,538736527	0,008134285	0,198318895	1,14736061	0,095399209	0,843048569
**BSU19430**	**YojJ**	**-1,86008968**	**0,275459156**	**2,78E-12**	**-1,54367515**	**0,343010549**	**5,63E-10**	**0,309234852**
BSU19440	YojI	-1,79410351	0,288350712	5,13E-11	-0,17654143	0,884821635	0,193699429	0,586586173
BSU19450	RsbRC	-1,10281938	0,465605699	1,61E-05	0,227838518	1,171079094	0,106834626	0,818342397
BSU19460	BshB2	-0,92410545	0,527007189	2,31E-05	-0,60962923	0,655365106	0,010584735	0,591186148

BSU19470	YojF	-1,20985613	0,432311725	2,24E-05	-0,48886304	0,712586451	0,020940868	0,572449088
BSU19479	YoyC	-1,70218658	0,30731997	2,75E-09	-0,21536759	0,861326674	0,40308688	0,584323322
BSU19480	YojE	-0,22006202	0,858528527	0,344540804	0,092882882	1,066499199	0,775270927	0,962513863
**BSU19490**	**GerT**	**-2,46558545**	**0,181044287**	**6,28E-13**	**-2,71553044**	**0,152245296**	**1,20E-16**	**0,166644791**
BSU19510	YojB	0,596230074	1,511760997	0,001530812	-1,20030891	0,435182089	8,58E-08	0,973471543
BSU19520	YojA	0,773690163	1,70963715	0,010819356	1,215787666	2,32267559	0,000931921	2,01615637
BSU19530	YodA	-0,06989336	0,952708417	0,616885715	-0,37668536	0,770205131	0,236543846	0,861456774
BSU19540	YodB	-0,56994217	0,673643791	0,008620342	-0,10653176	0,928818257	0,618625527	0,801231024
BSU19550	YodC	0,710867865	1,636788444	5,63E-06	-0,21984991	0,858654763	0,316438261	1,247721604
BSU19560	MhqD	-0,29027156	0,81774812	0,06118439	-0,30396689	0,810022064	0,136469425	0,813885092
BSU19570	MhqE	-1,06897662	0,476656996	5,16E-05	-0,35612499	0,781260192	0,078620455	0,628958594
BSU19579	YoyD	-0,47706011	0,718440155	0,011030254	0,002455555	1,00170351	0,776095427	0,860071832
BSU19580	YodF	-0,86094611	0,550591367	3,69E-05	-0,1031317	0,931009826	0,83914279	0,740800596
BSU19590	CtpA	-0,22692238	0,85445571	0,044542996	-0,32577848	0,797867738	0,278034629	0,826161724
BSU19600	YodH	-1,1590677	0,447801821	2,38E-05	-1,39611034	0,379952153	4,66E-07	0,413876987
BSU19610	YodI	-0,53917928	0,688162278	0,082298905	-0,93548964	0,522864982	3,78E-05	0,60551363
BSU19620	YodJ	-0,7547209	0,592661031	0,001807664	0,140675734	1,10242135	0,176411415	0,847541191
BSU19630	DeoD	0,164228067	1,120566344	0,197995161	0,482410858	1,397076342	0,052327166	1,258821343
**BSU19639**	**YoyE**	**-2,20336992**	**0,217129865**	**9,74E-14**	**-1,9727519**	**0,254766608**	**1,07E-12**	**0,235948236**
BSU19640	YodL	1,303423565	2,468138861	9,43E-09	0,481178332	1,395883299	0,005675266	1,93201108
BSU19650	YodM	0,650402942	1,569606523	0,001281148	-0,17068746	0,888419241	0,285473513	1,229012882
BSU19660	YozD	-0,07514194	0,949248723	0,023686882	-1,17571955	0,442662924	4,36E-07	0,695955823
BSU19669	YoyF	0,709218203	1,634917915	0,001733143	-0,51314962	0,700691054	0,005690945	1,167804485
BSU19670	YodN	-0,01007438	0,993041296	0,000198565	-0,17321561	0,886863752	0,214456713	0,939952524
BSU19680	YozE	-0,16248354	0,893485646	0,14272649	-0,49892621	0,707633275	0,022533899	0,800559461
BSU19689	YokU	-0,44360572	0,735294593	0,025954626	-0,58409001	0,667069963	0,111412892	0,701182278
BSU19690	KamA	-0,4607254	0,726620816	0,008706809	-0,37206945	0,772673353	0,377091729	0,749647085
BSU19700	YodP	-0,83036249	0,562387919	4,17E-05	0,119220087	1,086147538	0,094781228	0,824267728
BSU19710	YodQ	-0,71717992	0,608285316	0,000191566	0,089821103	1,064238206	0,098149139	0,836261761

BSU19720	YodR	0,052940892	1,037377434	0,141639353	0,114036046	1,082251684	0,089759127	1,059814559
BSU19730	YodS	-0,31290555	0,805018841	0,102504645	0,136598884	1,099310463	0,062601931	0,952164652
BSU19740	YodT	0,245430564	1,185446508	0,027658162	0,136305609	1,099087015	0,037254813	1,142266761
BSU19749	YoyG	-0,50256104	0,705852657	0,006980435	-0,65689172	0,634243299	0,002491309	0,670047978
BSU19750	CgeE	-0,55651998	0,679940315	0,020201562	-0,16940916	0,889206771	0,457953466	0,784573543
BSU19760	CgeD	-0,40059172	0,757547511	0,071621781	-0,1841852	0,880146024	0,520579748	0,818846768
BSU19770	CgeC	-0,85479857	0,552942525	0,005334038	-0,55846518	0,679024162	0,009720468	0,615983344
BSU19780	CgeA	-0,76870017	0,586946059	0,005762856	-0,65110517	0,636792314	0,004176633	0,611869187
BSU19790	CgeB	-1,17143888	0,443978314	0,001949849	-0,41864924	0,748124746	0,021439749	0,59605153
BSU19800	Phy	-1,27764015	0,412469643	9,21E-07	-0,3492148	0,785011233	0,191903105	0,598740438
BSU19820	YotN	0,076046605	1,054125475	1	0,080935539	1,057703703	1	1,055914589
BSU19830	YotM	-0,09749604	0,934653782	0,382952303	-0,61344422	0,653634383	0,159549967	0,794144082
BSU19840	YotL	0,036269408	1,025458714	0,733113383	0,080935539	1,057703703	0,471179771	1,041581208
BSU19850	YotK	0,873333745	1,831891103	0,02704583	-0,29339922	0,815977221	0,284805446	1,323934162
BSU19860	YotJ	-0,24962078	0,841117481	0,001655399	-0,19368326	0,874370563	0,440097085	0,857744022
BSU19870	YotI	0,109038595	1,078509284	1	-0,19257202	0,875044311	0,998127515	0,976776798
BSU19880	YotH	1,776985571	3,427093542	0,001486612	0,080935539	1,057703703	0,635823961	2,242398623
BSU19890	YotG	0,183526597	1,135656552	1	0,080935539	1,057703703	0,871012787	1,096680127
BSU19900	YotF	0,061410389	1,043485381	1	0,235856614	1,177605745	0,847122258	1,110545563
BSU19910	YotE	0,944709176	1,924800838	0,026049836	-1,13999814	0,453760162	0,089921533	1,1892805
BSU19920	YotD	0,085813015	1,061285649	1	0,080935539	1,057703703	1	1,059494676
BSU19930	YotC	1,065077512	2,092282289	0,064886069	0,080935539	1,057703703	0,586212235	1,574992996
BSU19940	YotB	1,165535922	2,243165277	0,005274195	-0,63935994	0,641997713	0,51253838	1,442581495
**BSU19950**	**SspC**	**-2,20813955**	**0,216413207**	**3,76E-09**	**-2,03426845**	**0,244131701**	**7,53E-09**	**0,230272454**
BSU19970	YosX	0,755278048	1,687956878	0,027964563	-0,44047389	0,736892519	0,065394943	1,212424698
BSU19980	YosW	0,125058217	1,090551738	0,128373549	0,431324351	1,348470865	0,491788503	1,219511302
BSU19990	YosV	-0,47886862	0,717540108	0,871717428	-0,90183396	0,535205943	0,636543677	0,626373026
BSU19999	YojW	0,872562616	1,830912209	0,102992193	0,686076033	1,608901532	0,189041374	1,71990687
BSU20000	YosU	0,094478884	1,067679682	1	0,080935539	1,057703703	0,899506231	1,062691692

BSU20010	YosT	-0,1335694	0,911573319	0,208600548	-0,25134107	0,840115115	0,571479275	0,875844217
BSU20020	YosS	0,198859602	1,14779071	0,056987039	-0,03285724	0,977482489	0,258410885	1,062636599
BSU20030	YosR	-0,40227426	0,756664538	0,90422239	-0,94838463	0,518212374	0,42668255	0,637438456
BSU20040	YosP	0,129960757	1,094263936	1	-0,008546	0,994093875	0,725934342	1,044178905
BSU20050	YosQ	0,057514586	1,040671391	1	0,080935539	1,057703703	0,372515764	1,049187547
BSU20060	NrdEB	0,072908576	1,051835122	0,565153675	0,080935539	1,057703703	0,593462773	1,054769413
BSU20070	YosM	1,367636657	2,580475006	7,33E-05	-0,38979259	0,763239324	0,015973302	1,671857165
BSU20080	YosL	-0,69323426	0,618465806	0,555700493	0,272866712	1,208206215	0,630332279	0,91333601
BSU20090	YosK	0,715882425	1,642487539	2,52E-05	0,080935539	1,057703703	0,743995855	1,350095621
BSU20100	YosJ	-0,50541493	0,704457742	0,365659915	0,080935539	1,057703703	0,817621245	0,881080723
BSU20110	YosI	0,079533533	1,05667633	0,878060847	0,080935539	1,057703703	1	1,057190017
BSU20120	YosH	0,145293591	1,105955694	1	0,080935539	1,057703703	0,868381346	1,081829699
BSU20130	YosG	0,144246905	1,105153606	7,76E-05	0,080935539	1,057703703	0,226615339	1,081428655
BSU20140	YosF	0,094147635	1,067434566	1	0,080935539	1,057703703	1	1,062569135
BSU20150	YosE	-0,584814	0,666735291	0,54254268	-0,17302343	0,886981899	0,484527883	0,776858595
BSU20160	YosD	0,050111374	1,035344848	0,831919666	-0,06065918	0,958825927	0,651484689	0,997085387
BSU20170	YosC	0,169519651	1,124683956	1	0,080935539	1,057703703	1	1,09119383
BSU20180	YosB	0,371378147	1,293587952	0,054248107	-0,85717419	0,552032768	0,28274894	0,92281036
BSU20190	YosA	0,102564791	1,073680532	0,763924406	0,080935539	1,057703703	1	1,065692118
BSU20200	YorZ	0,055673079	1,039343888	0,432684486	0,080935539	1,057703703	1	1,048523796
BSU20210	YorY	1,402023938	2,64272066	6,99E-10	-0,42696699	0,743823899	0,117286304	1,69327228
BSU20220	YorX	1,774973479	3,42231719	0,000279319	0,214062668	1,159950039	0,563309288	2,291133615
BSU20230	YorW	1,623414709	3,081034227	9,23E-07	-0,24768717	0,842245565	0,130627415	1,961639896
BSU20240	YorV	0,196071166	1,145574406	0,011375613	-0,41040825	0,752410429	0,170256945	0,948992418
BSU20250	MtbP	0,004185363	1,002905285	0,729533839	0,080935539	1,057703703	0,892396095	1,030304494
BSU20260	YorT	0,077798258	1,055406123	1	0,080935539	1,057703703	1	1,056554913
BSU20270	YorS	0,072391035	1,051457863	1	0,080935539	1,057703703	0,021191922	1,054580783
BSU20280	YorR	0,284430293	1,21792922	0,838242581	0,080935539	1,057703703	0,053829411	1,137816462
BSU20290	YorQ	0,289558136	1,222265869	0,586570176	-0,30603525	0,808861585	0,439405228	1,015563727

BSU20300	YorP	-0,03328935	0,977189763	0,789837419	0,080935539	1,057703703	1	1,017446733
BSU20310	YorO	-0,04268811	0,970844333	1	0,080935539	1,057703703	0,785321328	1,014274018
BSU20320	YorN	1,74381286	3,349191479	3,81E-05	-0,04685308	0,968045607	0,850569407	2,158618543
BSU20330	YorM	0,835729	1,784758657	0,012863269	-0,34914379	0,785049871	0,074712694	1,284904264
BSU20340	YorL	0,811401782	1,754915763	1,55E-05	-0,26096723	0,83452824	0,484346283	1,294722001
BSU20350	YorK	0,075075341	1,053416046	0,963069033	0,080935539	1,057703703	0,882297515	1,055559875
BSU20360	YorJ	-0,05190376	0,964662535	0,67472438	-0,11702042	0,922090067	0,322300762	0,943376301
BSU20370	YorI	0,287148023	1,220225701	0,908987669	0,080935539	1,057703703	0,923328451	1,138964702
BSU20380	YorH	0,090320852	1,064606922	0,945183386	-0,22121384	0,85784337	0,995132457	0,961225146
BSU20390	YorG	0,287651652	1,220651743	0,000284484	0,080935539	1,057703703	0,153744722	1,139177723
BSU20400	YorF	-0,00588876	0,995926542	1	0,215304514	1,160948934	0,681097852	1,078437738
BSU20410	YorE	0,149657607	1,10930617	0,280534288	0,080935539	1,057703703	0,986357321	1,083504937
BSU20420	YorD	-0,0891027	0,940107279	0,760207779	0,080935539	1,057703703	0,01404143	0,998905491
BSU20430	YorC	0,427465053	1,344868443	0,802823883	0,080935539	1,057703703	0,516509651	1,201286073
BSU20440	YorB	-0,5878806	0,665319579	0,588232108	0,133795138	1,097176128	0,450744213	0,881247854
BSU20450	YorA	0,490274178	1,40471181	0,367781352	0,080935539	1,057703703	0,493203298	1,231207757
BSU20460	YoqZ	0,277279047	1,211907045	0,150402954	0,080935539	1,057703703	0,682840989	1,134805374
BSU20470	YoqY	-0,66965316	0,628657804	0,006590935	0,080935539	1,057703703	0,285723966	0,843180754
BSU20480	YoqX	1,559648058	2,947819233	3,11E-07	-0,33564842	0,7924279	0,293895944	1,870123566
BSU20490	YoqW	-0,36220898	0,777972478	0,039129428	0,329698903	1,256751057	0,173916929	1,017361768
BSU20500	LigB	0,121958717	1,088211302	1	0,080935539	1,057703703	0,830068853	1,072957503
BSU20510	YoqU	1,555350127	2,939050458	7,04E-08	-0,11587662	0,922821411	0,752190241	1,930935935
BSU20520	YoqT	0,207612348	1,154775455	1	0,080935539	1,057703703	1	1,106239579
BSU20530	YoqS	0,178178487	1,131454435	0,158501731	0,080935539	1,057703703	0,912139952	1,094579069
BSU20540	YoqR	1,397058111	2,633639925	2,27E-09	-0,08883123	0,940284193	0,476917526	1,786962059
BSU20550	YoqP	1,334622538	2,522094859	4,25E-05	-0,24222139	0,845442541	0,096934661	1,6837687
BSU20560	YoqO	0,144733122	1,105526128	0,914753983	0,080935539	1,057703703	1	1,081614915
BSU20570	YoqN	0,226641437	1,17010779	0,168617121	-0,35002542	0,784570274	0,11717586	0,977339032
BSU20580	YoqM	0,07891543	1,056223708	1	0,080935539	1,057703703	1	1,056963706

BSU20590	YoqL	0,069540985	1,049382754	0,916433666	-0,13721974	0,909269751	0,984322568	0,979326252
BSU20600	YoqK	1,685883957	3,217374684	2,39E-12	0,06683672	1,047417577	0,44055944	2,132396131
BSU20610	YoqJ	0,252973775	1,191660912	2,47E-05	-0,41638984	0,749297302	0,099334397	0,970479107
BSU20620	YoqI	0,224341222	1,168243671	0,449822175	0,080935539	1,057703703	1	1,112973687
BSU20630	YoqH	1,997772926	3,993830004	0,00094994	0,565393498	1,47979107	0,1705233	2,736810537
BSU20640	YoqG	0,506250605	1,420354061	1	0,080935539	1,057703703	0,770138221	1,239028882
BSU20650	YoqF	1,214547626	2,320680039	0,000521014	-0,10182135	0,931855814	0,620670402	1,626267926
BSU20660	YoqE	-0,83476842	0,560673031	0,689242444	0,080935539	1,057703703	0,853225712	0,809188367
BSU20670	YoqD	0,110304413	1,079455981	0,866176111	0,080935539	1,057703703	0,939324908	1,068579842
BSU20680	YoqC	1,074603798	2,10614359	0,000372929	-0,29358135	0,815874216	0,179945455	1,461008903
BSU20690	YoqB	0,063747619	1,045177244	0,773762766	0,080935539	1,057703703	1	1,051440474
BSU20700	YoqA	1,354222252	2,556592548	0,00254664	0,327743992	1,255049261	0,150831117	1,905820905
BSU20710	YopZ	0,057248686	1,040479605	0,909634887	0,119495224	1,086354698	0,251261691	1,063417152
BSU20720	YopY	0,821460398	1,767193967	0,000996931	-0,03281957	0,977508013	0,5236049	1,37235099
BSU20730	YopX	0,282850779	1,216596517	0,597260706	0,098230718	1,070459874	0,754562218	1,143528196
BSU20740	YopW	0,050145928	1,035369646	1	0,080935539	1,057703703	0,852555465	1,046536675
BSU20750	YopV	1,565544951	2,959892851	4,32E-06	-0,26248128	0,833652894	0,305493483	1,896772872
BSU20760	YopU	0,079350821	1,056542514	1	0,080935539	1,057703703	0,998534748	1,057123109
BSU20770	YopT	0,136992358	1,099610325	1	0,080935539	1,057703703	0,895138277	1,078657014
BSU20780	YopS	0,157128285	1,115065366	0,898458312	0,080935539	1,057703703	1	1,086384535
BSU20790	YopR	0,173663252	1,127918832	1	0,080935539	1,057703703	1	1,092811268
BSU20800	YopQ	0,421658026	1,33946606	0,13176085	0,080935539	1,057703703	0,672641585	1,198584881
BSU20810	YopP	0,032895132	1,023063102	0,212219065	-0,21637978	0,860722576	0,5426381	0,941892839
BSU20820	YopO	1,102769803	2,147666234	0,266629329	-0,32982265	0,795634286	0,353932134	1,47165026
BSU20830	YopN	-0,04896413	0,96663013	0,845209018	0,080935539	1,057703703	0,868939704	1,012166917
BSU20840	YopM	0,078596714	1,055990396	0,232883549	0,080935539	1,057703703	1	1,05684705
BSU20850	YopL	0,10884652	1,078365705	1	0,080935539	1,057703703	0,881048483	1,068034704
BSU20860	YopK	0,076017594	1,054104278	0,206268772	0,080935539	1,057703703	0,895968694	1,055903991
BSU20870	YopJ	-0,52683986	0,694073399	0,676231892	0,080935539	1,057703703	0,908977592	0,875888551

BSU20880	YopI	0,07640267	1,054385672	0,93261886	-0,67366529	0,626911939	0,035119103	0,840648805
BSU20890	YopH	1,583776074	2,997533909	6,47E-09	-0,11587817	0,922820416	0,242235072	1,960177163
BSU20900	YopG	0,850317767	1,802897986	0,001149033	-0,28961521	0,818120237	0,222258715	1,310509111
BSU20910	YopF	-0,53749068	0,688968211	0,461348107	0,080935539	1,057703703	0,491989459	0,873335957
BSU20920	YopE	0,035789467	1,025117631	0,704663959	0,080935539	1,057703703	0,740336651	1,041410667
BSU20928	YoyH	1,456236825	2,743916967	5,34E-08	-0,31003233	0,806623684	0,169772396	1,775270326
BSU20929	YoyI	-0,03972964	0,972837241	0,960843854	0,080935539	1,057703703	0,512121322	1,015270472
BSU20930	YopD	-1,05203003	0,482289052	0,366161892	0,080935539	1,057703703	0,574135851	0,769996378
BSU20940	YopC	0,180747012	1,133470632	1	0,080935539	1,057703703	1	1,095587168
BSU20950	YopB	0,094991836	1,068059364	1	0,12636905	1,091543065	0,800546992	1,079801214
BSU20960	YopA	0,11435972	1,082494519	1	-0,19317461	0,874678896	0,698863806	0,978586708
BSU20970	YonX	0,496955975	1,411232777	0,021387106	-0,53323111	0,691005402	0,427028311	1,05111909
BSU20980	YonV	0,232472199	1,17484644	1	0,080935539	1,057703703	1	1,116275072
BSU20990	YonU	0,427210893	1,344631538	1	0,080935539	1,057703703	1	1,20116762
BSU20999	YoyJ	0,560640162	1,474923535	0,118771328	-0,03963341	0,972902134	0,606507221	1,223912835
BSU21000	YonT	1,751368012	3,366776639	0,003747132	0,276718713	1,21143644	0,467335354	2,28910654
BSU21010	YonS	0,203355418	1,151373105	0,623388866	0,401566594	1,320941516	0,481983691	1,236157311
BSU21020	YonR	0,362734126	1,28586049	0,308304264	0,080935539	1,057703703	0,252514817	1,171782097
BSU21030	YonP	0,061314425	1,043415974	1	0,080935539	1,057703703	0,710406416	1,050559838
BSU21040	YonO	3,595303278	12,08632116	1,30E-06	0,934193302	1,91082188	0,001492288	6,998571522
BSU21050	YonN	0,113763929	1,082047572	0,823982316	0,080935539	1,057703703	0,999342863	1,069875638
BSU21060	YonK	0,207535842	1,154714219	1	0,470257612	1,38535682	0,013953741	1,270035519
BSU21070	YonJ	0,52348335	1,437421679	0,044722991	0,080935539	1,057703703	0,538576808	1,247562691
BSU21080	YonI	0,157991686	1,115732893	1	-0,56667932	0,675169051	0,723723887	0,895450972
BSU21090	YonH	1,436739094	2,707082948	0,036266348	0,102229204	1,073430811	0,702758981	1,890256879
BSU21100	YonG	1,025742154	2,036006481	0,045024135	0,139318248	1,101384529	0,064784082	1,568695505
BSU21110	YonF	0,070303039	1,049937201	0,958837076	0,080935539	1,057703703	0,713665759	1,053820452
BSU21120	YonE	-1,01430084	0,495068191	0,578632944	0,080935539	1,057703703	0,787197022	0,776385947
BSU21130	YonD	0,083713374	1,05974222	0,763068489	0,080935539	1,057703703	0,488248059	1,058722961

BSU21140	YonC	-0,05725763	0,96108929	0,935032753	0,080935539	1,057703703	1	1,009396497
BSU21150	YonB	0,678784179	1,600790132	0,681582698	0,061948458	1,043874633	0,665437128	1,322332382
BSU21160	YonA	-0,19463123	0,873796223	0,863344546	-0,14225813	0,9060998	0,793831111	0,889948011
BSU21170	YomZ	0,070510063	1,050087875	0,932558686	0,080935539	1,057703703	0,715667009	1,053895789
BSU21180	YomY	0,065582811	1,046507616	1	0,080935539	1,057703703	1	1,05210566
BSU21190	YomX	0,671280342	1,592485617	0,500483598	0,040180036	1,028242134	0,687569712	1,310363875
BSU21200	YomW	0,105689549	1,076008556	1	0,080935539	1,057703703	1	1,066856129
BSU21210	YomV	0,458455793	1,374070277	0,873679068	0,080935539	1,057703703	1	1,21588699
BSU21220	YomU	0,267236078	1,203499947	0,085325903	0,080935539	1,057703703	0,072228494	1,130601825
BSU21229	YouA	0,257356351	1,195286409	0,708716931	0,080935539	1,057703703	0,465481263	1,126495056
BSU21230	YomT	0,140689138	1,102431593	1	0,080935539	1,057703703	1	1,080067648
BSU21240	YomS	-0,86572514	0,548770509	0,546204717	0,298906155	1,230211318	0,293954309	0,889490914
BSU21250	YomR	1,347648702	2,544970092	1,65E-05	-0,05785458	0,960691695	0,825214823	1,752830894
BSU21260	YomQ	-0,99579708	0,501458746	3,35E-05	-0,51797922	0,698349323	0,369879476	0,599904034
BSU21270	YomP	0,069488539	1,049344606	0,778298329	0,080935539	1,057703703	1	1,053524155
BSU21280	YomO	0,225703583	1,169347385	0,781456705	0,080935539	1,057703703	0,653179644	1,113525544
BSU21290	YomN	0,197427591	1,146651984	0,857100197	0,080935539	1,057703703	0,329787082	1,102177844
BSU21300	YomM	0,559801632	1,474066522	0,288107256	0,080935539	1,057703703	0,714954416	1,265885112
BSU21310	YozP	1,28630042	2,439018024	0,009017051	-0,01330584	0,990819495	0,392856798	1,714918759
BSU21320	YomL	1,065279548	2,092575314	0,002330671	0,409638435	1,328352863	0,177468689	1,710464089
BSU21329	YouB	1,293200914	2,450711934	2,86E-06	0,881816066	1,842693427	0,000421254	2,146702681
BSU21330	YomK	0,067250884	1,047718309	1	0,080935539	1,057703703	0,600873806	1,052711006
BSU21340	YomJ	0,06783191	1,048140349	0,709847386	-0,24562656	0,843449407	0,629424445	0,945794878
BSU21350	CwlP	-0,3908625	0,762673513	0,728007978	0,080935539	1,057703703	0,219003076	0,910188608
BSU21360	YomH	1,431084239	2,696492907	7,19E-06	0,024339695	1,01701411	0,120134728	1,856753508
BSU21370	YomG	0,166761664	1,122535962	0,00089756	0,080935539	1,057703703	0,77501673	1,090119833
BSU21380	YomF	0,1285393	1,093186312	0,000972538	0,65333532	1,572800102	0,653789109	1,332993207
BSU21390	YomE	0,079603662	1,056727696	0,835176839	0,080935539	1,057703703	0,003261104	1,057215699
BSU21400	YomD	-0,04751936	0,967598637	1	-0,02337285	0,983929705	0,95986035	0,975764171

BSU21410	BlyA	0,51999614	1,433951412	0,785650841	0,811388638	1,754899774	0,30803778	1,594425593
BSU21420	BhlA	-0,04131748	0,971767117	0,843462178	0,080935539	1,057703703	1	1,01473541
BSU21430	BhlB	0,077284338	1,055030231	0,99654588	-0,37975744	0,768566798	0,763512053	0,911798514
BSU21440	BdbB	1,296509846	2,45633928	0,371190901	-0,90323233	0,534687432	0,444487263	1,495513356
BSU21450	SunS	0,825468436	1,77211034	0,090634023	-0,16279997	0,893289699	0,455998697	1,332700019
BSU21460	BdbA	1,3563472	2,560360931	6,59E-07	0,043441466	1,030569261	0,367635823	1,795465096
BSU21470	SunT	0,16103494	1,118088929	1	0,080935539	1,057703703	0,668664917	1,087896316
BSU21480	SunA	1,442050053	2,717066834	2,55E-05	-0,18415537	0,880164221	0,307630459	1,798615528
BSU21490	SunI	0,584106979	1,49911076	0,608821357	0,080935539	1,057703703	0,01294202	1,278407232
BSU21500	UvrX	-0,3010065	0,811685926	0,136290277	-0,60029324	0,65961987	0,486824566	0,735652898
BSU21510	YolD	0,24066771	1,181539376	1	0,080935539	1,057703703	1	1,11962154
BSU21520	YolC	0,204160459	1,152015764	0,693959287	0,319320911	1,247743087	0,151133514	1,199879426
BSU21530	YolB	0,127246585	1,09220721	0,457720779	0,09947879	1,071386327	0,631211625	1,081796768
BSU21540	YolA	0,481432458	1,396129201	0,000248086	0,455893111	1,371631661	0,516618288	1,383880431
BSU21550	YokL	0,338880874	1,264775103	0,701727975	0,080935539	1,057703703	0,788079473	1,161239403
BSU21560	YokK	0,462843566	1,378255698	1	-0,77187719	0,585654943	0,294561967	0,981955321
BSU21570	YokJ	0,112348872	1,080986775	0,010417071	0,080935539	1,057703703	1	1,069345239
BSU21580	YokI	0,488852278	1,403328028	0,581338261	0,080935539	1,057703703	0,800457864	1,230515865
BSU21590	YokH	1,111524219	2,160738104	0,003553232	0,996012146	1,994479294	0,003332135	2,077608699
BSU21600	YokG	0,175620948	1,129450425	1	0,080935539	1,057703703	0,833230005	1,093577064
BSU21610	YokF	0,505225811	1,419345495	0,456358623	0,080935539	1,057703703	0,138280806	1,238524599
BSU21620	YokE	0,570678423	1,485221828	0,000297508	0,080935539	1,057703703	0,928354381	1,271462766
BSU21630	YokD	2,119635916	4,345842578	4,96E-07	0,057267071	1,040492864	0,817360294	2,693167721
BSU21640	YokC	0,155737819	1,113991187	0,68145221	0,080935539	1,057703703	1	1,085847445
BSU21650	YokB	0,077555099	1,055228254	1	0,466547338	1,381798583	0,500889071	1,218513419
BSU21660	YokA	0,123507727	1,089380333	1	0,080935539	1,057703703	0,538004896	1,073542018
BSU21680	MsrB	0,290853769	1,223364035	0,034803374	-0,28231313	0,822271579	0,302751299	1,022817807
BSU21690	MsrA	-0,37153293	0,772960755	0,128122528	-0,42407043	0,745318807	0,054081779	0,759139781
BSU21700	YpoP	0,420416013	1,338313413	0,028629808	0,019182191	1,013384868	0,867287436	1,17584914

BSU21710	YpnP	0,767844917	1,702724371	2,83E-05	0,102055014	1,073301214	0,182730539	1,388012792
BSU21720	YpmT	1,062293824	2,088249111	3,59E-07	0,27404535	1,209193685	0,113568169	1,648721398
BSU21730	YpmS	0,567667916	1,48212581	0,000636416	1,270562001	2,412555282	2,23E-07	1,947340546
BSU21740	YpmR	0,236356202	1,178013606	0,179971382	0,962536607	1,948733229	4,35E-06	1,563373417
BSU21750	Sco	0,675574234	1,597232391	0,000166605	0,755365761	1,688059506	0,000158665	1,642645948
BSU21760	YpmP	0,767002187	1,701730039	7,22E-05	0,137317941	1,099858509	0,383494378	1,400794274
BSU21770	IlvA	0,508285028	1,422358393	5,64E-06	0,423089465	1,340795735	0,009233261	1,381577064
BSU21780	YplP	-0,43770226	0,738309557	0,005352569	-0,66595478	0,630271451	0,00880107	0,684290504
BSU21790	YplQ	0,474962081	1,389881689	0,012508845	-0,66652026	0,630024459	0,000421427	1,009953074
BSU21800	YpkP	1,173364581	2,255370703	9,69E-08	0,485442463	1,400015173	0,009839888	1,827692938
BSU21810	DfrA	0,09471041	1,067851038	0,236806978	0,695327652	1,619252141	0,001905138	1,34355159
BSU21820	ThyB	0,17314352	1,127512572	0,120732663	0,818849518	1,763998725	0,000108894	1,445755648
BSU21830	YpjQ	0,912208727	1,881924469	6,55E-06	0,679851773	1,601975155	5,82E-05	1,741949812
BSU21840	YpjP	1,557442563	2,943316248	5,11E-09	0,716733094	1,643456298	0,000646071	2,293386273
BSU21850	YpiP	0,337401083	1,263478472	0,000434438	-0,57781683	0,669976859	0,001794923	0,966727666
BSU21860	YphP	-0,12518455	0,916886751	0,418928566	-0,51333131	0,700602817	0,021808598	0,808744784
BSU21870	IlvD	1,194256065	2,288268057	9,85E-08	0,669189258	1,590179094	3,63E-05	1,939223576
BSU21880	YpgR	0,801157956	1,742499155	0,000127913	0,649742057	1,568887666	0,00142057	1,65569341
BSU21890	YpgQ	0,395442748	1,315346365	0,000860194	0,82736176	1,7744375	2,69E-05	1,544891932
BSU21900	BsaA	0,055319582	1,039089254	0,01028167	0,892785515	1,856757641	0,000271578	1,447923447
BSU21910	MetA	0,145209115	1,105890938	0,055714225	0,477649235	1,392472885	0,006340882	1,249181911
BSU21920	UgtP	0,030744227	1,02153896	0,003450918	0,075787726	1,053936339	0,238943835	1,037737649
BSU21930	CspD	0,610098424	1,526363338	0,004580242	0,748074926	1,679550211	4,66E-07	1,602956774
BSU21940	DegR	-0,29723501	0,813810605	0,374472936	-0,68310345	0,622824046	0,008143589	0,718317325
**BSU21950**	**YpzA**	**-2,55398089**	**0,17028451**	**1,22E-14**	**-1,66199569**	**0,316001719**	**1,05E-10**	**0,243143114**
BSU21960	YpeQ	-1,10171166	0,465963334	1,39E-06	-1,08252283	0,472202363	4,05E-06	0,469082848
**BSU21970**	**YpeP**	**-1,63852055**	**0,321185674**	**3,95E-08**	**-1,1186331**	**0,460529953**	**1,85E-06**	**0,390857813**
BSU21980	YpdP	0,182654958	1,134970624	0,005927307	0,015080237	1,010507645	0,028885669	1,072739135
BSU21990	YpdQ	0,393100894	1,313212961	0,087329943	0,187011534	1,13840313	0,216664782	1,225808046

**BSU22000**	**SspL**	**-1,89876613**	**0,268172623**	**1,11E-10**	**-1,64614628**	**0,319492444**	**2,92E-10**	**0,293832534**
BSU22010	YpcP	0,445553101	1,361836118	0,03247855	-0,28201227	0,822443075	0,028009193	1,092139597
BSU22019	YpzF	-0,79225709	0,577439983	0,000405048	-1,32433687	0,399332703	9,19E-08	0,488386343
BSU22020	YpbS	0,285753561	1,219046841	0,07262332	-0,13547571	0,910369604	0,653561775	1,064708223
BSU22030	DynA	-1,01824943	0,493715065	0,00011945	0,089293446	1,063849039	0,247625484	0,778782052
**BSU22040**	**BpsB**	**-3,09189321**	**0,117286331**	**5,11E-13**	**-1,5137012**	**0,350211605**	**1,09E-07**	**0,233748968**
**BSU22050**	**BpsA**	**-2,07743994**	**0,236934478**	**1,55E-13**	**-1,34235091**	**0,394377484**	**4,53E-08**	**0,315655981**
BSU22060	PbuX	0,298934489	1,23023548	0,08935965	0,787837687	1,726484865	0,001425629	1,478360172
BSU22070	Xpt	-0,720504	0,606885392	0,001251456	1,046117406	2,06496511	1,13E-06	1,335925251
BSU22080	YpwA	-1,02359816	0,491888027	9,76E-05	0,107219179	1,077150008	0,453094216	0,784519018
BSU22090	KdgT	0,162847264	1,119494363	0,012656626	0,968057697	1,956205179	0,000885841	1,537849771
BSU22100	KdgA	0,41578246	1,334022001	0,016213952	1,248251354	2,375533175	3,07E-08	1,854777588
BSU22110	KdgK	-0,5248493	0,695031706	0,032782256	1,111452166	2,160630193	3,34E-07	1,42783095
BSU22120	KdgR	-0,37535333	0,770916583	0,046320059	1,238388174	2,359347909	9,28E-08	1,565132246
BSU22130	KduI	-1,13895806	0,454087408	8,87E-05	0,006319767	1,004390137	0,466470425	0,729238772
BSU22140	KduD	-0,66796075	0,629395711	0,008897307	-0,13606957	0,909994939	0,178089251	0,769695325
BSU22150	YpvA	-0,67056326	0,628261352	0,004133897	0,113225748	1,081644002	0,446726375	0,854952677
BSU22160	YptA	-0,30501939	0,809431338	0,062875569	-0,57107875	0,673113293	0,014624516	0,741272316
**BSU22169**	**YpzG**	**-2,81106017**	**0,142490716**	**5,55E-17**	**-2,60746295**	**0,164087478**	**2,65E-16**	**0,153289097**
BSU22170	YpsC	0,017377529	1,01211802	0,12842011	-0,25395933	0,838591824	0,194049861	0,925354922
BSU22180	GpsB	-0,66770522	0,629507198	0,003749808	-0,38644614	0,765011775	0,173144179	0,697259487
BSU22190	YpsA	-0,93535712	0,522913013	0,000452975	0,194655339	1,144450718	0,021222265	0,833681866
BSU22200	CotD	-0,13654161	0,909697247	0,090438698	-0,3341378	0,793258072	0,196102626	0,85147766
BSU22210	YprB	-1,09036879	0,469641309	3,74E-05	-0,55129212	0,68240867	0,00666114	0,576024989
BSU22220	YprA	-0,04386662	0,970051588	0,035554507	0,840501053	1,79067194	0,000427909	1,380361764
BSU22230	YpqE	0,131347684	1,095316406	0,032867327	-0,5155182	0,699541618	0,04179877	0,897429012
**BSU22240**	**YpqA**	**-2,72623358**	**0,151119991**	**9,00E-14**	**-1,83145053**	**0,28098197**	**4,74E-13**	**0,21605098**
**BSU22250**	**YppG**	**-1,49416661**	**0,354985841**	**4,15E-09**	**-1,73402414**	**0,300612283**	**5,63E-11**	**0,327799062**
BSU22260	YppF	-0,59665714	0,66128444	0,001467285	-0,8916932	0,538981178	0,000209231	0,600133809

BSU22270	YppE	1,501820456	2,831998411	7,09E-10	0,114776075	1,082806967	0,360772301	1,957402689
BSU22280	YppD	1,023543419	2,032905875	6,36E-06	0,506985089	1,421077355	0,012443217	1,726991615
**BSU22290**	**SspM**	**-2,31603619**	**0,200818461**	**5,24E-14**	**-2,83394203**	**0,140248571**	**9,17E-17**	**0,170533516**
**BSU22300**	**YppC**	**-1,49930675**	**0,353723322**	**9,77E-09**	**-1,05886707**	**0,480008857**	**2,42E-06**	**0,41686609**
BSU22310	RecU	-0,74752854	0,595623036	0,001676827	-0,29254998	0,816457686	0,292606843	0,706040361
BSU22320	PonA	-0,95361369	0,516337509	8,39E-06	-0,44804356	0,733036244	0,038912787	0,624686876
BSU22330	YpoC	0,428340813	1,345685067	0,000869263	0,270207084	1,205980921	0,085590736	1,275832994
BSU22340	Nth	0,330067015	1,257071766	0,006609308	0,407067135	1,325987463	0,019508219	1,291529615
BSU22350	DnaD	-0,05999563	0,959267024	0,044599701	0,483657397	1,398283986	0,006240964	1,178775505
BSU22360	AsnS	0,026266453	1,018373267	0,437845337	0,344530198	1,269737436	0,014920907	1,144055351
BSU22370	AspB	0,000929024	1,000644158	0,023834788	-0,21477914	0,861678064	0,478729319	0,931161111
BSU22380	YpmB	-0,64794761	0,63818756	0,001411354	-0,16802327	0,890061376	0,446891019	0,764124468
BSU22390	YpmA	-0,57428848	0,671617405	0,028804493	0,27400494	1,209159816	0,182676595	0,940388611
BSU22400	DinG	-0,6575345	0,633960784	0,003977113	0,293066776	1,225242042	0,041501109	0,929601413
BSU22410	PanD	-0,51460873	0,69998275	0,055857668	-0,06605555	0,955246153	0,917984598	0,827614452
BSU22420	PanC	-0,27640762	0,825644357	8,13E-05	0,252188619	1,191012553	0,106168362	1,008328455
BSU22430	PanB	-0,51053214	0,701963468	0,020845042	0,283384538	1,217046709	0,137115399	0,959505089
BSU22440	BirA	-1,2317271	0,425807393	6,87E-07	0,579470734	1,49430095	0,002420799	0,960054171
BSU22450	Cca	-1,25216227	0,419818523	2,04E-07	0,276273366	1,211062538	0,138159703	0,81544053
BSU22460	BshA	-1,07163088	0,475780853	1,25E-07	0,415802292	1,334040339	0,006141566	0,904910596
BSU22470	BshB1	-1,17161802	0,443923189	2,49E-05	0,546535862	1,460574413	0,003992646	0,952248801
BSU22480	MgsA	-0,46323281	0,725359044	0,006129365	0,586901709	1,502017591	0,007275866	1,113688317
BSU22490	DapB	-1,27241279	0,413966869	4,48E-06	0,323476463	1,251342279	0,109616675	0,832654574
BSU22500	YpjD	-1,15305705	0,449671374	5,41E-06	0,099377043	1,07131077	0,324840947	0,760491072
BSU22510	YpjC	0,148758493	1,108615046	0,32030073	-0,04110405	0,97191089	0,410396947	1,040262968
BSU22520	YpjB	0,774985592	1,711172962	0,000215068	0,407252612	1,326157947	0,001150551	1,518665454
BSU22530	YpjA	-0,98052212	0,506796294	0,002389032	0,663778079	1,584225911	0,008898276	1,045511103
BSU22540	QcrC	0,156553688	1,114621346	0,125560167	0,44583248	1,362099864	0,000420355	1,238360605
BSU22550	QcrB	0,265821879	1,202320796	0,176722003	0,325281434	1,252908826	0,001841878	1,227614811

BSU22560	QcrA	0,270622677	1,206328375	0,299813766	0,309143552	1,238971973	0,00922487	1,222650174
BSU22570	YpiF	0,783891613	1,721769018	4,35E-06	0,602910529	1,518777498	0,001217568	1,620273258
BSU22580	YpiB	-0,00213177	0,998523459	0,604515039	0,216101776	1,161590674	0,065670536	1,080057067
BSU22590	YpiA	-0,34345238	0,788152999	0,03298147	0,02261321	1,015797769	0,259152034	0,901975384
BSU22600	AroE	0,257513152	1,195416327	0,01372536	0,891153011	1,854657786	3,66E-06	1,525037056
BSU22610	TyrA	-0,24297525	0,845000878	0,365132869	1,277510692	2,424203299	3,84E-08	1,634602089
BSU22620	HisC	0,482234394	1,396905469	0,096423191	1,106497393	2,15322248	2,07E-05	1,775063975
BSU22630	TrpA	0,278615116	1,213029904	0,098046992	0,609977433	1,526235335	0,000334867	1,369632619
BSU22640	TrpB	-0,49117882	0,711443545	0,003663479	0,804527625	1,746573825	2,89E-05	1,229008685
BSU22650	TrpF	-0,61681068	0,652110935	0,002793161	0,663325097	1,583728569	0,000828114	1,117919752
BSU22660	TrpC	-0,83216512	0,56168566	6,48E-05	0,837709563	1,787210496	6,23E-05	1,174448078
BSU22670	TrpD	-0,78010066	0,582326162	0,001543928	0,57599719	1,490707483	0,001065708	1,036516822
BSU22680	TrpE	-0,04076318	0,972140556	0,532906701	0,731897957	1,660822573	0,000169184	1,316481564
BSU22690	AroH	-0,67554563	0,626095383	0,007128705	0,920551475	1,892838699	8,00E-06	1,259467041
BSU22700	AroB	0,045050004	1,031718938	0,083299625	0,906359052	1,874309307	5,04E-05	1,453014122
BSU22710	AroF	-0,64272754	0,640500882	0,007866462	1,030529236	2,042773482	7,05E-07	1,341637182
BSU22720	CheR	-0,40163499	0,756999898	0,013225953	1,236173706	2,355729201	6,87E-06	1,55636455
BSU22730	Ndk	0,894243682	1,858635259	7,19E-06	0,001009072	1,00069968	0,300490281	1,42966747
BSU22740	HepT	-0,15399103	0,898760717	0,727568613	0,621162082	1,538113623	0,001658054	1,21843717
BSU22750	MenH	0,482708097	1,397364212	0,007183123	0,565942371	1,480354164	0,004601809	1,438859188
BSU22760	HepS	-0,07249611	0,950991197	0,86601614	0,433589395	1,350589639	0,016840886	1,150790418
BSU22770	MtrB	0,183942307	1,135983836	0,173572176	0,292039143	1,224369611	0,121384966	1,180176724
BSU22780	FolE	0,559524598	1,473783491	0,003187655	0,28019999	1,214363211	0,047692659	1,344073351
BSU22790	Hbs	0,725340503	1,653290798	3,02E-05	0,015678946	1,010927086	0,664578788	1,332108942
BSU22800	SpoIVA	0,405251041	1,324319333	7,43E-05	-0,47309255	0,720418656	0,027885855	1,022368994
BSU22810	YphF	0,432913285	1,349956842	0,000142628	0,096348672	1,069064332	0,35906485	1,209510587
BSU22820	YphE	0,5984442	1,514082901	0,00554229	0,017666977	1,012321102	0,481529604	1,263202002
BSU22830	GpsA	-0,67010415	0,628461316	0,000388077	-0,11365851	0,924241319	0,604983817	0,776351318
BSU22840	YphC	-0,98545873	0,505065103	0,000286073	0,194036781	1,143960137	0,242888903	0,82451262

BSU22849	YpzH	-1,52483636	0,34751897	2,63E-09	-0,60698603	0,656566923	0,017466321	0,502042946
BSU22850	SeaA	-1,70461577	0,306802943	4,10E-10	-0,3844747	0,766057879	0,094289217	0,536430411
BSU22860	YphA	-0,7559516	0,592155673	0,000119475	-0,41465429	0,75019924	0,043245135	0,671177456
**BSU22869**	**YpzI**	**-2,11139442**	**0,231423228**	**3,43E-13**	**-2,73905292**	**0,149783133**	**7,31E-17**	**0,190603181**
BSU22870	Fni	1,117888061	2,170290344	4,36E-07	-0,23645127	0,848830692	0,25621364	1,509560518
BSU22880	YpfD	0,598858615	1,514517885	0,000653908	0,321736093	1,249833654	0,004348644	1,38217577
BSU22890	Cmk	-0,87620527	0,544798535	9,41E-05	-0,28266667	0,822070102	0,201826655	0,683434318
BSU22900	YpfB	-1,66285094	0,315814443	1,14E-09	-1,0223938	0,492298823	1,93E-05	0,404056633
BSU22910	YpfA	-1,39292264	0,380792604	2,44E-08	-1,00414998	0,498563792	9,45E-05	0,439678198
**BSU22920**	**YpeB**	**-2,94295327**	**0,130041746**	**8,17E-13**	**-2,0653501**	**0,238928342**	**2,35E-13**	**0,184485044**
**BSU22930**	**SleB**	**-2,95844383**	**0,128652925**	**5,10E-18**	**-2,08874049**	**0,235085833**	**7,62E-13**	**0,181869379**
BSU22940	PrsW	1,692458553	3,232070244	6,64E-09	-0,86190232	0,550226556	5,65E-05	1,8911484
BSU22950	YpdA	-0,084779	0,94292897	0,072158508	-0,19927685	0,870987037	0,22718928	0,906958004
BSU22960	GudB	0,770998465	1,706450381	0,000285507	0,459060888	1,374646711	0,021120679	1,540548546
BSU22970	YpbH	0,358039629	1,28168313	0,001891928	0,1872306	1,138576004	0,09263172	1,210129567
BSU22980	YpbG	-0,56794039	0,674579139	0,031649576	0,328484352	1,255693491	0,111764192	0,965136315
BSU22990	YpbF	0,77409573	1,710117827	6,32E-06	0,017817218	1,01242653	0,460298182	1,361272178
BSU23000	YpbE	-0,50477343	0,70477105	0,022718079	-0,30928776	0,807040089	0,25045042	0,755905569
BSU23010	YpbD	-0,78163805	0,581705944	0,005041703	0,612150896	1,528536386	0,00431778	1,055121165
BSU23020	RecQ	-0,48137948	0,71629239	0,019147568	0,345446494	1,270544137	0,028480563	0,993418264
BSU23030	YpbB	0,076381703	1,054370348	0,212316422	1,137244392	2,199604879	3,65E-07	1,626987614
BSU23040	Fer	0,101381732	1,072800439	0,005176449	-0,53083361	0,692154681	0,05207434	0,88247756
BSU23050	RibU	-0,29719968	0,813830538	0,059562141	-1,25995681	0,417556459	1,79E-07	0,615693499
BSU23060	YpzE	0,504579252	1,418709543	0,00832413	-0,99322405	0,502353889	5,29E-05	0,960531716
BSU23070	SerA	-0,37263702	0,772369436	0,084864815	0,296492094	1,228154528	0,016761633	1,000261982
BSU23080	AroC	0,087829255	1,062769887	0,18236289	0,639570576	1,557865385	0,001339989	1,310317636
BSU23090	RsiX	-0,02840212	0,980505673	0,011969765	-0,40977335	0,752741623	0,059320395	0,866623648
BSU23100	SigX	-0,35624158	0,781197059	0,017743283	-0,15457686	0,898395831	0,283944886	0,839796445
BSU23110	ResE	0,720042553	1,647230619	0,000129649	0,410040019	1,328722671	0,012911182	1,487976645

BSU23120	ResD	0,354252558	1,278323126	0,05228958	0,667811347	1,588661046	0,00087474	1,433492086
BSU23130	ResC	0,087795576	1,062745077	0,638153271	0,393767948	1,313820287	0,008724706	1,188282682
BSU23140	ResB	-0,04890083	0,966672543	0,033469042	0,282266441	1,216103856	0,030253843	1,091388199
BSU23150	ResA	0,088476371	1,063246695	0,136567385	0,020033841	1,013983264	0,410827949	1,03861498
BSU23160	RluB	1,035333844	2,049587872	1,85E-05	0,562392467	1,476716073	0,005183243	1,763151972
BSU23170	SpmB	1,653284729	3,145489898	1,13E-10	0,431209228	1,348363265	0,008361515	2,246926582
BSU23180	SpmA	1,296892671	2,456991168	8,61E-08	0,37519844	1,297017945	0,027145855	1,877004556
BSU23190	DacB	1,01568734	2,021865936	7,17E-07	0,458963355	1,374553781	0,009549473	1,698209859
BSU23200	YpuI	-0,30981346	0,806746066	0,027095159	-1,05210031	0,482265561	1,19E-05	0,644505814
BSU23210	ScpB	-0,16484189	0,892026274	0,587544408	0,479564338	1,394322548	0,020980443	1,143174411
BSU23220	ScpA	-0,0940894	0,936863388	0,210056781	0,395261969	1,315181553	0,018127646	1,12602247
BSU23230	YpuF	-0,95560096	0,515626759	0,000256746	-0,64168077	0,640965775	0,004357582	0,578296267
BSU23240	RibT	1,589591444	3,009641076	6,29E-10	-0,17636991	0,884926841	0,531008325	1,947283958
BSU23250	RibH	0,02051562	1,014321934	0,030330035	0,214124118	1,159999447	0,275698669	1,087160691
BSU23260	RibA	0,568699152	1,48318561	1,50E-05	0,002301111	1,001596281	0,338484369	1,242390946
BSU23270	RibE	-0,41527326	0,74987745	0,08257453	0,063668363	1,045119828	0,326681212	0,897498639
BSU23280	RibD	0,556235392	1,470427234	0,009046913	-0,05637779	0,961675593	0,778009585	1,216051414
BSU23300	YpuD	0,020505868	1,014315078	0,257195214	-0,56550971	0,675716642	0,015370172	0,84501586
BSU23310	SipS	1,233296588	2,351035931	1,66E-07	0,222321886	1,166609628	0,093049853	1,75882278
**BSU23320**	**YpzC**	**-1,59461954**	**0,331109534**	**4,53E-07**	**-1,36790352**	**0,387453875**	**2,02E-07**	**0,359281704**
BSU23328	YpzJ	0,124527562	1,090150684	0,035710251	0,327836228	1,255129503	0,047463382	1,172640093
BSU23340	YpuB	-0,10973178	0,926760345	0,036889042	-0,63206033	0,645254264	0,001726321	0,786007304
BSU23350	YpzD	-0,90954357	0,532353487	0,041894819	-0,27969645	0,823764322	0,093475464	0,678058904
BSU23360	PpiB	0,554544794	1,468705147	0,002731814	0,112435824	1,081051928	0,529649193	1,274878538
BSU23370	YpuA	0,607170519	1,523268771	0,003238813	0,639507593	1,557797376	0,003624849	1,540533074
BSU23380	LysA	-0,60136986	0,659127806	0,024088289	-0,74111428	0,59827709	0,001776139	0,628702448
**BSU23390**	**SpoVAF**	**-3,40032061**	**0,094711235**	**2,37E-18**	**-1,87741069**	**0,272171764**	**4,25E-11**	**0,183441499**
**BSU23401**	**SpoVAEA**	**-3,14290548**	**0,113211665**	**1,00E-18**	**-1,73880374**	**0,299618013**	**8,92E-11**	**0,206414839**
**BSU23402**	**SpoVAEB**	**-3,70514651**	**0,076672524**	**4,42E-20**	**-1,78574347**	**0,290026479**	**4,44E-11**	**0,183349502**

**BSU23410**	**SpoVAD**	**-3,55163048**	**0,08528108**	**1,45E-19**	**-1,75416876**	**0,296443947**	**1,19E-10**	**0,190862513**
**BSU23420**	**SpoVAC**	**-3,43060272**	**0,092743969**	**4,55E-18**	**-1,79613071**	**0,287945822**	**1,95E-11**	**0,190344895**
**BSU23430**	**SpoVAB**	**-2,99374914**	**0,125542771**	**7,82E-18**	**-1,96710255**	**0,255766187**	**2,75E-12**	**0,190654479**
**BSU23440**	**SpoVAA**	**-3,03121034**	**0,12232487**	**5,00E-17**	**-1,61353949**	**0,326795611**	**1,43E-09**	**0,224560241**
BSU23450	SigF	0,913241935	1,883272721	2,95E-05	0,22664982	1,170114589	0,055226926	1,526693655
BSU23460	SpoIIAB	0,848184845	1,8002345	0,00027724	0,428881977	1,346189936	0,002947528	1,573212218
BSU23470	SpoIIAA	0,343702543	1,269009213	0,008494821	0,352556792	1,276821449	0,009331836	1,272915331
BSU23480	DacF	-1,17036776	0,444308067	1,80E-05	-0,67790689	0,62507149	0,010021069	0,534689778
BSU23490	PupG	0,058461488	1,041354652	0,135885792	0,16690337	1,122646226	0,228997165	1,082000439
BSU23500	Drm	-0,24992658	0,840939212	0,233282776	0,544163142	1,458174262	0,007464213	1,149556737
BSU23510	RipX	0,518171872	1,432139346	0,007050168	0,312659758	1,241995338	0,050849065	1,337067342
BSU23519	YqzK	0,232172908	1,17460274	0,061849131	0,036448893	1,025586298	0,566752638	1,100094519
BSU23520	Fur	0,507014097	1,421105929	9,78E-05	0,220717047	1,165312625	0,095543236	1,293209277
BSU23530	SpoIIM	1,693829122	3,235142189	8,80E-12	0,38659489	1,307304205	0,072468139	2,271223197
BSU23540	YqkK	0,06213557	1,044010028	0,821134272	0,080935539	1,057703703	0,61797602	1,050856866
BSU23550	MleA	0,467871045	1,383066998	0,003193108	0,803975198	1,745905167	0,000553554	1,564486083
BSU23560	MleN	-0,1464169	0,903491606	0,208446567	0,824228613	1,770588078	0,000305695	1,337039842
BSU23570	AnsB	-1,55740815	0,339760925	3,59E-09	-0,62940916	0,646441102	0,008842529	0,493101014
BSU23580	AnsA	-2,37983908	0,192130827	6,81E-15	-0,96012153	0,514013613	3,15E-05	0,35307222
BSU23590	AnsR	0,33964245	1,265442935	0,115918833	-1,46082492	0,363285348	4,14E-09	0,814364141
BSU23600	YqxK	-1,3645495	0,388355687	1,77E-07	-0,9589332	0,514437173	6,97E-06	0,45139643
BSU23610	NudF	-0,65151914	0,636609619	0,002524203	-0,12945479	0,914176861	0,415853319	0,77539324
BSU23620	YqkF	-1,27694515	0,412668394	3,38E-08	-0,96499636	0,512279705	4,17E-05	0,46247405
BSU23630	YqkE	-0,6556672	0,634781859	0,00356499	-1,09449074	0,468301403	1,78E-05	0,551541631
BSU23640	YqkD	-0,16963663	0,88906658	0,56552782	-0,75341346	0,593198372	0,000449882	0,741132476
BSU23650	YqkC	0,085981438	1,061409553	0,177404742	-0,36277254	0,777668636	0,165315288	0,919539094
BSU23660	YqkB	-0,07593557	0,948726687	0,188278056	-0,15095543	0,900653807	0,566934686	0,924690247
BSU23670	YqkA	-0,34966081	0,784768582	0,033663269	0,715606211	1,642173103	0,000197774	1,213470842
BSU23680	YqjZ	-0,15571724	0,897685978	0,028515759	0,993635486	1,991196344	7,16E-06	1,444441161

BSU23690	YqjY	-0,42078037	0,747020442	0,014612218	0,5726892	1,48729332	0,002145437	1,117156881
BSU23700	YqjX	0,389656569	1,310081504	0,005777603	-0,12167033	0,919122889	0,499174186	1,114602197
BSU23710	PolY2	-1,98624325	0,252395266	1,64E-10	-0,04877075	0,966759707	0,466760393	0,609577486
BSU23720	YqzH	0,42901162	1,346310913	0,067671767	0,905201988	1,872806684	0,075891645	1,609558798
BSU23730	YqjV	-1,71075093	0,305501013	1,72E-07	-0,49684788	0,708653415	0,040664764	0,507077214
BSU23740	YqjU	-1,27051409	0,41451204	2,67E-08	-0,70257919	0,614472697	0,002734991	0,514492369
BSU23750	YqjT	0,731532725	1,660402172	0,000177519	0,31771926	1,246358636	0,09961975	1,453380404
BSU23760	CoaA	0,111884762	1,080639081	0,007981084	0,31866453	1,247175533	0,103082355	1,163907307
BSU23770	DsdA	-0,52173178	0,696535226	0,005871977	0,320033413	1,248359461	0,105565336	0,972447343
BSU23780	YqjQ	-0,72758225	0,603915143	0,008721657	0,075367179	1,05362916	0,291474919	0,828772152
BSU23790	YqjP	-0,63033326	0,646027166	0,001821065	0,59689067	1,512453376	0,000837633	1,079240271
BSU23800	ProI	-1,69706062	0,308413833	7,73E-10	-0,69018138	0,619775925	0,002236396	0,464094879
BSU23810	YqjN	0,339426024	1,265253113	0,015369559	1,515086893	2,85816042	1,20E-08	2,061706766
BSU23820	YqjM	-1,79287341	0,288596676	8,61E-09	-0,62344827	0,649117578	0,010590976	0,468857127
BSU23830	YqjL	0,988422918	1,984014979	8,25E-06	1,326948669	2,508715148	5,86E-08	2,246365063
BSU23840	Rnz	0,028291871	1,019803978	0,261332456	-0,08574429	0,942298276	0,469414912	0,981051127
BSU23850	Zwf	-0,49269017	0,710698632	0,030721192	-0,16756058	0,890346879	0,65353473	0,800522756
BSU23860	GndA	0,53777538	1,451732236	0,000931446	0,620616263	1,537531814	0,00367494	1,494632025
BSU23870	PolY1	0,188862647	1,139864746	0,084493097	0,419930907	1,337863481	0,100692673	1,238864113
BSU23880	MifM	0,229313496	1,172276991	0,147908004	0,316481357	1,245289658	0,144797361	1,208783325
BSU23890	YqjG	1,316857518	2,491228782	1,29E-09	-0,18218401	0,881367736	0,528957744	1,686298259
BSU23900	YqjF	-0,68649415	0,62136197	0,072930742	-0,80987978	0,570429391	0,001498041	0,595895681
BSU23910	YqjE	-0,45186985	0,731094675	0,007536764	-0,69166781	0,619137688	0,004884592	0,675116181
BSU23920	YqjD	-0,97431892	0,508980074	7,05E-05	-0,24514967	0,843728262	0,575774348	0,676354168
BSU23930	YqjC	-1,59443993	0,33115076	1,94E-10	-0,87953717	0,543541778	0,00034911	0,437346269
BSU23940	YqjB	-1,57338495	0,336019078	1,23E-08	-0,6190363	0,651105712	0,000805472	0,493562395
BSU23950	YqjA	0,265044351	1,201672991	0,113160187	-0,59166533	0,663576485	0,012236763	0,932624738
BSU23960	ArtR	-0,33488749	0,792845964	0,177770995	-0,47260388	0,720662722	0,029062164	0,756754343
BSU23970	ArtQ	0,179500996	1,132492107	0,1662034	-0,49025251	0,711900487	0,091909794	0,922196297

BSU23980	ArtP	0,584600416	1,49962358	0,005296765	-0,61415344	0,653313138	0,005392515	1,076468359
BSU23990	YqiW	-0,05140925	0,964993244	0,571406592	-0,52072152	0,697023149	0,024011778	0,831008197
BSU24000	BmrU	-0,12014485	0,920095264	0,17628593	0,132288743	1,096031106	0,35904444	1,008063185
BSU24010	Bmr	-0,16979535	0,888968774	0,153358299	-0,39412765	0,760949357	0,140926102	0,824959066
BSU24020	BmrR	-0,72889372	0,603366406	4,52E-05	-0,39603356	0,759944752	0,083166322	0,681655579
BSU24030	BkdB	-0,66110007	0,632395905	0,017403763	-0,03640768	0,975079882	0,858532255	0,803737894
BSU24040	BkdAB	0,151792773	1,110949142	0,24502539	0,186306074	1,137846601	0,325646627	1,124397871
BSU24050	BkdAA	-0,47892689	0,717511125	0,006819016	0,238010197	1,179364928	0,182863839	0,948438027
BSU24060	LpdV	-0,82560395	0,564245944	0,001185468	0,467787267	1,382986686	0,043723913	0,973616315
BSU24070	Buk	-0,34167827	0,789122802	0,085850903	0,346626975	1,271584182	0,062825173	1,030353492
BSU24080	Bcd	0,024804036	1,017341495	0,232319238	0,23579919	1,177558872	0,181668722	1,097450184
BSU24090	Ptb	-0,51356204	0,700490779	0,071691299	0,389582681	1,31001441	0,069280169	1,005252595
BSU24100	BkdR	-0,12968639	0,914030121	0,123801371	0,598388667	1,514024621	0,010685018	1,214027371
BSU24110	YqzF	1,262269146	2,398727287	3,18E-08	-0,72288027	0,60588661	0,002106314	1,502306949
BSU24120	YqiQ	0,093767929	1,067153662	0,009637315	0,070675493	1,050208293	0,233229721	1,058680978
BSU24130	MmgE	-0,20977404	0,864672647	0,172254289	0,323202701	1,25110485	0,004883326	1,057888748
BSU24140	MmgD	0,075702485	1,053874069	0,198040836	0,661143655	1,581335685	3,94E-06	1,317604877
BSU24150	MmgC	-0,42307006	0,745835792	0,007596129	0,689363533	1,612571951	7,38E-06	1,179203871
BSU24160	MmgB	-0,19661261	0,872596982	0,14890738	0,679002294	1,601032167	4,35E-06	1,236814574
BSU24170	MmgA	0,00727766	1,005057235	0,002734245	0,280761644	1,214836065	0,01208338	1,10994665
BSU24180	YqiK	-0,47917042	0,717390018	0,123679983	-0,12058654	0,91981362	0,246242583	0,818601819
BSU24190	YqiI	-0,72601972	0,604569574	0,178600985	0,016668162	1,011620489	0,555398678	0,808095031
BSU24200	YqiH	-2,31964373	0,200316931	0,000149457	-0,02320713	0,984042731	0,512847297	0,592179831
**BSU24210**	**YqiG**	**-2,24402515**	**0,211096542**	**3,85E-12**	**-1,41931192**	**0,373890594**	**5,83E-08**	**0,292493568**
BSU24220	Spo0A	0,564213528	1,478581255	0,001104089	0,385888488	1,306664253	0,054689409	1,392622754
**BSU24230**	**SpoIVB**	**-2,30009946**	**0,2030491**	**2,00E-14**	**-2,56234841**	**0,169299731**	**3,32E-15**	**0,186174416**
BSU24240	RecN	0,109817634	1,079091823	0,286240633	0,122451475	1,088583048	0,506370284	1,083837436
BSU24250	AhrC	-0,48458825	0,714701018	0,026353486	0,57981784	1,494660515	0,004474735	1,104680766
BSU24260	YqxC	0,832081601	1,780252158	5,45E-05	0,291018561	1,223503782	0,029304314	1,50187797

BSU24270	Dxs	0,118480195	1,085590646	0,141117193	0,682718749	1,605161823	0,000845828	1,345376235
BSU24280	YqiD	0,215614649	1,161198528	0,004227312	0,713818532	1,640139505	0,004510588	1,400669017
BSU24290	YqiC	-0,30475145	0,809581683	0,505889941	0,386249341	1,306991122	0,052522146	1,058286402
BSU24300	YqiB	-0,96256973	0,51314209	0,000161874	0,163327169	1,119866819	0,382126522	0,816504454
BSU24310	FolD	-0,00990914	0,993155044	0,473302575	0,242721117	1,183222274	0,112756775	1,088188659
BSU24320	NusB	0,10291659	1,073942379	0,102810331	0,238039611	1,179388973	0,079900775	1,126665676
BSU24330	YqhY	0,191229378	1,141736221	0,262276931	-0,11813296	0,921379268	0,705260736	1,031557744
BSU24340	AccC	0,271138017	1,20675936	0,34541865	0,235031556	1,17693248	0,034673987	1,19184592
BSU24350	AccB	0,132106852	1,09589293	0,002772862	0,567694589	1,482153213	0,001798901	1,289023071
BSU24360	SpoIIIAH	1,410882227	2,658997144	2,58E-08	0,063560068	1,045041379	0,413097922	1,852019261
BSU24370	SpoIIIAG	1,088097165	2,125934525	1,84E-06	-0,08721198	0,941340137	0,640791884	1,533637331
BSU24380	SpoIIIAF	0,968047025	1,956190708	0,000330118	0,183259855	1,135446598	0,021937013	1,545818653
BSU24390	SpoIIIAE	0,677643969	1,599525474	0,002679993	0,681258389	1,603537829	0,000174048	1,601531651
BSU24400	SpoIIIAD	0,303802127	1,234393292	0,043588019	1,004351464	2,006041517	8,40E-07	1,620217405
BSU24410	SpoIIIAC	0,664862259	1,585416898	0,002557744	0,715979628	1,642598207	0,000207922	1,614007553
BSU24420	SpoIIIAB	0,544965413	1,458985366	0,00021047	0,808187836	1,751010622	5,60E-05	1,604997994
BSU24430	SpoIIIAA	0,684976144	1,607675398	0,000186674	0,970240768	1,959167529	6,75E-06	1,783421463
BSU24440	YqhV	0,724756017	1,652621128	0,000189165	0,898750958	1,864451098	9,94E-06	1,758536113
BSU24450	Efp	-0,56539173	0,675771903	0,007461465	0,049734617	1,035074505	0,580574525	0,855423204
BSU24460	YqhT	-0,37911118	0,768911156	0,027634345	0,244398033	1,184598392	0,047313377	0,976754774
BSU24470	YqhS	-0,73470528	0,600940774	0,005449732	0,188765429	1,139787937	0,230215679	0,870364355
BSU24480	YqhR	-1,56040002	0,339057057	1,07E-07	-0,94164516	0,520638839	7,05E-05	0,429847948
BSU24490	YqhQ	-0,34982345	0,78468012	0,131615533	-0,27408084	0,826977027	0,263869807	0,805828574
BSU24500	YqhP	0,556024067	1,470211863	0,001203285	-0,86750435	0,548094154	0,000226029	1,009153008
**BSU24510**	**YqhO**	**-1,42850826**	**0,371514839**	**7,40E-08**	**-1,29216592**	**0,40833753**	**7,39E-07**	**0,389926185**
BSU24520	MntR	0,034778656	1,024399643	0,029579688	-0,42960745	0,742463779	0,117286924	0,883431711
BSU24530	LipM	1,108406146	2,15607318	3,20E-09	0,760256752	1,693792037	0,00116072	1,924932608
BSU24540	YqhL	1,639768379	3,116157988	1,59E-09	0,24716129	1,18686948	0,044093079	2,151513734
BSU24550	GcvPB	2,090157197	4,257944652	1,09E-15	0,300135009	1,231259631	0,060178695	2,744602141

BSU24560	GcvPA	1,95582077	3,879365666	2,30E-12	0,760105037	1,693613925	9,94E-05	2,786489796
BSU24570	GcvT	1,477269561	2,784212953	1,24E-09	0,752886534	1,685161118	0,000184291	2,234687035
BSU24580	YqhH	-0,43155636	0,741461475	0,068410408	-0,44406602	0,735060031	0,084911479	0,738260753
BSU24590	YqhG	-0,79977016	0,574440688	0,000925951	-0,35911886	0,779640605	0,100525809	0,677040647
BSU24600	SinI	-0,29964438	0,812452641	0,276302565	-0,76149434	0,589885011	0,000810164	0,701168826
BSU24610	SinR	0,253737336	1,192291778	0,124828602	-0,90120666	0,535438709	6,99E-05	0,863865243
BSU24620	TasA	-3,00819128	0,124292291	2,98E-18	-0,53825357	0,688603985	0,022054849	0,406448138
BSU24630	SipW	-3,1434695	0,113167414	6,98E-18	-0,09618684	0,935502335	0,61356897	0,524334874
BSU24640	TapA	-2,96986568	0,127638399	1,69E-16	0,121829928	1,088114162	0,537585764	0,60787628
BSU24650	YqzG	-0,36470029	0,776630198	0,084271088	-1,78870532	0,289431666	1,00E-11	0,533030932
BSU24660	YqzE	0,314433645	1,243523391	0,017268651	-0,48846209	0,712784519	0,000104584	0,978153955
BSU24670	ComGG	-0,64828437	0,638038608	0,006160182	-0,43409141	0,740159752	0,023203472	0,68909918
BSU24680	ComGF	-1,48227462	0,357924048	5,84E-08	-0,2333969	0,85062968	0,203329793	0,604276864
BSU24690	ComGE	-0,9325441	0,523933603	1,65E-05	-0,10534463	0,929582856	0,365112742	0,72675823
BSU24700	ComGD	-1,81345975	0,284507828	4,22E-11	-0,01165806	0,991951811	0,42514517	0,638229819
BSU24710	ComGC	-1,50306253	0,352803668	2,57E-09	0,045837707	1,032282404	0,471614345	0,692543036
BSU24720	ComGB	-1,61506104	0,326451135	6,71E-08	-0,04516093	0,969181704	0,286363897	0,647816419
BSU24730	ComGA	-1,22851627	0,426756114	1,71E-07	-0,24497075	0,843832907	0,266279955	0,635294511
BSU24740	CorA	1,00153646	2,00213112	0,000313167	0,319891711	1,248236853	0,04726236	1,625183986
**BSU24750**	**YqhB**	**1,345792827**	**2,541698361**	**5,34E-05**	**1,55709032**	**2,942597708**	**5,98E-07**	**2,742148034**
**BSU24760**	**RsbRD**	**1,942704622**	**3,844256557**	**5,67E-11**	**1,38958199**	**2,620027564**	**6,38E-06**	**3,23214206**
**BSU24770**	**MgsR**	**1,610402582**	**3,053370338**	**3,66E-12**	**1,031211349**	**2,043739543**	**2,57E-06**	**2,54855494**
BSU24780	YqgY	2,311076498	4,962532318	6,81E-12	-0,51369916	0,700424201	0,104544872	2,831478259
BSU24790	YqgX	0,656840298	1,57662581	1,46E-06	0,009267409	1,006444355	0,122775234	1,291535083
BSU24800	YqgW	2,385520071	5,225322463	1,31E-15	-1,21587319	0,430512434	6,13E-07	2,827917449
BSU24810	YqgV	-1,27797041	0,412375233	2,93E-07	-0,79027524	0,578233765	0,000332449	0,495304499
BSU24820	YqgU	-1,66874268	0,314527337	2,19E-09	-0,5952688	0,661921116	0,007866342	0,488224227
BSU24830	YqgT	-1,28787164	0,409554788	3,67E-08	-0,39971943	0,758005681	0,055820009	0,583780235
BSU24840	YqgS	-0,8062155	0,571880057	0,000503044	0,138670901	1,100890441	0,168868985	0,836385249

BSU24850	GlcK	-0,46054888	0,726709724	0,07348498	0,567719398	1,4821787	0,009223475	1,104444212
BSU24860	YqgQ	-0,0116428	0,991962306	0,288791783	0,724278559	1,652074286	0,001071453	1,322018296
BSU24870	YqgP	0,523218161	1,437157484	0,00646667	0,58574818	1,50081711	0,007710452	1,468987297
**BSU24880**	**YqgO**	**-1,93198267**	**0,262068767**	**3,28E-13**	**-1,97928759**	**0,253615076**	**8,86E-13**	**0,257841921**
BSU24890	YqgN	0,054548071	1,038533728	0,050823585	-0,08866718	0,940391118	0,633941846	0,989462423
BSU24900	RpmGA	1,007383283	2,01026164	3,91E-06	-0,58919955	0,664711606	0,01035465	1,337486623
BSU24910	YqgM	0,199180764	1,14804625	0,056184818	0,242776797	1,183267941	0,197755809	1,165657096
BSU24920	YqgL	-0,94172492	0,520610054	0,002200048	0,65063813	1,56986242	0,009937068	1,045236237
BSU24930	YqzD	-0,32826687	0,796492749	0,063286289	0,295428059	1,227249057	0,14038811	1,011870903
BSU24940	YqzC	-1,43276345	0,370420681	0,038311708	0,231867603	1,174354195	0,250136982	0,772387438
BSU24950	PstBB	-1,49518496	0,354735358	3,68E-06	0,02968808	1,0207914	0,352873818	0,687763379
BSU24960	PstBA	-0,98430606	0,505468798	0,000255576	0,335863006	1,262132178	0,00487729	0,883800488
BSU24970	PstA	-2,09955914	0,233329538	3,96E-12	0,567072067	1,481513803	0,002852423	0,85742167
BSU24980	PstC	-2,64375503	0,160011219	7,22E-11	0,793209202	1,732924982	0,000152512	0,946468101
BSU24990	PstS	-1,62473613	0,324269192	1,35E-07	0,232378062	1,174769783	0,040078726	0,749519487
BSU25000	PbpA	-1,31475787	0,401992957	4,15E-06	0,091961927	1,065818609	0,436216049	0,733905783
**BSU25010**	**YqgE**	**-3,38823163**	**0,095508197**	**1,17E-18**	**-1,48787184**	**0,356538102**	**2,38E-09**	**0,226023149**
BSU25020	SodA	0,360245897	1,283644667	0,067963764	-0,48556371	0,714217948	0,105058529	0,998931307
BSU25030	YqgC	-0,05282951	0,964043725	0,472343218	-0,12642891	0,916096254	0,668814908	0,94006999
BSU25040	YqgB	0,27077024	1,206451768	0,099253477	-0,22150747	0,857668792	0,272176827	1,03206028
BSU25050	YqgA	1,417946284	2,672048665	5,56E-10	0,345162521	1,270294074	0,047772376	1,97117137
BSU25060	YqfZ	0,208356154	1,155370973	0,026736461	0,239888577	1,180901454	0,124555921	1,168136213
BSU25070	IspG	0,97988444	1,972307421	2,94E-05	-0,39983216	0,757946457	0,151337827	1,365126939
**BSU25080**	**YqfX**	**-3,10319217**	**0,116371351**	**6,46E-19**	**-3,22019109**	**0,107306466**	**2,63E-18**	**0,111838908**
**BSU25090**	**YqfW**	**-1,69751207**	**0,308317339**	**3,91E-11**	**-1,00385552**	**0,498665563**	**1,33E-05**	**0,403491451**
BSU25100	Zur	0,755511664	1,688230231	8,87E-05	0,139186953	1,1012843	0,323995408	1,394757266
BSU25110	YqfU	-0,04355292	0,970262544	0,075600968	0,010201203	1,007095993	0,469909645	0,988679269
**BSU25120**	**YqfT**	**-2,3183245**	**0,200500188**	**3,24E-14**	**-1,19768334**	**0,435974803**	**7,12E-07**	**0,318237496**
BSU25130	Nfo	-0,34384673	0,78793759	0,144045714	-0,05031827	0,965723262	0,661863536	0,876830426

BSU25140	CshB	-0,62535355	0,648260892	0,008123276	0,090938599	1,065062874	0,356608305	0,856661883
**BSU25150**	**YqfQ**	**-2,25687423**	**0,2092248**	**1,13E-12**	**-2,17705119**	**0,221127262**	**2,28E-14**	**0,215176031**
BSU25160	IspH	-0,64895292	0,637743008	0,011259999	-0,57532851	0,671133413	0,018976146	0,654438211
BSU25170	YqfO	0,623923021	1,541059987	0,006796885	0,134486639	1,097702144	0,413315051	1,319381065
BSU25180	TrmK	-0,27402222	0,827010633	0,242353184	0,164967163	1,12114056	0,374494285	0,974075596
BSU25190	CccA	0,394920797	1,314870572	0,018952144	-0,44099045	0,736628722	0,032339905	1,025749647
BSU25200	SigA	0,118772493	1,085810615	0,467583916	-0,5022009	0,706028877	0,040006493	0,895919746
BSU25210	DnaG	-0,69426238	0,61802522	0,000466803	-0,22897309	0,853242015	0,720815182	0,735633618
BSU25220	AntE	0,809929055	1,75312523	0,000198613	-1,05165958	0,482412911	4,02E-06	1,11776907
BSU25230	YqxD	-1,09221441	0,469040884	5,64E-07	-0,25823327	0,836111197	0,482281882	0,652576041
BSU25240	YqfL	-1,18565465	0,439625004	1,82E-07	0,074358085	1,052892456	0,583284661	0,74625873
BSU25250	CcpN	-0,92477264	0,526763526	1,01E-05	-0,20082402	0,870053478	0,297406118	0,698408502
BSU25260	GlyS	-0,63303726	0,644817472	0,001007157	-0,07405824	0,94996203	0,55700603	0,797389751
BSU25270	GlyQ	-0,41426857	0,750399841	0,163922169	0,641426656	1,559870927	0,004753098	1,155135384
BSU25280	RecO	0,295511404	1,227319958	0,016400672	-0,02866231	0,980328852	0,364709186	1,103824405
BSU25289	YqzL	0,276027678	1,210856314	0,159591305	0,083138472	1,059320005	0,085959127	1,135088159
BSU25290	Era	0,017538072	1,012230655	0,454106832	0,45169072	1,367642082	0,000651996	1,189936369
BSU25300	Cdd	0,199651592	1,14842098	0,314163597	0,362411132	1,285572641	0,004161364	1,216996811
BSU25310	DgkA	-0,54701939	0,684432709	0,027001854	0,760930056	1,694582712	0,000454036	1,18950771
BSU25320	YqfG	-0,61746945	0,651813235	0,015587016	0,570480678	1,485018267	0,000998194	1,068415751
BSU25330	YqfF	-0,20536845	0,867317155	0,218886398	0,719122452	1,646180408	0,000227147	1,256748782
BSU25340	PhoH	0,437578433	1,354329174	0,022706773	0,134899907	1,098016632	0,335045354	1,226172903
BSU25350	YqfD	-0,06333788	0,957047289	0,202731435	0,073611264	1,05234756	0,149943045	1,004697424
BSU25360	YqfC	-0,13167252	0,912772656	0,275875653	-0,58772795	0,665389982	0,004764295	0,789081319
BSU25370	YqfB	-0,88692997	0,540763631	1,42E-05	-0,95929903	0,514306743	0,000252516	0,527535187
BSU25380	YqfA	-0,89791488	0,536661807	0,000302251	-0,4619919	0,725983216	0,204269109	0,631322511
BSU25390	YqeZ	-0,91806344	0,529218927	7,87E-05	-0,16619066	0,891192711	0,633203997	0,710205819
BSU25400	YqeY	0,383370755	1,304385903	0,038857709	-0,27436922	0,82681174	0,358526543	1,065598822
BSU25410	RpsU	0,382786847	1,30385808	0,01279037	-0,26933145	0,829703942	0,857359417	1,066781011

BSU25420	YqeW	1,41458733	2,665834707	5,85E-10	0,8447403	1,795941429	6,86E-05	2,230888068
BSU25430	YqeV	-0,89866163	0,536384098	0,000685539	-0,23730655	0,848327629	0,161717124	0,692355863
BSU25440	YqeU	-0,78715062	0,579487473	0,00034149	0,157224594	1,115139807	0,234624934	0,84731364
BSU25450	YqeT	-0,84649007	0,556136113	0,000727227	0,368647069	1,291141456	0,054422459	0,923638785
BSU25460	DnaJ	-0,78101738	0,581956255	0,004412367	0,096944826	1,069506185	0,417319083	0,82573122
BSU25470	DnaK	-0,47368477	0,72012299	0,004078355	-0,20650576	0,866633701	0,742048785	0,793378345
BSU25480	GrpE	-0,32420778	0,798736875	0,138176767	0,227000233	1,170398831	0,097861503	0,984567853
BSU25490	HrcA	-0,3798031	0,768542477	0,03077344	0,346097549	1,271117634	0,042584922	1,019830056
BSU25500	HemN	-1,39061851	0,381401254	1,38E-06	-0,23435329	0,85006597	0,258493901	0,615733612
BSU25510	LepA	0,447982883	1,364131649	0,011230823	0,446272005	1,362514898	0,013782124	1,363323274
BSU25520	YqxA	0,24152611	1,182242599	0,036112897	-0,1257896	0,916502299	0,569927937	1,049372449
BSU25530	SpoIIP	-0,28247291	0,82218052	0,116312783	-0,1518491	0,900096072	0,561845114	0,861138296
BSU25540	Gpr	-0,75093899	0,594216681	0,00010236	-0,53348991	0,690881452	0,048380043	0,642549067
BSU25550	RpsT	0,127915085	1,092713421	0,082775397	-1,13414135	0,455605999	1,59E-06	0,77415971
BSU25560	HolA	-0,01222349	0,991563112	0,054978305	-0,27866031	0,824356162	0,276012758	0,907959637
BSU25569	YqzM	2,693324249	6,468020488	2,15E-17	-1,62987374	0,323116484	6,44E-10	3,395568486
BSU25570	ComEC	-0,74943346	0,594837102	0,000860845	-0,21790544	0,859812837	0,287799931	0,72732497
BSU25580	ComEB	-0,39929107	0,758230779	0,023844229	-0,16269879	0,893352352	0,399993348	0,825791565
BSU25590	ComEA	-0,79297297	0,577153522	0,004945096	-0,62136886	0,650053852	0,005593775	0,613603687
BSU25600	ComER	-1,11798622	0,460736495	1,21E-05	-0,86142636	0,550408112	0,003303285	0,505572303
BSU25610	YqeM	-0,43577689	0,739295538	0,066979413	-0,28402993	0,821293662	0,153956704	0,7802946
BSU25620	YqeL	-0,79748952	0,575349491	0,002047639	-0,21053949	0,864214	0,389413272	0,719781746
BSU25630	YqeK	-1,35962727	0,389682955	3,86E-06	-0,29398007	0,815648763	0,358077609	0,602665859
BSU25640	NadD	-1,26674942	0,41559511	1,12E-05	-0,30108312	0,811642815	0,225496363	0,613618963
BSU25650	YqeI	-1,30922372	0,403537956	7,97E-07	-0,18604573	0,879011698	0,638416053	0,641274827
BSU25660	AroD	-1,15550577	0,448908785	0,000108551	-0,20362588	0,868365384	0,601671953	0,658637084
BSU25670	YqeH	-1,45847921	0,3638765	2,20E-07	-0,24989328	0,840958621	0,235217233	0,60241756
BSU25680	YqeG	-1,23062821	0,426131849	2,87E-06	-0,24420034	0,844283639	0,112238701	0,635207744
BSU25690	Sda	0,220978798	1,165524069	0,190474274	1,26218191	2,398582246	9,63E-08	1,782053158

BSU25700	YqeF	-0,7298741	0,60295653	0,01808084	-0,53199231	0,691599	0,026935517	0,647277765
**BSU25710**	**CwlH**	**-1,90663159**	**0,266714545**	**1,68E-08**	**-1,58174159**	**0,334078353**	**6,45E-09**	**0,300396449**
BSU25720	YqeD	0,704701037	1,629806892	6,12E-05	0,356738792	1,280527993	0,162393745	1,455167443
BSU25730	YqeC	-1,7602666	0,295193611	3,87E-10	-0,80691225	0,571603933	0,000530697	0,433398772
BSU25740	YqeB	-1,06605804	0,477622254	3,63E-05	-1,04455248	0,484795268	0,000107105	0,481208761
BSU25750	NucB	-0,34484001	0,787395294	0,161700985	-1,13823845	0,454313964	1,57E-05	0,620854629
BSU25770	SpoIVCA	-1,74395465	0,298550182	1,52E-11	-0,93605333	0,522660731	8,95E-05	0,410605457
BSU25780	ArsC	0,110479227	1,079586788	0,073313045	-0,08450461	0,943108323	0,674352107	1,011347556
BSU25790	ArsB	-0,20154153	0,869620872	0,141113359	0,179395028	1,132408928	0,104244836	1,0010149
BSU25800	YqcK	-0,7930544	0,577120947	0,001269359	0,346542424	1,271509662	0,028142542	0,924315304
BSU25810	ArsR	-0,53021735	0,692450403	0,081428641	-0,33944164	0,790347135	0,029845032	0,741398769
**BSU25820**	**YqcI**	**-3,02358894**	**0,122972792**	**3,11E-12**	**-2,34215982**	**0,197214862**	**2,90E-13**	**0,160093827**
BSU25830	RapE	0,084029928	1,059974772	0,114292093	0,314664589	1,243722468	0,091964182	1,15184862
BSU25840	PhrE	1,761382093	3,390227513	6,75E-12	0,017636384	1,012299636	0,697696551	2,201263574
BSU25850	YqzI	-0,07153128	0,951627403	0,662467671	0,689618887	1,612857398	0,001291803	1,282242401
BSU25860	YqcG	0,189833345	1,140631946	0,071471494	0,288993567	1,221787654	0,039831897	1,1812098
BSU25870	YqcF	-0,39912394	0,758318624	0,017481233	0,40621922	1,325208369	0,030673714	1,041763496
BSU25880	YqxJ	0,211889441	1,158204046	0,310762502	0,536998984	1,450951186	0,001790341	1,304577616
BSU25890	YqxI	0,52289392	1,436834523	5,34E-05	0,50982823	1,423880656	0,001334861	1,430357589
BSU25900	CwlA	1,025754884	2,036024447	0,000312368	0,30093177	1,23193981	0,44352941	1,633982128
BSU25910	YqxH	0,739746278	1,669882136	0,408577181	-0,26140182	0,834276887	0,773896983	1,252079511
BSU25920	YqxG	-0,41379666	0,750645343	0,493800426	-0,36627806	0,77578132	0,005442687	0,763213332
BSU25930	YqcE	0,068278781	1,048465058	0,870695473	0,080935539	1,057703703	0,897426167	1,05308438
BSU25940	YqcD	0,658280858	1,57820089	0,092951287	-0,33689353	0,791744294	0,023875248	1,184972592
BSU25950	YqcC	0,721806103	1,649245425	2,85E-05	-0,29124227	0,817198085	1,90E-05	1,233221755
BSU25960	YqcB	0,116149921	1,08383859	1	0,080935539	1,057703703	0,86368825	1,070771147
BSU25970	YqcA	-0,20683323	0,866437009	0,923318088	-0,7701698	0,586348459	0,686542103	0,726392734
BSU25980	YqbT	1,240709777	2,363147658	1,20E-05	-0,08418933	0,943314451	0,290806385	1,653231055
BSU25990	YqbS	0,147579049	1,107709092	1	0,080935539	1,057703703	1	1,082706398

BSU26000	YqbR	-0,0215708	0,985159483	0,193372146	0,217890538	1,163031795	0,549549952	1,074095639
BSU26010	YqbQ	-0,98470008	0,505330765	0,000674226	0,198274304	1,147325148	0,586875551	0,826327957
BSU26020	YqbP	-0,04284144	0,970741151	0,394647776	0,375328006	1,297134433	0,205543726	1,133937792
BSU26030	YqbO	-1,44188886	0,36808507	0,084572935	0,080935539	1,057703703	0,423874334	0,712894387
BSU26050	TxpA	3,087162315	8,498229561	1,65E-12	-0,18838693	0,877586397	0,540789658	4,687907979
BSU26060	YqbM	1,240964205	2,363564451	1,18E-08	-0,05595567	0,961957014	0,360652059	1,662760732
BSU26075	YqbK	-1,73513267	0,300381388	0,008110843	0,420058061	1,337981401	0,58471486	0,819181394
BSU26089	YqzN	0,051950221	1,036665331	0,987514252	0,080935539	1,057703703	0,544389092	1,047184517
BSU26090	YqbJ	-0,53693537	0,68923345	0,294079476	-0,84481205	0,556783339	0,439698911	0,623008395
BSU26100	YqbI	0,686172518	1,609009136	0,080111518	0,650683056	1,569911307	0,216184679	1,589460222
BSU26110	YqbH	0,638272368	1,556464172	4,04E-05	-0,00912138	0,993697486	0,797599866	1,275080829
BSU26120	YqbG	-1,03075768	0,489453029	0,25449855	0,080935539	1,057703703	0,098708307	0,773578366
BSU26130	YqbF	-0,76099697	0,590088411	0,260677217	0,080935539	1,057703703	0,595296514	0,823896057
BSU26140	YqbE	-0,30276373	0,81069788	0,28533827	0,90938124	1,878239764	0,003010654	1,344468822
BSU26150	YqbD	0,137426141	1,099941	0,64253824	-0,63942043	0,641970793	0,155732374	0,870955897
BSU26160	YqbC	1,36024278	2,567283787	0,001551228	0,904383861	1,871744949	0,009425587	2,219514368
BSU26170	YqbB	-0,58828762	0,665131904	0,243652185	0,283622562	1,217247521	0,105455868	0,941189713
BSU26180	YqbA	-0,58293638	0,667603591	0,001031063	0,47997413	1,394718657	0,410372112	1,031161124
BSU26190	YqaT	-1,15708387	0,448418012	0,198534971	-0,0770418	0,947999501	0,497367259	0,698208757
BSU26200	YqaS	-1,33283216	0,396988148	0,122698268	-0,10126724	0,93221379	0,252389578	0,664600969
BSU26210	YqaR	0,776323042	1,71276004	0,000511851	0,28205271	1,215923707	0,240696823	1,464341874
BSU26220	YqaQ	-0,02578979	0,982282709	0,066069503	0,080935539	1,057703703	0,323315255	1,019993206
BSU26230	YqaP	1,12994089	2,188497733	1,14E-06	-0,09507546	0,93622328	0,54809369	1,562360507
BSU26240	YqaO	0,993108202	1,990468723	0,00292805	-0,75354535	0,593144142	0,026360621	1,291806432
BSU26250	YqaN	1,248540749	2,376009737	3,26E-07	-0,2504712	0,840621813	0,117537281	1,608315775
BSU26259	YqzO	-1,55839759	0,339527989	0,026876913	-0,64206834	0,640793607	0,296148983	0,490160798
BSU26260	YqaM	-0,94080064	0,520943698	0,589538563	-0,07734293	0,947801645	0,82395819	0,734372672
BSU26270	YqaL	0,821017821	1,766651925	0,003077136	0,09652128	1,069192246	0,556896011	1,417922085
BSU26280	YqaK	-0,45462918	0,729697707	0,001075148	-0,16541005	0,891675046	0,552461119	0,810686376

BSU26290	YqaJ	0,103250631	1,074191069	0,882097545	0,080935539	1,057703703	0,176937782	1,065947386
BSU26300	YqaI	1,806189345	3,497173434	4,21E-12	-0,47358561	0,720172486	0,020165316	2,10867296
BSU26310	YqaH	-0,28918015	0,818366987	0,765020968	-0,34949752	0,784857408	0,908805295	0,801612198
BSU26320	YqaG	0,000871749	1,000604433	0,898830732	-0,82701066	0,563696043	0,312280707	0,782150238
BSU26330	YqdA	0,140708332	1,10244626	1	0,080935539	1,057703703	0,995969071	1,080074982
BSU26340	YqaF	-0,84579138	0,556405512	0,553336138	-1,40478675	0,377673968	0,158125219	0,46703974
BSU26350	SknR	0,545937831	1,459969096	0,00107147	-0,04224509	0,9711425	0,672452549	1,215555798
BSU26360	YqaD	-0,91947202	0,528702472	0,004882554	0,001064228	1,000737939	0,19468705	0,764720205
BSU26370	YqaC	-1,16196467	0,446903525	1,02E-07	-0,55005246	0,682995292	0,021076637	0,564949408
BSU26380	YqaB	-0,25726361	0,836673351	0,333706641	0,026542629	1,018568233	0,205945594	0,927620792
BSU26400	YrkS	-1,01086776	0,496247671	1,12E-05	-0,24105514	0,846126258	0,294912635	0,671186965
BSU26410	YrkR	-0,60417287	0,657848432	0,002789947	0,283252884	1,216935652	0,076446228	0,937392042
BSU26420	YrkQ	-0,82628169	0,563980941	0,001069193	0,611017571	1,5273361	0,009397075	1,04565852
BSU26430	YrkP	1,073123725	2,103983988	1,78E-05	0,055041964	1,038889321	0,147340038	1,571436654
BSU26440	YrkO	-0,23090426	0,852100641	0,082137851	0,054314877	1,038365876	0,287322911	0,945233259
BSU26450	YrkN	-0,02000725	0,986227749	0,134255027	-0,09810478	0,934259493	0,43839617	0,960243621
BSU26470	YrkL	-0,0920971	0,938158051	0,584347612	0,339166878	1,265025861	0,082870097	1,101591956
BSU26480	YrkK	0,121640453	1,087971265	0,037391801	1,239113455	2,360534314	1,71E-06	1,724252789
BSU26490	YrkJ	1,281755651	2,431346735	2,87E-08	-0,8100095	0,570378102	0,001097073	1,500862419
BSU26500	YrkI	-1,2245027	0,427945	0,000220348	-0,34434573	0,787665105	0,348015268	0,607805053
BSU26510	YrkH	-2,79634603	0,143951425	7,15E-11	-0,30312617	0,810494237	0,219699926	0,477222831
BSU26530	YrkF	-1,96982136	0,25528464	9,18E-05	-0,1318254	0,912675935	0,104092869	0,583980287
BSU26540	YrkE	-1,66651155	0,31501413	0,022262542	-0,99362703	0,502213589	0,233568519	0,40861386
BSU26550	YrkD	-0,03288274	0,977465211	0,25850594	-0,49232622	0,710877944	0,014920781	0,844171577
BSU26560	YrkC	-0,83292393	0,56139031	0,003069299	-1,17759262	0,442088583	7,41E-07	0,501739447
BSU26570	YrkB	0,385136996	1,305983795	0,003796351	-0,16544964	0,891650577	0,577530737	1,098817186
BSU26580	BltR	0,313218343	1,242476309	0,002385179	0,111743388	1,080533191	0,505357396	1,16150475
BSU26590	Blt	1,202132698	2,300795402	0,000168007	0,014277552	1,009945577	0,078255447	1,655370489
BSU26600	BltD	0,572449628	1,487046362	0,038325245	0,022376758	1,015631297	0,004072384	1,25133883

BSU26610	YrkA	1,348444119	2,546373626	1,74E-07	0,017843458	1,012444944	0,469586917	1,779409285
BSU26619	YrzO	0,396776938	1,316563346	0,187379436	-0,15483257	0,898236611	0,331902653	1,107399979
BSU26620	YrdR	-0,75758595	0,59148523	0,002921292	-0,00864288	0,994027118	0,267935109	0,792756174
BSU26630	YrdQ	0,374126994	1,296055047	0,022663791	0,140457106	1,102254301	0,122003467	1,199154674
BSU26640	TrkA	0,634033046	1,551897256	0,000774146	-0,28738439	0,81938626	0,233932985	1,185641758
BSU26650	CzcD	-2,33491559	0,198207631	8,82E-12	-0,1577735	0,896407425	0,162731834	0,547307528
BSU26660	YrdN	-1,40258613	0,378250493	0,00014865	-0,84422638	0,557009416	0,006151611	0,467629955
BSU26670	GltR	-0,75799022	0,591319508	0,014558667	-0,27036551	0,829109465	0,256145517	0,710214486
BSU26680	YrdK	-0,61560178	0,652657598	0,043169797	0,487454163	1,401968722	0,097926936	1,02731316
BSU26690	BrnQ	0,88327044	1,844551974	0,000443245	0,555582728	1,469762175	0,008033581	1,657157074
BSU26700	AzlD	-0,01675002	0,988456907	0,351485692	0,693640985	1,617360166	0,00270674	1,302908537
BSU26710	AzlC	0,546490085	1,46052807	0,005167775	0,312263515	1,241654264	0,014029029	1,351091167
BSU26720	AzlB	1,085481681	2,122083876	7,07E-06	-0,05679703	0,961396173	0,762565215	1,541740025
BSU26730	YrdF	-1,04179081	0,485724172	0,001995665	0,136747182	1,09942347	0,423666181	0,792573821
BSU26740	CypA	-1,48104395	0,358229499	4,69E-10	-0,32012212	0,801002071	0,073512611	0,579615785
BSU26760	YrdC	-0,0145623	0,989956956	0,001650438	0,16649063	1,122325094	0,174126939	1,056141025
BSU26770	YrdB	1,111922956	2,161335379	4,23E-06	0,204829093	1,152549803	0,232998932	1,656942591
BSU26780	YrdA	-0,29972578	0,812406802	0,328711093	-0,17294146	0,887032296	0,473709469	0,849719549
BSU26790	AadK	0,409987398	1,328674208	0,031877184	1,039607038	2,055667653	6,64E-07	1,692170931
BSU26800	YrpB	0,390098687	1,310483044	0,054532506	0,724973481	1,652870254	0,001611426	1,481676649
BSU26810	YrpC	-0,24074984	0,846305334	0,111739601	0,726527008	1,654651061	0,0757819	1,250478197
BSU26820	YrpD	1,268312292	2,408796124	8,32E-08	0,499463422	1,413687676	0,017833182	1,9112419
BSU26830	ZinT	0,261667501	1,198863579	0,001838636	-0,15977946	0,895161899	0,776944655	1,047012739
BSU26840	SigZ	1,217092099	2,32477662	0,001152157	-0,0763755	0,948437428	0,31096324	1,636607024
BSU26850	YrpG	-1,16118522	0,447145039	0,058070532	0,024352035	1,017022809	0,784639323	0,732083924
BSU26860	YraO	0,393365192	1,31345356	0,011867314	1,289676692	2,44473263	9,36E-08	1,879093095
BSU26870	YraN	-0,3587603	0,779834399	0,075133137	1,769317394	3,408926265	2,95E-09	2,094380332
BSU26880	YraM	-0,69592005	0,617315512	1,35E-06	-0,65930405	0,63318367	0,257286916	0,625249591
BSU26890	Csn	0,445036917	1,361348951	0,012298853	0,680941375	1,603185511	0,001219032	1,482267231

BSU26900	YraL	1,101007817	2,145044856	6,89E-07	-0,40207495	0,756769081	0,083246838	1,450906969
BSU26910	YraK	-1,29374849	0,40788985	1,96E-07	-0,48286858	0,715553441	0,018506003	0,561721645
BSU26920	YraJ	-0,25663604	0,837037382	0,459567779	-0,17893103	0,88335728	0,389636007	0,860197331
BSU26930	YraI	-0,41796125	0,748481596	0,12144065	0,027710123	1,019392838	0,497611892	0,883937217
BSU26940	YraH	-0,34531817	0,787134362	0,001747141	-0,06723324	0,954466696	0,682639764	0,870800529
**BSU26950**	**YraG**	**-3,15725131**	**0,112091493**	**4,68E-18**	**-1,76335329**	**0,29456271**	**5,37E-11**	**0,203327101**
**BSU26960**	**YraF**	**-3,04293763**	**0,121334555**	**1,85E-18**	**-1,54647755**	**0,342344907**	**1,45E-09**	**0,231839731**
**BSU26970**	**AdhB**	**-2,9000425**	**0,133967737**	**9,45E-18**	**-1,34753045**	**0,392964136**	**5,21E-08**	**0,263465936**
**BSU26980**	**YraE**	**-3,46424491**	**0,090606294**	**2,48E-19**	**-1,63857273**	**0,321174057**	**2,13E-10**	**0,205890176**
**BSU26990**	**YraD**	**-3,36398763**	**0,097126741**	**5,04E-19**	**-1,83002982**	**0,281258809**	**3,59E-11**	**0,189192775**
BSU27000	AdhR	0,074705645	1,053146139	0,067543224	-0,11677848	0,922244714	0,193749639	0,987695426
BSU27010	AdhA	-1,2948967	0,407565349	1,82E-05	-0,02090379	0,985615062	0,566457647	0,696590205
BSU27020	YraA	-0,16441261	0,892291742	0,575091533	-0,36249042	0,777820726	0,104055576	0,835056234
BSU27030	SacC	0,69173647	1,615226483	9,17E-05	0,140645226	1,102398039	0,337343839	1,358812261
BSU27040	LevG	-1,17087631	0,444151476	4,30E-08	0,531828635	1,445760555	0,010450135	0,944956015
BSU27050	LevF	-1,95234558	0,258395781	3,29E-12	0,59772946	1,51333298	0,011074823	0,885864381
BSU27060	LevE	-1,37288965	0,386117098	2,94E-07	0,538178806	1,452138245	0,02054196	0,919127672
BSU27070	LevD	-1,68201584	0,311646878	2,23E-10	0,270249802	1,206016631	0,180109344	0,758831754
BSU27080	LevR	-0,69829172	0,616301531	0,019273126	0,009201658	1,006398487	0,131050802	0,811350009
BSU27090	AapA	-1,20975765	0,432341237	5,60E-05	-0,20363417	0,868360393	0,428208853	0,650350815
BSU27100	YrhP	-0,31983966	0,801158914	0,20190004	-0,63330132	0,644699462	0,007189894	0,722929188
BSU27110	YrhO	0,676446152	1,598197998	0,000164622	-0,44771578	0,733202809	0,079163645	1,165700403
BSU27120	SigV	-1,52359727	0,347817572	1,09E-05	-0,82838492	0,563159339	0,034061847	0,455488456
BSU27130	RsiV	-1,35725565	0,390324074	0,000246398	-0,65363415	0,635677026	0,032811739	0,51300055
BSU27140	Oat	-0,43136777	0,741558404	0,144115551	-0,32841859	0,796408988	0,289207094	0,768983696
BSU27150	YrhK	-0,1110635	0,925905271	0,130334404	-1,16631128	0,445559104	0,006749757	0,685732188
BSU27160	YrhJ	-1,20483097	0,43382017	5,21E-06	0,006199619	1,004306495	0,625439227	0,719063332
BSU27170	FatR	-2,36794364	0,193721551	8,17E-13	0,009291838	1,006461397	0,149120217	0,600091474
BSU27180	YrhH	0,436157178	1,352995629	0,236731739	0,052747982	1,03723873	0,750092109	1,195117179

BSU27190	YrzI	-0,85560926	0,552631896	0,000519631	0,043159256	1,030367688	0,520192447	0,791499792
BSU27200	YrhG	-0,54986715	0,683083028	0,029035186	-0,43081662	0,741841755	0,071925853	0,712462391
BSU27210	YrhF	1,262386086	2,398921728	1,19E-06	0,400891705	1,320323728	0,13140343	1,859622728
BSU27220	YrhE	-0,78834376	0,579008421	0,005675104	-0,01815082	0,987497626	0,404866555	0,783253024
BSU27230	YrhD	0,135082074	1,098155285	0,476417941	-0,39886571	0,75845437	0,125664916	0,928304828
BSU27240	YrhC	1,305824622	2,47224997	0,000694288	0,216992016	1,162307675	0,398603292	1,817278822
BSU27250	MccB	-0,70650662	0,612802199	0,000858421	0,450358208	1,366379474	0,118610465	0,989590837
BSU27260	MccA	-1,80518852	0,286143647	2,97E-09	0,07619458	1,054233601	0,393583967	0,670188624
BSU27270	MtnN	-0,47676664	0,718586313	0,032130276	-0,04952135	0,966256853	0,164307326	0,842421583
BSU27280	YrrT	-2,76925454	0,146680141	6,92E-12	0,899940569	1,865989113	0,003467395	1,006334627
BSU27290	YrzA	-0,81032691	0,570252625	0,001055852	-0,76815163	0,587169271	0,001094179	0,578710948
BSU27300	YrrS	0,442059185	1,358542016	0,006559066	0,384922729	1,305789847	0,040271033	1,332165932
BSU27310	PbpI	0,198673758	1,147642864	0,48222622	0,461644192	1,377110372	0,006445931	1,262376618
BSU27320	GreA	0,293731622	1,225806807	0,030032	-0,40017963	0,757763928	0,118858062	0,991785367
BSU27330	Udk	-1,29435353	0,407718825	2,12E-08	0,02761424	1,019325091	0,6830897	0,713521958
BSU27340	YrrO	-0,93915599	0,521537902	0,000297205	-0,13756629	0,909051362	0,720724967	0,715294632
BSU27350	YrrN	-0,50472952	0,704792499	0,056215495	0,666863319	1,587617444	0,001149996	1,146204972
BSU27360	YrrM	-1,11467322	0,461795742	2,50E-05	0,806494473	1,74895658	0,000437168	1,105376161
BSU27370	YrrL	-0,94624629	0,51898103	0,004438956	-0,22026437	0,858408121	0,283960008	0,688694576
BSU27380	YrzB	0,323762508	1,251590409	0,003451161	-0,38095484	0,767929171	0,109372776	1,00975979
BSU27390	YrrK	-0,42916765	0,742690148	0,036975643	-0,36881229	0,77441978	0,17148817	0,758554964
BSU27400	YrzL	0,3616868	1,284927357	0,056421597	0,155570088	1,113861679	0,371213508	1,199394518
BSU27410	AlaS	-0,73056589	0,602667474	2,47E-05	-0,34062221	0,789700655	0,28118388	0,696184065
**BSU27420**	**YrrI**	**-2,62717212**	**0,161861063**	**7,85E-16**	**-1,46762581**	**0,361576842**	**3,81E-09**	**0,261718952**
BSU27430	GlnQ	-0,98647882	0,504708112	6,35E-07	-0,29399606	0,81563972	0,868945381	0,660173916
BSU27440	GlnH	-0,64776762	0,638267182	0,00431099	-0,57822168	0,669788874	0,239766485	0,654028028
BSU27450	GlnM	-0,78772976	0,579254898	0,000103309	-0,53618562	0,689591731	0,116248007	0,634423315
BSU27460	GlnP	-0,67269491	0,627333752	0,002370027	-0,85157013	0,554181274	0,000870406	0,590757513
**BSU27468**	**YrzQ**	**-1,89815094**	**0,268287002**	**5,60E-11**	**-2,6453677**	**0,159832456**	**6,50E-16**	**0,214059729**

**BSU27469**	**YrzR**	**-1,88702077**	**0,270364799**	**1,43E-12**	**-2,46321707**	**0,181341739**	**1,07E-14**	**0,225853269**
**BSU27470**	**YrrD**	**-2,75303175**	**0,148338835**	**1,49E-17**	**-2,63831876**	**0,1606153**	**8,87E-16**	**0,154477068**
BSU27480	YrrC	-0,25995063	0,835116497	0,039598855	0,12338224	1,089285582	0,218805088	0,962201039
BSU27490	YrrB	-0,83014403	0,562473087	0,004684263	0,433791003	1,350778389	0,133678593	0,956625738
BSU27500	TrmU	-0,34602776	0,786747304	0,044739141	-0,22499527	0,85559783	0,290665626	0,821172567
BSU27510	YrvO	-0,36913137	0,774248524	0,002512537	0,14644244	1,106836741	0,280137062	0,940542633
BSU27520	CymR	-0,76272057	0,58938385	0,000671032	0,14070155	1,102441077	0,411307559	0,845912464
BSU27530	YrvN	0,3086557	1,238553081	0,137787867	0,177169452	1,130663362	0,429003181	1,184608222
BSU27540	YrvM	1,130082852	2,188713094	4,14E-07	0,008185577	1,005689936	0,448215234	1,597201515
BSU27550	AspS	-0,33697471	0,791699746	0,064124113	0,236055046	1,177767726	0,137330841	0,984733736
BSU27560	HisS	-0,254678	0,838174187	0,050128112	0,427603619	1,344997619	0,015753287	1,091585903
**BSU27570**	**YrzK**	**-1,59262966**	**0,331566542**	**1,16E-09**	**-1,4714335**	**0,360623796**	**6,09E-09**	**0,346095169**
BSU27580	YrvJ	-0,33909917	0,790534772	0,155069515	0,366925915	1,289602026	0,035795095	1,040068399
BSU27590	YrvI	0,645540369	1,564325103	0,00061048	0,456881168	1,372571371	0,011768819	1,468448237
BSU27600	RelA	0,019944832	1,013920707	0,06363092	0,571181292	1,48573961	0,004154544	1,249830158
BSU27610	Apt	-0,27230849	0,827993592	0,052476817	-0,05199076	0,964604365	0,534490086	0,896298978
BSU27620	YrvE	-0,58189057	0,668087711	0,005708358	0,200732488	1,149281722	0,125787853	0,908684716
BSU27630	YrvD	0,041500625	1,029183779	0,00026682	-0,45599516	0,729007137	0,02658235	0,879095458
BSU27640	YrvC	-0,42078568	0,747017695	0,006443021	0,431314761	1,348461901	0,033380682	1,047739798
BSU27650	SecDF	0,395101874	1,315035616	0,051123916	0,54729273	1,461340862	0,000527027	1,388188239
BSU27660	ComN	0,569067033	1,483563864	0,000494304	0,738808265	1,668796764	0,000560784	1,576180314
BSU27670	SpoVB	0,543977475	1,457986614	0,000573521	0,77189991	1,707516962	0,000293626	1,582751788
**BSU27680**	**YrbG**	**-2,02676346**	**0,245404999**	**7,03E-12**	**-1,74934079**	**0,297437655**	**8,21E-11**	**0,271421327**
BSU27690	YrzE	1,278717756	2,426232414	5,84E-08	0,491008019	1,405426512	0,005526183	1,915829463
BSU27700	YrbF	0,30665943	1,23684047	0,003795396	0,327039817	1,254436825	0,05196159	1,245638647
BSU27710	Tgt	0,440373865	1,356955929	0,000420081	0,351753942	1,276111104	0,026110393	1,316533516
BSU27720	QueA	-0,00577151	0,996007483	0,735199962	0,169342428	1,124545807	0,269915259	1,060276645
BSU27729	YrzS	-0,29808442	0,813331602	0,101735082	0,049287177	1,034753535	0,289269971	0,924042568
BSU27730	RuvB	-1,24308689	0,422467746	1,71E-05	0,622579381	1,539625404	0,007798893	0,981046575

BSU27740	RuvA	-0,982507	0,506099515	0,00031484	0,339529275	1,265343668	0,094331752	0,885721592
BSU27750	BofC	0,85660455	1,810771556	3,92E-05	0,034624641	1,02429029	0,800372189	1,417530923
BSU27760	CsbX	1,69576985	3,239497064	3,78E-10	0,859586957	1,81451874	2,25E-05	2,527007902
BSU27770	YrbE	0,701476854	1,626168613	1,19E-05	0,776765312	1,713285181	4,42E-05	1,669726897
BSU27785	YrzF	-0,20488849	0,867605742	0,098935316	-0,46641808	0,723759318	0,007699981	0,79568253
BSU27800	YrzH	-0,08837176	0,940583704	0,089195347	-0,23088449	0,852112315	0,143814797	0,896348009
BSU27809	YrzT	1,158253487	2,231870758	0,000130464	-0,50077089	0,706729046	0,044408408	1,469299902
BSU27810	YrbD	-1,17173008	0,443888709	7,70E-07	-0,59636604	0,661417883	0,055948729	0,552653296
BSU27820	YrbC	0,374850943	1,296705574	0,000118459	-0,57247452	0,672462386	0,026965444	0,98458398
**BSU27830**	**CoxA**	**-2,50382125**	**0,176309089**	**5,61E-16**	**-2,18193122**	**0,220380546**	**1,12E-13**	**0,198344818**
BSU27840	SafA	-0,84494215	0,556733134	1,02E-05	-0,7272916	0,604036822	0,24920516	0,580384978
BSU27850	NadA	-1,09862848	0,466960209	4,33E-07	-0,4224208	0,746171519	0,840175244	0,606565864
BSU27860	NadC	-0,51990485	0,697417829	0,007083856	-0,09570551	0,935814504	0,560419875	0,816616167
BSU27870	NadB	-0,48538276	0,714307534	0,004203859	-0,18467446	0,879847591	0,508654836	0,797077562
BSU27880	NifS	-0,22099317	0,857974592	0,04175647	-0,15459993	0,898381467	0,505987774	0,87817803
BSU27890	NadR	-0,42430254	0,745198904	0,035604007	-0,50213296	0,706062128	0,037411155	0,725630516
BSU27900	PheA	-0,62883457	0,646698618	0,021712973	0,571660476	1,486233173	0,013894227	1,066465895
BSU27910	PheB	-0,11339296	0,924411454	0,637903588	0,319126373	1,247574848	0,072436843	1,085993151
BSU27920	Obg	-0,28861887	0,818685435	0,069835817	0,363058574	1,2861497	0,028888439	1,052417568
BSU27930	Spo0B	-1,04296317	0,485329624	9,27E-06	-0,13371466	0,911481536	0,639510678	0,69840558
BSU27940	RpmA	0,947385992	1,928375482	6,89E-05	0,098492525	1,070654149	0,104579936	1,499514815
BSU27950	YsxB	0,881509486	1,842301886	4,82E-05	0,236406702	1,178054842	0,014400434	1,510178364
BSU27960	RplU	0,533985566	1,447923686	0,000792021	0,192114538	1,142436943	0,045155328	1,295180315
BSU27970	SpoIVFB	1,050668919	2,07149009	8,85E-05	0,841307143	1,791672738	1,07E-05	1,931581414
BSU27980	SpoIVFA	0,293461244	1,225577099	0,113957304	0,491075587	1,405492336	0,005077673	1,315534717
BSU27990	MinD	0,988298366	1,9838437	4,59E-05	1,044812899	2,063098782	1,15E-05	2,023471241
BSU28000	MinC	0,903936037	1,871164035	0,000116316	0,925552339	1,899411296	6,10E-06	1,885287665
BSU28010	MreD	0,280663204	1,214753175	0,045014805	0,93323607	1,909554465	3,49E-06	1,56215382
BSU28020	MreC	0,255289547	1,193575268	0,165157111	0,930856068	1,906406886	2,42E-05	1,549991077

BSU28030	MreB	-0,41758553	0,74867655	0,195770425	0,877020492	1,836578411	2,86E-05	1,292627481
BSU28040	RadC	0,454927167	1,370713604	0,023353337	0,794416918	1,734376264	2,01E-05	1,552544934
BSU28050	Maf	0,727912146	1,656240461	6,47E-05	0,741350941	1,671740525	0,002011436	1,663990493
BSU28060	SpoIIB	1,700771268	3,250746977	7,81E-12	0,23636865	1,17802377	0,122919852	2,214385374
BSU28070	ComC	0,530729253	1,444659256	0,006946649	-0,45819264	0,727897574	0,09929752	1,086278415
BSU28080	FolC	0,078867931	1,056188934	0,029166106	0,412083748	1,33060628	0,047615468	1,193397607
BSU28090	ValS	-0,94134717	0,520746388	0,000557266	0,454490606	1,370298887	0,004830469	0,945522638
**BSU28099**	**YszA**	**-1,23414273**	**0,425095022**	**2,38E-05**	**-2,47130952**	**0,180327393**	**1,08E-15**	**0,302711208**
BSU28100	YsxE	-0,53581929	0,689766856	0,003127483	-0,72624409	0,604475557	0,071386947	0,647121206
BSU28110	SpoVID	-0,89622615	0,537290358	0,000401184	-0,44280613	0,735702231	0,627602766	0,636496295
BSU28120	HemL	0,2632815	1,200205544	0,290368863	0,152660015	1,111617164	0,294978255	1,155911354
BSU28130	HemB	-0,76910557	0,586781149	0,000183992	0,335343325	1,261677621	0,032588758	0,924229385
BSU28140	HemD	-0,97283222	0,509504849	4,42E-06	0,389718676	1,310137904	0,029405606	0,909821376
BSU28150	HemC	-1,00937048	0,496762963	0,000240689	0,377425794	1,299021936	0,093080471	0,89789245
BSU28160	HemX	-0,74329868	0,597371918	0,00350367	0,125485837	1,09087503	0,299816056	0,844123474
BSU28170	HemA	-0,66765414	0,629529486	0,002273942	-0,1236325	0,917873662	0,516236541	0,773701574
BSU28180	YsxD	0,903651809	1,870795429	0,000355935	-1,5512762	0,341208101	9,57E-08	1,106001765
BSU28190	YsxC	-0,30139824	0,811465555	0,000156994	-0,36651778	0,775652424	0,031896451	0,79355899
BSU28200	LonA	-0,66324883	0,631454713	0,004176627	-0,17716319	0,884440386	0,608094932	0,75794755
BSU28210	LonB	-0,02599599	0,982142328	0,198019069	-0,22835585	0,853607142	0,270023508	0,917874735
BSU28220	ClpX	0,133638337	1,097056887	0,225510772	-0,49918426	0,707506714	0,030267558	0,9022818
BSU28230	Tig	0,345886354	1,270931569	0,07293658	0,294550134	1,226502466	0,069093815	1,248717018
BSU28240	YsoA	-0,28410155	0,821252889	0,22991139	0,058040357	1,041050719	0,491825096	0,931151804
BSU28250	LeuD	0,194364329	1,144219891	0,361371991	0,051935102	1,036654467	0,261286658	1,090437179
BSU28260	LeuC	0,609963964	1,526221086	0,007126734	0,328660051	1,255846425	0,008829961	1,391033756
BSU28270	LeuB	0,276703052	1,211423289	0,276211301	0,689753673	1,613008089	5,09E-06	1,412215689
BSU28280	LeuA	-0,1419508	0,906292845	0,327725072	0,732749887	1,661803599	2,75E-05	1,284048222
BSU28290	IlvC	-0,07142947	0,951694563	0,096903777	0,352018027	1,276344717	0,003305276	1,11401964
BSU28300	IlvH	-0,07968467	0,94626445	0,332611792	0,123665455	1,08949944	0,070635641	1,017881945

BSU28310	IlvB	-0,15704247	0,896861759	0,079369105	0,304824125	1,23526804	0,009753325	1,0660649
**BSU28320**	**YsnD**	**-2,81971901**	**0,14163807**	**3,10E-17**	**-2,69694767**	**0,15421899**	**1,52E-16**	**0,14792853**
**BSU28330**	**YsnE**	**-2,48091341**	**0,179130958**	**1,08E-15**	**-1,26315167**	**0,416632801**	**1,27E-07**	**0,297881879**
BSU28340	YsnF	1,267573143	2,407562321	7,46E-06	0,948732844	1,93017659	2,87E-05	2,168869456
BSU28350	YsnB	0,333657609	1,260204275	0,061844498	-1,0733406	0,475217346	0,00013435	0,867710811
BSU28360	YsnA	0,333410233	1,259988209	0,015911476	0,278117738	1,212611776	0,099251646	1,236299992
BSU28370	Rph	-0,21529285	0,861371293	0,011870895	0,515867117	1,42985328	0,005718389	1,145612287
BSU28380	GerM	0,840030198	1,790087611	2,99E-05	0,386508416	1,307225848	0,018379217	1,54865673
BSU28390	RacE	-0,27945393	0,823902809	0,250860303	0,048898254	1,034474622	0,558800487	0,929188716
BSU28400	YsmB	-0,47005824	0,721935453	0,045629976	0,127643212	1,092507522	0,430780267	0,907221487
BSU28410	GerE	-0,39695129	0,759461485	1,25E-06	-1,08086843	0,47274417	5,00E-06	0,616102828
BSU28420	YsmA	-0,53859224	0,688442355	0,039324637	0,023761798	1,016606809	0,793990294	0,852524582
BSU28430	SdhB	0,752627967	1,684859122	0,000566639	0,135826431	1,098722024	0,381417506	1,391790573
BSU28440	SdhA	0,089204042	1,063783115	0,415560901	0,514227483	1,428229164	0,002215402	1,246006139
BSU28450	SdhC	0,245767243	1,185723186	0,051522365	0,393446819	1,313527877	0,026523363	1,249625531
BSU28460	YslB	-0,3085426	0,807457037	0,14758855	-0,52889126	0,693087181	0,015424126	0,750272109
**BSU28470**	**LysC**	**-1,84382055**	**0,278583062**	**1,53E-10**	**-1,92181317**	**0,263922605**	**6,28E-12**	**0,271252834**
BSU28490	UvrC	-0,85389995	0,553287046	0,001180979	-0,79310079	0,577102391	0,001020218	0,565194718
BSU28500	TrxA	-0,65987972	0,632931065	0,020986069	-1,23854024	0,423801252	1,36E-07	0,528366159
BSU28510	Abf2	0,397116345	1,316873116	0,030402291	0,667063539	1,587837792	0,00421638	1,452355454
BSU28520	EtfA	1,695308037	3,238460252	2,12E-12	0,063044672	1,04466811	0,349429565	2,141564181
BSU28530	EtfB	0,501368205	1,415555393	0,017336068	0,553611439	1,467755272	0,002188557	1,441655332
BSU28540	FadB	0,773881651	1,709864084	4,77E-05	0,370338588	1,29265617	0,022550284	1,501260127
BSU28550	FadR	0,185110734	1,136904233	0,001764614	0,356805326	1,280587051	0,019186596	1,208745642
BSU28560	LcfA	-0,21876163	0,859302721	0,291521995	0,162630623	1,119326268	0,222467869	0,989314494
BSU28570	YshE	1,557630364	2,943699417	4,23E-09	-0,20384247	0,868235029	0,427100671	1,905967223
BSU28580	MutSB	-0,57655472	0,670563232	0,007032573	0,06066623	1,042947278	0,579416302	0,856755255
BSU28590	PolX	-0,05903726	0,95990447	0,270165775	0,674936076	1,596526031	0,000976179	1,27821525
BSU28600	YshB	0,496747403	1,411028769	0,010235782	0,447790727	1,36394997	0,085588303	1,387489369

BSU28610	ZapA	0,385877157	1,30665399	0,038889285	0,143765259	1,10478471	0,533346287	1,20571935
BSU28620	RnhC	0,165681819	1,121696067	0,060632217	0,142266087	1,103637273	0,149500666	1,11266667
BSU28630	PheT	0,278828089	1,213208986	0,050009257	0,193553937	1,143577339	0,35842361	1,178393162
BSU28640	PheS	-0,41535157	0,749836743	0,091031072	0,401068318	1,32048537	0,021325723	1,035161057
BSU28650	YsgA	0,097976462	1,070271237	0,339812665	-0,16683118	0,890797136	0,519877924	0,980534186
**BSU28660**	**SspI**	**-2,20006539**	**0,217627777**	**8,99E-13**	**-2,08426191**	**0,235816749**	**6,61E-14**	**0,226722263**
BSU28670	YsfB	-0,99758727	0,500836889	6,92E-07	-0,13296826	0,911953229	0,223822609	0,706395059
BSU28680	YsfC	-1,33139871	0,397382789	1,01E-05	0,019329781	1,013488544	0,811460168	0,705435666
BSU28690	YsfD	-0,13019726	0,91370651	0,139588911	-0,53809554	0,688679414	0,021036352	0,801192962
BSU28700	YsfE	0,836824387	1,786114276	1,87E-05	0,765736657	1,700237938	0,000106459	1,743176107
BSU28710	CstA	0,59055741	1,505828438	0,004423573	0,863012389	1,818832123	4,09E-06	1,66233028
BSU28720	AbfA	0,218234806	1,163309361	0,211973176	0,836627719	1,785870809	0,000132649	1,474590085
BSU28730	AraQ	0,419809987	1,337751352	0,00400985	1,162319898	2,238170431	9,83E-07	1,787960892
BSU28740	AraP	0,342217625	1,267703736	0,001852623	1,310310439	2,479948978	8,02E-08	1,873826357
BSU28750	AraN	0,183660837	1,135762227	0,144484671	1,425393255	2,685877032	7,40E-09	1,91081963
BSU28760	AraM	-0,06945382	0,952998722	0,07255797	1,273931806	2,418197045	5,33E-08	1,685597884
BSU28770	AraL	-0,4276694	0,743461843	0,069662118	1,477145715	2,783973957	3,73E-10	1,7637179
BSU28780	AraD	-0,23008653	0,852583753	0,004081334	1,168167439	2,247260608	1,60E-07	1,549922181
BSU28790	AraB	0,068041723	1,048292792	0,117496631	1,191335517	2,283640442	2,95E-08	1,665966617
BSU28800	AraA	0,367062389	1,289724023	0,025609746	1,232764877	2,350169608	1,11E-07	1,819946816
BSU28810	AbnA	0,782322126	1,71989695	0,000802167	1,087671721	2,125307689	7,77E-07	1,92260232
BSU28820	YsdC	-1,23282158	0,425484482	3,17E-07	-0,52809322	0,693470676	0,022740995	0,559477579
BSU28830	YsdB	0,534301311	1,44824061	0,002762628	-0,23365446	0,850477834	0,33656846	1,149359222
BSU28840	YsdA	0,695291824	1,61921193	0,003168397	-0,05810962	0,960521878	0,150542679	1,289866904
BSU28850	RplT	0,955560228	1,93933257	3,25E-07	-0,50559141	0,704371572	0,019716707	1,321852071
BSU28860	RpmI	0,035798572	1,025124101	0,296920964	-0,28594753	0,820202743	0,617082549	0,922663422
BSU28870	InfC	0,394509098	1,314495404	0,014081148	-0,05286704	0,96401865	0,44941545	1,139257027
BSU28890	YscB	-0,05671698	0,961449525	0,790834483	-0,70661463	0,612756325	0,001123443	0,787102925
BSU28900	YsbB	-0,21327026	0,86257974	0,39891452	0,33615335	1,26238621	0,307420407	1,062482975

BSU28910	YsbA	-0,78048807	0,582169809	0,484729838	0,588949403	1,504150999	0,327085091	1,043160404
BSU28920	LytT	0,083036552	1,059245171	0,499033792	0,418904551	1,336912042	0,019205799	1,198078607
BSU28930	LytS	0,280200996	1,214364058	0,061770916	0,654826427	1,57442652	0,001252368	1,394395289
BSU28940	YsaA	0,576733222	1,491468205	0,004169752	-0,07041216	0,952365878	0,65995908	1,221917041
BSU28950	ThrS	0,465966565	1,381242437	0,037414468	0,75390815	1,686354854	0,000696933	1,533798645
BSU28960	YtxC	-0,04691872	0,968001562	0,000234722	0,538239283	1,452199119	0,003785697	1,210100341
BSU28970	YtxB	-0,22594096	0,85503717	0,074264705	0,012222978	1,008508315	0,561205471	0,931772743
BSU28980	DnaI	-0,88365807	0,541991424	0,000504542	0,094057826	1,067368119	0,09047632	0,804679771
BSU28990	DnaB	-0,42013264	0,747355911	0,068692594	0,837622107	1,787102158	0,000266624	1,267229034
BSU29000	YtcG	0,603829381	1,519745115	0,010181058	-0,26734183	0,830848979	0,098844808	1,175297047
BSU29010	SpeD	-0,63753746	0,642809228	0,000321566	-0,42627338	0,744181596	0,438512235	0,693495412
BSU29020	GapB	-0,6320372	0,645264605	0,00204144	-0,20693621	0,866375163	0,436200335	0,755819884
BSU29030	YtcD	-0,87412462	0,54558481	0,003963986	-0,36551628	0,776191059	0,109391078	0,660887934
BSU29040	YtbD	0,700167239	1,624693118	0,001995648	-0,0179929	0,987605722	0,630499885	1,30614942
BSU29050	YtbE	-0,67107962	0,628036529	0,002915264	-0,55917929	0,678688141	0,001987628	0,653362335
BSU29060	YtaG	-0,71710584	0,60831655	0,003687434	-0,20029976	0,870369701	0,157175443	0,739343126
BSU29070	YtaF	-0,83366127	0,561103467	3,32E-05	-0,43442963	0,73998625	0,075043995	0,650544858
BSU29080	MutM	-0,02893133	0,980146067	0,23294195	0,450196357	1,366226194	0,007134773	1,17318613
BSU29090	PolA	-0,61460848	0,653107109	0,016604733	0,374209804	1,296129441	0,049775895	0,974618275
BSU29100	PhoR	0,214787167	1,160532694	0,255610569	0,008035296	1,005585182	0,355728226	1,083058938
BSU29110	PhoP	-0,28190444	0,822504549	0,247297154	0,3579167	1,281573925	0,065359583	1,052039237
BSU29120	Mdh	1,199156249	2,296053486	4,60E-06	0,196609715	1,146002122	0,209087776	1,721027804
BSU29130	Icd	1,001544525	2,002142313	6,55E-05	-0,0083104	0,99425623	0,314480219	1,498199272
BSU29140	CitZ	0,9547	1,93817656	8,34E-05	-0,0576332	0,960839125	0,248397494	1,449507842
BSU29150	YtwI	-0,88556262	0,541276396	0,000322707	-0,76261858	0,589425516	0,000435054	0,565350956
BSU29160	YtvI	0,856011527	1,810027388	2,30E-05	0,072503749	1,051540014	0,38581628	1,430783701
BSU29170	YtzA	-0,20181316	0,869457152	0,083566811	-0,21650662	0,860646911	0,121800245	0,865052032
BSU29180	Pyk	0,284977072	1,218390901	0,030538505	-0,12065538	0,919769727	0,749753132	1,069080314
BSU29190	PfkA	-0,6244793	0,64865385	0,000577345	0,065739255	1,046621104	0,451440636	0,847637477

BSU29200	AccA	0,729620492	1,658202836	7,52E-05	0,61910086	1,535917643	0,001149722	1,597060239
BSU29210	AccD	0,46841359	1,383587217	0,023844366	0,515016148	1,429010135	0,000843762	1,406298676
BSU29220	YtsJ	-0,12722376	0,915591669	0,213749892	-0,24714181	0,842564004	0,569621627	0,879077836
BSU29230	DnaE	-0,91290366	0,531115057	2,71E-05	0,024850072	1,017373959	0,595520982	0,774244508
BSU29239	YtrH	0,771791037	1,70738811	0,000175011	0,592287966	1,507635808	0,005697781	1,607511959
BSU29240	YtrI	0,525942914	1,439874343	0,006780678	0,909457845	1,878339499	8,36E-05	1,659106921
BSU29249	YtzJ	0,081861496	1,058382782	0,743767107	-0,85705786	0,552077283	0,001105423	0,805230032
BSU29250	NrnA	-0,25574395	0,837555121	0,002822035	-0,69944977	0,615807026	0,002917001	0,726681074
BSU29260	YtpI	-0,40205007	0,756782132	0,005806038	-0,53986166	0,687836863	0,0247509	0,722309497
BSU29270	YtoI	0,063783763	1,045203429	0,49103522	0,167711768	1,123275464	0,175830188	1,084239447
BSU29280	YtnM	0,033961388	1,023819499	2,95E-05	0,008948395	1,00622183	0,296655149	1,015020665
BSU29290	YtnL	-2,74327298	0,149345639	1,01E-09	0,526158489	1,440089513	0,007458488	0,794717576
BSU29300	RibR	-2,8758554	0,136232668	3,41E-12	1,181262657	2,267751655	1,97E-06	1,201992162
BSU29310	YtnJ	-1,83739864	0,279825891	6,13E-11	0,30645821	1,236667973	0,034699703	0,758246932
BSU29320	YtnI	-0,8788241	0,543810494	6,62E-06	0,451790103	1,367736298	0,020490067	0,955773396
BSU29330	YtmO	-1,63596163	0,32175587	8,60E-08	0,750414827	1,682276476	0,002033689	1,002016173
BSU29340	TcyN	-2,34738726	0,19650157	8,04E-06	0,873749874	1,832419568	0,000465881	1,014460569
BSU29350	TcyM	-1,50573608	0,352150471	9,51E-08	0,549011135	1,463082513	0,002659182	0,907616492
BSU29360	TcyL	-1,40477302	0,377677561	0,000450479	1,070901959	2,100746323	9,57E-05	1,239211942
BSU29370	TcyK	-0,88574126	0,541209376	0,000728491	0,710347541	1,636198225	0,000660831	1,088703801
BSU29380	TcyJ	-0,63245052	0,645079769	7,82E-05	-0,12489579	0,917070281	0,134528827	0,781075025
BSU29390	YtmI	-2,25396133	0,209647666	0,00020477	0,455258251	1,371028205	0,303228459	0,790337936
BSU29400	YtlI	-2,08572991	0,235576917	6,50E-10	-0,48802907	0,71299849	0,028474148	0,474287704
BSU29410	YtkL	-0,68298891	0,622873498	0,005488466	-0,96035768	0,513929483	0,000174407	0,56840149
BSU29420	YtkK	-1,01967596	0,493227121	3,44E-05	-0,268314	0,830289292	0,286398473	0,661758207
BSU29430	YtzD	-1,0664879	0,477479964	7,66E-05	-0,84446896	0,556915768	0,000184511	0,517197866
BSU29440	ArgH	-0,29172492	0,816924741	0,033253368	-0,49790666	0,708133534	0,144877345	0,762529137
BSU29450	ArgG	-0,59909181	0,66016941	0,000881676	-0,59912051	0,660156276	0,033485709	0,660162843
BSU29460	MoaB	0,623790582	1,540918525	0,230895461	-0,32851027	0,796358379	0,264650186	1,168638452

**BSU29470**	**AckA**	**-1,92753399**	**0,262878127**	**2,65E-11**	**-1,25517885**	**0,418941628**	**3,37E-07**	**0,340909878**
BSU29480	YtxK	0,07231492	1,05140239	0,469671536	-0,30711838	0,808254547	0,046745854	0,929828469
**BSU29490**	**Tpx**	**0,326827119**	**1,254251896**	**0,047287458**	**-0,03252589**	**0,977707018**	**0,343630784**	**1,115979457**
**BSU29500**	**YtfJ**	**-1,38264266**	**0,383515645**	**9,91E-07**	**-1,31882813**	**0,400860418**	**1,40E-07**	**0,392188032**
BSU29510	YtfI	0,521572489	1,435519063	0,01042159	-0,07619604	0,948555416	0,588865595	1,192037239
BSU29520	YteJ	-0,42292451	0,745911044	0,015719681	-0,46374575	0,72510119	0,010959348	0,735506117
BSU29530	SppA	-0,80472098	0,572472787	0,001422412	0,091445349	1,065437045	0,414926631	0,818954916
BSU29540	YtdI	2,295673387	4,90983107	7,93E-13	0,709645229	1,635401908	0,000536115	3,272616489
BSU29550	YtcJ	-0,04634047	0,968389624	0,730049958	0,105235921	1,075670279	0,64825767	1,022029951
BSU29560	YtcI	-0,94593358	0,519093533	6,09E-05	0,529850822	1,443779898	0,002585321	0,981436716
**BSU29570**	**SspA**	**-3,12467636**	**0,114651222**	**1,44E-17**	**-2,82219471**	**0,141395224**	**1,34E-16**	**0,128023223**
BSU29580	YtbJ	-1,29619618	0,407198405	4,16E-07	-0,8176511	0,567364938	0,000475302	0,487281672
BSU29590	NifZ	-1,53539351	0,344985228	6,51E-08	-0,28683039	0,819700973	0,296539947	0,5823431
BSU29600	BraB	-1,03943228	0,486518889	0,000639085	-0,33308816	0,793835421	0,148045854	0,640177155
BSU29610	EzrA	0,611784428	1,528148163	0,000420189	0,371475076	1,293674866	0,087935802	1,410911514
BSU29620	HisJ	0,676573193	1,598338739	0,001764063	0,442923725	1,359356371	0,023047634	1,478847555
BSU29630	YttP	1,100953063	2,144963448	2,04E-06	0,297529351	1,229037854	0,082804807	1,687000651
BSU29640	YtsP	0,498969393	1,413203663	0,005199648	0,022843238	1,015959744	0,801832611	1,214581703
BSU29650	YtrP	0,742752322	1,673365181	0,00251093	0,185473882	1,137190445	0,014982488	1,405277813
BSU29660	RpsD	0,424359631	1,341976707	0,002934492	-0,41991243	0,747469995	0,084718559	1,044723351
BSU29670	TyrS	-1,16149571	0,447048819	3,12E-05	-0,23382986	0,850374442	0,214919695	0,648711631
BSU29679	YtzK	0,902107311	1,868793694	4,06E-05	-0,18423361	0,880116487	0,71408602	1,37445509
BSU29680	AcsA	1,193160541	2,286531099	5,14E-07	0,665307003	1,585905715	0,000212292	1,936218407
BSU29690	AcuA	0,46337506	1,378763546	0,001116051	1,127250512	2,184420365	1,73E-07	1,781591955
BSU29700	AcuB	0,917512705	1,88885598	2,65E-05	0,871463944	1,829518424	7,89E-06	1,859187202
BSU29710	AcuC	1,08445466	2,120573752	3,06E-06	0,864523144	1,820737757	2,61E-05	1,970655754
BSU29720	MotS	-0,42537803	0,744643586	0,237477274	0,260359057	1,197776769	0,135939303	0,971210177
BSU29730	MotP	-0,41801945	0,748451401	0,151635498	0,468728424	1,383889185	0,015628071	1,066170293
BSU29740	CcpA	-0,60278161	0,658483132	0,006580462	-0,47881742	0,717565575	0,043263124	0,688024354

BSU29750	AroA	0,168154089	1,123619907	0,099108068	-0,2574045	0,83659165	0,380893663	0,980105778
BSU29760	YtxJ	0,977757243	1,969401475	5,75E-06	-0,17819085	0,88381061	0,717042224	1,426606042
BSU29770	YtxH	0,423777279	1,341435121	0,066103818	0,049010214	1,034554906	0,340724036	1,187995013
BSU29780	YtxG	0,859286262	1,814140587	7,81E-05	0,464595493	1,379930391	0,00798756	1,597035489
BSU29790	MurC	-0,81823721	0,567134486	0,001780622	-0,28964894	0,818101109	0,287984079	0,692617798
BSU29805	SftA	-0,59895489	0,660232064	0,023882689	0,381165377	1,302393475	0,063022436	0,98131277
BSU29820	YtpR	0,320101183	1,248418103	0,029880344	0,386273477	1,307012988	0,025898545	1,277715546
BSU29830	YtpQ	-0,2922836	0,816608447	0,217363269	0,213791432	1,159731982	0,297245149	0,988170214
BSU29840	YtpP	-0,34372539	0,788003863	0,173141855	0,280980368	1,215020258	0,090228101	1,00151206
BSU29850	YtoQ	-0,62187201	0,649827178	0,007179107	-0,29995924	0,812275347	0,203588968	0,731051262
BSU29860	YtoP	0,30801514	1,238003282	0,046933001	-0,56650902	0,675248754	0,001591674	0,956626018
BSU29870	YtzB	1,260555009	2,395878934	2,42E-08	0,318707703	1,247212855	0,025193354	1,821545895
BSU29880	MalS	-0,81365683	0,568937929	0,000165783	-0,43953724	0,737371091	0,062971574	0,65315451
BSU29890	YtnP	-0,7140704	0,609597799	0,001749185	0,226122578	1,169687042	0,071797761	0,88964242
BSU29900	TrmB	-0,44258164	0,735816719	0,101957289	-0,13507832	0,910620399	0,390082214	0,823218559
**BSU29910**	**YtzH**	**-2,23916258**	**0,211809238**	**1,99E-13**	**-1,33410724**	**0,396637438**	**2,32E-08**	**0,304223338**
BSU29920	YtmP	0,78157575	1,719007393	1,52E-06	0,267053759	1,203347865	0,020193788	1,461177629
BSU29930	AmyX	-0,48122453	0,716369324	0,089004443	-0,36575404	0,776063153	0,088427455	0,746216239
BSU29940	YtlR	-1,0859276	0,471089276	1,68E-06	-0,32040052	0,800847515	0,227358486	0,635968395
BSU29950	YtlQ	-1,45557621	0,364609432	2,23E-07	-0,42269485	0,746029793	0,079913826	0,555319613
BSU29960	YtlP	-1,42111696	0,373423089	8,33E-08	-0,481536	0,716214684	0,029319423	0,544818887
BSU29970	YtkP	-0,06146642	0,958289577	0,350397331	0,062762414	1,044463745	0,64709074	1,001376661
BSU29980	YtjP	-0,65876632	0,633419717	0,02656486	-0,12950559	0,91414467	0,139696213	0,773782193
BSU29990	PbuO	-0,53047592	0,69232631	0,003142517	-0,01563117	0,989223783	0,045368603	0,840775046
BSU30000	YthQ	1,417343858	2,670933132	1,95E-07	0,741027823	1,671366151	0,031994127	2,171149641
BSU30010	YthP	0,896288772	1,861271833	0,002048038	0,445152177	1,361457716	0,073573315	1,611364775
BSU30020	YtzE	-0,24531586	0,843631072	0,036281378	-0,81666128	0,567754333	0,001370382	0,705692702
BSU30035	YtzG	-0,57352583	0,671972534	0,001560671	-0,09588041	0,935701057	0,34034518	0,803836795
BSU30050	YtgP	0,22979562	1,172668811	0,067420466	-0,27005109	0,829290176	0,251431053	1,000979494

BSU30060	YtfP	0,31098056	1,240550581	0,099512281	0,168984381	1,124266752	0,111540393	1,182408667
BSU30070	OpuD	-0,1766054	0,884782402	0,590152416	0,123885372	1,08966553	0,139381727	0,987223966
BSU30080	YteV	2,196198439	4,582701888	6,63E-11	-0,43104292	0,741725401	0,04092233	2,662213645
BSU30090	YteU	-0,75410024	0,592916051	0,004588361	-0,57644132	0,670615941	0,020824695	0,631765996
BSU30100	YteT	-1,50633567	0,352004148	7,13E-07	-0,04929977	0,966405275	0,648991402	0,659204711
BSU30110	YteS	-0,25588942	0,837470673	0,002244129	0,474410106	1,389350022	0,081168553	1,113410348
BSU30120	YteR	-1,06000048	0,479631901	0,022838846	1,120276342	2,173886084	0,001216655	1,326758993
BSU30135	YteP	1,151775812	2,221872162	3,27E-07	0,348825614	1,273523529	0,126796826	1,747697845
BSU30150	YtdP	0,457313815	1,37298305	0,138647129	0,488745794	1,403224453	0,001019436	1,388103752
BSU30160	YtcQ	-0,09772825	0,934503355	0,208192532	0,924016735	1,897390639	1,44E-06	1,415946997
BSU30170	YtcP	-0,10826026	0,927706105	0,358033254	1,276603484	2,42267937	4,01E-08	1,675192738
BSU30180	YtbQ	-0,2349515	0,849713562	0,040147405	0,014278384	1,009946159	0,515744826	0,92982986
BSU30190	BioI	0,24111296	1,181904084	0,148053108	0,190311491	1,141010044	0,124506029	1,161457064
BSU30200	BioB	-0,25272321	0,839310649	0,023181743	0,117794925	1,08507512	0,064209268	0,962192884
BSU30210	BioD	0,163075869	1,119671769	0,096233247	0,505481436	1,419597006	0,000383116	1,269634387
BSU30220	BioF	0,081605694	1,058195138	0,246251026	0,517401193	1,43137451	0,000240607	1,244784824
BSU30230	BioA	-0,11260951	0,924913586	0,047404912	0,339538441	1,265351707	0,001128596	1,095132646
BSU30240	BioW	-0,29350169	0,815919267	0,059899266	0,004685311	1,003252889	0,294042917	0,909586078
BSU30250	YtaP	0,519356586	1,433315874	0,001822666	0,29707479	1,228650673	0,171876286	1,330983274
BSU30260	MsmR	-0,32912651	0,796018294	0,212912582	0,850296557	1,802871481	0,00032261	1,299444887
BSU30270	MsmE	-0,6269935	0,647524416	0,003629468	0,266776901	1,203116961	0,084764406	0,925320689
BSU30280	AmyD	-0,21299043	0,86274707	0,233181074	0,562676829	1,47700717	0,002101234	1,16987712
BSU30290	AmyC	-0,00358159	0,997520512	0,068197302	0,073628745	1,052360311	0,41371577	1,024940412
BSU30300	MelA	-0,84074041	0,558356941	0,000205011	-0,17676583	0,884684021	0,378308999	0,721520481
BSU30310	YtwF	-0,4979953	0,708090026	0,001936215	-0,32865031	0,796281085	0,100764688	0,752185556
BSU30320	LeuS	0,870919301	1,828827878	1,81E-05	0,787225883	1,72575287	0,000861818	1,777290374
BSU30330	YtvB	-0,66134317	0,632289353	0,002695476	-1,6636089	0,315648565	3,00E-11	0,473968959
BSU30340	YtvA	-0,77324479	0,585100036	0,000836823	-0,25595616	0,837431935	0,118315674	0,711265985
BSU30350	YttB	-0,77386691	0,584847782	0,194224701	0,11989346	1,086654612	0,33001835	0,835751197

BSU30360	YttA	-0,05506767	0,962549292	0,027982624	-0,43817518	0,738067578	0,047777283	0,850308435
BSU30370	BceB	-0,12843619	0,914822537	0,356363231	-0,0929905	0,937577268	0,335411939	0,926199903
BSU30380	BceA	-0,06232737	0,957717873	0,090429344	0,863584917	1,819554062	0,000745986	1,388635968
BSU30390	BceS	0,909914741	1,878934455	8,91E-06	0,845324569	1,796668905	0,002215975	1,83780168
BSU30400	BceR	0,759181373	1,692529961	6,95E-05	0,617610774	1,534332091	0,016571741	1,613431026
BSU30410	YtrF	-0,50930037	0,702563061	0,008211835	0,530250863	1,444180295	0,052295299	1,073371678
BSU30420	YtrE	-0,36504796	0,776443062	0,01634985	0,697108534	1,621252202	0,000136943	1,198847632
BSU30430	YtrD	-0,69217889	0,618918396	0,000647607	0,437615665	1,354364126	0,003677606	0,986641261
BSU30440	YtrC	-0,33468355	0,792958052	0,122038188	0,718819987	1,645835318	0,000332143	1,219396685
BSU30450	YtrB	-0,9034331	0,53461303	0,00176384	0,898564609	1,864210288	0,000325574	1,199411659
BSU30460	YtrA	0,043637214	1,0307091	0,153383449	0,905099262	1,872673336	0,00059455	1,451691218
**BSU30470**	**YtzC**	**-1,8812601**	**0,271446521**	**4,49E-13**	**-1,92673029**	**0,263024612**	**6,61E-13**	**0,267235567**
BSU30480	YtqA	-1,3375206	0,395700118	8,60E-07	-0,32741431	0,796963573	0,209184762	0,596331846
BSU30490	YtqB	-0,64069601	0,641403438	0,00131827	-1,12967767	0,457017821	6,46E-06	0,54921063
BSU30500	YtpB	0,346431575	1,271411969	0,038543131	0,284951238	1,218369083	0,124717866	1,244890526
BSU30510	YtpA	-0,22341206	0,856537276	0,288991604	0,274727896	1,209765896	0,221856122	1,033151586
BSU30520	YtoA	-1,15141649	0,450183008	2,36E-06	-1,03262103	0,48882127	3,70E-06	0,469502139
BSU30530	YtnA	1,270245408	2,412025915	3,26E-08	0,678698625	1,600695205	0,000938987	2,00636056
BSU30540	AsnB	0,952370823	1,93504997	0,000175431	0,789047046	1,727932722	2,12E-05	1,831491346
BSU30550	MetK	-0,66934629	0,628791539	0,004971052	-0,54898295	0,683501805	0,054715897	0,656146672
BSU30560	PckA	-0,44587442	0,734139217	0,008370763	-0,48646742	0,713770699	0,246741392	0,723954958
BSU30570	YtmB	-0,20920156	0,865015833	0,095379318	-0,6804855	0,623955266	0,006492269	0,744485549
BSU30580	YtmA	-0,44815012	0,732982104	0,000256426	-0,62624853	0,647858869	0,002422373	0,690420487
**BSU30595**	**YtlA**	**-3,58452162**	**0,083358804**	**7,79E-20**	**-2,76916682**	**0,146689059**	**9,21E-17**	**0,115023931**
**BSU30610**	**YtlC**	**-3,59682277**	**0,082651066**	**6,82E-20**	**-2,58020474**	**0,167217213**	**4,35E-16**	**0,124934139**
**BSU30620**	**YtlD**	**-3,99748102**	**0,062609222**	**3,11E-19**	**-2,86225924**	**0,137522612**	**4,88E-17**	**0,100065917**
BSU30630	MutTA	0,255585897	1,193820471	0,028391141	-0,50492872	0,704695194	0,023959581	0,949257832
BSU30640	YtkC	-0,07564572	0,94891731	0,239313622	0,600912509	1,516675565	0,023224291	1,232796438
BSU30650	Dps	0,553221828	1,467358947	0,002008653	0,408226294	1,327053279	0,051528479	1,397206113

BSU30659	YtzI	0,901610239	1,868149923	1,38E-05	-0,8620998	0,550151248	8,45E-05	1,209150585
BSU30660	YtkA	0,070187056	1,049852796	0,478842783	0,301474069	1,232402974	0,109181022	1,141127885
BSU30670	LuxS	0,720428456	1,647671292	0,000219954	0,138424198	1,100702203	0,384123648	1,374186747
BSU30680	YtjA	0,446903106	1,363111055	0,004772884	-0,32310176	0,799349447	0,079539544	1,081230251
BSU30690	YtiB	0,450603312	1,366611632	0,001293536	0,863402821	1,819324414	6,58E-05	1,592968023
BSU30700	YtiA	0,394542935	1,314526235	0,010179967	-0,35995381	0,779189526	0,084462054	1,04685788
**BSU30710**	**YthA**	**-3,20290885**	**0,108599634**	**5,52E-19**	**-0,96561324**	**0,512060706**	**3,18E-05**	**0,31033017**
**BSU30720**	**YthB**	**-3,14386873**	**0,113136101**	**6,04E-18**	**-1,15896228**	**0,447834543**	**4,01E-06**	**0,280485322**
**BSU30739**	**YtzL**	**-2,12066053**	**0,229941611**	**4,45E-14**	**-2,1960302**	**0,21823733**	**2,23E-13**	**0,224089471**
BSU30740	MntD	-0,81545128	0,568230715	1,02E-05	-0,65618677	0,634553288	0,001483054	0,601392001
BSU30750	MntC	-1,83176191	0,280921333	7,67E-09	-0,07433038	0,949782854	0,570136408	0,615352094
BSU30760	MntB	-2,37922792	0,192212236	3,00E-13	-0,48996916	0,712040318	0,038031502	0,452126277
BSU30770	MntA	-1,56162231	0,33876992	1,68E-07	-0,36667612	0,775567298	0,089262595	0,557168609
BSU30780	MenC	0,656399551	1,576144221	0,007036308	0,587401648	1,502538178	0,022227618	1,539341199
BSU30790	MenE	0,261036553	1,198339382	0,022521666	0,371175509	1,29340627	0,055867245	1,245872826
BSU30800	MenB	-0,77378988	0,584879012	0,004715468	0,232408352	1,174794447	0,091840194	0,87983673
BSU30810	YtxM	-1,10422352	0,465152757	0,068146871	0,168686641	1,124034753	0,087592385	0,794593755
BSU30820	MenD	-0,63153843	0,645487727	0,00365838	-0,03445015	0,976403822	0,362443137	0,810945775
BSU30830	MenF	-0,75735719	0,591579027	0,004279103	-0,050603	0,965532683	0,862101233	0,778555855
**BSU30840**	**YteA**	**-3,25255878**	**0,104925789**	**1,75E-17**	**-2,51640239**	**0,174778255**	**2,33E-15**	**0,139852022**
**BSU30850**	**YtdA**	**-2,34365305**	**0,197010844**	**1,30E-14**	**-0,89987819**	**0,535931979**	**0,000183415**	**0,366471412**
**BSU30860**	**YtcA**	**-2,80836954**	**0,14275671**	**5,61E-13**	**-2,06956431**	**0,238231434**	**5,38E-10**	**0,190494072**
**BSU30870**	**YtcB**	**-4,11987567**	**0,057516685**	**4,75E-20**	**-2,17294726**	**0,221757183**	**6,57E-14**	**0,139636934**
**BSU30880**	**YtcC**	**-4,05635971**	**0,060105478**	**2,62E-15**	**-2,48411502**	**0,178733873**	**1,02E-14**	**0,119419675**
**BSU30890**	**YtxO**	**-2,8122965**	**0,14236866**	**5,80E-11**	**-1,21105798**	**0,431951733**	**1,06E-06**	**0,287160196**
BSU30900	CotS	-0,85542197	0,552703644	1,22E-06	-0,54216058	0,686741676	0,026082631	0,61972266
BSU30910	CotSA	-2,096321	0,233853836	0,00013717	-0,39485893	0,760563742	0,099218477	0,497208789
**BSU30920**	**CotI**	**-3,25778079**	**0,104546684**	**3,88E-19**	**-2,41453573**	**0,187565224**	**4,67E-15**	**0,146055954**
**BSU30930**	**YtaB**	**-1,31168024**	**0,402851424**	**2,61E-06**	**-1,39337199**	**0,380674018**	**2,88E-08**	**0,391762721**

BSU30940	GlgP	-1,40892759	0,376591518	1,33E-06	-0,5562966	0,680045602	0,023903615	0,52831856
BSU30950	GlgA	-1,55035356	0,341426381	8,21E-09	-0,1631491	0,893073548	0,558214539	0,617249964
BSU30960	GlgD	-1,1743778	0,443074807	9,90E-08	-0,2757937	0,825995769	0,887820495	0,634535288
BSU30970	GlgC	-1,3318247	0,397265469	6,06E-09	-0,29433158	0,815450053	0,55885135	0,606357761
BSU30980	GlgB	-1,58781739	0,332674366	1,29E-09	-0,47918541	0,717382564	0,24373829	0,525028465
BSU30990	ThiT	0,612365066	1,528763317	0,000977882	-0,2509694	0,840331578	0,06516563	1,184547447
BSU31000	YuaI	-0,93037121	0,52472331	0,00102019	-0,82261264	0,565417078	0,000645307	0,545070194
BSU31010	FloT	-0,76595633	0,588063427	0,000169046	-0,8955447	0,537544205	0,000576793	0,562803816
BSU31020	YuaF	-1,1960288	0,436475083	7,19E-08	-0,54454129	0,68560936	0,056566918	0,561042222
BSU31030	YuaE	-0,90212943	0,53509634	0,000345851	-1,03982477	0,486386548	9,10E-06	0,510741444
BSU31040	YuaD	1,491280195	2,811383363	6,14E-09	-0,00951165	0,993428714	0,072451752	1,902406039
BSU31050	GbsB	-0,43814621	0,738082401	0,001159616	-1,01408081	0,495143702	6,56E-05	0,616613051
**BSU31060**	**GbsA**	**-1,49672389**	**0,354357161**	**4,20E-09**	**-1,35602122**	**0,390658195**	**1,60E-06**	**0,372507678**
BSU31070	YuaC	0,88108814	1,841763912	0,004305359	-0,14616395	0,903650029	0,534697624	1,372706971
BSU31080	YuaB	-0,55853627	0,678990704	0,000935751	0,049909206	1,035199773	0,501284976	0,857095238
BSU31090	KtrA	-1,9106956	0,26596428	3,40E-07	0,07542088	1,05366838	0,274663021	0,65981633
BSU31100	KtrB	-1,42991799	0,37115199	2,11E-06	-0,14980538	0,90137205	0,413046411	0,63626202
BSU31110	YubF	-0,45219908	0,730927857	0,111751583	-0,36875596	0,774450021	0,056558739	0,752688939
BSU31120	LytG	0,826372782	1,773221527	0,000365164	0,735296103	1,66473911	0,031578617	1,718980319
BSU31130	YubD	-1,01291297	0,495544673	0,055836377	-0,69955814	0,615760769	0,050742311	0,555652721
**BSU31140**	**YubC**	**-1,29359905**	**0,407932104**	**3,66E-06**	**-1,75309889**	**0,296663865**	**3,85E-09**	**0,352297984**
BSU31150	YubB	1,445513299	2,723597095	1,57E-10	0,615551234	1,532143296	0,001600641	2,127870195
BSU31160	YubA	0,413001295	1,331452808	0,015591157	0,732590212	1,661619684	0,000139403	1,496536246
BSU31170	YulF	0,895739937	1,860563896	4,68E-05	0,096417354	1,069115229	0,097725022	1,464839562
BSU31180	YulE	0,601331674	1,517116289	0,003718908	0,829479938	1,777044659	0,00060843	1,647080474
BSU31190	YulD	-0,20070305	0,87012643	0,02626462	0,550336664	1,464427391	0,005020415	1,16727691
BSU31200	YulC	-0,27870166	0,824332533	0,235077608	0,362233416	1,28541429	0,043707106	1,054873412
BSU31210	YulB	-0,64724671	0,638497685	0,004990766	0,649006911	1,56808842	0,003702786	1,103293052
BSU31220	YuxG	0,102787655	1,073846404	2,94E-05	0,730646148	1,659382122	0,003499298	1,366614263

BSU31230	TlpB	0,113291371	1,081693203	0,073902787	-0,25121369	0,840189295	0,387322779	0,960941249
BSU31240	McpA	-0,55401322	0,681122774	0,009942989	-0,48341917	0,715280409	0,079032102	0,698201591
BSU31250	TlpA	-1,32140628	0,400144704	1,27E-06	-0,14736865	0,902895765	0,478216551	0,651520234
BSU31260	McpB	-0,67978024	0,624260357	0,011778812	-0,92233502	0,527654314	0,000481001	0,575957336
**BSU31270**	**Tgl**	**-2,87311686**	**0,136491512**	**2,18E-15**	**-1,7085115**	**0,305975596**	**1,38E-09**	**0,221233554**
BSU31279	YuzH	1,490899854	2,810642291	4,53E-05	0,423305341	1,340996379	0,098565153	2,075819335
BSU31280	YugU	0,89898338	1,864751491	4,71E-05	0,723617058	1,651316955	0,001935146	1,758034223
BSU31290	YugT	-1,29209532	0,408357514	4,57E-07	-0,92791634	0,525616932	4,27E-05	0,466987223
**BSU31300**	**YugS**	**-2,23202978**	**0,212859033**	**3,16E-13**	**-1,14965566**	**0,4507328**	**8,37E-07**	**0,331795916**
**BSU31310**	**YugP**	**-1,4397811**	**0,368623231**	**3,41E-10**	**-1,55124832**	**0,341214693**	**1,03E-09**	**0,354918962**
BSU31319	YuzI	-0,26195928	0,833954584	0,001311061	-1,06626506	0,47755372	6,82E-06	0,655754152
BSU31321	MstX	0,776515164	1,712988142	1,18E-06	-0,13823776	0,908628363	0,233362414	1,310808252
BSU31322	YugO	-0,42555724	0,744551092	0,018329433	-0,33627192	0,792085503	0,113109476	0,768318298
BSU31330	YugN	0,677560888	1,599433364	1,47E-05	0,252567948	1,191325748	0,07912021	1,395379556
BSU31340	YugM	1,242278039	2,365717883	2,07E-09	0,197774538	1,14692777	0,217261108	1,756322826
BSU31350	Pgi	0,679131949	1,601176058	0,000161291	0,846094489	1,797627985	2,92E-05	1,699402021
BSU31360	YugK	-1,44238094	0,367959544	7,76E-07	-0,51168723	0,701401668	0,024253939	0,534680606
BSU31370	YugJ	-1,52372589	0,347786565	1,81E-09	-0,68920683	0,620194731	0,001404722	0,483990648
**BSU31380**	**YuzA**	**-2,14779077**	**0,225657906**	**2,11E-14**	**-2,3303704**	**0,198833065**	**1,12E-13**	**0,212245485**
BSU31390	YugI	-0,09681226	0,935096878	0,631936342	-0,62352174	0,649084525	0,015047599	0,792090702
BSU31400	AlaT	0,973040296	1,962972954	6,54E-06	1,07652176	2,108945423	7,02E-07	2,035959189
BSU31410	AlaR	0,579508347	1,494339909	0,001028113	1,361153518	2,568904962	8,75E-09	2,031622436
**BSU31420**	**YugF**	**-3,05651254**	**0,120198222**	**7,55E-18**	**-1,51131573**	**0,350791153**	**1,73E-09**	**0,235494688**
BSU31430	YugE	0,794171421	1,734081158	0,018919785	-0,51296344	0,700781482	0,024597249	1,21743132
BSU31440	PatB	-0,29755187	0,813631891	0,207502002	0,024105871	1,016849291	0,939497606	0,915240591
BSU31450	KinB	0,304120856	1,234666032	0,08952408	0,604658568	1,520618837	0,001869191	1,377642434
BSU31460	KapB	0,14892262	1,108741174	0,004830573	0,625829555	1,543097857	0,006462926	1,325919515
BSU31470	KapD	-0,53901706	0,688239665	0,050200035	-0,51629887	0,699163186	0,011271824	0,693701425
BSU31480	YuxJ	-0,02308681	0,984124804	0,301732157	0,221390974	1,165857106	0,374520739	1,074990955

BSU31490	PbpD	0,227353847	1,170685738	0,155072704	0,420912949	1,338774474	0,001226207	1,254730106
BSU31500	YuxK	1,730405902	3,318211631	6,68E-13	0,290710979	1,22324296	0,140800316	2,270727295
BSU31510	YufK	0,638327035	1,556523151	0,003102802	-0,24569831	0,843407463	0,20828247	1,199965307
BSU31520	MalK	-0,91171091	0,531554339	0,001189771	-0,2059528	0,866965924	0,151695859	0,699260132
BSU31530	MalR	-0,60633917	0,656861371	0,024426269	-0,56748549	0,674791874	0,010220167	0,665826623
BSU31540	NupN	-0,81208644	0,569557561	0,001697455	-0,35294624	0,782983474	0,265340911	0,676270517
BSU31550	NupO	-0,70317469	0,614219112	0,000268251	0,276948317	1,211629254	0,129668449	0,912924183
BSU31560	NupP	-0,97559364	0,508530551	0,000443742	0,263794154	1,200632107	0,083734174	0,854581329
BSU31570	NupQ	0,120274307	1,086941509	0,021787942	-0,12418464	0,917522448	0,592627197	1,002231978
BSU31580	MaeN	-0,36745828	0,775146936	0,033430602	-0,11808942	0,921407074	0,234732531	0,848277005
BSU31590	YufS	0,154160467	1,112773884	0,003424233	-1,31625405	0,401576276	6,98E-08	0,75717508
BSU31600	MrpA	0,038433809	1,026998311	0,614069431	0,002794557	1,001938916	0,409552284	1,014468614
BSU31610	MrpB	0,196410442	1,14584384	0,192658756	-0,1562847	0,897332954	0,531326326	1,021588397
BSU31620	MrpC	0,105787594	1,076081683	0,277874474	0,047836062	1,033713265	0,596823818	1,054897474
BSU31630	MrpD	0,83400914	1,782632285	9,47E-05	-0,0328244	0,977504735	0,285992123	1,38006851
BSU31640	MrpE	0,518670141	1,432634054	0,005075562	-0,06189857	0,958002569	0,864301395	1,195318312
BSU31650	MrpF	1,332437045	2,518277109	3,75E-08	-0,23678653	0,848633463	0,306238635	1,683455286
BSU31660	MrpG	1,637425006	3,111100512	3,15E-11	-0,4333949	0,740517173	0,035106484	1,925808843
BSU31670	YuxO	0,234994505	1,176902254	0,019090794	0,495921162	1,410220895	0,008066453	1,293561574
BSU31680	ComA	-0,01276863	0,991188513	0,448573501	0,498306528	1,412554497	0,005072663	1,201871505
BSU31690	ComP	-0,50799598	0,703198561	0,003867489	0,299283778	1,230533367	0,105418896	0,966865964
BSU31700	ComX	-0,9462157	0,518992033	2,65E-05	0,103165684	1,074127821	0,46948736	0,796559927
BSU31710	ComQ	-0,59349282	0,662736453	0,002585968	-0,33396615	0,793352456	0,381648105	0,728044454
BSU31720	DegQ	-1,03011788	0,489670137	0,000117363	-0,68235578	0,623146904	0,00167267	0,556408521
**BSU31730**	**YuzC**	**-1,47611587**	**0,359455262**	**3,65E-08**	**-1,37931059**	**0,384402444**	**3,89E-08**	**0,371928853**
BSU31740	YuxH	0,139063619	1,101190156	0,179794115	-1,00102828	0,499643751	5,53E-05	0,800416954
BSU31750	PncB	-0,41287219	0,751126502	0,008522126	0,118924245	1,085924834	0,318771611	0,918525668
BSU31760	PncA	-0,49974848	0,707230068	0,000790899	0,328917875	1,256070878	0,026296542	0,981650473
BSU31770	YueI	0,239820784	1,180845964	0,003855721	0,244934725	1,185039152	0,189225834	1,182942558

BSU31780	YueH	-0,30213604	0,811050671	0,126865196	0,415067709	1,333361253	0,030554149	1,072205962
BSU31790	YueG	-0,29664988	0,814140739	0,172711751	-0,12280384	0,918401025	0,408889889	0,866270882
BSU31800	YueF	0,19070965	1,141324986	0,02441832	0,070602317	1,050155026	0,366582366	1,095740006
BSU31810	YuzE	-0,86788246	0,547950524	0,008236085	-0,58218365	0,667952004	0,031403953	0,607951264
BSU31820	YuzF	-0,78508186	0,58031903	0,00150208	-1,01591989	0,494512917	8,54E-06	0,537415973
BSU31830	YueE	-0,13400341	0,911299129	0,432136438	-0,42697041	0,743822135	0,01015918	0,827560632
BSU31840	YueD	0,068782892	1,048831479	0,616764345	0,347518953	1,27237061	0,022944104	1,160601044
BSU31850	YueC	-0,24607029	0,843190026	0,220288158	0,875474725	1,834611674	4,56E-05	1,33890085
BSU31860	YueB	0,116057794	1,083769382	0,147337684	0,88547644	1,847374604	4,49E-06	1,465571993
BSU31875	YukB	-0,83814962	0,559360538	0,000182303	0,594390144	1,509834214	0,001273765	1,034597376
BSU31890	YukC	-0,94914147	0,517940592	0,000499554	0,174379162	1,12847868	0,276151481	0,823209636
BSU31900	YukD	-1,55152201	0,34114997	1,20E-09	0,175299799	1,129199033	0,31878481	0,735174502
BSU31910	YukE	-0,53341673	0,690916499	0,015321233	-0,0805365	0,945705901	0,420922715	0,8183112
BSU31920	YukF	-0,27327722	0,827437804	0,249565048	-0,30108739	0,811640413	0,130073806	0,819539108
**BSU31930**	**Ald**	**-1,09382379**	**0,468517944**	**1,93E-06**	**-1,67829437**	**0,312451816**	**8,96E-09**	**0,39048488**
BSU31945	YukJ	0,899967323	1,866023717	2,04E-05	0,10667051	1,076740436	0,459361808	1,471382077
BSU31959	YbdZ	1,258294298	2,392127518	5,63E-09	1,043852508	2,061725849	8,39E-07	2,226926683
BSU31960	DhbF	1,009007562	2,012526196	0,000130894	1,421342669	2,678346599	9,79E-11	2,345436398
BSU31970	DhbB	0,309309806	1,239114759	0,230910788	1,553330485	2,934938932	9,56E-12	2,087026845
BSU31980	DhbE	0,453841945	1,369682916	0,008953864	1,623773593	3,081800758	5,78E-12	2,225741837
BSU31990	DhbC	0,351923652	1,276261227	0,026194612	1,447916344	2,728137471	1,80E-10	2,002199349
BSU32000	DhbA	-0,06521884	0,955800323	0,524172852	1,474627294	2,779118389	1,03E-10	1,867459356
BSU32010	BesA	-0,10728901	0,928330866	0,498117744	0,564633551	1,479011788	1,49E-05	1,203671327
BSU32020	YuiH	-1,14013648	0,453716655	0,000342683	-0,60354177	0,658136268	0,00110937	0,555926462
BSU32030	YuiG	0,808812093	1,751768453	1,36E-05	0,017061351	1,011896231	0,205964171	1,381832342
BSU32040	YuiF	-0,92819298	0,525516157	0,03869394	0,401006869	1,320429128	0,147942132	0,922972642
BSU32050	YuiE	-0,21978452	0,858693681	0,129238442	0,099701624	1,071551823	0,3910698	0,965122752
BSU32060	YuiD	0,641793439	1,56026755	6,12E-05	-1,08107545	0,472676338	7,21E-06	1,016471944
BSU32070	YuiC	-0,35400357	0,782409845	0,094044284	-0,51822392	0,698230884	0,025579582	0,740320365

BSU32080	YuiB	0,599007894	1,514674604	5,07E-05	-0,49735164	0,708406014	0,117014288	1,111540309
BSU32090	YuiA	0,265775465	1,202282117	0,136917425	-0,48332669	0,715326259	0,575632876	0,958804188
BSU32100	YumB	-1,00091449	0,499683162	4,13E-07	-1,09134398	0,469323959	1,03E-06	0,484503561
BSU32110	YumC	0,025901139	1,01811543	0,615892302	-0,23072714	0,852205257	0,271797473	0,935160344
BSU32120	YuzG	0,255180547	1,193485093	0,062167148	-1,04482532	0,484703591	5,78E-06	0,839094342
BSU32130	GuaC	-1,84156012	0,279019891	1,16E-06	0,154016873	1,112663134	0,366142726	0,695841513
BSU32140	PaiB	-1,03004554	0,489694691	0,000106714	0,072308308	1,051397572	0,634870758	0,770546131
BSU32150	PaiA	-0,24778467	0,842188648	0,12717281	0,690853953	1,614238728	0,011619647	1,228213688
BSU32160	SufA	0,978628205	1,97059077	1,36E-05	-0,21420825	0,862019105	0,514867395	1,416304937
BSU32170	DapF	-0,30625679	0,808737387	0,329413577	-0,06045675	0,958960469	0,015693761	0,883848928
BSU32180	YutK	-0,96465171	0,512402099	0,006458687	-1,49300429	0,355271954	3,47E-05	0,433837027
BSU32190	YuzB	1,934345374	3,822046614	2,44E-10	0,21983998	1,164604404	0,426284129	2,493325509
BSU32200	YutJ	0,194109708	1,144017965	0,00386896	-0,20507704	0,867492362	0,33651069	1,005755163
BSU32210	YuzD	0,230201012	1,172998373	0,05608967	0,105788163	1,076082107	0,604372365	1,12454024
BSU32220	YutI	0,724916382	1,652804837	3,00E-05	-0,50967689	0,702379729	0,021802446	1,177592283
BSU32230	YuxL	-0,61738402	0,651851834	0,012426507	-0,27719389	0,825194498	0,132633021	0,738523166
BSU32240	ThrB	1,204379729	2,304381738	1,41E-05	0,614394654	1,5309155	0,001153727	1,917648619
BSU32250	ThrC	-0,31954728	0,801321295	0,165388987	0,354591802	1,278623754	0,005011719	1,039972525
BSU32260	Hom	-0,48671187	0,713649765	0,01216653	0,257812398	1,195664307	0,01760876	0,954657036
BSU32270	YutH	-1,57382653	0,335916245	6,96E-10	-0,43801114	0,738151505	0,064541835	0,537033875
BSU32280	YutG	-0,93292207	0,523796358	0,000409184	-1,06021805	0,479559574	8,64E-06	0,501677966
BSU32290	YutF	-0,00602385	0,99583329	0,05810009	0,564037977	1,478401347	0,037252528	1,237117319
BSU32300	YutE	0,116613428	1,084186861	0,013978814	0,409049806	1,327810998	0,018558133	1,205998929
BSU32310	YutD	-0,0138704	0,990431839	0,77757887	0,454115404	1,36994256	0,031773867	1,180187199
**BSU32320**	**YutC**	**-2,32464895**	**0,199623165**	**1,37E-14**	**-2,42711394**	**0,185937035**	**5,21E-15**	**0,1927801**
BSU32330	LipA	0,74247433	1,673042774	0,000106459	0,341536354	1,267105241	0,104145494	1,470074007
**BSU32340**	**LytH**	**-2,3347893**	**0,198224981**	**1,72E-11**	**-1,91513574**	**0,265146986**	**1,76E-12**	**0,231685984**
BSU32350	YunB	-0,05320451	0,963793174	0,022798141	-0,73343381	0,601470628	0,000771146	0,782631901
BSU32360	YunC	-0,22058986	0,858214476	0,119843218	0,392312127	1,312495183	0,133945582	1,085354829

BSU32370	YunD	-0,02733026	0,981234415	0,026659958	0,566044598	1,480459063	0,003353916	1,230846739
BSU32380	YunE	-0,14321622	0,905498263	0,073980767	0,028895718	1,020230912	0,555694067	0,962864587
BSU32390	YunF	0,298575936	1,229929768	0,047162867	0,248085737	1,187630243	0,116980549	1,208780005
BSU32400	YunG	-0,76753607	0,587419851	0,003446556	0,153636395	1,112369732	0,53573417	0,849894792
BSU32410	PucH	-1,32383595	0,399471381	2,57E-06	-1,09549983	0,467973965	4,15E-06	0,433722673
BSU32420	PucR	-1,71420065	0,304771383	1,70E-09	-0,41856224	0,748169864	0,02700356	0,526470623
BSU32430	PucJ	-1,41066906	0,376137209	6,40E-09	-0,98120233	0,506557403	0,000320312	0,441347306
BSU32440	PucK	-1,30894033	0,403617232	7,48E-07	-0,78985778	0,57840111	0,000124953	0,491009171
BSU32450	PucL	-1,3775146	0,384881279	1,04E-06	-0,91871879	0,52897858	3,23E-05	0,456929929
BSU32460	PucM	0,291909745	1,2242598	0,001089984	-0,64661597	0,638776892	0,004667607	0,931518346
BSU32469	YuzJ	0,138571853	1,100814862	0,350642898	-2,00820834	0,248581643	6,50E-06	0,674698252
BSU32470	PucE	-0,9069216	0,533321873	0,265321244	0,080935539	1,057703703	0,288541023	0,795512788
BSU32480	PucD	-0,091358	0,938638798	0,021645118	-0,13412194	0,91122426	0,408791475	0,924931529
BSU32490	PucC	-2,03179953	0,244549847	0,013187641	0,417009025	1,335156656	0,143755459	0,789853251
BSU32500	PucB	-1,32864107	0,398143091	0,128858947	0,032043087	1,022459067	0,562471689	0,710301079
BSU32510	PucA	-0,95479526	0,515914801	0,049020588	-0,16416048	0,892447691	0,277327588	0,704181246
BSU32520	PucG	-1,13173709	0,456365901	4,38E-06	0,42147909	1,339299938	0,052462408	0,89783292
BSU32530	PucF	-1,62398412	0,324438263	4,58E-06	-0,47128895	0,721319862	0,058091199	0,522879063
BSU32540	YurI	-1,21399387	0,431073604	8,22E-06	-0,55925874	0,678650767	0,020556945	0,554862186
**BSU32550**	**YurJ**	**1,042221106**	**2,059395764**	**2,30E-06**	**1,844444392**	**3,59114621**	**4,26E-12**	**2,825270987**
BSU32560	FrlR	0,567109641	1,481552388	0,001612043	-0,19495968	0,873597311	0,292374251	1,17757485
BSU32570	FrlD	0,76171929	1,695509996	0,000147394	1,994131752	3,983762808	4,63E-12	2,839636402
**BSU32580**	**FrlM**	**0,991777954**	**1,988634241**	**2,34E-06**	**1,990783936**	**3,974529088**	**1,30E-13**	**2,981581665**
**BSU32590**	**FrlN**	**1,113967455**	**2,164400463**	**4,79E-08**	**1,815943054**	**3,520897075**	**2,22E-12**	**2,842648769**
**BSU32600**	**FrlO**	**0,906714087**	**1,874770614**	**6,92E-06**	**1,764466494**	**3,39748338**	**2,22E-12**	**2,636126997**
**BSU32610**	**FrlB**	**1,165810123**	**2,243591657**	**6,91E-08**	**1,66026228**	**3,160739813**	**8,23E-12**	**2,702165735**
BSU32620	YurQ	-1,71908763	0,303740749	2,26E-09	-0,92302916	0,527400499	4,16E-05	0,415570624
BSU32630	YurR	-1,01742694	0,493996614	6,43E-05	0,32835458	1,255580545	0,070291807	0,87478858
BSU32640	SspG	0,277210692	1,211849627	0,015189451	0,788016248	1,726698564	0,00024253	1,469274095

BSU32650	YurS	0,319687157	1,248059882	0,027706895	0,828885279	1,776312336	0,000150667	1,512186109
BSU32660	YurT	0,29239959	1,22467555	0,002835269	0,238788611	1,180001432	0,38621811	1,202338491
BSU32669	YuzN	0,09741867	1,069857515	0,357137637	0,593585618	1,508992482	0,029154374	1,289424999
BSU32670	SufB	-0,78140513	0,581799866	0,001281397	-0,40423256	0,755638149	0,27259803	0,668719007
BSU32680	SufU	-0,64488359	0,639544394	0,000739741	0,122668001	1,088746439	0,186537671	0,864145417
BSU32690	SufS	-1,00826507	0,497143733	8,35E-07	0,200770119	1,1493117	0,076042213	0,823227717
BSU32700	SufD	-1,02175822	0,492515756	7,27E-07	0,106225831	1,076408607	0,140338507	0,784462181
BSU32710	SufC	-0,91853855	0,529044671	0,000152401	-0,21727625	0,860187902	0,789275059	0,694616286
BSU32719	YuzK	-0,40422185	0,755643756	0,053500663	-0,91445659	0,53054367	0,000207741	0,643093713
**BSU32720**	**YurZ**	**-2,8948104**	**0,134454468**	**3,45E-17**	**-1,52832736**	**0,346679068**	**2,76E-09**	**0,240566768**
BSU32730	MetQ	-2,03383411	0,244205212	1,15E-12	-0,5856747	0,666337642	0,000431802	0,455271427
BSU32740	MetP	-1,98877098	0,251953434	3,49E-11	-0,03303398	0,977362744	0,812558499	0,614658089
BSU32750	MetN	-1,81191029	0,284813554	7,31E-11	0,120721481	1,087278466	0,207065508	0,68604601
BSU32760	YusD	0,88524559	1,847079023	1,17E-05	-0,12824351	0,914944722	0,514880581	1,381011872
BSU32770	YusE	0,128228029	1,092950475	0,102720282	-0,10794652	0,927907871	0,802658788	1,010429173
BSU32780	YusF	-0,07179627	0,951452627	0,399926894	0,284145113	1,217688493	0,157682929	1,08457056
BSU32790	YusG	0,068168017	1,048384564	0,161179752	-0,29619941	0,81439499	0,070974305	0,931389777
BSU32800	GcvH	0,443735005	1,360121002	0,048040084	0,480878933	1,395593645	0,010553795	1,377857323
BSU32810	YusI	0,136545418	1,099269723	0,00043894	0,389335658	1,309790124	0,039586278	1,204529924
BSU32820	FadE	0,780122438	1,717276608	0,000263212	-0,02987384	0,979505952	0,676743783	1,34839128
BSU32830	FadA	0,947085818	1,927974297	5,94E-06	0,202740997	1,150882858	0,058877914	1,539428577
BSU32840	FadN	0,855070176	1,808846739	0,000142523	0,285279082	1,218645982	0,026415369	1,513746361
**BSU32849**	**YuzL**	**-1,71569057**	**0,304456798**	**6,13E-11**	**-1,9154892**	**0,265082033**	**3,71E-12**	**0,284769416**
**BSU32850**	**FadM**	**-1,79157235**	**0,288857058**	**2,14E-10**	**-1,09572596**	**0,467900618**	**2,02E-06**	**0,378378838**
**BSU32859**	**YuzM**	**-2,70819611**	**0,153021247**	**4,14E-17**	**-2,16768772**	**0,222567104**	**9,18E-14**	**0,187794176**
**BSU32860**	**YusN**	**-2,78381948**	**0,145206759**	**7,22E-16**	**-2,2703924**	**0,207273502**	**1,86E-14**	**0,176240131**
BSU32870	MdtR	-2,49078542	0,177909392	1,35E-11	-0,54211389	0,6867639	0,0116152	0,432336646
BSU32880	MdtP	-1,23007063	0,426296575	2,26E-06	-0,41265189	0,751241208	0,066544615	0,588768892
BSU32890	YusQ	0,257402984	1,195325045	0,036481712	0,260644257	1,198013576	0,331909056	1,19666931

BSU32900	YusR	-1,30859301	0,40371441	0,030234739	0,377761369	1,299324128	0,254978656	0,851519269
BSU32910	YusS	-1,17215632	0,443757583	4,24E-05	-0,20150666	0,86964189	0,09645097	0,656699736
BSU32920	YusT	0,931376236	1,907094371	0,020418486	-0,54937673	0,683315267	0,041228909	1,295204819
BSU32930	YusU	0,220001334	1,164734663	0,112821883	-0,35725714	0,780647342	0,111993675	0,972691003
BSU32940	YusV	0,900911407	1,867245223	0,001184585	-0,52188744	0,696460075	0,068920847	1,281852649
BSU32950	YusW	-0,96654419	0,511730386	0,000940955	-0,57465341	0,671447538	0,006975284	0,591588962
BSU32980	YusZ	-0,88255362	0,542406502	0,000139141	-0,07706677	0,947983093	0,125775287	0,745194797
BSU32990	MrgA	-0,23290475	0,850919906	0,101198024	-0,06223883	0,957776648	0,639565578	0,904348277
BSU33000	HtrB	-0,07956106	0,94634553	0,143806059	-0,25533654	0,837791678	0,237303356	0,892068604
BSU33010	CssR	-0,11604764	0,922712025	0,575150636	0,612390221	1,528789973	0,028936733	1,225750999
BSU33020	CssS	0,443106631	1,359528722	0,015477682	0,637913134	1,556076657	0,014556007	1,45780269
BSU33029	YirB	0,260362916	1,197779973	0,115381201	0,417605342	1,335708637	0,041387382	1,266744305
BSU33030	YuxN	0,076896215	1,054746437	0,042367378	-0,39733232	0,759260933	0,047276738	0,907003685
BSU33040	CitG	0,80241318	1,744015884	1,60E-05	0,475240531	1,390149971	0,013180091	1,567082927
BSU33049	YvzF	-0,6026028	0,658564749	0,005349021	0,553665241	1,467810009	0,009567511	1,063187379
**BSU33050**	**GerAA**	**-2,83321411**	**0,140319351**	**4,54E-17**	**-1,58515346**	**0,333289214**	**8,86E-10**	**0,236804283**
**BSU33060**	**GerAB**	**-2,04377528**	**0,242528252**	**2,90E-09**	**-1,18826039**	**0,438831688**	**5,17E-07**	**0,34067997**
**BSU33070**	**GerAC**	**-1,6440826**	**0,319949784**	**6,16E-11**	**-1,37976577**	**0,384281182**	**1,63E-07**	**0,352115483**
**BSU33080**	**LiaR**	**1,582459326**	**2,994799308**	**7,91E-10**	**1,185281069**	**2,274076944**	**4,37E-08**	**2,634438126**
**BSU33090**	**LiaS**	**2,418924022**	**5,347720342**	**8,20E-13**	**1,772592298**	**3,416673284**	**3,23E-12**	**4,382196813**
**BSU33100**	**LiaF**	**1,483892379**	**2,797023507**	**5,03E-10**	**1,863385038**	**3,638603976**	**9,26E-13**	**3,217813742**
**BSU33110**	**LiaG**	**2,081377437**	**4,232110911**	**1,06E-13**	**1,873361818**	**3,663853499**	**7,12E-13**	**3,947982205**
**BSU33120**	**LiaH**	**2,447311717**	**5,45398872**	**2,75E-15**	**2,002116439**	**4,005872321**	**1,21E-13**	**4,729930521**
**BSU33130**	**LiaI**	**2,599721907**	**6,061697704**	**1,72E-16**	**2,081304885**	**4,231898088**	**3,56E-14**	**5,146797896**
BSU33140	YvqJ	0,290392842	1,222973245	0,048506282	-0,54177259	0,686926388	0,024903481	0,954949816
BSU33150	YvqK	-0,46360532	0,725171775	6,93E-08	-0,37570189	0,770730349	0,047442843	0,747951062
BSU33160	YvrA	-1,32437958	0,399320881	3,01E-08	0,109070434	1,078533086	0,362476024	0,738926983
BSU33170	YvrB	-0,22152381	0,857659076	0,04144281	-0,1595509	0,895303726	0,449412298	0,876481401
BSU33180	YvrC	-0,18945459	0,876937185	0,411503931	0,253564041	1,19214857	0,073645597	1,034542877

BSU33190	YvrD	-1,27897964	0,412086858	1,11E-07	-0,89026329	0,539515647	0,000115036	0,475801253
BSU33200	YvrE	-1,47557755	0,359589413	1,15E-09	-0,61999007	0,650675408	0,012910917	0,505132411
BSU33210	YvrG	0,704805918	1,629925381	0,008100256	0,610729051	1,527030683	0,006409795	1,578478032
BSU33221	YvrHb	-0,40602738	0,754698664	0,052633873	1,064196302	2,091004696	8,24E-05	1,42285168
BSU33222	YvrHa	-0,50983758	0,702301499	0,47456159	-0,15728697	0,896709774	0,617782984	0,799505637
BSU33230	YvrI	0,969460004	1,958107544	0,004684579	0,058737629	1,041553993	0,522403177	1,499830769
BSU33239	YvrJ	0,01129544	1,007860132	0,293669277	0,069031356	1,049012127	0,769168756	1,02843613
BSU33240	OxdC	-0,0720895	0,951259262	0,002027731	-0,81420257	0,568722753	0,000658862	0,759991008
BSU33250	YvrL	0,103071104	1,074057406	0,476724418	-1,15087884	0,450350809	0,31272937	0,762204107
BSU33260	YvrN	0,984176471	1,97818379	8,64E-05	0,516359402	1,430341267	0,004052145	1,704262528
BSU33270	YvrO	0,608496863	1,524669835	0,000553906	0,838549045	1,788250748	7,77E-05	1,656460291
BSU33280	YvrP	0,346811728	1,271747033	0,004316609	0,789142844	1,728047464	0,0007798	1,499897248
BSU33290	FhuC	0,447744319	1,363906096	0,00022469	0,215530105	1,161130483	0,059527156	1,262518289
BSU33300	FhuG	0,300991282	1,231990629	0,044969177	0,264682097	1,201371294	0,140803467	1,216680961
BSU33310	FhuB	0,644447786	1,563140856	5,13E-06	0,077711292	1,055342504	0,420541938	1,30924168
BSU33320	FhuD	0,034175626	1,023971546	0,147247748	-0,02732694	0,981236671	0,730449618	1,002604109
**BSU33330**	**YvsH**	**-1,57746885**	**0,335069238**	**1,66E-10**	**-1,95884176**	**0,257234889**	**5,63E-12**	**0,296152064**
**BSU33340**	**SspJ**	**-2,03890794**	**0,243347871**	**1,27E-12**	**-3,12015271**	**0,115011282**	**2,29E-18**	**0,179179577**
BSU33350	YvsG	-1,10628865	0,464487395	3,25E-09	-0,6640852	0,631088744	0,00704536	0,54778807
BSU33360	YvgJ	-0,50154323	0,706350803	0,020346023	-0,69646331	0,6170831	0,000171531	0,661716951
BSU33370	YvgK	0,477613043	1,392437954	0,001641795	-0,00111355	0,999228446	0,555424936	1,1958332
BSU33380	YvgL	0,472690502	1,387694988	0,017168164	-0,33715795	0,791599196	0,25040311	1,089647092
BSU33390	YvgM	1,26403383	2,401663171	0,000236346	-0,29533518	0,814882992	0,387327879	1,608273081
BSU33400	YvgN	-0,11245488	0,925012726	0,501575237	-0,34674973	0,786353692	0,074589603	0,855683209
BSU33410	YvgO	2,828263119	7,10218588	2,30E-17	0,677297369	1,599141243	0,001816432	4,350663561
BSU33420	NhaK	-1,66249722	0,315891885	2,98E-08	-0,58863697	0,664970861	0,007300035	0,490431373
BSU33430	CysI	-0,92818968	0,525517356	0,000576789	0,208448064	1,15544458	0,148491652	0,840480968
BSU33440	CysJ	-0,90262438	0,534912796	9,78E-05	0,172425319	1,126951414	0,276639203	0,830932105
BSU33450	HelD	-0,74372272	0,59719636	4,75E-06	-0,73278902	0,601739504	0,000236339	0,599467932

BSU33460	YvgT	-0,21671196	0,860524423	0,615793691	-1,20791693	0,432893208	3,76E-08	0,646708816
BSU33470	BdbC	1,237316174	2,357595438	1,87E-09	0,548304581	1,46236615	0,001469422	1,909980794
BSU33480	BdbD	0,822841806	1,768886898	3,81E-06	0,547475931	1,461526443	0,004611346	1,61520667
BSU33490	CadA	-2,36451918	0,194181925	5,93E-14	-0,6993945	0,615830617	0,005485865	0,405006271
BSU33500	CopA	-0,93404376	0,523389265	1,68E-05	-0,53838266	0,688542372	0,026333255	0,605965818
BSU33510	CopZ	0,535937418	1,449883937	0,003462128	0,322068515	1,250121671	0,110593501	1,350002804
BSU33520	CsoR	0,391002029	1,311303859	0,056069282	0,829163567	1,77665501	0,001405019	1,543979434
BSU33530	IolW	0,164987386	1,121156275	0,259894664	0,222288496	1,166582628	0,120931495	1,143869452
BSU33540	AzoR2	-1,09286062	0,46883084	4,59E-06	-0,64525014	0,639381921	0,004739394	0,554106381
**BSU33550**	**YvaC**	**-2,25902924**	**0,208912506**	**4,80E-14**	**-1,10055335**	**0,466337595**	**3,18E-06**	**0,33762505**
BSU33560	YvaD	1,553685727	2,935661706	3,01E-09	0,736864519	1,666549904	0,004416336	2,301105805
BSU33570	YvaE	1,327074546	2,508934046	6,71E-09	0,517257634	1,431232084	0,013373128	1,970083065
BSU33580	YvaF	0,991860755	1,98874838	7,64E-05	0,929062421	1,904038197	0,002932045	1,946393288
BSU33590	YvaG	-0,41033457	0,752448856	0,1568115	-0,14259253	0,905889802	0,669281346	0,829169329
BSU33600	SmpB	-0,40279859	0,756389586	0,129919021	-0,4458081	0,734172966	0,035216606	0,745281276
BSU33610	Rnr	-1,05420615	0,48156213	2,14E-06	-0,38837491	0,763989701	0,115950033	0,622775916
BSU33620	YvaK	-0,99124972	0,503041831	3,41E-06	-0,19383493	0,874278649	0,478311114	0,68866024
BSU33630	SecG	1,858295233	3,625789659	1,28E-11	-0,40894406	0,753174435	0,158150812	2,189482047
**BSU33640**	**YvaM**	**-2,24860052**	**0,21042813**	**1,30E-13**	**-1,70926017**	**0,305816856**	**2,05E-11**	**0,258122493**
BSU33650	YvzC	1,119302838	2,172419681	9,61E-09	0,455477271	1,371236361	0,01291189	1,771828021
BSU33660	RghR	0,349568781	1,274179721	0,013832415	-0,66743012	0,629627247	0,007532438	0,951903484
BSU33670	YvaO	0,101413146	1,072823798	0,353953443	-0,81262805	0,56934378	0,003363201	0,821083789
BSU33680	CatR	0,352470717	1,276745272	0,160623698	-0,05497073	0,962613973	0,27324944	1,119679622
BSU33690	YvaQ	-0,60935428	0,655490019	7,24E-05	-1,13143753	0,456460673	1,86E-06	0,555975346
BSU33700	OpuBD	0,73900818	1,669028025	6,59E-05	0,259348768	1,196938285	0,097860018	1,432983155
BSU33710	OpuBC	0,887631038	1,850135632	1,64E-05	0,435662888	1,35253215	0,015831053	1,601333891
BSU33720	OpuBB	0,546421623	1,460458763	0,000582897	0,441176541	1,357711111	0,008786057	1,409084937
BSU33730	OpuBA	0,636073907	1,55409415	3,05E-05	0,226391828	1,169905361	0,204318944	1,361999755
BSU33740	YvaV	-1,07350916	0,475161827	0,000128865	-0,64995476	0,637300299	0,003903155	0,556231063

BSU33750	SdpA	0,238911027	1,180101563	0,234647653	0,874426382	1,833279026	4,37E-05	1,506690294
BSU33760	SdpB	-0,11159143	0,925566514	0,514044912	1,189021954	2,279981241	1,95E-07	1,602773877
BSU33770	SdpC	1,160525684	2,235388651	5,75E-07	0,689274405	1,612472331	0,000235319	1,923930491
BSU33780	SdpI	0,862741874	1,818491111	2,16E-06	0,175386023	1,129266523	0,144708815	1,473878817
BSU33790	SdpR	0,136265618	1,099056549	0,382290864	0,37528127	1,297092413	0,027175353	1,198074481
BSU33800	OpuCD	1,487032878	2,803118779	1,75E-10	0,799907444	1,74098943	0,000352791	2,272054105
BSU33810	OpuCC	0,883838155	1,845277966	1,67E-06	0,868404974	1,825643375	2,03E-05	1,835460671
BSU33820	OpuCB	0,76396964	1,698156756	4,38E-06	0,986692376	1,981636539	4,61E-06	1,839896648
BSU33830	OpuCA	1,096697425	2,138645593	1,74E-07	0,724871693	1,652753641	0,000482267	1,895699617
BSU33840	YvbF	0,608630752	1,524811338	2,84E-05	0,871208122	1,829194038	9,79E-05	1,677002688
BSU33850	YvbG	-0,1055471	0,929452412	0,392168899	-0,0244774	0,983176676	0,37979855	0,956314544
BSU33860	YvbH	0,729071811	1,657572314	0,000394832	0,879137041	1,839274796	8,72E-05	1,748423555
BSU33870	YvbI	1,523636754	2,875149061	1,81E-09	-0,16520888	0,891799393	0,478021498	1,883474227
BSU33880	YvbJ	0,600550972	1,516295536	0,018281974	0,178542925	1,131740287	0,139136524	1,324017911
BSU33890	YvbK	0,661299736	1,581506774	0,00094051	0,683968364	1,606552764	0,010086973	1,594029769
BSU33900	Eno	-0,24954482	0,841161766	0,04204926	-0,27701227	0,825298388	0,240055589	0,833230077
BSU33910	Pgm	-0,6490067	0,637719232	0,000649615	0,244723215	1,184865429	0,030033048	0,911292331
BSU33920	Tpi	-0,78035001	0,582225521	0,00021801	0,476025674	1,390906724	0,003477711	0,986566123
BSU33930	Pgk	-0,5296255	0,692734531	0,002360759	0,46716029	1,382385787	0,001231363	1,037560159
BSU33940	GapA	-1,17157414	0,443936691	1,25E-06	-0,13775764	0,908930795	0,626018355	0,676433743
BSU33950	CggR	-1,44167234	0,368140317	2,58E-08	-0,19480734	0,873689565	0,467210621	0,620914941
BSU33960	AraE	1,041173251	2,057900532	2,53E-05	0,267947951	1,204093939	0,178925231	1,630997235
BSU33970	AraR	-0,30820262	0,807647341	0,377470354	-0,52242931	0,696198538	0,032825426	0,751922939
BSU33980	YvbT	-1,04291941	0,485344346	2,86E-06	-0,51856812	0,698064322	0,027969352	0,591704334
BSU33990	YvbU	-0,67974918	0,624273798	0,025141889	0,370124351	1,292464228	0,015492428	0,958369013
BSU34000	YvbV	-1,38514969	0,382849773	1,27E-06	-0,63827176	0,642482134	0,003212992	0,512665954
BSU34010	YvbW	-0,56730493	0,674876333	0,363089423	0,115344872	1,083233959	0,63655945	0,879055146
BSU34020	YvbX	-0,21398731	0,862151131	0,110956206	0,228979447	1,172005587	0,213997495	1,017078359
BSU34030	LutC	0,564822597	1,479205606	0,026329938	-0,22719217	0,854295938	0,62494375	1,166750772

BSU34040	LutB	0,345181881	1,270311121	0,123021351	0,372789948	1,294854459	0,003731903	1,28258279
BSU34050	LutA	1,207702396	2,309695068	9,13E-06	0,03000473	1,021015473	0,098018471	1,665355271
BSU34060	YvfU	1,05687667	2,080422679	0,00015271	0,56367407	1,478028482	0,035845904	1,77922558
BSU34070	YvfT	-0,45654869	0,728727484	0,10439479	0,296037108	1,227767263	0,240693024	0,978247373
BSU34080	YvfS	-1,32615435	0,398829947	0,001672828	-0,41885669	0,748017179	0,048612559	0,573423563
BSU34090	YvfR	-0,78590958	0,579986177	1,99E-05	-0,78108847	0,581927583	0,000337666	0,58095688
BSU34100	RsbQ	0,719204723	1,646274286	0,000796251	0,764806253	1,699141796	9,25E-05	1,672708041
BSU34110	RsbP	0,713383588	1,63964511	0,001744158	1,336904099	2,526086601	2,18E-07	2,082865856
BSU34120	YvfO	-0,44282932	0,735690404	0,06982175	0,491052511	1,405469856	0,06240723	1,07058013
BSU34130	LacA	-1,30455511	0,404845933	7,39E-07	0,164515388	1,120789533	0,054680651	0,762817733
BSU34140	YvfM	-0,62407782	0,648834385	0,000847414	0,751266439	1,683269804	0,002624228	1,166052095
BSU34150	YvfL	-0,03340561	0,977111018	0,159310579	0,865018003	1,821362395	1,57E-05	1,399236706
BSU34160	CycB	0,228343281	1,171488897	0,176892713	1,39531327	2,630456641	2,95E-08	1,900972769
BSU34170	LacR	-0,07810156	0,947303382	0,354752528	0,163623526	1,120096885	0,272757102	1,033700133
BSU34180	LutR	0,121307836	1,087720459	0,002998322	0,146631737	1,106981979	0,539843565	1,097351219
**BSU34190**	**LutP**	**1,647724259**	**3,13338981**	**1,56E-10**	**1,222912717**	**2,334174982**	**3,17E-09**	**2,733782396**
BSU34200	SigL	0,271458464	1,207027432	0,222983393	-0,02028526	0,986037721	0,633516516	1,096532576
BSU34210	YvfG	-0,4047857	0,755348484	0,022973842	-0,29609506	0,814453899	0,236138972	0,784901192
BSU34220	EpsO	-2,11986671	0,230068167	2,89E-13	0,043507687	1,030616566	0,263485762	0,630342366
BSU34230	EpsN	-2,6348318	0,161003974	9,66E-16	0,345292722	1,270408722	0,105564115	0,715706348
BSU34240	EpsM	-2,8969453	0,134255649	2,66E-17	0,458191194	1,373818287	0,015295584	0,754036968
BSU34250	EpsL	-2,80553416	0,143037551	1,34E-12	0,269864302	1,205694417	0,222598037	0,674365984
BSU34265	EpsK	-2,71223341	0,152593625	3,86E-15	0,139817899	1,101766039	0,20521985	0,627179832
BSU34280	EpsJ	-2,46819948	0,180716548	2,56E-15	0,363607368	1,286639038	0,066362197	0,733677793
BSU34290	EpsI	-2,04091411	0,243009714	6,58E-13	0,445070359	1,361380509	0,004017606	0,802195111
BSU34300	EpsH	-2,39414576	0,190234952	1,61E-14	0,515198311	1,429190581	0,001361543	0,809712767
BSU34310	EpsG	-1,67193102	0,313833003	2,73E-10	0,551293345	1,465398806	0,019398255	0,889615904
BSU34320	EpsF	-2,40506296	0,188800834	4,48E-15	0,507114509	1,421204842	0,0081687	0,805002838
BSU34330	EpsE	-1,84292935	0,278755205	1,07E-13	0,37956148	1,300946361	0,008025635	0,789850783

BSU34340	EpsD	-2,24676479	0,210696056	4,43E-15	0,347133355	1,272030581	0,043842705	0,741363319
BSU34350	EpsC	-2,26056319	0,208690496	8,19E-13	0,308729937	1,238616815	0,090180241	0,723653656
BSU34360	EpsB	-1,33745903	0,395717005	5,38E-08	-0,12829384	0,914912804	0,207547438	0,655314905
BSU34370	EpsA	-2,4041482	0,188920584	1,17E-14	0,254272916	1,192734481	0,145503803	0,690827533
BSU34380	SlrR	-1,95956934	0,257105194	7,39E-12	0,055646045	1,039324413	0,55753191	0,648214803
BSU34390	PnbA	-0,47402043	0,719955463	0,009612925	-0,13745783	0,909119701	0,677546133	0,814537582
BSU34400	PadC	-1,79907935	0,287357907	0,02136872	0,045534137	1,032065216	0,352514805	0,659711562
**BSU34410**	**YveG**	**-1,28645772**	**0,409956369**	**0,000549272**	**-1,56276228**	**0,338502341**	**0,000186367**	**0,374229355**
BSU34420	YveF	-1,26961608	0,414770134	0,293070562	-0,30568992	0,80905522	0,35243059	0,611912677
BSU34430	RacX	1,287556665	2,441142755	1,37E-08	0,541088976	1,45507042	0,012218654	1,948106587
BSU34440	PbpE	1,099299236	2,142505988	1,85E-07	1,076651108	2,109134513	1,09E-06	2,12582025
BSU34460	LevB	-1,81335849	0,284527797	0,03267917	0,25901712	1,196663164	0,430435096	0,74059548
**BSU34470**	**YveA**	**-3,53277684**	**0,086402877**	**4,22E-19**	**-1,73271414**	**0,300885369**	**2,54E-11**	**0,193644123**
BSU34480	YvdT	0,127608381	1,092481145	0,008063801	-0,35508986	0,781820948	0,111397514	0,937151047
BSU34490	YvdS	0,595983779	1,511502933	0,093805764	-0,15757662	0,896529762	0,232021594	1,204016348
BSU34500	YvdR	-0,00529864	0,996334	0,162533148	-0,79710378	0,575503344	0,00260611	0,785918672
**BSU34510**	**YvdQ**	**-3,08816359**	**0,117589929**	**5,97E-18**	**-1,69216729**	**0,309461686**	**1,09E-10**	**0,213525807**
BSU34520	CotQ	-2,09985343	0,233281948	4,36E-11	-0,77029327	0,586298278	8,87E-05	0,409790113
BSU34530	CotR	-0,38960305	0,763339607	0,008318624	-0,84813676	0,555501705	7,57E-05	0,659420656
BSU34540	ClpP	-0,72699072	0,604162807	0,000984634	-0,24982748	0,840996977	0,127064991	0,722579892
BSU34550	PgcM	0,27511499	1,210090536	6,37E-05	0,427838675	1,345216775	0,031575804	1,277653656
BSU34560	MalL	-0,23865527	0,847534929	0,225424749	0,35899779	1,282534638	0,108981695	1,065034784
BSU34570	YvdK	-0,21384156	0,862238234	0,544596418	0,989235047	1,985132145	7,87E-06	1,423685189
BSU34580	YvdJ	-0,08265742	0,944316627	0,42786016	1,395745701	2,631245208	2,20E-09	1,787780917
BSU34590	MdxG	0,788737163	1,727561611	0,001355812	1,215298174	2,321887664	1,14E-07	2,024724637
BSU34600	MdxF	0,369393008	1,291809207	0,044331588	0,975741672	1,96665197	2,09E-05	1,629230588
BSU34610	MdxE	0,056956028	1,04026856	0,426284164	1,03619356	2,050809605	9,32E-06	1,545539082
BSU34620	YvdF	0,368992292	1,29145045	0,106311899	1,109607355	2,157869106	8,61E-07	1,724659778
BSU34630	YvdE	-0,14970632	0,901433941	6,21E-05	0,509148315	1,423209765	0,001861688	1,162321853

BSU34640	YvdD	0,398245405	1,317904111	4,40E-05	-0,23132032	0,851854938	0,036847474	1,084879524
BSU34650	YvdC	0,963023709	1,949391298	9,96E-07	0,248148242	1,187681699	0,259345383	1,568536498
BSU34660	YvdB	-1,08288037	0,472085354	3,28E-06	0,283316112	1,216988986	0,122458611	0,84453717
BSU34670	YvdA	-0,99145314	0,502970908	8,89E-06	0,400378226	1,319853886	0,051264262	0,911412397
BSU34680	YvcT	-0,35725611	0,780647897	0,057190351	-0,12784264	0,915198988	0,589526507	0,847923443
BSU34690	PsdB	-0,46375955	0,725094255	0,078129093	0,490330241	1,404766398	0,011352727	1,064930326
BSU34700	PsdA	0,352009497	1,276337171	0,028105242	0,81641953	1,761030053	5,63E-05	1,518683612
BSU34710	PsdS	0,306119456	1,23637763	0,00721527	0,322456594	1,250457993	0,01772168	1,243417811
BSU34720	PsdR	0,407173313	1,326085055	0,068119089	0,811140508	1,754597974	0,000408375	1,540341514
BSU34729	YvzJ	-1,20548249	0,433624298	0,085470697	-1,17417166	0,44313812	0,070367236	0,438381209
BSU34730	YvcN	0,087977085	1,062878792	0,022748037	-0,09106633	0,938828584	0,472925849	1,000853688
BSU34740	Crh	0,297476794	1,228993081	0,143407723	-0,04276567	0,970792138	0,886596827	1,099892609
BSU34750	YvcL	-0,42864657	0,742958447	0,107192418	0,155133765	1,113524858	0,237414136	0,928241653
BSU34760	YvcK	-0,38980377	0,763233411	0,046200711	0,355480729	1,279411831	0,042562365	1,021322621
BSU34770	YvcJ	-0,67754709	0,625227401	0,007569029	0,405246899	1,324315531	0,023625006	0,974771466
BSU34780	YvcI	-0,6223733	0,649601422	0,009409161	0,258990946	1,196641454	0,072291503	0,923121438
BSU34790	TrxB	-0,36070286	0,778785075	0,240417715	-0,6891511	0,620218688	0,015518611	0,699501882
BSU34800	CwlO	1,93323118	3,819095986	2,56E-10	0,189151719	1,140093163	0,07079974	2,479594574
BSU34810	YvcD	0,815770286	1,76023773	0,000318993	0,322680558	1,250652129	0,103895346	1,50544493
BSU34820	BmrA	0,512072598	1,426097478	0,0004436	1,021348279	2,029815048	5,73E-06	1,727956263
BSU34830	YvzA	-1,41738156	0,374391204	5,81E-07	-0,11926936	0,920653792	0,180227329	0,647522498
BSU34840	YvcB	-1,48341816	0,357640453	2,23E-07	-0,05261447	0,964187433	0,463290671	0,660913943
BSU34850	YvcA	-1,27005815	0,414643059	1,42E-06	-0,06087922	0,958679695	0,639683887	0,686661377
BSU34860	HisI	-0,87917473	0,543678346	0,00386734	-0,07755948	0,947659387	0,68608289	0,745668867
BSU34870	HisF	-0,66624818	0,630143288	0,040104633	0,257481029	1,19538971	0,104151529	0,912766499
BSU34880	HisA	-0,51364282	0,700451554	0,006296499	0,414714308	1,333034675	0,032788336	1,016743114
BSU34890	HisH	-0,48509448	0,714450281	0,037968517	0,505702208	1,41981426	0,009799521	1,06713227
BSU34900	HisB	-1,50700955	0,351839766	2,26E-09	0,724231535	1,652020437	0,000188039	1,001930102
BSU34910	HisD	-1,54811272	0,341957108	2,78E-08	0,599566916	1,51526163	0,002366292	0,928609369

BSU34920	HisG	-0,97180451	0,509867926	0,000132119	0,468281191	1,383460249	0,017223481	0,946664087
BSU34930	HisZ	-0,76269614	0,589393829	0,001404232	0,621405558	1,538373224	0,003122376	1,063883527
BSU34940	YvpB	-0,04174504	0,971479168	0,299991643	0,170640463	1,125558048	0,292887648	1,048518608
BSU34950	PelC	0,738512236	1,668454375	0,003477574	0,29238735	1,22466516	0,119205718	1,446559767
BSU34960	YvoF	-1,12829567	0,457455824	5,33E-06	-0,17371403	0,886557417	0,378684188	0,67200662
BSU34970	YvoE	-1,27466827	0,413320187	7,11E-08	0,320593115	1,248843863	0,083997511	0,831082025
BSU34980	YvoD	-0,73878606	0,599243366	0,00108327	0,015935742	1,011107045	0,497981441	0,805175205
BSU34990	Lgt	-0,82945272	0,562742677	5,54E-05	-0,19375205	0,874328877	0,681528976	0,718535777
BSU35000	HprK	-0,82822016	0,56322366	0,000185232	-0,12099617	0,919552485	0,593459858	0,741388072
BSU35010	NagA	-0,97790797	0,507715436	0,000209633	0,509737137	1,423790753	0,009656256	0,965753095
BSU35020	NagB	-0,46030425	0,726832963	0,006786195	0,169247416	1,124471749	0,183042138	0,925652356
BSU35030	NagR	-0,25896729	0,835685907	0,017327731	0,106888641	1,076903248	0,588640931	0,956294578
**BSU35040**	**YvnB**	**-2,42286475**	**0,186485485**	**7,04E-16**	**-1,28448888**	**0,410516217**	**4,70E-08**	**0,298500851**
BSU35050	YvnA	-0,76381867	0,588935411	0,000400468	-0,67940566	0,624422462	0,004488808	0,606678937
BSU35060	CypX	1,113316825	2,163424577	9,44E-07	0,355146967	1,279115878	0,120372333	1,721270228
BSU35070	YvmC	0,659853971	1,579922697	7,50E-06	0,684351807	1,606979815	0,005998729	1,593451256
BSU35080	YvmB	-0,27975098	0,82373319	6,29E-05	-0,22659443	0,854649964	0,000862339	0,839191577
BSU35090	YvmA	-0,2453367	0,843618888	0,325865936	-0,21491271	0,861598291	0,024124139	0,852608589
BSU35100	YvlD	-0,40158375	0,757026787	0,05976026	-0,5166765	0,698980202	0,01081344	0,728003495
BSU35110	YvlC	-1,01239591	0,495722309	0,000270283	-0,54983255	0,683099411	0,018916579	0,58941086
BSU35120	YvlB	-0,97743257	0,507882766	3,39E-06	-0,69964282	0,615724628	0,014835039	0,561803697
BSU35130	YvlA	-0,52320193	0,695825794	0,056300928	-0,25887228	0,835740941	0,213361307	0,765783367
BSU35140	YvkN	-0,73506139	0,600792458	0,095140572	0,681732389	1,604064761	0,113570233	1,10242861
BSU35150	YvzB	-2,02785277	0,245219775	0,01350698	0,886088944	1,848159082	0,005066245	1,046689429
BSU35160	UvrA	0,452481161	1,368391608	0,044995606	0,362241725	1,285421693	0,037407322	1,32690665
BSU35170	UvrB	-0,38904404	0,763635437	0,209276935	0,555300072	1,469474243	0,002225267	1,11655484
BSU35180	CsbA	1,268459329	2,409041637	2,42E-08	0,26564405	1,202172605	0,171605575	1,805607121
BSU35190	YvkC	-0,94200465	0,520509121	0,000411501	0,29545431	1,227271389	0,200512009	0,873890255
BSU35200	YvkB	-1,10134752	0,466080959	1,83E-05	0,084293356	1,060168335	0,431058988	0,763124647

BSU35210	YvkA	0,312569999	1,241918068	0,128841513	-0,426371	0,744131244	0,166691465	0,993024656
BSU35220	MinJ	1,40037054	2,639693711	3,43E-06	0,600134602	1,515857988	0,035980206	2,07777585
**BSU35240**	**CtpB**	**-2,15502045**	**0,224529911**	**2,89E-13**	**-1,09851271**	**0,466997682**	**4,94E-06**	**0,345763796**
BSU35250	FtsX	1,321541371	2,499329948	5,85E-06	0,499972233	1,414186343	0,061781265	1,956758146
BSU35260	FtsE	0,280990892	1,215029122	0,002310426	0,757292481	1,690315416	0,000181472	1,452672269
BSU35270	CccB	0,387787542	1,308385378	0,08443621	-0,42775825	0,743416053	0,022732698	1,025900716
BSU35280	YvjA	-1,09481048	0,468197624	1,26E-05	0,085397417	1,060979968	0,612827432	0,764588796
BSU35290	PrfB	0,315915714	1,244801509	0,013412053	0,249001324	1,188384196	0,058259544	1,216592852
BSU35300	SecA	0,237895145	1,17927088	0,184710078	0,706757835	1,632132107	0,000395933	1,405701493
BSU35310	YvyD	0,979765103	1,972144281	3,70E-06	-0,42937744	0,742582159	0,082973507	1,35736322
BSU35319	SmiA	0,420106497	1,338026322	0,002811155	-0,47124305	0,721342809	0,043247208	1,029684565
BSU35320	FliT	-0,47832018	0,717812934	0,024779916	-0,31447267	0,804144865	0,205768516	0,7609789
BSU35330	FliS	-0,3757111	0,770725431	0,067246431	-0,31015805	0,806553396	0,101905039	0,788639413
BSU35340	FliD	-0,53473998	0,690283075	0,049619607	-0,38502987	0,765763144	0,114280408	0,72802311
BSU35350	YvyC	-0,3355252	0,792495585	0,073190842	-0,24264556	0,845194007	0,276754432	0,818844796
BSU35360	Hag	-0,65971079	0,63300518	5,94E-05	-1,30554325	0,404568739	1,03E-06	0,51878696
BSU35370	CsrA	-0,35129325	0,7838811	3,56E-05	0,175954346	1,129711464	0,453534337	0,956796282
BSU35380	FliW	-0,05374578	0,96343165	0,479645587	-0,19537286	0,873347153	0,344964602	0,918389401
BSU35390	YviE	-0,46611207	0,723912848	0,024155641	-0,1576178	0,896504173	0,534588807	0,810208511
BSU35400	FlgL	-0,30588279	0,808947068	0,019950608	-0,26733811	0,83085112	0,280625737	0,819899094
BSU35410	FlgK	-0,68087486	0,623786892	0,006079482	-0,11245523	0,9250125	0,594098402	0,774399696
BSU35420	YvyG	-0,84927232	0,555064635	0,000113037	-0,42062134	0,747102794	0,064748315	0,651083714
BSU35430	FlgM	-0,96456215	0,512433908	3,44E-05	-0,51373609	0,700406274	0,030677752	0,606420091
BSU35440	YvyF	-0,97590213	0,508421826	1,99E-06	-0,20439613	0,867901891	0,185068819	0,688161859
BSU35450	ComFC	-1,00718278	0,497516825	1,10E-05	-0,06181849	0,958055746	0,781185623	0,727786286
BSU35460	ComFB	-0,97481773	0,508804123	2,24E-05	0,262322399	1,199407915	0,129394685	0,854106019
BSU35470	ComFA	-1,10780319	0,464000031	4,81E-05	-0,40114515	0,757256966	0,042793201	0,610628498
BSU35480	DegV	0,958317823	1,943042992	1,64E-07	0,483843793	1,398464657	0,015435916	1,670753825
BSU35490	DegU	0,560497234	1,474777421	0,009010546	0,745093492	1,676082882	9,13E-05	1,575430152

BSU35500	DegS	0,460672001	1,376182689	0,004571407	0,575266376	1,489952539	0,00159305	1,433067614
BSU35510	YvyE	0,221299013	1,165782793	0,009522124	1,06348219	2,089969936	4,89E-06	1,627876365
BSU35520	TagV	-0,33618462	0,792133434	0,157016208	0,320691875	1,248929356	0,120818664	1,020531395
BSU35530	TagO	0,014936237	1,010406788	0,78834485	-0,16174185	0,893945106	0,334471523	0,952175947
BSU35540	TuaH	-0,36235902	0,777891571	0,086014341	0,087149586	1,062269322	0,470705283	0,920080446
BSU35550	TuaG	-1,08061668	0,472826671	8,91E-05	0,267975716	1,204117113	0,032473532	0,838471892
BSU35560	TuaF	-0,99387381	0,502127689	0,000253966	0,153507158	1,112270091	0,281110077	0,80719889
BSU35570	TuaE	-0,43995287	0,737158691	0,004007078	0,104108	1,074829632	0,19413741	0,905994161
BSU35580	TuaD	-0,51869847	0,698001252	0,013173621	0,157262404	1,115169032	0,280629565	0,906585142
BSU35590	TuaC	-0,48377804	0,715102503	0,001523945	0,164374622	1,120680182	0,145207181	0,917891342
BSU35600	TuaB	-0,64232746	0,640678525	0,010818733	0,458375286	1,373993602	0,028793591	1,007336063
BSU35620	LytC	0,510790863	1,424831052	0,000945269	0,616831784	1,533503845	0,001939133	1,479167449
BSU35630	LytB	0,503670599	1,417816278	0,018752746	0,971952703	1,961493702	6,95E-06	1,68965499
BSU35640	LytA	0,986901581	1,981923917	5,81E-08	0,566880504	1,481317098	0,005291253	1,731620507
BSU35650	TagU	0,691632112	1,615109649	0,001284633	0,312324286	1,241706568	0,099033038	1,428408109
BSU35660	MnaA	-0,58028491	0,66883168	0,010911209	-0,18857995	0,877468993	0,091646955	0,773150336
BSU35670	GtaB	1,472170213	2,774389249	4,42E-10	0,368868342	1,291339499	0,025242551	2,032864374
BSU35680	GgaB	-0,24818259	0,841956386	0,067343269	0,456007791	1,371740696	0,018184493	1,106848541
BSU35690	GgaA	0,713214378	1,639452812	0,031089583	0,709102937	1,634787296	0,005164479	1,637120054
BSU35700	TagH	-0,04584766	0,968720477	0,581054695	0,404353928	1,323496086	0,042202017	1,146108282
BSU35710	TagG	0,622554881	1,539599258	0,00717163	0,686058827	1,608882344	0,000858569	1,574240801
BSU35720	TagF	0,007832014	1,005443501	0,051266755	0,879286815	1,839465751	5,72E-05	1,422454626
BSU35730	TagE	-0,08614062	0,942039447	0,814897896	0,680878847	1,603116029	0,000933503	1,272577738
BSU35740	TagD	1,166549271	2,244741428	9,71E-09	-0,04179107	0,971448167	0,90312079	1,608094798
BSU35750	TagA	0,088510362	1,063271747	0,552838366	-0,06133193	0,958378915	0,437090255	1,010825331
BSU35760	TagB	0,368082136	1,290635968	0,005203043	-0,29795356	0,813405379	0,154927994	1,052020673
BSU35770	TagC	-0,1895569	0,876874996	0,013894213	0,505447278	1,419563394	0,034418766	1,148219195
**BSU35780**	**LytD**	**-2,15763882**	**0,224122777**	**1,25E-13**	**-1,66695171**	**0,314918035**	**1,14E-10**	**0,269520406**
BSU35790	Pmi	0,951937666	1,934469076	5,37E-07	0,382375696	1,30348655	0,056269252	1,618977813

**BSU35800**	**GerBA**	**-3,36582923**	**0,097002838**	**3,19E-19**	**-1,27614337**	**0,412897798**	**2,49E-06**	**0,254950318**
**BSU35810**	**GerBB**	**-2,96191026**	**0,128344177**	**1,33E-15**	**-1,22799934**	**0,426909054**	**4,68E-07**	**0,277626615**
**BSU35820**	**GerBC**	**-2,77999007**	**0,145592701**	**5,76E-17**	**-1,62848078**	**0,323428612**	**5,57E-10**	**0,234510656**
**BSU35830**	**YwtG**	**2,227073477**	**4,681833006**	**4,40E-14**	**1,415208195**	**2,666982197**	**2,10E-07**	**3,674407602**
BSU35840	TagT	0,643922044	1,562571325	0,000470378	0,811014644	1,754444905	0,000794127	1,658508115
BSU35850	YwtE	-0,63486939	0,643999115	0,033563904	-0,20318115	0,868633108	0,399294793	0,756316111
BSU35860	PgdS	0,639992571	1,558321135	0,002586564	-0,24196884	0,845590553	0,332391654	1,201955844
BSU35870	CapE	0,420655519	1,338535609	0,425680903	0,289215502	1,22197562	0,014869658	1,280255615
BSU35880	CapA	0,164216572	1,120557415	0,646600915	0,326467909	1,253939644	0,213820375	1,18724853
BSU35890	CapC	-0,045145	0,969192401	0,074224501	0,986651715	1,98158069	0,000655717	1,475386546
BSU35900	CapB	-0,32167992	0,800137629	0,031445387	0,390535568	1,310879948	0,196267036	1,055508789
BSU35910	RbsR	0,485440663	1,400013427	0,041866955	1,126711382	2,183604208	1,65E-07	1,791808817
BSU35920	RbsK	1,137353531	2,199771284	2,33E-05	1,100760055	2,144676507	1,51E-07	2,172223895
BSU35930	RbsD	0,195167275	1,144856895	0,380293197	1,297087022	2,457322179	6,61E-09	1,801089537
BSU35940	RbsA	0,641953858	1,560441051	0,009075387	1,366887949	2,579136177	1,55E-10	2,069788614
BSU35950	RbsC	0,48244384	1,397108281	0,001489204	1,668713959	3,179310584	3,82E-12	2,288209433
BSU35960	RbsB	0,263654021	1,200515493	0,183488883	1,42366073	2,682653512	1,53E-10	1,941584502
BSU35970	YwsB	1,573409888	2,976072946	8,01E-09	0,502543094	1,416708649	0,000591436	2,196390797
**BSU35980**	**YwsA**	**-2,42519768**	**0,186184171**	**3,45E-14**	**-1,41716571**	**0,374447221**	**3,39E-08**	**0,280315696**
BSU35990	YwrO	-1,09150357	0,469272045	2,26E-06	-0,89574959	0,537467866	4,31E-05	0,503369955
**BSU36000**	**AlsD**	**-1,62813219**	**0,323506769**	**1,17E-06**	**-1,1621458**	**0,446847421**	**0,001588212**	**0,385177095**
**BSU36010**	**AlsS**	**-1,51765155**	**0,349253978**	**7,40E-09**	**-1,07974763**	**0,473111577**	**0,00037974**	**0,411182778**
BSU36020	AlsR	0,011665063	1,008118382	0,01570266	0,117444444	1,08481155	0,275309709	1,046464966
BSU36030	YwrK	-0,96079512	0,513773676	0,002376974	-1,15308293	0,449663308	8,09E-07	0,481718492
**BSU36040**	**YwrJ**	**-2,49632168**	**0,177227983**	**3,64E-11**	**-1,37496461**	**0,385562164**	**3,55E-07**	**0,281395073**
**BSU36050**	**CotB**	**-1,61948918**	**0,325450678**	**5,29E-11**	**-1,41726191**	**0,374422255**	**5,31E-09**	**0,349936467**
**BSU36060**	**CotH**	**-1,97530198**	**0,254316684**	**1,25E-12**	**-1,40616342**	**0,377313749**	**2,72E-08**	**0,315815216**
BSU36070	CotG	-0,61592011	0,652513604	0,006501595	-2,09984698	0,23328299	5,00E-13	0,442898297
BSU36080	YwrF	-0,46330959	0,72532044	0,012745385	0,010766936	1,007490989	0,419011857	0,866405715

BSU36090	YwrE	0,450667432	1,366672372	0,314320665	0,126608606	1,091724328	0,601746491	1,22919835
BSU36100	YwrD	-0,84058532	0,558416967	0,282419855	-0,37661526	0,770242559	0,375311367	0,664329763
BSU36110	YwrC	-1,38148494	0,383823529	4,79E-09	-0,72434975	0,60526979	0,000472681	0,494546659
BSU36120	YwrB	-1,37133106	0,386534461	2,65E-06	-0,71855283	0,607706727	0,001640167	0,497120594
BSU36130	YwrA	-1,96100086	0,256850207	1,68E-07	-0,84080423	0,558332238	0,000952266	0,407591223
BSU36140	YwqO	-0,74491693	0,596702227	0,00769216	-0,02720619	0,981318803	0,768179631	0,789010515
BSU36150	YwqN	-0,38347919	0,766586667	0,000173734	-0,23225719	0,851301931	0,127197656	0,808944299
BSU36160	YwqM	-0,64241785	0,640638384	0,015669564	-0,24915399	0,841389668	0,20564826	0,741014026
BSU36170	YwqL	-0,7151339	0,609148591	0,00182778	-0,02917418	0,979981096	0,702718455	0,794564844
BSU36180	YwqK	-1,17415773	0,443142398	1,57E-06	0,11776158	1,085050042	0,393352262	0,76409622
BSU36190	YwqJ	-0,60197977	0,658849214	0,001700437	-0,16824332	0,88992563	0,622825983	0,774387422
BSU36200	YwqI	-1,35810072	0,390095506	1,04E-08	-0,22654291	0,854680485	0,41364157	0,622387996
BSU36210	YwqH	-1,40150169	0,378534924	1,68E-07	-0,29046227	0,817640026	0,360251053	0,598087475
BSU36220	YwqG	-0,09252026	0,937882917	0,157236798	0,185916185	1,137539139	0,043453726	1,037711028
BSU36230	Ugd	-1,40731562	0,37701253	3,67E-09	-0,66866632	0,629087972	0,003250153	0,503050251
BSU36240	PtpZ	-0,21705804	0,860318018	0,337175676	1,012366508	2,017217306	0,000521426	1,438767662
BSU36250	PtkA	0,012756278	1,008881184	0,489906374	0,650823629	1,570064283	0,00613062	1,289472734
BSU36260	TkmA	-0,13671358	0,909588814	0,343066235	-0,00259614	0,998202113	0,679185759	0,953895463
BSU36269	YwzD	-0,05331204	0,963721345	0,903656524	-0,78665025	0,57968849	0,001054655	0,771704918
BSU36270	YwqB	-1,70437535	0,306854076	3,71E-10	0,041874968	1,029450861	0,135975247	0,668152468
BSU36280	YwqA	-0,45838876	0,72779863	0,008941114	-0,36128359	0,778471652	0,109375621	0,753135141
BSU36290	YwpJ	-0,31143224	0,805841359	0,064152986	-0,41710863	0,748924076	0,021881761	0,777382717
BSU36300	GlcR	-1,73181883	0,30107215	1,58E-09	0,142480278	1,103801138	0,490733035	0,702436644
BSU36310	SsbB	-0,18576488	0,87918283	0,216095006	-0,77806977	0,583146484	0,00012024	0,731164657
BSU36320	YwpG	-0,06326947	0,957092673	0,554417113	0,140849504	1,102554143	0,594855643	1,029823408
BSU36330	YwpF	-0,06348265	0,956951257	0,196871009	-0,7029021	0,614335177	0,003858374	0,785643217
BSU36340	YwpE	-0,55166804	0,682230877	0,67050653	0,729826349	1,65843946	0,378065864	1,170335169
BSU36350	YwpD	-0,4847441	0,714623815	0,000130215	-0,07562137	0,948933327	0,011597021	0,831778571
BSU36360	MscL	0,508467246	1,422538054	0,000814082	0,339599038	1,265404856	0,082608124	1,343971455

BSU36370	YwpB	0,924413726	1,897912822	1,37E-05	0,359155288	1,282674659	0,089199674	1,590293741
BSU36380	RapD	0,774018301	1,710026048	0,000278334	0,297120688	1,228689762	0,105539936	1,469357905
BSU36390	FlhP	-0,02113257	0,985458782	0,346250211	-0,56542822	0,675754809	0,010876043	0,830606796
BSU36400	FlhO	-0,32730644	0,797023165	0,196425455	-0,37328968	0,772020103	0,097874878	0,784521634
BSU36410	Mbl	-0,12722892	0,915588392	0,01004311	-0,47737561	0,718283059	0,009800962	0,816935725
BSU36420	SpoIIID	-0,67421314	0,626673919	1,46E-05	-1,04963535	0,483090255	0,000540861	0,554882087
**BSU36430**	**Usd**	**-1,45703021**	**0,364242152**	**4,09E-10**	**-1,05428032**	**0,481537374**	**0,001001415**	**0,422889763**
BSU36440	YwoH	-0,19858108	0,87140719	0,295089885	0,04932895	1,034783496	0,16272174	0,953095343
BSU36450	YwoG	0,11465215	1,08271396	0,001829424	-0,77036504	0,586269115	0,000609436	0,834491538
BSU36460	YwoF	-0,73178612	0,602157952	0,000932501	0,416689942	1,33486139	0,03411423	0,968509671
BSU36470	PucI	-2,15047023	0,225239189	0,003052065	0,30821019	1,23817067	0,327278772	0,73170493
**BSU36480**	**YwoD**	**-2,96531798**	**0,128041379**	**3,70E-14**	**-1,48738981**	**0,356657247**	**2,16E-09**	**0,242349313**
BSU36490	YwoC	-3,21047731	0,108031406	1,38E-18	-0,85086988	0,554450328	0,000782061	0,331240867
BSU36500	YwoB	-0,77889212	0,58281418	0,051345692	0,71781971	1,644694591	0,035937982	1,113754386
BSU36510	NrgA	-0,51067983	0,701891614	0,000644819	-0,21346011	0,862466237	0,031050137	0,782178925
BSU36520	NrgB	-0,00263001	0,998178678	0,285631649	-0,78150933	0,581757845	0,000471179	0,789968262
BSU36530	BcrC	1,241069978	2,363737744	4,38E-08	0,086372575	1,061697356	0,608069329	1,71271755
BSU36540	YwnJ	0,971484003	1,96085656	0,000128166	0,120493434	1,087106614	0,115389148	1,523981587
BSU36550	SpoIIQ	0,853621481	1,807031282	0,000338272	0,323836984	1,251655021	0,001625526	1,529343151
BSU36560	YwnH	1,022899635	2,031998919	4,37E-06	-0,04421412	0,969817965	0,592756754	1,500908442
BSU36570	YwnG	-0,16411787	0,892474052	0,024124632	-0,92220577	0,527701588	0,000250758	0,71008782
BSU36580	YwnF	-0,77245333	0,58542111	0,004705088	-1,00857482	0,497037009	1,18E-05	0,54122906
BSU36590	ClsA	-1,27169009	0,414174293	3,45E-07	-1,09986238	0,466560998	9,85E-06	0,440367645
BSU36600	Mta	-0,12587126	0,916450419	0,000317187	0,082738188	1,059026132	0,336989991	0,987738276
BSU36610	YwnC	-1,00260407	0,499098313	0,003018602	-0,59298474	0,662969895	0,007753014	0,581034104
BSU36620	YwnB	-0,45216305	0,730946109	0,354380551	0,263999048	1,200802635	0,219921878	0,965874372
BSU36630	YwnA	-0,11146093	0,925650237	0,32571354	-0,67209863	0,62759309	0,005378872	0,776621663
BSU36640	UreC	0,605452991	1,5214564	0,179432459	1,470517404	2,771212618	0,000152444	2,146334509
BSU36650	UreB	0,611157087	1,527483808	0,000221208	0,92742009	1,901871917	8,68E-05	1,714677863

BSU36660	UreA	0,398561662	1,318193043	0,034749467	1,02781168	2,0389292	2,50E-05	1,678561122
BSU36670	CsbD	2,123343031	4,357023912	1,25E-14	0,583585913	1,498569416	0,008627718	2,927796664
BSU36680	YwmF	0,110509356	1,079609335	1	0,080935539	1,057703703	0,929887094	1,068656519
BSU36690	RapB	0,258697852	1,196398371	0,097189125	0,378148111	1,299672484	0,036281304	1,248035427
BSU36700	MoaA	-0,22199196	0,857380816	0,135590499	-0,03229033	0,977866663	0,41367353	0,917623739
BSU36710	FdhD	-0,33597883	0,792246439	6,83E-05	-0,33001599	0,795527668	0,177220455	0,793887054
BSU36720	YwmE	-0,89419694	0,538046609	0,001844383	-1,93207499	0,262051998	6,42E-12	0,400049303
BSU36730	YwmD	-2,31814216	0,200525531	0,00012559	-0,23757346	0,848170693	0,09322415	0,524348112
BSU36740	YwmC	-0,11494576	0,923417026	0,000119541	-0,33776733	0,791264903	0,054657804	0,857340964
BSU36750	SpoIID	0,334743106	1,261152822	0,001320999	-0,13665294	0,90962705	0,773954105	1,085389936
BSU36760	MurAA	0,642252858	1,560764489	0,005752319	-0,30003789	0,812231064	0,203474414	1,186497776
BSU36770	YwmB	0,083447562	1,059546983	0,024717088	0,180712995	1,133443907	0,354791072	1,096495445
BSU36780	YwzB	-0,27347091	0,827326723	0,046131028	-0,09131151	0,938669048	0,906061183	0,882997886
BSU36790	YwmA	0,343979426	1,269252785	0,214075324	-0,7055294	0,613217426	0,002179586	0,941235106
BSU36800	AtpC	0,909166464	1,877960169	6,59E-05	0,405753582	1,32478072	0,004251088	1,601370444
BSU36810	AtpD	0,55358048	1,467723776	0,000469897	0,648759388	1,567819406	4,67E-05	1,517771591
BSU36820	AtpG	0,258441299	1,196185636	0,015307944	0,877136967	1,836726693	4,52E-07	1,516456164
BSU36830	AtpA	-0,18653114	0,878715992	0,102627095	0,905335296	1,872979743	1,50E-08	1,375847868
BSU36840	AtpH	0,018282652	1,012753206	0,157828621	0,788663141	1,727472975	3,58E-07	1,37011309
BSU36850	AtpF	0,150088959	1,109637892	0,499955394	0,789375927	1,728326671	6,09E-07	1,418982282
BSU36860	AtpE	0,708888139	1,634543916	0,003369552	0,870574406	1,828390726	8,18E-08	1,731467321
BSU36870	AtpB	-0,04525575	0,969118001	0,320611824	1,061585331	2,087223845	3,65E-10	1,528170923
BSU36880	AtpI	0,133428686	1,096897475	0,220519488	0,5181549	1,432122498	0,001026067	1,264509986
BSU36890	Upp	0,759305151	1,692675181	0,0001032	0,568729674	1,483216989	0,008705968	1,587946085
BSU36900	GlyA	-0,11792899	0,921509543	0,407130077	0,348673891	1,273389603	0,051255687	1,097449573
BSU36910	YwlG	0,467889661	1,383084845	0,001258144	0,29026846	1,222867811	0,073711645	1,302976328
BSU36920	YwlF	0,53417364	1,448112454	0,017525804	0,447102249	1,363299226	0,0152923	1,40570584
BSU36930	YwlE	0,015944719	1,011113336	0,383264507	0,604467884	1,520417867	0,002995482	1,265765602
BSU36940	YwlD	1,810714432	3,50815972	3,67E-11	0,204341469	1,152160313	0,110132138	2,330160016

BSU36950	YwlC	0,449488162	1,3655557	0,000818199	0,28833139	1,221226999	0,0590686	1,293391349
BSU36960	YwlB	0,940002672	1,918531792	4,61E-08	-0,30290777	0,810616938	0,24976223	1,364574365
BSU36970	SpoIIR	0,780646303	1,71790029	5,04E-05	0,296472627	1,228137956	0,15513215	1,473019123
BSU36980	YwlA	1,042201391	2,059367621	0,020626268	-0,77011327	0,586371437	0,008821131	1,322869529
BSU36990	YwkF	-0,03995596	0,972684641	0,191558326	-0,91379289	0,530787796	0,000318319	0,751736219
BSU37000	YwkE	-0,44132714	0,736456828	0,039010517	-0,18057283	0,882352584	0,488394112	0,809404706
BSU37010	PrfA	-0,36917882	0,774223059	0,005085094	-0,47871515	0,717616444	0,031103107	0,745919751
BSU37020	YwkD	-0,06362026	0,956859988	0,405098734	-0,63969107	0,641850377	0,002780735	0,799355183
BSU37030	RacA	1,632037873	3,099505089	4,41E-12	-0,41529417	0,749866581	0,058168432	1,924685835
**BSU37040**	**YwkB**	**-1,96982782**	**0,255283496**	**1,17E-10**	**-1,82799722**	**0,28165535**	**7,28E-12**	**0,268469423**
BSU37050	MaeA	-1,50190281	0,353087387	0,034051217	1,161287151	2,236568819	0,135019839	1,294828103
BSU37060	Tdk	1,03374961	2,04733844	1,50E-05	-0,17205738	0,887576035	0,378797901	1,467457238
BSU37070	RpmE	1,019297284	2,026931429	5,81E-07	-0,56259333	0,677083973	0,028258911	1,352007701
BSU37080	Rho	0,073395113	1,052189904	0,14018314	0,230467319	1,173214917	0,145741393	1,11270241
BSU37090	GlpX	0,672177306	1,593476017	3,95E-05	0,346617174	1,271575543	0,045110613	1,43252578
BSU37100	MurAB	0,252334067	1,191132633	0,222896353	0,544027776	1,45803745	0,003506293	1,324585041
BSU37110	YwjH	0,153995728	1,112646826	0,114488335	0,527572762	1,441501924	0,003904279	1,277074375
BSU37120	FbaA	-0,02782168	0,98090024	0,516553825	0,395756256	1,315632229	0,00185571	1,148266234
BSU37130	Spo0F	0,490502776	1,404934407	0,011645175	0,997407524	1,996409293	3,27E-06	1,70067185
BSU37140	YwjG	-0,23863784	0,847545169	0,212523286	-0,37331541	0,772006333	0,011289043	0,809775751
BSU37150	PyrG	-1,68493829	0,311016216	1,78E-10	-0,86869243	0,547642975	0,000920467	0,429329596
BSU37160	RpoE	-0,06523804	0,955787604	0,664814838	0,063654112	1,045109504	0,548515629	1,000448554
BSU37170	AcdA	0,238347661	1,179640829	0,035078405	0,094439736	1,06765071	0,450724221	1,123645769
BSU37180	FadF	0,36364467	1,286672306	0,07960554	0,340610496	1,26629233	0,074802084	1,276482318
**BSU37190**	**YwjE**	**-2,56860003**	**0,168567694**	**7,28E-16**	**-1,48243975**	**0,357883082**	**4,22E-09**	**0,263225388**
**BSU37200**	**YwjD**	**-2,6480684**	**0,159533532**	**1,93E-13**	**-1,81246345**	**0,284704372**	**4,22E-09**	**0,222118952**
BSU37210	YwjC	0,686965803	1,609894115	0,000472961	-0,39318463	0,761446915	0,085232922	1,185670515
BSU37220	YwjB	1,338541169	2,528954655	3,22E-07	0,839063689	1,788888773	0,000146375	2,158921714
BSU37230	YwjA	0,291841885	1,224202217	0,053851459	0,746158836	1,677321026	0,00020811	1,450761621

BSU37240	YwiE	1,166272277	2,244310485	1,17E-06	0,937925674	1,915771735	9,91E-05	2,08004111
BSU37250	NarI	-0,77313842	0,585143177	0,142932179	-2,02967233	0,244910692	2,27E-07	0,415026935
BSU37260	NarJ	-1,52532101	0,347402247	0,006935209	-0,9918866	0,50281981	0,205337215	0,425111029
BSU37270	NarH	-2,43268998	0,185219773	8,19E-06	0,167282324	1,122941151	0,270420029	0,654080462
BSU37280	NarG	-2,13092497	0,228311436	9,35E-07	-0,31234544	0,805331437	0,167318902	0,516821437
BSU37290	ArfM	0,264580915	1,20128704	0,000164893	0,080935539	1,057703703	0,075204763	1,129495372
BSU37300	YwiC	1,615727726	3,064661485	6,23E-07	-0,26740363	0,830813386	0,041443252	1,947737435
BSU37310	Fnr	-0,85989298	0,550993431	2,24E-05	-1,22396168	0,428105514	6,98E-07	0,489549473
BSU37320	NarK	-0,74578524	0,596343203	0,000936839	0,398459305	1,318099523	0,141576242	0,957221363
BSU37330	ArgS	0,1524616	1,111464293	0,257034397	0,423848545	1,341501386	0,019854315	1,226482839
BSU37340	YwiB	-0,33147708	0,794722405	0,123591277	0,488063626	1,402561106	0,007503998	1,098641756
BSU37350	SboA	1,239071446	2,36046558	8,42E-09	-0,31254304	0,805221145	0,132142311	1,582843362
BSU37360	SboX	0,422342946	1,340102123	0,001585306	-0,06867174	0,95351548	0,002559247	1,146808801
BSU37370	AlbA	-0,17502982	0,88574921	0,134916878	-0,22616305	0,854905551	0,486351417	0,87032738
BSU37380	AlbB	-0,17652228	0,884833384	0,269237577	0,141563245	1,103099742	0,407182119	0,993966563
BSU37390	AlbC	-0,12167039	0,919122848	0,726043714	0,292975925	1,225164887	0,138824941	1,072143867
BSU37400	AlbD	0,100525692	1,072164069	0,082821733	0,38024177	1,301559956	0,048305426	1,186862012
BSU37410	AlbE	0,396817924	1,316600749	0,000292049	0,872872552	1,831305587	6,10E-05	1,573953168
BSU37420	AlbF	0,861755561	1,817248307	3,09E-07	0,681183957	1,603455102	0,000523818	1,710351704
BSU37430	AlbG	0,741260142	1,671635315	0,000514048	0,443159062	1,359578131	0,006444845	1,515606723
BSU37440	YwhL	-0,55338411	0,681419855	2,02E-05	0,354462572	1,278509227	0,003599582	0,979964541
BSU37450	YwhK	-1,15708832	0,448416628	3,77E-06	1,30791082	2,475827535	2,79E-08	1,462122082
BSU37460	RapF	-0,99665091	0,501162055	7,79E-06	-0,17315444	0,886901358	0,206537336	0,694031706
BSU37470	PhrF	-0,81557709	0,568181163	5,51E-05	-0,42807639	0,743252138	0,86072368	0,65571665
BSU37480	YwhH	-0,4666287	0,723653661	0,003792112	-1,14176626	0,453204391	3,05E-06	0,588429026
BSU37490	SpeB	-0,96241793	0,513196087	0,000100353	-0,43412751	0,74014123	0,048410762	0,626668658
BSU37500	SpeE	-1,04666757	0,484085047	5,11E-07	-0,52147681	0,696658335	0,060728354	0,590371691
BSU37510	PbpG	-0,17293773	0,887034594	0,030949424	-0,83560382	0,560348463	0,00014397	0,723691528
BSU37520	YwhD	-0,3489888	0,785134213	0,144071659	-0,05371721	0,963450728	0,65332214	0,874292471

BSU37530	YwhC	-0,05392798	0,963309983	0,650584362	-0,39905771	0,758353435	0,155381948	0,860831709
BSU37540	YwhB	0,444999906	1,361314027	0,034593299	-0,61579052	0,65257222	0,007703342	1,006943124
BSU37550	YwhA	-1,26169004	0,417055115	4,34E-07	-0,37573347	0,770713481	0,195014581	0,593884298
BSU37560	ThrZ	-2,32728583	0,199258638	1,22E-10	-0,30934937	0,807005625	0,208300999	0,503132132
BSU37570	Mmr	0,316620516	1,245409782	0,091873067	0,525900234	1,439831747	0,031619779	1,342620765
BSU37580	YwgB	0,220539051	1,165168861	0,1680357	0,734271618	1,663557368	0,01754342	1,414363114
BSU37590	YwgA	1,444181041	2,721083147	8,28E-08	1,089199475	2,127559494	4,16E-06	2,424321321
BSU37600	YwfO	0,854537674	1,808179213	6,61E-05	1,294495789	2,45291253	1,59E-08	2,130545872
BSU37610	YwzC	0,785305269	1,723456959	0,001536024	-0,018898	0,986986324	0,350146772	1,355221641
BSU37620	RsfA	1,052009635	2,073416049	1,13E-05	-0,32339492	0,799187033	0,220060233	1,436301541
BSU37630	YwfM	0,823053521	1,769146501	2,31E-05	-0,65089841	0,636883585	0,00466011	1,203015043
BSU37640	LipL	0,516298937	1,430281321	0,107401671	-0,3519476	0,783525643	0,185069159	1,106903482
BSU37650	CysL	-0,32473546	0,798444778	0,200761663	0,259901769	1,197397172	0,227796648	0,997920975
BSU37660	Pta	-1,08093584	0,472722082	5,60E-05	-1,11519102	0,461630029	1,39E-06	0,467176055
BSU37670	HemQ	0,86262693	1,818346232	0,000150376	-0,16165303	0,894000145	0,371111248	1,356173189
BSU37680	YwfH	0,991381852	1,988088324	4,60E-06	0,384102761	1,305047901	0,052230173	1,646568112
BSU37690	BacF	0,587753909	1,502905094	0,004522278	0,733973915	1,663214124	0,000662034	1,583059609
BSU37700	BacE	0,064006235	1,045364618	0,073546025	1,03218024	2,045112547	9,40E-07	1,545238583
BSU37710	BacD	-0,29065475	0,817530949	0,002187482	1,277693032	2,424509711	1,30E-08	1,62102033
BSU37720	BacC	-0,21495655	0,861572109	0,280558994	1,406885203	2,651640517	2,97E-09	1,756606313
BSU37730	BacB	0,211519758	1,1579073	0,084418273	1,540626834	2,909208777	9,45E-10	2,033558038
BSU37740	BacA	-0,9053693	0,533896019	0,000146427	1,476064813	2,781888916	2,07E-09	1,657892467
BSU37750	YwfA	0,275860258	1,210715806	0,174543387	-0,19178236	0,875523401	0,344848881	1,043119604
BSU37760	RocC	0,470186864	1,385288885	0,00120363	0,234648621	1,176620128	0,38259414	1,280954506
BSU37770	RocB	0,291722873	1,224101233	0,003837753	0,423357292	1,341044668	0,043117652	1,28257295
BSU37780	RocA	-1,00940957	0,496749503	0,001407922	0,911370298	1,880831097	0,000114924	1,1887903
BSU37790	RocG	0,270749853	1,206434719	0,014827812	0,48154565	1,396238744	0,00015454	1,301336731
BSU37800	YweA	0,649738062	1,568883321	9,02E-05	0,673776503	1,595243331	0,002902921	1,582063326
BSU37810	SpsL	-1,18640169	0,439397422	5,94E-07	-0,77868243	0,582898894	0,001804358	0,511148158

BSU37820	SpsK	-1,95741126	0,257490076	1,59E-11	-0,41699554	0,748982781	0,0599118	0,503236429
BSU37830	SpsJ	-2,00206597	0,24964225	3,49E-13	-0,5462339	0,684805454	0,018822382	0,467223852
BSU37840	SpsI	-2,41904024	0,186980504	4,93E-15	-0,74009334	0,598700615	0,000368392	0,39284056
**BSU37850**	**SpsG**	**-2,60011769**	**0,164925034**	**4,77E-16**	**-0,96198826**	**0,513348952**	**5,94E-05**	**0,339136993**
**BSU37860**	**SpsF**	**-2,43258531**	**0,18523321**	**2,47E-14**	**-1,02748515**	**0,490564538**	**2,61E-05**	**0,337898874**
**BSU37870**	**SpsE**	**-2,65787226**	**0,158453095**	**4,11E-15**	**-1,06532816**	**0,477863949**	**1,15E-05**	**0,318158522**
**BSU37880**	**SpsD**	**-2,62305843**	**0,162323251**	**2,26E-16**	**-0,99575799**	**0,501472331**	**3,34E-05**	**0,331897791**
**BSU37890**	**SpsC**	**-2,61478762**	**0,163256505**	**4,11E-16**	**-0,82001504**	**0,566436037**	**0,000250249**	**0,364846271**
**BSU37900**	**SpsB**	**-2,6635991**	**0,157825355**	**8,20E-16**	**-1,04984333**	**0,483020617**	**1,25E-05**	**0,320422986**
**BSU37910**	**SpsA**	**-2,65153933**	**0,159150178**	**3,06E-15**	**-1,0211078**	**0,492737849**	**1,30E-05**	**0,325944013**
BSU37920	GerQ	-0,81806786	0,567201063	1,73E-06	-1,51553669	0,349766328	1,58E-06	0,458483695
BSU37930	YwdK	0,572027307	1,486611123	0,000441519	0,434427559	1,35137452	0,024074304	1,418992821
BSU37940	YwdJ	-0,07763759	0,947608081	0,064427818	0,56264545	1,476975044	0,000921893	1,212291563
BSU37950	YwdI	0,363716219	1,286736119	0,004963973	0,52679897	1,440728979	0,006056113	1,363732549
BSU37960	YwdH	-0,49940577	0,707398089	0,134583222	-0,22892138	0,853272594	0,098204685	0,780335342
BSU37970	Ung	1,017427774	2,024306541	0,000101821	0,761421483	1,695160039	0,000242368	1,85973329
BSU37980	YwdF	-0,32710252	0,79713583	0,322493732	0,626504492	1,543819935	0,008343985	1,170477883
BSU37990	YwdE	0,509296018	1,423355481	0,003510881	-0,28345337	0,821621951	0,045413599	1,122488716
BSU38000	YwdD	0,317489642	1,246160283	0,008580093	-0,06911337	0,953223635	0,818267502	1,099691959
BSU38018	YwzG	0,343962807	1,269238164	0,054647778	-0,1526058	0,899624089	0,410959049	1,084431127
BSU38020	PdxK	-0,09634581	0,935399261	0,000114062	-0,16324267	0,893015628	0,409112366	0,914207445
BSU38030	YwdA	0,035821244	1,025140211	0,63329679	0,062913006	1,044572774	0,559743484	1,034856492
BSU38040	SacA	0,384909583	1,305777948	0,001056678	0,314203472	1,24332501	0,116779864	1,274551479
BSU38050	SacP	0,167023783	1,12273993	0,003265033	1,011056357	2,015386248	0,000130857	1,569063089
BSU38060	YwcJ	1,606236443	3,044565693	0,000963249	0,084196542	1,060097194	0,233987663	2,052331444
BSU38070	SacT	1,681567876	3,207763714	2,24E-12	0,667202757	1,587991023	1,26E-05	2,397877369
BSU38080	YwcI	1,598961304	3,029251381	3,47E-11	0,533236432	1,447172032	1,50E-05	2,238211707
BSU38090	Vpr	0,233780646	1,175912445	0,072263488	0,572865119	1,487474689	0,001246674	1,331693567
BSU38100	YwcH	-1,24967878	0,420541832	0,000154551	-0,85160707	0,554167087	0,001455777	0,487354459

BSU38110	NfrA	-0,62160655	0,649946761	0,001181528	-0,31483604	0,803942352	0,072911083	0,726944557
BSU38120	RodA	0,014547923	1,010134865	0,099464605	-0,16218067	0,893673243	0,222420185	0,951904054
BSU38130	YwcE	0,337357737	1,263440511	0,056371615	-0,36773887	0,774996196	0,149582397	1,019218354
BSU38140	QoxD	1,485183868	2,799528502	1,10E-08	0,535574954	1,449519713	0,004243495	2,124524108
BSU38150	QoxC	0,749035537	1,680668903	0,000365007	0,757856266	1,690976096	0,000136237	1,6858225
BSU38160	QoxB	0,15206583	1,11115943	0,208320185	1,044825692	2,063117076	3,97E-08	1,587138253
BSU38170	QoxA	-0,3255477	0,797995378	0,012067699	1,115648528	2,166923959	4,98E-08	1,482459669
BSU38180	YwzA	1,835077343	3,567905348	8,01E-13	-0,61453078	0,653142286	0,00907639	2,110523817
BSU38190	GalT	0,696976433	1,621103758	0,005205768	0,621434572	1,538404163	0,006980638	1,57975396
BSU38200	GalK	0,196955551	1,146276869	0,244861179	0,806994909	1,749563356	5,63E-05	1,447920112
BSU38210	GtaC	0,012574632	1,008754166	0,081774221	0,679569906	1,601662199	0,000996645	1,305208182
BSU38220	YwcC	0,499024483	1,413257627	0,003983948	0,615457081	1,532043309	0,005975146	1,472650468
BSU38229	SlrA	0,319060612	1,247517983	0,01325659	0,617653582	1,53437762	0,007732271	1,390947801
BSU38230	YwcB	0,535433277	1,449377373	0,009678817	0,692232068	1,615781445	0,000235192	1,532579409
BSU38240	YwcA	0,910445026	1,879625214	5,31E-05	0,309366866	1,239163767	0,013501794	1,559394491
BSU38250	YwbO	1,044456923	2,062589787	0,001408194	0,224542787	1,168406903	0,482768353	1,615498345
BSU38260	YwbN	1,416979194	2,670258097	1,28E-08	0,807160431	1,749764097	0,000111401	2,210011097
BSU38270	YwbM	0,469401481	1,384534958	0,00218944	1,074736852	2,10633784	4,58E-07	1,745436399
BSU38280	YwbL	1,465439895	2,761476585	5,91E-06	1,13475102	2,195806643	2,88E-07	2,478641614
BSU38290	ThiE	1,444953077	2,722539682	3,46E-06	-0,34061453	0,789704859	0,102166503	1,75612227
BSU38300	ThiM	-0,71154395	0,610666264	0,012101591	-0,41071156	0,752252257	0,060733163	0,681459261
BSU38310	YwbI	-0,45495399	0,729533437	0,070803881	-0,3332668	0,793737133	0,039277682	0,761635285
BSU38320	YwbH	-1,39847207	0,379330671	0,263316029	-1,15287667	0,4497276	0,0546655	0,414529136
BSU38330	YwbG	-1,11127404	0,462885077	0,098148812	-0,23207092	0,851411851	0,113655863	0,657148464
BSU38340	YwbF	-0,76954847	0,58660104	0,000224504	-0,38853475	0,76390506	0,12330095	0,67525305
BSU38350	YwbE	-1,28914177	0,409194379	8,68E-07	-0,7896518	0,578483694	0,000546923	0,493839037
BSU38360	YwbD	-0,47586245	0,719036819	0,159176295	0,089465668	1,063976044	0,450273111	0,891506432
BSU38370	YwbC	0,37008986	1,292433329	0,012510006	-0,18026092	0,882543368	0,503278974	1,087488348
BSU38380	YwbB	0,832347984	1,780580899	1,54E-06	-0,01811635	0,987521214	0,188512849	1,384051056

BSU38390	YwbA	-0,20827928	0,865568989	0,133191934	0,225515784	1,169195178	0,231946911	1,017382084
BSU38400	Epr	-0,31903949	0,801603386	0,268392496	-1,25995677	0,417556473	3,14E-08	0,609579929
BSU38410	SacX	-0,50208119	0,706087463	0,011234243	0,589593332	1,504822507	0,08480428	1,105454985
BSU38420	SacY	-0,42616757	0,744236177	0,029259835	0,796188812	1,736507705	0,016645058	1,240371941
BSU38430	GspA	0,577920736	1,49269637	8,29E-06	0,843328078	1,794184281	0,000104727	1,643440326
**BSU38440**	**YwaF**	**-3,03597358**	**0,121921665**	**1,72E-17**	**-1,93406697**	**0,261690423**	**4,78E-12**	**0,191806044**
BSU38450	YwaE	-0,14654372	0,90341219	0,066772454	-0,98832758	0,504061761	2,17E-05	0,703736976
BSU38460	TyrZ	-1,54000765	0,34388363	0,00459401	0,008018165	1,005573241	0,421637598	0,674728436
BSU38470	YwaD	-0,75291885	0,593401776	0,098836309	-0,28059948	0,823248865	0,163082474	0,708325321
BSU38480	YwaC	0,918248141	1,8898191	3,82E-05	1,18351505	2,271294924	2,53E-05	2,080557012
BSU38490	MenA	0,117685558	1,084992867	0,403102576	0,023368466	1,016329681	0,717682769	1,050661274
BSU38499	YwzH	0,164958332	1,121133697	0,211988752	0,674574291	1,59612572	0,00094698	1,358629709
BSU38500	DltA	0,124467685	1,090105439	0,40489731	1,0278876	2,039036499	4,28E-06	1,564570969
BSU38510	DltB	0,411783248	1,330329156	0,003043576	1,169997616	2,250113251	2,73E-07	1,790221203
BSU38520	DltC	0,265692886	1,2022133	0,035459848	1,507283793	2,842743229	2,03E-09	2,022478264
BSU38530	DltD	0,958909542	1,943840092	1,03E-05	0,904962661	1,872496032	0,000102722	1,908168062
BSU38540	DltE	-0,19216244	0,87529277	0,00192976	-0,55661693	0,679894627	0,009402386	0,777593698
BSU38550	YwaA	-0,53614628	0,689610534	0,029394464	-0,52272044	0,696058061	0,055928297	0,692834297
BSU38560	LicH	0,763938543	1,698120154	8,99E-05	0,552789392	1,466919184	0,001652653	1,582519669
BSU38570	LicA	0,117366714	1,084753104	0,182655237	0,909267009	1,878091053	1,22E-05	1,481422079
BSU38580	LicC	0,220103745	1,164817346	0,003043455	0,844305469	1,79540021	2,26E-05	1,480108778
BSU38590	LicB	0,407802549	1,326663558	9,74E-05	1,005447199	2,007565695	6,58E-06	1,667114627
BSU38600	LicR	0,65191675	1,571254362	0,000581665	0,654541925	1,574116071	3,84E-05	1,572685216
BSU38610	YxzF	2,470619083	5,542815871	1,88E-14	-0,28805554	0,819005166	0,226508983	3,180910519
BSU38620	Aag	0,154375819	1,112940001	0,076844483	0,063269337	1,044830804	0,727918757	1,078885403
BSU38630	KatX	0,013281651	1,009248646	0,027273479	0,012523618	1,008718497	0,611772808	1,008983572
BSU38640	YxlH	1,09332025	2,133645136	2,79E-07	-0,28289968	0,821937342	0,084481108	1,477791239
BSU38650	YxlG	0,23493727	1,176855565	0,062072929	0,153634477	1,112368254	0,22106114	1,144611909
BSU38660	YxlF	-0,41134918	0,751919866	0,001388773	0,130050532	1,094332031	0,36871252	0,923125948

BSU38670	YxlE	-0,06796288	0,953984097	0,746850067	-0,11872749	0,920999649	0,56824823	0,937491873
BSU38680	YxlD	-0,2725982	0,827827337	0,343928497	-0,0800682	0,946012925	0,233620867	0,886920131
BSU38690	YxlC	-0,55265596	0,681763866	0,005948868	-0,47088607	0,721521319	0,053427493	0,701642593
BSU38700	SigY	-1,03344189	0,488543224	2,59E-05	-0,31344809	0,804716159	0,193366092	0,646629691
BSU38710	YxlA	-0,30722709	0,808193642	1,18E-05	-0,71821533	0,607848912	0,006204453	0,708021277
BSU38720	YxkO	0,131914339	1,095746704	0,01894698	-0,25461047	0,838213422	0,192352932	0,966980063
BSU38730	CydD	-0,69590894	0,617320264	0,019781852	-0,06233695	0,957711512	0,623973637	0,787515888
BSU38740	CydC	-0,84066916	0,558384516	0,00071679	0,115791176	1,083569113	0,210227253	0,820976815
BSU38750	CydB	-0,42106002	0,746875656	0,037880075	0,242426327	1,182980528	0,182126819	0,964928092
BSU38760	CydA	-2,67250415	0,156854177	2,19E-09	-0,25520625	0,837867343	0,160729033	0,49736076
BSU38770	CimH	1,512296361	2,852637368	1,17E-05	0,28686694	1,219987985	0,438398904	2,036312676
BSU38780	YxkI	0,658309085	1,578231768	0,000344568	1,271570649	2,41424259	1,91E-05	1,996237179
BSU38790	YxzE	1,599811744	3,031037589	6,62E-07	0,661749933	1,582000365	0,010872596	2,306518977
BSU38800	YxkH	-0,04320694	0,970495253	0,04801481	-0,03054777	0,9790485	0,641062781	0,974771876
BSU38810	MsmX	0,656728802	1,576503969	6,01E-05	0,44009082	1,356689731	0,016963221	1,46659685
BSU38820	YxkF	0,434326679	1,351280029	0,020444503	1,052942284	2,074756869	2,04E-06	1,713018449
BSU38830	AldY	-0,35015727	0,784498573	0,08044366	-0,38234835	0,767187783	0,026165332	0,775843178
BSU38840	YxkD	-0,35849032	0,779980347	0,007806326	0,591275986	1,506578646	0,011703435	1,143279496
BSU38850	YxkC	0,741905336	1,672383061	0,000253816	-0,52114638	0,696817916	0,002268712	1,184600488
BSU38860	GalE	1,016359038	2,022807508	1,33E-07	0,924918103	1,898576463	0,000109467	1,960691986
BSU38870	YxkA	-1,80308127	0,286561904	0,00330049	-0,51794694	0,698364951	0,329509181	0,492463428
BSU38880	YxjO	-1,33293838	0,396958919	2,63E-05	0,078280027	1,05575862	0,065712239	0,72635877
BSU38890	YxjN	-0,1518712	0,900082285	0,593004863	0,023709648	1,016570061	0,471293232	0,958326173
BSU38900	YxjM	-0,91956242	0,528669347	0,000390516	0,425826211	1,343341595	0,062931745	0,936005471
BSU38910	YxjL	-1,24418392	0,422146624	5,78E-09	-0,4719317	0,72099857	0,033890922	0,571572597
BSU38920	PepT	-0,26687838	0,831115923	0,254510305	-0,27224837	0,828028098	0,425700524	0,82957201
BSU38930	YxjJ	1,554894642	2,938122691	1,54E-09	-1,69834466	0,308139459	1,21E-10	1,623131075
BSU38940	YxjI	1,127877976	2,18537063	1,95E-05	-0,12805056	0,915067097	0,625417593	1,550218864
BSU38950	YxjH	0,322091499	1,250141586	0,099988438	0,802440829	1,744049308	4,37E-05	1,497095447

BSU38960	YxjG	1,637137709	3,110481032	7,43E-09	0,518600395	1,432564796	0,01862731	2,271522914
BSU38970	YxjF	0,633415064	1,551232639	0,007078119	0,479866079	1,394614203	0,006639141	1,472923421
BSU38980	ScoB	1,093701779	2,134209465	2,84E-06	0,621344189	1,538307787	0,000455945	1,836258626
BSU38990	ScoA	0,936394828	1,913739986	9,34E-05	0,766639774	1,701302609	1,24E-05	1,807521297
BSU39000	YxjC	0,268430003	1,204496334	0,00209491	1,252027811	2,381759612	7,87E-09	1,793127973
BSU39010	YxjB	0,281211646	1,215215053	0,00034585	0,354531877	1,278570645	0,516318026	1,246892849
**BSU39020**	**NupG**	**-1,89450021**	**0,268966758**	**4,82E-11**	**-1,08514957**	**0,4713434**	**7,24E-07**	**0,370155079**
BSU39040	YxiS	0,6976517	1,621862708	0,000152902	0,690618972	1,613975828	0,000955731	1,617919268
BSU39050	KatE	1,731066069	3,319730367	5,96E-11	0,935297521	1,912284957	3,90E-06	2,616007662
BSU39060	CitH	1,054571921	2,077101794	4,46E-08	0,689058108	1,612230598	0,000521443	1,844666196
BSU39070	BglS	0,761027457	1,694697123	0,000293557	1,083347405	2,118946856	1,08E-06	1,906821989
BSU39080	LicT	0,047579122	1,033529181	0,491447131	0,028763331	1,020137296	0,637873783	1,026833238
BSU39090	YxiP	-0,82903848	0,562904279	0,001066851	-0,70596293	0,613033181	0,000484899	0,58796873
BSU39100	YxiO	-0,7910132	0,577938066	0,002974934	-0,48007403	0,716940837	0,042013277	0,647439451
BSU39110	DeaD	-1,21065166	0,432073406	1,53E-06	-0,27902736	0,824146454	0,226740018	0,62810993
BSU39120	YxiM	-0,92175291	0,527867258	0,001262025	0,519072151	1,433033316	0,002392329	0,980450287
BSU39139	YxzJ	-0,71636178	0,608630365	0,010529655	1,255697663	2,387825918	6,33E-06	1,498228142
BSU39140	YxiK	-0,53732616	0,68904678	0,004538697	0,228256902	1,171418758	0,011904835	0,930232769
BSU39150	YxiJ	0,002792491	1,001937482	0,545055771	0,665200644	1,585788802	0,012383426	1,293863142
BSU39160	YxiI	-0,06164365	0,958171864	0,509250791	0,947607847	1,928672047	0,000272437	1,443421955
BSU39170	YxzG	0,549925086	1,464009674	0,025502191	0,847005129	1,798763019	0,000166865	1,631386346
BSU39180	YxiH	0,737773173	1,667599879	2,59E-05	0,387946108	1,30852919	0,066958985	1,488064534
BSU39190	YxiG	-0,21553067	0,861229312	0,498351601	0,211772641	1,158110281	0,181896794	1,009669797
BSU39200	YxzC	-0,00367462	0,997456191	0,025669918	0,231587578	1,174126277	0,393660937	1,085791234
BSU39210	YxiF	0,046551965	1,032793599	0,070939069	0,506337613	1,420439724	0,057582389	1,226616662
BSU39220	YxxG	-0,56549036	0,675725706	0,008458103	0,289222377	1,221981443	0,157001837	0,948853574
BSU39230	WapA	-0,44708176	0,733525103	0,068296062	-0,15999318	0,895029303	0,002260553	0,814277203
BSU39240	YxxF	1,136774363	2,198888367	5,89E-06	-0,59929496	0,660076452	0,007114719	1,42948241
BSU39250	YxiE	0,535196475	1,449139494	0,015784761	-0,23937313	0,847113314	0,281659628	1,148126404

BSU39260	BglH	0,753266756	1,685605301	3,41E-06	0,522530624	1,436472749	0,002613649	1,561039025
BSU39270	BglP	0,084741031	1,060497361	0,135164981	0,99975222	1,999656533	1,22E-05	1,530076947
BSU39280	YxxE	0,49425864	1,408596732	0,038871057	0,64969968	1,568841582	0,007055278	1,488719157
BSU39290	YxxD	0,274899848	1,209910094	0,096855786	0,73696349	1,666664236	0,000329578	1,438287165
BSU39300	YxiD	-0,97047126	0,510339334	0,000559678	0,47015476	1,385258059	0,024811182	0,947798696
BSU39310	YxiC	-0,90688572	0,533335136	1,82E-06	0,1831747	1,13537958	0,246096976	0,834357358
BSU39320	YxiB	-0,64200922	0,640819865	0,001805426	0,439439379	1,356077263	0,073114521	0,998448564
BSU39330	Abn2	0,249192269	1,188541492	0,184448391	0,071455134	1,050775985	0,325443416	1,119658739
BSU39339	YxzL	1,527659517	2,883177219	8,35E-06	0,161260378	1,118263658	0,132959874	2,000720438
**BSU39340**	**HutP**	**1,506527305**	**2,841253006**	**4,37E-11**	**1,134541059**	**2,195487102**	**2,33E-07**	**2,518370054**
BSU39350	HutH	1,260146948	2,395201364	4,04E-07	1,130685783	2,189627992	1,89E-06	2,292414678
BSU39360	HutU	1,308966014	2,477639029	1,24E-08	1,015640367	2,021800107	1,66E-06	2,249719568
BSU39370	HutI	0,965927943	1,953319494	4,59E-07	0,977525582	1,969085263	2,21E-05	1,961202379
BSU39380	HutG	1,372883566	2,58987697	6,95E-08	0,771488444	1,707030037	0,000393566	2,148453504
BSU39390	HutM	1,963246917	3,899385846	3,82E-15	0,461392741	1,376870372	0,008210464	2,638128109
BSU39400	Pdp	-0,64651591	0,638821196	0,001618932	0,211470117	1,157867459	0,060350851	0,898344327
BSU39410	NupC	-0,66513458	0,630629874	0,000349564	0,753955323	1,686409995	0,000185458	1,158519934
BSU39420	Dra	-0,63872902	0,642278531	0,020730078	0,853592665	1,806995189	0,000399514	1,22463686
BSU39430	DeoR	-0,36290296	0,777598338	0,043367315	-0,02215062	0,984763624	0,219623748	0,881180981
BSU39440	YxxB	-0,69557061	0,617465054	0,159208692	0,382930274	1,303987711	0,025681196	0,960726383
BSU39450	YxeR	-0,42028148	0,747278812	0,013352941	-0,12136015	0,91932052	0,39883367	0,833299666
BSU39460	YxeQ	-0,34612707	0,786693152	0,047822177	1,319005269	2,494940255	8,92E-05	1,640816704
BSU39470	YxeP	-1,57422142	0,335824311	3,11E-06	1,599760361	3,030929638	5,11E-07	1,683376975
BSU39480	YxeO	-1,52502581	0,347473338	9,13E-06	1,001020803	2,001415634	1,82E-05	1,174444486
BSU39490	YxeN	-0,82290178	0,565303771	4,31E-05	1,324968932	2,505274927	1,98E-06	1,535289349
BSU39500	YxeM	-1,82129421	0,282967013	1,50E-07	0,928362141	1,903114207	1,26E-05	1,09304061
BSU39510	YxeL	-0,80872936	0,570884438	0,013401843	0,379729946	1,301098284	0,018938793	0,935991361
BSU39520	YxeK	-1,06026104	0,479545284	1,81E-05	0,870009743	1,827675243	0,006876303	1,153610264
BSU39530	YxeJ	1,110001481	2,158458689	0,000454083	0,022255258	1,015545767	0,911288901	1,587002228

BSU39540	YxeI	-0,30267123	0,810749857	0,268228527	-0,17215229	0,887517649	0,44751401	0,849133753
BSU39550	YxeH	0,15216357	1,111234711	0,142346669	0,001193997	1,000827958	0,587478123	1,056031335
BSU39560	YxeG	-0,42773391	0,743428598	0,108255254	0,494430815	1,408764847	0,01536723	1,076096723
BSU39570	YxeF	-0,00651798	0,995492273	0,334098638	0,461086765	1,376578388	0,033973137	1,186035331
**BSU39580**	**YxeE**	**-2,81272866**	**0,142326019**	**6,64E-17**	**-3,8177387**	**0,070916311**	**6,55E-20**	**0,106621165**
**BSU39590**	**YxeD**	**-2,8792178**	**0,135915529**	**1,10E-17**	**-3,3257443**	**0,09973583**	**4,34E-19**	**0,117825679**
BSU39600	YxeC	0,181063799	1,133719547	0,261945363	-0,86159927	0,550342147	0,000257964	0,842030847
BSU39610	YxeB	0,715934811	1,642547181	0,000389005	-0,31130152	0,80591438	0,149727793	1,224230781
BSU39620	YxeA	-0,60036357	0,659587715	0,15013754	-0,7049835	0,613449503	0,035484425	0,636518609
BSU39630	YxdM	-2,00357151	0,249381869	4,19E-07	-0,00800361	0,994467682	0,574763442	0,621924776
BSU39640	YxdL	-0,95537646	0,515707003	0,000184713	-0,6571856	0,634114118	0,006622195	0,574910561
BSU39650	YxdK	-1,12012857	0,460052826	0,027924683	-0,1082757	0,927696175	0,123602595	0,693874501
BSU39660	YxdJ	-0,24446334	0,84412974	0,016695393	0,208879542	1,155790199	0,339220347	0,99995997
BSU39670	IolJ	0,358031009	1,281675472	0,007127704	1,091005523	2,130224562	8,77E-06	1,705950017
BSU39680	IolI	0,372196288	1,294321743	0,000161834	1,376661676	2,596668194	4,97E-09	1,945494969
BSU39690	IolH	-0,00910873	0,993706201	0,396557308	1,419873661	2,675620791	2,64E-09	1,834663496
BSU39700	IolG	0,03495929	1,024527912	0,074913071	1,315293682	2,488529833	2,08E-08	1,756528872
BSU39710	IolF	0,477960875	1,392773709	0,020790121	1,246067155	2,371939405	3,19E-08	1,882356557
BSU39720	IolE	0,161546491	1,118485452	0,197703245	1,55314272	2,934556977	6,42E-10	2,026521214
BSU39730	IolD	0,214390116	1,160213343	0,197335077	1,181448301	2,268043485	5,26E-08	1,714128414
BSU39740	IolC	0,146795553	1,107107683	0,074946305	1,276021484	2,421702231	1,08E-08	1,764404957
BSU39750	IolB	-0,14517136	0,904271966	0,373312071	1,204669506	2,304844637	1,98E-06	1,604558301
BSU39760	IolA	0,027784517	1,019445406	0,660809263	1,016591134	2,023132956	6,17E-06	1,521289181
BSU39770	IolR	-0,32306453	0,799370077	0,000259968	-0,00273223	0,998107957	0,380155095	0,898739017
BSU39780	IolS	0,162391978	1,119141128	0,001040116	0,398041177	1,317717561	0,028323315	1,218429345
BSU39790	YxcE	0,170665815	1,125577828	0,012507316	0,024616701	1,017209401	0,184289772	1,071393615
BSU39800	YxcD	0,71108368	1,637033312	6,42E-08	0,80728944	1,749920572	0,008399112	1,693476942
BSU39810	CsbC	2,943487438	7,692686085	6,05E-17	0,443962401	1,360335399	0,016214821	4,526510742
BSU39820	HtpG	0,414750124	1,333067768	0,019684639	0,438305327	1,355011717	0,050168728	1,344039743

BSU39830	YxcA	1,870367448	3,656256915	3,02E-06	-0,79363138	0,576890184	0,004257255	2,116573549
BSU39840	YxbG	1,012208423	2,01699628	0,005955002	0,254626548	1,193026879	0,066191309	1,605011579
BSU39850	YxbF	-0,15814742	0,896175119	9,67E-06	-0,28483093	0,8208378	0,086698377	0,85850646
BSU39860	AldX	0,304883785	1,235319123	0,00122085	0,106228692	1,076410741	0,146358581	1,155864932
BSU39870	YxbD	1,116520192	2,168233592	2,53E-07	0,873007326	1,831476673	5,21E-05	1,999855133
BSU39880	YxbC	0,77730296	1,713923789	7,65E-06	0,798029339	1,738724478	9,25E-06	1,726324134
BSU39890	YxbB	0,394471267	1,314460935	0,064848614	1,043280353	2,060908357	8,47E-08	1,687684646
BSU39900	YxbA	0,413576612	1,33198387	0,01888653	1,016767628	2,023380473	6,10E-07	1,677682172
BSU39910	YxnB	0,224603697	1,168456234	0,220772901	1,025426575	2,035561168	6,85E-07	1,602008701
BSU39920	AsnH	-0,08768528	0,94103137	0,116202268	1,306258665	2,472993873	4,88E-08	1,707012621
BSU39930	YxaM	1,066186315	2,093890959	5,80E-05	0,889469125	1,852494328	2,26E-05	1,973192643
BSU39940	YxaL	0,561857081	1,476168164	0,001087852	0,357316447	1,28104082	0,146569403	1,378604492
BSU39950	YxaJ	0,803282207	1,745066732	0,000444134	-0,57545584	0,671074181	0,009982762	1,208070457
**BSU39960**	**YxaI**	**1,883576378**	**3,689886342**	**1,12E-08**	**1,316552739**	**2,490702549**	**4,82E-09**	**3,090294445**
BSU39970	YxaH	0,048362741	1,034090708	0,19720073	0,17890233	1,132022262	0,345791801	1,083056485
BSU39980	QdoI	0,091735261	1,065651168	0,551158522	0,246604476	1,186411491	0,210890812	1,126031329
BSU39990	QdoR	-0,17956551	0,882968878	0,107361596	-0,30502804	0,809426483	0,475677597	0,846197681
BSU40000	YxnA	1,260781733	2,396255482	0,00219824	-0,2803208	0,823407901	0,059195852	1,609831692
BSU40010	YxaD	-0,54460606	0,685578581	0,075197059	-0,74078675	0,598412931	0,004930007	0,641995756
BSU40021	YxzK	-0,81009583	0,57034397	0,000659221	-0,77892426	0,582801196	0,430763872	0,576572583
BSU40022	YxaC	-0,95447924	0,516027822	0,265653615	-0,59143159	0,663684005	0,107488993	0,589855913
BSU40030	YxaB	0,238485714	1,179753715	0,03902496	0,115092807	1,083044714	0,647264571	1,131399215
BSU40040	YxaA	-0,06759582	0,954226844	0,63936494	0,869866135	1,827493323	0,000660253	1,390860083
BSU40050	GntR	-0,616763	0,652132488	0,003622564	0,863514647	1,819465439	0,000480857	1,235798964
BSU40060	GntK	0,211608818	1,157978781	0,005682811	0,700024837	1,62453276	0,00024992	1,39125577
BSU40070	GntP	-0,09993242	0,933076698	0,131616363	0,955596291	1,939381049	2,04E-05	1,436228873
BSU40080	GntZ	0,126492847	1,091636734	0,005378923	0,359682455	1,283143439	0,073867034	1,187390086
BSU40090	AhpC	0,674311857	1,595835403	0,001819767	0,398356479	1,318005581	0,00737211	1,456920492
BSU40100	AhpF	0,340064529	1,26581321	0,016192607	0,362010682	1,285215853	0,012302301	1,275514532

BSU40110	BglA	0,386158824	1,306909121	0,076057651	0,884883024	1,846614889	0,00099454	1,576762005
BSU40120	YyzE	1,502764267	2,83385171	2,84E-11	0,07743666	1,055141628	0,630561976	1,944496669
BSU40130	YydK	-0,46204025	0,725958887	0,050276249	0,302547295	1,233320104	0,275609427	0,979639495
BSU40139	YyzN	0,344117091	1,269373906	0,07835821	0,364865422	1,287761498	0,171396289	1,278567702
BSU40140	YydJ	0,962689292	1,948939481	1,50E-07	0,61211688	1,528500347	0,001099454	1,738719914
BSU40150	YydI	0,015436987	1,010757555	0,310273057	0,696050557	1,620063718	0,000503301	1,315410637
BSU40160	YydH	0,037552087	1,026370839	0,613819084	0,592117802	1,507457995	0,013530921	1,266914417
BSU40170	YydG	0,564377311	1,478749121	0,005966029	0,793913986	1,733771756	0,000175048	1,606260438
BSU40180	YydF	3,02265218	8,126601663	1,16E-18	0,439236346	1,355886433	0,017143065	4,741244048
BSU40190	Fbp	0,409834223	1,328533146	0,010414165	0,343464935	1,268800227	0,1355836	1,298666687
BSU40200	YydD	0,298231884	1,229636491	0,014914804	0,700585245	1,625163924	0,002928842	1,427400207
BSU40210	YydC	0,998135831	1,997417383	8,04E-06	0,59968568	1,515386373	0,002686915	1,756401878
BSU40220	YydB	0,189647986	1,140485406	0,273660429	0,581707595	1,496619622	0,010404965	1,318552514
BSU40230	YydA	-0,66100293	0,632438487	0,002126013	-0,28780422	0,819147851	0,037882808	0,725793169
BSU40239	YyzF	-1,39688386	0,379748491	1,85E-06	-0,28959619	0,818131019	0,072141202	0,598939755
BSU40240	YycS	-0,59636883	0,661416607	0,093790341	-0,85122649	0,554313294	0,000169621	0,607864951
BSU40250	YycR	0,610686617	1,526985769	9,07E-05	0,636436424	1,554484707	0,001293495	1,540735238
BSU40259	YyzG	-0,00229124	0,998413093	0,388361098	-0,16571968	0,891483695	0,478481136	0,944948394
BSU40260	YycQ	0,637785091	1,555938558	0,003147109	-1,05602289	0,480956096	9,53E-06	1,018447327
BSU40270	YycP	-1,09041461	0,469626392	6,28E-06	-1,14287359	0,45285667	4,74E-06	0,461241531
**BSU40280**	**YycO**	**-1,56157878**	**0,338780144**	**1,02E-09**	**-1,14058914**	**0,453574317**	**2,21E-06**	**0,39617723**
BSU40290	YycN	-0,92485281	0,526734253	0,000237073	-0,95564187	0,515612138	1,91E-05	0,521173196
BSU40300	RapG	0,572534378	1,48713372	0,016615096	0,689666284	1,612910386	0,00387411	1,550022053
BSU40310	PhrG	0,652625259	1,572026196	0,000107458	-0,88439169	0,54171589	0,000130401	1,056871043
BSU40320	RocF	-0,29185746	0,816849695	0,243746402	0,069197443	1,049132899	0,544730297	0,932991297
BSU40330	RocE	0,300858505	1,231877249	0,171678061	0,342487993	1,267941332	0,01758439	1,249909291
BSU40340	RocD	0,300950101	1,231955463	0,005691708	0,510913568	1,424952243	0,001662133	1,328453853
BSU40350	RocR	-0,74093652	0,59835081	0,007127803	-0,29907408	0,812773869	0,249654908	0,70556234
**BSU40360**	**YyxA**	**-1,8807484**	**0,271542816**	**1,60E-11**	**-1,0218345**	**0,492489715**	**8,31E-06**	**0,382016265**

BSU40370	WalJ	0,029889858	1,02093418	0,405369487	0,928095817	1,902762922	1,29E-05	1,461848551
BSU40380	YycI	-0,26334287	0,833155177	0,146867306	0,977074059	1,968469091	7,47E-06	1,400812134
BSU40390	YycH	0,041871091	1,029448095	0,310893681	0,905007968	1,872554837	3,06E-05	1,451001466
BSU40400	WalK	0,056190815	1,039716942	0,459604549	0,89353543	1,857723037	1,60E-05	1,44871999
BSU40410	WalR	0,113566616	1,081899594	0,209104905	0,867689746	1,824738522	6,95E-05	1,453319058
BSU40420	PurA	-2,13913883	0,227015257	3,69E-11	-0,62926013	0,646507887	0,001784583	0,436761572
**BSU40430**	**YycE**	**-1,77389736**	**0,29241772**	**4,58E-12**	**-1,23718064**	**0,424200833**	**1,40E-07**	**0,358309276**
BSU40440	DnaC	-1,14700882	0,451560496	8,30E-05	-0,70968159	0,611455073	0,00086337	0,531507784
BSU40450	YycD	1,110342608	2,158969119	7,51E-06	-0,26478926	0,832320306	0,163125954	1,495644712
**BSU40460**	**YyzB**	**-2,33848988**	**0,197717178**	**1,06E-11**	**-1,34816539**	**0,392791227**	**3,60E-08**	**0,295254202**
BSU40470	YycC	0,550348772	1,464439682	0,000124355	0,557601596	1,471820359	0,00451865	1,468130021
BSU40480	YycB	0,12204524	1,088276567	0,351250103	-0,24537761	0,843594967	0,374933736	0,965935767
BSU40490	YycA	-0,97357426	0,509242857	0,006050283	-0,61640886	0,652292589	0,004725418	0,580767723
BSU40500	RplI	0,630104339	1,547676921	0,005485906	-0,27103394	0,828725408	0,274420156	1,188201165
BSU40510	GdpP	-1,28360208	0,410768631	1,13E-07	-0,02657272	0,981749786	0,728574979	0,696259209
BSU40520	YybS	-0,60816639	0,656029962	0,000152544	-0,13888118	0,908223219	0,7120778	0,78212659
BSU40529	YyzH	2,20434157	4,608641576	5,08E-07	0,45919659	1,374776018	0,095394372	2,991708797
**BSU40530**	**CotF**	**-3,20945689**	**0,108107844**	**2,20E-18**	**-2,88583526**	**0,135293528**	**2,69E-17**	**0,121700686**
BSU40540	HypR	-0,50643513	0,703959761	0,009274625	-0,63419115	0,644301945	0,006746306	0,674130853
BSU40550	PpaC	-0,20724237	0,866191326	0,069736112	-0,34808653	0,785625394	0,21064923	0,82590836
BSU40560	YybP	-0,20847228	0,865453205	0,008348195	-1,11171482	0,462743677	5,42E-05	0,664098441
**BSU40570**	**YybO**	**-3,55417976**	**0,085130519**	**2,31E-18**	**-1,41580087**	**0,37480163**	**3,11E-07**	**0,229966075**
BSU40580	YybN	0,993901645	1,991563728	1,22E-07	0,637823534	1,555980019	0,0007676	1,773771873
BSU40590	YybM	0,874758807	1,833701498	5,87E-05	0,900889602	1,867217002	2,20E-05	1,85045925
BSU40600	YybL	0,172254147	1,126817712	0,207185252	1,507201261	2,842580608	6,38E-10	1,98469916
BSU40610	YybK	1,073575064	2,10464231	1,29E-05	0,798455085	1,73923766	0,000171032	1,921939985
BSU40620	YybJ	0,4135592	1,331967794	0,018640798	0,893288344	1,857404898	0,000248004	1,594686346
**BSU40630**	**YybI**	**-1,89217395**	**0,269400802**	**1,37E-12**	**-1,07839447**	**0,473555534**	**1,75E-05**	**0,371478168**
BSU40640	YybH	-0,13126952	0,913027662	0,026116877	0,177854089	1,13120005	0,234015737	1,022113856

BSU40650	YybG	-0,00463493	0,996792466	0,482786653	-0,0065111	0,995497016	0,46083715	0,996144741
BSU40660	YybF	1,515515741	2,859010149	5,68E-10	-0,03264364	0,977627224	0,644152978	1,918318686
BSU40670	YybE	-0,38392307	0,766350846	0,166363875	0,239672634	1,180724709	0,113832277	0,973537778
BSU40680	YybD	-0,50841726	0,702993249	0,166241472	0,65173165	1,571052781	0,022681305	1,137023015
BSU40690	YybC	-0,9400661	0,521209	0,000168369	0,410091466	1,328770055	0,032224758	0,924989528
BSU40700	YybB	0,804723958	1,746811529	0,000741196	0,495018217	1,409338551	0,005547641	1,57807504
BSU40710	YybA	-0,14789946	0,902563625	0,111830333	0,519076066	1,433037205	0,048281704	1,167800415
BSU40720	YyaT	0,291383438	1,223813262	6,23E-05	0,149917217	1,109505806	0,587409809	1,166659534
BSU40730	YyaS	-0,9915486	0,502937628	0,00405134	-0,73932459	0,599019721	0,000321281	0,550978675
BSU40740	YyaR	0,52806295	1,44199179	0,026263736	0,645171526	1,563925214	0,021982885	1,502958502
BSU40750	YyaQ	-0,3260203	0,797734016	0,293620039	0,275268736	1,2102195	0,129628615	1,003976758
BSU40760	YyaP	1,410184838	2,657712113	2,17E-07	0,525499627	1,439431991	0,049253021	2,048572052
BSU40770	TetB	-0,01167249	0,99194189	0,208416621	0,389516364	1,309954193	0,026734476	1,150948041
BSU40790	YyaO	1,204350856	2,304335619	7,98E-06	-0,44690514	0,733614908	0,016178195	1,518975264
BSU40800	YyaN	-0,69131271	0,619290102	0,499407833	0,192956642	1,14310398	0,313590799	0,881197041
BSU40810	YyaM	0,461453418	1,376928282	0,012816073	0,449009045	1,365102277	0,257987319	1,371015279
**BSU40820**	**YyaL**	**-2,37938355**	**0,192191502**	**1,85E-14**	**-1,1321978**	**0,456220191**	**3,20E-06**	**0,324205846**
BSU40830	YyaK	-0,77521884	0,584299988	0,001292704	-0,0954779	0,935962157	0,59075909	0,760131073
BSU40840	YyaJ	0,149476422	1,109166864	0,48796273	0,404156286	1,323314786	0,032180541	1,216240825
BSU40850	Maa	-0,62990639	0,646218343	0,006244356	0,550431242	1,464523397	0,061414293	1,05537087
BSU40860	YyaH	-0,27889505	0,824222044	0,384199883	0,227904247	1,17113245	0,266072294	0,997677247
BSU40870	CcpB	-0,30501977	0,809431125	0,338381243	0,448453593	1,364576799	0,009334251	1,087003962
BSU40880	ExoA	-0,88806966	0,54033661	0,148247744	-0,34734661	0,786028424	0,091188168	0,663182517
BSU40890	RpsR	0,510699371	1,424740696	0,000438227	-0,14085284	0,906982843	0,583638563	1,165861769
BSU40900	SsbA	-0,26991491	0,82936846	0,137063319	0,369221589	1,291655725	0,021758782	1,060512093
BSU40910	RpsF	-0,17267156	0,887198261	0,049666283	0,347628884	1,272467566	0,026535217	1,079832913
BSU40920	YyaF	-0,82669772	0,563818328	0,000662139	0,435014448	1,351924372	0,001269714	0,95787135
BSU40930	YyaE	-0,18790319	0,877880704	0,052314094	0,781947008	1,719449814	0,000425788	1,298665259
BSU40939	YyzM	0,547230016	1,461277338	0,000373199	0,648595461	1,567641272	0,001267521	1,514459305

BSU40940	YyaD	1,066066855	2,093717586	4,60E-07	0,548608665	1,462674413	0,001236968	1,778195999
BSU40950	YyaC	-0,22144585	0,857705422	0,132221718	-0,59745556	0,660918573	0,006673553	0,759311998
BSU40960	Spo0J	0,544972529	1,458992563	0,001962213	0,918964201	1,890757315	4,93E-05	1,674874939
BSU40970	Soj	0,674922335	1,596510825	5,01E-05	0,86288327	1,818669347	4,60E-06	1,707590086
BSU40980	YyaB	1,244726487	2,369736233	5,48E-06	-0,16617666	0,891201361	0,111993551	1,630468797
BSU40990	Noc	0,749627881	1,681359096	0,00060393	0,510843994	1,424883526	0,003035755	1,553121311
BSU41000	RsmG	-0,48666736	0,713671786	0,041664618	0,604055215	1,519983028	0,002162381	1,116827407
BSU41010	GidA	-0,61309406	0,653793048	0,002181897	0,467791739	1,382990973	0,017621585	1,01839201
BSU41020	ThdF	-0,40938483	0,752944362	0,09772868	0,35110827	1,275540114	0,050211242	1,014242238
BSU41030	Jag	0,833496569	1,781999051	2,55E-06	0,453834232	1,369675593	0,016015997	1,575837322
BSU41040	SpoIIIJ	0,908490205	1,877080087	1,68E-06	0,764353835	1,698609042	5,34E-05	1,787844565
BSU41050	RnpA	0,097990941	1,070281978	0,4573015	0,368907961	1,291374962	0,041098436	1,18082847
BSU41060	RpmH	1,411370836	2,659897839	5,58E-10	-0,61246393	0,654078667	0,012157937	1,656988253
BSU04940	conE	-0,07699969	0,948027166	1	-0,31856667	0,801866141	0,673528126	0,874946654
BSU16900	ymfK/2	-0,31709478	0,802684656	0,055942532	0,292571018	1,22482108	0,066280728	1,013752868
BSU35609	tuaA/1	-0,05857287	0,960213506	0,499997489	0,43870521	1,355387349	0,050133037	1,157800427
**BSU05899**	**ydhU/2**	**-1,93231038**	**0,262009245**	**6,67E-12**	**-1,28243206**	**0,411101899**	**1,20E-07**	**0,336555572**
BSU07180	yezB	0,178743573	1,131897699	0,150220379	0,297991098	1,229431281	0,171578337	1,18066449
BSU06076	ydzW/3	1,141435703	2,206004453	3,66E-07	0,801733224	1,743194107	0,000176202	1,97459928
BSU19810	yodU	0,771328383	1,706840659	0,001729772	0,210782295	1,157315563	0,177617625	1,432078111
BSU39129	yxzI	-0,70421877	0,613774763	0,00999017	0,412765969	1,331235645	0,039897015	0,972505204
BSU16890	ymfK/1	0,322742951	1,250706219	0,120550023	-0,01686323	0,988379347	0,790920026	1,119542783
**BSU25760**	**spoIVCB**	**-2,69103889**	**0,154851913**	**8,53E-17**	**-1,65669411**	**0,317165089**	**1,55E-09**	**0,236008501**
BSU32960	yusY/1	0,190928741	1,141498324	0,038848377	-0,14583656	0,903855114	0,648176912	1,022676719
BSU03570	sfp/2	-0,40630516	0,754553362	0,065363475	0,177652229	1,131041785	0,375300378	0,942797573
**BSU39030**	**yxiT/2**	**-2,2418982**	**0,211407988**	**2,19E-14**	**-1,92733824**	**0,262913797**	**3,67E-13**	**0,237160893**
BSU21670	ypqP	-0,39195477	0,762096307	0,209650982	0,324575411	1,25229583	0,140635626	1,007196069
BSU07170	yetI	-0,37709132	0,769988437	0,05211404	0,269144896	1,205093342	0,113625363	0,987540889
BSU35230	swrAA/1	-0,37942453	0,768744169	0,092374731	0,578712674	1,49351598	0,003808469	1,131130074

BSU32970	yusY/2	-0,27781754	0,82483786	0,141047605	0,064318358	1,045590804	0,496339591	0,935214332
BSU11382	appA/2	-0,26091837	0,834556503	0,158916574	-0,26194855	0,833960784	0,805185459	0,834258644
BSU35698	yvzI	-0,10521072	0,929669149	0,322462983	1,018112019	2,025266862	0,001175096	1,477468005
BSU11381	appA/1	-0,1584233	0,896003766	0,066557203	-0,14145501	0,906604354	0,429662642	0,90130406
**BSU26559**	**yrzN**	**-2,0036009**	**0,24937679**	**1,92E-06**	**-1,48449804**	**0,357372854**	**1,17E-06**	**0,303374822**
BSU18909	yozZ	-0,1879924	0,877826422	0,435053665	-0,32614822	0,797663287	0,629985433	0,837744854
BSU06074	ydzW/2	1,329328101	2,512856178	2,41E-08	0,436993195	1,353779893	0,093389523	1,933318036
BSU26040	yqbN/2	-0,17721599	0,884408019	0,21756356	-0,49228049	0,710900479	0,165076596	0,797654249
BSU35239	swrAA/2	0,054454948	1,038466695	0,350808125	-0,07865717	0,946938631	0,837259975	0,992702663
bsrG	bsrG	0,373781704	1,29574489	1	0,080935539	1,057703703	1	1,176724297
BSU06083	ydzW/7	1,447656697	2,727646524	2,90E-08	0,034998263	1,02455559	0,776275401	1,876101057
BSU40578	yyzK	-1,36418805	0,388452999	2,15E-07	-0,9665406	0,511731659	1,15E-05	0,450092329
BSU06073	ydzW/1	2,056610478	4,160077709	1,86E-12	0,047377525	1,033384769	0,596365947	2,596731239
BSU06078	ydzW/5	1,490583068	2,810025199	1,63E-10	0,597156109	1,512731675	0,002040629	2,161378437
**BSU39029**	**yxiT/1**	**-2,17595715**	**0,221295014**	**9,45E-14**	**-1,99557944**	**0,2507672**	**6,38E-13**	**0,236031107**
BSU35699	yvzE	0,691174814	1,614597781	0,000214582	0,469455123	1,384586439	0,027111574	1,49959211
bsrF	bsrF	2,270327996	4,824327994	6,75E-15	-1,56073029	0,338979448	2,04E-09	2,581653721
BSU26558	yrzM	0,985072207	1,979412379	3,13E-05	-0,29349786	0,815921431	0,215733958	1,397666905
BSU23329	ypuC/1	0,638970702	1,557217758	0,000633373	-0,31096162	0,806104274	0,157839291	1,181661016
BSU35679	yvzH	0,09257432	1,066271122	0,476477188	-0,0748817	0,949419971	0,439543041	1,007845547
**BSU27009**	**yrzP**	**-2,14103309**	**0,226717383**	**1,39E-08**	**-1,20070032**	**0,435064039**	**1,41E-07**	**0,330890711**
BSU06038	ydzT/5	0,982793818	1,976288842	0,049544779	-0,02973463	0,97960047	0,365237102	1,477944656
BSU36668	ywzE	0,42811362	1,345473168	0,207907813	1,346253737	2,54251051	1,69E-05	1,943991839
BSU09259	yhzG	1,03116746	2,04367737	0,066039424	-1,08881061	0,470148815	0,000488276	1,256913093
BSU40573	yyzI	-1,11848759	0,460576405	2,34E-05	-0,5686172	0,674262747	0,010332522	0,567419576
BSU05344	ydzS/2	0,197669531	1,146844293	0,36459604	-0,11348602	0,924351828	0,518294575	1,035598061
BSU06037	ydzT/4	0,013690207	1,009534495	0,714690619	-0,42158832	0,74660221	0,286430897	0,878068352
BSU36669	ywzF	1,054119543	2,07645059	0,000121826	0,493018573	1,407386493	0,249046569	1,741918542
BSU18819	yozU	-0,11079953	0,9260747	0,689930877	0,985236066	1,979637211	0,004388771	1,452855956

BSU13299	ykzO	0,645884868	1,564698691	0,123906127	-0,64576996	0,639151586	0,081593187	1,101925138
BSU26748	yrdD/1	-1,61408478	0,326672116	4,53E-10	-0,28863398	0,818676856	0,19445118	0,572674486
bsrH	bsrH	4,911352359	30,09292338	2,88E-21	-0,74459752	0,596834353	0,006041612	15,34487887
BSU05343	ydzS/1	1,758748752	3,384044998	5,61E-11	-0,18211271	0,881411293	0,355840281	2,132728146
BSU26039	yqbN/1	-1,01456123	0,494978846	0,451085203	0,080935539	1,057703703	0,164202121	0,776341275
BSU17266	ymzE/1	-0,24482512	0,843918089	0,221766794	0,012624485	1,008789025	0,52869903	0,926353557
BSU06077	ydzW/4	1,379570254	2,601908546	7,95E-09	0,757029557	1,690007392	0,001695599	2,145957969
BSU26749	yrdD/2	-1,39669293	0,379798752	1,14E-07	-0,91375625	0,530801278	0,00014213	0,455300015
**BSU05890**	**ydhU/1**	**-1,97928249**	**0,253615972**	**1,28E-11**	**-1,03535761**	**0,487894929**	**8,43E-06**	**0,37075545**
BSU06079	ydzW/6	1,392438647	2,625220578	9,16E-11	0,703747887	1,628730478	0,001007623	2,126975528
BSU06033	ydzT/1	1,172062356	2,253335848	6,81E-05	-0,35013522	0,784510566	0,093898784	1,518923207
BSU03788	yczM	1,618593244	3,070754643	1,56E-09	-0,64184912	0,640890985	0,005308762	1,855822814
BSU17678	ynzJ	-0,37031197	0,773615189	0,025726808	0,55793658	1,472162145	0,024738319	1,122888667
BSU35610	tuaA/2	0,372544381	1,294634074	0,019931087	0,595171121	1,510651756	0,001310617	1,402642915
BSU06036	ydzT/3	-1,31553138	0,401777484	0,217928279	0,281612322	1,215552598	0,500366862	0,808665041
bsrE	bsrE	3,642017473	12,48407888	1,47E-19	-1,17985236	0,441396668	5,29E-05	6,462737775
BSU06034	ydzT/2	0,739773284	1,669913396	0,002322304	-0,36450504	0,776735312	0,037219153	1,223324354
LB_1008_shd5	LB_1008_shd5	0,126090344	1,091332216	0,910050676	0,080935539	1,057703703	0,766198187	1,07451796
LB_1009_-	LB_1009_-	-0,24182861	0,845672749	0,191435194	0,151944662	1,11106611	0,311344878	0,97836943
LB_1010_-	LB_1010_-	-1,98093103	0,253326335	0,000363208	0,817047009	1,761796154	0,026986449	1,007561244
LB_1019_-	LB_1019_-	0,680136363	1,602291196	0,191233574	0,520748628	1,434699534	0,27614552	1,518495365
LB_1029_shd5	LB_1029_shd5	1,024925502	2,034854306	1,39E-05	-0,84521412	0,556628189	1,45E-06	1,295741248
LB_1040_-	LB_1040_-	0,245981018	1,185898896	1	0,080935539	1,057703703	1	1,1218013
LB_1042_-	LB_1042_-	-0,67655358	0,625658112	0,732066458	-1,17923505	0,441585576	0,274177772	0,533621844
LB_1060_-	LB_1060_-	0,225025666	1,168798042	0,113264268	0,080935539	1,057703703	1	1,113250872
LB_1064_-	LB_1064_-	0,085392755	1,060976539	1	0,080935539	1,057703703	1	1,059340121
LB_1072_-	LB_1072_-	0,380393477	1,301696828	0,002154021	0,080935539	1,057703703	0,884947695	1,179700266
LB_1083_-	LB_1083_-	1,227859897	2,342192896	0,006164247	0,189206719	1,140136627	0,085585147	1,741164762
LB_1087_ncr4	LB_1087_ncr4	0,208196405	1,155243045	1	0,080935539	1,057703703	1	1,106473374

LB_1094_-	LB_1094_-	0,075666862	1,053848047	0,149348482	0,080935539	1,057703703	0,389604788	1,055775875
LB_11_-	LB_11_-	0,062543085	1,044304969	0,639904662	0,080935539	1,057703703	1	1,051004336
LB_1120_-	LB_1120_-	1,168347453	2,247541029	0,046179323	0,030035667	1,021037368	0,117070172	1,634289199
LB_1138_-	LB_1138_-	0,093591575	1,067023222	0,046103665	0,080935539	1,057703703	0,598468074	1,062363463
LB_1159_ncr4	LB_1159_ncr4	0,520248615	1,434202379	1	0,080935539	1,057703703	0,169462179	1,245953041
LB_1168_-	LB_1168_-	0,46807285	1,383260476	0,502760266	-0,32646012	0,797490855	0,17677039	1,090375666
LB_1174_-	LB_1174_-	0,186690649	1,138149954	1	0,080935539	1,057703703	0,829528884	1,097926829
LB_1177_ncr4	LB_1177_ncr4	0,037050964	1,026014389	7,61E-06	0,080935539	1,057703703	0,979331881	1,041859046
LB_1183_-	LB_1183_-	0,071018609	1,050458094	0,586384634	0,080935539	1,057703703	1	1,054080898
LB_120_-	LB_120_-	1,156572475	2,229271721	4,25E-05	-0,30044171	0,812003749	0,246934958	1,520637735
LB_1222_-	LB_1222_-	-0,87721769	0,544416352	0,000215139	-0,43784339	0,738237339	0,029453544	0,641326845
LB_1235_-	LB_1235_-	1,516003509	2,859976929	1,12E-09	-0,08492725	0,942832082	0,317151476	1,901404506
LB_1237_-	LB_1237_-	0,071510354	1,050816205	1	0,080935539	1,057703703	1	1,054259954
LB_1243_-	LB_1243_-	0,06675885	1,047361044	1	0,080935539	1,057703703	1	1,052532373
LB_1246_-	LB_1246_-	-0,3547633	0,781997932	0,91532837	0,080935539	1,057703703	0,956182658	0,919850818
LB_1263_-	LB_1263_-	-0,32426337	0,798706098	0,922200991	0,04847623	1,034172058	0,905440377	0,916439078
LB_1272_-	LB_1272_-	0,048473094	1,03416981	1	0,080935539	1,057703703	1	1,045936756
LB_1290_-	LB_1290_-	0,074852676	1,053253475	0,81973141	0,080935539	1,057703703	0,95968569	1,055478589
LB_130_shd1(	LB_130_shd1(	0,379449311	1,300845217	0,068877355	0,785534134	1,723730384	0,004687104	1,512287801
LB_1312_-	LB_1312_-	1,224733609	2,337122912	1,45E-06	0,400769782	1,320212151	0,154496552	1,828667531
LB_1320_-	LB_1320_-	0,04136599	1,029087739	0,9928945	-0,57393577	0,671781622	0,66184653	0,85043468
LB_1325_-	LB_1325_-	0,070154988	1,04982946	0,246988661	0,080935539	1,057703703	0,683359587	1,053766582
LB_1331_-	LB_1331_-	0,065672674	1,046572803	0,736862505	-0,39472536	0,76063416	0,774324226	0,903603482
LB_1348_-	LB_1348_-	1,279090003	2,426858516	0,027903055	0,123490449	1,089367286	0,666969426	1,758112901
LB_1377_-	LB_1377_-	1,385137174	2,611967913	2,66E-07	0,069627969	1,049446026	0,418402152	1,830706969
LB_1387_-	LB_1387_-	0,144047713	1,105001029	1	0,080935539	1,057703703	0,549011683	1,081352366
LB_1402_-	LB_1402_-	0,076307746	1,054316299	1	0,080935539	1,057703703	1	1,056010001
LB_1405_shd8	LB_1405_shd8	0,059692621	1,042243678	1	0,080935539	1,057703703	0,949427405	1,049973691
LB_1432_-	LB_1432_-	1,454239105	2,740120057	9,79E-09	0,469193967	1,384335824	0,008573956	2,06222794

LB_1435_-	LB_1435_-	0,113716118	1,082011714	0,990055477	0,080935539	1,057703703	1	1,069857709
LB_1465_-	LB_1465_-	0,290495304	1,223060105	1	0,080935539	1,057703703	1	1,140381904
LB_1466_-	LB_1466_-	1,072147576	2,102560883	0,000807007	-0,11332427	0,924455467	0,588457603	1,513508175
LB_1474_-	LB_1474_-	-0,22994976	0,852664582	0,280187343	0,42870978	1,346029268	0,065401315	1,099346925
LB_1499_shd8	LB_1499_shd8	1,544575834	2,917182882	3,00E-10	-0,05810621	0,960524151	0,776769978	1,938853517
LB_1509_-	LB_1509_-	-0,2683117	0,83029062	0,017661888	-0,61551412	0,652697257	0,007048608	0,741493938
LB_1523_-	LB_1523_-	0,579302141	1,494126337	0,765058511	0,066933391	1,047487763	0,703279922	1,27080705
LB_1529_-	LB_1529_-	0,591925142	1,507256699	0,399226374	0,080935539	1,057703703	0,199639891	1,282480201
LB_1540_-	LB_1540_-	0,491592279	1,405995794	0,527145154	0,080935539	1,057703703	0,826313887	1,231849749
LB_1549_-	LB_1549_-	0,047214204	1,03326779	1	0,080935539	1,057703703	1	1,045485747
LB_155_-	LB_155_-	0,199170286	1,148037913	1	0,080935539	1,057703703	0,607301725	1,102870808
LB_1565_-	LB_1565_-	0,462880639	1,378291116	1	0,080935539	1,057703703	0,097447692	1,21799741
LB_1575_-	LB_1575_-	0,298196299	1,229606161	2,30E-06	0,080935539	1,057703703	0,915566285	1,143654932
LB_1579_shd8	LB_1579_shd8	0,877351654	1,837000035	0,000299543	-0,63735903	0,642888732	0,00421514	1,239944384
LB_1597_shd8	LB_1597_shd8	1,223318869	2,334832198	6,08E-09	-1,1458479	0,451924007	1,31E-06	1,393378103
LB_16_shd2_l	LB_16_shd2_l	-1,23044615	0,426185629	2,87E-09	-1,32612464	0,398838162	2,54E-06	0,412511895
LB_1612_-	LB_1612_-	0,828448113	1,775774159	0,053711426	-0,59897948	0,660220811	0,2489547	1,217997485
LB_1616_-	LB_1616_-	0,574515069	1,489176824	0,006802749	-0,33792095	0,79118065	0,077155637	1,140178737
LB_1638_-	LB_1638_-	-1,65582782	0,317355593	3,13E-05	-0,24499961	0,843816027	0,427508816	0,58058581
LB_164_-	LB_164_-	0,0224558	1,015686943	1	0,080935539	1,057703703	0,96054692	1,036695323
LB_1650_-	LB_1650_-	0,209645951	1,156404358	0,770008039	0,080935539	1,057703703	1	1,107054031
LB_1658_-	LB_1658_-	2,707294399	6,530956953	3,70E-18	-1,09932553	0,466734645	0,00010053	3,498845799
LB_1659_-	LB_1659_-	4,34104774	20,26681869	3,93E-21	-1,31131454	0,402953553	5,22E-07	10,33488612
LB_1660_-	LB_1660_-	0,071774112	1,051008336	1	0,080935539	1,057703703	1	1,05435602
LB_1661_ncr6	LB_1661_ncr6	1,358187273	2,563628605	3,15E-05	-0,3841293	0,766241306	0,032316372	1,664934956
LB_1681_-	LB_1681_-	-0,57545313	0,671075445	0,723212393	-0,14753905	0,902789127	0,957469626	0,786932286
LB_1684_-	LB_1684_-	0,559034023	1,47328243	0,001123649	0,080935539	1,057703703	0,353481098	1,265493067
LB_169_-	LB_169_-	0,080796118	1,057601492	0,699314273	-0,00562928	0,996105685	0,511401515	1,026853589
LB_1693_shd8	LB_1693_shd8	0,946961398	1,927808032	0,00017438	-0,87893723	0,543767852	8,20E-05	1,235787942

LB_1708_shd9	LB_1708_shd9	0,333724086	1,260262345	2,11E-06	0,080935539	1,057703703	1	1,158983024
LB_1710_-	LB_1710_-	0,56993621	1,484457932	0,000681765	-0,19978194	0,870682154	0,178275156	1,177570043
**LB_1713_-**	**LB_1713_-**	**-1,30670593**	**0,404242824**	**1,94E-09**	**-1,42161062**	**0,373295334**	**9,48E-08**	**0,388769079**
LB_1716_-	LB_1716_-	0,04664155	1,032857733	0,000126357	0,080935539	1,057703703	0,018588089	1,045280718
LB_1719_-	LB_1719_-	0,963890788	1,950563259	2,92E-05	0,693641861	1,617361148	0,002358821	1,783962204
LB_1722_shd9	LB_1722_shd9	1,753794975	3,37244514	4,01E-10	-0,48792936	0,713047773	0,016390973	2,042746457
LB_1743_-	LB_1743_-	0,079476583	1,056634618	0,021707265	-0,34258879	0,788624925	0,594063604	0,922629772
LB_1784_shd1	LB_1784_shd1	0,499261671	1,413489995	1	0,080935539	1,057703703	1	1,235596849
LB_1787_-	LB_1787_-	0,074653943	1,053108398	0,804683359	0,080935539	1,057703703	1	1,055406051
**LB_1790_-**	**LB_1790_-**	**-2,95099045**	**0,129319304**	**1,40E-17**	**-1,18599669**	**0,43952079**	**3,82E-07**	**0,284420047**
LB_1805_-	LB_1805_-	-0,86747676	0,548104635	0,000680142	-1,14739905	0,45143837	0,311664391	0,499771502
LB_1815_-	LB_1815_-	0,47502598	1,38994325	0,030718828	-0,12982922	0,91393963	0,406931713	1,15194144
LB_1822_-	LB_1822_-	-0,38933085	0,763483641	0,59637133	0,080935539	1,057703703	0,82870951	0,910593672
**LB_1825_-**	**LB_1825_-**	**-2,70738528**	**0,153107273**	**2,91E-06**	**-1,26039537**	**0,417429549**	**0,000326449**	**0,285268411**
LB_183_-	LB_183_-	0,577942321	1,492718703	0,000197089	0,355519176	1,279445927	0,023880541	1,386082315
LB_1837_-	LB_1837_-	1,430626346	2,69563721	1,01E-08	0,797300337	1,737846114	2,85E-05	2,216741662
LB_1849_-	LB_1849_-	1,09188372	2,131521666	3,71E-05	-0,34124605	0,78935925	0,018010253	1,460440458
LB_1858_-	LB_1858_-	-0,06564755	0,955516342	0,193570497	-0,96918929	0,510793019	0,000556019	0,73315468
LB_1874_-	LB_1874_-	-0,14825079	0,902343858	0,116560925	-0,19164893	0,875604377	0,469901368	0,888974118
LB_1891_shd1	LB_1891_shd1	1,190673544	2,282592846	3,03E-09	-0,5042226	0,705040186	0,043023828	1,493816516
LB_1892_-	LB_1892_-	0,064317239	1,045589993	1	0,080935539	1,057703703	1	1,051646848
LB_1899_-	LB_1899_-	1,454227317	2,740097668	0,031116187	0,259843693	1,197348972	0,516383273	1,96872332
LB_1905_-	LB_1905_-	0,077343828	1,055073736	0,403485457	0,080935539	1,057703703	0,782178794	1,05638872
LB_1907_-	LB_1907_-	0,07675499	1,054643194	0,993321722	0,080935539	1,057703703	0,550674198	1,056173449
LB_1909_-	LB_1909_-	0,146244077	1,106684567	0,521965391	0,080935539	1,057703703	1	1,082194135
LB_1910_-	LB_1910_-	-0,02211598	0,984787275	1	0,080935539	1,057703703	0,977252634	1,021245489
LB_1919_-	LB_1919_-	0,07420905	1,052783694	1	0,080935539	1,057703703	1	1,055243699
LB_1941_shd1	LB_1941_shd1	0,337016137	1,26314139	0,005636152	0,772735433	1,708506141	0,000465717	1,485823765
LB_1942_ncr7	LB_1942_ncr7	0,283514221	1,217156114	0,011462372	0,080935539	1,057703703	0,376447627	1,137429908

LB_1945_-	LB_1945_-	0,45843758	1,374052931	1	0,080935539	1,057703703	0,888474203	1,215878317
LB_1950_-	LB_1950_-	-0,41189721	0,751634289	0,298407835	0,327080817	1,254472475	0,134096026	1,003053382
LB_1954_-	LB_1954_-	0,208683953	1,155633517	1	0,080935539	1,057703703	1	1,10666861
LB_1960_-	LB_1960_-	0,843919573	1,794920036	0,000117608	0,353329768	1,277505735	0,144438012	1,536212886
LB_1961_-	LB_1961_-	1,310730163	2,480670576	1,19E-06	0,890210121	1,853446049	5,11E-05	2,167058313
LB_1968_-	LB_1968_-	-0,184555	0,879920444	0,161105244	-0,36746047	0,775145761	0,054220003	0,827533102
LB_1983_-	LB_1983_-	0,07179508	1,051023612	1	0,080935539	1,057703703	1	1,054363657
LB_1987_-	LB_1987_-	0,353131051	1,277329784	0,069395238	-0,77885417	0,58282951	0,000651697	0,930079647
LB_1990_-	LB_1990_-	1,700548384	3,250244803	1,98E-06	0,080935539	1,057703703	0,52430132	2,153974253
LB_1997_-	LB_1997_-	0,368591187	1,291091445	0,00173413	0,080935539	1,057703703	0,670828718	1,174397574
LB_2006_-	LB_2006_-	0,143155083	1,10431755	0,57071505	-1,29387804	0,407853223	4,24E-08	0,756085386
LB_201_shd18	LB_201_shd18	0,069394349	1,049276099	0,911545121	0,080935539	1,057703703	0,992508464	1,053489901
LB_2024_ncr7	LB_2024_ncr7	-0,67904033	0,624580605	0,095623044	-0,17930236	0,883129948	0,078873135	0,753855277
LB_2041_-	LB_2041_-	0,520996527	1,43494608	0,17019389	0,177574612	1,130980936	0,112077027	1,282963508
LB_2091_-	LB_2091_-	0,050679885	1,035752918	0,001886128	0,351085665	1,275520128	0,45439123	1,155636523
LB_2102_-	LB_2102_-	0,374158087	1,296082979	0,931713263	0,080935539	1,057703703	0,58430901	1,176893341
LB_2104_-	LB_2104_-	1,675531348	3,194369823	1,48E-09	-0,27255189	0,827853911	0,184945317	2,011111867
LB_2109_-	LB_2109_-	-0,73965874	0,598880998	0,172153038	-1,34297441	0,39420708	0,00506103	0,496544039
LB_211_-	LB_211_-	0,602306428	1,518141672	0,189351107	0,41598061	1,334205237	0,212675506	1,426173455
LB_2112_-	LB_2112_-	0,067901123	1,048190634	0,026114048	0,080935539	1,057703703	0,53065653	1,052947169
LB_2123_shd1	LB_2123_shd1	0,076357841	1,054352909	1	0,080935539	1,057703703	0,835226433	1,056028306
LB_2126_-	LB_2126_-	0,179107418	1,132183197	1	0,080935539	1,057703703	1	1,09494345
LB_2139_shd1	LB_2139_shd1	-0,95845598	0,51460737	0,038162416	-0,04250688	0,970966296	0,773389273	0,742786833
LB_2143_ncr7	LB_2143_ncr7	1,37279923	2,589725579	5,65E-08	-0,505404	0,704463077	0,01134588	1,647094328
LB_2152_shd1	LB_2152_shd1	0,079157576	1,056401003	1	0,080935539	1,057703703	0,595075857	1,057052353
LB_2154_-	LB_2154_-	1,193220158	2,286625587	0,003758459	-1,40512186	0,377586252	8,58E-08	1,33210592
LB_2161_-	LB_2161_-	1,360302067	2,56738929	1,52E-06	-0,0447588	0,969451885	0,152442834	1,768420588
LB_2176_-	LB_2176_-	0,302555647	1,233327244	1	0,080935539	1,057703703	0,829523586	1,145515474
LB_2182_shd1	LB_2182_shd1	-1,16546181	0,445821529	1,98E-06	-1,02343154	0,491944838	1,49E-05	0,468883183

LB_2204_-	LB_2204_-	0,358328617	1,281939891	0,117162723	-1,16485184	0,446010061	0,245475939	0,863974976
LB_2207_-	LB_2207_-	-1,47523403	0,359675045	0,051189345	-0,05598427	0,961937942	0,371414085	0,660806493
LB_2208_-	LB_2208_-	-0,68466689	0,622149461	0,003125516	-1,46953794	0,36109793	4,00E-09	0,491623696
LB_2240_shd1	LB_2240_shd1	0,281677936	1,215607883	0,024706572	-1,59504412	0,331012106	1,31E-08	0,773309994
LB_2243_-	LB_2243_-	0,720450756	1,647696761	0,483094448	-0,40628956	0,754561523	0,254983597	1,201129142
**LB_2248_-**	**LB_2248_-**	**-1,90450307**	**0,26710834**	**4,31E-06**	**-1,3272489**	**0,398527476**	**1,30E-05**	**0,332817908**
LB_226_-	LB_226_-	0,240987296	1,18180114	0,718500064	-0,10569085	0,9293598	0,704603321	1,05558047
LB_2264_ncr8	LB_2264_ncr8	-1,48767347	0,356587127	0,0073147	0,614079829	1,53058146	0,143536766	0,943584293
LB_2266_-	LB_2266_-	-0,34277146	0,788525074	0,964935363	0,080935539	1,057703703	0,989181173	0,923114389
LB_227_-	LB_227_-	1,846955683	3,597402742	7,26E-07	-1,03424266	0,488272129	0,000171209	2,042837436
LB_2270_-	LB_2270_-	-2,03105324	0,244676384	5,49E-13	-0,98494567	0,505244749	2,79E-05	0,374960566
LB_2271_-	LB_2271_-	-1,75298674	0,296686926	1,21E-11	-0,94165383	0,52063571	6,60E-05	0,408661318
LB_2273_-	LB_2273_-	0,095048345	1,0681012	0,954359655	0,080935539	1,057703703	1	1,062902451
LB_2275_-	LB_2275_-	0,107643164	1,077466612	0,71454772	0,080935539	1,057703703	0,312229084	1,067585157
LB_2278_-	LB_2278_-	0,573291923	1,487914804	0,001589876	0,080935539	1,057703703	0,351302729	1,272809254
LB_2291_-	LB_2291_-	0,073347565	1,052155227	0,988467872	0,080935539	1,057703703	0,722935747	1,054929465
LB_250_-	LB_250_-	0,114975092	1,082956348	0,666479586	0,442696246	1,359142049	0,662152666	1,221049199
LB_26_-	LB_26_-	0,153723149	1,112436625	1	0,080935539	1,057703703	1	1,085070164
LB_269_shd21	LB_269_shd21	0,176214627	1,129915297	1	0,080935539	1,057703703	1	1,0938095
LB_271_shd22	LB_271_shd22	0,068780794	1,048829954	1	0,080935539	1,057703703	1	1,053266829
LB_277_-	LB_277_-	-0,65490951	0,635115327	0,61156187	-0,3045076	0,809718531	0,723456294	0,722416929
LB_288_-	LB_288_-	-0,90670346	0,533402519	0,574475231	-1,6135638	0,326790103	0,000141811	0,430096311
LB_295_-	LB_295_-	0,089082662	1,063693618	1	0,080935539	1,057703703	0,63637776	1,06069866
LB_309_-	LB_309_-	-0,34704882	0,786190685	0,109140942	0,031174134	1,021843412	0,418953981	0,904017049
LB_333_-	LB_333_-	0,074576273	1,053051704	1	0,080935539	1,057703703	0,58921568	1,055377704
LB_343_ncr10	LB_343_ncr10	0,09426163	1,067518913	1	0,080935539	1,057703703	1	1,062611308
LB_348_-	LB_348_-	0,497802792	1,41206137	0,000911836	-0,12162522	0,919151627	0,345348042	1,165606499
LB_352_-	LB_352_-	-1,3426435	0,394297508	1,14E-06	-0,23890146	0,84739031	0,143271567	0,620843909
LB_354_-	LB_354_-	1,361505876	2,569532458	6,70E-08	-0,05503038	0,962574177	0,18039871	1,766053317

LB_364_-	LB_364_-	0,17011442	1,125147716	1	0,080935539	1,057703703	1	1,09142571
LB_365_-	LB_365_-	0,09309722	1,066657658	0,267313269	0,080935539	1,057703703	0,904368496	1,062180681
LB_376_shd27	LB_376_shd27	0,68047194	1,60266394	0,000588926	-0,60309995	0,65833785	0,008522293	1,130500895
LB_378_ncr13	LB_378_ncr13	1,251119062	2,380259821	0,05325909	-0,09773095	0,934501607	0,500294469	1,657380714
LB_383_-	LB_383_-	0,860485239	1,815648888	2,57E-05	-0,1590192	0,895633749	0,35869671	1,355641319
LB_387_ncr14	LB_387_ncr14	0,015637308	1,01089791	0,948220642	0,080935539	1,057703703	0,861285083	1,034300807
LB_388_-	LB_388_-	0,467770208	1,382970332	0,905558512	0,080935539	1,057703703	1	1,220337018
LB_395_-	LB_395_-	0,301542603	1,232461519	1	0,98237582	1,975716327	0,359988839	1,604088923
LB_40_-	LB_40_-	1,019948072	2,027845968	0,11522168	-1,07231029	0,475556845	0,001661645	1,251701407
LB_404_-	LB_404_-	0,330716629	1,257637925	0,056379345	0,080935539	1,057703703	0,17589796	1,157670814
LB_427_-	LB_427_-	0,28148735	1,215447307	0,021571283	1,197352234	2,293184183	5,79E-07	1,754315745
LB_454_shd34	LB_454_shd34	0,014848645	1,010345445	1	0,080935539	1,057703703	1	1,034024574
LB_458_-	LB_458_-	-0,69286598	0,618623703	0,001701731	-0,31368046	0,80458656	0,113096114	0,711605132
LB_467_-	LB_467_-	1,107523104	2,154753899	2,81E-08	-1,79860091	0,287453219	6,23E-13	1,221103559
LB_474_-	LB_474_-	0,968995124	1,957476685	2,87E-05	-0,2926313	0,816411665	0,07031848	1,386944175
LB_499_-	LB_499_-	-0,96637814	0,511789288	0,002076762	-0,57962423	0,66913804	0,054504746	0,590463664
LB_514_-	LB_514_-	-0,62614342	0,647906071	0,074055393	-0,93912383	0,521549529	1,85E-05	0,5847278
LB_520_-	LB_520_-	0,454348188	1,370163622	0,000907708	-0,50394971	0,705173561	0,024954844	1,037668592
LB_531_-	LB_531_-	0,274082188	1,209224562	1	0,080935539	1,057703703	0,282778754	1,133464133
LB_533_-	LB_533_-	-1,03601716	0,487671932	0,36550762	0,041219156	1,028983006	0,572014885	0,758327469
LB_551_-	LB_551_-	-1,41447614	0,375145944	0,001808308	-0,4111821	0,752006951	0,047232763	0,563576448
LB_56_shd6_l	LB_56_shd6_l	0,301043428	1,23203516	0,017135189	-0,66795307	0,629399059	0,200993441	0,930717109
LB_566_-	LB_566_-	0,065379676	1,046360276	0,57182436	-0,94562845	0,519203335	4,61E-05	0,782781805
LB_570_-	LB_570_-	0,53418398	1,448122833	0,294273363	0,093943078	1,067283227	0,352262985	1,25770303
LB_574_ncr17	LB_574_ncr17	0,159024923	1,11653225	0,281348871	-0,16354591	0,892827946	0,412215119	1,004680098
LB_587_-	LB_587_-	-0,27535047	0,826249578	0,035414964	0,381813837	1,302979004	0,148682255	1,064614291
LB_612_-	LB_612_-	-0,58004238	0,668944125	0,430902427	-0,35724272	0,780655146	0,078399684	0,724799636
LB_62_-	LB_62_-	0,091904936	1,065776506	1	0,080935539	1,057703703	0,404414661	1,061740105
LB_622_-	LB_622_-	-0,02407447	0,983451311	0,971071032	0,080935539	1,057703703	1	1,020577507

LB_624_-	LB_624_-	0,8684596	1,825712503	4,37E-05	0,19826734	1,14731961	0,307720679	1,486516056
LB_63_-	LB_63_-	1,32726838	2,509271158	0,00129383	-2,22532418	0,213850698	1,27E-12	1,361560928
LB_633_-	LB_633_-	0,287435597	1,220468954	1	0,080935539	1,057703703	1	1,139086329
LB_642_-	LB_642_-	2,073698487	4,20964474	2,51E-12	-0,24428563	0,844233726	0,06481546	2,526939233
LB_645_-	LB_645_-	0,172293479	1,126848434	1	0,080935539	1,057703703	0,904039439	1,092276068
LB_650_ncr19	LB_650_ncr19	-0,26764947	0,830671829	0,48436035	-0,87397754	0,545640434	0,000485846	0,688156131
LB_666_-	LB_666_-	0,623836796	1,540967886	0,168797148	0,748556716	1,680111193	0,281688245	1,610539539
LB_681_-	LB_681_-	-0,95548441	0,515668414	0,000523669	-0,52009424	0,697326282	0,019469668	0,606497348
LB_70_-	LB_70_-	-0,11183716	0,925408875	0,002383082	-0,58357672	0,667307343	0,030158188	0,796358109
LB_700_ncr22	LB_700_ncr22	2,867884823	7,299941095	1,92E-17	-1,90857016	0,266356399	2,72E-11	3,783148747
LB_729_-	LB_729_-	0,142743535	1,104002573	0,385652895	0,036815847	1,025847193	0,808172385	1,064924883
LB_759_-	LB_759_-	0,36636068	1,28909687	0,051414348	-1,04300686	0,485314929	0,009059652	0,887205899
LB_76_-	LB_76_-	0,131315653	1,095292088	0,936990489	0,080935539	1,057703703	0,956644495	1,076497896
LB_765_ncr26	LB_765_ncr26	0,033874737	1,023758008	0,077341155	0,080935539	1,057703703	0,161522806	1,040730856
LB_767_-	LB_767_-	0,064767514	1,04591638	1	0,080935539	1,057703703	1	1,051810042
LB_77_-	LB_77_-	-0,05209999	0,964531331	0,890791733	0,080935539	1,057703703	1	1,011117517
LB_774_ncr28	LB_774_ncr28	0,956070159	1,940018162	0,000290524	-0,37493501	0,771140152	0,115365793	1,355579157
LB_78_-	LB_78_-	0,042308176	1,029760028	1	0,080935539	1,057703703	1	1,043731866
LB_786_-	LB_786_-	1,036891353	2,051801767	3,20E-07	-0,46000644	0,726983014	0,02822667	1,389392391
LB_791_-	LB_791_-	0,462855169	1,378266783	0,010390522	-0,31450506	0,804126815	0,258851999	1,091196799
LB_8_-	LB_8_-	-1,66678871	0,314953619	3,77E-05	-0,91712868	0,529561931	0,056326438	0,422257775
LB_80_-	LB_80_-	0,660342668	1,580457968	0,213896773	-2,76263152	0,147355058	4,18E-14	0,863906513
LB_807_-	LB_807_-	0,779822462	1,716919576	0,421464059	-0,98648399	0,504706304	0,263708941	1,11081294
LB_822_-	LB_822_-	-0,36701496	0,775385165	0,227197629	0,080935539	1,057703703	0,660585599	0,916544434
LB_829_-	LB_829_-	0,365283479	1,288134714	0,000109484	0,089620354	1,06409013	0,061741931	1,176112422
LB_837_ncr36	LB_837_ncr36	1,73610095	3,331336181	3,50E-10	-1,28384587	0,410699225	0,000242374	1,871017703
LB_841_-	LB_841_-	-0,02721255	0,981314477	0,012748963	-0,8038916	0,572801985	0,001451799	0,777058231
LB_846_-	LB_846_-	-1,04747706	0,483813505	0,126501642	-0,40351686	0,756013103	0,15596126	0,619913304
LB_847_-	LB_847_-	0,833736651	1,782295622	0,000622591	-1,55755077	0,33972734	1,90E-09	1,061011481

LB_849_-	LB_849_-	0,468907282	1,384060764	0,192482474	0,550862812	1,464961563	0,019456357	1,424511163
LB_855_-	LB_855_-	0,291348254	1,223783416	0,776674661	0,118804381	1,085834615	0,850659838	1,154809016
LB_863_-	LB_863_-	-0,58018357	0,668878662	0,070644456	-0,11055814	0,926229656	0,668486025	0,797554159
LB_867_-	LB_867_-	0,666204841	1,586892985	0,399607764	0,015124417	1,010538591	0,999251733	1,298715788
LB_877_-	LB_877_-	-0,69633395	0,617138435	0,011430587	-0,63876548	0,6422623	0,008919615	0,629700368
LB_904_-	LB_904_-	0,995421091	1,993662347	2,01E-07	-0,04038953	0,972392364	0,601397231	1,483027356
LB_929_-	LB_929_-	0,142368283	1,103715455	1	0,080935539	1,057703703	1	1,080709579
LB_934_-	LB_934_-	0,29488773	1,226789505	0,091334538	0,080576146	1,057440249	0,663222685	1,142114877
LB_939_-	LB_939_-	0,063464189	1,04497193	1	0,080935539	1,057703703	1	1,051337817
LB_953_-	LB_953_-	0,720105655	1,64730267	0,002485948	0,508701399	1,422768954	0,021243025	1,535035812
LB_970_-	LB_970_-	-0,49780889	0,708181524	0,013965785	-0,46213549	0,725910962	0,076249865	0,717046243
LB_98_-	LB_98_-	1,293784249	2,451703047	1,22E-08	-0,46571313	0,724113059	0,05850709	1,587908053
LB_996_-	LB_996_-	-0,00693815	0,995202387	1	0,080935539	1,057703703	1	1,026453045
M9_1000_-	M9_1000_-	0,269491438	1,205382845	1	0,080935539	1,057703703	0,7118671	1,131543274
M9_1004_-	M9_1004_-	0,149168241	1,108929955	1	0,080935539	1,057703703	1	1,083316829
M9_1008_shd	M9_1008_shd	-0,11000253	0,926586435	0,850281443	-0,04457295	0,969576776	0,858412502	0,948081605
M9_101_shd8	M9_101_shd8	2,2469473	4,746773793	0,000394285	-0,49147636	0,711296833	0,127639045	2,729035313
M9_1011_-	M9_1011_-	-0,67822928	0,624931828	0,001730191	0,25645242	1,194537728	0,101416109	0,909734778
M9_1019_-	M9_1019_-	-0,58484118	0,666722733	0,015358714	0,604523948	1,520476952	0,008416517	1,093599842
M9_1024_-	M9_1024_-	-2,59255794	0,165791513	5,70E-06	0,04492732	1,031631206	0,392859911	0,59871136
M9_1029_-	M9_1029_-	0,420749115	1,33862245	1	0,080935539	1,057703703	1	1,198163077
M9_1032_-	M9_1032_-	-0,34743719	0,785979076	0,36638038	0,224216855	1,168142968	0,331711856	0,977061022
M9_1041_-	M9_1041_-	-0,10859332	0,927491957	0,689062543	1,633314033	3,102248021	0,085987596	2,014869989
M9_1047_-	M9_1047_-	-0,06522112	0,955798813	0,087125399	0,249632357	1,188904107	0,283855609	1,07235146
M9_1052_-	M9_1052_-	0,090165276	1,064492124	1	0,080935539	1,057703703	1	1,061097914
M9_106_-	M9_106_-	-1,81915476	0,283386953	0,006476866	-0,0242737	0,983315508	0,868345217	0,633351231
M9_1060_shd	M9_1060_shd	0,078638971	1,056021327	0,004761108	0,080935539	1,057703703	1	1,056862515
M9_1069_-	M9_1069_-	-0,63483781	0,644013212	0,001174691	-1,94523436	0,25967259	1,39E-10	0,451842901
M9_1074_-	M9_1074_-	0,165096586	1,121241141	0,000383184	1,114230694	2,164795423	0,00014898	1,643018282

M9_1080_-	M9_1080_-	0,416116854	1,334331242	0,002928022	1,370205705	2,585074225	2,96E-09	1,959702734
M9_1081_-	M9_1081_-	-0,43122827	0,741630112	0,003430694	1,563982476	2,956688948	1,58E-10	1,84915953
M9_1083_-	M9_1083_-	-0,6719459	0,627659533	8,33E-05	1,178867257	2,263989485	5,18E-09	1,445824509
M9_1084_-	M9_1084_-	-0,89834612	0,536501417	0,000983636	1,159588676	2,233937271	3,91E-07	1,385219344
M9_1086_-	M9_1086_-	-0,69329463	0,618439928	0,013335945	1,229804703	2,345352387	1,33E-06	1,481896157
M9_1088_-	M9_1088_-	-0,60803253	0,656090834	0,311609095	0,080935539	1,057703703	1	0,856897269
M9_109_-	M9_109_-	-1,27927035	0,412003829	5,46E-05	-1,02650556	0,490897743	2,00E-05	0,451450786
M9_1109_-	M9_1109_-	0,065994628	1,046806384	1	0,080935539	1,057703703	0,730807937	1,052255044
M9_1141_-	M9_1141_-	0,189475543	1,140349094	0,71809088	-0,020496	0,985893697	0,720500658	1,063121395
M9_1143_-	M9_1143_-	0,098593474	1,070729068	0,767315526	0,662849543	1,583206612	0,112647687	1,32696784
M9_1147_-	M9_1147_-	0,151771298	1,110932605	1	0,080935539	1,057703703	1	1,084318154
M9_1154_shd	M9_1154_shd	0,130545196	1,094707314	0,936579939	-1,04959237	0,483104644	0,106325909	0,788905979
M9_1169_-	M9_1169_-	0,918924705	1,890705554	0,416786865	-0,56519804	0,675862634	0,440102479	1,283284094
M9_1177_-	M9_1177_-	0,089097322	1,063704426	0,135839186	0,82914829	1,776636197	0,007591488	1,420170312
M9_1183_-	M9_1183_-	0,311883061	1,241326871	0,002754029	0,080935539	1,057703703	0,041376671	1,149515287
M9_1194_-	M9_1194_-	0,289519507	1,222233142	1	0,080935539	1,057703703	0,494454921	1,139968423
M9_1199_-	M9_1199_-	0,446932159	1,363138505	0,070439048	0,651656233	1,570970657	0,000250419	1,467054581
M9_1215_-	M9_1215_-	0,11041809	1,07954104	1	0,080935539	1,057703703	1	1,068622372
M9_1216_ncr	M9_1216_ncr	0,303959605	1,23452804	1	0,080935539	1,057703703	0,986990389	1,146115872
M9_1229_ncr	M9_1229_ncr	-0,12856262	0,914742368	0,117175839	-0,52462313	0,695140677	0,021313042	0,804941522
M9_1231_-	M9_1231_-	-2,03390892	0,244192548	1,02E-11	-0,65028335	0,637155162	0,008583486	0,440673855
M9_1235_-	M9_1235_-	0,076422537	1,054400191	0,984275589	0,080935539	1,057703703	0,910769584	1,056051947
M9_125_-	M9_125_-	1,995190607	3,986687733	3,25E-12	0,906317246	1,874254994	0,000794064	2,930471363
M9_1251_-	M9_1251_-	0,074804044	1,053217971	0,93111238	0,080935539	1,057703703	1	1,055460837
M9_1258_-	M9_1258_-	0,171086262	1,125905905	1	0,080935539	1,057703703	0,955287206	1,091804804
M9_1268_-	M9_1268_-	0,225859459	1,169473733	1	0,080935539	1,057703703	1	1,113588718
M9_1276_ncr	M9_1276_ncr	0,069191525	1,049128596	1	0,080935539	1,057703703	1	1,05341615
M9_1278_-	M9_1278_-	0,112179469	1,080859851	0,30185981	0,008089851	1,005623209	0,002427399	1,04324153
M9_129_-	M9_129_-	0,288615375	1,221467413	0,54902332	0,080935539	1,057703703	0,818755688	1,139585558

M9_1302_-	M9_1302_-	0,132406818	1,096120812	1	0,080935539	1,057703703	1	1,076912258
M9_1308_-	M9_1308_-	0,230977916	1,173630213	0,015988488	-0,30009503	0,812198896	0,15857301	0,992914555
M9_1309_shd	M9_1309_shd	0,688851657	1,611999903	0,052546581	0,24634324	1,18619668	0,380872513	1,399098291
M9_1321_-	M9_1321_-	0,032295334	1,022637854	0,976245967	0,080935539	1,057703703	0,97798552	1,040170778
M9_1323_ncr	M9_1323_ncr	0,235139308	1,177020386	1	-0,08636137	0,941895313	0,840806365	1,05945785
M9_1346_shd	M9_1346_shd	0,074347184	1,0528845	0,648300657	0,080935539	1,057703703	0,450239568	1,055294102
M9_1351_shd	M9_1351_shd	1,056346869	2,079658825	0,013423122	-1,10504099	0,464889263	5,79E-05	1,272274044
M9_1357_-	M9_1357_-	1,701838172	3,253151864	0,044102343	-0,42580733	0,744422038	0,343628006	1,998786951
M9_1365_-	M9_1365_-	0,997957637	1,997170687	0,001418953	0,123224614	1,089166575	0,505891471	1,543168631
M9_1367_-	M9_1367_-	0,078854609	1,056179181	0,927627784	0,080935539	1,057703703	0,101160262	1,056941442
M9_1372_-	M9_1372_-	0,308215499	1,238175227	1	0,080935539	1,057703703	1	1,147939465
M9_138_-	M9_138_-	-1,69539319	0,308770497	7,96E-08	-0,64101801	0,641260294	0,006482729	0,475015396
M9_1417_-	M9_1417_-	0,069300169	1,049207604	1	0,080935539	1,057703703	1	1,053455654
M9_1429_-	M9_1429_-	0,069478081	1,049337	1	0,080935539	1,057703703	0,303003806	1,053520352
M9_1433_-	M9_1433_-	-0,37526576	0,770963381	0,736604219	0,080935539	1,057703703	0,686107771	0,914333542
M9_1435_-	M9_1435_-	1,885826224	3,695645114	5,64E-12	-0,47143881	0,721244937	0,082566767	2,208445025
M9_1440_shd	M9_1440_shd	0,078781868	1,056125929	0,675928837	0,080935539	1,057703703	0,818485704	1,056914816
M9_1445_shd	M9_1445_shd	1,016828486	2,023465829	1,88E-07	-0,69621026	0,617191345	0,001675632	1,320328587
M9_1456_shd	M9_1456_shd	0,07829118	1,055766782	0,399112085	-0,86083784	0,550632686	0,010470679	0,803199734
M9_1468_-	M9_1468_-	0,20100272	1,149497015	1	0,080935539	1,057703703	0,877556816	1,103600359
M9_1476_shd	M9_1476_shd	0,093814695	1,067188255	1	0,080935539	1,057703703	0,284876752	1,062445979
M9_1500_-	M9_1500_-	-0,05273145	0,964109252	0,051860899	1,295217976	2,454140721	0,000715044	1,709124987
M9_1506_-	M9_1506_-	0,342334266	1,267806233	0,126292818	1,304676767	2,470283749	0,190531985	1,869044991
M9_1516_-	M9_1516_-	0,323263459	1,251157541	0,108167476	-0,49528125	0,709423364	0,032022568	0,980290452
M9_1520_shd	M9_1520_shd	-0,60805888	0,656078849	0,014703065	-1,46352297	0,362606586	1,69E-09	0,509342717
M9_1537_shd	M9_1537_shd	0,457267254	1,37293874	0,024775876	-0,68787564	0,620767256	0,000530986	0,996852998
M9_1539_-	M9_1539_-	0,411094462	1,329694169	1	0,080935539	1,057703703	0,834625682	1,193698936
M9_1554_shd	M9_1554_shd	0,322412587	1,250419851	1	0,080935539	1,057703703	1	1,154061777
M9_1575_-	M9_1575_-	0,620305042	1,537200171	0,466079995	-0,51418161	0,700190015	0,50628083	1,118695093

M9_1576_-	M9_1576_-	0,879813931	1,840137957	0,014599779	-0,95429822	0,516092575	0,389985941	1,178115266
M9_159_shd1	M9_159_shd1	0,068731435	1,048794071	0,954678163	0,080935539	1,057703703	0,866142262	1,053248887
M9_1603_-	M9_1603_-	-0,70718074	0,612515926	0,013626456	-0,21024363	0,864391247	0,404794301	0,738453587
**M9_1613_shc**	**M9_1613_shc**	**-1,36685184**	**0,387736421**	**0,035874792**	**-2,11982405**	**0,23007497**	**5,60E-09**	**0,308905696**
M9_1620_-	M9_1620_-	-0,23127623	0,851880973	0,058390951	-0,89952718	0,536062388	7,83E-05	0,69397168
M9_1623_-	M9_1623_-	0,135497335	1,098471421	1	0,080935539	1,057703703	1	1,078087562
M9_1627_-	M9_1627_-	0,555648852	1,46982954	0,635384187	0,080935539	1,057703703	0,720585433	1,263766622
M9_1639_-	M9_1639_-	1,025752844	2,036021567	0,000210915	-0,77535231	0,584245934	0,000993734	1,31013375
**M9_165_-**	**M9_165_-**	**-1,98278689**	**0,25300067**	**1,34E-10**	**-1,0563686**	**0,480840859**	**3,27E-06**	**0,366920765**
M9_1654_ncr	M9_1654_ncr	1,15977442	2,234224905	1,21E-07	0,080935539	1,057703703	0,749418978	1,645964304
M9_1674_-	M9_1674_-	0,076886952	1,054739666	0,984980836	0,080935539	1,057703703	0,862414427	1,056221684
M9_1680_-	M9_1680_-	0,264163514	1,200939533	0,024543726	-0,21735074	0,860143489	0,357110415	1,030541511
M9_1681_-	M9_1681_-	-0,87712831	0,544450085	0,002933039	-0,53445665	0,690418657	0,012341084	0,617434371
M9_1685_-	M9_1685_-	0,090492149	1,064733335	0,601846339	0,080935539	1,057703703	0,667396443	1,061218519
M9_1688_-	M9_1688_-	0,134942462	1,09804902	0,32008942	-0,5192207	0,697748632	0,0164052	0,897898826
M9_1704_-	M9_1704_-	0,11015941	1,079347492	0,210133944	0,080935539	1,057703703	0,643813838	1,068525598
M9_1709_-	M9_1709_-	0,044055698	1,031008122	0,97627372	0,080935539	1,057703703	1	1,044355913
M9_1713_-	M9_1713_-	-0,64724841	0,638496929	0,015777702	-1,4445999	0,367394033	1,30E-08	0,502945481
M9_1716_-	M9_1716_-	0,067472769	1,047879459	0,868929612	0,080935539	1,057703703	0,934396831	1,052791581
M9_1718_-	M9_1718_-	0,96570297	1,953014919	0,000116609	-0,2876184	0,819253366	0,185253437	1,386134142
M9_1725_-	M9_1725_-	-0,00138054	0,99904354	0,409911527	0,080935539	1,057703703	0,803012102	1,028373622
M9_1732_-	M9_1732_-	0,108741296	1,078287056	1	0,080935539	1,057703703	1	1,06799538
M9_1736_-	M9_1736_-	0,068248709	1,048443203	0,856226791	0,080935539	1,057703703	0,95225599	1,053073453
M9_1745_-	M9_1745_-	0,582535318	1,49747853	0,00094591	-0,84208188	0,557838	0,000316812	1,027658265
M9_1746_ncr	M9_1746_ncr	0,070937084	1,050398735	0,416707997	-2,31430521	0,201059553	9,34E-09	0,625729144
M9_1752_-	M9_1752_-	0,08212441	1,058575677	1	0,080935539	1,057703703	0,730213199	1,05813969
M9_1754_-	M9_1754_-	0,327027306	1,254425947	1	0,080935539	1,057703703	1	1,156064825
M9_1777_-	M9_1777_-	0,432899087	1,349943557	0,529925644	1,070942008	2,100804641	0,076217328	1,725374099
M9_1780_-	M9_1780_-	-0,58677812	0,6658282	0,266314636	-0,54775297	0,684084775	0,536756756	0,674956487

M9_1781_-	M9_1781_-	0,100796234	1,072365145	0,880636725	-0,51550565	0,699547705	0,6926278	0,885956425
M9_1789_-	M9_1789_-	0,426091779	1,343588898	0,283471734	-0,60971371	0,655326732	0,016459981	0,999457815
M9_1790_-	M9_1790_-	1,609344273	3,051131316	6,87E-09	0,113287938	1,081690629	0,276009795	2,066410973
M9_1791_-	M9_1791_-	0,085146025	1,060795106	0,001637609	0,080935539	1,057703703	0,967751816	1,059249405
M9_1801_-	M9_1801_-	0,047723405	1,033632548	1	-0,67694933	0,625486506	0,536937878	0,829559527
M9_1827_-	M9_1827_-	-0,76715258	0,587576017	0,000226462	-2,05612079	0,240461731	6,40E-13	0,414018874
M9_1837_-	M9_1837_-	1,407311724	2,65242457	0,000974104	-0,25110601	0,840252008	0,147963441	1,746338289
M9_1849_-	M9_1849_-	-0,04901106	0,966598687	0,594765018	0,080935539	1,057703703	1	1,012151195
M9_185_ncr6	M9_185_ncr6	0,696512588	1,620582636	8,14E-05	-1,08468233	0,471496076	8,57E-06	1,046039356
M9_1852_-	M9_1852_-	0,290186705	1,222798514	1	0,080935539	1,057703703	0,945686965	1,140251109
M9_1863_shd	M9_1863_shd	-1,47190929	0,360504885	2,66E-08	-0,99426685	0,50199091	1,67E-05	0,431247898
M9_1868_-	M9_1868_-	-0,79309738	0,577103752	0,001481277	-0,95102403	0,517265174	4,07E-05	0,547184463
M9_1873_-	M9_1873_-	1,813183605	3,514169077	8,34E-12	-0,06526151	0,955772055	0,870135557	2,234970566
M9_1888_-	M9_1888_-	0,111034737	1,080002563	1	0,080935539	1,057703703	0,934415294	1,068853133
M9_19_-	M9_19_-	0,103462965	1,074349178	1	0,080935539	1,057703703	1	1,066026441
M9_191_-	M9_191_-	1,169967838	2,250066808	9,97E-08	0,976252548	1,967348508	1,65E-05	2,108707658
M9_1914_shd	M9_1914_shd	1,267618229	2,40763756	1,09E-07	-0,60307486	0,658349297	0,005456798	1,532993429
M9_1922_shd	M9_1922_shd	-0,02492764	0,982869898	0,434374611	-1,45000502	0,36602015	3,37E-08	0,674445024
M9_1936_-	M9_1936_-	1,473592597	2,777125927	7,76E-06	-0,42367834	0,745521395	0,10951265	1,761323661
M9_1940_-	M9_1940_-	1,406121682	2,650237556	0,006329993	0,564583647	1,478960629	0,159373116	2,064599092
M9_1946_shd	M9_1946_shd	-0,80546773	0,572176549	0,000124184	-2,43265724	0,185223975	3,70E-14	0,378700262
M9_1965_-	M9_1965_-	0,026816915	1,018761903	0,789268689	0,24229769	1,182875053	0,529278013	1,100818478
M9_1966_-	M9_1966_-	0,075300566	1,053580512	1	0,080935539	1,057703703	1	1,055642108
M9_1974_-	M9_1974_-	0,070895332	1,050368337	0,745653149	0,080935539	1,057703703	0,269766727	1,05403602
M9_1989_-	M9_1989_-	-0,14792592	0,902547068	0,594246407	0,080935539	1,057703703	0,234348902	0,980125386
M9_1995_-	M9_1995_-	-0,84144062	0,558086009	0,378107401	-2,10569394	0,232339453	0,036381592	0,395212731
M9_1996_-	M9_1996_-	0,830125105	1,777839523	0,059671396	0,002316052	1,001606654	0,616796488	1,389723089
**M9_2010_-**	**M9_2010_-**	**-1,09411424**	**0,468423629**	**0,002964147**	**-1,81774451**	**0,283664101**	**1,65E-08**	**0,376043865**
M9_2013_shd	M9_2013_shd	2,29097486	4,893866883	9,95E-15	-0,00261506	0,998189017	0,249317366	2,94602795

M9_2014_-	M9_2014_-	1,321682734	2,499574858	0,042016439	0,080935539	1,057703703	0,681627951	1,77863928
M9_2017_-	M9_2017_-	0,286964432	1,220070431	5,63E-05	0,080935539	1,057703703	0,901899336	1,138887067
M9_2020_shd	M9_2020_shd	0,421170017	1,339013046	0,00546743	0,480499826	1,395226963	0,039280832	1,367120004
M9_2021_-	M9_2021_-	0,041875661	1,029451356	1	0,080935539	1,057703703	1	1,04357753
M9_2047_-	M9_2047_-	0,072959772	1,051872448	0,651391439	-0,13695168	0,90943871	0,260755755	0,980655579
M9_2053_-	M9_2053_-	-0,07601971	0,948671352	0,09551517	-0,902981	0,534780587	0,001959638	0,74172597
M9_2078_-	M9_2078_-	0,040019804	1,02812794	0,983241513	0,080935539	1,057703703	0,675055266	1,042915822
M9_2080_-	M9_2080_-	0,077557781	1,055230216	1	0,080935539	1,057703703	1	1,05646696
M9_2084_-	M9_2084_-	0,08051647	1,05739651	1	0,080935539	1,057703703	1	1,057550107
M9_209_shd1	M9_209_shd1	0,842177598	1,792754076	4,88E-05	-0,1137288	0,924196288	0,699554072	1,358475182
**M9_2098_shc**	**M9_2098_shc**	**-2,03980277**	**0,243196981**	**2,16E-11**	**-1,03012925**	**0,489666279**	**4,13E-05**	**0,36643163**
M9_2105_-	M9_2105_-	0,050702846	1,035769403	0,964401258	0,080935539	1,057703703	1	1,046736553
M9_2107_-	M9_2107_-	1,029882907	2,041858522	3,30E-06	-0,03739096	0,974415535	0,820211796	1,508137029
M9_2126_-	M9_2126_-	1,21801938	2,326271333	1,72E-06	1,090424675	2,129367078	8,41E-08	2,227819206
M9_213_-	M9_213_-	1,229901598	2,345509913	1,06E-09	-0,235035	0,849664383	0,358510174	1,597587148
M9_2130_-	M9_2130_-	0,734090234	1,663348228	0,503699465	-0,80937307	0,570629773	0,493545001	1,116989001
M9_2135_-	M9_2135_-	1,640522052	3,117786312	7,24E-06	-1,1225787	0,459272178	0,001531039	1,788529245
M9_214_-	M9_214_-	0,077758548	1,055377073	1	0,080935539	1,057703703	1	1,056540388
M9_2149_-	M9_2149_-	-1,04506444	0,484623262	0,000216934	-0,78877717	0,578834505	0,000214881	0,531728883
M9_2173_-	M9_2173_-	-0,68031866	0,624027426	0,282121906	-1,2093856	0,432452746	0,011319105	0,528240086
M9_218_shd1	M9_218_shd1	-0,8867974	0,540813325	0,00013283	-0,29495567	0,815097379	0,208920992	0,677955352
M9_219_-	M9_219_-	0,393080906	1,313194767	1	0,080935539	1,057703703	1	1,185449235
M9_2197_-	M9_2197_-	1,674296765	3,191637418	7,51E-08	-0,33443646	0,793093872	0,111723363	1,992365645
M9_2206_shd	M9_2206_shd	-1,74030551	0,299306288	0,035509778	0,535224952	1,449168098	0,066428495	0,874237193
M9_2218_-	M9_2218_-	0,208213959	1,155257102	1	0,080935539	1,057703703	1	1,106480403
M9_2220_-	M9_2220_-	0,05256615	1,037108009	0,655155303	0,080935539	1,057703703	1	1,047405856
M9_2228_-	M9_2228_-	-0,60625108	0,656901481	0,024137492	-1,4176351	0,374325412	2,42E-08	0,515613446
M9_2229_shd	M9_2229_shd	-0,66421338	0,631032679	0,005283025	-0,98004248	0,506964811	8,55E-05	0,568998745
M9_2231_-	M9_2231_-	1,1119221	2,161334097	0,076850169	0,062115129	1,043995236	0,478676682	1,602664666

M9_2237_sho	M9_2237_sho	0,990397378	1,986732144	3,92E-05	-0,39040839	0,762913612	0,046146112	1,374822878
M9_2252_-	M9_2252_-	0,015174587	1,010573733	0,475433216	0,788901546	1,727758463	6,00E-05	1,369166098
M9_2255_-	M9_2255_-	0,298330352	1,22972042	1	0,080935539	1,057703703	0,803838509	1,143712061
M9_2265_-	M9_2265_-	0,350873488	1,275332551	0,074536149	0,256321106	1,194429006	0,174599009	1,234880778
M9_227_-	M9_227_-	0,40894449	1,327714072	1	0,080935539	1,057703703	0,708522102	1,192708887
M9_2270_-	M9_2270_-	0,428612141	1,345938174	0,016779011	0,080935539	1,057703703	0,8480111	1,201820939
M9_2272_-	M9_2272_-	0,255460156	1,193716425	1	0,080935539	1,057703703	1	1,125710064
M9_2275_-	M9_2275_-	-1,39938711	0,379090154	1,44E-08	-0,28563875	0,820378306	0,331023062	0,59973423
M9_228_-	M9_228_-	0,667320825	1,588120987	0,086753551	-1,06168872	0,479070964	0,04091736	1,033595975
M9_2283_ncr	M9_2283_ncr	0,407075454	1,325995109	0,499178881	0,080935539	1,057703703	0,514279204	1,191849406
M9_2293_-	M9_2293_-	0,966764792	1,954452864	8,68E-05	-0,6345905	0,644123617	0,083608003	1,299288241
M9_230_-	M9_230_-	0,07339491	1,052189756	0,978789272	0,080935539	1,057703703	0,049614818	1,05494673
M9_2301_-	M9_2301_-	1,532884763	2,89363863	6,19E-07	0,080935539	1,057703703	0,73212842	1,975671167
M9_2303_sho	M9_2303_sho	0,174578982	1,12863499	0,889721132	0,080935539	1,057703703	0,211127178	1,093169347
M9_2305_-	M9_2305_-	0,04447916	1,03131079	0,962171894	0,080935539	1,057703703	0,930138444	1,044507247
M9_2306_-	M9_2306_-	1,621365999	3,076662085	0,000795838	-0,3679882	0,774862267	0,043257944	1,925762176
M9_2311_-	M9_2311_-	0,566996679	1,481436388	0,421302407	0,080935539	1,057703703	1	1,269570045
M9_2313_-	M9_2313_-	2,682632248	6,420262336	3,12E-11	-0,24285446	0,845071635	0,331021771	3,632666985
M9_2320_-	M9_2320_-	0,932370902	1,908409671	0,315308335	0,080935539	1,057703703	0,463628119	1,483056687
M9_2326_sho	M9_2326_sho	-0,18815313	0,877728627	0,858629767	0,080935539	1,057703703	1	0,967716165
M9_2331_-	M9_2331_-	-0,07702961	0,948007506	0,60346283	-0,77460634	0,584548107	0,547799028	0,766277807
M9_2333_-	M9_2333_-	-0,10809697	0,927811114	0,785108858	-0,94440706	0,519643077	0,522557018	0,723727096
M9_2347_-	M9_2347_-	0,386940133	1,307617086	0,141710514	0,080935539	1,057703703	0,525326402	1,182660394
M9_2352_-	M9_2352_-	0,350262486	1,274792544	1	0,080935539	1,057703703	0,197936482	1,166248124
M9_2357_-	M9_2357_-	1,490926076	2,810693377	0,006191235	-0,13538902	0,910424305	0,151135792	1,860558841
M9_2361_-	M9_2361_-	1,445233902	2,723069684	6,52E-06	0,306869905	1,237020925	0,04451992	1,980045305
M9_2363_-	M9_2363_-	-0,70787057	0,612223119	0,501183349	0,368184711	1,290727734	0,371933006	0,951475427
M9_2372_ncr	M9_2372_ncr	0,50178277	1,415962218	0,007222934	1,149869107	2,218937616	3,49E-08	1,817449917
M9_238_-	M9_238_-	0,066225335	1,046973797	0,65464318	0,080935539	1,057703703	1	1,05233875

M9_2380_-	M9_2380_-	0,315300903	1,244271144	0,226695445	-0,29543755	0,814825167	0,245145762	1,029548156
M9_2388_-	M9_2388_-	0,061817853	1,043780137	0,948694363	0,080935539	1,057703703	0,894892534	1,05074192
M9_2394_-	M9_2394_-	0,126492845	1,091636732	0,912371128	0,080935539	1,057703703	1	1,074670218
M9_2396_-	M9_2396_-	1,250874476	2,37985632	2,93E-05	0,144373849	1,105250854	0,520086194	1,742553587
M9_2406_-	M9_2406_-	0,251877054	1,190755369	1	0,080935539	1,057703703	1	1,124229536
M9_2408_-	M9_2408_-	0,101772162	1,073090805	1	0,080935539	1,057703703	0,918573549	1,065397254
M9_2411_-	M9_2411_-	0,16881585	1,124135426	1	0,080935539	1,057703703	1	1,090919565
**M9_2413_-**	**M9_2413_-**	**-1,17655395**	**0,442406978**	**0,216125969**	**-2,13092577**	**0,228311309**	**1,04E-05**	**0,335359144**
M9_2423_-	M9_2423_-	-1,03974605	0,486413086	0,001558834	-1,54209752	0,343385847	2,43E-07	0,414899467
M9_2432_-	M9_2432_-	-0,18118487	0,881978342	0,157727741	-1,21185627	0,431712787	4,99E-07	0,656845565
M9_2436_-	M9_2436_-	0,197257415	1,146516737	0,744353735	0,737485654	1,667267572	0,545176323	1,406892154
M9_2439_shd	M9_2439_shd	-0,49537818	0,709375702	0,072148695	-1,96469162	0,256193962	5,91E-13	0,482784832
M9_2459_-	M9_2459_-	0,235549068	1,177354735	0,956383659	0,080935539	1,057703703	0,892402053	1,117529219
M9_2463_shd	M9_2463_shd	0,584596496	1,499619506	0,001809793	-0,91291484	0,531110944	0,000351215	1,015365225
M9_251_-	M9_251_-	0,137984158	1,100366527	0,218879605	0,583399788	1,498376095	0,117340691	1,299371311
M9_256_-	M9_256_-	0,433767294	1,35075619	0,01441741	0,104575598	1,075178056	0,024865265	1,212967123
M9_264_-	M9_264_-	-1,94371121	0,259946889	8,38E-09	-0,8586687	0,551461205	0,000243269	0,405704047
M9_266_shd2	M9_266_shd2	0,070492991	1,050075449	1	0,080935539	1,057703703	0,967143643	1,053889576
M9_279_-	M9_279_-	0,09219886	1,065993662	1	0,080935539	1,057703703	0,419211651	1,061848683
M9_28_-	M9_28_-	0,067476701	1,047882315	1	0,080935539	1,057703703	1	1,052793009
M9_284_-	M9_284_-	0,828151161	1,775408686	9,56E-06	0,080935539	1,057703703	0,208607341	1,416556195
M9_293_-	M9_293_-	0,29010997	1,222733477	0,16009033	-0,16427656	0,892375887	0,450809108	1,057554682
M9_308_-	M9_308_-	0,498251593	1,41250071	0,493804167	-0,33718337	0,791585247	0,421739537	1,102042979
M9_310_-	M9_310_-	0,035270724	1,024749101	0,840033812	0,080935539	1,057703703	0,696681099	1,041226402
M9_326_-	M9_326_-	-0,11787148	0,921546275	0,085596093	-0,45753901	0,72822743	0,024409848	0,824886852
M9_33_-	M9_33_-	0,033036971	1,023163689	0,424020782	0,080935539	1,057703703	0,834216377	1,040433696
M9_333_-	M9_333_-	2,715752094	6,569356663	8,82E-16	-0,48901186	0,712512952	0,023394181	3,640934807
M9_348_-	M9_348_-	0,279490447	1,213766112	0,001819539	0,309489638	1,239269223	0,594156645	1,226517668
M9_351_-	M9_351_-	-0,20257662	0,868997165	0,056093146	0,289204639	1,221966419	0,238802521	1,045481792

M9_357_-	M9_357_-	-1,92934556	0,262548242	0,016189337	0,080935539	1,057703703	0,042507214	0,660125973
M9_359_-	M9_359_-	0,830497083	1,778297972	0,000100894	-0,28811161	0,818973337	0,132545344	1,298635655
M9_37_-	M9_37_-	-0,13220976	0,912432815	0,88161184	0,080935539	1,057703703	0,976160363	0,985068259
M9_373_-	M9_373_-	1,644656001	3,12673294	0,007691097	-0,25161772	0,83995403	8,62E-07	1,983343485
M9_384_-	M9_384_-	0,069204632	1,049138127	0,946392859	0,080935539	1,057703703	0,590024527	1,053420915
M9_385_-	M9_385_-	0,734039687	1,663289952	6,16E-05	-0,21547587	0,861262027	0,494532222	1,262275989
M9_390_-	M9_390_-	0,045215933	1,031837606	1	-0,5197506	0,697492399	0,778503384	0,864665003
M9_396_-	M9_396_-	0,236809735	1,17838399	0,042925069	0,430504187	1,347704485	0,102774187	1,263044237
M9_40_-	M9_40_-	0,677400737	1,599255824	0,057857064	-0,05331731	0,96371782	0,344751437	1,281486822
M9_402_-	M9_402_-	-0,01420552	0,990201801	0,946901065	0,501845161	1,416023454	0,596415988	1,203112627
M9_404_-	M9_404_-	0,270547336	1,206265379	0,055426175	0,038894512	1,02732632	0,582889738	1,116795849
M9_42_ncr2_	M9_42_ncr2_	0,041443305	1,02914289	1	0,080935539	1,057703703	0,940227406	1,043423297
M9_425_-	M9_425_-	-1,33156688	0,397336468	0,323521218	0,080935539	1,057703703	0,532886857	0,727520086
M9_429_-	M9_429_-	0,074173466	1,052757728	1	0,080935539	1,057703703	1	1,055230716
M9_433_-	M9_433_-	0,82482351	1,771318333	1,33E-05	-0,08846866	0,940520527	0,262085189	1,35591943
M9_435_-	M9_435_-	0,164772713	1,12098946	0,101021335	-0,0553783	0,96234207	0,770103121	1,041665765
M9_440_shd3	M9_440_shd3	0,718385429	1,645339646	0,000326215	1,087877905	2,125611451	4,65E-06	1,885475549
M9_462_-	M9_462_-	0,49847681	1,412721231	0,076586967	0,335339279	1,261674083	0,426610138	1,337197657
M9_47_-	M9_47_-	1,068440508	2,097165195	1,16E-06	0,982699642	1,976159839	6,12E-06	2,036662517
M9_482_-	M9_482_-	1,015480183	2,021575637	2,15E-06	0,737527516	1,66731595	0,005490024	1,844445793
M9_49_-	M9_49_-	0,300898164	1,231911113	0,750290446	0,080935539	1,057703703	1	1,144807408
M9_492_-	M9_492_-	0,303121788	1,23381132	1	0,070260956	1,049906574	0,524521737	1,141858947
M9_510_-	M9_510_-	-0,0448208	0,969410223	0,53101087	-0,63073527	0,645847177	0,001410921	0,8076287
M9_519_-	M9_519_-	0,136459834	1,099204514	0,501114662	0,080935539	1,057703703	0,493008934	1,078454109
M9_526_-	M9_526_-	-0,50782554	0,703281643	0,098507944	-0,73633215	0,600263501	1,58E-05	0,651772572
M9_53_shd5_	M9_53_shd5_	0,074856642	1,053256371	1	0,080935539	1,057703703	1	1,055480037
M9_538_-	M9_538_-	-0,68295822	0,622886745	0,003848452	-0,07292764	0,950706785	0,209882847	0,786796765
M9_543_shd3	M9_543_shd3	0,065775602	1,046647473	1	0,080935539	1,057703703	1	1,052175588
M9_559_shd3	M9_559_shd3	1,323405739	2,502561874	1,90E-08	-1,22850128	0,42676055	1,38E-07	1,464661212

M9_567_-	M9_567_-	1,249659343	2,377852693	0,015410985	-0,83925837	0,55893082	0,206872049	1,468391756
M9_57_-	M9_57_-	0,433636856	1,35063407	0,000201595	0,080935539	1,057703703	0,143081886	1,204168887
M9_587_-	M9_587_-	0,207243253	1,154480058	0,026006724	0,072063984	1,05121953	0,889937283	1,102849794
M9_593_-	M9_593_-	0,210072461	1,156746281	1	0,080935539	1,057703703	1	1,107224992
M9_601_-	M9_601_-	0,566424737	1,480849204	0,000692207	-0,0529119	0,963988674	0,130430626	1,222418939
M9_61_-	M9_61_-	0,04979527	1,035118022	1	0,118637869	1,085709298	0,749519809	1,06041366
M9_621_-	M9_621_-	0,159872092	1,117188085	0,029000687	0,080935539	1,057703703	0,512657461	1,087445894
M9_624_-	M9_624_-	-1,27703352	0,412643117	9,10E-07	-0,53614998	0,689608765	0,001881974	0,551125941
M9_635_ncr1	M9_635_ncr1	1,822046962	3,535825218	6,87E-12	-0,45581542	0,729097967	0,252523469	2,132461593
M9_636_-	M9_636_-	0,346789732	1,271727643	0,000633656	-0,38943867	0,763426586	0,032652978	1,017577115
M9_659_shd4	M9_659_shd4	-1,14564034	0,45198903	0,028994242	-1,09964725	0,466630577	0,001951821	0,459309804
M9_670_-	M9_670_-	0,12741387	1,092333862	1	0,080935539	1,057703703	1	1,075018783
M9_683_-	M9_683_-	-0,9510271	0,517264075	0,000257908	-0,53926049	0,688123546	0,058193988	0,60269381
M9_686_-	M9_686_-	0,325508451	1,253105995	0,011492722	-0,63983165	0,641787834	0,020891438	0,947446914
M9_692_-	M9_692_-	-1,0210999	0,492740549	3,11E-06	0,217501217	1,162717986	0,202438886	0,827729267
M9_694_-	M9_694_-	-0,48801535	0,713005273	0,019019588	-0,94479291	0,519504117	1,13E-05	0,616254695
M9_695_-	M9_695_-	0,136655685	1,099353745	0,266246164	-0,34648653	0,786497164	0,563756862	0,942925455
M9_699_-	M9_699_-	0,581417431	1,496318643	0,009922439	1,116723188	2,168538697	8,03E-07	1,83242867
M9_7_-	M9_7_-	0,498355665	1,412602608	1	0,080935539	1,057703703	1	1,235153156
**M9_702_-**	**M9_702_-**	**-1,77478246**	**0,292238375**	**2,71E-07**	**-1,22883222**	**0,426662666**	**3,12E-06**	**0,35945052**
M9_703_ncr2	M9_703_ncr2	2,560606928	5,899558236	2,89E-16	0,436195381	1,353031457	0,029815635	3,626294846
M9_711_shd4	M9_711_shd4	0,478132779	1,392939674	0,116219838	0,802754451	1,744428482	0,000621378	1,568684078
M9_715_-	M9_715_-	0,188542875	1,139612124	0,913953259	-0,04884847	0,96670763	0,561161896	1,053159877
M9_728_shd4	M9_728_shd4	0,232535537	1,17489802	1	0,080935539	1,057703703	1	1,116300862
M9_730_-	M9_730_-	0,75430583	1,686819763	0,001107597	-0,73467681	0,600952635	0,007643837	1,143886199
M9_731_shd4	M9_731_shd4	1,183231656	2,27084881	0,240177562	-0,64692571	0,638639765	0,343738036	1,454744287
M9_737_shd4	M9_737_shd4	0,116463009	1,084073827	1	0,080935539	1,057703703	1	1,070888765
M9_746_shd4	M9_746_shd4	0,615792109	1,532399127	2,31E-05	0,080935539	1,057703703	0,074593467	1,295051415
M9_764_-	M9_764_-	0,059524378	1,042122141	0,3507688	0,080935539	1,057703703	1	1,049912922

M9_770_-	M9_770_-	0,078283304	1,055761019	0,703452104	0,791789759	1,731220825	0,550069815	1,393490922
M9_773_-	M9_773_-	0,087869972	1,062799882	1	0,080935539	1,057703703	1	1,060251792
M9_782_ncr2	M9_782_ncr2	-0,92200583	0,527774725	0,000452431	-0,15444184	0,898479918	0,245544608	0,713127322
M9_790_-	M9_790_-	0,216462137	1,161880856	0,300325558	0,764121387	1,698335383	0,000338151	1,43010812
M9_806_-	M9_806_-	-0,03826516	0,973825266	0,11386629	-0,45676756	0,72861694	0,016606636	0,851221103
M9_808_-	M9_808_-	0,091084148	1,06517033	1	0,080935539	1,057703703	0,984343912	1,061437017
**M9_819_-**	**M9_819_-**	**1,269117334**	**2,410140639**	**2,30E-09**	**1,473594653**	**2,777129885**	**9,68E-11**	**2,593635262**
**M9_821_-**	**M9_821_-**	**2,006832597**	**4,018988913**	**4,71E-11**	**2,332853392**	**5,038007928**	**7,68E-16**	**4,52849842**
M9_827_-	M9_827_-	-1,62506883	0,324194421	0,00944533	0,103148904	1,074115328	0,341931342	0,699154874
M9_849_-	M9_849_-	0,180692387	1,133427716	1	0,080935539	1,057703703	1	1,09556571
M9_85_ncr3_	M9_85_ncr3_	0,755423069	1,688126562	0,003380322	0,177039965	1,130561885	0,312442749	1,409344223
M9_869_-	M9_869_-	-0,7522176	0,59369028	0,026141228	-1,07422316	0,474926723	4,30E-05	0,534308502
M9_872_-	M9_872_-	0,43378076	1,350768799	0,210227432	0,080935539	1,057703703	0,371493497	1,204236251
**M9_885_ncr3**	**M9_885_ncr3**	**-1,46858871**	**0,361335596**	**0,001447437**	**-2,05721989**	**0,240278607**	**1,38E-05**	**0,300807102**
M9_888_ncr3	M9_888_ncr3	-0,081712	0,944935658	0,351196644	0,957115469	1,941424319	0,000191114	1,443179989
M9_890_ncr3	M9_890_ncr3	0,611010044	1,527328131	1,33E-06	-0,77397895	0,584802364	0,153775523	1,056065247
M9_892_-	M9_892_-	-0,52025031	0,69725085	0,027009518	0,675639776	1,597304955	0,002319127	1,147277902
M9_897_-	M9_897_-	0,335674574	1,261967341	0,001674178	0,080935539	1,057703703	1	1,159835522
M9_90_-	M9_90_-	0,874032243	1,832778251	2,15E-05	-0,36905756	0,774288135	0,132667635	1,303533193
M9_910_-	M9_910_-	0,028787991	1,020154733	0,307550274	1,014667156	2,020436705	2,93E-06	1,520295719
M9_916_-	M9_916_-	-1,77841443	0,291503593	2,16E-11	-0,77982904	0,582435808	0,003237719	0,4369697
M9_941_-	M9_941_-	-0,84096001	0,558271955	0,001819879	-1,0488444	0,483355176	5,68E-07	0,520813566
M9_945_-	M9_945_-	0,660002527	1,580085392	0,540083308	0,080935539	1,057703703	1	1,318894547
M9_968_shd5	M9_968_shd5	1,022669441	2,031674723	0,001487081	0,16905057	1,124318334	0,278743078	1,577996528
M9_980_-	M9_980_-	0,439397072	1,356037497	0,011846275	-0,29270984	0,816367223	0,319870858	1,08620236
M9_989_-	M9_989_-	1,186106731	2,275378782	1,66E-07	0,016822698	1,011728855	0,706629825	1,643553819
M9_990_-	M9_990_-	-0,14492778	0,904424652	0,084027063	0,669636154	1,590671752	0,009993376	1,247548202
rnpB_401	rnpB_401	1,284657471	2,436242039	4,01E-07	-1,33454687	0,396516589	2,87E-07	1,416379314
scr_354	scr_354	3,308485801	9,90725785	4,91E-19	-1,38712685	0,382325451	7,92E-09	5,144791651

ssrSA_203	ssrSA_203	3,583418892	11,98716749	4,37E-20	-1,07127446	0,47589841	3,12E-06	6,231532952
ssrSB_201	ssrSB_201	3,988877544	15,87712229	1,24E-20	-1,34304704	0,394187234	1,18E-06	8,13565476
